# m^6^A RNA modification modulates PI3K/Akt/mTOR signal pathway in Gastrointestinal Cancer

**DOI:** 10.7150/thno.42971

**Published:** 2020-07-25

**Authors:** Qijie Zhao, Yueshui Zhao, Wei Hu, Yan Zhang, Xu Wu, Jianwei Lu, Mingxing Li, Wei Li, Weiqing Wu, Jianhong Wang, Fukuan Du, Huijiao Ji, Xiao Yang, Zhenyu Xu, Lin Wan, Qinglian Wen, Xiang Li, Chi Hin Cho, Chang Zou, Jing Shen, Zhangang Xiao

**Affiliations:** 1 Laboratory of Molecular Pharmacology, Department of Pharmacology, School of Pharmacy, Southwest Medical University, Luzhou, 646000, Sichuan, PR China.; 2 South Sichuan Institute of Translational Medicine, Luzhou, 646000, Sichuan, PR China.; 3 Department of Pathophysiology, College of Basic Medical Science, Southwest Medical University, Luzhou, 646000, Sichuan, PR China.; 4 Department of Gastroenterology, Shenzhen Hospital, Southern Medical University, Shenzhen, Guangdong, PR China.; 5 Department of Anaesthesia and Intensive Care, The Chinese University of Hong Kong, Hong Kong SAR, PR China.; 6 Department of Oncology, Jiangsu Cancer Hospital and Jiangsu Institute of Cancer Research and The Affiliated Cancer Hospital of Nanjing Medical University, Nanjing 210000, PR China.; 7 Clinical Medical Research Center, the Second Clinical Medical College of Jinan University, The First Affiliated Hospital of Southern University, Shenzhen People's Hospital, Shenzhen, Guangdong 518020, PR China.; 8 Department of Breast and Thyroid Surgery, the Second Clinical Medical College of Jinan University, The First Affiliated Hospital of Southern University, Shenzhen People's Hospital, Shenzhen, Guangdong 518020, PR China.; 9 Department of Pharmacy, Yijishan Affiliated Hospital of Wannan Medical College, Wuhu, Anhui, PR China.; 10 Department of Hematology and Oncology, The Children's Hospital of Soochow, Jiangsu, PR China.; 11 Department of Oncology, The Affiliated Hospital of Southwest Medical University, Luzhou, Sichuan 646000, PR China.

**Keywords:** m^6^A RNA methylation, gastrointestinal cancer, bioinformatics, PI3K/Akt signal pathway, mTOR signaling pathway

## Abstract

**Rationale:** Methylation at the N6 position of adenosine (m^6^A) is the most prevalent RNA modification within protein-coding mRNAs in mammals, and it is a reversible modification with various important biological functions. The formation and function of m^6^A are regulated by methyltransferases (writers), demethylases (erasers), and special binding proteins (readers) as key factors. However, the underlying modification mechanisms of m^6^A in gastrointestinal (GI) cancer remain unclear. Here, we performed comprehensive molecular profiling of the nine known m^6^A writer, eraser, and reader proteins in GI cancer.

**Methods:** Data from The Cancer Genome Atlas (TCGA) and Gene Expression Omnibus (GEO) were used. Gene alteration and pathway analysis were done in cBioportal. The protein network of m^6^A regulators and its related pathway members was analyzed in STRING online platform. Phylogenetic tree was constructed in MEGA7. m^6^A modification sites were predicted by SRAMP. m^6^A related SNPs were analyzed by m^6^ASNP. The modulation of m^6^A on its related pathway members was validated by m^6^A-seq, real-time PCR and phosphor-MAPK array.

**Results:** We found that m^6^A regulators were mostly upregulated in GI cancer and their differential expression significantly influenced the overall survival of patients with GI cancer. The phosphatidylinositol-3-kinase (PI3K)/Akt and mammalian target of rapamycin (mTOR) signaling pathways were found to be potentially affected by m^6^A modification in most human cancers, including GI cancer, which was further verified by m^6^A-Seq and phospho-MAPK array.

**Conclusions:** Our findings suggest that m^6^A RNA modification has a fundamental role in the regulation of PI3K/Akt and mTOR signaling pathway function in cancer.

## Introduction

**Figure 1:**
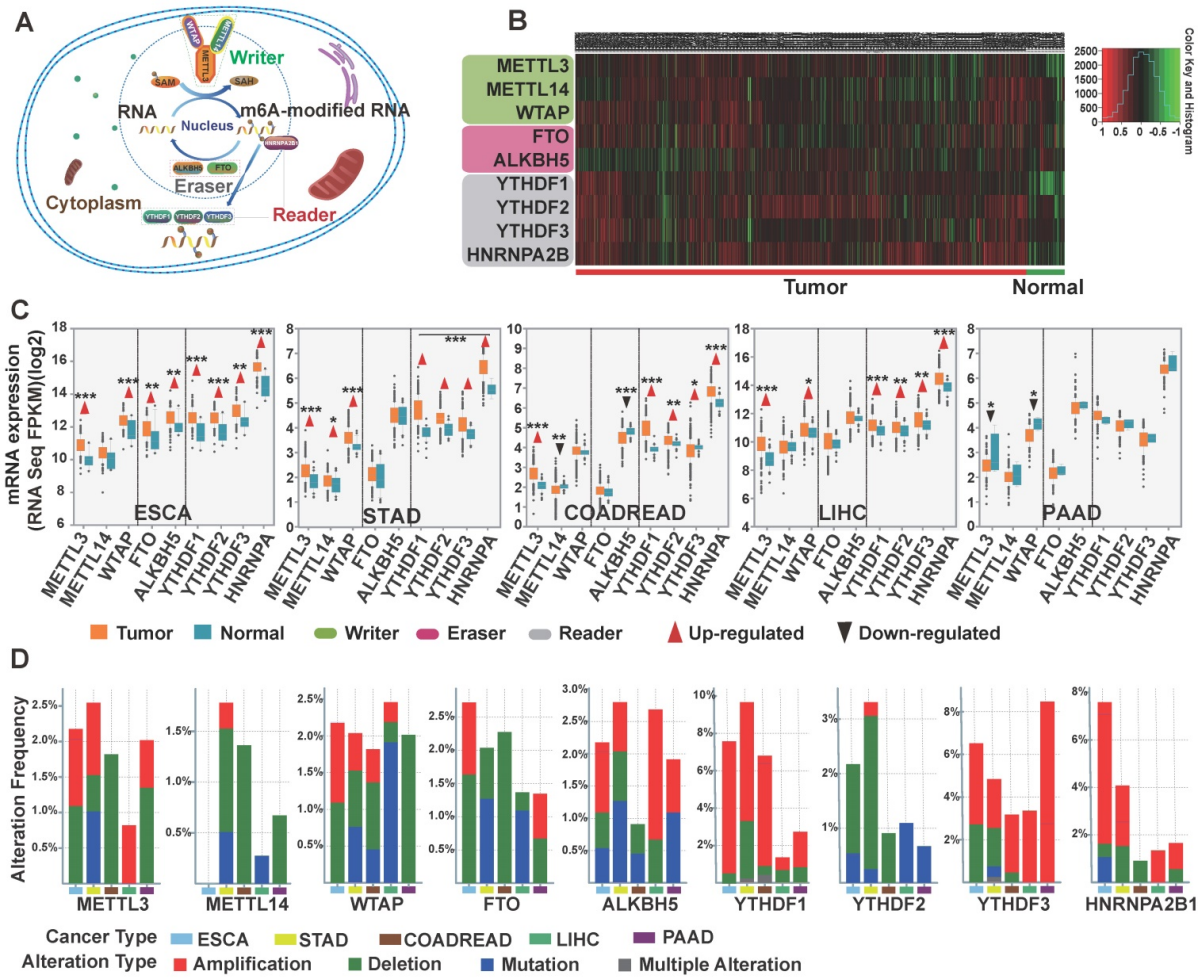
** Comprehensive molecular profiling of m^6^A writers, erasers, and readers in GI cancer.** (**A**) Schematic of eukaryotic cellular m^6^A methylatransferases (writers: METTL3, METTL14, and/or WTAP), demethylases (erasers: FTO and ALKBH5), and binding proteins (readers: YTHDF1-3, HNRNPA2B1). These proteins led to dynamic and reversible m^6^A modification of RNA. (**B**) Heatmap depicting the RNA expression profiles of the nine m^6^A regulators in GI cancer based on RNA sequencing data from TCGA database. Data were obtained from cancer patients (n=1727) and healthy patients (n=160), and each sample was normalized by counting the number of reads. The red and green regions represent higher and lower expression levels, respectively. (**C**) The expression level of the nine m^6^A regulators in different GI cancer types was analyzed using the RNA sequencing data from TCGA database consisting of 1727 cancer patients and 160 healthy patients. (**D**) The frequency of genetic alterations of m^6^A regulators in GI cancer. Cases with mutations, copy number alterations (amplification and deletion), and multiple alterations were selected from TCGA database and analyzed in cBioPortal. About 24.7% (46/186) of esophageal cancer, 26.8% (99/396) of stomach cancer, 13.7 (88/640) of colorectal cancer, 18.3 (67/366) of liver cancer, and 13.4(20/149) of pancreatic cancer clinical samples had genomic alterations of m^6^A regulators. (*p < 0.05, **p < 0.01, ***p < 0.001 between tumor and normal group).

N^6^ methylation of adenosine (m^6^A) is the most common reversible mRNA modification in eukaryotes since its emergence a decade ago 1, 2. It was recently recognized that reversible m^6^A modification influences mRNA fate in cells 3-6. Unlike DNA methylation and histone modification, RNA modifications have recently been emphasized as a new layer of epigenetic regulation at the post-transcriptional RNA level 7-9. All m^6^A have strict sequence specificity and no requirement for extended sequences or secondary structures, and only occur in a fraction of transcripts 2-5, 7-10. The cellular pathway of m^6^A modification of RNA is shown in (**Figure 1A**). m^6^A is catalyzed by a methytransferase complex consisting of the “writer” proteins methyltransferase-like 3 (METTL3), methyltransferase-like 14 (METTL14), and Wilms tumor 1 associated protein (WTAP). Its demethylation is catalyzed by two “eraser” demethylases, fat mass and obesity-associated protein (FTO) and AlkB homolog 5 (ALKBH5) 11-13. YTHDF1, YTHDF2, YTDHF3, YTHDC1, and NHRNPA2B1, members of the YTH domain family of proteins; are m^6^A “readers” that recognize its modification and affect pre-mRNA splicing as well as mRNA transport, stability, and translation 14, 15. The METTL3-METTL14 hetero methyltransferase complex (stoichiometric ratio=1:1) 16 interacted with WTAP to affect nuclear RNA m^6^A load (**Figure 1A**) 12. The writer proteins can colocalize the mammalian RNA methylation through m^6^A deposition 17. The methyl donor S-adenosylmethionine is catalyzed by writer proteins and is present during RNA methylation (Fig. 1a) 18. As a reversible internal modification, FTO and ALKBH5 catalyze the demethylation of m^6^A 5, 7-10, 19. The YTHDF1/2/3 reader proteins selectively bind m^6^A-containing mRNA in the cytoplasm of eukaryotes 20, and NHRNPA2B1 is widely distributed in the nucleus where it functions as a special m^6^A-binding protein 14. To date, more than 12,000 m^6^A sites in the transcripts of more than 7,000 mammal genes have been characterized by methylated RNA immunoprecipitation sequencing (MeRIP-Seq) 15, 21. RNA modification plays a crucial role in important biological processes in mammals. Recently, some key studies demonstrated that the alteration of core genes during m^6^A modification affects tumorigenesis, cancer cell proliferation, tumor microenvironment, and prognosis in cancers such as glioblastoma, lung cancer, liver cancer, and breast cancer 22-26. However, the biological function and critical target genes of these m^6^A regulators remain unknown for the majority of human cancers.

In the United States, there were about 310,000 new cases diagnosed of gastrointestinal (GI) cancer in 2017 and 2018, including cancers of the esophagus, stomach, rectum, and colon, as well as cancers of the liver, pancreas, and gallbladder, and more than 150,000 deaths occurred 27. GI cancer shares several characteristics such as similar endodermal development, high mortality, influenced by multiple factors, and chromosomal instability 28, 29. In recent years, genetic alterations, single nucleotide polymorphisms (SNPs), and epigenetic modification have been increasingly recognized as important regulators of GI cancer 30. However, there has been little progress towards understanding the biological functions and mechanisms of m^6^A in malignant tumors 31. To gain further insight into the role of m^6^A in tumors, we studied the correlation between five GI subtypes (esophageal, stomach, colorectal, liver, and pancreatic) and m^6^A writer, reader, and eraser proteins. To this end, bioinformatics analysis of tumor profiles obtained from The Cancer Genome Atlas (TCGA) and Gene Expression Omnibus (GEO) databases and comprehensive molecular profiling of the m^6^A regulators in GI cancer were performed. The results showed that genomic alterations in m^6^A caused significant changes in the overall survival (OS) of patients with GI cancer. The identification of risk factors for GI cancer may help with the development of targets and early detection and therapeutic strategies for the treatment of cancer.

## Methods

### Data Processing

We started with a set of 10,037 samples that were included in the final whitelist for the TCGA (http://cancergenome.nih.gov). There were 1920/10037 (19.1%) GI cancer samples. After filtering with eligibility formats, a total of 9,628 samples were used for pathway alteration analysis. Gene expression (generated by RNA-sequencing) was downloaded using the bioinformatics tool. Protein enrichment data (generated by Reverse phase protein array) were analyzed using cBioportal pipeline (http://www.cbioportal.org/). RNA-Sequence data was processed using Fragments per Kilobase of transcript per Million fragments mapped (FPKM). The American, East Asian, and European Genomic SNPs were collected from 1,000 Genome Project Data.

### Pathway Analysis

Proteomic data were collected by Reverse Phase Protein Array (RPPA) based on TCGA data and analyzed by cBioportal in 27 kinds of human cancers. RPPA quality control and methodology stander were previously explained 32. For the enriched proteins, significant change in expression was determined by the standard of a log ratio of Log2 based ratio (μ mean altered/ σ mean unaltered) (log>0 for overexpression and log<0 for underexpression) and queried event results *p* value<0.05. The selected proteins from this criterion were used to predict pathways by two conditions: (a) the sum of altered protein in each pathway, and (b) the statistical *P*-value score of significant pathway. DAVID function annotation tool (https://david.ncifcrf.gov) is a method that infers conditional dependencies between target genes and KEGG pathway. The significance of pathway level association was empirically estimated by *P*-value score and enriched counts, and the estimating score threshold was restricted with FDR (False Discovery Rate) of Benjamini-Hochberg method. We still considered the corrected *P* value smaller than 0.05 based on:

Q-value(i)=P(i)*length(P)/rank(P)

### Correlation Analysis

The gene co-expression and DNA methylation correlation in GI cancer from TCGA data were analyzed in cBioportal and Prism6 with Pearson and Spearman correlation significances. In human, pathway gene co-expression was analyzed utilizing NCBI GEO microarray expression experiments deposit. The score from single- and dual-channel arrays (thresholds of 0.75 and 0.95) of gene-to-gene Pearson correlation were combined to get final scores, and calibration was depended on KEGG benchmark improvement in STRING 33. The PPI network among the final cluster of genes was derived from the STRING online platform (http://string-db.org). STRING is a widely used database and online resource devoted to investigating PPI, including physical and function interaction 34. All of the associations available in STRING were denoted with a probability confidence score. Interaction with a confidence score greater than 0.4 was selected to construct the PPI network. To define the phylogenetic characterization of target genes, a total of 66 whole genome sequences were recruited from GenBank for the phylogenetic tree construction. Neighbour-Joining (NJ) algorithm in MEGA7 was employed, respectively, with random addition of taxa and tree-bisection reconnection branch swapping.

### m^6^A Methylation Site Prediction

Genetic mRNA sequence was used in m^6^A prediction tool SRAMP. Results were based on three type random forest classifiers, namely, Binary (Positional binary encoding of nucleotide sequence), KNN (K-nearest neighbor) and Spectrum (Nucleotide pair spectrum). Positive samples (with m^6^A sites) were retained by DRACH (D=A, G or U; R=A or G; H=A, C or U) consensus motif and 10-fold likelihood to correct proof negative sample (non-m^6^A sites) to avoid prominent bias, keeping the 1:10 positive-to-negative calculate ratio. For multiple transcripts with same locus, only the longest transcript with the largest number of m^6^A sit was retained. The different prediction scores of random forest classifiers were combined using the weighted summing formula:


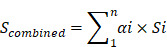


### RNA Sequencing Data Analysis

In order to confirm the m^6^A function in human cancer, we applied the MeT-DB v2.0 bioinformatics tool with different Input FASTQ file from immunoprecipitation experiment. High-throughput sequencing data of expression profile after METTL3 knockdown for U2SO cells (GSE48037), Hela cells (GSE46705), HEK293T cells (GSE55572), and A549 cells (GSE76367) was obtained from Gene Expression Omnibus (GEO). Pathway member gene symbols with its chromosomal start/end position were used to query based on NCBI GEO high-throughput Input data. For each peak/site, the genomic location, gene ID, and read counts were displayed in the column of the newly lightweight JBrowse. Interesting target genes were screened by JBrowse track filtering panel. The target genetic exon peak region output were reconstructed and calculated within Origin 9.

### m^6^A SNP Analysis

Genetic variant files variant call format (VCF) from the 1,000 Genome Project database was used in m^6^ASNP, a new m^6^A modification site predictor. There were 55,548 human m^6^A sites according to the literature 35, 36. The sequences of 30 nucleotides upstream and downstream of m^6^A residue in the flanking regions were extracted for random forest prediction. Genetic variants were first mapped to known transcript structures. Based on PA-m^6^A-seq, MeRIP-seq, and miCLIP-seq data in m6ASNP, a total of 13,703 high-confidence, 54,222 medium-confidence, and 243,880 low-confidence m^6^A-associated variants were found for human. To avoid any bias, the deleteriousness of each variant score was aggregated from 0 to 5 using the five kinds of software. LRT 37, PolyPhen2 HVAR 38, PolyPhen2 HDIV 38, SIFT 39, and FATHMM 40, with a higher score indicating higher possibility of deleteriousness.

### m^6^A-seq coverage analysis

The m^6^A-seq was performed as previously described 41. Fragmented RNA (5μg mRNA) was incubated for 2 h at 4°C with 10 μg of affinity purified anti-m^6^A polyclonal antibody (Synaptic Systems) in 450 μL of immunoprecipitation (IP) buffer (150 mM NaCl, 0.1% NP-40 (v/v), 10mM Tris-HCl, pH 7.4) and 300 U mL^-1^ RNase inhibitor (Promega). The mixture was then immunoprecipitated by incubation with protein-Abeads (Repligen) at 4°C for an additional 2h. Then, the eluted mRNAs were re-covered with phenol-chloroform extraction and ethanol precipitation in IP buffer. 50 ng of immunoprecipitated mRNAs and pre-immunoprecipitated mRNAs (input control) was used for library construction with NEBNext ultra RNA library prepare kit for Illumina (NEB). High-throughput sequencing was performed on the Illumina HiSeq X sequencer with a paired-end read length of 150 bp according to the standard protocols. From the smoothed mountain plots for both the control and IPed data, we compute the potential m^6^A situs for any given site on a gene (e.g., an DRACH position).

### Mammalian Cell Culture, siRNA Knockdown

Human gastric cancer cell TMK1, pancreatic cancer cell PANC-1, liver cancer cell Huh-7 were grown in DMEM media (Gibco) with 10% FBS (Gibco) and 1% 100X Pen/Strep (Gibco). Human colorectal cancer cell line HCT116, and esophageal cancer cell TE-1 were grown in IMEM and RPMI1640 media, respectively, with 10% FBS and 1% 100X Pen/Strep. Control siRNA and METTL3 siRNA were purchased from GenePharma (Shanghai, China) with reported sequences^12^. Transfection was achieved by using Lipofectamine Lipofectamine 3000 (Invitrogen) with 20 nM of siRNAs. At 48 h post-transfection, the mRNA levels of METTL3, AKT1, mTOR, PIK3CA, and PTEN were checked by Q-PCR.

### m^6^A RNA Methylation Quantification

Cancer cells were treated with SAH or MA for 48 h, then total RNAs were extracted by TRIZOL, concentrations of RNAs were analyzed by Nanodrop 2000. Then, 200 ng of RNA for each sample were used for m^6^A RNA methylation analyses. m^6^A levels were analyzed by relative quantification method according to the manufacturer's instruction (Abcam, ab185912).

### Real-Time PCR

Total RNA was isolated from cultured cells using TRIzol Reagent (Thermo Fisher), and cDNA was made with the FastKing RT Kit (With gDNase, KR116). The RNA concentration was measured by NanoDrop. Total RNA isolation for Quantitative Real-time PCR (qPCR) was performed using the SuperReal PreMix Plus (SYBR Green, FP205) according to the manufacturer's instruction.

### mRNA Lifetime Measurements

The half-life time of mRNA was measured using reported method 12, 42. In brief, HCT116 and TMK1 cells were treated with SAH or MA for 48 h, then actinomycin D (ActD, 5 μg/ml, Millipore) was used to treat cells for 6 h, 3 h, and 0 h. Total RNAs were isolated, and reverse-transcripted into cDNA, the mRNA levels of AKT1, mTOR, PIK3CA, and PTEN were detected by qPCR, and the mRNA half-life time of these genes were calculated using reported method 12.

### Human Phosphor-MAPK Array

Phosphorylation of PI3K/Akt and mTOR signaling effectors were detected using human phosphor-MAPK array kit (R&D, USA) following protocol provided by manufacturer. The signals were detected by the ChemiDoc^TM^ Imaging System and quantified using the software Image-Pro plus 6.0.

### Statistical Analysis

Students' t-test was used in comparison of two groups and one-way ANOVA was applied to compare multiple groups. Overall survival analysis was performed with the Kaplan-Meier curve with *P* value calculated using the log-rank test.

## Results

### Deregulation of m^6^A Writers, Erasers, and Readers in GI Cancer

First, we used TCGA database to investigate the profile of m^6^A writers, erasers, and readers, including METTL3, METTL14, WTAP, FTO, ALKBH5, YTHDF1, YTHDF2, YTHDF3, in GI cancer. After comparing the expression pattern of m^6^A-related genes between 1,782 cancer samples and 148 normal control samples, heatmap analysis showed that the expression of m^6^A regulators was remarkably higher in GI cancer (**Figure 1B**). Further comparison between cancer and normal samples in the five subtypes of GI cancer (esophageal, stomach, colorectal, liver, and pancreatic) (**Figure 1C**) indicated that readers and writers were mostly upregulated in GI cancer compared to the normal control, except in pancreatic cancer. It is worth noting that nearly all m^6^A-related genes were overexpressed in esophageal cancer while such overexpression was not seen in pancreatic cancer. On the contrary, two of the writers were significantly downregulated in pancreatic cancer (**Figure 1C**). We further analyzed genetic alterations of m6A regulators using the cBioPortal and applied Significant Targets in Cancer algorithm (GISTIC 2.0). The frequency of copy number amplification, deletion and genetic mutation of m6A regulators is shown in **Figure 1D**. Overall, about 11.9% of the clinical samples (204/1710) had genetic amplification of m6A regulators, and nearly 4.6% of the samples (79/1710) had genomic mutations. Of note, some m^6^A-binding proteins (readers), YTHDF1, YTHDF3, and HNRNPA2B1, showed relatively higher frequency of copy number amplification.

### Impact of m^6^A Regulator Alterations on the Survival Rate of GI Cancer Patients

**Figure 2:**
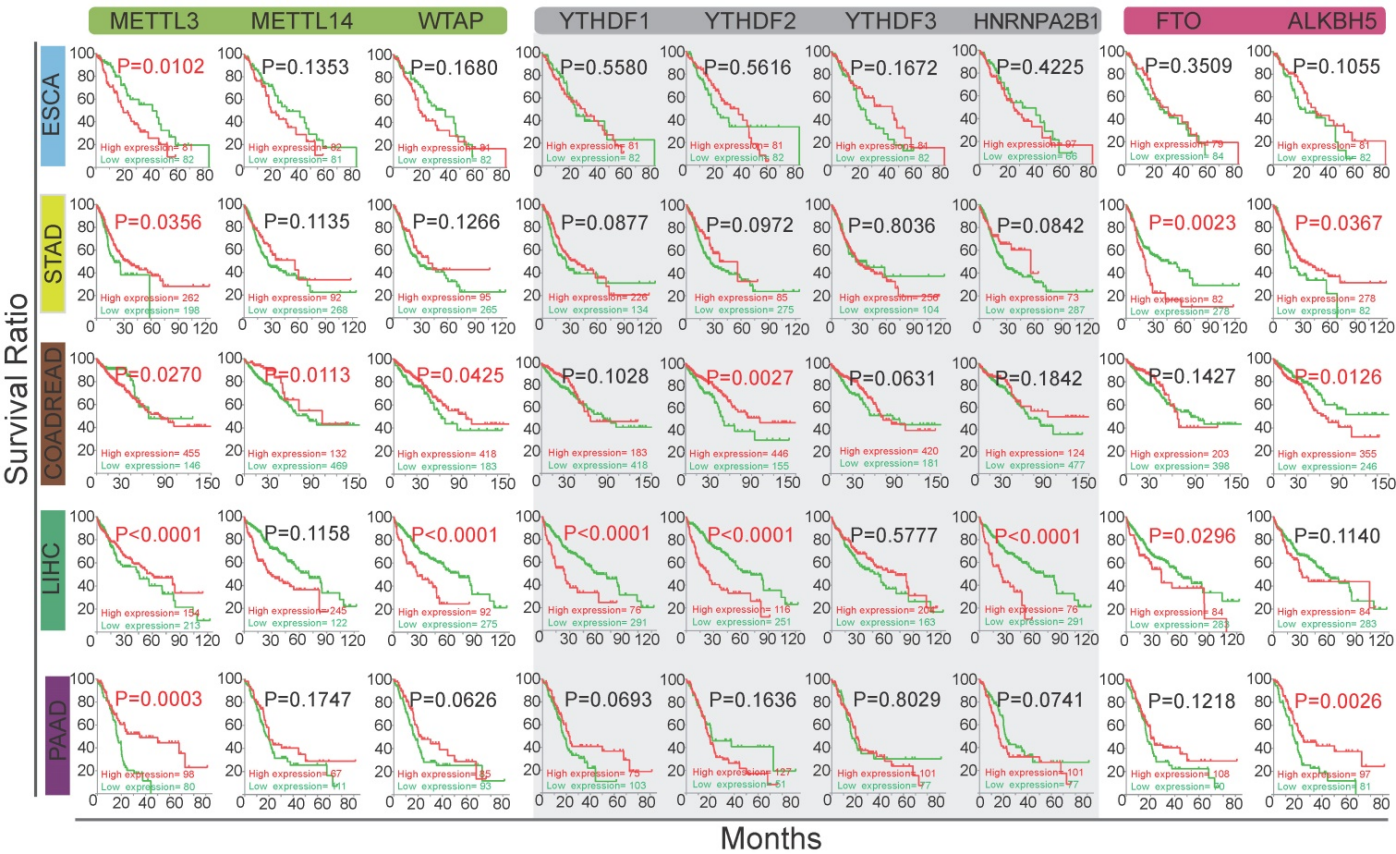
** The relationship between the alterations of m6A methylation regulators and the overall survival rate of GI cancer patients.** Kaplan-Meier analysis of overall survival of GI patients based on the expression level of m^6^A regulators. The red and green curves indicate the survival curves of patients in the high expression and low expression groups, respectively.

Next, we performed OS analysis on m^6^A regulators in five subtypes of GI cancer using TCGA database (**Figure 2**). Kaplan-Meier analysis revealed that the differential expression of m^6^A regulators was significantly associated with OS. Specifically, high expression of METTL3 was significantly associated with poor survival in esophageal and colorectal cancers (*p*<0.05). In contrast, low expression of METTL3 was significantly associated with poor survival in gastric cancer, liver cancer and pancreatic cancer. High expression of ALKBH5 was significantly associated with low OS in colorectal cancer while it was associated with better survival in stomach and pancreatic cancer. In liver cancer, high expression of writer, reader, and eraser proteins was associated with poor survival except METTL3, YTHDF3 and ALKBH5. Together, these results indicate that m^6^A regulators may have important influence in GI cancer survival.

### Identification of Signaling Pathways Associated with the Deregulation of m^6^A in GI Cancer

**Figure 3:**
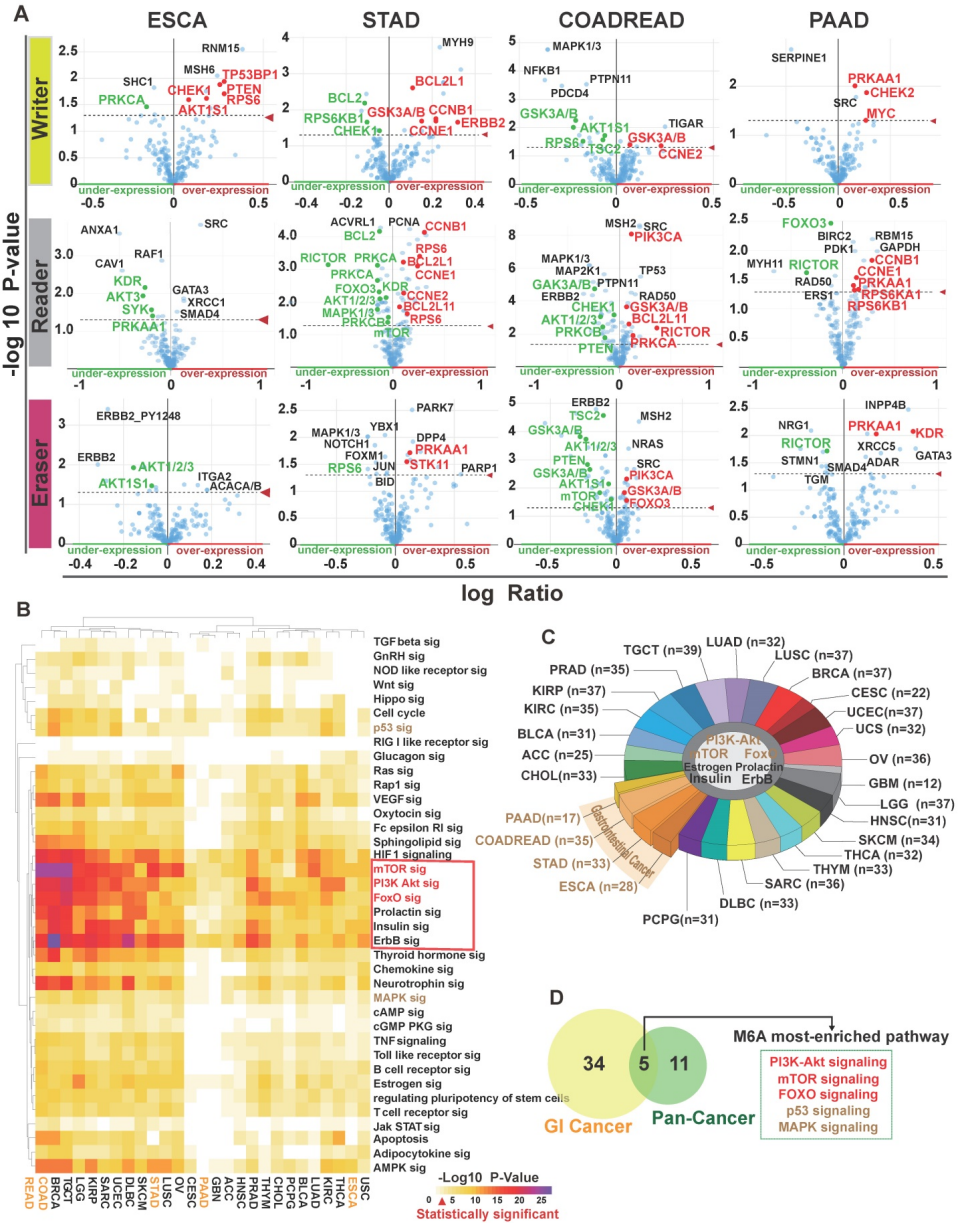
** The signaling pathways associated with the deregulation of m^6^A in GI cancer.** (**A**) Protein enrichment in esophageal, stomach, colorectal, and pancreatic cancer upon alteration of writer, eraser, or reader proteins. p<0.05 was considered significantly changed. The fold change in expression level was based on the log ratio (mean of altered expression/mean of unaltered expression). (**B**) Heatmap of pathways related to m^6^A gene alterations in 27 human cancers was analyzed by KEGG pathway analysis via DAVID. The degree of correlation was denoted by P values in different cancer types. (**C**) Pathway enrichment in different cancer types. Seven pathways were universally altered by m^6^A in human cancers. (n=number of pathways altered in each type of cancer.) (**D**) Identification of five m^6^A regulator-related pathways in cancer. Overlap was seen among 39 m^6^A regulator-related pathways and 16 cancer pathways from KEGG. The overlap resulted in five pathways that was represented in the Venn diagram.

As m^6^A modification is frequently involved in both mRNA metabolism and translation to influence protein expression levels 43, we tried to find out proteins associated with m^6^A regulators. We applied correlational analysis of protein expression (both parent and phosphorylated proteins) changes with m^6^A regulatory gene alterations (mutations, copy number alterations, mRNA expression changes) in GI and other cancers in cBioportal. The main proteins with significant changes (*p*<0.05) are shown in **Figure 3A and Table S1**. Then, we used DAVID to analyze these proteins in 27 individual human cancers relying on the KEGG pathway database (**Table S2**). 41 different pathways were shown with *P-*value scores in heatmap (**Figure 3B**). The enriched proteins were related to several key pathways, including the phosphatidylinositol-3-kinase (PI3K)/Akt, mammalian target of rapamycin (mTOR), class O of forkhead box transcription factors (FoxO), prolactin, estrogen, insulin, and ErbB signaling pathways, which typically exist in 27 major types of human cancers (**Figure 3C**). Importantly, five pathways (PI3K/Akt, mTOR, FoxO, mitogen-activated protein kinase [MAPK], and p53) that were positively associated with m^6^A modification in GI cancer, overlapped with KEGG cancer pathways (**Figure 3D**).

### Deregulation of PI3K/Akt, FoxO, mTOR, MAPK and p53 Signaling Pathways in Cancer and Their Relationship with m^6^A Members

**Figure 4:**
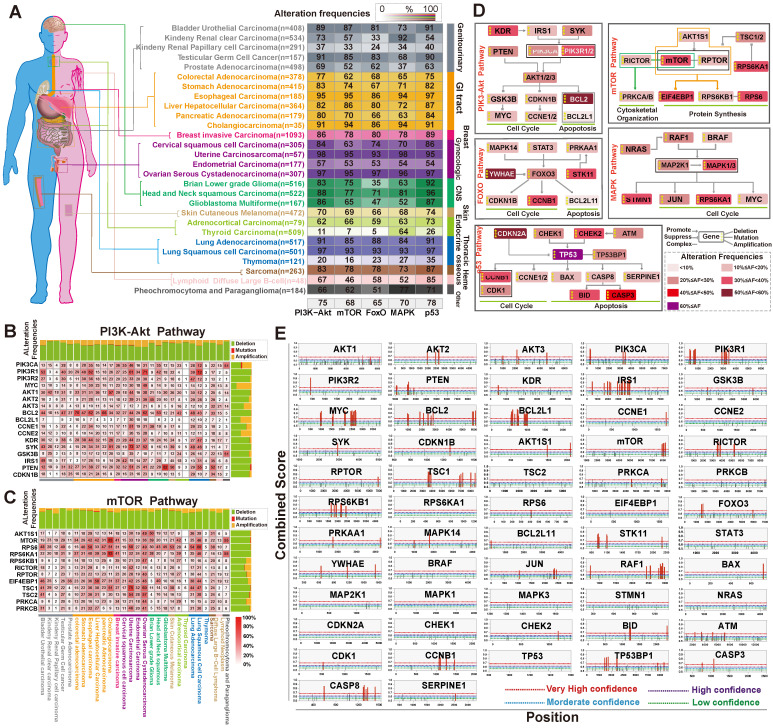
** The relationship between the selected signaling pathways and m^6^A regulators.** (**A**) The alteration of PI3K/Akt, mTOR, FoxO, MAPK and p53 pathways in different types of human cancer. Analysis was carried out in cBioPortal for Cancer Genomics using TCGA data. (**B,C**) Detailed heatmap of the alteration frequency in the PI3K/Akt and mTOR pathways. (**D**) Pathway diagram showing the frequency and type of alteration, cellular function, and interaction with other genes of the key genes in the five pathways. Different alteration frequencies were indicated by the color intensity of each gene from the entire GI cancer database. (**E**) m^6^A modification sites in the main pathway member mRNA sequences were predicted by SRAMP. The three random forest classifiers prediction scores were combined as combined score with weighted summing formula. The combined scores were divided into four degrees (very high confidence, high confidence, moderate confidence, low confidence).

Next, to assess alterations of PI3K/Akt, FoxO, mTOR, MAPK, and p53 signaling in cancer, we collected data of somatic mutations and DNA copy number variations (amplification, shallow deletion, and deep deletion) from tumor patients (n=9,628) from 27 different cancer types in TCGA through cBioPortal for Cancer Genomics 44. For each cancer type, target genes with at least one alteration were regard as being altered in a given pathway if one or more genes were involved in the pathway. As shown in **Figure 4A and Table S3**, the PI3K/Akt pathway showed the highest frequency of alterations (75%) in 27 human cancer types. The alteration frequency of GI cancer subtypes in this pathway were esophageal cancer (95% of patients), stomach cancer (83% of patients), colorectal cancer (77% of patients), liver cancer (82% of patients), and pancreatic cancer (80% of patients). The mTOR pathway also had a relatively high rate of alterations in GI cancer (75%), with an alteration frequency of 95%, 74%, 62%, 86% and 70% among the five cancer subtypes, respectively (**Figure 4A**). The FoxO, MAPK, and p53 signaling pathways also displayed high alteration frequency (**Figure 4A**). Further heatmap analysis indicated that the main genes in these five pathways had high frequency alterations across 27 cancer types (**Figure 4B-C, Figure S1A-C**). PIK3CA, PIK3R1, AKT1, PTEN, BCL2, and SYK in the PI3K/AKT pathway were among the most frequently altered genes (>20% of all patients; **Figure 4B**). In the mTOR pathway, the alteration of crucial members was the highest in GI and development tract cancer (43.4% across patients) than in other tumor types (**Figure 4C**). A simplified pathway diagram in **Figure 4D** shows the most frequently altered genes in GI cancer that were affected by m6A directly or indirectly in five pathways including the frequency and type of alteration, cellular function, and type of interaction with other genes in the pathway.

To get an overview of whether the five pathways are regulated by DNA methylation in tumor and normal samples, we used DiseaseMeth version 2.0 for further analysis based on dataset in TCGA HumanMethylation450. The expression of TP53, AKT3, PIK3R1, CHEK1, PRKAA1, and RAF1 were affected by DNA methylation in GI cancer (**Figure S3**). Interestingly, STRING analysis 33 of the protein-protein interaction (PPI) networks among the five pathway members (n=57 genes) and m^6^A regulators (writers: METTL3, METTL14 and WTAP; readers: YTHDF1, YTHDF2, YTHDF3, HNRNPA2B1 and erasers: FTO and ALKBH5) revealed that the node in **Figure S4A** could be classified into two large groups: one group consisted of m^6^A regulators and the other group was connected by the interaction of the five pathway proteins. AKT1 functionally interlinked the pathway proteins with the FTO (eraser) and HNRNPA2B1 (reader) proteins. The proxy of co-expression is a strong indicator of functional correlation 45. We found that there was strong similarity in expression between METTL14 (writer) and PIK3CA (score=0.195), suggesting functional correlation between the two proteins (**Figure S4B**).

To further explore the structural interlinks within the m^6^A regulators, we constructed a Phylogram tree of all genes (n=66) based on the nucleotide sequences (**Figure S4C**). In the tree, the length of branches was calculated from the likelihood ratio mapping the evolutionary messages among nuclear factors. The Phylogram tree showed a strong relationship between AKT1 and YTHDF1 (reader), indicating that they may have been a common ancestry or the same point of evolutionary origin. Moreover, the PI3K/Akt/mTOR pathway members (PIK3CA, AKTIS1, AKT2, AKT3, RPS6KB1, GSK3B) and m^6^A regulators (METTL3, METTL14, WTAP, FTO, YTHDF3, HNRNPA2B1) composed a monophyletic clade indicating a common ancestor. Moreover, we performed m^6^A site prediction. The special DRACH (D = A, G or U; R = A or G; H = A, C or U) and GAC consensus motif may be recognized as m^6^A sites and calculated by two random forest prediction modes of SRAMP to improve identity thresholds 46. As shown in **Figure 4E and Table S4**, very high confidence m^6^A sites universally existed in members of the PI3K/Akt (AKT1/2/3, PI3KCA, PTEN, PI3KR1/2, BCL2, BCL2L1, IRS1, GSK3B, MYC) and mTOR pathways (mTOR, AKT1S1, TSC1, RICTOR, RPTOR, RPS6KB1, PRKCA), as well as members from the FoxO, MAPK, and p53 pathways.

Taken together, these results indicated that m^6^A plays a fundamental role in the regulation cancer pathway such as the PI3K/Akt/mTOR pathway.

### Impact of m^6^A Deregulation on the PI3K/Akt/mTOR Pathway in GI Cancer

**Figure 5:**
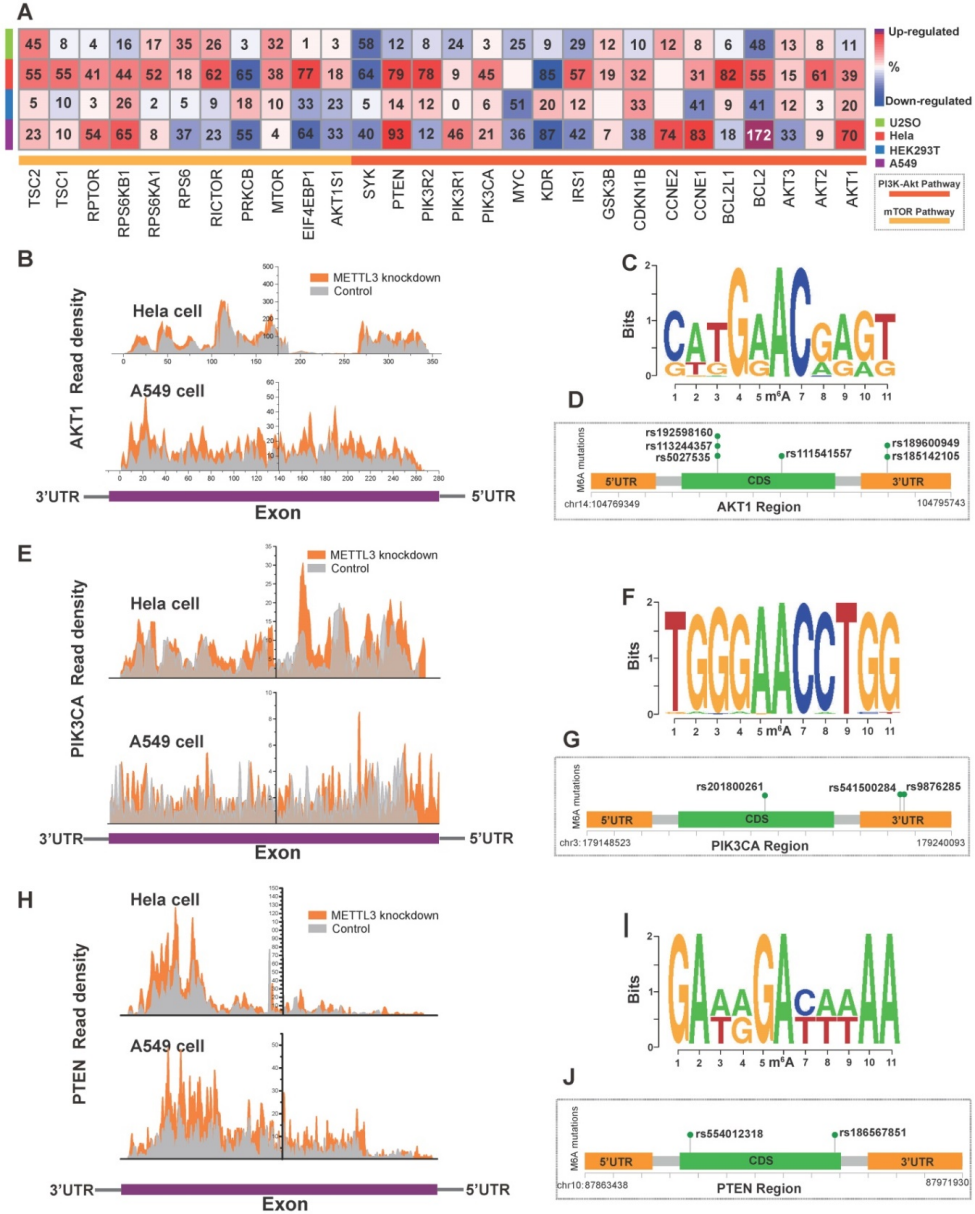
** The modulation of m^6^A regulators on PI3K/Akt/mTOR pathway members**. (**A**) Alteration in expression of PI3K/Akt and mTOR pathway genes in U2SO, Hela, HEK293T and A549 cells upon METTL3 knockdown based on the MeT-DB V2.0 and GEO databases (GSE48037, GSE46705, GSE55572, and GSE76367). (**B, E, H**) The transcript reads coverage peaks visualized exon region expression of AKT1, PIK3CA, and PTEN upon METTL3 knockdown in Hela and A549 cells. Figure was reconstructed by Origin 9. (**C, F, I**) To present the SNP alteration of m^6^A motif, the sequence logo was generated automatically based on DRA(m^6^A)CH structure with the 1000 Genome Project database SNP. (**D, G,** J) Recurrent known SNP mutation relevant to m^6^A modification in AKT1, PIK3CA, and PTEN. Recurrent known SNP mutations were color coded in green, and each mutation was annotated with dbSNP label. Distribution of m^6^A-related SNP variants within the gene across their 3'-UTR, 5'-UTR and exon regions was shown.

We found co-occurrence of PI3K/Akt and mTOR pathway alteration in cancer (**Figure S2A**) and the PI3K/Akt and mTOR pathway were interconnected (**Figure S2B**). To investigate the direct effects of m^6^A on the PI3K/Akt/mTOR pathway, we first employed MeT-DB V2.0 47 to detect genomic expression profiles based on the GEO database. Since the presence of m^6^A can induce mRNA degradation in eukaryotes 48, we focused on upregulated genes with m^6^A writer downregulation based on the GEO database. Knockdown of the “writer” METTL3 in U2SO cells (GSE48037), Hela cells (GSE46705), HEK293T cells (GSE55572), and A549 cells (GSE76367) resulted in a relatively elevated expression of PI3K/Akt/mTOR signaling pathway proteins such as AKT1, AKT2, PTEN, PIK3CA and BCL2 as shown in the heatmap (**Figure 5A, Figure S4D**). The gene read density of AKT1, PTEN, and PIK3CA in Hela and A549 cells was shown in **Figure 5B, E and H**, A recent study indicated that post-transcription is influenced by genomic variants and is closely related to multiple disease such as cancer and genetic diseases 49. To investigate the dysregulation of PI3K/Akt pathway in relation to the abnormity of variants in critical gene site, we analyzed SNPs from the 1000 Genomes Project database (among AMR, EAS, and EUR). Then we applied m^6^A SNP to detect the m^6^A site that is altered by variants (nonsynonymous and synonymous) around these sites 50. The DRACH motif of AKT1 mutation sequence is shown in **Figure 5C**. We identified SNPs (rs185142105, rs189600949, rs5027535, rs113244357, rs192598160 and rs111541557 with dbSNP annotation) variants in AKT1 that disrupted m^6^A modification in the same or diverse sites (**Figure 5D**). The same phenomenon was found in PIK3CA (**Figure 5F-G**) with rs201800261, rs541500284, and rs9876285 and PTEN with rs554012318 and rs186567851 (**Figure 5I-J**) and AKT1S1 with rs546549861 (**Figure S4E-G**).

### *In vitro* Study of m^6^A Deregulation on the PI3K/Akt/mTOR Pathway in GI Cancer

**Figure 6:**
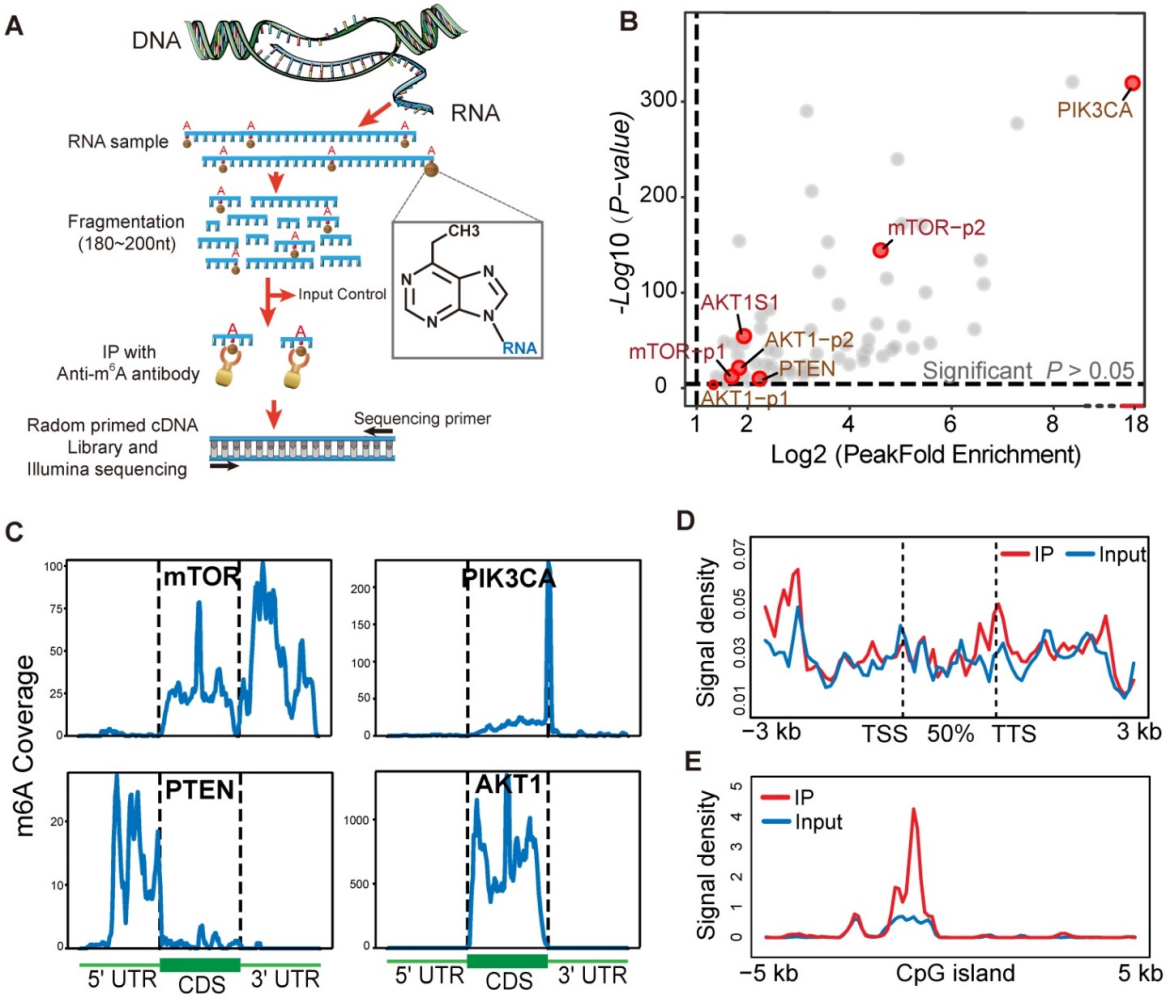
** m6A-seq capture of modified RNA in PI3K/Akt/mTOR pathway members.** (**A**) Schematic diagram of the immunoprecipitation with high-throughput RNA sequencing. (**B**) Scatter plot of m^6^A Peak enrichment fold change in TMK1 cell. Within part of PI3K/AKT/ mTOR pathway genes, AKT1 mTOR, PTEN and PIK3CA show a significant m^6^A peak enrichment. (**C**) Representative gene transcripts harbouring m^6^A peaks in TMK1 cell. Black dashed lines signify CDS borders and flanking thin parts corresponding to UTRs. Uniquely, peak signal summarizing were divided into 100 bin for each regions. (**D**) Distribution of AKT1 mTOR, PTEN and PIK3CA-binding peaks across gene bodies. The signal density of m^6^A signaling was estimated on these gene bodies (between transcription start site, TSS and transcription termination site, TTS), as well as 3-kb upstream of TSS and 3-kb downstream of TTS regions. (**E**) Enrichment of m^6^A-binding peaks on CpG islands (CpGI). The signal density of m^6^A-binding peaks were estimated on CpG islands and 5-kb side regions.

To identify and localize m^6^A sites at a transcriptome-wide level we applied m^6^A-seq to RNA purified from a human gastric cancer cell line (TMK1). Libraries were prepared from immunoprecipitated as well as input control fragments, and subjected to massively parallel sequencing (**Figure 6A**). This analysis yielded ~20,000 putative m^6^A sites peak (**Figure S5A**) and the overlapping transcript can be divided into five segments, TSS, 5' UTR; coding sequence (CDS); stop codon; and 3'UTR (**Figure S5B-C**). mTOR, AKT1, PIK3CA and PTEN were identified to possess a sequence element for specifying m^6^A RNA-methylation (**Figure 6B**). In mTOR and PIK3CA, the 3' UTR and contiguous segment stood out as most enriched in m^6^A peaks (**Figure 6C**). Moreover, PTEN showed m^6^A peaks enrichment near the 5' UTR region, and a corresponding decrease of m^6^A levels after the start codon (**Figure 6C**). AKT1 m^6^A modification predominantly target codon region. Furthermore, in these four genes, the average binding location analysis shows that m^6^A-binding activity a little bit higher in transcription termination sites (TTS) (**Figure 6D**). As shown in **Figure 6E**, mTOR, AKT1, PIK3CA and PTEN m^6^A modification peak was obviously enriched at the centre of CpGI (CpG islands). m^6^A/MeRIP-seq of the polyA^+^ RNA from human TMK-1 cell identified multiple peaks of varying intensities and one m^6^A-consensus sequences consisting of DRACH was present in the largest peak of mTOR and AKT1 (**Figure S5G**). This result may explain the structural context of the PI3K/AKT/mTOR pathway alteration by m^6^A methylation sites. The TMK1 cell genome m^6^A modification distribution landscape was showed in supplementary (**Figure S5D-F**).

**Figure 7:**
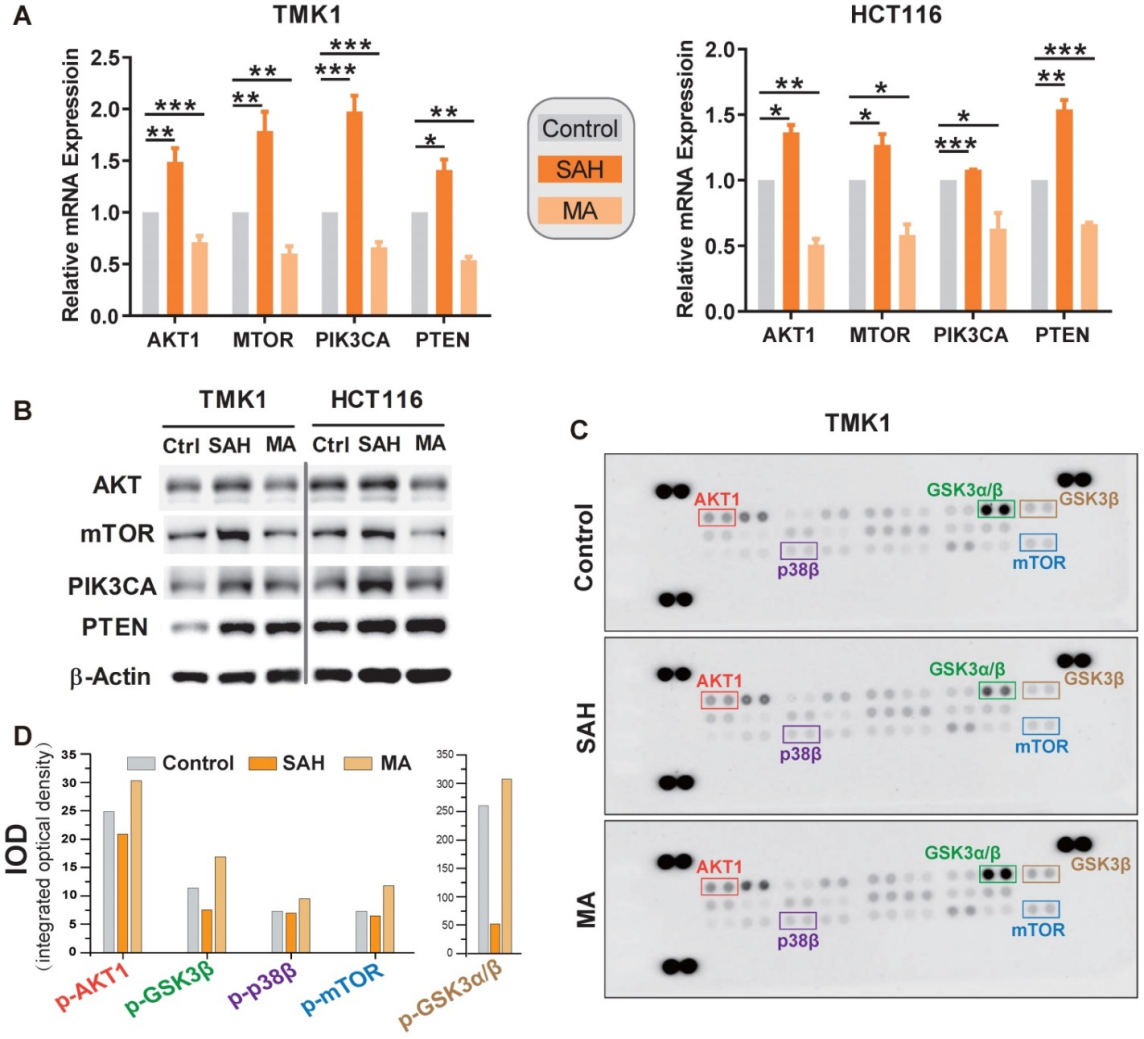
** Validation of m^6^A regulation on PI3K/Akt/mTOR pathway.** (**A,B**) mRNA expression level of m^6^A candidate targets AKT1, PTEN, PIK3CA, and mTOR after m^6^A manipulation in stomach cancer cell TMK1 and colon cancer cell HCT116. Expression mRNA levels and protein levels of target genes were measured by qPCR and by western blotting, respectively, after treatment with 1 μM of METTL3-METTL14 inhibitor S-adenosylhomocysteine (SAH) or m^6^A demethylase FTO inhibitor meclofenamic acid (MA) for 48 h. *P<0.05, **P<0.01, ***P<0.0001 compared with control. (**C**) m^6^A-related phosphorylation of PI3K/Akt/mTOR signaling effectors. Human phosphor-MAPK array was used to detect the phosphorylation of PI3K/Akt/mTOR signaling effectors in TMK1 cell. Cells were treated with culture media, 1µM SAH or 1µM MA for 48 h. (**D**) Quantification of protein phosphorylation for the phosphor-MAPK array. Integrated optical density was determined by densitometry analysis of the spots using Image-Pro Plus software.

Finally, we validated whether m^6^A deregulation causes alterations in the PI3K/Akt/mTOR signaling pathway in GI cancer by inhibiting the activity of the methyltransferase (METTL3-METTL14 complex) with S-adenosylhomocysteine (SAH) or by METTL3 siRNA, and by inhibiting the activity of demethylase (FTO) with meclofenamic acid (MA). m^6^A levels were deregulated in GI cancer cells under SAH or MA treatment (**Figure S6A**). The mRNA and protein levels of PTEN, AKT1, PIK3CA, and mTOR were significantly upregulated under SAH treatment in GI cancer cells, including gastric cancer cell TMK1, colorectal cancer cell line HCT116, esophageal cancer cell TE-1, pancreatic cancer cell PANC-1, liver cancer cell lines Huh-7 (**Figure 7A-B, and S6B**) or knocking down the expression levels of METTL3 in TMK1 and HCT116 cells (**Figure 7C**); Simultaneously, the expression levels were decreased under MA treatment in GI cancer cells (**Figure 7A-B**).

One possible explanation for the dynamic changes observed among the four genes could be compensatory feedback of m^6^A modification induced by methyltransferase or demethylase. Previous studies showed that the lifetime of mRNAs was elongated under reduced global m^6^A methylation condition 12, 42, to check whether m^6^A regulate the degradation of the components of PI3K/Akt/mTOR signaling pathway, after 48 h of SAH or MA treatment, TMK1 and HCT116 were treated with Act D, an inhibitor of gene transcription. We found that the lifetime of PTEN, AKT1, PIK3CA, and mTOR were significantly increased under SAH and ActD treatment, while they were significantly decreased under MA and ActD treatment (**Figure S6D**), indicating that m^6^A methylation regulates the expression levels of PTEN, AKT1, PIK3CA, and mTOR by inhibiting the degradation of these genes. To investigate the effect of m^6^A alteration on the aforementioned signaling pathway activation, we analyzed m^6^A-related phosphorylation of PI3K/Akt and mTOR signaling effectors using the phospho-MAPK array kit. In TMK1 cell, densitometry analysis of the signal demonstrated that phosphorylation of AKT1, GSK3α/β, p38β, and mTOR was clearly inhibited or activated after treatment with SAH and MA, respectively (**Figure 7C-D**).

## Discussion

To date, several m^6^A modification mechanisms have been explored in cancer; however, the specific functions of the various regulators of m^6^A have yet to be fully identified and characterized in human cancer 22, 24, 25, 51. In this study, we observed dysregulation of m^6^A methyltransferase, demethylase, and special binding protein expression in GI cancer (**Figure 1B-C**), indicating that m^6^A was potentially involved in GI cancer carcinogenesis. Due to the tumor heterogeneity and the limited clinical data, in our study, the METTL3 and ALKBH5 presented conflicting results in overall survival of stomach, liver and pancreatic cancer, further studies need to be performed to investigate the detailed mechanisms. We demonstrated that m^6^A regulators had high frequency copy-number amplification and other genetic alteration in GI cancer (**Figure 1D**). Moreover, we found significant connection between the abnormal expression of several m^6^A regulators and certain GI cancer survival (**Figure 2**). Up to now, there are only a few studies addressing the relationship of m^6^A regulator expression and survival. High expression of YTHDF1 and HNRNPA1 were associated with poor survival in liver cancer 52, 53. High FTO expression was reported to be significantly associated with poor prognosis in gastric cancer 54, METTL3 downregulation acts as an adverse prognostic indicator in liver cancer similar to METTL14 25. These results were all consistent with our bioinformatics analysis result.

The m^6^A levels vary in different tissues and m^6^A regulators widely regulate cellular mRNAs and can contribute to pathway dysregulation, ultimately affecting human physiological processes 15. A previous study demonstrated that m^6^A accelerates mRNA metabolism and translation to influence protein function 11. In this study, protein enrichment in human cancers demonstrated that the alteration in m^6^A regulator expression influenced the expression of multiple proteins in GI cancer (**Figure 3A, Table S1**). Furthermore, we performed a comprehensive analysis of pathways influenced by m^6^A in 27 human cancers (**Figure 3B**). The results highlighted five crucial pathways (PI3K/Akt, mTOR, FoxO, MAPK, and p53) that may be affected by m^6^A and they were also cancer pathways in KEGG (**Figure 3C-D**). Of the enriched five crucial pathways (PI3K-Akt, mTOR, FoxO, MAPK, and p53 pathways), PI3K/Akt/mTOR closely influences the MAPK pathway function by ERK feedback activation 55, 56. Activation or alteration of these pathways are closely involved in cell fate, tumorigenesis, cancer progress, and drug resistance in many cancer types, the therapies targeting these pathways are still investigational 57-63. Of note, the PI3K/Akt and mTOR pathways were found universally altered in different cancers (**Figure 3C**). Our further analysis discovered that the above mentioned five pathways were frequently altered in cancers (**Figure 4A-D**). The crucial molecules PIK3CA, AKT1, PTEN, and mTOR in the PI3K/Akt and mTOR pathways were all frequently dysregulated in different types of cancer (**Figure 4B-C**).

We further carried out structural and functional investigation between m^6^A regulators and the five pathways by protein-protein interaction (PPI), genetic Phylogram tree, co-expression, and m^6^A sites prediction (**Figure 4E, Figure S4A-C**). Mechanistically, m^6^A regulators and PI3K-Akt pathway proteins possessed structural homology (**Figure S4A**). m^6^A was post-transcriptionally installed by “writer” (methyltransferase) within the DRA(m^6^A)CH (D = A, G or U; R = A or G; H = A, C or U) consensus motif and GA(m^6^A)C consensus motif 46, 64. To gain mechanistic understanding of m^6^A function on the five pathways, we analyzed the m^6^A distribution in 57 genes mRNA and found that m^6^A modification sites were mostly enriched in the PI3K-Akt pathway and the mTOR pathway. AKT1/2/3, PI3KCA, PTEN, PI3KR1/2, BCL2, BCL2L1, IRS1, GSK3B, MYC, mTOR, AKT1S1, TSC1, RICTOR, RPTOR, and RPS6KB1 had very high confidence m^6^A modification sites (**Figure 4E**). Consistent with the result, the direct modification of m^6^A on MYC, BCL2 and PTEN has been reported in the literature 65, 66. Taken together, our results demonstrated that the PI3K/Akt/mTOR pathway was most closely related to m^6^A in cancer and m^6^A modification sites were generally distributed in the PI3K/Akt and mTOR pathways. In accordance with our finding, several reports have revealed the influence of m^6^A regulators on the PI3K/Akt/mTOR pathway 51, 67-73, especially for FTO and HNRNPA2B1. However, our study demonstrates for the first time the comprehensive regulation of m^6^A regulators on PI3K/Akt/mTOR pathway members.

To further support of this mechanism, we next applied two independent bioinformatics tools, MeT-DB V2.0 47 and m^6^A SNP 50, to validate the genes m^6^A modulated in the PI3K/Akt/mTOR pathway in tumors. Since the function of m^6^A in eukaryotes involves mRNA degradation 48, we first investigated the effect of METTL3 knockdown in human cancer cells (U2SO, Hela, and A549) and HEK293T cells based on RNA Sequencing analysis (**Figure 5A, Figure S4C**). Results showed that gene expression in the PI3K/Akt/mTOR pathways was generally upregulated after METTL3 knockdown, suggesting METTL3 inhibited their expression. Furthermore, the expression upregulation after METTL3 knockdown was obviously higher in tumor cell lines than in HEK293T. Second, to further confirm the relationship between these genes and m^6^A modification, we employed m^6^A SNP analysis. More than 55,548 human m^6^A sites were referenced for prediction of genetic variants with m^6^A modification sites 35, 36. In the PI3K-Akt pathway, the potential variants that may influence m^6^A modification on AKT1, PTEN, and PIK3CA were mapped on core AC motif (DRACH motif) (**Figure 5C, F, I**). Noteworthy, the C→T transition at reliable m^6^A position in PTEN was in accordance with previous m^6^A variants study 35. The results of detailed m^6^ASNP within AKT1, PTEN, and PIK3CA in the PI3K-Akt pathway are shown in genomic regions (**Figure 5D, G, J**). Such variants may disrupt m^6^A modification and cause disease, including cancer.

With the epigenetic regulation is an important mechanism to m^6^A modification in mRNA 74. It is vital to clarify whether it also plays dual roles in PI3K/AKT/mTOR pathway dysregulation. Our m^6^A sequencing data demonstrated that m^6^A modification are wildly present in TMK1 cells, like AKT1, mTOR, PIK3CA and PTEN m^6^A binding peak enrichment. Moreover, the distribution of m^6^A in these vital pathway genes provides hints as to its functions in expression regulation (**Figure 6C**). Broadly, the appearance of mRNA methylation in translational regulation might be interwoven with other function of m^6^A in RNA biology, such as RNA status and lifetime 48. We further validated modulation of m^6^A on target genes by *in vitro* study using methyltransferase and demethylase selective inhibitors (**Figure 7A-B**). The m^6^A methyltransferase inhibitor SAH 75 elevated the expression of AKT1, PTEN, mTOR, and PIK3CA in stomach and colon cancer cell lines. At the same time, demethylase inhibitor MA decreased their expression 76. In addition, we employed low-scale semiquantitative phosphoproteomic analysis of the PI3K-Akt and mTOR signal pathway critical members to analyze protein phosphorylation in the presence or absence of m^6^A modification. The intracellular kinases activity of AKT1, GSK3B, p38, and mTOR inhibited by the methyltransferase inhibitor SAH and promoted by demethylase inhibitor MA (**Figure 7C-D**). Our current finding demonstrated for the first time that m^6^A modification directly modulate PI3K/Akt and mTOR signal pathway activity by regulating critical kinases in GI cancer.

In summary, we present here a comprehensive study of the major target of m^6^A modification in GI cancer, especially its influence on signal pathways. The findings from the current study reveal the critical influence of m^6^A in GI cancer and contribute to the knowledge of this prevalently existed mRNA regulation mechanism in cancer. This pioneering work provides potential avenues to further investigate the underlying mechanism of m^6^A modification in human cancer.

## Supplementary Material

Supplementary materials and methods, figures, and tables.

## Abbreviations

m^6^A: Methylation at the N6 position of Adenosine
GI: Gastrointestinal
TCGA: The Cancer Genome Atlas
GEO: Gene Expression Omnibus
SNPs: Single Nucleotide Polymorphisms
PI3K: Phosphatidylinositol-3-kinase
mTOR: Mammalian Target of Rapamycin
METTL: Methyltransferase Like
WTAP: Wilms Tumor 1 Associated Protein
YTHDF: YTH N6-methyladenosine RNA Binding Protein
ALKBH5: AlkB Homolog 5
FTO: Fat mass and Obesity-associated protein
MeRIP-Seq: Methylated RNA Immunoprecipitation Sequencing
OS: Overall Survival
FoxO: Forkhead Box Transcription Factors
MAPK: Mitogen-activated Protein Kinase
UTR: Untranslated Region
CDS: Coding Sequence
TSS: Transcription Start Sites
TTS: Transcription Termination Sites
SAH: S-adenosylhomocysteine
MA: Meclofenamic Acid
PPI: Protein-Protein Interaction
FPKM: Per Kilobase of Transcript Per Million Fragments mapped
RPPA: Reverse Phase Protein Array
FDR: False Discovery Rate
NJ: Neighbour-Joining
KNN: K-Nearest Neighbor
VCF: Variant Call Format
qPCR: Quantitative Real-time PCR
KEGG: Kyoto Encyclopedia of Genes and Genomes
