# Supplementary material for: m6A RNA modification modulates PI3K/Akt/mTOR signal pathway in Gastrointestinal Cancer
Source: Theranostics. 2020 Jul 25;10(21):9528–43. doi: 10.7150/thno.42971 (PMC7449908; doi:10.7150/thno.42971)
Supplement: Supplementary file 1 — Supplementary materials and methods, figures, and tables. [file thnov10p9528s1.pdf]

Supplemental Materials and Methods

Primers used to detect endogenous mRNA by qPCR are list as below:

AKT1: Forward: 5'-AGCGACGTGGCTATTGTGAAG-3', Reverse: 5'-GCCATCATTCTTGAGGAGGAAGT-3';

PTEN: Forward 5'-TGGATTCGACTTAGACTTGACCT-3', Reverse 5'-GGTGGGTTATGGTCTTCAAAAGG-3';

mTOR: Forward: 5'-TCCGAGAGATGAGTCAAGAGG-3', Reverse, 5'-CACCTTCCACTCCTATGAGGC-3';

PIK3CA: Forward 5'-CCACGACCATCATCAGGTGAA-3', Reverse, 5'-CCTCACGGAGGCATTCTAAAGT-3'.

GAPDH: Forward, 5'-CTGGGCTACACTGAGCACC-3', Reverse, 5'-AAGTGGTCGTTGAGGGCAATG-3'.

Cell Lines

HCT116 cell was purchased from American Type Culture Collection (Manassas, VA, USA). Gastric cancer TMK1 cell was obtained from Dr. Eiichi Tahara (University of Hiroshima, Hiroshima, Japan). Cells were maintained in complete 1640 medium supplemented with 10% fetal bovine serum (Gibco), 100 IU/mL penicillin and 100 μg/mL streptomycin (Gibco, Invitrogen, CA).

Drug Treatment

The METTL3-METTL14 inhibitor SAH (S-adenosylhomocysteine) was purchased from Sigma (No. A9384). The FTO inhibitor MA (Meclofenamic acid) was purchased from Toronto Research Chemicals (M202750). Cells were treated with a concentration of 1 μM for both drugs at 37°C for 48 h.

Somatic Alteration Data

We applied two platforms to determine the alteration of m^6A-related genes in each tumor sample. First, a copy number collection of functional relevant amplifications and deletions was done using the cBioportal analysis of the GISTIC 2.0 database,

GISTIC was applied separately on each individual cancer type. Second, somatic mutations were mainly obtained from the OncoKB database (http://oncokb.org/) and cBioPortal and calculated with R statistical software.

Supplementary Figures

Figure S1. (A-E), Co-expression analysis of GI cancer related genes with m6A "writer" (METTL3, METTL14, WTAP) and "eraser" (FTO, ALKBH5) by cBioportal. For Pearson and Spearman correlation analysis, score⩾0.3 indicates positive correlation between the genes and score⩽-0.3 indicates negative correlation between the genes (Score >0.3 or <-0.3). Candidate genes were presented with Venny diagrams. (F) multiple condition alteration of TP53BP1 in stomach adenocarcinoma. Missense mutation, truncating mutation, mRNA up-regulation and protein down-regulation were shown in various color type columns. (G) TP53BP1 Copy number units from TCGA stomach statistic. Copy numbers have a similar status in blood (n=236) and stomach (n=94) Copy number in stomach adenocarcinoma (n=173) was obviously lower than which in normal blood and stomach. (H) Kaplan-Meier survival curves represents the overall survival of stomach adenocarcinoma with TP53BP1 expression above. (I) correlation of TP53BP1 expression with TP53BP1 DNA methylation status in stomach adenocarcinoma. The Pearson and Spearman correlation score represent negative expression relationship.

Figure S2. Genes deregulated in FOXO, MAPK and p53 pathways and co-occurrence of PI3K-Akt and mTOR signaling pathway. (A-C) Heatmap of gene alteration frequency in MAPK, FoxO and p53 pathway. Circumambient column displayed the fraction of samples influenced by each type of somatic alteration (mutation, copy-number amplification or deletion) in each pathway gene (Side) and each cancer type (Top). (D) Co-occurrence of genetic alteration in the PI3K-Akt and mTOR signaling pathway by tumor subtypes. e, Simplified pathway network showing the interaction of key PI3K-Akt and mTOR signaling pathway members.

Figure S3. DNA methylation status of five pathway genes in GI cancer. (A-F) Clustered Heatmap showing methylation of m^6A regulators (n=9) and five pathway genes (n=57) in GI cancer. The tumor/normal samples (columns) and genes (rows) were both

clustered.

Figure S4. (A) Phylogenetic tree of five pathway genes and m^{6}A regulators constructed by MEGA7 software. Each branch of the binary tree structure was given a weight based on the distance from node. The PI3K/Akt pathway genes were very closely related to known m^{6}A regulators as indicated by green lines. Red dot size showing the stability of the phylogenetic relationships by bootstrap method. (B) Combined genetic co-expression detected by the STRING database. Correlated gene were displayed with co-expression score. Color intensity reflect the reliability of co-expression. Co-expression of METTL14 and PIK3CA was found (score=0.195). (C) Multi-centric protein-protein interaction (PPI) network of five pathway members and m^{6}A regulators in human by STRING database. Two highly interconnected regions were detected: m^{6}A regulator group and the pathway group. (D) Alteration in expression of FOXO, MAPK and p53 pathway genes in U2SO, Hela, HEK293T and A549 cells upon METTL3 knockdown based on the MeT-DB V2.0 and GEO databases. (E) AKT1S1 transcript reads coverage peaks visualized exon region expression upon METTL3 knockdown in Hela and A549 cells. (F) SNP alteration of m^{6}A motif, the sequence logo was generated automatically based on DRA(m^{6}A)CH structure. (G) Recurrent known SNP mutation relevant to m^{6}A modification in AKT1S1. Recurrent known SNP mutations were color coded in green and annotated with dbSNP label.

Figure S5. (A) Distribution of m6A peak count in the genome. Light blue and light red were represented advance and reverse chain, respectively. (B) m^{6}A peak enrichment in different sub-transcript regions. (C) m^{6}A peak detection number compared to expected. Grey represented the expected detection number in different sub-transcript regions and the colored bar showed the actual detection number. (D) Distribution of m^{6}A peak across the length of TMK1 mRNA. Each region of 5'UTR, CDS, and 3'UTR were binned into 100 segments in the window. (E) TMK1 cell genome m^{6}A signaling was estimated on these gene bodies (between transcription start site, TSS and transcription termination site, TTS). (F) TMK1 cell genome m^{6}A-binding peaks on CpG islands

(CpGI). (G) m^6A/MeRIP-seq analysis of four RNAs (mTOR, AKT1, PIK3CA and PTEN) tested for m^6A peak status in this work. The red lines correspond to all DRACH consensus motifs. The m^6A-antibody-IP data are in blue color and the input sample is in gray color.

Figure S6. Validation of m^6A regulation on PI3K/Akt/mTOR pathway. (A) m^6A levels in GI cancer cells, including esophageal cancer cell TE-1, gastric cancer cell TMK1, colon cancer cell SW480, liver cancer cell lines Huh-7, colorectal cancer cell line HCT116 after treating with 1μM of SAH or MA. (B) Expression mRNA levels of m^6A methylation target genes were measured by qPCR, after the cells were treatment with 1 μM of METTL3-METTL14 inhibitor S-adenosylhomocysteine (SAH) or 1 μM of m^6A demethylase FTO inhibitor meclofenamic acid (MA) for 48 h. (C) Forty-eight hours after siRNA transfection, the mRNA levels of METTL3, AKT1, mTOR, PIK3CA, and PTEN were analyzed by qPCR. (D) mRNA lifetime detection. TMK1 and HCT116 cells were treated with SAH or MA for 48 hours, then cells were treated with Act D (5 μg/mL), an inhibitor of gene transcription, for 0 h, 3 h, and 6 h. The half-life time of AKT1, mTOR, PIK3CA, and PTEN was calculated after checking the mRNA expression levels of these genes. (Student's t-test, *$P<0.05$, **$P<0.01$, ***$P<0.001$, compared with control).

Supplementary Table S1. List of statistically significant enriched protein related to m^6A alteration in different cancer types.

Supplementary Table S2. Pathways significantly influenced by m^6A by DAVID analysis.

Supplementary Table S3. Alteration frequency of PI3K/Akt, FoxO, mTOR, MAPK and p53 signaling pathway genes in different cancer.

Supplementary Table S4. Results for m^6A sit prediction.

Additional file 3: Supplementary materials and methods.

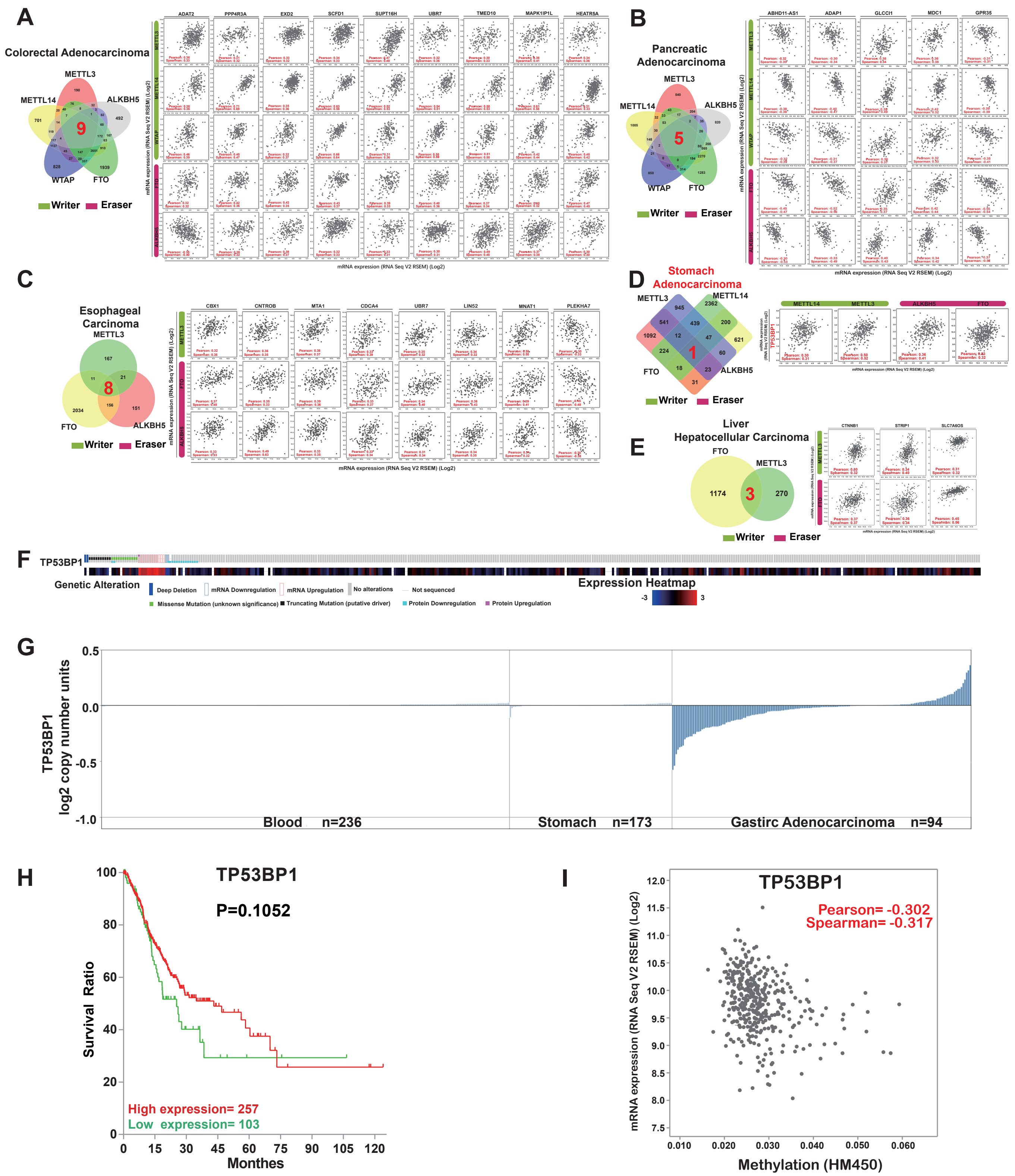
A
Colorectal Adenocarcinoma
METTL3
METTL14
ALKBH5
WTAP
FTO
9
Writer
Eraser
ADAT2
PPP4R3A
EXD2
SCFD1
SUPT16H
UBR7
TMED10
MAPK1IP1L
HEATR5A
mRNA expression (RNA Seq V2 RSEM) (Log2)
B
Pancreatic
Adenocarcinoma
5
ABHD11-AS1
ADAP1
GLCCI1
MDC1
GPR35
C
Esophageal
Carcinoma
8
CBX1
CNTROB
MTA1
CDCA4
UBR7
LIN52
MNAT1
PLEKHA7
D
Stomach
Adenocarcinoma
1
TP53BP1
E
Liver
Hepatocellular Carcinoma
3
CTNNB1
STRIP1
SLC7A6OS
F
TP53BP1
Genetic Alteration
Deep Deletion
mRNA Downregulation
mRNA Upregulation
No alterations
Not sequenced
Missense Mutation (unknown significance)
Truncating Mutation (putative driver)
Protein Downregulation
Protein Upregulation
Expression Heatmap
G
TP53BP1
log2 copy number units
Blood n=236
Stomach n=173
Gastirc Adenocarcinoma n=94
H
TP53BP1
P=0.1052
Survival Ratio
Monthes
High expression= 257
Low expression= 103
I
TP53BP1
Pearson= -0.302
Spearman= -0.317
mRNA expression (RNA Seq V2 RSEM) (Log2)
Methylation (HM450)

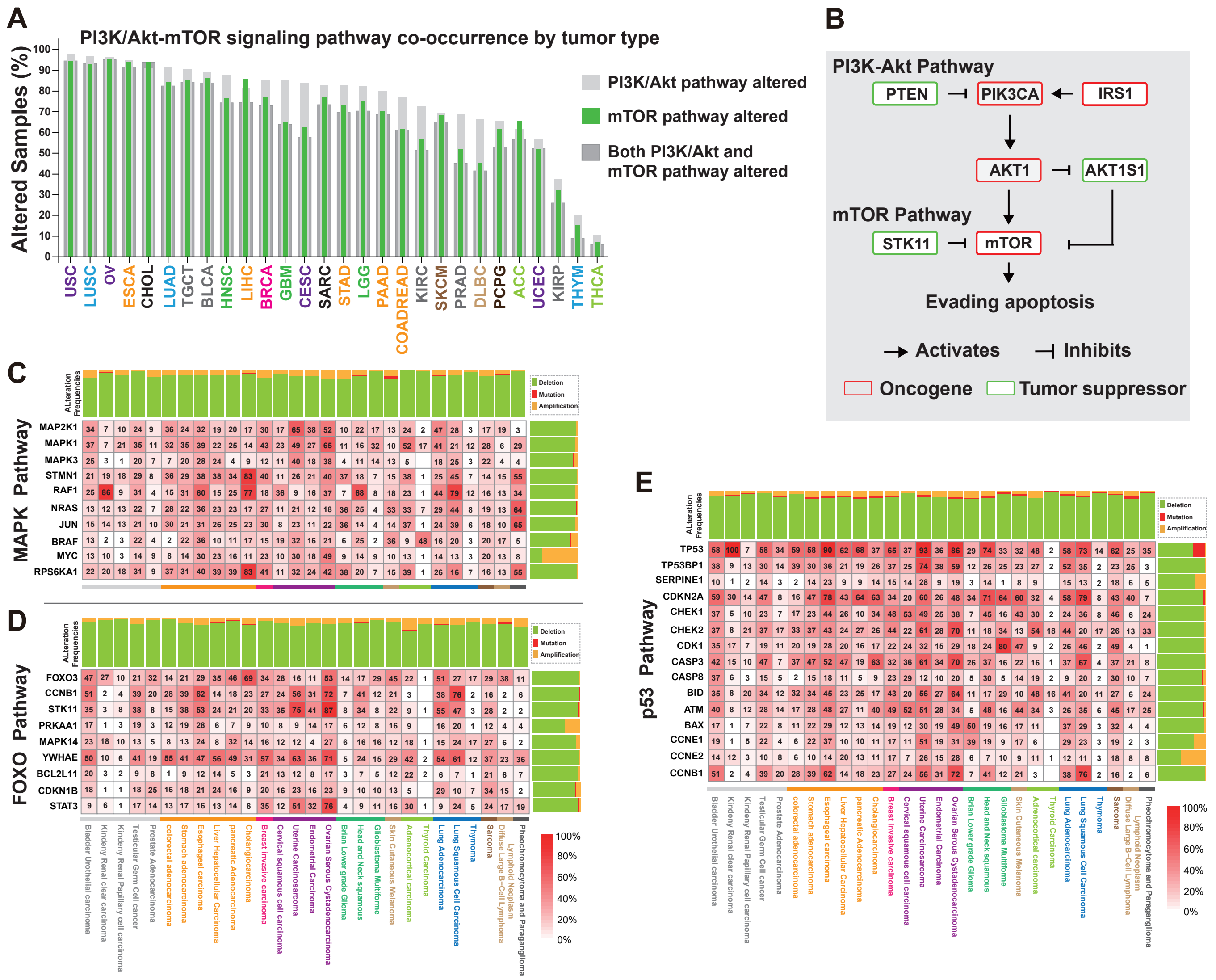

A
PI3K/Akt-mTOR signaling pathway co-occurrence by tumor type
Altered Samples (%)
0
10
20
30
40
50
60
70
80
90
100
PI3K/Akt pathway altered
mTOR pathway altered
Both PI3K/Akt and mTOR pathway altered
USC
LUSC
OV
ESCA
CHOL
LUAD
TGCT
BLCA
HNSC
LIHC
BRCA
GBM
CESC
SARC
STAD
LGG
PAAD
COADREAD
KIRC
SKCM
PRAD
DLBC
PCPG
ACC
UCEC
KIRP
THYM
THCA
B
PI3K-Akt Pathway
PTEN
PIK3CA
IRS1
AKT1
AKT1S1
mTOR Pathway
STK11
mTOR
Evading apoptosis
Activates
Inhibits
Oncogene
Tumor suppressor
C
ALteration Frequencies
Deletion
Mutation
Amplification
MAPK Pathway
MAP2K1
MAPK1
MAPK3
STMN1
RAF1
NRAS
JUN
BRAF
MYC
RPS6KA1
D
ALteration Frequencies
Deletion
Mutation
Amplification
FOXO Pathway
FOXO3
CCNB1
STK11
PRKAA1
MAPK14
YWHAE
BCL2L11
CDKN1B
STAT3
Bladder Urothelial carcinoma
Kindeny Renal clear carcinoma
Kindeny Renal Papillary cell carcinoma
Testicular Germ Cell cancer
Prostate Adenocarcinoma
colorectal adenocarcinoma
Stomach adenocarcinoma
Esophageal carcinoma
Liver Hepatocellular Carcinoma
pancreatic Adenocarcinoma
Cholangiocarcinoma
Breast invasive carcinoma
Cervical squamous cell carcinoma
Uterine Carcinosarcoma
Endometrial Carcinoma
Ovarian Serous Cystadenocarcinoma
Brian Lower grade Glioma
Head and Neck squamous
Glioblastoma Multiforme
Skin Cutaneous Melanoma
Adrenocortical carcinoma
Thyroid Carcinoma
Lung Adenocarcinoma
Lung Squamous Cell Carcinoma
Thymoma
Sarcoma
Diffuse Large B-Cell Lymphoma
Lymphoid Neoplasm
Pheochromocytoma and Paraganglioma
100%
80%
60%
40%
20%
0%
E
ALteration Frequencies
Deletion
Mutation
Amplification
p53 Pathway
TP53
TP53BP1
SERPINE1
CDKN2A
CHEK1
CHEK2
CDK1
CASP3
CASP8
BID
ATM
BAX
CCNE1
CCNE2
CCNB1
100%
80%
60%
40%
20%
0%

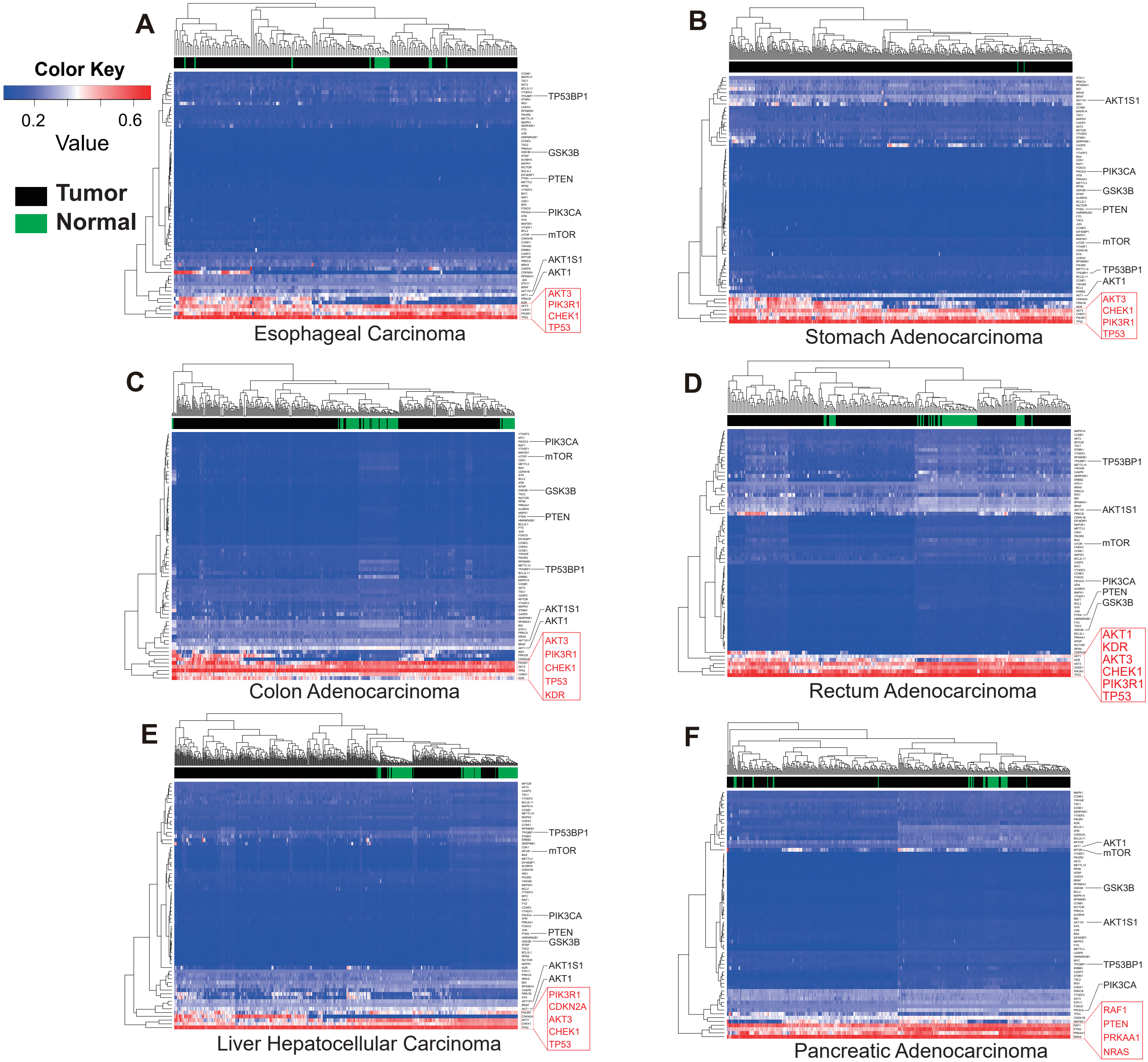

A
Color Key
0.2
0.6
Value
Tumor
Normal
TP53BP1
GSK3B
PTEN
PIK3CA
mTOR
AKT1S1
AKT1
AKT3
PIK3R1
CHEK1
TP53
Esophageal Carcinoma
B
AKT1S1
PIK3CA
GSK3B
PTEN
mTOR
TP53BP1
AKT1
AKT3
CHEK1
PIK3R1
TP53
Stomach Adenocarcinoma
C
PIK3CA
mTOR
GSK3B
PTEN
TP53BP1
AKT1S1
AKT1
AKT3
PIK3R1
CHEK1
TP53
KDR
Colon Adenocarcinoma
D
TP53BP1
AKT1S1
mTOR
PIK3CA
PTEN
GSK3B
AKT1
KDR
AKT3
CHEK1
PIK3R1
TP53
Rectum Adenocarcinoma
E
TP53BP1
mTOR
PIK3CA
PTEN
GSK3B
AKT1S1
AKT1
PIK3R1
CDKN2A
AKT3
CHEK1
TP53
Liver Hepatocellular Carcinoma
F
AKT1
mTOR
GSK3B
AKT1S1
TP53BP1
PIK3CA
RAF1
PTEN
PRKAA1
NRAS
Pancreatic Adenocarcinoma

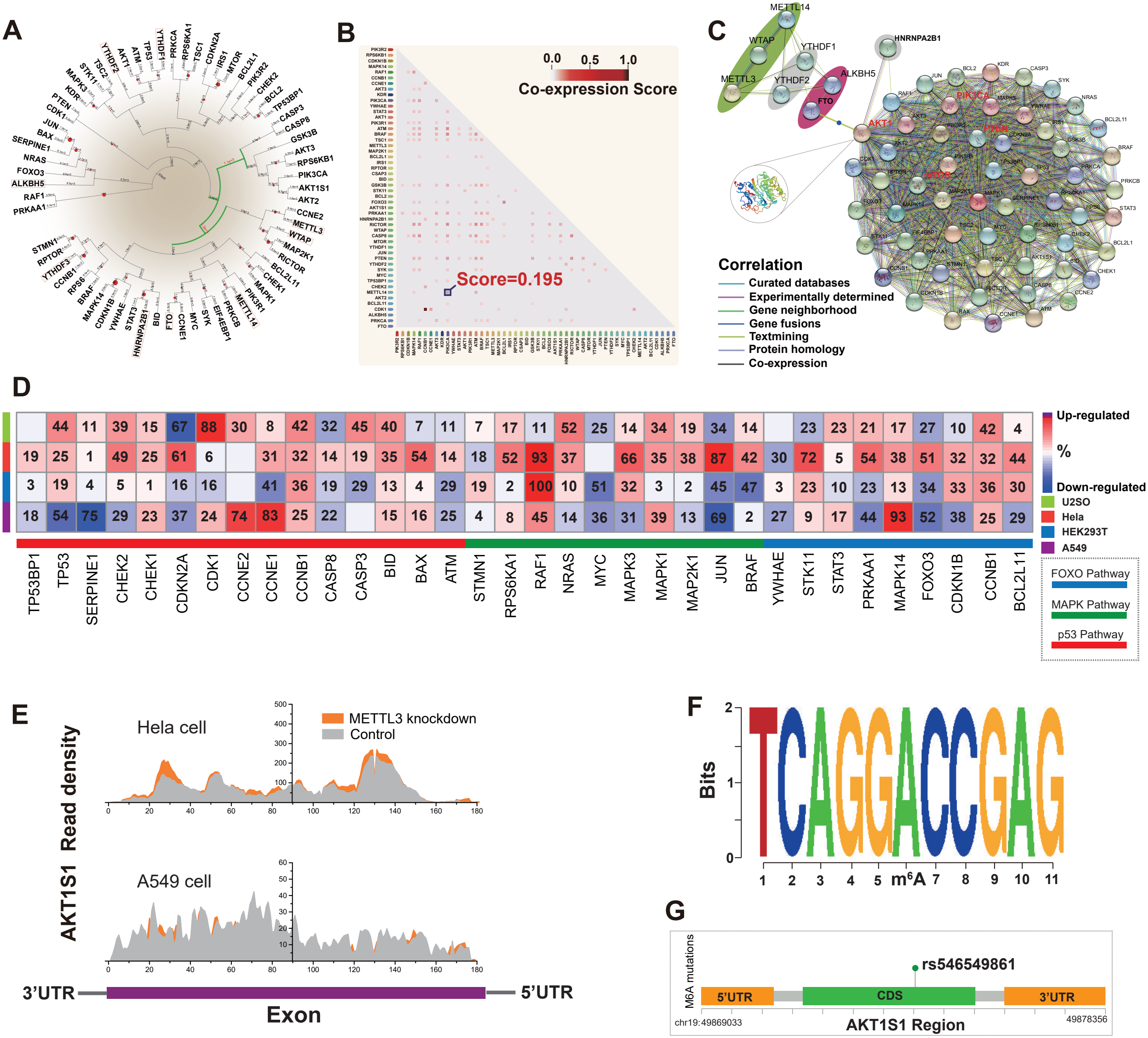
A
B
0.0 0.5 1.0
Co-expression Score
Score=0.195
C
METTL14
WTAP
YTHDF1
METTL3
YTHDF2
ALKBH5
FTO
HNRNPA2B1
AKT1
PIK3CA
PTEN
MTOR
Correlation
Curated databases
Experimentally determined
Gene neighborhood
Gene fusions
Textmining
Protein homology
Co-expression
D
44 11 39 15 67 88 30 8 42 32 45 40 7 11 7 17 11 52 25 14 34 19 34 14 23 23 21 17 27 10 42 4
19 25 1 49 25 61 6 31 32 14 19 35 54 14 18 52 93 37 66 35 38 87 42 30 72 5 54 38 51 32 32 44
3 19 4 5 1 16 16 41 36 19 29 13 4 29 19 2 100 10 51 32 3 2 45 47 3 23 10 23 13 34 33 36 30
18 54 75 29 23 37 24 74 83 25 22 15 16 25 4 8 45 14 36 31 39 13 69 2 27 9 17 44 93 52 38 25 29
TP53BP1 TP53 SERPINE1 CHEK2 CHEK1 CDKN2A CDK1 CCNE2 CCNE1 CCNB1 CASP8 CASP3 BID BAX ATM STMN1 RPS6KA1 RAF1 NRAS MYC MAPK3 MAPK1 MAP2K1 JUN BRAF YWHAE STK11 STAT3 PRKAA1 MAPK14 FOXO3 CDKN1B CCNB1 BCL2L11
Up-regulated
%
Down-regulated
U2SO
Hela
HEK293T
A549
FOXO Pathway
MAPK Pathway
p53 Pathway
E
AKT1S1 Read density
Hela cell
METTL3 knockdown
Control
A549 cell
3'UTR
Exon
5'UTR
F
Bits
1 2 3 4 5 m6A 7 8 9 10 11
G
M6A mutations
rs546549861
5'UTR
CDS
3'UTR
chr19:49869033
AKT1S1 Region
49878356

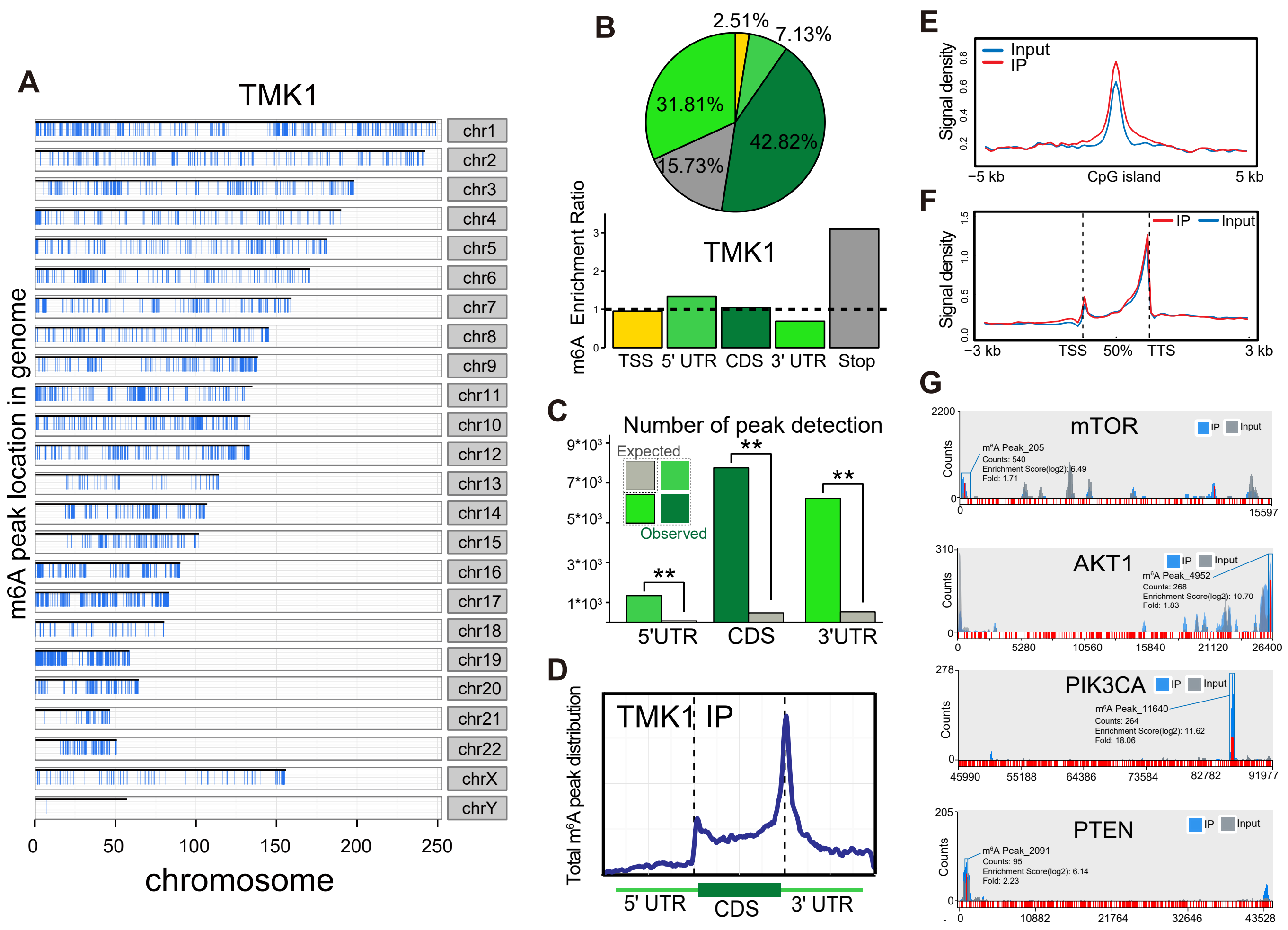

A
TMK1
m6A peak location in genome
chr1
chr2
chr3
chr4
chr5
chr6
chr7
chr8
chr9
chr11
chr10
chr12
chr13
chr14
chr15
chr16
chr17
chr18
chr19
chr20
chr21
chr22
chrX
chrY
0
50
100
150
200
250
chromosome
B
2.51%
7.13%
31.81%
42.82%
15.73%
m6A Enrichment Ratio
TMK1
TSS
5' UTR
CDS
3' UTR
Stop
C
Number of peak detection
Expected
Observed
9*10³
7*10³
5*10³
3*10³
1*10³
**
5'UTR
CDS
3'UTR
D
Total m⁶A peak distribution
TMK1 IP
5' UTR
CDS
3' UTR
E
Signal density
Input
IP
−5 kb
CpG island
5 kb
F
Signal density
IP
Input
−3 kb
TSS
50%
TTS
3 kb
G
mTOR
IP
Input
m⁶A Peak_205
Counts: 540
Enrichment Score(log2): 6.49
Fold: 1.71
Counts
2200
0
15597
AKT1
m⁶A Peak_4952
Counts: 268
Enrichment Score(log2): 10.70
Fold: 1.83
310
5280
10560
15840
21120
26400
PIK3CA
m⁶A Peak_11640
Counts: 264
Enrichment Score(log2): 11.62
Fold: 18.06
278
45990
55188
64386
73584
82782
91977
PTEN
m⁶A Peak_2091
Counts: 95
Enrichment Score(log2): 6.14
Fold: 2.23
205
10882
21764
32646
43528

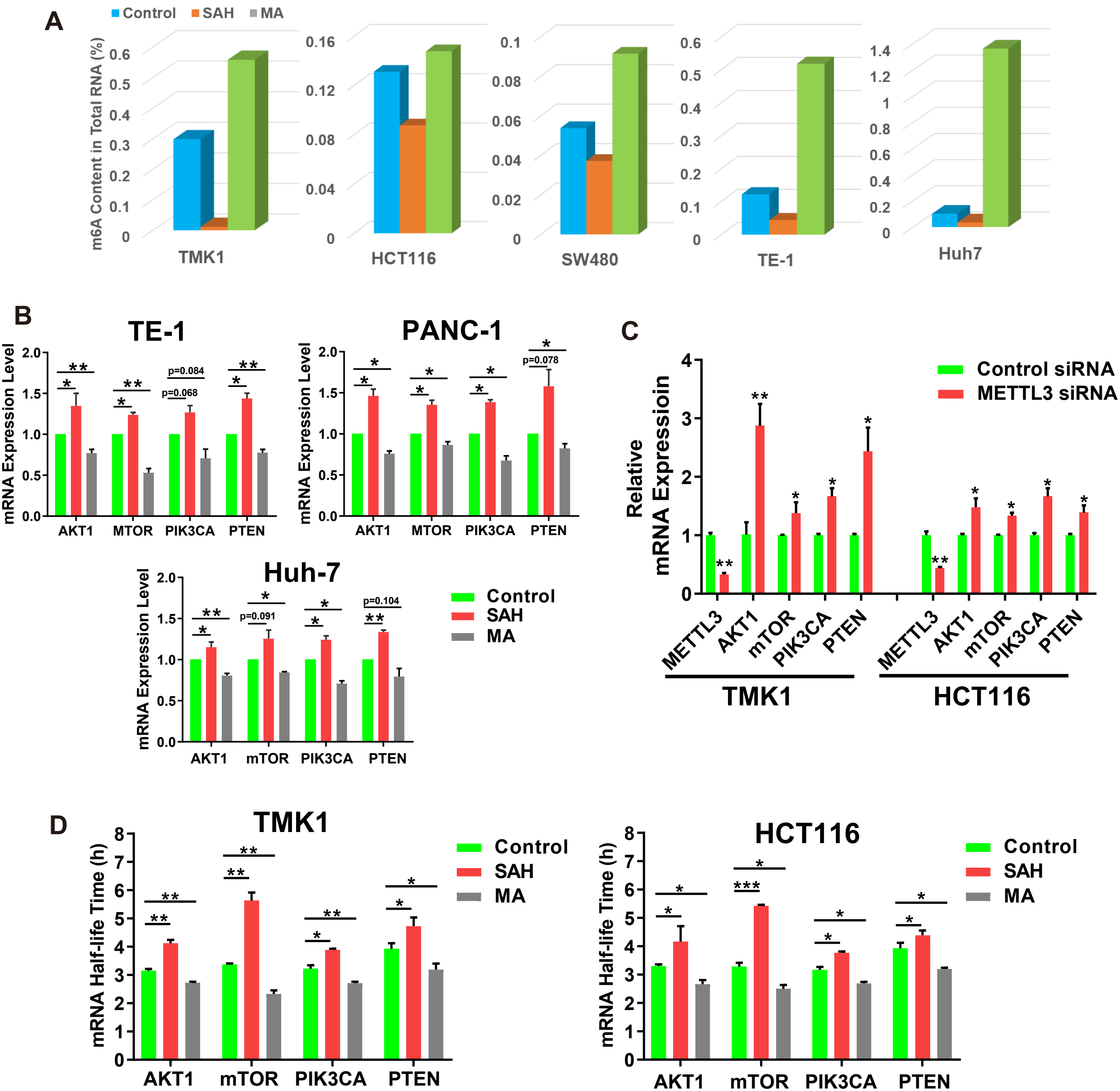

A
Control
SAH
MA
m6A Content in Total RNA (%)
TMK1
HCT116
SW480
TE-1
Huh7
B
TE-1
PANC-1
Huh-7
mRNA Expression Level
AKT1
MTOR
PIK3CA
PTEN
mTOR
p=0.084
p=0.068
p=0.078
p=0.091
p=0.104
Control
SAH
MA
C
Relative
mRNA Expressioin
Control siRNA
METTL3 siRNA
METTL3
AKT1
mTOR
PIK3CA
PTEN
TMK1
HCT116
D
TMK1
HCT116
mRNA Half-life Time (h)
AKT1
mTOR
PIK3CA
PTEN
Control
SAH
MA

Cancer	Writer	Reader	Eraser
Adrenocortical Carcinoma	NOTCH1, EIF4E, ERBB2, STAT5A, GATA3, ETS1, RPTOR	PDK1, YWHAE, SQSTM1, MAPK1, MAPK3, RPS6KA1	PDK1, YBX1, PCNA, BIRC2, ARAF, HSPA1A, MTOR, CCND1, NF2, MAPK14, GAB2, RPS6KA1
Bladder Urothelial Carcinoma	PCNA, CHEK1, RBM15, CCNB1, EEF2, BAX, SRSF1, TFRC, CCNE2, RICTOR, PRKCA, MYH11, GATA6, ESR1, MAPK9, MAP2K1, ACVRL1, HSPA1A, PGR, TGM2	CHEK2, DIABLO, CHEK2, BAK1, CHEK1, SRC, RB1, CCNE2, AKT1S1, ESR1, BCL2L1, CDKN1B, ASNS, EEF2, SRSF1, IGFBP2, FOXM1, EGFR, RAF1, BCL2L11, KDR, ANXA1, PRKCA, TSC1, PRKCB, MAPK14, NRAS, PDK1, PIK3R1, PIK3R2, EIF4E, PEA15, PARK7, MAPK1	IGFBP2, RB1, FOXM1, CDH1, BAP1, RBM15, AKT1S1, XRCC1, RPS6KB1, CHEK1, GAB2, MSH2, KDR, TGM2, G6PD, LCK, CASP7, PIK3R1, PIK3R2, MAPK1, PEA15, ANXA1, MAPK14, YAP1, BAX, PARK7
Breast invasive Carcinoma	NKX2-1, BAP1, RBM15, CDK1, EIF4EBP1, CASP9, RPS6KB1, MSH6, RPS6, CDH1, ADAR, CCNE1, SLC1A5, ENY2, JAK2, CASP3, EIF4G1, MYH9, KAT2A, FOXM1, ASNS, CCNB1, ERCC1, EEF2, MSH2, CHEK2, YWHAZ, CHEK1, DVL3, CCNE2, BRD4, CTNNB1, ERRFI1, PARP1, MET, MTOR, TP53, SCD, SQSTM1, RB1, BRAF, FASN, BRCA2, GAPDH, GATA6, ACACA, COG3, EGFR, CASP8, PGR, BAK1, CAV1, PIK3R1, PIK3R2, BCL2A1, ETS1, MS4A1, STK11, RAB11A, RAB11B, YAP1, PEA15, MAPK9, YWHAB, MYH11, TIGAR, BID, PRKCA, PXN, MAP2K1, MAPK14, ANXA1, RAD51, CDH2, MAPK1, MAPK3, IRS1, ERBB3, COL6A1, ACVRL1, ESR1, PARK7, ANXA7, STAT3, RICTOR, PDK1, STAT5A, BCL2, BCL2L1, AR, AXL, PRKAA1	CCNB1, FOXM1, YWHAZ, RPS6, CCNE2, ASNS, SRC, MSH2, SQSTM1, ADAR, RPS6KB1, ERBB2, EIF4EBP1, CCNE1, MSH6, CASP3, CDH1, CHEK1, EGFR, ACACA, PCNA, NDRG1, SCD, BAP1, BRCA2, G6PD, CASP7, CDK1, EIF4G1, RBM15, BRAF, SRSF1, MYH9, FASN, TGM2, PRDX1, EIF4E, DIABLO, ACACB, TSC2, PARP1, TP53, CCND1, ARAF, RPS6KA1, PARK7, PRKAA1, YAP1, PIK3R1, PIK3R2, MAPK1, PGR, MAPK9, CAV1, PDCD4, MAPK8, STK11, ATM, TP53BP1, STAT5A, STAT3, MAPK14, MYH11, BCL2, AKT1, AKT2, AKT3, AR, PEA15, COL6A1, PRKCB, ANXA7, DPP4, PRKCA, CASP8, INPP4B, RAB11A, RAB11B, COG3, ESR1, CDKN1B, IRF1, EEF2K, RAD50, BCL2A1, YWHAE, MAP2K1, BAD, MAPK3	NKX2-1, MTOR, RBM15, SQSTM1, MYH9, BAP1, RPS6, DVL3, CASP9, ADAR, AKT1S1, GSK3A, GSK3B, MSH2, ACACA, PTEN, EIF4G1, DIABLO, CDH1, CDK1, RPS6KB1, ERCC5, MSH6, TSC2, JAK2, CTNNB1, CHEK2, BRAF, KAT2A, CTNNA1, EEF2, CCNE1, ACACB, ABL1, RB1, EPPK1, TIGAR, STK11, MS4A1, BCL2A1, RAD51, YWHAB, BAK1, BID, CDH2, YAP1, LCK, MYH11, CAV1, RAB11A, RAB11B, ETS1, MRE11, STMN1, CASP8, MAP2K1, HSPA1A, ANXA1, WWTR1, RAF1, ACVRL1, RICTOR, EGFR, BCL2L1, CLDN7, PEA15, NRAS, MYC, CDKN1B, EIF4EBP1
Brian Lower grade Glioma	MTOR, PARK7, CCNE1, PRKCA, SMAD4, KDR, CTNNB1, PIK3CA, ERBB2, SERPINE1, EGFR, WWTR1, RAB11A, RAB11B, FN1, YBX1, XBP1, ANXA1, MYC, CDH2, HSPA1A, COL6A1, RPS6	[illegible] JUN, ERBB2, XRCC1, EGFR, RBM15, EIF4EBP1, BAX, TFRC, CDKN1B, EIF4G1, IGFBP2, CAV1, TP53BP1, RAD50, BCL2L1, XRCC5, RAF1, RPS6, BID, PRDX1, MAPK1, PEA15, PARK7, RPS6KB1, ANXA7, MAP2K1, PRKCA, PGR, PRRT, PRKCD, MTOR, PTEN, SRC, RPTOR, MAPK3, ESR1, PRKAA1	CHEK1, PXN, MAPK14, PARP1, MSH6, MSH2, YWHAB, SYK, YBX1, MAPK8, CCND1, YWHAZ, PDK1, RPS6KA1, AKT1, AKT2, AKT3
Cervical squamous cell Carcinoma	MSH2, MSH6, CHEK1, MTOR, EEF2K, BAP1, CDH1, SRSF1, SMAD3, CCND1, RICTOR, ETS1, EGFR, BID, LCK, YWHAB, COL6A1, CDH2, IRS1, RAD51, STK11, RAF1	SMAD1, GSK3A, GSK3B, BRAF, BAP1, RAD50, MAPK9, NRG1, LCK, BCL2L1, SMAD3, RAB11A, RAB11B, CCND1, YWHAZ, CDKN1B, HSPA1A, RAD51	RPTOR, RPS6KB1, FN1, G6PD
Cholangio carcinoma	AKT1, AKT2, AKT3, SRC, EGFR, MTOR, MAP2K1, SMAD3, RPTOR, KIT, CDH3, CDK1, TFRC, PARP1, MAPK14, GAB2, CCNB1, ERRFI1, RB1	CDKN1B, EGFR	PCNA, FASN, ATM, BCL2, SQSTM1

**Colorectal Adenocarcinoma**	NKX2-1, CLDN7, GSK3A, GSK3B, AKT1, AKT2, AKT3, SLC1A5, MAPK1, MAPK3, MAP2K1, MAPK14, EEF2, ERBB2, CAV1, NF2, BCL2L1, DPP4, PGR, STMN1, IRS1, PIK3CA, RICTOR, NRG1, PRDX1, ESR1, RB1, RPS6KA1, ERRFI1, BAK1, MSH2, FOXM1, CHEK1	MSH2, SRC, PIK3CA, TP53, SRSF1, RAD50, NRAS, YAP1, GSK3A, GSK3B, XRCC5, BCL2L11, RB1, BAP1, RICTOR, ESR1, PRKCA, RPTOR, PEA15, PDK1, STAT3, ASNS, FN1, CHEK2, ARID1A, COPS5, TIGAR, SCD, YWHAE, BAK1, PRDX1, ARAF, DPP4, XBP1, TSC1, CDKN1B, ATM, RAB25, MAPK1, MAPK3, MAP2K1, PTPN11, IGFBP2, MAPK14, CASP7, ERBB2, TSC2, GAPDH, PDCD4, CHEK1, AKT1, AKT2, AKT3, RAB11A, RAB11B, EGFR, CLDN7, EEF2, YBX1, PRKCB, TUBA1B, MYH9943, ETS1, EGFR068, PTEN, NDRG1, EIF4EBP1,	NKX2-1, PTPN11, CAV1, GSK3A, GSK3B, MYH9, ERBB2, MTOR, KDR, GSK3A9, YBX1, EGFR, NFKB1, AKT1S1, KIT, FOXO3, PRKCA, MAPK1, MAPK3, INPP4B, PRKAA1, AXL, PEA15, ETS1, MAPK14, RAB11A, RAB11B, PRKCB, SRC, TP53, SETD2, STMN1, YAP1, YWHAE, CHEK1, CCNE2, CDH3, ARID1A, BCL2A1, ERRFI1, MSH2, PIK3CA, BAK1, PIK3R1, PIK3R2, BAX, SRSF1, ARAF, SMAD4, RB1, NRAS, TIGAR, PARP1, BRCA2, MET, BECN1, ESR1
**Endometrial Carcinoma**	MAP2K1, ARID1A, EIF4E, PRDX1, COL6A1, ARAF, PCNA, SMAD3, CHEK1, ERBB3	TP53, PIK3CA, CCNE1, FOXM1, BAP1, MRE11, YWHAB, PREX1, SHC1, CDH3, PECAM1, DIRAS3, PTEN, CHEK2, CCNB1, JUN, TIGAR, BAX, AKT1, AKT2, AKT3, EEF2, GSK3A, GSK3B, PRKAA1, ANXA1, ESR1, PARK7, EGFR, CTNNB1, MAPK1, ETS1, RPS6KB1	DVL3, RBM15, MTOR, RPTOR, CCNE2, TSC2, CCNB1, ASNS, TFRC, CASP8, ESR1, SNAI1, MAPK8, COL6A1, EGFR, ITGA2, TUBA1B, STK11, ACVRL1
**Esophageal Carcinoma**	SHC1, PRKCA, ANXA7, RBM15, MSH6, TP53BP1, PTEN, XRCC5, RPS6, CHEK1, AKT1S1, SRSF1, FASN, ANXA1, RAF1, CAV1, KDR, AKT1, AKT2, AKT3, SYK, PRKAA1, YBX1, EEF2, SRC, GATA3, XRCC1, SMAD4, ERBB2, RAB11A, RAB11B, XBP1, MS4A1, ARAF, RICTOR, SMAD1, ITGA2, ACACA, ACACB	ANXA1, RAF1, CAV1, KDR, AKT1, AKT2, AKT3, SYK, PRKAA1, YBX1, EEF2, SRC, GATA3, XRCC1, SMAD4	ERBB2, RAB11A, RAB11B, AKT1, AKT2, AKT3, XBP1, AKT1S1, MS4A1, ARAF, RICTOR, SMAD1, ITGA2, ACACA, ACACB
**Glioblastoma Multiforme**	CDKN1B, MYH9, BRD4, RICTOR, DVL3, ARID1A, CAV1, EIF4EBP1, PECAM1, TFRC, AKT1S1, MAPK8, CDKN1B, RPS6	DVL3, XRCC5, BAP1, RBM15, LCK, MRE11, BCL2L1, BAK1, DIRAS3, NRG1, SYK	TUBA1B, NDRG1, RPS6KB1, EGFR, FOXM1, ERBB2, SRSF1, SHC1, ESR1, CDKN1A, YBX1, CHEK1, MYH9, XBP1, PCNA, ETS1
**Head and Neck squamous cell**	PIK3R1, PIK3R2, CHEK2, CCNB1, MYC, EZH2, TP53BP1, TYMS, ANXA1, MAPK14, EIF4EBP1, EIF4E, ERBB2, YAP1	BAK1, ERRFI1, RET, MAPK8, IRS1, EGFR7, ATM, BCL2, SYK	ESR1, ERBB3, PRKCA, YBX1, RPS6KB1
**Kindeny Renal clear Carcinoma**	FOXM1, CLDN7, AKT1S1, XRCC1, SDHB, CCND1, ERRFI1, GAB2, ADAR, ANXA1, ETS1, YAP1, SYK, BID, RAD50, NKX2-1, STK11, RB1, SMAD3	TFRC, DIABLO, ACACA, ACACB, PCNA, SERPINE1, CHEK1, CCNE1, BCL2L1, CCNB1, ASNS, FASN, EEF2, SRC, TIGAR, IGFBP2, FOXM1, CDKN1A, XRCC1, MET, SRSF1, CDKN1B, BAX, TP53, ARAF, RAB25, CHEK2, PRKCD, SQSTM1, YWHAZ, EIF4E, ITGA2, AKT1S1, PTEN, GAB2, ERRFI1, CTNNB1, MYH11, TSC1, RAB11A, RAB11B, AKT1, AKT2, AKT3, SHC1, RAD50, NOTCH1, NFKB1, EGFR, VHL, TUBA1B, MAPK1, MAPK3, MYH9, AR	BAP1, NOTCH1, ADAR, ERRFI1, FOXO3, PEA15, PTEN, HSPA1A, RPTOR, ETS1, EGFR, ANXA1

**Kindeny Renal papillary Carcinoma**	RB1, CCND1, TP53BP1	[illegible] TSC1, GSK3A, GSK3B, RPS6KB1, MTOR, BRAF, RPTOR, ERBB2, BCL2L1, EEF2K, BAX, EIF4EBP1, EEF2, RBM15, RPS6, ATM, CCNE1, MSH6, PRKAA1, TFRC, SMAD3, SRC, EGFR, TP53BP1, RAD50, NRAS, PECAM1, TP53, ESR1, RPS6KA1, PRDX1, CDK1, BRCA2, SCD, CHEK2, SMAD4, FOXO3, BAK1, FOXM1, CDH3, MYC, PGR, RAB11A, RAB11B, INPP4B, RAD51, MAPK8, PREX1, CDKN1B, MRE11, MAPK3, ACVRL1, SRSF1, CHEK1, NDRG1, NOTCH1, STMN1, EIF4E, RAF1	RAF1, MAP2K1, PXN, GAB2, ERBB2, ARAF, EIF4EBP1, TSC2, MYC, BCL2, FOXO3, PREX1, PECAM1, RPS6KB19, XRCC1, YAP1
**Lung Adenocarcinoma**	EIF4E, GATA3, MAP2K1, MAPK14, PTEN, SQSTM1, YBX1	[illegible] FOXM1, CCNB1, CCNE2, CHEK2, EIF4EBP1, CDKN1B, MSH2, RBM15, DVL3, XRCC1, BAP1, BAK1, RPS6, CCNE1, TFRC, CHEK1, STMN1, SMAD1, CAV1, MAPK1, MAPK3, MTOR, PRKCA, STAT3, SRC, MYH11, MAPK14, ANXA1, COL6A1, YAP1, RPS6KA1, KDR, PXN, LCK, PIK3R1, PIK3R2, YBX1, PTEN, TGM2, RICTOR	CASP7, ERCC1, CHEK2
**Lung Squamous Cell Carcinoma**	TYMS, CHEK2, CASP9, SMAD3, PCNA, EGFR, RAF1, AKT1, AKT2, AKT3, AKT1S1, STK11	BAX, PARK7, FASN, EIF4EBP1, ASNS, ACACA, YWHAZ, PCNA, GSK3A, GSK3B, ESR1, DIABLO, CCNB1, YAP1, ACACB, CCNE1, SRC, CASP7, PDK1, MAPK1, MAPK3, RPS6KA1, FOXO3, JUN	IGFBP2, DIABLO, EIF4EBP1, NDRG1, MAPK14, DVL3, AKT1, AKT2, AKT3, AKT1S1, PIK3CA, XRCC1, GAPDH, GSK3A, GSK3B, ARID1A, RPS6KB1, PDK1, ESR1, GATA3, IRS1, RAB11A, RAB11B, LCK, ETS1, NAPSA, PECAM1, INPP4B, XBP1, ERRFI1, MACC1
**Lymphoid Neoplasm Diffuse Large B-Cell Lymphoma**	ATM, PRKCD, ERBB3, MS4A1, IRS1, YBX1, BAX, CHEK2, MSH6, ATM, BCL2L11, MET, RAB11A, RAB11B, WWTR1, TFRC, ERBB2, NRAS, STK11, SHC1, BAD, MTOR, AKT1, AKT2, AKT3, RPS6KB1	ERCC1, RAF1, XRCC5, EIF4EBP1, MSH6, EIF4G1, RPS6KB1, RBM15, EEF2K, STAT5A, BRAF, NFKB1, BIRC2, TSC2, ESR1, CTNNB1, COPS5, XBP1, EGFR, FOXO3, DIRAS3, MAPK1, MAPK3, BECN1, ANXA1, RAB25, SCD, MAPK8, ERBB3, CAV1	SHC1, BAD, MTOR, AKT1, AKT2, AKT3, RPS6KB1, BCL2L11, MET, RAB11A, RAB11B, WWTR1, TFRC, ERBB2, NRAS, STK11
**Ovarian Serous Cystadenocarcinoma**	FASN, NF2, GAB2, RPTOR, RPS6KB1, DVL3, ARID1A, MET, CASP8, KIT, ERCC1, STMN1, RICTOR, MTOR	EIF4G1, RB1, JUN, EIF4E, NFKB1, XRCC1, TSC2, PGR, AKT1S1, SRC, RPS6KA1, BAP1, RPS6KB1, DVL3, GSK3A, GSK3B, IGFBP2, CDKN1B, NF2, ETS1, PRKCA, ERBB3, INPP4B, XBP1, ACACA, PRKAA1, CASP8, PIK3R1	MSH6, XRCC1, EIF4G1, SQSTM1, CDK1, AKT1, AKT2, AKT3, GAB2, EGFR, CCNB1, ESR1, TIGAR, RB1, KDR, BRCA2, CASP8, MET, PDK1, MAPK8, RICTOR
**Pancreatic Adenocarcinoma**	PRKAA1, CHEK2, SRC, MYC, SERPINE1	RBM15, BIRC2, PDK1, GAPDH, CCNB1, BRAF, CCNE1, ERBB2, CDH3, CLDN7, PRKAA1, ERBB3, TFRC, RPS6KB1, RPS6KA1, FOXO3, MYH11, RICTOR, RAD50, ESR1	INPP4B, KDR, BIRC2, PRKAA1, GATA3, RBM15, XRCC5, ADAR, NRG1, COL6A1, RICTOR, ACVRL1, SMAD4, STMN1, BAK1, TGM2, NRAS

**Pheochromocytoma and Paraganglioma**	ATM, MAPK14, BIRC2, ACACA, LCK, SERPINE1, SQSTM1, BAX, SMAD4, ACACB, PIK3CA, XRCC1, RICTOR, YWHAZ, MS4A1, TTF1, PARK7, PRDX1, CDKN1B, EGFR, BCL2L11, AR, PGR, ATM, KIT, EEF2, XRCC5, EEF2K, EIF4EBP1, DVL3, ERBB3, ASNS, CCNE2, GAB2, KDR, CCND1, NRAS, MAP2K1	STAT5A, ANXA7, GAB2, MAPK8, FASN, GATA3, RAF1, MAPK14, ERCC1, KDR, ERBB3, SERPINE1, YBX1, PARK7, YAP1, PRDX1, EIF4E, EEF2, CDH3, NOTCH1, PREX1, NDRG1, MYH9, ACACA, SQSTM1, CAV1, PGR, EIF4EBP1, RBM15, RAD50, CDKN1B, FOXO3	ERBB3, ASNS, CCNE2, GAB2, KDR, CCND1, NRAS, MAP2K1, KIT, EEF2, XRCC5, EEF2K, EIF4EBP1, DVL3
**Prostate Adenocarcinoma**	PDK1, KDR, FN1	RPS6, ACACA, CHEK2, MYH9, XRCC1, BCL2L11, MTOR, FASN, SQSTM1, SYK, BAP1, AR, RAF1, SRSF1, CCNB1, BRAF, EIF4G1, ACACB, ATM, RBM15, TSC2, RPS6KB1, ESR1, CDH1, CCNE2, XRCC5, PCNA, ERBB3, PDK1, PREX1, TFRC, PRKAA1, IGFBP2, BIRC2, CTNNB1, VHL, TP53BP1, GSK3A, GSK3B, RB1, ASNS, EIF4EBP1, PIK3CA, BAK1, CHEK1, MS4A1, EGFR, CDH2, ACVRL1, RICTOR, BID, PRKCA, STMN1, YAP1, COL6A1, PRRT2, PRKCD, MRE11, PGR, CDKN1A, HSPA1A, MYC, STK11, MET, CCND1, BRCA2, KIT, RAB11A, RAB11B, PEA15, MAPK1, CAV1, NRG1, MAP2K1, MAPK8, YBX1, YWHAB, TTF1, TGM2, MAPK14, BCL2L1	GAB2, ACACB, TP53BP1, TSC2, ACACA, RPS6KB1, XRCC1, ERCC1, RBM15, ERRFI1, CHEK2, BCL2L11, PDK, XRCC5, BAP1, EIF4G1, DVL3, TFRC, BAK1, ESR1, BRAF, EIF4EBP1, CCNE2, RPS6, ACVRL1, PGR, MAP2K1, MAPK8, PRKCA, YAP1, NF2, MTOR, RICTOR, YWHAB, SHC1, CAV1, ITGA2, HSPA1A, PEA15, MAPK1, MAPK3, PRKCD, PRRT2, STAT3, PDCD4
**Sarcoma**	NF2, CHEK2, ACACA, ACACB, ESR1, MTOR, XRCC5, BRAF, ERCC1, SQSTM1, BAK1, RB1, TFRC, CCNE1, COL6A1, ACVRL1, PGR, RAB25, NRG1, RAD50, CCND1	ERCC1, BAP1, CCNB1, PRKAA1, FOXM1, MTOR, TFRC, RBM15, RPS6, RAF1, NF2, CCNE1, RPS6KB1, TSC2, BCL2L11, EIF4G1, MSH6, TP53BP1, ACACA, ACACB, XRCC1, SMAD3, ESR1, ASNS, NDRG1, PXN, RB1, EGFR, YBX1, PEA15, ACVRL1, FOXO3, YAP1, NOTCH1, MAP2K1, PECAM1, MYH11, CDH1, MAPK14, CTNNB1, COL6A1, PGR, RAB25, EIF4E, MAPK1, MAPK3, ITGA2, RAD50, BRCA2, INPP4B, PARK7, BAX, GAPDH, NRAS, RICTOR	CCNB1, GSK3A, GSK3B, CAV1, CCNE1, PCNA, AKT1S1, EIF4EBP1, NDRG1, CDK1, BAD, AKT1, AKT2, AKT3, RICTOR, VHL, CHEK1, SRSF1, CDH1, PRKAA1, TFRC, DVL3, MYH11, CTNNB1, ARAF, FN1, ASNS, MTOR, RAF1, RAB11A, RAB11B, ANXA7, ERBB2, EGFR, RPS6KB1, LCK, BIRC2, PREX1, BCL2, RAD51, ERBB3, PDCD4, TIGAR, MAPK14, BAX, ANXA1, BCL2L1, RPS6KA1
**Skin Cutaneous Melanoma**	MAPK14, MET, TFRC, CTNNA1, ERRFI1, PIK3R1, PIK3R2, PRKAA1, PCNA, ADAR, CDK1, G6PD, SQSTM1, EIF4EBP1, PDK1, TIGAR, FOXO3, IGFBP2, PRKCA, MAP2K1, INPP4B, SRC, CASP7, NOTCH1, BRCA2, FASN, MYH11, ITGA2, AXL, RBM15, PREX1, WWTR1,	CDK1, CCNB1, YWHAZ, EEF2K, PCNA, FOXM1, MYC, CCNE1, CCNE2, YBX1, KIT, PRKCA, MYH9, DIRAS3, PRKAA1, ANXA7, ESR1, G6PD, ITGA2, ANXA1, RAB25, EGFR, MAPK9, PIK3CA, INPP4B	ADAR, CCNB1, CCNE1, COPS5, DIABLO, KIT, MSH2, SQSTM1, STAT3, AKT1, AKT2, AKT3, IGFBP2, SMAD1, MYH11

**Stomach Adenocarcinoma**	MYH9, TFRC, ACACB, BCL2L1, ACACA, CCNB1, CCNE1, GSK3A, GSK3B, ERBB2, RB1, SRSF1, RAD50, NF2, ESR1, BCL2, PREX1, IGFBP2, RPS6KB1, DIRAS3, CHEK16	PCNA, TFRC, FOXM1, RPS6, CCNE1, EIF4EBP1, CLDN7, SRC, ASNS, BCL2L11, CDK1, SCD, MYH9, BAP1, ESR1, CCNB1, RBM15, XRCC1, BCL2L1, SRSF1, MSH6, FASN, CCNE2, CASP3, CDKN2A, DIABLO, SMAD1, BRD4, MSH2, ARID1A, YWHAZ, ACVRL1, BCL2, KIT, RICTOR, PRKCA, PEA15, CAV1, BRCA2, COL6A1, KDR, ABL1, FOXO3, AKT1, AKT2, AKT3, YAP1, RAB11A, RAB11B, PGR, MAPK1, MAPK3, PIK3R1, PIK3R2, STMN1, PREX1, HSPA1A, PRKCB, PRKCD, MTOR	PARK7, DPP4, RAD50, PRKAA1, PRKCD, ERCC5, STK11, DIABLO, PARP1, YBX1, MAPK1, MAPK3, SRC, NOTCH1, FOXM1, RPS6, JUN, PTPN11, BID
**Testicular Germ Cell Cancer**	GSK3A, GSK3B, PREX1, PCNA, ASNS, ITGA2, TGM2, CAV1, ERRFI1, EIF4G1, SMAD4	NF2, CHEK2, EEF2, MSH2, MSH6, ADAR, SMAD3, XRCC5, MTOR, ESR1, CCNB1, MS4A1, PARP1, YWHAZ, RPS6, EEF2K, PRDX1, ACACA, GAPDH, BCL2L11, PDCD4, CCNE2, SCD, CHEK1, RBM15, ACACB, KIT, BAX, ASNS, BRAF, RPTOR, BAP1, EIF4EBP1, CCNE1, GSK3A, GSK3B, RAF1, FOXM1, XRCC1, SRSF1, CASP7, PCNA, EIF4G1, CDKN1B, PRKCD, AKT1S1, SHC1, PXN, EIF4E, RB1, TIGAR, PREX1, SMAD1, CDH3, CLDN7, RPS6KB1, PARK7, AKT1, AKT2, AKT3, PRKCA, MAPK14, MAPK8, YAP1, EGFR, ANXA1, PRKAA1, STK11, RPS6KA1, HSPA1A, FOXO3, CDH2, DIRAS3, BID, MAP2K1, YBX1, FN1, WWTR1, MRE11, CAV1, SRC, BCL2, ACVRL1, PECAM1, NOTCH1, IRS1, ERBB2, CCND1, TSC1, ETS1, IGFBP2, MAPK9, RAD51, MAPK1, MAPK3, PDK1, TP53, TGM2, NFKB1, GATA3, AKT, PGR, PRRT2, PTEN, STAT3, RICTOR	AKT1, AKT2, AKT3, WWTR1, IRS1, RAF1, MAPK8, YAP1, AKT1, TUBA1B, YBX1, ERBB2, NOTCH1, RICTOR, PGR, MAP2K1, STK11, ERBB3, MRE11, BID, COL6A1, KDR, RPS6KA1, CCNE1, ASNS, BAX, PARK7, SYK, EEF2, BIRC2, PRDX1, SRC, SQSTM1, CASP7, ATM, PCNA, STAT5A, COPS5, TFRC, PREX1, SERPINE1, YWHAE, CCNB1, G6PD, CCNE2, EIF4G1, RPS6, PIK3R1, CHEK2, GAPDH, TIGAR, MSH2, NDRG1, CHEK1, ERRFI1, MSH6

**Testicular Germ Cell Cancer**	GSK3A, GSK3B, PREX1, PCNA, ASNS, ITGA2, TGM2, CAV1, ERRFI1, EIF4G1, SMAD5	NF2, CHEK2, EEF2, MSH2, MSH6, ADAR, SMAD3, XRCC5, MTOR, ESR1, CCNB1, MS4A1, PARP1, YWHAZ, RPS6, EEF2K, PRDX1, ACACA, GAPDH, BCL2L11, PDCD4, CCNE2, SCD, CHEK1, RBM15, ACACB, KIT, BAX, ASNS, BRAF, RPTOR, BAP1, EIF4EBP1, CCNE1, GSK3A, GSK3B, RAF1, FOXM1, XRCC1, SRSF1, CASP7, PCNA, EIF4G1, CDKN1B, PRKCD, AKT1S1, SHC1, PXN, EIF4E, RB1, TIGAR, PREX1, SMAD1, CDH3, CLDN7, RPS6KB1, PARK7, AKT1, AKT2, AKT3, PRKCA, MAPK14, MAPK8, YAP1, EGFR, ANXA1, PRKAA1, STK11, RPS6KA1, HSPA1A, FOXO3, CDH2, DIRAS3, BID, MAP2K1, YBX1, FN1, WWTR1, MRE11, CAV1, SRC, BCL2, ACVRL1, PECAM1, NOTCH1, IRS1, ERBB2, CCND1, TSC1, ETS1, IGFBP2, MAPK9, RAD51, MAPK1, MAPK3, PDK1, TP53, TGM2, NFKB1, GATA3, AKT, PGR, PRRT2, PTEN, STAT4, RICTOR	AKT1, AKT2, AKT3, WWTR1, IRS1, RAF1, MAPK8, YAP1, AKT1, TUBA1B, YBX1, ERBB2, NOTCH1, RICTOR, PGR, MAP2K1, STK11, ERBB3, MRE11, BID, COL6A1, KDR, RPS6KA1, CCNE1, ASNS, BAX, PARK7, SYK, EEF2, BIRC2, PRDX1, SRC, SQSTM1, CASP7, ATM, PCNA, STAT5A, COPS5, TFRC, PREX1, SERPINE1, YWHAE, CCNB1, G6PD, CCNE2, EIF4G1, RPS6, PIK3R1, CHEK2, GAPDH, TIGAR, MSH2, NDRG1, CHEK1, ERRFI1, MSH7
**Thyroid Carcinoma**	BCL2L11, COL6A1, PARK7, WWTR1, PDK1, KIT, BID, EGFR, FN1, PGR, IGF1R, RB1, ANXA1, RPS6KA1, ARID1A, PXN, CASP3, RPS6KB1	BCL2L11, ERBB2, TP53BP1, YBX1, KIT, COL6A1, WWTR1, TFF1, MET, AKT1, AKT2, AKT3, MSH6, BCL2, CTNNA1, EGFR, GSK3A, GSK3B, PTK2, SMAD1, TSC2, AKT1S1, SMAD3, LCK, PIK3CA, INPP4B, NOTCH1, FOXO3, CHEK2, YWHAE, EIF4EBP1, RPS6KB1, ANXA1, CCNE1, CDK1, SRC, CASP3, MAPT, CCNB1	SNAI2, KIT, PRKCA, BCL2L11, ARID1A, AKT1, AKT2, AKT3, CCNB1, ACACA, ACACB, RB1, WWTR1, ANXA1, PXN, MAPK14, FN1, XIAP, NEK7, SRC, YAP1, EEF2, GSK3A, GSK3B, IGF1R, BAX, PEA15, CHEK2, BCL2L1
**Uterine Carcinosarcoma**	FOXM1, IGFBP2, BAK1, CDH3, SHC1, CDKN1A, EIF4E, LCK, RAD50	XBP1, YWHAB, CDKN1B, CHEK2, CDKN1A, RAF1, CHEK1, SMAD1, GSK3A, GSK3B, PARK7, PEA15, RPS6KA1, MTOR, IGFBP2, TSC1, YBX1, BAX, TUBA1B, BRAF	ERBB3, XRCC1, STAT3, AKT1, AKT2, AKT3, EIF4EBP1, ERCC1

Key Pathways	Esophageal Carcinoma	Stomach Adenocarcinoma	Colorectal Adenocarcinoma	Pancreatic Adenocarcinoma
Adipocytokine signaling pathway	3.0E-04	5.4E-06	2.6E-06	
AMPK signaling pathway	4.5E-08	5.2E-12	5.7E-14	
Apoptosis			1.9E-12	
B cell receptor signaling pathway	2.6E-04	2.8E-07	6.0E-10	
Calcium signaling pathway				
cAMP signaling pathway	6.2E-02	3.1E-03	7.8E-04	
Cell cycle			2.4E-05	2.7E-03
cGMP-PKG signaling pathway	4.0E-02	5.4E-03	2.0E-04	
Chemokine signaling pathway	1.1E-03	8.4E-07	3.2E-09	4.8E-02
ErbB signaling pathway	3.2E-09	5.6E-17	2.3E-19	1.4E-07
Estrogen signaling pathway	6.3E-05	2.0E-08	1.5E-09	7.6E-02
Fanconi anemia pathway				
Fc epsilon RI signaling pathway	2.9E-04	6.0E-05	1.3E-08	
FoxO signaling pathway	1.5E-06	3.8E-08	2.3E-18	3.9E-04
Glucagon signaling pathway	7.3E-05	2.6E-03		
GnRH signaling pathway		6.5E-02	1.1E-03	
HIF-1 signaling pathway	6.3E-05	8.1E-15	6.5E-20	1.1E-04

**Hippo signaling pathway**		6.1E-02		
**Insulin signaling pathway**	2.9E-10	2.3E-12	4.2E-14	2.7E-02
**Jak-STAT signaling pathway**		2.7E-03	4.0E-04	
**MAPK signaling pathway**	2.9E-02	8.4E-04	3.2E-06	3.0E-02
**mTOR signaling pathway**	1.2E-10	2.8E-18	1.0E-24	1.6E-04
**Neurotrophin signaling pathway**	2.3E-03	1.2E-10	1.5E-17	1.9E-02
**NF-kappa B signaling pathway**			2.6E-02	
**NOD-like receptor signaling pathway**			4.7E-03	
**Oxytocin signaling pathway**	6.5E-03	1.8E-04	7.8E-07	
**p53 signaling pathway**		6.0E-05	2.4E-06	3.9E-03
**PI3K-Akt signaling pathway**	1.5E-05	3.6E-14	1.4E-20	1.1E-03
**Prolactin signaling pathway**	1.4E-05	9.7E-10	1.1E-16	4.6E-03
**Rap1 signaling pathway**	3.6E-04	5.7E-06	7.3E-10	7.9E-02
**Ras signaling pathway**	5.4E-04	2.5E-07	3.4E-11	
**RIG-I-like receptor signaling pathway**				
**Signaling pathways regulating pluripotency of stem cells**	3.7E-04	5.2E-05	1.2E-08	
**Sphingolipid signaling pathway**	1.9E-04	1.9E-07	5.7E-14	
**T cell receptor signaling pathway**	1.3E-02	8.1E-06	6.1E-09	
**TGF-beta signaling pathway**		4.8E-02		5.6E-02
**Thyroid hormone signaling pathway**	1.6E-04	4.9E-11	2.8E-14	1.7E-02
**TNF signaling pathway**	8.6E-02	8.1E-06	6.1E-09	

**Toll-like receptor signaling pathway**	**8.0E-02**	**5.8E-05**	**4.6E-08**	
**VEGF signaling pathway**	**3.9E-07**	**4.6E-10**	**2.7E-14**	**3.3E-02**
**Wnt signaling pathway**			**9.5E-02**	

Adrenocortical Carcinoma	Cholangiocarcinoma	Bladder Urothelial Carcinoma	Breast invasive Carcinoma	Brian Lower grade Glioma	Glioblastoma Multiforme	Cervical squamous cell Carcinoma
	1.5E-03		3.1E-08	3.6E-04		2.6E-02
4.3E-02	3.1E-05	9.7E-04	9.6E-14	5.1E-08	7.5E-02	3.8E-05
	3.3E-05	1.0E-03	7.9E-12	5.7E-07		
	1.0E-03	7.3E-05	2.2E-07	2.5E-09		
	2.7E-02	8.7E-03	1.5E-04	5.5E-05		
4.6E-02	8.1E-08	1.4E-04	3.1E-11	8.0E-08	1.1E-02	4.8E-06
	1.5E-02	2.2E-02	5.4E-04	4.8E-04		
	1.7E-03	8.5E-04	4.3E-08	1.0E-05		1.8E-01
2.9E-06	1.9E-07	3.7E-12	7.2E-28	1.2E-19	1.6E-08	5.0E-06
2.4E-03	1.1E-05	1.2E-07	6.3E-08	8.1E-13	4.7E-02	4.8E-02
7.8E-04	1.3E-06	1.2E-08	2.1E-09	8.8E-11		
4.2E-04	1.1E-10	3.6E-10	8.1E-19	2.6E-16	1.1E-02	7.8E-07
	3.6E-02		2.6E-02	4.7E-02		
2.3E-02	2.2E-03	2.1E-06	3.3E-05	4.4E-07		1.8E-01
5.9E-06	4.2E-10	1.1E-11	3.0E-18	2.1E-18	8.2E-08	9.7E-03

6.5E-02			5.8E-05	2.2E-04		1.1E-03
2.8E-05	2.9E-06	1.7E-07	1.4E-20	3.6E-12	1.7E-03	7.2E-06
	9.3E-02		1.7E-04	2.4E-03		
5.6E-04	9.2E-04	2.1E-04	6.9E-08	1.3E-08		4.1E-02
4.4E-07	2.7E-05	7.0E-11	6.7E-25	1.1E-17	8.3E-05	9.3E-07
3.4E-04	3.1E-05	9.2E-07	9.1E-16	1.4E-11		1.9E-02
			6.9E-02		4.2E-02	
3.7E-04		4.9E-02	2.4E-03	8.9E-03		
6.6E-02	8.5E-02	6.5E-08	7.5E-07	5.4E-07		1.2E-01
	1.5E-03	1.1E-03	9.7E-12	1.3E-07		3.3E-03
2.7E-04	5.2E-07	1.4E-08	1.3E-20	5.6E-19	2.3E-04	1.6E-08
4.4E-05	4.5E-06	4.0E-10	7.7E-20	2.1E-12	3.5E-02	2.7E-02
	2.1E-06	6.0E-09	1.9E-08	1.0E-08		
2.6E-02	4.6E-05	1.4E-08	1.1E-08	2.1E-07	8.0E-03	9.8E-02
5.6E-02	5.7E-05	2.3E-04	1.2E-09	1.5E-08		1.0E-01
4.2E-03	1.4E-06	1.5E-10	1.2E-12	1.2E-11		
3.5E-02	2.5E-04	4.6E-06	2.4E-07	1.3E-07		
				4.4E-03		1.7E-01
2.3E-04	2.1E-05	3.5E-08	7.0E-19	2.5E-13		6.8E-02
3.1E-03	3.3E-04	5.7E-05	2.8E-08	2.4E-07		

**3.4E-02**	**2.7E-04**	**4.9E-04**	**1.3E-06**	**1.6E-07**		
**1.1E-02**	**8.1E-07**	**5.9E-12**	**6.8E-15**	**2.8E-14**		
			**6.1E-04**	**8.1E-04**		

Head and Neck squamous cell Carcinoma	Kindeny Renal clear Carcinoma	Kindeny Renal papillary Carcinoma	Lung Adenocarcinoma	Lung Squamous Cell Carcinoma	Ovarian Serous Cystadenocarcinoma	Prostate Adenocarcinoma
	4.2E-04	7.3E-04		1.5E-04	2.6E-06	1.8E-03
4.4E-04	4.0E-10	6.5E-13	3.5E-02	2.2E-12	6.6E-10	2.2E-06
9.4E-04	2.4E-06	1.4E-06	6.6E-02	6.8E-06	7.7E-07	5.2E-03
1.8E-02	3.0E-05	3.1E-07	7.4E-04	5.1E-07	5.0E-08	1.7E-04
9.0E-02						
	3.1E-02	6.0E-04	3.1E-02	3.4E-03	1.3E-04	3.0E-02
6.4E-03	1.0E-08	1.8E-09	9.5E-05	3.0E-04	5.0E-03	2.0E-07
8.2E-02	5.9E-02	5.6E-03	1.7E-02	9.6E-04	1.3E-02	
	1.5E-04	6.6E-10	7.4E-05	4.4E-06	3.1E-06	3.3E-05
6.5E-11	5.1E-08	6.3E-20	3.2E-08	1.5E-14	1.5E-14	9.0E-17
3.3E-02	1.6E-06	2.1E-08	2.4E-04	2.0E-08	4.3E-08	1.6E-05
3.6E-05	3.5E-05	1.1E-08	6.0E-05	6.2E-06	1.1E-06	9.4E-07
1.5E-06	4.1E-12	1.8E-16	3.1E-09	6.6E-11	4.6E-07	2.2E-12
	1.1E-02	6.4E-02		5.0E-03	1.6E-02	
	5.2E-02	1.4E-05	2.4E-03	3.3E-04	1.1E-02	4.4E-04
1.7E-08	2.5E-11	2.2E-14	1.6E-13	1.8E-10	1.0E-10	8.9E-12

	1.1E-02	1.5E-02		2.4E-02		3.7E-04
2.7E-06	2.0E-09	5.1E-19	1.3E-06	2.3E-10	1.0E-10	2.1E-13
7.5E-02	3.9E-02	2.0E-02		9.6E-02	1.2E-02	
3.7E-02	7.7E-03	7.6E-08	4.2E-03	6.0E-05	6.5E-06	6.7E-04
2.5E-08	5.4E-11	3.4E-18	1.7E-14	1.5E-14	8.3E-17	2.5E-12
2.3E-05	9.9E-08	2.7E-13	8.3E-05	3.3E-11	1.3E-08	1.3E-06
2.9E-02						8.6E-02
	8.5E-02	9.0E-02	4.3E-02	5.5E-02	3.2E-02	3.2E-03
7.5E-02	3.3E-03	1.9E-06	4.1E-04	9.8E-05	1.9E-04	7.8E-05
2.1E-02	4.4E-10	2.2E-07	6.0E-05	2.1E-03	7.3E-03	1.1E-06
4.9E-06	4.2E-14	2.9E-17	4.7E-10	1.6E-08	2.0E-09	9.9E-16
8.7E-05	8.0E-09	7.8E-14	1.5E-07	2.0E-10	3.9E-10	5.0E-10
2.1E-02	5.6E-04	3.1E-05	3.3E-05	1.2E-05	1.1E-07	9.7E-07
2.7E-02	4.7E-06	8.2E-06	2.4E-03	1.6E-04	7.8E-11	1.8E-06
					7.2E-02	
7.7E-03	1.3E-03	7.6E-08	1.3E-06	4.9E-07	9.5E-05	5.1E-06
2.1E-05	7.8E-08	1.6E-09	6.1E-06	1.8E-06	2.2E-07	1.3E-05
3.8E-02	2.5E-03	6.8E-06	3.8E-05	4.4E-08	1.1E-06	1.5E-03
		1.0E-03	9.0E-02	2.5E-02		7.0E-02
2.9E-04	6.4E-07	5.8E-15	4.6E-06	4.5E-09	6.9E-09	7.2E-09
3.8E-03	2.5E-03	4.5E-04	3.8E-05	8.3E-07	1.0E-07	1.3E-03

3.4E-03	2.1E-03	6.2E-04	3.5E-04	8.1E-06	6.9E-08	9.3E-03
6.4E-04	2.6E-04	4.4E-09	1.0E-10	2.0E-10	2.2E-08	6.0E-06
6.1E-02		9.3E-03		7.3E-02	5.9E-03	4.9E-03

Skin Cutaneous Melanoma	Sarcoma	Testicular Germ Cell Cancer	Thymoma	Thyroid Carcinoma	Endometrial Carcinoma	Uterine Carcinosarcoma
1.0E-04	1.8E-03	1.0E-08	2.1E-02	1.5E-02	6.7E-06	2.2E-05
3.7E-09	2.5E-08	1.8E-13	1.6E-02	1.1E-09	3.4E-09	2.6E-04
5.6E-06	5.4E-06	7.3E-09		7.4E-11	2.0E-06	3.4E-04
5.1E-06	1.5E-06	8.2E-08	7.1E-07	1.9E-03	1.5E-07	1.8E-05
2.9E-04	2.3E-03	2.4E-04	2.5E-02		3.4E-04	2.5E-03
1.3E-06	1.4E-05	6.5E-10	5.0E-08	6.0E-06	8.6E-08	3.2E-04
7.0E-04	3.7E-03	4.0E-03			4.3E-03	4.6E-02
1.5E-07	9.2E-08	1.2E-09	6.7E-06	9.6E-05	1.0E-06	1.0E-07
1.5E-13	3.4E-18	3.1E-20	7.4E-14	2.2E-11	8.0E-17	4.9E-12
7.9E-09	1.7E-06	1.9E-08	3.8E-09	8.0E-04	8.3E-09	1.1E-03
						2.6E-02
1.5E-08	9.4E-07	8.6E-09	3.2E-08	1.5E-03	2.9E-06	4.8E-03
4.3E-14	1.5E-13	3.0E-21	5.9E-14	4.4E-08	1.0E-12	2.3E-06
2.6E-02	5.2E-03	1.8E-02		1.0E-02	2.4E-02	8.2E-02
3.4E-03	4.4E-04	1.0E-04	3.0E-07		2.0E-03	
1.7E-14	2.8E-14	1.3E-20	1.1E-06	1.5E-10	5.3E-10	4.3E-10

1.8E-02	1.9E-03	1.2E-04	2.0E-05	1.9E-05	1.4E-02	
1.5E-08	4.2E-17	4.2E-18	3.2E-11	8.7E-07	2.5E-15	5.8E-10
3.8E-05		2.4E-02		2.1E-02	8.8E-02	3.2E-02
2.5E-04	1.5E-04	9.2E-06	2.4E-05	6.0E-04	1.7E-04	1.4E-03
2.8E-09	8.9E-17	2.6E-24	2.9E-07	3.0E-10	6.7E-16	2.1E-10
7.8E-08	6.3E-12	5.0E-18	1.3E-10	2.2E-09	1.8E-10	4.4E-09
	8.6E-02	5.0E-02		4.5E-03		
	2.3E-02	1.9E-04	7.8E-03		4.2E-02	
1.0E-05	2.2E-03	4.4E-05	3.4E-06	3.4E-02	4.4E-04	
1.1E-02	1.3E-05	8.6E-09	1.6E-06	1.6E-06	6.0E-10	4.8E-03
1.3E-12	9.5E-15	2.1E-22	9.6E-12	3.2E-14	3.9E-16	3.2E-08
1.1E-14	2.6E-11	2.5E-19	6.5E-10	8.1E-07	1.7E-09	3.3E-08
7.9E-09	6.1E-06	4.6E-06	3.1E-05	4.9E-06	3.0E-04	1.9E-02
2.0E-07	2.8E-04	4.2E-07	7.8E-06	6.3E-05	8.6E-06	4.8E-03
		9.4E-02				
1.5E-08	7.2E-08	1.7E-07	1.1E-07	9.0E-06	1.7E-07	4.6E-05
6.3E-08	1.6E-06	2.4E-12	3.8E-07	3.1E-06	5.3E-08	2.6E-04
5.3E-06	3.7E-07	5.2E-06	1.2E-06	1.3E-04	3.2E-06	1.1E-05
	7.0E-02	9.5E-03	2.6E-02		9.2E-02	
1.2E-10	2.9E-12	1.7E-10	2.1E-07	2.3E-06	7.7E-11	2.1E-04
2.3E-08	2.0E-04	7.4E-08	1.7E-03	1.4E-03	3.5E-07	1.5E-02

**2.8E-07**	**1.8E-03**	**3.7E-06**	**1.3E-03**	**8.2E-03**	**2.4E-07**	**1.3E-02**
**2.5E-10**	**1.5E-09**	**2.1E-10**	**3.2E-07**	**3.3E-07**	**3.2E-05**	**3.8E-03**
		**2.0E-02**	**4.0E-03**		**1.5E-03**	

Lymphoid Neoplasm Diffuse Large B-Cell Lymphoma	Pheochromocytoma and Paraganglioma
1.6E-07	
2.6E-12	1.2E-05
6.1E-08	7.8E-03
1.4E-07	1.1E-02
1.8E-06	
	1.3E-03
5.0E-05	
8.9E-08	7.1E-03
1.4E-22	7.2E-08
1.8E-10	3.8E-03
1.1E-07	4.4E-06
1.2E-14	3.2E-11
1.4E-04	2.4E-04
7.5E-12	4.2E-05

	3.0E-03
6.2E-16	2.0E-07
6.0E-02	
1.9E-06	2.6E-02
2.0E-14	6.8E-03
1.7E-16	1.5E-05
2.4E-04	4.6E-02
2.0E-03	5.7E-05
8.4E-03	1.2E-03
2.8E-13	2.8E-08
6.5E-15	2.2E-08
3.5E-07	4.6E-04
6.9E-09	1.3E-04
1.5E-05	2.8E-04
2.5E-08	1.2E-05
2.8E-06	6.8E-04
8.7E-02	
3.3E-11	8.3E-04
2.8E-06	5.4E-03

**3.2E-05**	**3.2E-02**
**4.3E-08**	**3.9E-05**
	**6.1E-02**

Table S3: Results for M^6A sit prediction numbers.									
**Gene**	**Very high confidence**	**High confidence**	**Moderate confidence**	**Low confidence**	**Gene**	**Very high confidence**	**High confidence**	**Moderate confidence**	**Low confidence**
**AKT1**	0	4	3	3	**MAPK3**	0	2	1	0
**AKT1S1**	2	1	0	0	**MAPK14**	2	5	5	3
**ATM**	6	19	15	22	**MTOR**	5	4	7	8
**AKT2**	4	0	1	3	**MYC**	18	2	1	2
**AKT3**	4	8	6	8	**NRAS**	0	3	6	4
**BAX**	1	0	0	1	**PIK3CA**	4	6	9	6
**BCL2**	12	2	3	3	**PIK3R1**	15	2	5	7
**BCL2L1**	15	0	3	0	**PIK3R2**	1	0	5	2
**BCL2L11**	1	1	6	6	**PRKAA1**	2	0	1	1
**BID**	4	4	2	4	**PRKCA**	4	5	8	8
**BRAF**	0	4	4	4	**PRKCB**	0	8	9	10
**CASP3**	2	3	3	2	**RAF1**	12	0	2	0
**CASP8**	8	4	2	1	**PTEN**	6	4	5	7
**CCNB1**	5	3	3	1	**RICTOR**	8	5	11	5
**CCNE1**	1	1	0	2	**RPTOR**	1	4	6	6
**CCNE2**	0	2	5	4	**RPS6**	1	0	2	2
**CDK1**	1	1	1	3	**RPS6KA1**	0	0	0	0
**CDKN1B**	1	2	1	1	**RPS6KB1**	7	3	3	3
**CDKN2A**	0	0	0	0	**SERPINE1**	1	3	2	6
**CHEK1**	0	2	4	1	**STAT3**	0	4	3	6
**CHEK2**	1	2	2	3	**STK11**	6	1	1	0
**EIF4EBP1**	0	1	0	0	**STMN1**	0	4	3	1
**FOXO3**	3	3	10	9	**SYK**	1	6	4	4
**GSK3B**	2	7	4	6	**TP53**	0	0	0	2
**IRS1**	22	2	2	5	**TP53BP1**	8	11	7	12
**JUN**	5	7	2	3	**TSC1**	11	1	7	4
**KDR**	3	6	9	8	**TSC2**	0	3	5	9
**MAP2K1**	0	3	1	1	**YWHAE**	3	2	0	2
**MAPK1**	0	0	0	0					

Cancer Type	Sample_Id	PI3K-Akt	mTOR	FOXO	MAPK	P53
Adrenocortical carcinoma	TCGA-OR-A5J1	1	1	1	0	1
Adrenocortical carcinoma	TCGA-OR-A5J2	0	1	1	1	1
Adrenocortical carcinoma	TCGA-OR-A5J3	0	0	0	1	1
Adrenocortical carcinoma	TCGA-OR-A5J5	1	0	0	0	1
Adrenocortical carcinoma	TCGA-OR-A5J6	1	1	0	1	1
Adrenocortical carcinoma	TCGA-OR-A5J7	1	1	1	1	1
Adrenocortical carcinoma	TCGA-OR-A5J8	1	1	1	1	1
Adrenocortical carcinoma	TCGA-OR-A5J9	0	1	0	1	1
Adrenocortical carcinoma	TCGA-OR-A5JA	1	1	1	1	1
Adrenocortical carcinoma	TCGA-OR-A5JB	1	1	1	1	1
Adrenocortical carcinoma	TCGA-OR-A5JC	1	1	1	1	1
Adrenocortical carcinoma	TCGA-OR-A5JD	1	1	1	1	1
Adrenocortical carcinoma	TCGA-OR-A5JE	0	1	1	1	1
Adrenocortical carcinoma	TCGA-OR-A5JF	0	0	0	0	0
Adrenocortical carcinoma	TCGA-OR-A5JG	1	1	1	1	1
Adrenocortical carcinoma	TCGA-OR-A5JI	1	1	0	1	1
Adrenocortical carcinoma	TCGA-OR-A5JJ	0	1	1	0	1
Adrenocortical carcinoma	TCGA-OR-A5JK	1	1	1	1	1
Adrenocortical carcinoma	TCGA-OR-A5JL	1	1	1	1	1
Adrenocortical carcinoma	TCGA-OR-A5JM	1	1	1	1	1
Adrenocortical carcinoma	TCGA-OR-A5JO	0	0	0	0	0
Adrenocortical carcinoma	TCGA-OR-A5JP	1	1	1	1	1
Adrenocortical carcinoma	TCGA-OR-A5JQ	1	1	1	1	1
Adrenocortical carcinoma	TCGA-OR-A5JR	0	0	0	1	1
Adrenocortical carcinoma	TCGA-OR-A5JS	1	1	1	1	1
Adrenocortical carcinoma	TCGA-OR-A5JT	1	1	0	0	1
Adrenocortical carcinoma	TCGA-OR-A5JV	1	1	1	1	1
Adrenocortical carcinoma	TCGA-OR-A5JW	1	1	1	1	1
Adrenocortical carcinoma	TCGA-OR-A5JX	1	1	1	1	1
Adrenocortical carcinoma	TCGA-OR-A5JY	1	1	1	1	1
Adrenocortical carcinoma	TCGA-OR-A5JZ	0	1	0	0	0
Adrenocortical carcinoma	TCGA-OR-A5K0	1	1	1	1	1
Adrenocortical carcinoma	TCGA-OR-A5K1	0	0	0	0	0
Adrenocortical carcinoma	TCGA-OR-A5K2	1	1	1	1	1
Adrenocortical carcinoma	TCGA-OR-A5K3	0	0	0	0	0
Adrenocortical carcinoma	TCGA-OR-A5K4	0	0	1	0	1
Adrenocortical carcinoma	TCGA-OR-A5K5	0	1	1	1	1
Adrenocortical carcinoma	TCGA-OR-A5K6	1	1	0	1	1
Adrenocortical carcinoma	TCGA-OR-A5K8	0	0	0	0	0
Adrenocortical carcinoma	TCGA-OR-A5K9	1	1	1	1	1
Adrenocortical carcinoma	TCGA-OR-A5KO	1	1	0	1	1
Adrenocortical carcinoma	TCGA-OR-A5KT	1	1	1	1	1
Adrenocortical carcinoma	TCGA-OR-A5KU	0	0	0	0	0
Adrenocortical carcinoma	TCGA-OR-A5KV	0	0	0	0	0
Adrenocortical carcinoma	TCGA-OR-A5KW	1	1	0	0	1
Adrenocortical carcinoma	TCGA-OR-A5KX	0	1	1	1	1
Adrenocortical carcinoma	TCGA-OR-A5KY	1	1	1	1	1
Adrenocortical carcinoma	TCGA-OR-A5KZ	1	1	1	1	1
Adrenocortical carcinoma	TCGA-OR-A5L3	1	1	0	1	1
Adrenocortical carcinoma	TCGA-OR-A5L4	0	0	0	0	0
Adrenocortical carcinoma	TCGA-OR-A5L5	0	0	0	0	0
Adrenocortical carcinoma	TCGA-OR-A5L6	1	1	1	1	1
Adrenocortical carcinoma	TCGA-OR-A5L8	0	0	0	0	0
Adrenocortical carcinoma	TCGA-OR-A5L9	0	0	0	0	0
Adrenocortical carcinoma	TCGA-OR-A5LA	1	0	0	0	0
Adrenocortical carcinoma	TCGA-OR-A5LB	0	1	1	1	1
Adrenocortical carcinoma	TCGA-OR-A5LC	1	1	1	1	1

Adrenocortical carcinoma	TCGA-OR-A5LD	1	0	1	1	1
Adrenocortical carcinoma	TCGA-OR-A5LE	1	1	1	0	1
Adrenocortical carcinoma	TCGA-OR-A5LG	1	1	1	1	1
Adrenocortical carcinoma	TCGA-OR-A5LH	0	0	0	0	0
Adrenocortical carcinoma	TCGA-OR-A5LJ	1	0	0	0	1
Adrenocortical carcinoma	TCGA-OR-A5LK	1	0	0	0	0
Adrenocortical carcinoma	TCGA-OR-A5LL	1	1	1	1	1
Adrenocortical carcinoma	TCGA-OR-A5LM	1	1	1	1	1
Adrenocortical carcinoma	TCGA-OR-A5LN	1	1	1	1	1
Adrenocortical carcinoma	TCGA-OR-A5LO	0	0	1	0	0
Adrenocortical carcinoma	TCGA-OR-A5LP	0	0	1	0	0
Adrenocortical carcinoma	TCGA-OR-A5LR	0	0	0	0	0
Adrenocortical carcinoma	TCGA-OR-A5LS	1	1	1	1	1
Adrenocortical carcinoma	TCGA-OR-A5LT	1	1	1	1	1
Adrenocortical carcinoma	TCGA-OU-A5PI	1	1	1	1	1
Adrenocortical carcinoma	TCGA-P6-A5OF	0	0	0	0	0
Adrenocortical carcinoma	TCGA-P6-A5OG	1	1	1	1	1
Adrenocortical carcinoma	TCGA-PA-A5YG	0	0	0	0	0
Adrenocortical carcinoma	TCGA-PK-A5H8	0	0	0	0	0
Adrenocortical carcinoma	TCGA-PK-A5H9	0	0	0	1	1
Adrenocortical carcinoma	TCGA-PK-A5HA	1	1	1	1	1
Adrenocortical carcinoma	TCGA-PK-A5HB	1	1	1	1	1
Acute Myeloid Leukemia	TCGA-AB-2803	0	0	0	0	0
Acute Myeloid Leukemia	TCGA-AB-2805	0	0	0	1	1
Acute Myeloid Leukemia	TCGA-AB-2806	0	0	0	0	0
Acute Myeloid Leukemia	TCGA-AB-2807	0	0	0	0	0
Acute Myeloid Leukemia	TCGA-AB-2808	0	0	0	1	0
Acute Myeloid Leukemia	TCGA-AB-2810	0	0	0	0	0
Acute Myeloid Leukemia	TCGA-AB-2811	0	0	0	0	0
Acute Myeloid Leukemia	TCGA-AB-2812	0	0	0	0	0
Acute Myeloid Leukemia	TCGA-AB-2813	1	1	1	1	1
Acute Myeloid Leukemia	TCGA-AB-2814	0	0	0	0	0
Acute Myeloid Leukemia	TCGA-AB-2815	0	0	0	0	0
Acute Myeloid Leukemia	TCGA-AB-2816	0	0	0	1	0
Acute Myeloid Leukemia	TCGA-AB-2817	0	0	0	1	1
Acute Myeloid Leukemia	TCGA-AB-2818	0	0	0	0	0
Acute Myeloid Leukemia	TCGA-AB-2819	0	0	0	0	0
Acute Myeloid Leukemia	TCGA-AB-2820	0	0	0	0	1
Acute Myeloid Leukemia	TCGA-AB-2821	0	0	0	0	0
Acute Myeloid Leukemia	TCGA-AB-2822	0	0	0	0	0
Acute Myeloid Leukemia	TCGA-AB-2823	1	0	0	1	0
Acute Myeloid Leukemia	TCGA-AB-2824	0	0	0	0	0
Acute Myeloid Leukemia	TCGA-AB-2825	0	0	0	0	0
Acute Myeloid Leukemia	TCGA-AB-2826	1	0	0	0	0
Acute Myeloid Leukemia	TCGA-AB-2828	0	0	0	0	0
Acute Myeloid Leukemia	TCGA-AB-2830	0	0	0	0	0
Acute Myeloid Leukemia	TCGA-AB-2832	0	0	0	0	0
Acute Myeloid Leukemia	TCGA-AB-2833	0	0	0	0	0
Acute Myeloid Leukemia	TCGA-AB-2834	0	0	0	0	0
Acute Myeloid Leukemia	TCGA-AB-2835	0	0	0	0	0
Acute Myeloid Leukemia	TCGA-AB-2836	0	0	0	0	0
Acute Myeloid Leukemia	TCGA-AB-2837	0	0	0	0	0
Acute Myeloid Leukemia	TCGA-AB-2838	0	0	1	1	1
Acute Myeloid Leukemia	TCGA-AB-2839	1	0	0	1	0
Acute Myeloid Leukemia	TCGA-AB-2840	0	0	0	0	0
Acute Myeloid Leukemia	TCGA-AB-2841	0	0	0	0	0
Acute Myeloid Leukemia	TCGA-AB-2842	0	0	0	0	0
Acute Myeloid Leukemia	TCGA-AB-2843	0	0	0	0	0

Acute Myeloid Leukemia	TCGA-AB-2844	0	0	0	0	0
Acute Myeloid Leukemia	TCGA-AB-2845	0	0	0	0	0
Acute Myeloid Leukemia	TCGA-AB-2846	0	0	0	0	0
Acute Myeloid Leukemia	TCGA-AB-2847	0	0	0	0	0
Acute Myeloid Leukemia	TCGA-AB-2848	0	0	0	0	0
Acute Myeloid Leukemia	TCGA-AB-2849	0	0	1	0	1
Acute Myeloid Leukemia	TCGA-AB-2851	0	0	0	0	0
Acute Myeloid Leukemia	TCGA-AB-2853	0	0	0	0	0
Acute Myeloid Leukemia	TCGA-AB-2854	0	0	0	0	0
Acute Myeloid Leukemia	TCGA-AB-2855	1	0	1	0	1
Acute Myeloid Leukemia	TCGA-AB-2856	0	0	0	0	0
Acute Myeloid Leukemia	TCGA-AB-2857	1	1	1	1	1
Acute Myeloid Leukemia	TCGA-AB-2858	0	0	0	0	0
Acute Myeloid Leukemia	TCGA-AB-2859	0	0	0	0	0
Acute Myeloid Leukemia	TCGA-AB-2860	0	1	1	1	1
Acute Myeloid Leukemia	TCGA-AB-2861	0	0	0	0	1
Acute Myeloid Leukemia	TCGA-AB-2862	0	0	0	0	0
Acute Myeloid Leukemia	TCGA-AB-2863	0	0	0	0	0
Acute Myeloid Leukemia	TCGA-AB-2865	0	0	0	0	0
Acute Myeloid Leukemia	TCGA-AB-2866	0	0	0	0	0
Acute Myeloid Leukemia	TCGA-AB-2867	0	0	0	0	0
Acute Myeloid Leukemia	TCGA-AB-2868	1	1	1	0	1
Acute Myeloid Leukemia	TCGA-AB-2869	0	0	0	0	0
Acute Myeloid Leukemia	TCGA-AB-2870	0	0	0	1	0
Acute Myeloid Leukemia	TCGA-AB-2871	0	0	0	0	0
Acute Myeloid Leukemia	TCGA-AB-2872	1	0	0	0	0
Acute Myeloid Leukemia	TCGA-AB-2873	0	0	0	0	0
Acute Myeloid Leukemia	TCGA-AB-2874	0	0	0	1	1
Acute Myeloid Leukemia	TCGA-AB-2875	0	0	0	0	0
Acute Myeloid Leukemia	TCGA-AB-2876	0	0	0	0	0
Acute Myeloid Leukemia	TCGA-AB-2877	0	0	0	0	0
Acute Myeloid Leukemia	TCGA-AB-2878	0	0	0	0	0
Acute Myeloid Leukemia	TCGA-AB-2879	0	0	0	0	0
Acute Myeloid Leukemia	TCGA-AB-2880	0	0	0	0	0
Acute Myeloid Leukemia	TCGA-AB-2881	0	0	0	0	0
Acute Myeloid Leukemia	TCGA-AB-2882	0	0	0	1	1
Acute Myeloid Leukemia	TCGA-AB-2883	0	0	0	0	0
Acute Myeloid Leukemia	TCGA-AB-2884	0	0	0	0	0
Acute Myeloid Leukemia	TCGA-AB-2885	1	1	1	1	1
Acute Myeloid Leukemia	TCGA-AB-2886	1	0	0	0	0
Acute Myeloid Leukemia	TCGA-AB-2887	0	0	0	1	1
Acute Myeloid Leukemia	TCGA-AB-2888	0	0	0	0	0
Acute Myeloid Leukemia	TCGA-AB-2889	0	0	1	0	0
Acute Myeloid Leukemia	TCGA-AB-2890	0	0	0	0	0
Acute Myeloid Leukemia	TCGA-AB-2891	0	0	0	0	0
Acute Myeloid Leukemia	TCGA-AB-2892	0	0	0	0	0
Acute Myeloid Leukemia	TCGA-AB-2893	0	0	0	0	0
Acute Myeloid Leukemia	TCGA-AB-2894	0	0	0	0	0
Acute Myeloid Leukemia	TCGA-AB-2895	0	0	0	0	0
Acute Myeloid Leukemia	TCGA-AB-2896	0	0	0	0	0
Acute Myeloid Leukemia	TCGA-AB-2897	0	0	1	0	0
Acute Myeloid Leukemia	TCGA-AB-2898	0	0	0	0	0
Acute Myeloid Leukemia	TCGA-AB-2899	1	0	1	0	0
Acute Myeloid Leukemia	TCGA-AB-2900	0	0	0	0	0
Acute Myeloid Leukemia	TCGA-AB-2901	0	0	0	0	0
Acute Myeloid Leukemia	TCGA-AB-2903	0	0	0	0	0
Acute Myeloid Leukemia	TCGA-AB-2904	0	0	1	0	1
Acute Myeloid Leukemia	TCGA-AB-2908	0	1	1	0	1

Acute Myeloid Leukemia	TCGA-AB-2909	0	0	0	0	0
Acute Myeloid Leukemia	TCGA-AB-2910	0	0	0	0	0
Acute Myeloid Leukemia	TCGA-AB-2911	0	0	0	0	0
Acute Myeloid Leukemia	TCGA-AB-2912	0	0	0	0	0
Acute Myeloid Leukemia	TCGA-AB-2913	0	0	0	0	0
Acute Myeloid Leukemia	TCGA-AB-2914	0	0	0	0	0
Acute Myeloid Leukemia	TCGA-AB-2915	1	0	0	1	1
Acute Myeloid Leukemia	TCGA-AB-2916	0	0	0	0	0
Acute Myeloid Leukemia	TCGA-AB-2917	0	0	1	1	1
Acute Myeloid Leukemia	TCGA-AB-2918	0	0	0	0	0
Acute Myeloid Leukemia	TCGA-AB-2919	0	0	0	0	0
Acute Myeloid Leukemia	TCGA-AB-2920	1	1	1	1	1
Acute Myeloid Leukemia	TCGA-AB-2921	0	0	0	0	0
Acute Myeloid Leukemia	TCGA-AB-2924	0	0	0	0	0
Acute Myeloid Leukemia	TCGA-AB-2925	0	0	0	0	0
Acute Myeloid Leukemia	TCGA-AB-2927	0	0	0	0	0
Acute Myeloid Leukemia	TCGA-AB-2928	0	0	0	1	1
Acute Myeloid Leukemia	TCGA-AB-2929	1	0	1	1	1
Acute Myeloid Leukemia	TCGA-AB-2930	0	0	0	0	0
Acute Myeloid Leukemia	TCGA-AB-2931	0	0	0	0	0
Acute Myeloid Leukemia	TCGA-AB-2932	0	0	0	1	0
Acute Myeloid Leukemia	TCGA-AB-2933	0	0	1	0	1
Acute Myeloid Leukemia	TCGA-AB-2934	0	0	0	0	0
Acute Myeloid Leukemia	TCGA-AB-2935	1	1	1	1	1
Acute Myeloid Leukemia	TCGA-AB-2936	0	0	0	0	0
Acute Myeloid Leukemia	TCGA-AB-2937	0	0	0	0	0
Acute Myeloid Leukemia	TCGA-AB-2938	1	0	1	0	1
Acute Myeloid Leukemia	TCGA-AB-2939	0	1	0	1	1
Acute Myeloid Leukemia	TCGA-AB-2940	0	0	0	0	0
Acute Myeloid Leukemia	TCGA-AB-2941	1	1	1	1	1
Acute Myeloid Leukemia	TCGA-AB-2942	0	0	0	0	0
Acute Myeloid Leukemia	TCGA-AB-2943	1	1	1	0	1
Acute Myeloid Leukemia	TCGA-AB-2944	0	0	0	0	0
Acute Myeloid Leukemia	TCGA-AB-2946	1	0	1	0	0
Acute Myeloid Leukemia	TCGA-AB-2948	0	0	0	0	0
Acute Myeloid Leukemia	TCGA-AB-2949	0	0	0	1	1
Acute Myeloid Leukemia	TCGA-AB-2950	0	0	0	0	0
Acute Myeloid Leukemia	TCGA-AB-2952	1	0	1	1	1
Acute Myeloid Leukemia	TCGA-AB-2954	0	0	0	0	0
Acute Myeloid Leukemia	TCGA-AB-2955	1	1	1	1	1
Acute Myeloid Leukemia	TCGA-AB-2956	0	0	0	0	0
Acute Myeloid Leukemia	TCGA-AB-2959	0	0	0	0	0
Acute Myeloid Leukemia	TCGA-AB-2963	0	0	0	0	0
Acute Myeloid Leukemia	TCGA-AB-2964	0	0	0	0	0
Acute Myeloid Leukemia	TCGA-AB-2965	0	0	0	0	0
Acute Myeloid Leukemia	TCGA-AB-2966	0	0	0	0	0
Acute Myeloid Leukemia	TCGA-AB-2967	0	0	0	1	0
Acute Myeloid Leukemia	TCGA-AB-2969	0	0	0	0	0
Acute Myeloid Leukemia	TCGA-AB-2970	0	0	0	0	0
Acute Myeloid Leukemia	TCGA-AB-2971	0	0	0	1	0
Acute Myeloid Leukemia	TCGA-AB-2972	0	0	0	0	0
Acute Myeloid Leukemia	TCGA-AB-2973	0	0	0	0	0
Acute Myeloid Leukemia	TCGA-AB-2975	0	0	0	0	0
Acute Myeloid Leukemia	TCGA-AB-2976	0	0	0	0	0
Acute Myeloid Leukemia	TCGA-AB-2977	0	0	0	0	0
Acute Myeloid Leukemia	TCGA-AB-2978	0	0	0	1	0
Acute Myeloid Leukemia	TCGA-AB-2979	0	0	0	0	0
Acute Myeloid Leukemia	TCGA-AB-2980	0	0	0	0	0

Acute Myeloid Leukemia	TCGA-AB-2981	0	0	0	0	0
Acute Myeloid Leukemia	TCGA-AB-2982	0	0	0	0	0
Acute Myeloid Leukemia	TCGA-AB-2983	0	0	0	0	0
Acute Myeloid Leukemia	TCGA-AB-2984	0	0	0	1	0
Acute Myeloid Leukemia	TCGA-AB-2985	0	0	0	1	0
Acute Myeloid Leukemia	TCGA-AB-2986	0	0	0	0	0
Acute Myeloid Leukemia	TCGA-AB-2987	0	0	0	0	0
Acute Myeloid Leukemia	TCGA-AB-2988	1	0	0	0	0
Acute Myeloid Leukemia	TCGA-AB-2990	0	0	0	0	0
Acute Myeloid Leukemia	TCGA-AB-2991	0	0	0	0	0
Acute Myeloid Leukemia	TCGA-AB-2992	0	0	0	0	0
Acute Myeloid Leukemia	TCGA-AB-2993	0	0	0	0	0
Acute Myeloid Leukemia	TCGA-AB-2994	0	0	0	0	0
Acute Myeloid Leukemia	TCGA-AB-2995	0	0	0	0	0
Acute Myeloid Leukemia	TCGA-AB-2996	0	0	0	0	0
Acute Myeloid Leukemia	TCGA-AB-2998	0	0	0	0	0
Acute Myeloid Leukemia	TCGA-AB-2999	0	0	0	0	0
Acute Myeloid Leukemia	TCGA-AB-3000	0	0	0	0	0
Acute Myeloid Leukemia	TCGA-AB-3001	0	0	0	0	0
Acute Myeloid Leukemia	TCGA-AB-3002	0	0	0	0	0
Acute Myeloid Leukemia	TCGA-AB-3005	0	0	0	0	0
Acute Myeloid Leukemia	TCGA-AB-3006	0	0	0	0	0
Acute Myeloid Leukemia	TCGA-AB-3007	0	0	0	1	0
Acute Myeloid Leukemia	TCGA-AB-3008	0	0	0	0	0
Acute Myeloid Leukemia	TCGA-AB-3009	0	0	0	0	0
Acute Myeloid Leukemia	TCGA-AB-3011	0	0	0	0	0
Acute Myeloid Leukemia	TCGA-AB-3012	0	0	0	0	0
Acute Myeloid Leukemia	TCGA-2W-A8YY	0	0	0	0	1
Acute Myeloid Leukemia	TCGA-3X-AAV9	1	1	1	1	1
Acute Myeloid Leukemia	TCGA-3L-AA1B	1	0	1	0	1
Bladder Urothelial carcinoma	TCGA-2F-A9KO	1	1	1	1	1
Bladder Urothelial carcinoma	TCGA-2F-A9KP	1	1	1	1	1
Bladder Urothelial carcinoma	TCGA-2F-A9KQ	1	1	1	0	1
Bladder Urothelial carcinoma	TCGA-2F-A9KR	1	1	1	1	1
Bladder Urothelial carcinoma	TCGA-2F-A9KT	1	1	1	1	1
Bladder Urothelial carcinoma	TCGA-2F-A9KW	1	1	1	1	1
Bladder Urothelial carcinoma	TCGA-4Z-AA7M	1	1	1	1	1
Bladder Urothelial carcinoma	TCGA-4Z-AA7N	0	0	0	0	0
Bladder Urothelial carcinoma	TCGA-4Z-AA7O	1	1	1	0	1
Bladder Urothelial carcinoma	TCGA-4Z-AA7Q	1	1	1	1	1
Bladder Urothelial carcinoma	TCGA-4Z-AA7R	1	1	1	1	1
Bladder Urothelial carcinoma	TCGA-4Z-AA7S	1	1	1	1	1
Bladder Urothelial carcinoma	TCGA-4Z-AA7W	1	1	1	1	1
Bladder Urothelial carcinoma	TCGA-4Z-AA7Y	1	1	0	0	1
Bladder Urothelial carcinoma	TCGA-4Z-AA80	1	1	1	1	1
Bladder Urothelial carcinoma	TCGA-4Z-AA81	1	1	1	1	1
Bladder Urothelial carcinoma	TCGA-4Z-AA82	1	1	1	1	1
Bladder Urothelial carcinoma	TCGA-4Z-AA83	1	1	0	0	0
Bladder Urothelial carcinoma	TCGA-4Z-AA84	1	1	1	1	1
Bladder Urothelial carcinoma	TCGA-4Z-AA86	1	1	1	1	1
Bladder Urothelial carcinoma	TCGA-4Z-AA87	1	1	1	1	1
Bladder Urothelial carcinoma	TCGA-4Z-AA89	1	1	0	0	1
Bladder Urothelial carcinoma	TCGA-5N-A9KI	1	1	1	1	1
Bladder Urothelial carcinoma	TCGA-5N-A9KM	1	1	1	1	1
Bladder Urothelial carcinoma	TCGA-BL-A0C8	1	1	1	1	1
Bladder Urothelial carcinoma	TCGA-BL-A13I	0	0	0	0	1
Bladder Urothelial carcinoma	TCGA-BL-A13J	1	1	0	0	1
Bladder Urothelial carcinoma	TCGA-BL-A3JM	1	0	1	0	1

Bladder Urothelial carcinoma	TCGA-BL-A5ZZ	1	0	1	0	1
Bladder Urothelial carcinoma	TCGA-BT-A0S7	1	1	1	1	1
Bladder Urothelial carcinoma	TCGA-BT-A0YX	1	1	0	0	1
Bladder Urothelial carcinoma	TCGA-BT-A20J	1	1	1	1	1
Bladder Urothelial carcinoma	TCGA-BT-A20N	1	1	1	1	1
Bladder Urothelial carcinoma	TCGA-BT-A20O	1	0	0	0	1
Bladder Urothelial carcinoma	TCGA-BT-A20P	1	1	1	0	1
Bladder Urothelial carcinoma	TCGA-BT-A20Q	1	1	1	0	1
Bladder Urothelial carcinoma	TCGA-BT-A20R	1	1	1	1	1
Bladder Urothelial carcinoma	TCGA-BT-A20T	1	1	1	1	1
Bladder Urothelial carcinoma	TCGA-BT-A20U	1	0	1	0	1
Bladder Urothelial carcinoma	TCGA-BT-A20V	1	0	1	1	1
Bladder Urothelial carcinoma	TCGA-BT-A20W	1	1	1	1	1
Bladder Urothelial carcinoma	TCGA-BT-A20X	0	0	1	1	1
Bladder Urothelial carcinoma	TCGA-BT-A2LA	1	1	1	0	1
Bladder Urothelial carcinoma	TCGA-BT-A2LB	1	1	1	1	1
Bladder Urothelial carcinoma	TCGA-BT-A2LD	1	1	1	1	1
Bladder Urothelial carcinoma	TCGA-BT-A3PH	1	1	1	1	1
Bladder Urothelial carcinoma	TCGA-BT-A3PJ	1	1	1	1	1
Bladder Urothelial carcinoma	TCGA-BT-A3PK	1	1	1	1	1
Bladder Urothelial carcinoma	TCGA-BT-A42C	1	1	1	1	1
Bladder Urothelial carcinoma	TCGA-BT-A42E	1	1	1	1	1
Bladder Urothelial carcinoma	TCGA-BT-A42F	1	1	1	0	1
Bladder Urothelial carcinoma	TCGA-C4-A0EZ	1	1	1	1	1
Bladder Urothelial carcinoma	TCGA-C4-A0F0	1	1	1	1	1
Bladder Urothelial carcinoma	TCGA-C4-A0F1	0	0	0	0	0
Bladder Urothelial carcinoma	TCGA-C4-A0F6	1	1	1	0	1
Bladder Urothelial carcinoma	TCGA-C4-A0F7	0	0	0	0	0
Bladder Urothelial carcinoma	TCGA-CF-A1HR	1	1	1	1	1
Bladder Urothelial carcinoma	TCGA-CF-A1HS	1	1	1	1	1
Bladder Urothelial carcinoma	TCGA-CF-A27C	1	1	1	0	1
Bladder Urothelial carcinoma	TCGA-CF-A3MF	1	1	1	0	1
Bladder Urothelial carcinoma	TCGA-CF-A3MG	1	1	1	0	1
Bladder Urothelial carcinoma	TCGA-CF-A3MH	1	1	1	0	1
Bladder Urothelial carcinoma	TCGA-CF-A3MI	1	1	1	1	1
Bladder Urothelial carcinoma	TCGA-CF-A47S	1	1	0	0	1
Bladder Urothelial carcinoma	TCGA-CF-A47T	1	1	0	0	1
Bladder Urothelial carcinoma	TCGA-CF-A47V	1	1	0	0	0
Bladder Urothelial carcinoma	TCGA-CF-A47W	0	0	0	0	0
Bladder Urothelial carcinoma	TCGA-CF-A47X	1	1	1	1	1
Bladder Urothelial carcinoma	TCGA-CF-A47Y	0	0	0	0	0
Bladder Urothelial carcinoma	TCGA-CF-A5U8	1	1	0	0	1
Bladder Urothelial carcinoma	TCGA-CF-A5UA	1	1	0	0	1
Bladder Urothelial carcinoma	TCGA-CF-A7I0	1	1	1	0	1
Bladder Urothelial carcinoma	TCGA-CF-A8HX	1	1	0	1	1
Bladder Urothelial carcinoma	TCGA-CF-A8HY	1	1	0	0	1
Bladder Urothelial carcinoma	TCGA-CF-A9FF	1	1	0	0	1
Bladder Urothelial carcinoma	TCGA-CF-A9FH	1	1	0	0	1
Bladder Urothelial carcinoma	TCGA-CF-A9FL	1	1	1	0	1
Bladder Urothelial carcinoma	TCGA-CF-A9FM	1	0	0	0	0
Bladder Urothelial carcinoma	TCGA-CU-A0YN	1	1	1	1	1
Bladder Urothelial carcinoma	TCGA-CU-A0YO	1	1	0	1	1
Bladder Urothelial carcinoma	TCGA-CU-A0YR	1	1	0	1	1
Bladder Urothelial carcinoma	TCGA-CU-A3KJ	1	1	1	1	1
Bladder Urothelial carcinoma	TCGA-CU-A3QU	1	1	1	1	1
Bladder Urothelial carcinoma	TCGA-CU-A3YL	0	1	0	0	1
Bladder Urothelial carcinoma	TCGA-CU-A5W6	1	1	1	1	1
Bladder Urothelial carcinoma	TCGA-CU-A72E	1	1	0	0	1

Bladder Urothelial carcinoma	TCGA-DK-A1A3	1	1	0	1	1
Bladder Urothelial carcinoma	TCGA-DK-A1A5	1	1	1	1	1
Bladder Urothelial carcinoma	TCGA-DK-A1A6	1	1	1	1	1
Bladder Urothelial carcinoma	TCGA-DK-A1A7	1	1	1	1	1
Bladder Urothelial carcinoma	TCGA-DK-A1AA	1	1	0	0	1
Bladder Urothelial carcinoma	TCGA-DK-A1AB	1	1	1	1	1
Bladder Urothelial carcinoma	TCGA-DK-A1AC	1	1	1	1	1
Bladder Urothelial carcinoma	TCGA-DK-A1AD	1	1	1	1	1
Bladder Urothelial carcinoma	TCGA-DK-A1AE	1	1	1	1	1
Bladder Urothelial carcinoma	TCGA-DK-A1AF	1	1	0	0	1
Bladder Urothelial carcinoma	TCGA-DK-A1AG	1	1	0	0	1
Bladder Urothelial carcinoma	TCGA-DK-A2HX	1	1	1	1	1
Bladder Urothelial carcinoma	TCGA-DK-A2I1	1	1	1	1	1
Bladder Urothelial carcinoma	TCGA-DK-A2I2	1	1	1	1	1
Bladder Urothelial carcinoma	TCGA-DK-A2I4	1	1	1	1	1
Bladder Urothelial carcinoma	TCGA-DK-A2I6	1	1	1	1	1
Bladder Urothelial carcinoma	TCGA-DK-A3IK	1	1	1	1	1
Bladder Urothelial carcinoma	TCGA-DK-A3IL	1	1	1	1	1
Bladder Urothelial carcinoma	TCGA-DK-A3IM	1	1	1	1	1
Bladder Urothelial carcinoma	TCGA-DK-A3IN	1	1	1	1	1
Bladder Urothelial carcinoma	TCGA-DK-A3IQ	1	1	1	1	1
Bladder Urothelial carcinoma	TCGA-DK-A3IS	1	1	1	0	1
Bladder Urothelial carcinoma	TCGA-DK-A3IT	1	1	1	1	1
Bladder Urothelial carcinoma	TCGA-DK-A3IU	1	1	1	0	1
Bladder Urothelial carcinoma	TCGA-DK-A3IV	1	1	1	1	1
Bladder Urothelial carcinoma	TCGA-DK-A3WW	1	1	1	0	1
Bladder Urothelial carcinoma	TCGA-DK-A3WX	1	1	1	1	1
Bladder Urothelial carcinoma	TCGA-DK-A3WY	0	0	0	0	0
Bladder Urothelial carcinoma	TCGA-DK-A3X1	1	1	1	1	1
Bladder Urothelial carcinoma	TCGA-DK-A3X2	1	1	1	1	1
Bladder Urothelial carcinoma	TCGA-DK-A6AV	1	1	1	0	1
Bladder Urothelial carcinoma	TCGA-DK-A6AW	0	0	0	0	1
Bladder Urothelial carcinoma	TCGA-DK-A6B0	0	1	0	0	0
Bladder Urothelial carcinoma	TCGA-DK-A6B1	1	1	1	1	1
Bladder Urothelial carcinoma	TCGA-DK-A6B2	1	1	1	1	1
Bladder Urothelial carcinoma	TCGA-DK-A6B5	1	1	1	1	1
Bladder Urothelial carcinoma	TCGA-DK-A6B6	1	1	1	1	1
Bladder Urothelial carcinoma	TCGA-DK-AA6L	1	1	1	1	1
Bladder Urothelial carcinoma	TCGA-DK-AA6M	1	1	0	1	1
Bladder Urothelial carcinoma	TCGA-DK-AA6P	1	1	1	1	1
Bladder Urothelial carcinoma	TCGA-DK-AA6Q	1	1	1	1	1
Bladder Urothelial carcinoma	TCGA-DK-AA6R	1	1	1	1	1
Bladder Urothelial carcinoma	TCGA-DK-AA6S	1	1	1	1	1
Bladder Urothelial carcinoma	TCGA-DK-AA6T	0	0	1	0	0
Bladder Urothelial carcinoma	TCGA-DK-AA6U	1	1	1	1	1
Bladder Urothelial carcinoma	TCGA-DK-AA6W	1	1	1	1	1
Bladder Urothelial carcinoma	TCGA-DK-AA6X	1	1	1	1	1
Bladder Urothelial carcinoma	TCGA-DK-AA71	0	1	1	0	1
Bladder Urothelial carcinoma	TCGA-DK-AA74	1	1	1	1	0
Bladder Urothelial carcinoma	TCGA-DK-AA75	1	1	1	1	1
Bladder Urothelial carcinoma	TCGA-DK-AA76	1	1	1	1	1
Bladder Urothelial carcinoma	TCGA-DK-AA77	1	1	1	0	1
Bladder Urothelial carcinoma	TCGA-E5-A2PC	1	1	1	1	1
Bladder Urothelial carcinoma	TCGA-E5-A4TZ	1	1	1	1	1
Bladder Urothelial carcinoma	TCGA-E5-A4U1	1	1	1	1	1
Bladder Urothelial carcinoma	TCGA-E7-A3X6	1	1	1	0	1
Bladder Urothelial carcinoma	TCGA-E7-A3Y1	0	0	0	0	0
Bladder Urothelial carcinoma	TCGA-E7-A4IJ	1	1	1	1	1

Bladder Urothelial carcinoma	TCGA-E7-A4XJ	1	0	0	1	0
Bladder Urothelial carcinoma	TCGA-E7-A519	1	0	0	0	0
Bladder Urothelial carcinoma	TCGA-E7-A541	1	1	1	1	1
Bladder Urothelial carcinoma	TCGA-E7-A5KE	1	1	1	1	1
Bladder Urothelial carcinoma	TCGA-E7-A5KF	1	1	0	0	1
Bladder Urothelial carcinoma	TCGA-E7-A677	1	1	1	0	1
Bladder Urothelial carcinoma	TCGA-E7-A678	0	0	0	0	0
Bladder Urothelial carcinoma	TCGA-E7-A6MD	1	1	1	1	1
Bladder Urothelial carcinoma	TCGA-E7-A6ME	1	1	1	1	1
Bladder Urothelial carcinoma	TCGA-E7-A6MF	0	0	0	0	0
Bladder Urothelial carcinoma	TCGA-E7-A7DU	1	1	1	0	1
Bladder Urothelial carcinoma	TCGA-E7-A7DV	1	1	1	1	1
Bladder Urothelial carcinoma	TCGA-E7-A7PW	1	1	1	1	1
Bladder Urothelial carcinoma	TCGA-E7-A7XN	0	0	0	0	0
Bladder Urothelial carcinoma	TCGA-E7-A85H	1	1	1	1	1
Bladder Urothelial carcinoma	TCGA-E7-A8O7	1	0	0	0	1
Bladder Urothelial carcinoma	TCGA-E7-A8O8	0	0	0	0	0
Bladder Urothelial carcinoma	TCGA-E7-A97P	1	1	1	1	1
Bladder Urothelial carcinoma	TCGA-E7-A97Q	1	1	1	1	1
Bladder Urothelial carcinoma	TCGA-FD-A3B3	1	1	0	1	1
Bladder Urothelial carcinoma	TCGA-FD-A3B4	1	1	1	1	1
Bladder Urothelial carcinoma	TCGA-FD-A3B5	1	1	1	1	1
Bladder Urothelial carcinoma	TCGA-FD-A3B6	1	1	1	1	1
Bladder Urothelial carcinoma	TCGA-FD-A3B7	1	1	0	0	1
Bladder Urothelial carcinoma	TCGA-FD-A3B8	1	0	0	0	1
Bladder Urothelial carcinoma	TCGA-FD-A3N5	1	1	1	1	1
Bladder Urothelial carcinoma	TCGA-FD-A3N6	0	0	0	1	1
Bladder Urothelial carcinoma	TCGA-FD-A3NA	1	0	1	1	1
Bladder Urothelial carcinoma	TCGA-FD-A3SJ	1	1	1	1	1
Bladder Urothelial carcinoma	TCGA-FD-A3SL	1	1	1	1	1
Bladder Urothelial carcinoma	TCGA-FD-A3SM	1	1	1	0	1
Bladder Urothelial carcinoma	TCGA-FD-A3SN	1	1	1	1	1
Bladder Urothelial carcinoma	TCGA-FD-A3SO	1	1	1	1	1
Bladder Urothelial carcinoma	TCGA-FD-A3SP	1	1	1	1	1
Bladder Urothelial carcinoma	TCGA-FD-A3SQ	1	1	1	0	1
Bladder Urothelial carcinoma	TCGA-FD-A3SR	1	1	1	1	1
Bladder Urothelial carcinoma	TCGA-FD-A3SS	1	1	1	1	1
Bladder Urothelial carcinoma	TCGA-FD-A43N	1	1	1	1	1
Bladder Urothelial carcinoma	TCGA-FD-A43P	1	1	1	1	1
Bladder Urothelial carcinoma	TCGA-FD-A43S	0	0	0	0	0
Bladder Urothelial carcinoma	TCGA-FD-A43U	1	1	1	1	1
Bladder Urothelial carcinoma	TCGA-FD-A43X	1	1	1	0	1
Bladder Urothelial carcinoma	TCGA-FD-A43Y	1	1	1	1	1
Bladder Urothelial carcinoma	TCGA-FD-A5BR	1	1	1	1	1
Bladder Urothelial carcinoma	TCGA-FD-A5BS	0	0	0	0	0
Bladder Urothelial carcinoma	TCGA-FD-A5BT	0	0	1	0	0
Bladder Urothelial carcinoma	TCGA-FD-A5BU	1	1	1	1	1
Bladder Urothelial carcinoma	TCGA-FD-A5BV	1	1	1	1	1
Bladder Urothelial carcinoma	TCGA-FD-A5BX	1	1	1	0	1
Bladder Urothelial carcinoma	TCGA-FD-A5BY	1	1	1	1	1
Bladder Urothelial carcinoma	TCGA-FD-A5BZ	1	1	1	1	1
Bladder Urothelial carcinoma	TCGA-FD-A5C0	1	1	1	1	1
Bladder Urothelial carcinoma	TCGA-FD-A5C1	1	1	1	1	1
Bladder Urothelial carcinoma	TCGA-FD-A62N	1	1	1	1	1
Bladder Urothelial carcinoma	TCGA-FD-A62O	1	1	1	1	1
Bladder Urothelial carcinoma	TCGA-FD-A62P	1	1	1	1	1
Bladder Urothelial carcinoma	TCGA-FD-A62S	1	1	1	1	1
Bladder Urothelial carcinoma	TCGA-FD-A6TA	1	1	1	0	1

Bladder Urothelial carcinoma	TCGA-FD-A6TB	1	1	1	1	1
Bladder Urothelial carcinoma	TCGA-FD-A6TC	1	1	1	1	1
Bladder Urothelial carcinoma	TCGA-FD-A6TD	1	1	1	1	1
Bladder Urothelial carcinoma	TCGA-FD-A6TE	1	1	1	1	1
Bladder Urothelial carcinoma	TCGA-FD-A6TF	1	1	1	1	1
Bladder Urothelial carcinoma	TCGA-FD-A6TG	1	1	1	1	1
Bladder Urothelial carcinoma	TCGA-FD-A6TH	1	1	1	1	1
Bladder Urothelial carcinoma	TCGA-FD-A6TI	0	1	0	0	0
Bladder Urothelial carcinoma	TCGA-FD-A6TK	1	1	1	1	1
Bladder Urothelial carcinoma	TCGA-FJ-A3Z7	1	1	1	1	1
Bladder Urothelial carcinoma	TCGA-FJ-A3Z9	0	0	0	0	0
Bladder Urothelial carcinoma	TCGA-FJ-A3ZE	1	1	1	1	1
Bladder Urothelial carcinoma	TCGA-FJ-A3ZF	1	1	1	1	1
Bladder Urothelial carcinoma	TCGA-FJ-A871	1	1	1	0	1
Bladder Urothelial carcinoma	TCGA-FT-A3EE	0	0	0	0	1
Bladder Urothelial carcinoma	TCGA-FT-A61P	1	1	1	1	1
Bladder Urothelial carcinoma	TCGA-G2-A2EC	0	0	0	0	0
Bladder Urothelial carcinoma	TCGA-G2-A2EF	1	1	1	1	1
Bladder Urothelial carcinoma	TCGA-G2-A2EJ	1	0	1	1	1
Bladder Urothelial carcinoma	TCGA-G2-A2EK	1	1	0	0	1
Bladder Urothelial carcinoma	TCGA-G2-A2EL	1	1	1	1	1
Bladder Urothelial carcinoma	TCGA-G2-A2EO	1	1	1	1	1
Bladder Urothelial carcinoma	TCGA-G2-A2ES	1	1	1	1	1
Bladder Urothelial carcinoma	TCGA-G2-A3IB	1	1	1	1	1
Bladder Urothelial carcinoma	TCGA-G2-A3IE	1	1	1	1	1
Bladder Urothelial carcinoma	TCGA-G2-A3VY	1	1	1	1	1
Bladder Urothelial carcinoma	TCGA-G2-AA3B	1	1	1	1	1
Bladder Urothelial carcinoma	TCGA-G2-AA3C	1	1	0	0	1
Bladder Urothelial carcinoma	TCGA-G2-AA3D	1	1	1	1	1
Bladder Urothelial carcinoma	TCGA-G2-AA3F	1	1	1	1	1
Bladder Urothelial carcinoma	TCGA-GC-A3BM	1	1	1	1	1
Bladder Urothelial carcinoma	TCGA-GC-A3I6	1	1	1	1	1
Bladder Urothelial carcinoma	TCGA-GC-A3OO	1	0	1	1	1
Bladder Urothelial carcinoma	TCGA-GC-A3RB	1	1	1	1	1
Bladder Urothelial carcinoma	TCGA-GC-A3RC	1	1	0	1	1
Bladder Urothelial carcinoma	TCGA-GC-A3RD	1	1	1	1	1
Bladder Urothelial carcinoma	TCGA-GC-A3WC	1	1	0	1	1
Bladder Urothelial carcinoma	TCGA-GC-A3YS	1	1	1	1	1
Bladder Urothelial carcinoma	TCGA-GC-A4ZW	1	1	1	1	1
Bladder Urothelial carcinoma	TCGA-GC-A6I1	1	1	1	1	1
Bladder Urothelial carcinoma	TCGA-GC-A6I3	1	1	1	1	1
Bladder Urothelial carcinoma	TCGA-GD-A2C5	1	1	1	1	1
Bladder Urothelial carcinoma	TCGA-GD-A3OP	1	1	1	1	1
Bladder Urothelial carcinoma	TCGA-GD-A3OQ	0	1	1	1	1
Bladder Urothelial carcinoma	TCGA-GD-A3OS	1	1	1	0	1
Bladder Urothelial carcinoma	TCGA-GD-A6C6	1	1	1	1	1
Bladder Urothelial carcinoma	TCGA-GD-A76B	1	1	1	1	1
Bladder Urothelial carcinoma	TCGA-GU-A42P	1	1	1	1	1
Bladder Urothelial carcinoma	TCGA-GU-A42Q	1	1	1	1	1
Bladder Urothelial carcinoma	TCGA-GU-A42R	1	1	1	1	1
Bladder Urothelial carcinoma	TCGA-GU-A762	1	1	1	1	1
Bladder Urothelial carcinoma	TCGA-GU-A763	1	1	0	1	1
Bladder Urothelial carcinoma	TCGA-GU-A764	1	1	1	1	1
Bladder Urothelial carcinoma	TCGA-GU-A766	1	1	0	1	1
Bladder Urothelial carcinoma	TCGA-GU-A767	1	1	1	1	1
Bladder Urothelial carcinoma	TCGA-GU-AATO	1	1	1	1	1
Bladder Urothelial carcinoma	TCGA-GU-AATP	1	1	1	1	1
Bladder Urothelial carcinoma	TCGA-GU-AATQ	1	1	1	1	1

Bladder Urothelial carcinoma	TCGA-GV-A3JV	1	1	1	1	1
Bladder Urothelial carcinoma	TCGA-GV-A3JW	1	1	1	1	1
Bladder Urothelial carcinoma	TCGA-GV-A3JX	1	1	1	1	1
Bladder Urothelial carcinoma	TCGA-GV-A3JZ	1	1	1	1	1
Bladder Urothelial carcinoma	TCGA-GV-A3QF	1	1	1	1	1
Bladder Urothelial carcinoma	TCGA-GV-A3QG	1	1	1	1	1
Bladder Urothelial carcinoma	TCGA-GV-A3QH	1	1	1	1	1
Bladder Urothelial carcinoma	TCGA-GV-A3QI	1	1	1	1	1
Bladder Urothelial carcinoma	TCGA-GV-A3QK	1	1	1	0	1
Bladder Urothelial carcinoma	TCGA-GV-A40E	1	0	1	1	1
Bladder Urothelial carcinoma	TCGA-GV-A40G	1	1	1	1	1
Bladder Urothelial carcinoma	TCGA-GV-A6ZA	1	1	1	1	1
Bladder Urothelial carcinoma	TCGA-H4-A2HO	1	1	1	1	1
Bladder Urothelial carcinoma	TCGA-H4-A2HQ	1	1	1	1	1
Bladder Urothelial carcinoma	TCGA-HQ-A2OE	1	1	1	1	1
Bladder Urothelial carcinoma	TCGA-HQ-A2OF	1	1	1	1	1
Bladder Urothelial carcinoma	TCGA-HQ-A5ND	1	1	1	1	1
Bladder Urothelial carcinoma	TCGA-HQ-A5NE	1	1	1	1	1
Bladder Urothelial carcinoma	TCGA-K4-A3WS	1	1	1	1	1
Bladder Urothelial carcinoma	TCGA-K4-A3WU	1	1	1	1	1
Bladder Urothelial carcinoma	TCGA-K4-A3WV	1	1	1	1	1
Bladder Urothelial carcinoma	TCGA-K4-A4AB	1	1	1	1	1
Bladder Urothelial carcinoma	TCGA-K4-A4AC	1	1	1	1	1
Bladder Urothelial carcinoma	TCGA-K4-A54R	1	1	1	1	1
Bladder Urothelial carcinoma	TCGA-K4-A5RH	1	1	1	1	1
Bladder Urothelial carcinoma	TCGA-K4-A5RI	1	1	1	1	1
Bladder Urothelial carcinoma	TCGA-K4-A5RJ	1	1	1	1	1
Bladder Urothelial carcinoma	TCGA-K4-A6FZ	1	1	1	1	1
Bladder Urothelial carcinoma	TCGA-K4-A6MB	1	1	1	1	1
Bladder Urothelial carcinoma	TCGA-K4-A83P	0	1	0	0	0
Bladder Urothelial carcinoma	TCGA-K4-AAQO	1	1	1	0	1
Bladder Urothelial carcinoma	TCGA-KQ-A41N	1	0	1	0	1
Bladder Urothelial carcinoma	TCGA-KQ-A41O	1	1	1	1	1
Bladder Urothelial carcinoma	TCGA-KQ-A41P	1	1	1	1	1
Bladder Urothelial carcinoma	TCGA-KQ-A41Q	1	1	1	1	1
Bladder Urothelial carcinoma	TCGA-KQ-A41R	1	1	1	1	1
Bladder Urothelial carcinoma	TCGA-KQ-A41S	1	1	1	1	1
Bladder Urothelial carcinoma	TCGA-LC-A66R	1	1	1	1	1
Bladder Urothelial carcinoma	TCGA-LT-A5Z6	1	1	1	1	1
Bladder Urothelial carcinoma	TCGA-LT-A8JT	1	1	0	0	1
Bladder Urothelial carcinoma	TCGA-MV-A51V	1	1	1	1	1
Bladder Urothelial carcinoma	TCGA-PQ-A6FI	1	1	1	1	1
Bladder Urothelial carcinoma	TCGA-PQ-A6FN	1	1	1	1	1
Bladder Urothelial carcinoma	TCGA-R3-A69X	1	1	1	1	1
Bladder Urothelial carcinoma	TCGA-S5-A6DX	1	1	1	0	1
Bladder Urothelial carcinoma	TCGA-S5-AA26	1	1	1	0	1
Bladder Urothelial carcinoma	TCGA-SY-A9G0	1	1	1	1	1
Bladder Urothelial carcinoma	TCGA-SY-A9G5	1	1	1	1	1
Bladder Urothelial carcinoma	TCGA-UY-A78K	1	1	1	1	1
Bladder Urothelial carcinoma	TCGA-UY-A78L	1	1	1	1	1
Bladder Urothelial carcinoma	TCGA-UY-A78M	1	1	1	1	1
Bladder Urothelial carcinoma	TCGA-UY-A78N	1	1	1	1	1
Bladder Urothelial carcinoma	TCGA-UY-A78O	1	1	1	1	1
Bladder Urothelial carcinoma	TCGA-UY-A78P	1	0	1	0	1
Bladder Urothelial carcinoma	TCGA-UY-A8OB	1	1	1	1	1
Bladder Urothelial carcinoma	TCGA-UY-A8OC	0	1	1	0	1
Bladder Urothelial carcinoma	TCGA-UY-A8OD	1	1	1	1	1
Bladder Urothelial carcinoma	TCGA-UY-A9PA	1	1	1	1	1

Bladder Urothelial carcinoma	TCGA-UY-A9PB	1	1	1	1	1
Bladder Urothelial carcinoma	TCGA-UY-A9PD	1	1	1	1	1
Bladder Urothelial carcinoma	TCGA-UY-A9PE	1	1	1	1	1
Bladder Urothelial carcinoma	TCGA-UY-A9PF	1	1	1	1	1
Bladder Urothelial carcinoma	TCGA-UY-A9PH	1	0	1	0	1
Bladder Urothelial carcinoma	TCGA-XF-A8HB	1	0	1	1	1
Bladder Urothelial carcinoma	TCGA-XF-A8HC	1	1	1	1	1
Bladder Urothelial carcinoma	TCGA-XF-A8HD	1	0	1	1	1
Bladder Urothelial carcinoma	TCGA-XF-A8HE	1	0	1	0	1
Bladder Urothelial carcinoma	TCGA-XF-A8HF	1	1	1	1	1
Bladder Urothelial carcinoma	TCGA-XF-A8HG	1	1	1	1	1
Bladder Urothelial carcinoma	TCGA-XF-A8HH	1	1	1	1	1
Bladder Urothelial carcinoma	TCGA-XF-A8HI	1	1	1	1	1
Bladder Urothelial carcinoma	TCGA-XF-A9SH	1	1	1	0	0
Bladder Urothelial carcinoma	TCGA-XF-A9SI	1	1	1	1	1
Bladder Urothelial carcinoma	TCGA-XF-A9SJ	1	1	1	1	1
Bladder Urothelial carcinoma	TCGA-XF-A9SK	1	1	1	1	1
Bladder Urothelial carcinoma	TCGA-XF-A9SL	0	0	0	0	0
Bladder Urothelial carcinoma	TCGA-XF-A9SM	1	1	1	1	1
Bladder Urothelial carcinoma	TCGA-XF-A9SP	1	1	1	1	1
Bladder Urothelial carcinoma	TCGA-XF-A9ST	1	1	1	1	1
Bladder Urothelial carcinoma	TCGA-XF-A9SU	1	1	1	1	1
Bladder Urothelial carcinoma	TCGA-XF-A9SV	1	1	1	1	1
Bladder Urothelial carcinoma	TCGA-XF-A9SW	1	1	1	1	1
Bladder Urothelial carcinoma	TCGA-XF-A9SX	1	1	1	1	1
Bladder Urothelial carcinoma	TCGA-XF-A9SY	1	1	0	1	1
Bladder Urothelial carcinoma	TCGA-XF-A9SZ	1	1	1	1	1
Bladder Urothelial carcinoma	TCGA-XF-A9T0	1	1	1	1	1
Bladder Urothelial carcinoma	TCGA-XF-A9T2	0	0	0	0	0
Bladder Urothelial carcinoma	TCGA-XF-A9T3	1	1	1	1	1
Bladder Urothelial carcinoma	TCGA-XF-A9T4	0	1	0	0	1
Bladder Urothelial carcinoma	TCGA-XF-A9T5	1	1	1	1	1
Bladder Urothelial carcinoma	TCGA-XF-A9T6	1	1	1	1	1
Bladder Urothelial carcinoma	TCGA-XF-A9T8	1	0	0	0	1
Bladder Urothelial carcinoma	TCGA-XF-AAME	0	0	0	0	0
Bladder Urothelial carcinoma	TCGA-XF-AAMG	1	1	1	1	1
Bladder Urothelial carcinoma	TCGA-XF-AAMH	0	0	0	0	0
Bladder Urothelial carcinoma	TCGA-XF-AAMJ	1	0	1	0	1
Bladder Urothelial carcinoma	TCGA-XF-AAML	1	1	1	1	1
Bladder Urothelial carcinoma	TCGA-XF-AAMQ	1	1	1	0	1
Bladder Urothelial carcinoma	TCGA-XF-AAMR	1	1	1	0	1
Bladder Urothelial carcinoma	TCGA-XF-AAMT	1	1	1	1	1
Bladder Urothelial carcinoma	TCGA-XF-AAMW	1	1	1	1	1
Bladder Urothelial carcinoma	TCGA-XF-AAMX	1	1	1	1	1
Bladder Urothelial carcinoma	TCGA-XF-AAMY	1	1	1	1	1
Bladder Urothelial carcinoma	TCGA-XF-AAMZ	1	1	1	1	1
Bladder Urothelial carcinoma	TCGA-XF-AAN0	1	1	1	1	1
Bladder Urothelial carcinoma	TCGA-XF-AAN1	1	1	1	1	1
Bladder Urothelial carcinoma	TCGA-XF-AAN2	1	1	0	1	1
Bladder Urothelial carcinoma	TCGA-XF-AAN3	1	1	1	1	1
Bladder Urothelial carcinoma	TCGA-XF-AAN4	0	1	0	0	1
Bladder Urothelial carcinoma	TCGA-XF-AAN5	0	1	1	1	1
Bladder Urothelial carcinoma	TCGA-XF-AAN7	1	1	1	1	1
Bladder Urothelial carcinoma	TCGA-XF-AAN8	0	0	0	0	0
Bladder Urothelial carcinoma	TCGA-YC-A89H	0	1	0	0	0
Bladder Urothelial carcinoma	TCGA-YC-A8S6	0	0	0	0	0
Bladder Urothelial carcinoma	TCGA-YC-A9TC	0	0	0	0	1
Bladder Urothelial carcinoma	TCGA-YF-AA3L	1	1	1	1	1

Bladder Urothelial carcinoma	TCGA-YF-AA3M	1	1	1	1	1
Bladder Urothelial carcinoma	TCGA-ZF-A9R0	1	1	1	1	1
Bladder Urothelial carcinoma	TCGA-ZF-A9R1	1	1	1	1	1
Bladder Urothelial carcinoma	TCGA-ZF-A9R2	1	1	1	1	1
Bladder Urothelial carcinoma	TCGA-ZF-A9R3	1	1	1	1	1
Bladder Urothelial carcinoma	TCGA-ZF-A9R4	1	1	1	1	1
Bladder Urothelial carcinoma	TCGA-ZF-A9R5	0	0	0	0	0
Bladder Urothelial carcinoma	TCGA-ZF-A9R7	1	1	1	1	1
Bladder Urothelial carcinoma	TCGA-ZF-A9R9	1	1	1	1	1
Bladder Urothelial carcinoma	TCGA-ZF-A9RC	1	1	1	1	1
Bladder Urothelial carcinoma	TCGA-ZF-A9RD	1	1	1	1	1
Bladder Urothelial carcinoma	TCGA-ZF-A9RE	1	1	1	1	1
Bladder Urothelial carcinoma	TCGA-ZF-A9RF	1	1	1	1	1
Bladder Urothelial carcinoma	TCGA-ZF-A9RL	1	1	1	1	1
Bladder Urothelial carcinoma	TCGA-ZF-A9RM	1	1	1	1	1
Bladder Urothelial carcinoma	TCGA-ZF-A9RN	1	1	1	1	1
Bladder Urothelial carcinoma	TCGA-ZF-AA4N	1	1	1	1	1
Bladder Urothelial carcinoma	TCGA-ZF-AA4R	1	0	1	0	1
Bladder Urothelial carcinoma	TCGA-ZF-AA4T	1	1	1	1	1
Bladder Urothelial carcinoma	TCGA-ZF-AA4U	1	1	1	1	1
Bladder Urothelial carcinoma	TCGA-ZF-AA4V	1	1	1	1	1
Bladder Urothelial carcinoma	TCGA-ZF-AA4W	1	1	1	1	1
Bladder Urothelial carcinoma	TCGA-ZF-AA4X	1	1	1	1	1
Bladder Urothelial carcinoma	TCGA-ZF-AA51	1	1	1	1	1
Bladder Urothelial carcinoma	TCGA-ZF-AA52	1	1	1	1	1
Bladder Urothelial carcinoma	TCGA-ZF-AA53	1	1	1	1	1
Bladder Urothelial carcinoma	TCGA-ZF-AA54	1	1	1	1	1
Bladder Urothelial carcinoma	TCGA-ZF-AA56	1	1	1	1	1
Bladder Urothelial carcinoma	TCGA-ZF-AA58	1	1	1	1	1
Bladder Urothelial carcinoma	TCGA-ZF-AA5H	1	1	1	1	1
Bladder Urothelial carcinoma	TCGA-ZF-AA5N	0	0	0	0	0
Bladder Urothelial carcinoma	TCGA-ZF-AA5P	0	1	0	1	1
Breast invasive carcinoma	TCGA-3C-AAAU	1	1	1	1	1
Breast invasive carcinoma	TCGA-3C-AALI	1	1	1	1	1
Breast invasive carcinoma	TCGA-3C-AALJ	1	1	1	1	1
Breast invasive carcinoma	TCGA-3C-AALK	0	1	1	0	1
Breast invasive carcinoma	TCGA-4H-AAAK	1	0	1	0	1
Breast invasive carcinoma	TCGA-5L-AAT0	1	0	1	0	1
Breast invasive carcinoma	TCGA-5L-AAT1	0	0	0	0	0
Breast invasive carcinoma	TCGA-5T-A9QA	1	1	1	1	1
Breast invasive carcinoma	TCGA-A1-A0SB	0	0	0	0	0
Breast invasive carcinoma	TCGA-A1-A0SD	1	1	1	1	1
Breast invasive carcinoma	TCGA-A1-A0SE	0	0	1	0	1
Breast invasive carcinoma	TCGA-A1-A0SF	0	1	1	1	1
Breast invasive carcinoma	TCGA-A1-A0SG	0	0	1	1	1
Breast invasive carcinoma	TCGA-A1-A0SH	1	1	1	1	1
Breast invasive carcinoma	TCGA-A1-A0SI	1	1	1	1	1
Breast invasive carcinoma	TCGA-A1-A0SJ	1	1	1	1	1
Breast invasive carcinoma	TCGA-A1-A0SK	1	1	1	1	1
Breast invasive carcinoma	TCGA-A1-A0SM	1	1	1	1	1
Breast invasive carcinoma	TCGA-A1-A0SN	1	1	1	1	1
Breast invasive carcinoma	TCGA-A1-A0SO	1	1	1	1	1
Breast invasive carcinoma	TCGA-A1-A0SP	1	1	1	1	1
Breast invasive carcinoma	TCGA-A1-A0SQ	1	0	1	0	1
Breast invasive carcinoma	TCGA-A2-A04N	1	0	0	1	1
Breast invasive carcinoma	TCGA-A2-A04P	1	1	1	1	1
Breast invasive carcinoma	TCGA-A2-A04Q	1	1	1	1	1
Breast invasive carcinoma	TCGA-A2-A04R	1	1	1	1	1

Breast invasive carcinoma	TCGA-A2-A04T	1	1	1	1	1
Breast invasive carcinoma	TCGA-A2-A04U	1	1	1	1	1
Breast invasive carcinoma	TCGA-A2-A04V	1	1	1	1	1
Breast invasive carcinoma	TCGA-A2-A04W	1	1	1	1	1
Breast invasive carcinoma	TCGA-A2-A04X	1	1	1	1	1
Breast invasive carcinoma	TCGA-A2-A04Y	1	1	1	1	1
Breast invasive carcinoma	TCGA-A2-A0CK	0	0	1	0	1
Breast invasive carcinoma	TCGA-A2-A0CL	1	1	0	1	1
Breast invasive carcinoma	TCGA-A2-A0CM	1	1	1	1	1
Breast invasive carcinoma	TCGA-A2-A0CO	0	1	1	1	1
Breast invasive carcinoma	TCGA-A2-A0CP	1	1	0	1	1
Breast invasive carcinoma	TCGA-A2-A0CQ	0	0	1	1	1
Breast invasive carcinoma	TCGA-A2-A0CR	1	1	1	1	1
Breast invasive carcinoma	TCGA-A2-A0CS	1	1	1	1	1
Breast invasive carcinoma	TCGA-A2-A0CT	1	1	1	1	1
Breast invasive carcinoma	TCGA-A2-A0CU	1	1	1	1	1
Breast invasive carcinoma	TCGA-A2-A0CV	1	1	1	1	1
Breast invasive carcinoma	TCGA-A2-A0CW	1	1	1	1	1
Breast invasive carcinoma	TCGA-A2-A0CX	1	1	1	1	1
Breast invasive carcinoma	TCGA-A2-A0CY	0	0	0	0	0
Breast invasive carcinoma	TCGA-A2-A0CZ	0	0	0	0	0
Breast invasive carcinoma	TCGA-A2-A0D0	1	1	1	1	1
Breast invasive carcinoma	TCGA-A2-A0D1	1	1	1	1	1
Breast invasive carcinoma	TCGA-A2-A0D2	1	0	0	1	1
Breast invasive carcinoma	TCGA-A2-A0D3	1	0	0	0	0
Breast invasive carcinoma	TCGA-A2-A0D4	1	1	1	1	1
Breast invasive carcinoma	TCGA-A2-A0EM	1	0	0	0	1
Breast invasive carcinoma	TCGA-A2-A0EN	1	1	1	1	1
Breast invasive carcinoma	TCGA-A2-A0EO	1	1	1	1	1
Breast invasive carcinoma	TCGA-A2-A0EP	1	0	0	0	0
Breast invasive carcinoma	TCGA-A2-A0EQ	1	1	1	1	1
Breast invasive carcinoma	TCGA-A2-A0ER	0	0	0	0	1
Breast invasive carcinoma	TCGA-A2-A0ES	0	1	0	0	0
Breast invasive carcinoma	TCGA-A2-A0ET	1	1	1	1	1
Breast invasive carcinoma	TCGA-A2-A0EU	1	1	1	1	1
Breast invasive carcinoma	TCGA-A2-A0EV	1	0	1	1	1
Breast invasive carcinoma	TCGA-A2-A0EW	1	1	0	1	1
Breast invasive carcinoma	TCGA-A2-A0EX	1	1	1	1	1
Breast invasive carcinoma	TCGA-A2-A0EY	1	1	1	1	1
Breast invasive carcinoma	TCGA-A2-A0ST	1	0	0	0	1
Breast invasive carcinoma	TCGA-A2-A0SU	0	0	1	1	1
Breast invasive carcinoma	TCGA-A2-A0SV	1	1	1	1	1
Breast invasive carcinoma	TCGA-A2-A0SW	1	1	1	1	1
Breast invasive carcinoma	TCGA-A2-A0SX	1	1	1	1	1
Breast invasive carcinoma	TCGA-A2-A0SY	1	0	1	0	1
Breast invasive carcinoma	TCGA-A2-A0T0	1	1	1	1	1
Breast invasive carcinoma	TCGA-A2-A0T1	1	1	1	0	1
Breast invasive carcinoma	TCGA-A2-A0T2	1	0	0	0	1
Breast invasive carcinoma	TCGA-A2-A0T3	1	1	1	1	1
Breast invasive carcinoma	TCGA-A2-A0T4	1	1	1	1	1
Breast invasive carcinoma	TCGA-A2-A0T5	1	1	0	1	0
Breast invasive carcinoma	TCGA-A2-A0T6	0	0	1	1	1
Breast invasive carcinoma	TCGA-A2-A0T7	1	1	1	1	1
Breast invasive carcinoma	TCGA-A2-A0YC	1	0	1	0	1
Breast invasive carcinoma	TCGA-A2-A0YD	0	0	0	1	1
Breast invasive carcinoma	TCGA-A2-A0YE	1	1	1	1	1
Breast invasive carcinoma	TCGA-A2-A0YF	1	1	1	1	1
Breast invasive carcinoma	TCGA-A2-A0YG	1	1	1	1	1

Breast invasive carcinoma	TCGA-A2-A0YH	1	1	1	1	1
Breast invasive carcinoma	TCGA-A2-A0YI	1	1	0	1	1
Breast invasive carcinoma	TCGA-A2-A0YJ	1	1	1	1	1
Breast invasive carcinoma	TCGA-A2-A0YK	1	1	1	0	1
Breast invasive carcinoma	TCGA-A2-A0YL	1	1	1	0	1
Breast invasive carcinoma	TCGA-A2-A0YM	1	1	1	1	1
Breast invasive carcinoma	TCGA-A2-A0YT	1	1	1	1	1
Breast invasive carcinoma	TCGA-A2-A1FV	1	1	1	0	1
Breast invasive carcinoma	TCGA-A2-A1FW	1	1	1	1	1
Breast invasive carcinoma	TCGA-A2-A1FX	1	1	1	1	1
Breast invasive carcinoma	TCGA-A2-A1FZ	1	0	0	0	0
Breast invasive carcinoma	TCGA-A2-A1G0	1	0	1	0	1
Breast invasive carcinoma	TCGA-A2-A1G1	1	1	1	1	1
Breast invasive carcinoma	TCGA-A2-A1G4	0	0	1	0	1
Breast invasive carcinoma	TCGA-A2-A1G6	0	0	0	0	0
Breast invasive carcinoma	TCGA-A2-A259	1	1	1	0	0
Breast invasive carcinoma	TCGA-A2-A25A	1	1	1	1	1
Breast invasive carcinoma	TCGA-A2-A25B	1	1	1	1	1
Breast invasive carcinoma	TCGA-A2-A25C	1	1	1	1	1
Breast invasive carcinoma	TCGA-A2-A25D	1	0	1	0	1
Breast invasive carcinoma	TCGA-A2-A25E	1	1	1	1	1
Breast invasive carcinoma	TCGA-A2-A25F	0	0	0	1	0
Breast invasive carcinoma	TCGA-A2-A3KC	1	1	0	1	1
Breast invasive carcinoma	TCGA-A2-A3KD	0	0	1	0	1
Breast invasive carcinoma	TCGA-A2-A3XS	1	1	1	1	1
Breast invasive carcinoma	TCGA-A2-A3XT	1	1	1	1	1
Breast invasive carcinoma	TCGA-A2-A3XU	1	1	0	1	1
Breast invasive carcinoma	TCGA-A2-A3XV	0	1	1	0	1
Breast invasive carcinoma	TCGA-A2-A3XW	1	0	1	0	1
Breast invasive carcinoma	TCGA-A2-A3XX	1	1	1	1	1
Breast invasive carcinoma	TCGA-A2-A3XY	1	1	1	0	1
Breast invasive carcinoma	TCGA-A2-A3XZ	1	1	1	1	1
Breast invasive carcinoma	TCGA-A2-A3Y0	1	1	1	1	1
Breast invasive carcinoma	TCGA-A2-A4RW	1	1	1	1	1
Breast invasive carcinoma	TCGA-A2-A4RX	1	1	1	1	1
Breast invasive carcinoma	TCGA-A2-A4RY	1	1	1	1	1
Breast invasive carcinoma	TCGA-A2-A4S0	0	0	0	0	0
Breast invasive carcinoma	TCGA-A2-A4S1	1	1	1	1	1
Breast invasive carcinoma	TCGA-A2-A4S2	1	0	1	1	1
Breast invasive carcinoma	TCGA-A2-A4S3	1	1	1	1	1
Breast invasive carcinoma	TCGA-A7-A0CD	0	1	1	1	1
Breast invasive carcinoma	TCGA-A7-A0CE	1	1	1	1	1
Breast invasive carcinoma	TCGA-A7-A0CG	1	1	1	1	1
Breast invasive carcinoma	TCGA-A7-A0CH	1	0	0	1	1
Breast invasive carcinoma	TCGA-A7-A0CJ	1	1	1	1	1
Breast invasive carcinoma	TCGA-A7-A0D9	1	1	0	0	1
Breast invasive carcinoma	TCGA-A7-A0DA	1	1	1	1	1
Breast invasive carcinoma	TCGA-A7-A0DB	1	0	0	1	1
Breast invasive carcinoma	TCGA-A7-A13D	1	1	1	1	1
Breast invasive carcinoma	TCGA-A7-A13E	1	1	1	1	1
Breast invasive carcinoma	TCGA-A7-A13F	1	1	1	1	1
Breast invasive carcinoma	TCGA-A7-A13G	0	0	0	1	1
Breast invasive carcinoma	TCGA-A7-A13H	1	1	1	1	1
Breast invasive carcinoma	TCGA-A7-A26E	1	1	1	1	1
Breast invasive carcinoma	TCGA-A7-A26F	1	1	1	1	1
Breast invasive carcinoma	TCGA-A7-A26G	1	1	1	1	1
Breast invasive carcinoma	TCGA-A7-A26H	1	1	1	0	1
Breast invasive carcinoma	TCGA-A7-A26I	1	1	1	1	1

Breast invasive carcinoma	TCGA-A7-A26J	0	1	1	1	1
Breast invasive carcinoma	TCGA-A7-A2KD	1	1	1	1	1
Breast invasive carcinoma	TCGA-A7-A3IY	0	0	1	0	0
Breast invasive carcinoma	TCGA-A7-A3IZ	0	0	1	1	1
Breast invasive carcinoma	TCGA-A7-A3J0	0	0	0	0	0
Breast invasive carcinoma	TCGA-A7-A3J1	1	1	1	1	1
Breast invasive carcinoma	TCGA-A7-A3RF	0	0	0	0	1
Breast invasive carcinoma	TCGA-A7-A425	1	1	0	0	1
Breast invasive carcinoma	TCGA-A7-A426	0	0	1	1	1
Breast invasive carcinoma	TCGA-A7-A4SA	1	1	1	1	1
Breast invasive carcinoma	TCGA-A7-A4SB	1	1	1	0	1
Breast invasive carcinoma	TCGA-A7-A4SC	0	0	0	0	0
Breast invasive carcinoma	TCGA-A7-A4SD	1	1	1	1	1
Breast invasive carcinoma	TCGA-A7-A4SE	1	1	1	1	1
Breast invasive carcinoma	TCGA-A7-A4SF	1	1	1	1	1
Breast invasive carcinoma	TCGA-A7-A56D	1	1	1	1	1
Breast invasive carcinoma	TCGA-A7-A5ZV	1	1	1	1	1
Breast invasive carcinoma	TCGA-A7-A5ZW	1	0	0	0	0
Breast invasive carcinoma	TCGA-A7-A5ZX	1	0	1	0	1
Breast invasive carcinoma	TCGA-A7-A6VV	1	1	1	1	1
Breast invasive carcinoma	TCGA-A7-A6VW	1	1	1	1	1
Breast invasive carcinoma	TCGA-A7-A6VX	1	1	1	1	1
Breast invasive carcinoma	TCGA-A7-A6VY	1	1	1	1	1
Breast invasive carcinoma	TCGA-A8-A06N	1	0	1	1	1
Breast invasive carcinoma	TCGA-A8-A06O	1	1	1	1	1
Breast invasive carcinoma	TCGA-A8-A06P	1	0	0	1	1
Breast invasive carcinoma	TCGA-A8-A06Q	1	1	1	1	1
Breast invasive carcinoma	TCGA-A8-A06R	1	1	1	1	1
Breast invasive carcinoma	TCGA-A8-A06T	1	1	1	1	1
Breast invasive carcinoma	TCGA-A8-A06U	1	1	1	1	1
Breast invasive carcinoma	TCGA-A8-A06X	1	1	1	1	1
Breast invasive carcinoma	TCGA-A8-A06Y	1	1	1	1	1
Breast invasive carcinoma	TCGA-A8-A06Z	1	1	1	1	1
Breast invasive carcinoma	TCGA-A8-A075	1	1	1	1	1
Breast invasive carcinoma	TCGA-A8-A076	1	1	1	1	1
Breast invasive carcinoma	TCGA-A8-A079	1	1	1	1	1
Breast invasive carcinoma	TCGA-A8-A07B	1	1	1	1	1
Breast invasive carcinoma	TCGA-A8-A07C	0	0	0	0	0
Breast invasive carcinoma	TCGA-A8-A07E	1	1	1	1	1
Breast invasive carcinoma	TCGA-A8-A07F	1	1	1	1	1
Breast invasive carcinoma	TCGA-A8-A07G	1	1	1	1	1
Breast invasive carcinoma	TCGA-A8-A07I	1	1	1	1	1
Breast invasive carcinoma	TCGA-A8-A07J	0	0	1	1	1
Breast invasive carcinoma	TCGA-A8-A07L	1	1	1	1	1
Breast invasive carcinoma	TCGA-A8-A07O	1	1	1	1	1
Breast invasive carcinoma	TCGA-A8-A07P	1	1	1	1	1
Breast invasive carcinoma	TCGA-A8-A07R	1	1	1	1	1
Breast invasive carcinoma	TCGA-A8-A07S	0	1	1	0	0
Breast invasive carcinoma	TCGA-A8-A07U	1	1	1	0	1
Breast invasive carcinoma	TCGA-A8-A07W	1	1	1	1	1
Breast invasive carcinoma	TCGA-A8-A07Z	1	0	1	0	0
Breast invasive carcinoma	TCGA-A8-A081	1	1	1	1	1
Breast invasive carcinoma	TCGA-A8-A082	0	0	1	0	1
Breast invasive carcinoma	TCGA-A8-A083	0	0	0	1	1
Breast invasive carcinoma	TCGA-A8-A084	1	1	1	1	1
Breast invasive carcinoma	TCGA-A8-A085	1	1	1	1	1
Breast invasive carcinoma	TCGA-A8-A086	0	0	1	0	1
Breast invasive carcinoma	TCGA-A8-A08A	0	0	0	0	1

Breast invasive carcinoma	TCGA-A8-A08B	1	1	1	1	1
Breast invasive carcinoma	TCGA-A8-A08C	0	0	0	0	0
Breast invasive carcinoma	TCGA-A8-A08F	1	1	1	1	1
Breast invasive carcinoma	TCGA-A8-A08G	1	1	1	1	1
Breast invasive carcinoma	TCGA-A8-A08H	1	0	0	0	0
Breast invasive carcinoma	TCGA-A8-A08I	1	1	1	1	1
Breast invasive carcinoma	TCGA-A8-A08J	1	1	1	1	1
Breast invasive carcinoma	TCGA-A8-A08L	1	1	1	1	1
Breast invasive carcinoma	TCGA-A8-A08O	1	1	1	1	1
Breast invasive carcinoma	TCGA-A8-A08P	1	1	0	1	1
Breast invasive carcinoma	TCGA-A8-A08R	1	1	1	1	1
Breast invasive carcinoma	TCGA-A8-A08S	1	1	1	1	1
Breast invasive carcinoma	TCGA-A8-A08T	1	0	1	0	1
Breast invasive carcinoma	TCGA-A8-A08X	1	1	1	1	1
Breast invasive carcinoma	TCGA-A8-A08Z	1	0	1	1	1
Breast invasive carcinoma	TCGA-A8-A090	1	1	1	1	1
Breast invasive carcinoma	TCGA-A8-A091	0	1	0	0	1
Breast invasive carcinoma	TCGA-A8-A092	1	1	1	1	1
Breast invasive carcinoma	TCGA-A8-A093	1	0	1	1	1
Breast invasive carcinoma	TCGA-A8-A094	1	1	1	1	1
Breast invasive carcinoma	TCGA-A8-A095	1	0	0	0	1
Breast invasive carcinoma	TCGA-A8-A096	1	0	0	1	1
Breast invasive carcinoma	TCGA-A8-A097	1	1	1	1	1
Breast invasive carcinoma	TCGA-A8-A099	1	1	0	1	1
Breast invasive carcinoma	TCGA-A8-A09A	1	1	1	1	1
Breast invasive carcinoma	TCGA-A8-A09B	1	1	1	1	1
Breast invasive carcinoma	TCGA-A8-A09C	1	1	1	1	1
Breast invasive carcinoma	TCGA-A8-A09D	1	1	1	1	1
Breast invasive carcinoma	TCGA-A8-A09E	1	1	1	1	1
Breast invasive carcinoma	TCGA-A8-A09G	1	1	1	1	1
Breast invasive carcinoma	TCGA-A8-A09I	1	1	1	1	1
Breast invasive carcinoma	TCGA-A8-A09K	1	1	1	1	1
Breast invasive carcinoma	TCGA-A8-A09M	1	1	1	1	1
Breast invasive carcinoma	TCGA-A8-A09N	1	1	1	1	1
Breast invasive carcinoma	TCGA-A8-A09Q	1	1	1	1	1
Breast invasive carcinoma	TCGA-A8-A09R	1	1	1	1	1
Breast invasive carcinoma	TCGA-A8-A09T	1	1	1	1	1
Breast invasive carcinoma	TCGA-A8-A09V	0	0	0	0	0
Breast invasive carcinoma	TCGA-A8-A09W	1	1	1	1	1
Breast invasive carcinoma	TCGA-A8-A09X	1	1	1	1	1
Breast invasive carcinoma	TCGA-A8-A09Z	1	1	1	1	1
Breast invasive carcinoma	TCGA-A8-A0A1	1	0	1	1	1
Breast invasive carcinoma	TCGA-A8-A0A2	1	1	1	1	1
Breast invasive carcinoma	TCGA-A8-A0A4	0	0	1	1	1
Breast invasive carcinoma	TCGA-A8-A0A6	1	1	1	1	1
Breast invasive carcinoma	TCGA-A8-A0A7	1	1	1	1	1
Breast invasive carcinoma	TCGA-A8-A0A9	1	1	1	1	1
Breast invasive carcinoma	TCGA-A8-A0AB	1	1	1	1	1
Breast invasive carcinoma	TCGA-A8-A0AD	1	0	1	0	1
Breast invasive carcinoma	TCGA-AC-A23C	1	0	1	0	1
Breast invasive carcinoma	TCGA-AC-A23E	1	1	1	1	1
Breast invasive carcinoma	TCGA-AC-A23G	0	0	1	1	1
Breast invasive carcinoma	TCGA-AC-A23H	1	1	1	1	1
Breast invasive carcinoma	TCGA-AC-A2B8	1	1	1	0	1
Breast invasive carcinoma	TCGA-AC-A2BK	1	1	1	1	1
Breast invasive carcinoma	TCGA-AC-A2BM	1	1	1	1	1
Breast invasive carcinoma	TCGA-AC-A2FB	0	1	1	1	1
Breast invasive carcinoma	TCGA-AC-A2FE	0	1	1	1	1

Breast invasive carcinoma	TCGA-AC-A2FF	1	1	1	1	1
Breast invasive carcinoma	TCGA-AC-A2FG	0	0	0	0	1
Breast invasive carcinoma	TCGA-AC-A2FK	0	0	0	0	0
Breast invasive carcinoma	TCGA-AC-A2FM	1	1	1	1	1
Breast invasive carcinoma	TCGA-AC-A2FO	1	1	0	1	1
Breast invasive carcinoma	TCGA-AC-A2QH	1	1	1	1	1
Breast invasive carcinoma	TCGA-AC-A2QI	1	1	1	0	1
Breast invasive carcinoma	TCGA-AC-A2QJ	1	1	1	1	1
Breast invasive carcinoma	TCGA-AC-A3BB	1	1	1	1	1
Breast invasive carcinoma	TCGA-AC-A3EH	1	1	1	1	1
Breast invasive carcinoma	TCGA-AC-A3HN	0	1	0	1	1
Breast invasive carcinoma	TCGA-AC-A3OD	1	1	1	1	1
Breast invasive carcinoma	TCGA-AC-A3QP	1	1	0	1	1
Breast invasive carcinoma	TCGA-AC-A3QQ	1	1	1	1	1
Breast invasive carcinoma	TCGA-AC-A3TM	1	1	1	1	1
Breast invasive carcinoma	TCGA-AC-A3TN	1	1	1	0	1
Breast invasive carcinoma	TCGA-AC-A3W5	1	1	1	1	1
Breast invasive carcinoma	TCGA-AC-A3W6	1	1	1	0	1
Breast invasive carcinoma	TCGA-AC-A3W7	1	1	1	1	1
Breast invasive carcinoma	TCGA-AC-A3YI	1	1	0	1	1
Breast invasive carcinoma	TCGA-AC-A3YJ	1	0	0	0	0
Breast invasive carcinoma	TCGA-AC-A4ZE	0	1	1	1	1
Breast invasive carcinoma	TCGA-AC-A5EH	1	1	1	1	1
Breast invasive carcinoma	TCGA-AC-A5XS	1	1	1	1	1
Breast invasive carcinoma	TCGA-AC-A5XU	1	1	1	1	1
Breast invasive carcinoma	TCGA-AC-A62V	1	1	1	1	1
Breast invasive carcinoma	TCGA-AC-A62X	1	1	1	1	1
Breast invasive carcinoma	TCGA-AC-A62Y	1	1	1	1	1
Breast invasive carcinoma	TCGA-AC-A6IV	1	1	1	1	1
Breast invasive carcinoma	TCGA-AC-A6IW	1	1	1	1	1
Breast invasive carcinoma	TCGA-AC-A6IX	1	1	1	1	1
Breast invasive carcinoma	TCGA-AC-A6NO	1	1	0	1	1
Breast invasive carcinoma	TCGA-AC-A7VB	1	1	1	1	1
Breast invasive carcinoma	TCGA-AC-A7VC	1	1	0	1	1
Breast invasive carcinoma	TCGA-AC-A8OP	1	1	1	1	1
Breast invasive carcinoma	TCGA-AC-A8OQ	1	1	1	1	1
Breast invasive carcinoma	TCGA-AC-A8OR	0	0	1	0	0
Breast invasive carcinoma	TCGA-AC-A8OS	1	1	1	0	1
Breast invasive carcinoma	TCGA-AN-A03X	1	1	0	1	1
Breast invasive carcinoma	TCGA-AN-A03Y	1	1	1	1	1
Breast invasive carcinoma	TCGA-AN-A041	1	1	1	1	1
Breast invasive carcinoma	TCGA-AN-A046	1	1	1	1	1
Breast invasive carcinoma	TCGA-AN-A049	1	1	1	1	1
Breast invasive carcinoma	TCGA-AN-A04A	1	1	1	1	1
Breast invasive carcinoma	TCGA-AN-A04C	1	1	1	1	1
Breast invasive carcinoma	TCGA-AN-A04D	1	1	1	1	1
Breast invasive carcinoma	TCGA-AN-A0AJ	1	1	1	1	1
Breast invasive carcinoma	TCGA-AN-A0AK	1	1	1	1	1
Breast invasive carcinoma	TCGA-AN-A0AL	1	1	1	1	1
Breast invasive carcinoma	TCGA-AN-A0AM	1	1	1	1	1
Breast invasive carcinoma	TCGA-AN-A0AR	1	1	1	1	1
Breast invasive carcinoma	TCGA-AN-A0AS	1	1	1	1	1
Breast invasive carcinoma	TCGA-AN-A0AT	1	1	1	1	1
Breast invasive carcinoma	TCGA-AN-A0FD	1	1	1	1	1
Breast invasive carcinoma	TCGA-AN-A0FF	1	0	1	1	1
Breast invasive carcinoma	TCGA-AN-A0FJ	1	1	1	1	1
Breast invasive carcinoma	TCGA-AN-A0FK	1	1	1	1	1
Breast invasive carcinoma	TCGA-AN-A0FL	1	1	1	1	1

Breast invasive carcinoma	TCGA-AN-A0FN	1	1	0	1	0
Breast invasive carcinoma	TCGA-AN-A0FS	1	1	1	0	1
Breast invasive carcinoma	TCGA-AN-A0FT	1	1	1	1	1
Breast invasive carcinoma	TCGA-AN-A0FV	1	1	1	1	1
Breast invasive carcinoma	TCGA-AN-A0FW	1	1	1	1	1
Breast invasive carcinoma	TCGA-AN-A0FX	1	1	1	1	1
Breast invasive carcinoma	TCGA-AN-A0FY	1	0	1	1	1
Breast invasive carcinoma	TCGA-AN-A0FZ	1	1	1	1	1
Breast invasive carcinoma	TCGA-AN-A0G0	0	0	0	0	0
Breast invasive carcinoma	TCGA-AN-A0XL	1	1	1	0	1
Breast invasive carcinoma	TCGA-AN-A0XN	1	1	1	1	1
Breast invasive carcinoma	TCGA-AN-A0XO	1	1	1	1	1
Breast invasive carcinoma	TCGA-AN-A0XP	1	1	1	1	1
Breast invasive carcinoma	TCGA-AN-A0XR	1	1	1	1	1
Breast invasive carcinoma	TCGA-AN-A0XS	1	1	0	1	1
Breast invasive carcinoma	TCGA-AN-A0XT	1	1	1	1	1
Breast invasive carcinoma	TCGA-AN-A0XU	1	1	1	1	1
Breast invasive carcinoma	TCGA-AN-A0XV	1	1	1	1	1
Breast invasive carcinoma	TCGA-AN-A0XW	1	1	1	1	1
Breast invasive carcinoma	TCGA-AO-A03L	1	1	1	1	1
Breast invasive carcinoma	TCGA-AO-A03M	1	1	1	1	1
Breast invasive carcinoma	TCGA-AO-A03N	1	1	1	1	1
Breast invasive carcinoma	TCGA-AO-A03O	1	1	1	1	1
Breast invasive carcinoma	TCGA-AO-A03P	1	1	1	1	1
Breast invasive carcinoma	TCGA-AO-A03R	1	1	1	0	1
Breast invasive carcinoma	TCGA-AO-A03T	1	0	1	1	1
Breast invasive carcinoma	TCGA-AO-A03U	1	0	0	0	1
Breast invasive carcinoma	TCGA-AO-A03V	1	1	1	1	1
Breast invasive carcinoma	TCGA-AO-A0J2	1	1	1	1	1
Breast invasive carcinoma	TCGA-AO-A0J3	1	1	1	1	1
Breast invasive carcinoma	TCGA-AO-A0J4	1	1	1	1	1
Breast invasive carcinoma	TCGA-AO-A0J5	1	1	1	1	1
Breast invasive carcinoma	TCGA-AO-A0J6	1	1	1	1	1
Breast invasive carcinoma	TCGA-AO-A0J7	1	1	1	1	1
Breast invasive carcinoma	TCGA-AO-A0J8	0	0	1	1	1
Breast invasive carcinoma	TCGA-AO-A0J9	1	1	0	1	1
Breast invasive carcinoma	TCGA-AO-A0JA	1	1	1	1	1
Breast invasive carcinoma	TCGA-AO-A0JB	1	1	1	1	1
Breast invasive carcinoma	TCGA-AO-A0JC	0	0	0	0	0
Breast invasive carcinoma	TCGA-AO-A0JD	1	1	1	1	1
Breast invasive carcinoma	TCGA-AO-A0JE	1	1	1	1	1
Breast invasive carcinoma	TCGA-AO-A0JF	1	0	0	0	1
Breast invasive carcinoma	TCGA-AO-A0JG	0	1	1	1	1
Breast invasive carcinoma	TCGA-AO-A0JI	0	1	1	1	1
Breast invasive carcinoma	TCGA-AO-A0JJ	0	0	1	0	1
Breast invasive carcinoma	TCGA-AO-A0JL	1	1	1	1	1
Breast invasive carcinoma	TCGA-AO-A0JM	1	1	1	1	1
Breast invasive carcinoma	TCGA-AO-A124	1	1	1	1	1
Breast invasive carcinoma	TCGA-AO-A125	1	0	0	0	0
Breast invasive carcinoma	TCGA-AO-A126	1	1	1	1	1
Breast invasive carcinoma	TCGA-AO-A128	0	1	1	1	1
Breast invasive carcinoma	TCGA-AO-A129	1	1	1	1	1
Breast invasive carcinoma	TCGA-AO-A12A	1	1	1	1	1
Breast invasive carcinoma	TCGA-AO-A12B	0	0	0	0	0
Breast invasive carcinoma	TCGA-AO-A12C	0	0	0	0	0
Breast invasive carcinoma	TCGA-AO-A12D	1	1	1	1	1
Breast invasive carcinoma	TCGA-AO-A12E	1	1	0	1	1
Breast invasive carcinoma	TCGA-AO-A12F	1	1	1	1	1

Breast invasive carcinoma	TCGA-AO-A12G	1	1	1	0	1
Breast invasive carcinoma	TCGA-AO-A12H	0	0	0	0	0
Breast invasive carcinoma	TCGA-AO-A1KO	0	0	0	0	0
Breast invasive carcinoma	TCGA-AO-A1KP	1	1	1	1	1
Breast invasive carcinoma	TCGA-AO-A1KQ	1	1	1	1	1
Breast invasive carcinoma	TCGA-AO-A1KR	1	1	1	1	1
Breast invasive carcinoma	TCGA-AO-A1KS	1	1	1	1	1
Breast invasive carcinoma	TCGA-AO-A1KT	1	1	1	1	1
Breast invasive carcinoma	TCGA-AQ-A04H	1	1	1	1	1
Breast invasive carcinoma	TCGA-AQ-A04J	1	1	1	1	1
Breast invasive carcinoma	TCGA-AQ-A04L	1	1	1	1	1
Breast invasive carcinoma	TCGA-AQ-A0Y5	1	1	1	1	1
Breast invasive carcinoma	TCGA-AQ-A1H2	1	1	1	1	1
Breast invasive carcinoma	TCGA-AQ-A1H3	1	0	1	0	1
Breast invasive carcinoma	TCGA-AQ-A54N	1	1	1	1	1
Breast invasive carcinoma	TCGA-AQ-A54O	1	1	1	1	1
Breast invasive carcinoma	TCGA-AQ-A7U7	1	1	1	1	1
Breast invasive carcinoma	TCGA-AR-A0TP	1	1	1	1	1
Breast invasive carcinoma	TCGA-AR-A0TQ	1	1	1	1	1
Breast invasive carcinoma	TCGA-AR-A0TR	1	1	1	1	1
Breast invasive carcinoma	TCGA-AR-A0TS	1	1	1	1	1
Breast invasive carcinoma	TCGA-AR-A0TT	1	1	1	1	1
Breast invasive carcinoma	TCGA-AR-A0TU	0	0	0	0	0
Breast invasive carcinoma	TCGA-AR-A0TV	1	1	1	1	1
Breast invasive carcinoma	TCGA-AR-A0TW	1	1	1	1	1
Breast invasive carcinoma	TCGA-AR-A0TX	0	1	1	1	1
Breast invasive carcinoma	TCGA-AR-A0TY	1	1	1	1	1
Breast invasive carcinoma	TCGA-AR-A0TZ	1	1	1	1	1
Breast invasive carcinoma	TCGA-AR-A0U0	1	1	1	1	1
Breast invasive carcinoma	TCGA-AR-A0U2	1	1	1	1	1
Breast invasive carcinoma	TCGA-AR-A0U3	1	1	1	1	1
Breast invasive carcinoma	TCGA-AR-A0U4	1	1	1	1	1
Breast invasive carcinoma	TCGA-AR-A1AH	1	1	1	1	1
Breast invasive carcinoma	TCGA-AR-A1AI	1	1	1	1	1
Breast invasive carcinoma	TCGA-AR-A1AJ	1	1	1	1	1
Breast invasive carcinoma	TCGA-AR-A1AK	0	1	1	0	1
Breast invasive carcinoma	TCGA-AR-A1AL	1	0	0	1	0
Breast invasive carcinoma	TCGA-AR-A1AM	1	0	1	1	1
Breast invasive carcinoma	TCGA-AR-A1AN	1	1	1	1	1
Breast invasive carcinoma	TCGA-AR-A1AO	1	0	0	0	0
Breast invasive carcinoma	TCGA-AR-A1AP	1	1	1	1	1
Breast invasive carcinoma	TCGA-AR-A1AQ	1	1	1	1	1
Breast invasive carcinoma	TCGA-AR-A1AR	1	1	1	1	1
Breast invasive carcinoma	TCGA-AR-A1AS	1	1	1	0	1
Breast invasive carcinoma	TCGA-AR-A1AT	0	0	0	0	0
Breast invasive carcinoma	TCGA-AR-A1AU	1	0	1	1	1
Breast invasive carcinoma	TCGA-AR-A1AV	1	0	0	1	1
Breast invasive carcinoma	TCGA-AR-A1AW	1	1	1	1	1
Breast invasive carcinoma	TCGA-AR-A1AX	1	1	1	0	1
Breast invasive carcinoma	TCGA-AR-A1AY	1	1	1	1	1
Breast invasive carcinoma	TCGA-AR-A24H	1	1	1	1	1
Breast invasive carcinoma	TCGA-AR-A24K	1	1	1	1	1
Breast invasive carcinoma	TCGA-AR-A24L	1	1	1	1	1
Breast invasive carcinoma	TCGA-AR-A24M	1	0	1	0	0
Breast invasive carcinoma	TCGA-AR-A24N	1	1	0	1	1
Breast invasive carcinoma	TCGA-AR-A24O	1	1	1	1	1
Breast invasive carcinoma	TCGA-AR-A24P	0	0	1	1	1
Breast invasive carcinoma	TCGA-AR-A24Q	1	1	1	1	1

Breast invasive carcinoma	TCGA-AR-A24R	1	1	1	1	1
Breast invasive carcinoma	TCGA-AR-A24S	1	1	1	1	1
Breast invasive carcinoma	TCGA-AR-A24T	1	1	1	1	1
Breast invasive carcinoma	TCGA-AR-A24U	1	1	1	1	1
Breast invasive carcinoma	TCGA-AR-A24V	1	1	1	1	1
Breast invasive carcinoma	TCGA-AR-A24W	1	1	1	1	1
Breast invasive carcinoma	TCGA-AR-A24X	0	0	0	1	1
Breast invasive carcinoma	TCGA-AR-A24Z	1	1	1	1	1
Breast invasive carcinoma	TCGA-AR-A250	1	1	1	1	1
Breast invasive carcinoma	TCGA-AR-A251	1	1	1	1	1
Breast invasive carcinoma	TCGA-AR-A252	1	1	1	1	1
Breast invasive carcinoma	TCGA-AR-A254	1	1	1	1	1
Breast invasive carcinoma	TCGA-AR-A255	1	1	1	1	1
Breast invasive carcinoma	TCGA-AR-A256	1	1	1	1	1
Breast invasive carcinoma	TCGA-AR-A2LE	0	1	1	0	1
Breast invasive carcinoma	TCGA-AR-A2LH	0	0	0	0	0
Breast invasive carcinoma	TCGA-AR-A2LJ	1	0	1	0	1
Breast invasive carcinoma	TCGA-AR-A2LK	1	1	1	1	1
Breast invasive carcinoma	TCGA-AR-A2LL	1	1	1	1	1
Breast invasive carcinoma	TCGA-AR-A2LM	1	0	1	0	1
Breast invasive carcinoma	TCGA-AR-A2LN	1	0	0	0	0
Breast invasive carcinoma	TCGA-AR-A2LO	1	1	1	1	1
Breast invasive carcinoma	TCGA-AR-A2LQ	0	0	0	0	0
Breast invasive carcinoma	TCGA-AR-A2LR	1	1	1	0	1
Breast invasive carcinoma	TCGA-AR-A5QM	1	0	1	0	1
Breast invasive carcinoma	TCGA-AR-A5QN	1	1	0	1	1
Breast invasive carcinoma	TCGA-AR-A5QP	1	1	0	1	1
Breast invasive carcinoma	TCGA-AR-A5QQ	1	1	1	0	1
Breast invasive carcinoma	TCGA-B6-A0I1	1	1	1	1	1
Breast invasive carcinoma	TCGA-B6-A0I2	1	0	1	0	0
Breast invasive carcinoma	TCGA-B6-A0I5	1	0	0	0	0
Breast invasive carcinoma	TCGA-B6-A0I6	0	0	0	0	0
Breast invasive carcinoma	TCGA-B6-A0I8	0	0	0	0	0
Breast invasive carcinoma	TCGA-B6-A0I9	1	1	1	1	1
Breast invasive carcinoma	TCGA-B6-A0IA	1	0	1	0	0
Breast invasive carcinoma	TCGA-B6-A0IB	1	1	1	1	1
Breast invasive carcinoma	TCGA-B6-A0IC	1	0	0	1	1
Breast invasive carcinoma	TCGA-B6-A0IE	1	1	1	1	1
Breast invasive carcinoma	TCGA-B6-A0IG	1	1	1	1	1
Breast invasive carcinoma	TCGA-B6-A0IH	1	1	1	1	1
Breast invasive carcinoma	TCGA-B6-A0IJ	1	1	1	1	1
Breast invasive carcinoma	TCGA-B6-A0IK	1	1	1	1	1
Breast invasive carcinoma	TCGA-B6-A0IM	0	1	1	0	1
Breast invasive carcinoma	TCGA-B6-A0IN	1	1	1	1	1
Breast invasive carcinoma	TCGA-B6-A0IO	1	1	1	1	1
Breast invasive carcinoma	TCGA-B6-A0IP	1	0	0	0	0
Breast invasive carcinoma	TCGA-B6-A0IQ	1	1	1	1	1
Breast invasive carcinoma	TCGA-B6-A0RE	1	1	1	1	1
Breast invasive carcinoma	TCGA-B6-A0RG	1	1	1	1	1
Breast invasive carcinoma	TCGA-B6-A0RH	1	1	1	0	1
Breast invasive carcinoma	TCGA-B6-A0RI	1	0	1	1	1
Breast invasive carcinoma	TCGA-B6-A0RL	1	1	1	1	1
Breast invasive carcinoma	TCGA-B6-A0RM	1	0	0	1	1
Breast invasive carcinoma	TCGA-B6-A0RN	1	0	0	1	1
Breast invasive carcinoma	TCGA-B6-A0RO	1	1	1	1	1
Breast invasive carcinoma	TCGA-B6-A0RP	1	1	1	1	1
Breast invasive carcinoma	TCGA-B6-A0RQ	1	1	0	0	0
Breast invasive carcinoma	TCGA-B6-A0RS	1	1	1	1	1

Breast invasive carcinoma	TCGA-B6-A0RT	1	1	0	1	1
Breast invasive carcinoma	TCGA-B6-A0RU	1	1	1	1	1
Breast invasive carcinoma	TCGA-B6-A0RV	1	1	1	1	1
Breast invasive carcinoma	TCGA-B6-A0WS	1	1	1	1	1
Breast invasive carcinoma	TCGA-B6-A0WT	1	1	0	0	1
Breast invasive carcinoma	TCGA-B6-A0WV	1	1	1	1	1
Breast invasive carcinoma	TCGA-B6-A0WW	1	1	0	1	1
Breast invasive carcinoma	TCGA-B6-A0WX	1	1	1	1	1
Breast invasive carcinoma	TCGA-B6-A0WY	1	1	1	1	1
Breast invasive carcinoma	TCGA-B6-A0WZ	0	0	1	0	1
Breast invasive carcinoma	TCGA-B6-A0X0	0	1	0	0	0
Breast invasive carcinoma	TCGA-B6-A0X1	1	1	1	1	1
Breast invasive carcinoma	TCGA-B6-A0X4	0	0	0	0	0
Breast invasive carcinoma	TCGA-B6-A0X5	1	1	0	1	1
Breast invasive carcinoma	TCGA-B6-A0X7	1	0	1	1	1
Breast invasive carcinoma	TCGA-B6-A1KC	1	1	1	1	1
Breast invasive carcinoma	TCGA-B6-A1KF	1	1	1	1	1
Breast invasive carcinoma	TCGA-B6-A1KI	1	1	1	1	1
Breast invasive carcinoma	TCGA-B6-A1KN	1	1	1	1	1
Breast invasive carcinoma	TCGA-B6-A2IU	1	0	1	0	0
Breast invasive carcinoma	TCGA-B6-A3ZX	1	1	1	1	1
Breast invasive carcinoma	TCGA-B6-A400	1	1	1	0	1
Breast invasive carcinoma	TCGA-B6-A401	1	1	1	1	1
Breast invasive carcinoma	TCGA-B6-A402	1	1	1	1	1
Breast invasive carcinoma	TCGA-B6-A408	1	1	1	1	1
Breast invasive carcinoma	TCGA-B6-A409	1	1	1	1	1
Breast invasive carcinoma	TCGA-B6-A40B	1	1	0	1	1
Breast invasive carcinoma	TCGA-B6-A40C	0	1	1	1	1
Breast invasive carcinoma	TCGA-BH-A0AU	1	1	1	1	1
Breast invasive carcinoma	TCGA-BH-A0AV	1	1	1	1	1
Breast invasive carcinoma	TCGA-BH-A0AW	1	1	1	1	1
Breast invasive carcinoma	TCGA-BH-A0AY	0	1	1	1	1
Breast invasive carcinoma	TCGA-BH-A0AZ	1	1	1	1	1
Breast invasive carcinoma	TCGA-BH-A0B0	1	1	1	1	1
Breast invasive carcinoma	TCGA-BH-A0B1	0	0	0	0	0
Breast invasive carcinoma	TCGA-BH-A0B3	1	1	1	1	1
Breast invasive carcinoma	TCGA-BH-A0B4	1	1	1	1	1
Breast invasive carcinoma	TCGA-BH-A0B5	1	1	1	1	1
Breast invasive carcinoma	TCGA-BH-A0B6	1	1	1	1	1
Breast invasive carcinoma	TCGA-BH-A0B7	1	1	1	1	1
Breast invasive carcinoma	TCGA-BH-A0B8	0	0	0	0	0
Breast invasive carcinoma	TCGA-BH-A0B9	1	1	1	1	1
Breast invasive carcinoma	TCGA-BH-A0BA	1	1	1	1	1
Breast invasive carcinoma	TCGA-BH-A0BC	1	1	1	0	1
Breast invasive carcinoma	TCGA-BH-A0BD	1	0	1	0	1
Breast invasive carcinoma	TCGA-BH-A0BF	1	1	1	1	1
Breast invasive carcinoma	TCGA-BH-A0BG	1	1	1	1	1
Breast invasive carcinoma	TCGA-BH-A0BJ	1	0	1	1	1
Breast invasive carcinoma	TCGA-BH-A0BL	1	1	1	1	1
Breast invasive carcinoma	TCGA-BH-A0BM	1	0	1	0	1
Breast invasive carcinoma	TCGA-BH-A0BO	1	1	0	1	0
Breast invasive carcinoma	TCGA-BH-A0BP	1	1	1	1	1
Breast invasive carcinoma	TCGA-BH-A0BQ	0	0	1	0	1
Breast invasive carcinoma	TCGA-BH-A0BR	1	1	1	1	1
Breast invasive carcinoma	TCGA-BH-A0BS	0	0	0	0	0
Breast invasive carcinoma	TCGA-BH-A0BT	1	1	1	1	1
Breast invasive carcinoma	TCGA-BH-A0BV	1	1	1	1	1
Breast invasive carcinoma	TCGA-BH-A0BW	1	1	1	1	1

Breast invasive carcinoma	TCGA-BH-A0BZ	1	1	1	1	1
Breast invasive carcinoma	TCGA-BH-A0C0	1	1	1	1	1
Breast invasive carcinoma	TCGA-BH-A0C1	1	1	1	1	1
Breast invasive carcinoma	TCGA-BH-A0C3	1	1	1	1	1
Breast invasive carcinoma	TCGA-BH-A0C7	1	1	1	1	1
Breast invasive carcinoma	TCGA-BH-A0DD	1	1	1	1	1
Breast invasive carcinoma	TCGA-BH-A0DE	1	1	1	1	1
Breast invasive carcinoma	TCGA-BH-A0DG	1	1	1	1	1
Breast invasive carcinoma	TCGA-BH-A0DH	1	1	1	1	1
Breast invasive carcinoma	TCGA-BH-A0DI	1	1	1	1	1
Breast invasive carcinoma	TCGA-BH-A0DK	1	1	1	1	1
Breast invasive carcinoma	TCGA-BH-A0DL	1	1	1	1	1
Breast invasive carcinoma	TCGA-BH-A0DO	1	0	0	0	0
Breast invasive carcinoma	TCGA-BH-A0DP	1	1	1	1	1
Breast invasive carcinoma	TCGA-BH-A0DQ	1	1	1	1	1
Breast invasive carcinoma	TCGA-BH-A0DS	1	0	1	1	1
Breast invasive carcinoma	TCGA-BH-A0DT	1	0	0	0	0
Breast invasive carcinoma	TCGA-BH-A0DV	1	1	0	1	0
Breast invasive carcinoma	TCGA-BH-A0DX	1	0	1	1	1
Breast invasive carcinoma	TCGA-BH-A0DZ	1	1	1	1	1
Breast invasive carcinoma	TCGA-BH-A0E0	1	1	1	1	1
Breast invasive carcinoma	TCGA-BH-A0E1	0	0	0	1	0
Breast invasive carcinoma	TCGA-BH-A0E2	0	1	1	1	1
Breast invasive carcinoma	TCGA-BH-A0E6	1	1	1	1	1
Breast invasive carcinoma	TCGA-BH-A0E7	0	1	0	0	0
Breast invasive carcinoma	TCGA-BH-A0E9	1	1	1	1	1
Breast invasive carcinoma	TCGA-BH-A0EA	1	0	0	0	0
Breast invasive carcinoma	TCGA-BH-A0EB	1	1	1	1	1
Breast invasive carcinoma	TCGA-BH-A0EE	1	1	1	0	1
Breast invasive carcinoma	TCGA-BH-A0EI	0	1	1	0	1
Breast invasive carcinoma	TCGA-BH-A0GY	1	1	0	1	1
Breast invasive carcinoma	TCGA-BH-A0GZ	1	1	0	1	1
Breast invasive carcinoma	TCGA-BH-A0H0	1	1	1	1	1
Breast invasive carcinoma	TCGA-BH-A0H3	1	0	0	0	0
Breast invasive carcinoma	TCGA-BH-A0H5	0	1	0	1	0
Breast invasive carcinoma	TCGA-BH-A0H6	1	0	0	1	1
Breast invasive carcinoma	TCGA-BH-A0H7	1	1	1	1	1
Breast invasive carcinoma	TCGA-BH-A0H9	1	1	1	1	1
Breast invasive carcinoma	TCGA-BH-A0HA	1	1	1	1	1
Breast invasive carcinoma	TCGA-BH-A0HB	1	1	1	1	1
Breast invasive carcinoma	TCGA-BH-A0HF	0	0	0	0	0
Breast invasive carcinoma	TCGA-BH-A0HI	1	0	0	0	1
Breast invasive carcinoma	TCGA-BH-A0HK	0	0	1	0	1
Breast invasive carcinoma	TCGA-BH-A0HL	0	0	0	0	0
Breast invasive carcinoma	TCGA-BH-A0HN	0	0	0	0	0
Breast invasive carcinoma	TCGA-BH-A0HO	0	0	0	0	0
Breast invasive carcinoma	TCGA-BH-A0HP	1	1	1	1	1
Breast invasive carcinoma	TCGA-BH-A0HQ	1	0	0	1	1
Breast invasive carcinoma	TCGA-BH-A0HU	1	1	1	1	1
Breast invasive carcinoma	TCGA-BH-A0HW	1	1	1	1	1
Breast invasive carcinoma	TCGA-BH-A0HX	1	0	1	1	1
Breast invasive carcinoma	TCGA-BH-A0HY	1	1	1	1	1
Breast invasive carcinoma	TCGA-BH-A0RX	1	1	0	1	1
Breast invasive carcinoma	TCGA-BH-A0W3	1	1	1	1	1
Breast invasive carcinoma	TCGA-BH-A0W4	1	0	1	0	1
Breast invasive carcinoma	TCGA-BH-A0W5	1	1	0	1	1
Breast invasive carcinoma	TCGA-BH-A0W7	1	0	1	1	1
Breast invasive carcinoma	TCGA-BH-A0WA	1	1	1	1	1

Breast invasive carcinoma	TCGA-BH-A18F	1	1	1	1	1
Breast invasive carcinoma	TCGA-BH-A18G	0	1	1	0	1
Breast invasive carcinoma	TCGA-BH-A18H	1	1	1	1	1
Breast invasive carcinoma	TCGA-BH-A18I	1	1	1	1	1
Breast invasive carcinoma	TCGA-BH-A18J	1	1	1	1	1
Breast invasive carcinoma	TCGA-BH-A18K	1	1	1	1	1
Breast invasive carcinoma	TCGA-BH-A18L	1	1	1	1	1
Breast invasive carcinoma	TCGA-BH-A18M	1	0	0	1	1
Breast invasive carcinoma	TCGA-BH-A18N	0	1	0	1	0
Breast invasive carcinoma	TCGA-BH-A18P	1	1	1	1	1
Breast invasive carcinoma	TCGA-BH-A18Q	1	1	1	1	1
Breast invasive carcinoma	TCGA-BH-A18R	1	1	1	1	1
Breast invasive carcinoma	TCGA-BH-A18S	1	1	1	1	1
Breast invasive carcinoma	TCGA-BH-A18T	1	1	1	1	1
Breast invasive carcinoma	TCGA-BH-A18U	1	1	1	1	1
Breast invasive carcinoma	TCGA-BH-A18V	1	1	1	1	1
Breast invasive carcinoma	TCGA-BH-A1EN	1	1	1	1	1
Breast invasive carcinoma	TCGA-BH-A1EO	1	0	1	1	1
Breast invasive carcinoma	TCGA-BH-A1ES	1	1	1	1	1
Breast invasive carcinoma	TCGA-BH-A1ET	1	0	0	1	1
Breast invasive carcinoma	TCGA-BH-A1EU	1	1	1	1	1
Breast invasive carcinoma	TCGA-BH-A1EV	1	1	1	1	1
Breast invasive carcinoma	TCGA-BH-A1EW	1	0	1	1	1
Breast invasive carcinoma	TCGA-BH-A1EX	0	0	0	1	1
Breast invasive carcinoma	TCGA-BH-A1EY	1	1	1	1	1
Breast invasive carcinoma	TCGA-BH-A1F0	1	0	1	0	1
Breast invasive carcinoma	TCGA-BH-A1F2	1	1	1	1	1
Breast invasive carcinoma	TCGA-BH-A1F5	0	1	1	1	1
Breast invasive carcinoma	TCGA-BH-A1F6	1	1	1	1	1
Breast invasive carcinoma	TCGA-BH-A1F8	1	1	1	1	1
Breast invasive carcinoma	TCGA-BH-A1FB	0	1	0	0	0
Breast invasive carcinoma	TCGA-BH-A1FC	1	1	1	1	1
Breast invasive carcinoma	TCGA-BH-A1FD	1	1	1	1	1
Breast invasive carcinoma	TCGA-BH-A1FE	1	1	1	1	1
Breast invasive carcinoma	TCGA-BH-A1FG	1	1	1	1	1
Breast invasive carcinoma	TCGA-BH-A1FH	1	1	1	0	1
Breast invasive carcinoma	TCGA-BH-A1FJ	1	0	1	0	1
Breast invasive carcinoma	TCGA-BH-A1FL	1	0	0	1	1
Breast invasive carcinoma	TCGA-BH-A1FM	1	1	1	1	1
Breast invasive carcinoma	TCGA-BH-A1FN	1	1	1	1	1
Breast invasive carcinoma	TCGA-BH-A1FR	0	0	0	0	0
Breast invasive carcinoma	TCGA-BH-A1FU	1	1	1	1	1
Breast invasive carcinoma	TCGA-BH-A201	1	1	1	0	1
Breast invasive carcinoma	TCGA-BH-A202	1	1	1	1	1
Breast invasive carcinoma	TCGA-BH-A203	1	1	1	1	1
Breast invasive carcinoma	TCGA-BH-A204	1	1	1	1	1
Breast invasive carcinoma	TCGA-BH-A208	1	1	1	1	1
Breast invasive carcinoma	TCGA-BH-A209	1	1	1	1	1
Breast invasive carcinoma	TCGA-BH-A28O	1	0	0	0	0
Breast invasive carcinoma	TCGA-BH-A28Q	1	0	1	0	1
Breast invasive carcinoma	TCGA-BH-A2L8	1	0	1	0	0
Breast invasive carcinoma	TCGA-BH-A42T	1	1	1	1	1
Breast invasive carcinoma	TCGA-BH-A42U	0	0	0	0	0
Breast invasive carcinoma	TCGA-BH-A42V	1	1	0	1	1
Breast invasive carcinoma	TCGA-BH-A5IZ	1	1	1	1	1
Breast invasive carcinoma	TCGA-BH-A5J0	1	1	1	1	1
Breast invasive carcinoma	TCGA-BH-A6R8	0	0	0	1	1
Breast invasive carcinoma	TCGA-BH-A6R9	0	0	1	1	1

Breast invasive carcinoma	TCGA-BH-A8FY	0	0	0	0	1
Breast invasive carcinoma	TCGA-BH-A8FZ	1	1	1	0	1
Breast invasive carcinoma	TCGA-BH-A8G0	1	1	0	1	0
Breast invasive carcinoma	TCGA-BH-AB28	1	1	0	1	1
Breast invasive carcinoma	TCGA-C8-A12K	1	1	1	1	1
Breast invasive carcinoma	TCGA-C8-A12L	1	1	1	1	1
Breast invasive carcinoma	TCGA-C8-A12M	1	1	1	1	1
Breast invasive carcinoma	TCGA-C8-A12N	1	1	0	1	1
Breast invasive carcinoma	TCGA-C8-A12O	1	1	1	0	1
Breast invasive carcinoma	TCGA-C8-A12P	1	1	1	1	1
Breast invasive carcinoma	TCGA-C8-A12Q	1	1	1	0	1
Breast invasive carcinoma	TCGA-C8-A12T	1	1	1	1	1
Breast invasive carcinoma	TCGA-C8-A12U	1	1	1	1	1
Breast invasive carcinoma	TCGA-C8-A12V	0	0	0	0	0
Breast invasive carcinoma	TCGA-C8-A12W	1	1	1	1	1
Breast invasive carcinoma	TCGA-C8-A12X	1	1	1	1	1
Breast invasive carcinoma	TCGA-C8-A12Y	1	0	1	1	1
Breast invasive carcinoma	TCGA-C8-A12Z	1	1	1	1	1
Breast invasive carcinoma	TCGA-C8-A130	1	1	1	1	1
Breast invasive carcinoma	TCGA-C8-A131	1	1	1	1	1
Breast invasive carcinoma	TCGA-C8-A132	1	1	1	1	1
Breast invasive carcinoma	TCGA-C8-A133	1	0	0	0	0
Breast invasive carcinoma	TCGA-C8-A134	1	1	1	1	1
Breast invasive carcinoma	TCGA-C8-A135	1	1	1	1	1
Breast invasive carcinoma	TCGA-C8-A137	1	1	1	1	1
Breast invasive carcinoma	TCGA-C8-A138	1	1	1	1	1
Breast invasive carcinoma	TCGA-C8-A1HE	1	1	0	1	1
Breast invasive carcinoma	TCGA-C8-A1HF	1	1	1	1	1
Breast invasive carcinoma	TCGA-C8-A1HG	1	1	1	1	1
Breast invasive carcinoma	TCGA-C8-A1HI	1	1	1	1	1
Breast invasive carcinoma	TCGA-C8-A1HJ	1	1	1	1	1
Breast invasive carcinoma	TCGA-C8-A1HK	1	1	1	1	1
Breast invasive carcinoma	TCGA-C8-A1HL	1	1	1	1	1
Breast invasive carcinoma	TCGA-C8-A1HM	1	1	1	1	1
Breast invasive carcinoma	TCGA-C8-A1HN	1	1	1	1	1
Breast invasive carcinoma	TCGA-C8-A1HO	0	0	1	0	1
Breast invasive carcinoma	TCGA-C8-A26V	1	1	1	1	1
Breast invasive carcinoma	TCGA-C8-A26W	1	1	1	1	1
Breast invasive carcinoma	TCGA-C8-A26X	1	1	1	1	1
Breast invasive carcinoma	TCGA-C8-A26Y	1	1	1	1	1
Breast invasive carcinoma	TCGA-C8-A26Z	1	1	1	1	1
Breast invasive carcinoma	TCGA-C8-A273	1	1	1	1	1
Breast invasive carcinoma	TCGA-C8-A274	1	1	1	1	1
Breast invasive carcinoma	TCGA-C8-A275	1	1	1	0	1
Breast invasive carcinoma	TCGA-C8-A278	1	1	1	1	1
Breast invasive carcinoma	TCGA-C8-A27A	1	1	1	1	1
Breast invasive carcinoma	TCGA-C8-A27B	1	1	1	1	1
Breast invasive carcinoma	TCGA-C8-A3M7	1	1	1	1	1
Breast invasive carcinoma	TCGA-C8-A3M8	1	1	1	1	1
Breast invasive carcinoma	TCGA-C8-A8HP	1	1	1	1	1
Breast invasive carcinoma	TCGA-C8-A8HQ	1	1	1	1	1
Breast invasive carcinoma	TCGA-C8-A8HR	1	1	1	0	1
Breast invasive carcinoma	TCGA-D8-A13Y	1	1	1	1	1
Breast invasive carcinoma	TCGA-D8-A13Z	1	1	1	1	1
Breast invasive carcinoma	TCGA-D8-A140	1	1	1	1	1
Breast invasive carcinoma	TCGA-D8-A141	0	0	0	0	0
Breast invasive carcinoma	TCGA-D8-A142	1	1	1	1	1
Breast invasive carcinoma	TCGA-D8-A143	1	1	1	1	1

Breast invasive carcinoma	TCGA-D8-A145	1	1	1	1	1
Breast invasive carcinoma	TCGA-D8-A146	1	1	1	1	1
Breast invasive carcinoma	TCGA-D8-A147	1	1	1	1	1
Breast invasive carcinoma	TCGA-D8-A1J8	1	1	1	1	1
Breast invasive carcinoma	TCGA-D8-A1J9	1	1	1	1	1
Breast invasive carcinoma	TCGA-D8-A1JA	1	1	1	1	1
Breast invasive carcinoma	TCGA-D8-A1JB	1	1	1	1	1
Breast invasive carcinoma	TCGA-D8-A1JC	1	1	1	1	1
Breast invasive carcinoma	TCGA-D8-A1JD	1	1	1	1	1
Breast invasive carcinoma	TCGA-D8-A1JE	1	1	1	1	1
Breast invasive carcinoma	TCGA-D8-A1JF	1	1	1	1	1
Breast invasive carcinoma	TCGA-D8-A1JG	1	1	1	1	1
Breast invasive carcinoma	TCGA-D8-A1JH	1	0	0	0	0
Breast invasive carcinoma	TCGA-D8-A1JI	1	1	1	1	1
Breast invasive carcinoma	TCGA-D8-A1JJ	1	1	1	1	1
Breast invasive carcinoma	TCGA-D8-A1JK	1	1	1	1	1
Breast invasive carcinoma	TCGA-D8-A1JL	1	0	1	0	1
Breast invasive carcinoma	TCGA-D8-A1JM	1	1	1	1	1
Breast invasive carcinoma	TCGA-D8-A1JN	1	1	1	1	1
Breast invasive carcinoma	TCGA-D8-A1JP	1	1	1	1	1
Breast invasive carcinoma	TCGA-D8-A1JS	1	0	1	0	0
Breast invasive carcinoma	TCGA-D8-A1JT	1	0	1	1	1
Breast invasive carcinoma	TCGA-D8-A1JU	1	0	0	1	1
Breast invasive carcinoma	TCGA-D8-A1X5	1	1	1	1	1
Breast invasive carcinoma	TCGA-D8-A1X6	1	1	1	0	1
Breast invasive carcinoma	TCGA-D8-A1X7	1	0	1	0	1
Breast invasive carcinoma	TCGA-D8-A1X8	0	1	0	1	1
Breast invasive carcinoma	TCGA-D8-A1X9	1	1	1	1	1
Breast invasive carcinoma	TCGA-D8-A1XA	1	1	1	1	1
Breast invasive carcinoma	TCGA-D8-A1XB	1	0	1	1	1
Breast invasive carcinoma	TCGA-D8-A1XC	0	0	0	0	0
Breast invasive carcinoma	TCGA-D8-A1XD	1	1	1	1	1
Breast invasive carcinoma	TCGA-D8-A1XF	1	1	1	1	1
Breast invasive carcinoma	TCGA-D8-A1XG	1	1	0	1	1
Breast invasive carcinoma	TCGA-D8-A1XJ	1	1	1	1	1
Breast invasive carcinoma	TCGA-D8-A1XK	1	1	1	1	1
Breast invasive carcinoma	TCGA-D8-A1XL	1	1	1	1	1
Breast invasive carcinoma	TCGA-D8-A1XM	1	0	1	1	1
Breast invasive carcinoma	TCGA-D8-A1XO	1	1	1	1	1
Breast invasive carcinoma	TCGA-D8-A1XQ	1	1	1	1	1
Breast invasive carcinoma	TCGA-D8-A1XR	1	1	1	1	1
Breast invasive carcinoma	TCGA-D8-A1XS	1	1	0	1	1
Breast invasive carcinoma	TCGA-D8-A1XT	1	1	1	1	1
Breast invasive carcinoma	TCGA-D8-A1XU	1	1	1	1	1
Breast invasive carcinoma	TCGA-D8-A1XV	1	1	1	1	1
Breast invasive carcinoma	TCGA-D8-A1XW	1	0	1	1	1
Breast invasive carcinoma	TCGA-D8-A1XY	1	1	1	1	1
Breast invasive carcinoma	TCGA-D8-A1XZ	1	1	1	1	1
Breast invasive carcinoma	TCGA-D8-A1Y0	1	1	1	1	1
Breast invasive carcinoma	TCGA-D8-A1Y1	1	1	1	1	1
Breast invasive carcinoma	TCGA-D8-A1Y2	1	1	1	1	1
Breast invasive carcinoma	TCGA-D8-A1Y3	1	1	1	1	1
Breast invasive carcinoma	TCGA-D8-A27E	0	0	1	0	0
Breast invasive carcinoma	TCGA-D8-A27F	1	1	1	1	1
Breast invasive carcinoma	TCGA-D8-A27G	1	1	1	0	1
Breast invasive carcinoma	TCGA-D8-A27H	1	1	1	1	1
Breast invasive carcinoma	TCGA-D8-A27I	1	1	0	1	1
Breast invasive carcinoma	TCGA-D8-A27K	1	0	0	0	0

Breast invasive carcinoma	TCGA-D8-A27L	1	1	0	1	1
Breast invasive carcinoma	TCGA-D8-A27M	1	1	1	1	1
Breast invasive carcinoma	TCGA-D8-A27N	1	1	1	1	1
Breast invasive carcinoma	TCGA-D8-A27P	1	0	0	0	0
Breast invasive carcinoma	TCGA-D8-A27R	1	1	1	0	1
Breast invasive carcinoma	TCGA-D8-A27T	1	1	1	1	1
Breast invasive carcinoma	TCGA-D8-A27V	1	1	1	1	1
Breast invasive carcinoma	TCGA-D8-A27W	1	1	1	1	1
Breast invasive carcinoma	TCGA-D8-A3Z5	0	0	0	1	1
Breast invasive carcinoma	TCGA-D8-A3Z6	1	1	0	0	1
Breast invasive carcinoma	TCGA-D8-A4Z1	1	1	0	1	1
Breast invasive carcinoma	TCGA-D8-A73U	1	1	1	1	1
Breast invasive carcinoma	TCGA-D8-A73W	0	1	1	1	1
Breast invasive carcinoma	TCGA-D8-A73X	0	0	0	1	1
Breast invasive carcinoma	TCGA-E2-A105	1	1	1	1	1
Breast invasive carcinoma	TCGA-E2-A106	0	0	0	0	0
Breast invasive carcinoma	TCGA-E2-A107	1	1	1	1	1
Breast invasive carcinoma	TCGA-E2-A108	1	1	0	1	1
Breast invasive carcinoma	TCGA-E2-A109	1	1	1	1	1
Breast invasive carcinoma	TCGA-E2-A10A	1	1	1	1	1
Breast invasive carcinoma	TCGA-E2-A10B	1	0	0	0	0
Breast invasive carcinoma	TCGA-E2-A10C	1	1	1	1	1
Breast invasive carcinoma	TCGA-E2-A10E	1	1	0	1	1
Breast invasive carcinoma	TCGA-E2-A10F	1	0	1	1	1
Breast invasive carcinoma	TCGA-E2-A14N	1	1	1	1	1
Breast invasive carcinoma	TCGA-E2-A14O	1	1	1	1	1
Breast invasive carcinoma	TCGA-E2-A14P	1	1	1	1	1
Breast invasive carcinoma	TCGA-E2-A14Q	1	1	1	1	1
Breast invasive carcinoma	TCGA-E2-A14R	1	1	1	1	1
Breast invasive carcinoma	TCGA-E2-A14S	1	1	1	0	1
Breast invasive carcinoma	TCGA-E2-A14T	1	1	1	1	1
Breast invasive carcinoma	TCGA-E2-A14U	1	0	0	0	0
Breast invasive carcinoma	TCGA-E2-A14V	1	1	1	1	1
Breast invasive carcinoma	TCGA-E2-A14W	1	1	1	1	1
Breast invasive carcinoma	TCGA-E2-A14X	1	1	1	0	1
Breast invasive carcinoma	TCGA-E2-A14Y	1	1	1	1	1
Breast invasive carcinoma	TCGA-E2-A14Z	1	1	1	1	1
Breast invasive carcinoma	TCGA-E2-A150	1	1	1	1	1
Breast invasive carcinoma	TCGA-E2-A152	1	1	1	1	1
Breast invasive carcinoma	TCGA-E2-A153	1	0	1	1	1
Breast invasive carcinoma	TCGA-E2-A154	1	1	0	1	1
Breast invasive carcinoma	TCGA-E2-A155	1	1	1	0	1
Breast invasive carcinoma	TCGA-E2-A156	1	1	0	1	1
Breast invasive carcinoma	TCGA-E2-A158	1	1	1	1	1
Breast invasive carcinoma	TCGA-E2-A159	1	1	1	1	1
Breast invasive carcinoma	TCGA-E2-A15A	1	1	1	1	1
Breast invasive carcinoma	TCGA-E2-A15C	1	1	0	1	1
Breast invasive carcinoma	TCGA-E2-A15D	0	0	0	0	0
Breast invasive carcinoma	TCGA-E2-A15E	1	1	1	1	1
Breast invasive carcinoma	TCGA-E2-A15F	1	1	1	1	1
Breast invasive carcinoma	TCGA-E2-A15G	1	1	1	1	1
Breast invasive carcinoma	TCGA-E2-A15H	1	1	1	1	1
Breast invasive carcinoma	TCGA-E2-A15I	0	0	1	1	1
Breast invasive carcinoma	TCGA-E2-A15J	0	1	0	0	0
Breast invasive carcinoma	TCGA-E2-A15K	1	1	1	0	1
Breast invasive carcinoma	TCGA-E2-A15L	1	0	0	0	0
Breast invasive carcinoma	TCGA-E2-A15M	1	1	1	1	1
Breast invasive carcinoma	TCGA-E2-A15O	1	1	0	1	1

Breast invasive carcinoma	TCGA-E2-A15P	1	0	1	1	1
Breast invasive carcinoma	TCGA-E2-A15R	1	1	1	1	1
Breast invasive carcinoma	TCGA-E2-A15S	1	1	1	1	1
Breast invasive carcinoma	TCGA-E2-A15T	0	0	1	0	1
Breast invasive carcinoma	TCGA-E2-A1AZ	1	1	1	1	1
Breast invasive carcinoma	TCGA-E2-A1B0	1	0	1	1	1
Breast invasive carcinoma	TCGA-E2-A1B1	1	1	1	1	1
Breast invasive carcinoma	TCGA-E2-A1B4	1	0	0	0	0
Breast invasive carcinoma	TCGA-E2-A1B5	0	1	1	1	1
Breast invasive carcinoma	TCGA-E2-A1B6	1	1	1	0	1
Breast invasive carcinoma	TCGA-E2-A1BC	1	1	1	1	1
Breast invasive carcinoma	TCGA-E2-A1BD	1	0	1	1	1
Breast invasive carcinoma	TCGA-E2-A1IE	1	1	1	0	1
Breast invasive carcinoma	TCGA-E2-A1IF	1	1	0	1	1
Breast invasive carcinoma	TCGA-E2-A1IG	0	1	1	0	1
Breast invasive carcinoma	TCGA-E2-A1IH	1	1	1	1	1
Breast invasive carcinoma	TCGA-E2-A1II	1	1	1	1	1
Breast invasive carcinoma	TCGA-E2-A1IJ	1	0	1	1	1
Breast invasive carcinoma	TCGA-E2-A1IK	0	0	0	0	0
Breast invasive carcinoma	TCGA-E2-A1IL	1	0	1	1	1
Breast invasive carcinoma	TCGA-E2-A1IN	1	1	1	1	1
Breast invasive carcinoma	TCGA-E2-A1IO	1	1	1	1	1
Breast invasive carcinoma	TCGA-E2-A1IU	0	1	0	1	1
Breast invasive carcinoma	TCGA-E2-A1L6	1	0	1	0	1
Breast invasive carcinoma	TCGA-E2-A1L7	1	1	1	1	1
Breast invasive carcinoma	TCGA-E2-A1L8	1	1	1	1	1
Breast invasive carcinoma	TCGA-E2-A1L9	0	0	0	0	0
Breast invasive carcinoma	TCGA-E2-A1LA	1	1	1	1	1
Breast invasive carcinoma	TCGA-E2-A1LB	1	1	1	1	1
Breast invasive carcinoma	TCGA-E2-A1LE	1	1	1	1	1
Breast invasive carcinoma	TCGA-E2-A1LG	1	1	1	1	1
Breast invasive carcinoma	TCGA-E2-A1LH	1	1	1	1	1
Breast invasive carcinoma	TCGA-E2-A1LI	1	1	1	0	1
Breast invasive carcinoma	TCGA-E2-A1LK	1	1	1	1	1
Breast invasive carcinoma	TCGA-E2-A1LL	1	1	1	1	1
Breast invasive carcinoma	TCGA-E2-A1LS	0	0	0	0	0
Breast invasive carcinoma	TCGA-E2-A2P5	1	1	1	1	1
Breast invasive carcinoma	TCGA-E2-A2P6	1	1	1	1	1
Breast invasive carcinoma	TCGA-E2-A3DX	0	0	0	0	0
Breast invasive carcinoma	TCGA-E2-A56Z	1	0	1	1	1
Breast invasive carcinoma	TCGA-E2-A570	1	0	0	0	0
Breast invasive carcinoma	TCGA-E2-A572	0	0	0	0	0
Breast invasive carcinoma	TCGA-E2-A573	1	1	1	1	1
Breast invasive carcinoma	TCGA-E2-A574	1	1	1	1	1
Breast invasive carcinoma	TCGA-E2-A576	1	1	1	1	1
Breast invasive carcinoma	TCGA-E2-A9RU	1	1	1	1	1
Breast invasive carcinoma	TCGA-E9-A1N3	0	0	0	1	1
Breast invasive carcinoma	TCGA-E9-A1N4	1	0	1	0	1
Breast invasive carcinoma	TCGA-E9-A1N5	1	1	1	1	1
Breast invasive carcinoma	TCGA-E9-A1N6	1	1	1	1	1
Breast invasive carcinoma	TCGA-E9-A1N8	1	1	1	1	1
Breast invasive carcinoma	TCGA-E9-A1N9	1	1	1	1	1
Breast invasive carcinoma	TCGA-E9-A1NA	1	1	1	1	1
Breast invasive carcinoma	TCGA-E9-A1NC	1	1	1	1	1
Breast invasive carcinoma	TCGA-E9-A1ND	1	1	1	1	1
Breast invasive carcinoma	TCGA-E9-A1NE	0	1	1	0	1
Breast invasive carcinoma	TCGA-E9-A1NF	1	1	1	1	1
Breast invasive carcinoma	TCGA-E9-A1NG	1	1	1	1	1

Breast invasive carcinoma	TCGA-E9-A1NH	1	1	1	1	1
Breast invasive carcinoma	TCGA-E9-A1NI	1	1	1	1	1
Breast invasive carcinoma	TCGA-E9-A1QZ	1	1	1	1	1
Breast invasive carcinoma	TCGA-E9-A1R0	1	1	1	1	1
Breast invasive carcinoma	TCGA-E9-A1R2	1	1	1	0	1
Breast invasive carcinoma	TCGA-E9-A1R3	1	1	1	1	1
Breast invasive carcinoma	TCGA-E9-A1R4	1	1	1	1	1
Breast invasive carcinoma	TCGA-E9-A1R5	1	1	0	1	0
Breast invasive carcinoma	TCGA-E9-A1R6	1	1	1	1	1
Breast invasive carcinoma	TCGA-E9-A1R7	1	1	1	1	1
Breast invasive carcinoma	TCGA-E9-A1RA	1	1	1	1	1
Breast invasive carcinoma	TCGA-E9-A1RB	1	1	1	1	1
Breast invasive carcinoma	TCGA-E9-A1RC	1	1	1	1	1
Breast invasive carcinoma	TCGA-E9-A1RD	1	0	0	1	1
Breast invasive carcinoma	TCGA-E9-A1RE	1	1	1	1	1
Breast invasive carcinoma	TCGA-E9-A1RF	1	1	1	1	1
Breast invasive carcinoma	TCGA-E9-A1RG	1	1	1	1	1
Breast invasive carcinoma	TCGA-E9-A1RH	1	1	1	1	1
Breast invasive carcinoma	TCGA-E9-A1RI	1	0	1	1	1
Breast invasive carcinoma	TCGA-E9-A226	1	1	1	1	1
Breast invasive carcinoma	TCGA-E9-A227	1	0	1	0	0
Breast invasive carcinoma	TCGA-E9-A228	0	1	1	1	1
Breast invasive carcinoma	TCGA-E9-A229	1	0	1	1	1
Breast invasive carcinoma	TCGA-E9-A22A	1	1	1	1	1
Breast invasive carcinoma	TCGA-E9-A22B	1	1	1	1	1
Breast invasive carcinoma	TCGA-E9-A22D	1	1	1	1	1
Breast invasive carcinoma	TCGA-E9-A22E	1	1	1	1	1
Breast invasive carcinoma	TCGA-E9-A22G	1	1	1	1	1
Breast invasive carcinoma	TCGA-E9-A22H	0	0	0	1	1
Breast invasive carcinoma	TCGA-E9-A243	1	1	1	1	1
Breast invasive carcinoma	TCGA-E9-A244	1	1	1	1	1
Breast invasive carcinoma	TCGA-E9-A245	0	0	0	0	0
Breast invasive carcinoma	TCGA-E9-A247	1	1	1	1	1
Breast invasive carcinoma	TCGA-E9-A248	1	1	1	1	1
Breast invasive carcinoma	TCGA-E9-A249	1	1	1	1	1
Breast invasive carcinoma	TCGA-E9-A24A	0	0	0	1	1
Breast invasive carcinoma	TCGA-E9-A295	1	1	1	1	1
Breast invasive carcinoma	TCGA-E9-A2JS	1	1	1	1	1
Breast invasive carcinoma	TCGA-E9-A2JT	0	1	0	0	0
Breast invasive carcinoma	TCGA-E9-A3HO	1	1	1	1	1
Breast invasive carcinoma	TCGA-E9-A3Q9	0	0	0	0	0
Breast invasive carcinoma	TCGA-E9-A3QA	1	1	1	1	1
Breast invasive carcinoma	TCGA-E9-A3X8	1	1	1	1	1
Breast invasive carcinoma	TCGA-E9-A54X	0	1	1	1	1
Breast invasive carcinoma	TCGA-E9-A54Y	1	1	1	1	1
Breast invasive carcinoma	TCGA-E9-A5FK	1	1	0	1	1
Breast invasive carcinoma	TCGA-E9-A5FL	1	1	1	1	1
Breast invasive carcinoma	TCGA-E9-A5UO	1	1	1	1	1
Breast invasive carcinoma	TCGA-E9-A5UP	1	1	1	1	1
Breast invasive carcinoma	TCGA-E9-A6HE	1	0	1	1	1
Breast invasive carcinoma	TCGA-EW-A1IW	1	1	1	1	1
Breast invasive carcinoma	TCGA-EW-A1IX	1	1	1	1	1
Breast invasive carcinoma	TCGA-EW-A1IY	1	1	0	1	0
Breast invasive carcinoma	TCGA-EW-A1IZ	1	0	0	0	0
Breast invasive carcinoma	TCGA-EW-A1J1	1	1	1	1	1
Breast invasive carcinoma	TCGA-EW-A1J2	1	0	1	1	1
Breast invasive carcinoma	TCGA-EW-A1J3	1	0	1	0	1
Breast invasive carcinoma	TCGA-EW-A1J5	1	1	1	0	1

Breast invasive carcinoma	TCGA-EW-A1J6	1	1	1	1	1
Breast invasive carcinoma	TCGA-EW-A1OV	1	1	1	1	1
Breast invasive carcinoma	TCGA-EW-A1OW	1	1	1	1	1
Breast invasive carcinoma	TCGA-EW-A1OX	1	1	1	1	1
Breast invasive carcinoma	TCGA-EW-A1OY	1	1	1	1	1
Breast invasive carcinoma	TCGA-EW-A1OZ	1	1	1	1	1
Breast invasive carcinoma	TCGA-EW-A1P0	1	0	1	0	0
Breast invasive carcinoma	TCGA-EW-A1P1	1	0	0	1	1
Breast invasive carcinoma	TCGA-EW-A1P3	1	1	1	1	1
Breast invasive carcinoma	TCGA-EW-A1P4	1	1	1	1	1
Breast invasive carcinoma	TCGA-EW-A1P5	1	1	1	1	1
Breast invasive carcinoma	TCGA-EW-A1P6	0	1	0	1	1
Breast invasive carcinoma	TCGA-EW-A1P7	0	0	0	0	0
Breast invasive carcinoma	TCGA-EW-A1P8	1	1	1	1	1
Breast invasive carcinoma	TCGA-EW-A1PA	1	1	1	1	1
Breast invasive carcinoma	TCGA-EW-A1PB	1	0	0	1	0
Breast invasive carcinoma	TCGA-EW-A1PC	1	1	1	1	1
Breast invasive carcinoma	TCGA-EW-A1PD	1	1	1	1	1
Breast invasive carcinoma	TCGA-EW-A1PE	1	1	1	1	1
Breast invasive carcinoma	TCGA-EW-A1PF	1	1	1	1	1
Breast invasive carcinoma	TCGA-EW-A1PG	0	0	0	0	0
Breast invasive carcinoma	TCGA-EW-A1PH	1	1	1	1	1
Breast invasive carcinoma	TCGA-EW-A2FR	1	1	1	1	1
Breast invasive carcinoma	TCGA-EW-A2FS	1	1	1	1	1
Breast invasive carcinoma	TCGA-EW-A2FV	1	1	1	1	1
Breast invasive carcinoma	TCGA-EW-A2FW	0	1	1	1	1
Breast invasive carcinoma	TCGA-EW-A3E8	1	0	1	1	1
Breast invasive carcinoma	TCGA-EW-A3U0	1	1	1	1	1
Breast invasive carcinoma	TCGA-EW-A423	1	1	1	1	1
Breast invasive carcinoma	TCGA-EW-A424	1	1	0	1	1
Breast invasive carcinoma	TCGA-EW-A6S9	1	1	1	1	1
Breast invasive carcinoma	TCGA-EW-A6SA	1	1	1	1	1
Breast invasive carcinoma	TCGA-EW-A6SB	1	1	1	1	1
Breast invasive carcinoma	TCGA-EW-A6SC	1	0	1	1	1
Breast invasive carcinoma	TCGA-EW-A6SD	1	1	1	1	1
Breast invasive carcinoma	TCGA-GI-A2C8	1	1	1	1	1
Breast invasive carcinoma	TCGA-GI-A2C9	1	1	1	1	1
Breast invasive carcinoma	TCGA-GM-A2D9	1	1	1	1	1
Breast invasive carcinoma	TCGA-GM-A2DA	0	1	1	1	1
Breast invasive carcinoma	TCGA-GM-A2DB	1	1	1	1	1
Breast invasive carcinoma	TCGA-GM-A2DC	1	0	1	1	1
Breast invasive carcinoma	TCGA-GM-A2DD	1	1	1	1	1
Breast invasive carcinoma	TCGA-GM-A2DF	1	1	1	0	1
Breast invasive carcinoma	TCGA-GM-A2DH	1	1	1	1	1
Breast invasive carcinoma	TCGA-GM-A2DI	1	1	1	1	1
Breast invasive carcinoma	TCGA-GM-A2DK	1	0	1	1	1
Breast invasive carcinoma	TCGA-GM-A2DL	1	0	1	1	1
Breast invasive carcinoma	TCGA-GM-A2DM	1	0	0	0	0
Breast invasive carcinoma	TCGA-GM-A2DN	0	0	0	0	0
Breast invasive carcinoma	TCGA-GM-A2DO	0	1	0	0	1
Breast invasive carcinoma	TCGA-GM-A3NW	1	1	0	1	1
Breast invasive carcinoma	TCGA-GM-A3NY	1	1	0	1	1
Breast invasive carcinoma	TCGA-GM-A3XG	0	0	0	0	0
Breast invasive carcinoma	TCGA-GM-A3XL	1	1	1	1	1
Breast invasive carcinoma	TCGA-GM-A3XN	1	0	1	1	1
Breast invasive carcinoma	TCGA-GM-A4E0	1	1	1	1	1
Breast invasive carcinoma	TCGA-GM-A5PV	1	1	0	0	0
Breast invasive carcinoma	TCGA-GM-A5PX	0	0	0	0	0

Breast invasive carcinoma	TCGA-HN-A2NL	1	1	1	1	1
Breast invasive carcinoma	TCGA-HN-A2OB	1	1	1	0	1
Breast invasive carcinoma	TCGA-JL-A3YW	1	1	1	1	1
Breast invasive carcinoma	TCGA-JL-A3YX	1	1	0	1	1
Breast invasive carcinoma	TCGA-LD-A66U	0	0	1	1	1
Breast invasive carcinoma	TCGA-LD-A74U	1	1	1	0	1
Breast invasive carcinoma	TCGA-LD-A7W5	1	1	1	1	1
Breast invasive carcinoma	TCGA-LD-A7W6	1	1	1	0	1
Breast invasive carcinoma	TCGA-LD-A9QF	1	1	1	0	1
Breast invasive carcinoma	TCGA-LL-A440	0	1	0	0	0
Breast invasive carcinoma	TCGA-LL-A441	1	1	1	1	1
Breast invasive carcinoma	TCGA-LL-A442	0	0	0	1	1
Breast invasive carcinoma	TCGA-LL-A50Y	1	1	0	0	0
Breast invasive carcinoma	TCGA-LL-A5YL	1	1	0	1	1
Breast invasive carcinoma	TCGA-LL-A5YM	1	1	1	1	1
Breast invasive carcinoma	TCGA-LL-A5YN	1	1	1	1	1
Breast invasive carcinoma	TCGA-LL-A5YO	1	1	1	1	1
Breast invasive carcinoma	TCGA-LL-A5YP	1	1	1	1	1
Breast invasive carcinoma	TCGA-LL-A6FP	1	1	1	1	1
Breast invasive carcinoma	TCGA-LL-A6FQ	1	1	0	0	1
Breast invasive carcinoma	TCGA-LL-A6FR	1	1	1	0	1
Breast invasive carcinoma	TCGA-LL-A73Y	0	1	0	0	1
Breast invasive carcinoma	TCGA-LL-A73Z	1	1	1	1	1
Breast invasive carcinoma	TCGA-LL-A740	0	0	1	0	1
Breast invasive carcinoma	TCGA-LL-A7SZ	1	1	1	1	1
Breast invasive carcinoma	TCGA-LL-A7T0	1	1	1	1	1
Breast invasive carcinoma	TCGA-LL-A8F5	1	1	1	1	1
Breast invasive carcinoma	TCGA-LL-A9Q3	1	1	1	1	1
Breast invasive carcinoma	TCGA-LQ-A4E4	1	1	1	1	1
Breast invasive carcinoma	TCGA-MS-A51U	1	0	1	0	1
Breast invasive carcinoma	TCGA-OK-A5Q2	1	1	0	1	1
Breast invasive carcinoma	TCGA-OL-A5D6	1	1	1	0	1
Breast invasive carcinoma	TCGA-OL-A5D7	1	1	1	1	1
Breast invasive carcinoma	TCGA-OL-A5D8	1	1	1	1	1
Breast invasive carcinoma	TCGA-OL-A5DA	1	1	1	0	1
Breast invasive carcinoma	TCGA-OL-A5RU	1	0	1	0	0
Breast invasive carcinoma	TCGA-OL-A5RV	0	0	0	1	1
Breast invasive carcinoma	TCGA-OL-A5RW	1	1	1	1	1
Breast invasive carcinoma	TCGA-OL-A5RX	1	0	0	0	0
Breast invasive carcinoma	TCGA-OL-A5RY	1	1	1	1	1
Breast invasive carcinoma	TCGA-OL-A5RZ	1	1	1	1	1
Breast invasive carcinoma	TCGA-OL-A5S0	1	1	1	1	1
Breast invasive carcinoma	TCGA-OL-A66H	0	0	0	0	1
Breast invasive carcinoma	TCGA-OL-A66I	1	1	1	1	1
Breast invasive carcinoma	TCGA-OL-A66J	1	1	1	1	1
Breast invasive carcinoma	TCGA-OL-A66K	1	1	1	1	1
Breast invasive carcinoma	TCGA-OL-A66L	1	1	1	1	1
Breast invasive carcinoma	TCGA-OL-A66N	1	1	0	1	1
Breast invasive carcinoma	TCGA-OL-A66O	1	1	1	0	1
Breast invasive carcinoma	TCGA-OL-A66P	1	1	0	1	1
Breast invasive carcinoma	TCGA-OL-A6VO	1	1	1	1	1
Breast invasive carcinoma	TCGA-OL-A6VQ	1	0	1	1	1
Breast invasive carcinoma	TCGA-OL-A6VR	1	0	1	1	1
Breast invasive carcinoma	TCGA-OL-A97C	0	1	0	0	1
Breast invasive carcinoma	TCGA-PE-A5DC	1	1	1	1	1
Breast invasive carcinoma	TCGA-PE-A5DD	1	1	1	1	1
Breast invasive carcinoma	TCGA-PE-A5DE	1	1	1	1	1
Breast invasive carcinoma	TCGA-PL-A8LV	1	1	1	1	1

Breast invasive carcinoma	TCGA-PL-A8LX	1	1	1	1	1
Breast invasive carcinoma	TCGA-PL-A8LY	0	0	0	0	0
Breast invasive carcinoma	TCGA-PL-A8LZ	1	1	1	1	1
Breast invasive carcinoma	TCGA-S3-A6ZF	1	1	1	1	1
Breast invasive carcinoma	TCGA-S3-A6ZG	1	1	1	1	1
Breast invasive carcinoma	TCGA-S3-A6ZH	1	1	1	1	1
Breast invasive carcinoma	TCGA-S3-AA0Z	0	1	0	1	1
Breast invasive carcinoma	TCGA-S3-AA10	1	1	1	1	1
Breast invasive carcinoma	TCGA-S3-AA11	1	0	0	0	0
Breast invasive carcinoma	TCGA-S3-AA12	0	0	1	0	1
Breast invasive carcinoma	TCGA-S3-AA14	1	1	1	0	1
Breast invasive carcinoma	TCGA-S3-AA15	0	0	0	0	0
Breast invasive carcinoma	TCGA-S3-AA17	0	1	1	1	1
Breast invasive carcinoma	TCGA-UL-AAZ6	1	1	1	1	1
Breast invasive carcinoma	TCGA-UU-A93S	1	1	1	1	1
Breast invasive carcinoma	TCGA-V7-A7HQ	1	1	1	1	1
Breast invasive carcinoma	TCGA-W8-A86G	0	0	1	0	1
Breast invasive carcinoma	TCGA-WT-AB41	1	1	1	1	1
Breast invasive carcinoma	TCGA-WT-AB44	1	1	1	1	1
Breast invasive carcinoma	TCGA-XX-A899	1	1	0	0	1
Breast invasive carcinoma	TCGA-XX-A89A	1	1	1	1	1
Breast invasive carcinoma	TCGA-Z7-A8R5	1	1	0	1	1
Breast invasive carcinoma	TCGA-Z7-A8R6	1	1	1	0	1
Brian Lower grade Glioma	TCGA-CS-4938	1	0	0	0	1
Brian Lower grade Glioma	TCGA-CS-4941	1	0	0	0	1
Brian Lower grade Glioma	TCGA-CS-4942	1	1	0	0	1
Brian Lower grade Glioma	TCGA-CS-4943	1	1	1	1	1
Brian Lower grade Glioma	TCGA-CS-4944	1	1	1	1	1
Brian Lower grade Glioma	TCGA-CS-5390	1	1	0	1	1
Brian Lower grade Glioma	TCGA-CS-5393	0	1	0	0	1
Brian Lower grade Glioma	TCGA-CS-5394	1	1	0	1	1
Brian Lower grade Glioma	TCGA-CS-5395	1	1	0	0	1
Brian Lower grade Glioma	TCGA-CS-5396	1	1	0	1	1
Brian Lower grade Glioma	TCGA-CS-5397	1	1	1	0	1
Brian Lower grade Glioma	TCGA-CS-6186	1	1	1	1	1
Brian Lower grade Glioma	TCGA-CS-6188	1	1	0	1	1
Brian Lower grade Glioma	TCGA-CS-6290	1	0	0	1	1
Brian Lower grade Glioma	TCGA-CS-6665	1	1	1	1	1
Brian Lower grade Glioma	TCGA-CS-6666	1	1	1	0	1
Brian Lower grade Glioma	TCGA-CS-6667	0	1	0	0	1
Brian Lower grade Glioma	TCGA-CS-6668	1	1	0	1	1
Brian Lower grade Glioma	TCGA-CS-6669	0	0	0	0	0
Brian Lower grade Glioma	TCGA-CS-6670	1	1	0	1	1
Brian Lower grade Glioma	TCGA-DB-5270	1	1	0	0	0
Brian Lower grade Glioma	TCGA-DB-5273	0	0	0	0	1
Brian Lower grade Glioma	TCGA-DB-5274	1	1	0	1	1
Brian Lower grade Glioma	TCGA-DB-5275	1	1	1	1	1
Brian Lower grade Glioma	TCGA-DB-5276	1	1	1	0	1
Brian Lower grade Glioma	TCGA-DB-5277	1	1	1	1	1
Brian Lower grade Glioma	TCGA-DB-5278	1	1	0	1	1
Brian Lower grade Glioma	TCGA-DB-5279	1	1	1	1	1
Brian Lower grade Glioma	TCGA-DB-5280	0	0	1	0	1
Brian Lower grade Glioma	TCGA-DB-5281	1	1	1	1	1
Brian Lower grade Glioma	TCGA-DB-A4X9	0	0	0	0	1
Brian Lower grade Glioma	TCGA-DB-A4XA	1	1	0	1	1
Brian Lower grade Glioma	TCGA-DB-A4XB	1	1	0	1	1
Brian Lower grade Glioma	TCGA-DB-A4XC	0	1	0	0	1
Brian Lower grade Glioma	TCGA-DB-A4XD	1	0	0	0	1

Brian Lower grade Glioma	TCGA-DB-A4XE	1	1	1	0	1
Brian Lower grade Glioma	TCGA-DB-A4XF	1	1	1	0	1
Brian Lower grade Glioma	TCGA-DB-A4XG	1	1	0	1	1
Brian Lower grade Glioma	TCGA-DB-A4XH	1	1	1	1	1
Brian Lower grade Glioma	TCGA-DB-A64L	1	1	0	1	1
Brian Lower grade Glioma	TCGA-DB-A64O	1	1	1	1	1
Brian Lower grade Glioma	TCGA-DB-A64P	1	1	0	1	1
Brian Lower grade Glioma	TCGA-DB-A64Q	1	1	0	1	1
Brian Lower grade Glioma	TCGA-DB-A64R	1	1	0	1	1
Brian Lower grade Glioma	TCGA-DB-A64S	0	0	0	0	1
Brian Lower grade Glioma	TCGA-DB-A64U	1	1	1	1	1
Brian Lower grade Glioma	TCGA-DB-A64V	1	1	0	1	1
Brian Lower grade Glioma	TCGA-DB-A64W	1	1	0	1	1
Brian Lower grade Glioma	TCGA-DB-A64X	1	1	1	1	1
Brian Lower grade Glioma	TCGA-DB-A75K	1	1	0	1	1
Brian Lower grade Glioma	TCGA-DB-A75L	1	1	0	0	1
Brian Lower grade Glioma	TCGA-DB-A75M	0	0	0	0	0
Brian Lower grade Glioma	TCGA-DB-A75O	1	0	0	0	1
Brian Lower grade Glioma	TCGA-DB-A75P	1	1	0	0	1
Brian Lower grade Glioma	TCGA-DH-5140	1	1	1	1	1
Brian Lower grade Glioma	TCGA-DH-5141	1	1	1	1	1
Brian Lower grade Glioma	TCGA-DH-5142	1	1	1	1	1
Brian Lower grade Glioma	TCGA-DH-5143	1	0	0	0	1
Brian Lower grade Glioma	TCGA-DH-5144	1	1	0	1	1
Brian Lower grade Glioma	TCGA-DH-A669	1	1	1	1	1
Brian Lower grade Glioma	TCGA-DH-A66B	1	1	1	1	1
Brian Lower grade Glioma	TCGA-DH-A66D	1	1	1	1	1
Brian Lower grade Glioma	TCGA-DH-A66F	1	1	0	1	1
Brian Lower grade Glioma	TCGA-DH-A66G	1	1	1	0	1
Brian Lower grade Glioma	TCGA-DH-A7UR	1	1	1	1	1
Brian Lower grade Glioma	TCGA-DH-A7US	1	1	0	1	1
Brian Lower grade Glioma	TCGA-DH-A7UT	1	0	0	1	1
Brian Lower grade Glioma	TCGA-DH-A7UU	1	0	0	0	1
Brian Lower grade Glioma	TCGA-DH-A7UV	0	0	0	0	1
Brian Lower grade Glioma	TCGA-DU-5847	1	1	1	1	1
Brian Lower grade Glioma	TCGA-DU-5849	1	1	0	1	1
Brian Lower grade Glioma	TCGA-DU-5851	1	1	0	0	1
Brian Lower grade Glioma	TCGA-DU-5852	1	0	0	1	1
Brian Lower grade Glioma	TCGA-DU-5853	0	0	1	1	1
Brian Lower grade Glioma	TCGA-DU-5854	1	1	0	0	1
Brian Lower grade Glioma	TCGA-DU-5855	1	1	1	1	1
Brian Lower grade Glioma	TCGA-DU-5870	1	1	0	1	1
Brian Lower grade Glioma	TCGA-DU-5871	1	0	0	0	1
Brian Lower grade Glioma	TCGA-DU-5872	1	1	1	1	1
Brian Lower grade Glioma	TCGA-DU-5874	1	1	0	1	1
Brian Lower grade Glioma	TCGA-DU-6392	0	0	0	0	0
Brian Lower grade Glioma	TCGA-DU-6393	1	1	0	1	1
Brian Lower grade Glioma	TCGA-DU-6394	1	1	0	1	1
Brian Lower grade Glioma	TCGA-DU-6395	1	1	1	0	1
Brian Lower grade Glioma	TCGA-DU-6396	1	1	0	1	1
Brian Lower grade Glioma	TCGA-DU-6397	1	1	0	1	1
Brian Lower grade Glioma	TCGA-DU-6399	1	1	1	1	1
Brian Lower grade Glioma	TCGA-DU-6400	1	1	1	1	1
Brian Lower grade Glioma	TCGA-DU-6401	1	0	0	0	1
Brian Lower grade Glioma	TCGA-DU-6402	1	1	0	1	1
Brian Lower grade Glioma	TCGA-DU-6403	1	1	1	0	1
Brian Lower grade Glioma	TCGA-DU-6404	0	0	0	0	0
Brian Lower grade Glioma	TCGA-DU-6405	1	1	1	0	1

Brian Lower grade Glioma	TCGA-DU-6406	1	0	0	0	1
Brian Lower grade Glioma	TCGA-DU-6407	1	1	0	1	1
Brian Lower grade Glioma	TCGA-DU-6408	1	1	1	1	1
Brian Lower grade Glioma	TCGA-DU-6410	1	1	0	1	1
Brian Lower grade Glioma	TCGA-DU-6542	1	1	1	1	1
Brian Lower grade Glioma	TCGA-DU-7006	1	1	1	1	1
Brian Lower grade Glioma	TCGA-DU-7007	1	1	1	1	1
Brian Lower grade Glioma	TCGA-DU-7008	1	0	0	1	1
Brian Lower grade Glioma	TCGA-DU-7009	1	1	0	1	1
Brian Lower grade Glioma	TCGA-DU-7010	1	1	1	1	1
Brian Lower grade Glioma	TCGA-DU-7011	0	0	0	0	0
Brian Lower grade Glioma	TCGA-DU-7012	1	0	1	0	1
Brian Lower grade Glioma	TCGA-DU-7013	1	0	0	0	1
Brian Lower grade Glioma	TCGA-DU-7014	1	1	1	1	1
Brian Lower grade Glioma	TCGA-DU-7015	1	1	1	1	1
Brian Lower grade Glioma	TCGA-DU-7018	1	1	0	1	1
Brian Lower grade Glioma	TCGA-DU-7019	0	1	0	0	1
Brian Lower grade Glioma	TCGA-DU-7290	1	1	0	1	1
Brian Lower grade Glioma	TCGA-DU-7292	1	1	0	0	1
Brian Lower grade Glioma	TCGA-DU-7294	1	1	0	1	1
Brian Lower grade Glioma	TCGA-DU-7298	1	1	1	1	1
Brian Lower grade Glioma	TCGA-DU-7299	1	0	1	0	1
Brian Lower grade Glioma	TCGA-DU-7300	1	1	0	1	1
Brian Lower grade Glioma	TCGA-DU-7301	0	1	0	1	1
Brian Lower grade Glioma	TCGA-DU-7302	1	1	0	1	1
Brian Lower grade Glioma	TCGA-DU-7304	1	1	1	0	1
Brian Lower grade Glioma	TCGA-DU-7306	1	1	1	0	1
Brian Lower grade Glioma	TCGA-DU-7309	1	0	0	1	1
Brian Lower grade Glioma	TCGA-DU-8158	1	1	1	1	1
Brian Lower grade Glioma	TCGA-DU-8161	1	1	1	0	1
Brian Lower grade Glioma	TCGA-DU-8162	1	1	0	0	1
Brian Lower grade Glioma	TCGA-DU-8163	0	0	0	0	1
Brian Lower grade Glioma	TCGA-DU-8164	1	1	1	1	1
Brian Lower grade Glioma	TCGA-DU-8165	1	1	1	1	1
Brian Lower grade Glioma	TCGA-DU-8166	0	1	0	0	1
Brian Lower grade Glioma	TCGA-DU-8167	1	1	0	1	1
Brian Lower grade Glioma	TCGA-DU-8168	1	1	0	1	1
Brian Lower grade Glioma	TCGA-DU-A5TP	1	1	1	0	1
Brian Lower grade Glioma	TCGA-DU-A5TR	0	0	1	0	1
Brian Lower grade Glioma	TCGA-DU-A5TS	1	1	1	0	1
Brian Lower grade Glioma	TCGA-DU-A5TT	1	0	1	1	1
Brian Lower grade Glioma	TCGA-DU-A5TU	1	1	0	1	1
Brian Lower grade Glioma	TCGA-DU-A5TW	0	0	0	0	1
Brian Lower grade Glioma	TCGA-DU-A5TY	1	1	0	1	1
Brian Lower grade Glioma	TCGA-DU-A6S2	1	1	0	1	1
Brian Lower grade Glioma	TCGA-DU-A6S3	1	1	0	1	1
Brian Lower grade Glioma	TCGA-DU-A6S6	1	1	1	1	1
Brian Lower grade Glioma	TCGA-DU-A6S7	1	1	0	0	1
Brian Lower grade Glioma	TCGA-DU-A6S8	1	1	0	1	1
Brian Lower grade Glioma	TCGA-DU-A76K	1	1	0	1	1
Brian Lower grade Glioma	TCGA-DU-A76L	1	0	0	1	1
Brian Lower grade Glioma	TCGA-DU-A76O	1	0	0	0	0
Brian Lower grade Glioma	TCGA-DU-A76R	1	1	1	1	1
Brian Lower grade Glioma	TCGA-DU-A7T6	1	1	1	1	1
Brian Lower grade Glioma	TCGA-DU-A7T8	1	1	0	0	1
Brian Lower grade Glioma	TCGA-DU-A7TA	1	1	0	0	1
Brian Lower grade Glioma	TCGA-DU-A7TB	0	1	0	0	1
Brian Lower grade Glioma	TCGA-DU-A7TC	1	1	1	1	1

Brian Lower grade Glioma	TCGA-DU-A7TD	1	1	0	0	1
Brian Lower grade Glioma	TCGA-DU-A7TG	1	1	0	1	1
Brian Lower grade Glioma	TCGA-DU-A7TI	1	0	0	1	1
Brian Lower grade Glioma	TCGA-DU-A7TJ	1	1	0	0	1
Brian Lower grade Glioma	TCGA-E1-5302	1	1	1	0	1
Brian Lower grade Glioma	TCGA-E1-5303	1	0	0	1	1
Brian Lower grade Glioma	TCGA-E1-5304	1	1	1	1	1
Brian Lower grade Glioma	TCGA-E1-5305	1	1	1	1	1
Brian Lower grade Glioma	TCGA-E1-5307	1	1	1	1	1
Brian Lower grade Glioma	TCGA-E1-5311	1	1	0	1	1
Brian Lower grade Glioma	TCGA-E1-5318	1	1	1	1	1
Brian Lower grade Glioma	TCGA-E1-5319	1	1	0	1	1
Brian Lower grade Glioma	TCGA-E1-5322	1	1	0	1	1
Brian Lower grade Glioma	TCGA-E1-A7YD	1	1	0	1	1
Brian Lower grade Glioma	TCGA-E1-A7YE	1	1	1	1	1
Brian Lower grade Glioma	TCGA-E1-A7YH	1	0	1	0	1
Brian Lower grade Glioma	TCGA-E1-A7YI	1	1	1	1	1
Brian Lower grade Glioma	TCGA-E1-A7YJ	1	1	0	1	1
Brian Lower grade Glioma	TCGA-E1-A7YK	1	1	1	1	1
Brian Lower grade Glioma	TCGA-E1-A7YL	1	0	1	0	1
Brian Lower grade Glioma	TCGA-E1-A7YM	1	1	0	1	1
Brian Lower grade Glioma	TCGA-E1-A7YN	1	1	0	0	1
Brian Lower grade Glioma	TCGA-E1-A7YO	1	1	1	1	1
Brian Lower grade Glioma	TCGA-E1-A7YQ	1	1	1	1	1
Brian Lower grade Glioma	TCGA-E1-A7YS	1	1	1	1	1
Brian Lower grade Glioma	TCGA-E1-A7YU	1	1	0	1	1
Brian Lower grade Glioma	TCGA-E1-A7YV	1	1	1	1	1
Brian Lower grade Glioma	TCGA-E1-A7YW	1	0	0	1	1
Brian Lower grade Glioma	TCGA-E1-A7YY	1	1	0	1	1
Brian Lower grade Glioma	TCGA-E1-A7Z2	1	1	1	1	1
Brian Lower grade Glioma	TCGA-E1-A7Z3	0	1	0	0	1
Brian Lower grade Glioma	TCGA-E1-A7Z4	1	1	1	0	1
Brian Lower grade Glioma	TCGA-E1-A7Z6	1	0	0	1	0
Brian Lower grade Glioma	TCGA-EZ-7264	1	1	0	1	1
Brian Lower grade Glioma	TCGA-F6-A8O3	1	1	0	1	1
Brian Lower grade Glioma	TCGA-F6-A8O4	1	1	0	0	1
Brian Lower grade Glioma	TCGA-FG-5962	1	1	0	1	1
Brian Lower grade Glioma	TCGA-FG-5963	1	1	1	0	1
Brian Lower grade Glioma	TCGA-FG-5964	1	1	0	1	1
Brian Lower grade Glioma	TCGA-FG-5965	1	1	1	0	1
Brian Lower grade Glioma	TCGA-FG-6688	1	0	0	0	1
Brian Lower grade Glioma	TCGA-FG-6689	0	1	1	0	1
Brian Lower grade Glioma	TCGA-FG-6690	1	1	1	0	1
Brian Lower grade Glioma	TCGA-FG-6691	0	0	0	0	1
Brian Lower grade Glioma	TCGA-FG-6692	1	1	0	0	1
Brian Lower grade Glioma	TCGA-FG-7634	1	1	0	1	1
Brian Lower grade Glioma	TCGA-FG-7636	1	1	1	1	1
Brian Lower grade Glioma	TCGA-FG-7637	0	0	0	0	0
Brian Lower grade Glioma	TCGA-FG-7638	1	1	1	1	1
Brian Lower grade Glioma	TCGA-FG-7641	1	1	0	1	1
Brian Lower grade Glioma	TCGA-FG-7643	1	0	0	1	1
Brian Lower grade Glioma	TCGA-FG-8181	0	0	0	0	0
Brian Lower grade Glioma	TCGA-FG-8182	0	0	0	1	1
Brian Lower grade Glioma	TCGA-FG-8185	1	1	0	1	1
Brian Lower grade Glioma	TCGA-FG-8186	1	1	0	1	1
Brian Lower grade Glioma	TCGA-FG-8187	1	1	1	1	1
Brian Lower grade Glioma	TCGA-FG-8188	1	1	1	1	1
Brian Lower grade Glioma	TCGA-FG-8189	0	1	0	1	0

Brian Lower grade Glioma	TCGA-FG-8191	1	1	1	0	1
Brian Lower grade Glioma	TCGA-FG-A4MT	1	1	1	0	1
Brian Lower grade Glioma	TCGA-FG-A4MU	1	0	0	0	1
Brian Lower grade Glioma	TCGA-FG-A4MW	1	1	0	1	1
Brian Lower grade Glioma	TCGA-FG-A4MX	0	1	0	0	1
Brian Lower grade Glioma	TCGA-FG-A4MY	1	1	1	1	1
Brian Lower grade Glioma	TCGA-FG-A60J	1	1	1	0	1
Brian Lower grade Glioma	TCGA-FG-A60K	1	1	0	1	1
Brian Lower grade Glioma	TCGA-FG-A60L	0	0	0	0	1
Brian Lower grade Glioma	TCGA-FG-A6IZ	1	1	0	1	1
Brian Lower grade Glioma	TCGA-FG-A6J1	1	1	0	1	1
Brian Lower grade Glioma	TCGA-FG-A6J3	1	1	1	1	1
Brian Lower grade Glioma	TCGA-FG-A70Y	1	1	0	1	1
Brian Lower grade Glioma	TCGA-FG-A70Z	1	0	0	0	1
Brian Lower grade Glioma	TCGA-FG-A710	1	1	1	1	1
Brian Lower grade Glioma	TCGA-FG-A711	1	1	1	1	1
Brian Lower grade Glioma	TCGA-FG-A713	1	1	0	1	1
Brian Lower grade Glioma	TCGA-FG-A87N	1	1	0	0	1
Brian Lower grade Glioma	TCGA-FG-A87Q	1	1	1	1	1
Brian Lower grade Glioma	TCGA-FN-7833	0	0	0	0	1
Brian Lower grade Glioma	TCGA-HT-7467	1	1	0	1	1
Brian Lower grade Glioma	TCGA-HT-7468	1	1	0	1	1
Brian Lower grade Glioma	TCGA-HT-7469	1	1	0	1	1
Brian Lower grade Glioma	TCGA-HT-7470	1	1	1	0	1
Brian Lower grade Glioma	TCGA-HT-7471	1	1	0	1	1
Brian Lower grade Glioma	TCGA-HT-7472	1	1	0	1	1
Brian Lower grade Glioma	TCGA-HT-7473	0	0	0	0	1
Brian Lower grade Glioma	TCGA-HT-7474	0	1	1	0	1
Brian Lower grade Glioma	TCGA-HT-7475	1	1	1	1	1
Brian Lower grade Glioma	TCGA-HT-7476	1	1	0	0	1
Brian Lower grade Glioma	TCGA-HT-7477	1	1	1	1	1
Brian Lower grade Glioma	TCGA-HT-7478	1	1	1	1	1
Brian Lower grade Glioma	TCGA-HT-7479	0	0	0	0	1
Brian Lower grade Glioma	TCGA-HT-7480	1	1	1	1	1
Brian Lower grade Glioma	TCGA-HT-7481	1	1	0	1	1
Brian Lower grade Glioma	TCGA-HT-7482	0	0	0	0	1
Brian Lower grade Glioma	TCGA-HT-7483	0	0	0	0	1
Brian Lower grade Glioma	TCGA-HT-7485	0	0	0	0	1
Brian Lower grade Glioma	TCGA-HT-7601	0	1	0	0	1
Brian Lower grade Glioma	TCGA-HT-7602	0	0	0	0	1
Brian Lower grade Glioma	TCGA-HT-7603	1	0	0	0	1
Brian Lower grade Glioma	TCGA-HT-7604	1	1	0	0	1
Brian Lower grade Glioma	TCGA-HT-7605	1	1	0	1	1
Brian Lower grade Glioma	TCGA-HT-7606	1	1	1	1	1
Brian Lower grade Glioma	TCGA-HT-7607	1	1	0	1	1
Brian Lower grade Glioma	TCGA-HT-7608	1	1	1	1	1
Brian Lower grade Glioma	TCGA-HT-7609	0	0	0	1	1
Brian Lower grade Glioma	TCGA-HT-7610	0	1	0	0	1
Brian Lower grade Glioma	TCGA-HT-7611	0	0	0	0	1
Brian Lower grade Glioma	TCGA-HT-7616	1	1	0	1	1
Brian Lower grade Glioma	TCGA-HT-7620	1	1	0	1	1
Brian Lower grade Glioma	TCGA-HT-7676	1	0	0	1	1
Brian Lower grade Glioma	TCGA-HT-7677	1	1	0	1	1
Brian Lower grade Glioma	TCGA-HT-7680	0	0	0	0	0
Brian Lower grade Glioma	TCGA-HT-7681	1	1	1	1	1
Brian Lower grade Glioma	TCGA-HT-7684	0	0	0	0	0
Brian Lower grade Glioma	TCGA-HT-7686	0	0	0	1	1
Brian Lower grade Glioma	TCGA-HT-7687	1	1	0	1	1

Brian Lower grade Glioma	TCGA-HT-7688	1	1	1	1	1
Brian Lower grade Glioma	TCGA-HT-7689	1	1	1	0	1
Brian Lower grade Glioma	TCGA-HT-7690	1	0	1	1	1
Brian Lower grade Glioma	TCGA-HT-7691	0	0	0	1	0
Brian Lower grade Glioma	TCGA-HT-7692	1	1	1	1	1
Brian Lower grade Glioma	TCGA-HT-7693	0	0	0	0	1
Brian Lower grade Glioma	TCGA-HT-7694	1	1	0	1	1
Brian Lower grade Glioma	TCGA-HT-7695	1	1	0	1	1
Brian Lower grade Glioma	TCGA-HT-7854	0	0	0	0	0
Brian Lower grade Glioma	TCGA-HT-7855	0	1	1	0	1
Brian Lower grade Glioma	TCGA-HT-7856	1	1	0	1	1
Brian Lower grade Glioma	TCGA-HT-7857	1	1	1	1	1
Brian Lower grade Glioma	TCGA-HT-7858	0	0	0	0	1
Brian Lower grade Glioma	TCGA-HT-7860	1	0	0	1	1
Brian Lower grade Glioma	TCGA-HT-7873	1	0	1	0	1
Brian Lower grade Glioma	TCGA-HT-7874	1	1	0	1	1
Brian Lower grade Glioma	TCGA-HT-7875	1	1	0	1	1
Brian Lower grade Glioma	TCGA-HT-7877	1	1	0	1	1
Brian Lower grade Glioma	TCGA-HT-7879	1	0	1	0	1
Brian Lower grade Glioma	TCGA-HT-7880	0	0	0	0	1
Brian Lower grade Glioma	TCGA-HT-7881	1	1	0	1	1
Brian Lower grade Glioma	TCGA-HT-7882	1	0	1	0	1
Brian Lower grade Glioma	TCGA-HT-7884	1	1	1	0	1
Brian Lower grade Glioma	TCGA-HT-7902	1	1	0	0	1
Brian Lower grade Glioma	TCGA-HT-8010	1	1	0	1	1
Brian Lower grade Glioma	TCGA-HT-8011	1	1	0	0	1
Brian Lower grade Glioma	TCGA-HT-8012	1	1	0	1	1
Brian Lower grade Glioma	TCGA-HT-8013	0	0	0	0	1
Brian Lower grade Glioma	TCGA-HT-8015	0	0	0	1	0
Brian Lower grade Glioma	TCGA-HT-8018	1	0	1	0	1
Brian Lower grade Glioma	TCGA-HT-8019	0	0	0	0	0
Brian Lower grade Glioma	TCGA-HT-8104	1	1	0	0	1
Brian Lower grade Glioma	TCGA-HT-8105	1	1	0	1	1
Brian Lower grade Glioma	TCGA-HT-8106	1	1	1	0	1
Brian Lower grade Glioma	TCGA-HT-8107	0	0	0	0	0
Brian Lower grade Glioma	TCGA-HT-8108	1	1	1	0	1
Brian Lower grade Glioma	TCGA-HT-8109	1	1	0	1	1
Brian Lower grade Glioma	TCGA-HT-8110	1	0	0	0	1
Brian Lower grade Glioma	TCGA-HT-8111	1	1	1	1	1
Brian Lower grade Glioma	TCGA-HT-8113	1	1	0	1	0
Brian Lower grade Glioma	TCGA-HT-8114	0	0	0	1	1
Brian Lower grade Glioma	TCGA-HT-8558	0	0	0	0	0
Brian Lower grade Glioma	TCGA-HT-8563	1	1	0	0	1
Brian Lower grade Glioma	TCGA-HT-8564	1	1	1	1	1
Brian Lower grade Glioma	TCGA-HT-A4DS	1	1	1	1	1
Brian Lower grade Glioma	TCGA-HT-A4DV	1	1	0	1	1
Brian Lower grade Glioma	TCGA-HT-A5R5	0	0	0	0	1
Brian Lower grade Glioma	TCGA-HT-A5R7	1	1	1	1	1
Brian Lower grade Glioma	TCGA-HT-A5R9	1	1	0	1	1
Brian Lower grade Glioma	TCGA-HT-A5RA	1	1	1	0	1
Brian Lower grade Glioma	TCGA-HT-A5RB	1	1	1	0	1
Brian Lower grade Glioma	TCGA-HT-A5RC	1	1	0	0	1
Brian Lower grade Glioma	TCGA-HT-A614	1	1	1	1	1
Brian Lower grade Glioma	TCGA-HT-A615	1	1	0	1	1
Brian Lower grade Glioma	TCGA-HT-A616	1	0	0	1	0
Brian Lower grade Glioma	TCGA-HT-A617	1	1	1	1	1
Brian Lower grade Glioma	TCGA-HT-A618	1	1	1	1	1
Brian Lower grade Glioma	TCGA-HT-A619	1	1	1	1	1

Brian Lower grade Glioma	TCGA-HT-A61A	0	0	0	0	0
Brian Lower grade Glioma	TCGA-HT-A61B	1	1	1	1	1
Brian Lower grade Glioma	TCGA-HT-A61C	1	1	0	1	1
Brian Lower grade Glioma	TCGA-HT-A74H	1	0	1	0	1
Brian Lower grade Glioma	TCGA-HT-A74J	0	1	0	1	1
Brian Lower grade Glioma	TCGA-HT-A74K	1	1	0	1	1
Brian Lower grade Glioma	TCGA-HT-A74L	1	1	0	1	1
Brian Lower grade Glioma	TCGA-HT-A74O	0	0	0	0	0
Brian Lower grade Glioma	TCGA-HW-7486	1	1	0	1	1
Brian Lower grade Glioma	TCGA-HW-7487	1	1	0	1	1
Brian Lower grade Glioma	TCGA-HW-7489	1	0	0	0	1
Brian Lower grade Glioma	TCGA-HW-7490	0	1	0	0	1
Brian Lower grade Glioma	TCGA-HW-7491	1	1	0	1	1
Brian Lower grade Glioma	TCGA-HW-7493	0	0	1	0	1
Brian Lower grade Glioma	TCGA-HW-7495	1	1	0	1	1
Brian Lower grade Glioma	TCGA-HW-8319	0	1	0	0	1
Brian Lower grade Glioma	TCGA-HW-8320	1	1	0	0	1
Brian Lower grade Glioma	TCGA-HW-8321	1	0	0	0	1
Brian Lower grade Glioma	TCGA-HW-8322	1	1	0	1	1
Brian Lower grade Glioma	TCGA-HW-A5KJ	1	1	0	1	1
Brian Lower grade Glioma	TCGA-HW-A5KK	1	0	1	1	1
Brian Lower grade Glioma	TCGA-HW-A5KL	1	0	0	0	1
Brian Lower grade Glioma	TCGA-HW-A5KM	1	1	1	1	1
Brian Lower grade Glioma	TCGA-IK-7675	1	1	1	1	1
Brian Lower grade Glioma	TCGA-IK-8125	1	1	0	1	1
Brian Lower grade Glioma	TCGA-KT-A74X	1	1	0	1	1
Brian Lower grade Glioma	TCGA-KT-A7W1	1	1	0	0	1
Brian Lower grade Glioma	TCGA-P5-A5ET	1	1	0	1	1
Brian Lower grade Glioma	TCGA-P5-A5EU	1	1	1	1	1
Brian Lower grade Glioma	TCGA-P5-A5EV	1	1	1	0	1
Brian Lower grade Glioma	TCGA-P5-A5EW	1	0	1	0	1
Brian Lower grade Glioma	TCGA-P5-A5EX	1	1	0	1	1
Brian Lower grade Glioma	TCGA-P5-A5EY	0	0	0	0	0
Brian Lower grade Glioma	TCGA-P5-A5EZ	1	1	1	1	1
Brian Lower grade Glioma	TCGA-P5-A5F0	1	1	0	1	1
Brian Lower grade Glioma	TCGA-P5-A5F1	0	0	0	1	1
Brian Lower grade Glioma	TCGA-P5-A5F2	1	1	1	1	1
Brian Lower grade Glioma	TCGA-P5-A5F4	1	1	1	1	1
Brian Lower grade Glioma	TCGA-P5-A5F6	1	1	1	1	1
Brian Lower grade Glioma	TCGA-P5-A72U	1	1	0	1	1
Brian Lower grade Glioma	TCGA-P5-A72W	1	1	1	1	1
Brian Lower grade Glioma	TCGA-P5-A72X	1	0	1	0	0
Brian Lower grade Glioma	TCGA-P5-A72Z	1	1	0	1	1
Brian Lower grade Glioma	TCGA-P5-A730	1	1	0	1	1
Brian Lower grade Glioma	TCGA-P5-A731	0	1	0	1	1
Brian Lower grade Glioma	TCGA-P5-A733	1	1	1	1	1
Brian Lower grade Glioma	TCGA-P5-A735	1	0	0	0	1
Brian Lower grade Glioma	TCGA-P5-A736	0	0	0	0	0
Brian Lower grade Glioma	TCGA-P5-A737	1	1	0	1	1
Brian Lower grade Glioma	TCGA-P5-A77W	1	1	0	1	1
Brian Lower grade Glioma	TCGA-P5-A77X	1	1	0	1	1
Brian Lower grade Glioma	TCGA-P5-A780	1	1	1	1	1
Brian Lower grade Glioma	TCGA-P5-A781	1	1	0	1	1
Brian Lower grade Glioma	TCGA-QH-A65R	1	1	0	1	1
Brian Lower grade Glioma	TCGA-QH-A65S	1	1	1	1	1
Brian Lower grade Glioma	TCGA-QH-A65V	1	1	0	1	1
Brian Lower grade Glioma	TCGA-QH-A65X	1	1	0	1	1
Brian Lower grade Glioma	TCGA-QH-A65Z	1	1	0	1	1

Brian Lower grade Glioma	TCGA-QH-A6CS	1	0	1	1	1
Brian Lower grade Glioma	TCGA-QH-A6CU	1	1	1	1	1
Brian Lower grade Glioma	TCGA-QH-A6CV	1	1	0	1	1
Brian Lower grade Glioma	TCGA-QH-A6CW	1	0	1	1	1
Brian Lower grade Glioma	TCGA-QH-A6CX	1	1	1	0	1
Brian Lower grade Glioma	TCGA-QH-A6CY	1	1	0	1	1
Brian Lower grade Glioma	TCGA-QH-A6CZ	1	1	0	1	1
Brian Lower grade Glioma	TCGA-QH-A6X3	0	0	0	1	1
Brian Lower grade Glioma	TCGA-QH-A6X4	1	1	0	1	1
Brian Lower grade Glioma	TCGA-QH-A6X5	1	1	0	1	1
Brian Lower grade Glioma	TCGA-QH-A6X8	1	1	0	1	1
Brian Lower grade Glioma	TCGA-QH-A6X9	0	1	0	1	1
Brian Lower grade Glioma	TCGA-QH-A6XA	1	0	0	1	1
Brian Lower grade Glioma	TCGA-QH-A6XC	1	0	0	0	1
Brian Lower grade Glioma	TCGA-QH-A86X	1	1	0	1	1
Brian Lower grade Glioma	TCGA-QH-A870	1	1	1	1	1
Brian Lower grade Glioma	TCGA-R8-A6MK	1	1	0	1	1
Brian Lower grade Glioma	TCGA-R8-A6ML	1	1	0	1	1
Brian Lower grade Glioma	TCGA-R8-A6MO	1	1	0	1	1
Brian Lower grade Glioma	TCGA-R8-A6YH	1	1	1	0	1
Brian Lower grade Glioma	TCGA-R8-A73M	1	1	0	1	1
Brian Lower grade Glioma	TCGA-RY-A83X	1	1	0	1	1
Brian Lower grade Glioma	TCGA-RY-A83Y	1	1	0	1	1
Brian Lower grade Glioma	TCGA-RY-A83Z	1	1	1	1	1
Brian Lower grade Glioma	TCGA-RY-A840	1	1	0	1	1
Brian Lower grade Glioma	TCGA-RY-A843	0	0	0	0	0
Brian Lower grade Glioma	TCGA-RY-A845	0	0	0	0	0
Brian Lower grade Glioma	TCGA-RY-A847	1	1	0	1	1
Brian Lower grade Glioma	TCGA-S9-A6TS	1	1	1	1	1
Brian Lower grade Glioma	TCGA-S9-A6TU	0	0	0	0	1
Brian Lower grade Glioma	TCGA-S9-A6TV	1	1	1	0	1
Brian Lower grade Glioma	TCGA-S9-A6TW	1	1	0	1	1
Brian Lower grade Glioma	TCGA-S9-A6TX	1	1	0	1	1
Brian Lower grade Glioma	TCGA-S9-A6TY	1	1	0	1	1
Brian Lower grade Glioma	TCGA-S9-A6TZ	0	1	1	0	1
Brian Lower grade Glioma	TCGA-S9-A6U0	1	1	0	0	1
Brian Lower grade Glioma	TCGA-S9-A6U1	1	0	1	0	0
Brian Lower grade Glioma	TCGA-S9-A6U2	1	1	0	1	1
Brian Lower grade Glioma	TCGA-S9-A6U5	1	1	0	1	1
Brian Lower grade Glioma	TCGA-S9-A6U6	1	0	0	1	1
Brian Lower grade Glioma	TCGA-S9-A6U8	0	0	0	0	0
Brian Lower grade Glioma	TCGA-S9-A6U9	1	1	0	0	0
Brian Lower grade Glioma	TCGA-S9-A6UA	1	0	1	1	1
Brian Lower grade Glioma	TCGA-S9-A6UB	1	1	1	1	1
Brian Lower grade Glioma	TCGA-S9-A6WD	1	1	0	1	1
Brian Lower grade Glioma	TCGA-S9-A6WE	1	1	0	1	1
Brian Lower grade Glioma	TCGA-S9-A6WG	1	1	0	1	0
Brian Lower grade Glioma	TCGA-S9-A6WH	1	1	0	1	1
Brian Lower grade Glioma	TCGA-S9-A6WI	1	1	1	1	1
Brian Lower grade Glioma	TCGA-S9-A6WL	1	1	0	1	1
Brian Lower grade Glioma	TCGA-S9-A6WM	1	1	1	1	1
Brian Lower grade Glioma	TCGA-S9-A6WN	1	1	0	1	1
Brian Lower grade Glioma	TCGA-S9-A6WO	0	0	0	0	0
Brian Lower grade Glioma	TCGA-S9-A6WP	1	1	0	1	1
Brian Lower grade Glioma	TCGA-S9-A6WQ	0	1	0	1	0
Brian Lower grade Glioma	TCGA-S9-A7IQ	1	1	0	1	1
Brian Lower grade Glioma	TCGA-S9-A7IS	1	1	1	0	1
Brian Lower grade Glioma	TCGA-S9-A7IX	1	1	0	1	1

Brian Lower grade Glioma	TCGA-S9-A7IY	1	1	0	1	1
Brian Lower grade Glioma	TCGA-S9-A7IZ	1	1	0	0	1
Brian Lower grade Glioma	TCGA-S9-A7J0	1	1	1	1	1
Brian Lower grade Glioma	TCGA-S9-A7J1	1	1	0	1	1
Brian Lower grade Glioma	TCGA-S9-A7J2	1	1	0	1	1
Brian Lower grade Glioma	TCGA-S9-A7J3	1	1	0	1	1
Brian Lower grade Glioma	TCGA-S9-A7QW	0	1	0	0	1
Brian Lower grade Glioma	TCGA-S9-A7QX	1	0	1	0	0
Brian Lower grade Glioma	TCGA-S9-A7QY	1	1	0	1	1
Brian Lower grade Glioma	TCGA-S9-A7QZ	1	1	0	1	1
Brian Lower grade Glioma	TCGA-S9-A7R1	1	1	0	1	1
Brian Lower grade Glioma	TCGA-S9-A7R2	1	0	1	0	1
Brian Lower grade Glioma	TCGA-S9-A7R3	1	1	1	0	1
Brian Lower grade Glioma	TCGA-S9-A7R4	1	0	1	0	0
Brian Lower grade Glioma	TCGA-S9-A7R7	1	1	1	0	1
Brian Lower grade Glioma	TCGA-S9-A7R8	1	1	1	0	1
Brian Lower grade Glioma	TCGA-S9-A89V	1	1	1	1	1
Brian Lower grade Glioma	TCGA-S9-A89Z	1	1	0	1	1
Brian Lower grade Glioma	TCGA-TM-A7C3	1	0	0	0	1
Brian Lower grade Glioma	TCGA-TM-A7C4	0	0	0	0	1
Brian Lower grade Glioma	TCGA-TM-A7C5	1	1	0	1	1
Brian Lower grade Glioma	TCGA-TM-A7CA	0	0	0	0	1
Brian Lower grade Glioma	TCGA-TM-A7CF	1	1	1	1	1
Brian Lower grade Glioma	TCGA-TM-A84B	1	1	1	0	1
Brian Lower grade Glioma	TCGA-TM-A84C	0	0	0	0	0
Brian Lower grade Glioma	TCGA-TM-A84F	1	1	0	0	0
Brian Lower grade Glioma	TCGA-TM-A84G	1	1	0	1	1
Brian Lower grade Glioma	TCGA-TM-A84H	1	1	0	0	1
Brian Lower grade Glioma	TCGA-TM-A84I	1	1	1	1	1
Brian Lower grade Glioma	TCGA-TM-A84J	1	1	0	1	1
Brian Lower grade Glioma	TCGA-TM-A84L	0	0	0	0	1
Brian Lower grade Glioma	TCGA-TM-A84M	1	1	0	1	1
Brian Lower grade Glioma	TCGA-TM-A84O	1	1	0	1	1
Brian Lower grade Glioma	TCGA-TM-A84Q	1	1	1	0	1
Brian Lower grade Glioma	TCGA-TM-A84R	1	1	0	1	1
Brian Lower grade Glioma	TCGA-TM-A84S	1	1	0	1	1
Brian Lower grade Glioma	TCGA-TM-A84T	1	1	0	1	1
Brian Lower grade Glioma	TCGA-TQ-A7RF	1	1	1	0	1
Brian Lower grade Glioma	TCGA-TQ-A7RG	1	1	0	1	1
Brian Lower grade Glioma	TCGA-TQ-A7RH	1	1	1	1	1
Brian Lower grade Glioma	TCGA-TQ-A7RI	1	1	1	1	1
Brian Lower grade Glioma	TCGA-TQ-A7RJ	0	0	1	0	0
Brian Lower grade Glioma	TCGA-TQ-A7RK	0	1	0	0	1
Brian Lower grade Glioma	TCGA-TQ-A7RM	1	1	1	1	1
Brian Lower grade Glioma	TCGA-TQ-A7RN	1	1	0	1	1
Brian Lower grade Glioma	TCGA-TQ-A7RO	1	1	0	1	1
Brian Lower grade Glioma	TCGA-TQ-A7RP	1	1	0	0	1
Brian Lower grade Glioma	TCGA-TQ-A7RQ	1	1	0	1	1
Brian Lower grade Glioma	TCGA-TQ-A7RR	1	1	1	1	1
Brian Lower grade Glioma	TCGA-TQ-A7RS	1	1	0	1	1
Brian Lower grade Glioma	TCGA-TQ-A7RU	1	1	0	1	1
Brian Lower grade Glioma	TCGA-TQ-A7RV	0	0	0	0	1
Brian Lower grade Glioma	TCGA-TQ-A7RW	1	1	0	1	1
Brian Lower grade Glioma	TCGA-TQ-A8XE	1	1	1	0	1
Brian Lower grade Glioma	TCGA-VM-A8C8	1	1	1	1	1
Brian Lower grade Glioma	TCGA-VM-A8C9	0	0	0	1	0
Brian Lower grade Glioma	TCGA-VM-A8CA	1	1	0	1	1
Brian Lower grade Glioma	TCGA-VM-A8CB	1	1	0	1	1

Brian Lower grade Glioma	TCGA-VM-A8CD	1	1	1	1	1
Brian Lower grade Glioma	TCGA-VM-A8CE	1	1	0	1	1
Brian Lower grade Glioma	TCGA-VM-A8CF	1	1	1	1	1
Brian Lower grade Glioma	TCGA-VM-A8CH	0	0	0	0	0
Brian Lower grade Glioma	TCGA-VV-A829	1	1	0	1	1
Brian Lower grade Glioma	TCGA-VV-A86M	1	1	0	1	1
Brian Lower grade Glioma	TCGA-VW-A7QS	1	1	0	1	1
Brian Lower grade Glioma	TCGA-VW-A8FI	1	0	0	0	1
Brian Lower grade Glioma	TCGA-W9-A837	1	1	0	1	1
Brian Lower grade Glioma	TCGA-WH-A86K	1	1	0	1	1
Brian Lower grade Glioma	TCGA-WY-A858	1	1	1	0	1
Brian Lower grade Glioma	TCGA-WY-A859	0	0	0	0	1
Brian Lower grade Glioma	TCGA-WY-A85A	0	0	0	0	1
Brian Lower grade Glioma	TCGA-WY-A85B	1	1	1	0	1
Brian Lower grade Glioma	TCGA-WY-A85C	1	0	0	0	1
Brian Lower grade Glioma	TCGA-WY-A85D	1	0	0	1	1
Brian Lower grade Glioma	TCGA-WY-A85E	1	0	0	0	1
ervical squamous cell carcinom	TCGA-4J-AA1J	1	1	1	0	1
ervical squamous cell carcinom	TCGA-BI-A0VR	1	0	1	1	1
ervical squamous cell carcinom	TCGA-BI-A0VS	1	0	0	1	1
ervical squamous cell carcinom	TCGA-BI-A20A	1	1	1	1	1
ervical squamous cell carcinom	TCGA-C5-A0TN	1	1	1	1	1
ervical squamous cell carcinom	TCGA-C5-A1BE	1	1	1	0	1
ervical squamous cell carcinom	TCGA-C5-A1BF	0	0	1	1	1
ervical squamous cell carcinom	TCGA-C5-A1BI	0	0	1	1	1
ervical squamous cell carcinom	TCGA-C5-A1BJ	1	1	1	0	1
ervical squamous cell carcinom	TCGA-C5-A1BK	1	0	1	0	1
ervical squamous cell carcinom	TCGA-C5-A1BL	1	1	1	1	1
ervical squamous cell carcinom	TCGA-C5-A1BM	1	1	1	1	1
ervical squamous cell carcinom	TCGA-C5-A1BN	1	1	1	1	1
ervical squamous cell carcinom	TCGA-C5-A1BQ	1	0	1	1	1
ervical squamous cell carcinom	TCGA-C5-A1M5	0	1	1	1	1
ervical squamous cell carcinom	TCGA-C5-A1M6	1	1	1	1	1
ervical squamous cell carcinom	TCGA-C5-A1M7	0	0	0	0	1
ervical squamous cell carcinom	TCGA-C5-A1M8	1	1	1	1	1
ervical squamous cell carcinom	TCGA-C5-A1M9	1	1	1	1	1
ervical squamous cell carcinom	TCGA-C5-A1ME	1	1	1	1	1
ervical squamous cell carcinom	TCGA-C5-A1MF	0	1	1	0	1
ervical squamous cell carcinom	TCGA-C5-A1MH	1	1	1	1	1
ervical squamous cell carcinom	TCGA-C5-A1MI	1	1	1	1	1
ervical squamous cell carcinom	TCGA-C5-A1MJ	1	1	1	1	1
ervical squamous cell carcinom	TCGA-C5-A1MK	1	0	0	0	1
ervical squamous cell carcinom	TCGA-C5-A1ML	1	1	1	1	1
ervical squamous cell carcinom	TCGA-C5-A1MN	1	1	1	1	1
ervical squamous cell carcinom	TCGA-C5-A1MP	0	0	0	0	0
ervical squamous cell carcinom	TCGA-C5-A1MQ	0	0	0	0	0
ervical squamous cell carcinom	TCGA-C5-A2LS	0	0	0	0	0
ervical squamous cell carcinom	TCGA-C5-A2LT	1	1	1	1	1
ervical squamous cell carcinom	TCGA-C5-A2LV	1	1	1	1	1
ervical squamous cell carcinom	TCGA-C5-A2LX	1	1	1	1	1
ervical squamous cell carcinom	TCGA-C5-A2LY	1	0	1	1	1
ervical squamous cell carcinom	TCGA-C5-A2LZ	0	0	0	1	1
ervical squamous cell carcinom	TCGA-C5-A2M1	1	0	1	1	1
ervical squamous cell carcinom	TCGA-C5-A2M2	1	0	1	1	1
ervical squamous cell carcinom	TCGA-C5-A3HD	1	1	1	1	1
ervical squamous cell carcinom	TCGA-C5-A3HE	1	1	1	1	1
ervical squamous cell carcinom	TCGA-C5-A3HF	1	0	1	1	0
ervical squamous cell carcinom	TCGA-C5-A3HL	1	0	0	0	0

ervical squamous cell carcinom	TCGA-C5-A7CG	1	1	1	1	1
ervical squamous cell carcinom	TCGA-C5-A7CH	1	1	1	1	1
ervical squamous cell carcinom	TCGA-C5-A7CJ	1	0	0	1	0
ervical squamous cell carcinom	TCGA-C5-A7CK	1	0	0	1	1
ervical squamous cell carcinom	TCGA-C5-A7CL	1	0	1	1	1
ervical squamous cell carcinom	TCGA-C5-A7CM	1	1	1	1	1
ervical squamous cell carcinom	TCGA-C5-A7CO	1	0	1	1	1
ervical squamous cell carcinom	TCGA-C5-A7UC	1	1	1	1	1
ervical squamous cell carcinom	TCGA-C5-A7UE	1	0	0	1	1
ervical squamous cell carcinom	TCGA-C5-A7UH	1	1	1	1	1
ervical squamous cell carcinom	TCGA-C5-A7UI	1	1	1	1	1
ervical squamous cell carcinom	TCGA-C5-A7X3	1	1	1	1	1
ervical squamous cell carcinom	TCGA-C5-A7X5	1	1	1	1	1
ervical squamous cell carcinom	TCGA-C5-A7X8	1	1	1	1	1
ervical squamous cell carcinom	TCGA-C5-A7XC	0	1	1	0	1
ervical squamous cell carcinom	TCGA-C5-A8XH	1	1	1	1	1
ervical squamous cell carcinom	TCGA-C5-A8XI	1	0	0	0	1
ervical squamous cell carcinom	TCGA-C5-A8XJ	1	1	1	1	1
ervical squamous cell carcinom	TCGA-C5-A8XK	1	1	1	0	1
ervical squamous cell carcinom	TCGA-C5-A8YQ	1	1	1	1	1
ervical squamous cell carcinom	TCGA-C5-A8YR	1	0	1	1	1
ervical squamous cell carcinom	TCGA-C5-A8YT	0	0	0	0	0
ervical squamous cell carcinom	TCGA-C5-A8ZZ	1	0	1	1	1
ervical squamous cell carcinom	TCGA-C5-A901	0	0	0	0	0
ervical squamous cell carcinom	TCGA-C5-A902	0	0	0	0	0
ervical squamous cell carcinom	TCGA-C5-A905	0	0	0	0	0
ervical squamous cell carcinom	TCGA-C5-A907	1	1	1	0	0
ervical squamous cell carcinom	TCGA-DG-A2KH	1	1	1	1	1
ervical squamous cell carcinom	TCGA-DG-A2KJ	1	1	1	1	1
ervical squamous cell carcinom	TCGA-DG-A2KK	1	0	1	1	1
ervical squamous cell carcinom	TCGA-DG-A2KL	1	1	1	1	1
ervical squamous cell carcinom	TCGA-DG-A2KM	1	1	1	1	1
ervical squamous cell carcinom	TCGA-DR-A0ZL	1	0	1	1	1
ervical squamous cell carcinom	TCGA-DR-A0ZM	1	1	0	0	1
ervical squamous cell carcinom	TCGA-DS-A0VK	1	1	1	1	1
ervical squamous cell carcinom	TCGA-DS-A0VL	1	0	1	0	1
ervical squamous cell carcinom	TCGA-DS-A0VM	1	1	1	1	1
ervical squamous cell carcinom	TCGA-DS-A0VN	1	1	1	1	1
ervical squamous cell carcinom	TCGA-DS-A1O9	0	0	0	0	0
ervical squamous cell carcinom	TCGA-DS-A1OA	0	0	0	0	0
ervical squamous cell carcinom	TCGA-DS-A1OB	0	0	0	0	0
ervical squamous cell carcinom	TCGA-DS-A1OC	0	0	0	0	0
ervical squamous cell carcinom	TCGA-DS-A1OD	0	0	0	0	0
ervical squamous cell carcinom	TCGA-DS-A3LQ	1	1	1	0	1
ervical squamous cell carcinom	TCGA-DS-A5RQ	1	0	1	1	1
ervical squamous cell carcinom	TCGA-DS-A7WF	1	0	0	0	0
ervical squamous cell carcinom	TCGA-DS-A7WH	1	0	1	1	1
ervical squamous cell carcinom	TCGA-DS-A7WI	0	1	1	0	1
ervical squamous cell carcinom	TCGA-EA-A1QS	1	1	1	1	1
ervical squamous cell carcinom	TCGA-EA-A1QT	1	1	1	1	1
ervical squamous cell carcinom	TCGA-EA-A3HQ	1	0	1	0	1
ervical squamous cell carcinom	TCGA-EA-A3HR	1	1	1	1	1
ervical squamous cell carcinom	TCGA-EA-A3HS	1	0	0	1	0
ervical squamous cell carcinom	TCGA-EA-A3HT	1	0	0	1	1
ervical squamous cell carcinom	TCGA-EA-A3HU	0	0	0	0	0
ervical squamous cell carcinom	TCGA-EA-A3QD	1	0	0	1	1
ervical squamous cell carcinom	TCGA-EA-A3QE	1	0	1	1	1
ervical squamous cell carcinom	TCGA-EA-A3Y4	1	0	1	0	0

ervical squamous cell carcinom	TCGA-EA-A410	0	0	0	0	0
ervical squamous cell carcinom	TCGA-EA-A411	1	1	1	1	1
ervical squamous cell carcinom	TCGA-EA-A439	1	1	1	1	1
ervical squamous cell carcinom	TCGA-EA-A43B	1	1	1	1	1
ervical squamous cell carcinom	TCGA-EA-A44S	1	1	1	1	1
ervical squamous cell carcinom	TCGA-EA-A4BA	1	0	0	0	1
ervical squamous cell carcinom	TCGA-EA-A50E	1	0	1	1	1
ervical squamous cell carcinom	TCGA-EA-A556	1	1	1	1	1
ervical squamous cell carcinom	TCGA-EA-A5FO	1	0	1	0	1
ervical squamous cell carcinom	TCGA-EA-A5O9	1	0	1	0	1
ervical squamous cell carcinom	TCGA-EA-A5ZD	0	0	0	1	0
ervical squamous cell carcinom	TCGA-EA-A5ZE	0	0	1	1	1
ervical squamous cell carcinom	TCGA-EA-A5ZF	1	1	1	1	1
ervical squamous cell carcinom	TCGA-EA-A6QX	1	0	1	1	1
ervical squamous cell carcinom	TCGA-EA-A78R	1	0	1	1	1
ervical squamous cell carcinom	TCGA-EA-A97N	1	1	1	1	1
ervical squamous cell carcinom	TCGA-EK-A2GZ	1	1	1	1	1
ervical squamous cell carcinom	TCGA-EK-A2H0	1	1	1	1	1
ervical squamous cell carcinom	TCGA-EK-A2H1	1	0	0	1	1
ervical squamous cell carcinom	TCGA-EK-A2IP	1	1	1	1	1
ervical squamous cell carcinom	TCGA-EK-A2IR	0	0	0	0	0
ervical squamous cell carcinom	TCGA-EK-A2PG	1	1	1	1	1
ervical squamous cell carcinom	TCGA-EK-A2PI	1	1	1	1	1
ervical squamous cell carcinom	TCGA-EK-A2PK	1	0	1	1	1
ervical squamous cell carcinom	TCGA-EK-A2PL	1	1	1	1	1
ervical squamous cell carcinom	TCGA-EK-A2PM	1	1	1	1	1
ervical squamous cell carcinom	TCGA-EK-A2R7	1	1	1	1	1
ervical squamous cell carcinom	TCGA-EK-A2R8	1	1	1	1	1
ervical squamous cell carcinom	TCGA-EK-A2R9	0	0	1	0	1
ervical squamous cell carcinom	TCGA-EK-A2RA	1	1	1	1	1
ervical squamous cell carcinom	TCGA-EK-A2RB	0	0	1	1	0
ervical squamous cell carcinom	TCGA-EK-A2RC	0	0	1	1	0
ervical squamous cell carcinom	TCGA-EK-A2RE	0	1	0	1	1
ervical squamous cell carcinom	TCGA-EK-A2RJ	1	1	1	1	1
ervical squamous cell carcinom	TCGA-EK-A2RK	1	1	1	1	1
ervical squamous cell carcinom	TCGA-EK-A2RL	1	0	1	0	1
ervical squamous cell carcinom	TCGA-EK-A2RM	1	1	1	1	1
ervical squamous cell carcinom	TCGA-EK-A2RN	1	1	1	1	1
ervical squamous cell carcinom	TCGA-EK-A2RO	1	1	1	0	1
ervical squamous cell carcinom	TCGA-EK-A3GJ	1	1	1	0	1
ervical squamous cell carcinom	TCGA-EK-A3GK	1	1	1	1	1
ervical squamous cell carcinom	TCGA-EK-A3GM	1	1	1	1	1
ervical squamous cell carcinom	TCGA-EK-A3GN	1	0	1	0	0
ervical squamous cell carcinom	TCGA-EX-A1H5	1	1	1	1	1
ervical squamous cell carcinom	TCGA-EX-A1H6	1	1	1	0	1
ervical squamous cell carcinom	TCGA-EX-A3L1	1	1	1	1	1
ervical squamous cell carcinom	TCGA-EX-A449	1	1	0	0	0
ervical squamous cell carcinom	TCGA-EX-A69L	1	1	1	1	1
ervical squamous cell carcinom	TCGA-EX-A69M	0	1	1	0	1
ervical squamous cell carcinom	TCGA-EX-A8YF	1	0	0	1	1
ervical squamous cell carcinom	TCGA-FU-A23K	1	1	0	0	1
ervical squamous cell carcinom	TCGA-FU-A23L	1	1	1	1	1
ervical squamous cell carcinom	TCGA-FU-A2QG	1	0	1	1	1
ervical squamous cell carcinom	TCGA-FU-A3EO	1	1	0	1	0
ervical squamous cell carcinom	TCGA-FU-A3HY	1	1	1	1	1
ervical squamous cell carcinom	TCGA-FU-A3HZ	1	1	0	0	1
ervical squamous cell carcinom	TCGA-FU-A3NI	1	1	1	1	1
ervical squamous cell carcinom	TCGA-FU-A3TQ	1	1	1	1	1

ervical squamous cell carcinom	TCGA-FU-A3TX	1	0	0	1	1
ervical squamous cell carcinom	TCGA-FU-A3WB	1	1	0	0	1
ervical squamous cell carcinom	TCGA-FU-A3YQ	0	1	1	1	1
ervical squamous cell carcinom	TCGA-FU-A40J	1	1	1	1	1
ervical squamous cell carcinom	TCGA-FU-A57G	1	1	1	0	0
ervical squamous cell carcinom	TCGA-FU-A5XV	1	1	1	1	1
ervical squamous cell carcinom	TCGA-FU-A770	1	1	0	1	1
ervical squamous cell carcinom	TCGA-GH-A9DA	1	1	1	0	1
ervical squamous cell carcinom	TCGA-HG-A2PA	1	0	1	1	1
ervical squamous cell carcinom	TCGA-HM-A3JJ	1	1	1	1	1
ervical squamous cell carcinom	TCGA-HM-A3JK	1	0	0	1	1
ervical squamous cell carcinom	TCGA-HM-A4S6	0	1	1	0	0
ervical squamous cell carcinom	TCGA-HM-A6W2	1	1	0	1	1
ervical squamous cell carcinom	TCGA-HM-A6W2	0	0	0	0	0
ervical squamous cell carcinom	TCGA-IR-A3L7	1	1	1	1	1
ervical squamous cell carcinom	TCGA-IR-A3LA	1	1	1	1	1
ervical squamous cell carcinom	TCGA-IR-A3LB	1	1	0	1	1
ervical squamous cell carcinom	TCGA-IR-A3LC	1	1	1	1	1
ervical squamous cell carcinom	TCGA-IR-A3LF	1	1	0	1	1
ervical squamous cell carcinom	TCGA-IR-A3LH	1	1	1	1	1
ervical squamous cell carcinom	TCGA-IR-A3LI	1	0	0	0	1
ervical squamous cell carcinom	TCGA-IR-A3LK	1	1	1	1	1
ervical squamous cell carcinom	TCGA-IR-A3LL	1	1	0	0	1
ervical squamous cell carcinom	TCGA-JW-A5VG	0	0	0	1	1
ervical squamous cell carcinom	TCGA-JW-A5VH	1	0	0	0	0
ervical squamous cell carcinom	TCGA-JW-A5VI	1	1	1	1	1
ervical squamous cell carcinom	TCGA-JW-A5VJ	1	0	1	1	0
ervical squamous cell carcinom	TCGA-JW-A5VK	1	1	1	0	1
ervical squamous cell carcinom	TCGA-JW-A5VL	1	1	0	1	1
ervical squamous cell carcinom	TCGA-JW-A69B	1	0	1	0	1
ervical squamous cell carcinom	TCGA-JW-A852	1	1	0	0	0
ervical squamous cell carcinom	TCGA-JW-AAVH	1	0	0	1	0
ervical squamous cell carcinom	TCGA-JX-A3PZ	1	1	1	1	1
ervical squamous cell carcinom	TCGA-JX-A3Q0	1	0	0	0	1
ervical squamous cell carcinom	TCGA-JX-A3Q8	1	1	0	1	1
ervical squamous cell carcinom	TCGA-JX-A5QV	1	0	1	1	1
ervical squamous cell carcinom	TCGA-LP-A4AU	1	0	1	0	1
ervical squamous cell carcinom	TCGA-LP-A4AV	1	1	0	1	1
ervical squamous cell carcinom	TCGA-LP-A4AW	1	1	1	1	1
ervical squamous cell carcinom	TCGA-LP-A4AX	1	0	1	1	1
ervical squamous cell carcinom	TCGA-LP-A5U2	1	1	1	1	1
ervical squamous cell carcinom	TCGA-LP-A5U3	0	1	0	0	1
ervical squamous cell carcinom	TCGA-LP-A7HU	1	1	1	1	1
ervical squamous cell carcinom	TCGA-MA-AA3W	1	0	1	1	1
ervical squamous cell carcinom	TCGA-MA-AA3X	1	1	1	1	1
ervical squamous cell carcinom	TCGA-MA-AA3Y	0	1	1	0	1
ervical squamous cell carcinom	TCGA-MA-AA3Z	1	1	1	0	1
ervical squamous cell carcinom	TCGA-MA-AA41	1	1	0	0	0
ervical squamous cell carcinom	TCGA-MA-AA42	1	1	1	1	1
ervical squamous cell carcinom	TCGA-MA-AA43	1	1	1	1	1
ervical squamous cell carcinom	TCGA-MU-A51Y	0	1	0	1	1
ervical squamous cell carcinom	TCGA-MU-A5YI	1	1	1	1	1
ervical squamous cell carcinom	TCGA-MU-A8JM	0	0	0	1	1
ervical squamous cell carcinom	TCGA-MY-A5BD	1	1	0	1	1
ervical squamous cell carcinom	TCGA-MY-A5BE	1	0	0	0	1
ervical squamous cell carcinom	TCGA-MY-A5BF	1	0	0	1	1
ervical squamous cell carcinom	TCGA-MY-A913	1	1	1	1	1
ervical squamous cell carcinom	TCGA-PN-A8MA	1	1	1	1	1

ervical squamous cell carcinom	TCGA-Q1-A5R1	1	1	1	1	1
ervical squamous cell carcinom	TCGA-Q1-A5R2	1	1	1	1	1
ervical squamous cell carcinom	TCGA-Q1-A5R3	1	0	1	0	1
ervical squamous cell carcinom	TCGA-Q1-A6DT	1	1	1	1	1
ervical squamous cell carcinom	TCGA-Q1-A6DV	1	0	0	0	0
ervical squamous cell carcinom	TCGA-Q1-A6DW	1	0	0	0	1
ervical squamous cell carcinom	TCGA-Q1-A73O	1	1	1	0	1
ervical squamous cell carcinom	TCGA-Q1-A73P	1	0	0	0	1
ervical squamous cell carcinom	TCGA-Q1-A73Q	1	0	1	1	1
ervical squamous cell carcinom	TCGA-Q1-A73R	1	0	0	0	1
ervical squamous cell carcinom	TCGA-Q1-A73S	1	1	1	1	1
ervical squamous cell carcinom	TCGA-R2-A69V	1	1	1	1	1
ervical squamous cell carcinom	TCGA-RA-A741	1	0	1	1	1
ervical squamous cell carcinom	TCGA-UC-A7PD	1	1	1	1	1
ervical squamous cell carcinom	TCGA-UC-A7PF	0	1	0	1	1
ervical squamous cell carcinom	TCGA-UC-A7PG	1	1	1	1	1
ervical squamous cell carcinom	TCGA-UC-A7PG	0	0	0	0	0
ervical squamous cell carcinom	TCGA-UC-A7PI	1	0	1	0	1
ervical squamous cell carcinom	TCGA-VS-A8EB	1	1	1	1	1
ervical squamous cell carcinom	TCGA-VS-A8EC	1	1	1	1	1
ervical squamous cell carcinom	TCGA-VS-A8EG	0	0	0	0	1
ervical squamous cell carcinom	TCGA-VS-A8EH	1	1	1	1	1
ervical squamous cell carcinom	TCGA-VS-A8EI	1	1	1	1	1
ervical squamous cell carcinom	TCGA-VS-A8EJ	1	1	1	1	1
ervical squamous cell carcinom	TCGA-VS-A8EK	0	0	0	0	0
ervical squamous cell carcinom	TCGA-VS-A8EL	1	1	1	1	1
ervical squamous cell carcinom	TCGA-VS-A8Q8	1	0	1	1	1
ervical squamous cell carcinom	TCGA-VS-A8Q9	1	1	1	1	1
ervical squamous cell carcinom	TCGA-VS-A8QA	1	1	1	1	1
ervical squamous cell carcinom	TCGA-VS-A8QC	1	1	1	1	1
ervical squamous cell carcinom	TCGA-VS-A8QF	1	1	1	1	1
ervical squamous cell carcinom	TCGA-VS-A8QH	1	1	1	1	1
ervical squamous cell carcinom	TCGA-VS-A8QM	1	0	0	1	1
ervical squamous cell carcinom	TCGA-VS-A94W	0	0	0	0	1
ervical squamous cell carcinom	TCGA-VS-A94X	1	0	1	1	1
ervical squamous cell carcinom	TCGA-VS-A94Y	1	1	1	1	1
ervical squamous cell carcinom	TCGA-VS-A94Z	1	1	1	1	1
ervical squamous cell carcinom	TCGA-VS-A950	1	1	1	1	1
ervical squamous cell carcinom	TCGA-VS-A952	1	1	0	1	1
ervical squamous cell carcinom	TCGA-VS-A953	1	1	1	1	1
ervical squamous cell carcinom	TCGA-VS-A954	1	0	1	0	1
ervical squamous cell carcinom	TCGA-VS-A957	1	1	1	1	1
ervical squamous cell carcinom	TCGA-VS-A958	1	0	1	0	1
ervical squamous cell carcinom	TCGA-VS-A959	1	1	1	0	1
ervical squamous cell carcinom	TCGA-VS-A9U5	1	1	1	1	1
ervical squamous cell carcinom	TCGA-VS-A9U6	1	1	1	0	1
ervical squamous cell carcinom	TCGA-VS-A9U7	1	1	1	1	1
ervical squamous cell carcinom	TCGA-VS-A9UB	1	0	1	0	0
ervical squamous cell carcinom	TCGA-VS-A9UC	0	0	1	0	1
ervical squamous cell carcinom	TCGA-VS-A9UD	1	1	1	1	1
ervical squamous cell carcinom	TCGA-VS-A9UH	1	0	0	1	1
ervical squamous cell carcinom	TCGA-VS-A9UI	1	1	1	1	1
ervical squamous cell carcinom	TCGA-VS-A9UJ	0	0	0	0	0
ervical squamous cell carcinom	TCGA-VS-A9UL	1	1	1	1	1
ervical squamous cell carcinom	TCGA-VS-A9UM	1	1	1	1	1
ervical squamous cell carcinom	TCGA-VS-A9UO	1	1	1	1	1
ervical squamous cell carcinom	TCGA-VS-A9UP	1	1	1	1	1
ervical squamous cell carcinom	TCGA-VS-A9UQ	1	1	1	1	1

ervical squamous cell carcinom	TCGA-VS-A9UR	1	1	1	1	1
ervical squamous cell carcinom	TCGA-VS-A9UT	1	1	1	1	1
ervical squamous cell carcinom	TCGA-VS-A9UU	1	1	1	0	1
ervical squamous cell carcinom	TCGA-VS-A9UV	1	1	1	1	1
ervical squamous cell carcinom	TCGA-VS-A9UY	0	1	1	1	1
ervical squamous cell carcinom	TCGA-VS-A9UZ	1	0	0	1	1
ervical squamous cell carcinom	TCGA-VS-A9V0	1	1	1	1	1
ervical squamous cell carcinom	TCGA-VS-A9V1	1	0	1	0	0
ervical squamous cell carcinom	TCGA-VS-A9V2	1	0	1	0	1
ervical squamous cell carcinom	TCGA-VS-A9V3	1	1	1	1	1
ervical squamous cell carcinom	TCGA-VS-A9V4	1	1	1	1	1
ervical squamous cell carcinom	TCGA-VS-A9V5	1	1	1	1	1
ervical squamous cell carcinom	TCGA-VS-AA62	0	1	1	0	1
ervical squamous cell carcinom	TCGA-WL-A834	1	1	1	1	1
ervical squamous cell carcinom	TCGA-XS-A8TJ	1	1	1	1	1
ervical squamous cell carcinom	TCGA-ZJ-A8QO	1	0	1	1	1
ervical squamous cell carcinom	TCGA-ZJ-A8QQ	1	1	1	1	1
ervical squamous cell carcinom	TCGA-ZJ-A8QR	1	1	1	1	1
ervical squamous cell carcinom	TCGA-ZJ-AAX4	1	1	1	1	1
ervical squamous cell carcinom	TCGA-ZJ-AAX8	1	0	1	0	1
ervical squamous cell carcinom	TCGA-ZJ-AAXA	1	1	1	1	1
ervical squamous cell carcinom	TCGA-ZJ-AAXB	1	1	1	1	1
ervical squamous cell carcinom	TCGA-ZJ-AAXD	1	0	0	1	1
ervical squamous cell carcinom	TCGA-ZJ-AAXF	1	1	1	0	1
ervical squamous cell carcinom	TCGA-ZJ-AAXI	1	1	1	0	1
ervical squamous cell carcinom	TCGA-ZJ-AAXJ	1	1	1	1	1
ervical squamous cell carcinom	TCGA-ZJ-AAXN	1	1	1	1	1
ervical squamous cell carcinom	TCGA-ZJ-AAXT	1	1	1	1	1
ervical squamous cell carcinom	TCGA-ZJ-AAXU	1	0	0	1	1
ervical squamous cell carcinom	TCGA-ZJ-AB0H	1	0	1	1	1
ervical squamous cell carcinom	TCGA-ZJ-AB0I	1	0	1	1	1
ervical squamous cell carcinom	TCGA-ZX-AA5X	0	1	0	1	1
Cholangiocarcinoma	TCGA-3X-AAVA	1	1	1	1	1
Cholangiocarcinoma	TCGA-3X-AAVB	1	1	1	1	1
Cholangiocarcinoma	TCGA-3X-AAVC	1	1	1	1	1
Cholangiocarcinoma	TCGA-3X-AAVE	1	1	1	1	1
Cholangiocarcinoma	TCGA-4G-AAZO	1	1	1	1	1
Cholangiocarcinoma	TCGA-4G-AAZT	1	1	1	1	1
Cholangiocarcinoma	TCGA-W5-AA2G	1	1	1	1	1
Cholangiocarcinoma	TCGA-W5-AA2H	0	0	0	0	0
Cholangiocarcinoma	TCGA-W5-AA2I	1	1	1	1	1
Cholangiocarcinoma	TCGA-W5-AA2O	1	1	1	1	1
Cholangiocarcinoma	TCGA-W5-AA2Q	1	1	0	1	1
Cholangiocarcinoma	TCGA-W5-AA2R	1	1	1	1	1
Cholangiocarcinoma	TCGA-W5-AA2T	1	1	1	1	1
Cholangiocarcinoma	TCGA-W5-AA2U	1	1	1	1	1
Cholangiocarcinoma	TCGA-W5-AA2W	1	1	1	1	1
Cholangiocarcinoma	TCGA-W5-AA2X	1	1	1	1	1
Cholangiocarcinoma	TCGA-W5-AA2Z	0	1	1	1	1
Cholangiocarcinoma	TCGA-W5-AA30	1	1	1	1	1
Cholangiocarcinoma	TCGA-W5-AA31	1	1	1	1	1
Cholangiocarcinoma	TCGA-W5-AA33	1	1	1	1	1
Cholangiocarcinoma	TCGA-W5-AA34	1	1	1	1	1
Cholangiocarcinoma	TCGA-W5-AA36	1	1	1	1	1
Cholangiocarcinoma	TCGA-W5-AA38	1	1	1	1	1
Cholangiocarcinoma	TCGA-W5-AA39	1	1	0	1	1
Cholangiocarcinoma	TCGA-W6-AA0S	1	1	1	1	1
Cholangiocarcinoma	TCGA-WD-A7RX	1	1	1	1	1

Cholangiocarcinoma	TCGA-YR-A95A	1	1	1	1	1
Cholangiocarcinoma	TCGA-ZD-A8I3	1	1	1	1	1
Cholangiocarcinoma	TCGA-ZH-A8Y1	1	1	0	1	0
Cholangiocarcinoma	TCGA-ZH-A8Y2	1	1	1	1	1
Cholangiocarcinoma	TCGA-ZH-A8Y4	1	1	1	1	1
Cholangiocarcinoma	TCGA-ZH-A8Y5	1	1	1	1	1
Cholangiocarcinoma	TCGA-ZH-A8Y6	0	0	0	0	0
Cholangiocarcinoma	TCGA-ZH-A8Y8	1	1	1	1	1
Cholangiocarcinoma	TCGA-ZU-A8S4	1	1	1	1	1
colorectal adenocarcinoma	TCGA-4N-A93T	1	1	1	1	1
colorectal adenocarcinoma	TCGA-4T-AA8H	1	0	0	1	1
colorectal adenocarcinoma	TCGA-5M-AAT4	1	1	1	1	1
colorectal adenocarcinoma	TCGA-5M-AAT5	1	1	1	1	1
colorectal adenocarcinoma	TCGA-5M-AAT6	1	1	0	1	1
colorectal adenocarcinoma	TCGA-5M-AATA	1	1	1	1	1
colorectal adenocarcinoma	TCGA-5M-AATE	1	1	1	1	1
colorectal adenocarcinoma	TCGA-A6-2675	1	1	1	1	1
colorectal adenocarcinoma	TCGA-A6-2682	1	1	1	1	1
colorectal adenocarcinoma	TCGA-A6-2684	1	1	1	0	1
colorectal adenocarcinoma	TCGA-A6-2685	1	1	1	1	1
colorectal adenocarcinoma	TCGA-A6-2686	1	0	1	0	1
colorectal adenocarcinoma	TCGA-A6-4105	1	1	1	1	1
colorectal adenocarcinoma	TCGA-A6-5656	1	1	1	0	1
colorectal adenocarcinoma	TCGA-A6-5657	1	1	1	0	1
colorectal adenocarcinoma	TCGA-A6-5659	1	0	1	1	1
colorectal adenocarcinoma	TCGA-A6-5660	1	1	1	1	1
colorectal adenocarcinoma	TCGA-A6-5661	0	0	0	0	0
colorectal adenocarcinoma	TCGA-A6-5662	1	1	0	1	1
colorectal adenocarcinoma	TCGA-A6-5664	1	0	0	0	0
colorectal adenocarcinoma	TCGA-A6-5665	0	0	0	0	0
colorectal adenocarcinoma	TCGA-A6-5666	1	1	1	1	1
colorectal adenocarcinoma	TCGA-A6-5667	1	1	1	1	1
colorectal adenocarcinoma	TCGA-A6-6137	1	1	1	0	1
colorectal adenocarcinoma	TCGA-A6-6138	1	1	1	1	1
colorectal adenocarcinoma	TCGA-A6-6140	1	1	1	1	1
colorectal adenocarcinoma	TCGA-A6-6141	0	0	0	0	0
colorectal adenocarcinoma	TCGA-A6-6142	1	1	1	1	1
colorectal adenocarcinoma	TCGA-A6-6648	1	1	1	1	1
colorectal adenocarcinoma	TCGA-A6-6649	1	1	1	1	1
colorectal adenocarcinoma	TCGA-A6-6650	1	1	0	1	1
colorectal adenocarcinoma	TCGA-A6-6651	1	1	1	1	1
colorectal adenocarcinoma	TCGA-A6-6652	1	0	1	0	0
colorectal adenocarcinoma	TCGA-A6-6653	1	0	1	0	0
colorectal adenocarcinoma	TCGA-A6-6654	1	1	1	1	1
colorectal adenocarcinoma	TCGA-A6-6780	0	0	0	0	1
colorectal adenocarcinoma	TCGA-A6-6781	0	0	0	0	0
colorectal adenocarcinoma	TCGA-A6-6782	1	0	1	1	1
colorectal adenocarcinoma	TCGA-A6-A565	0	0	0	0	0
colorectal adenocarcinoma	TCGA-A6-A566	0	0	0	0	0
colorectal adenocarcinoma	TCGA-A6-A567	1	1	1	1	1
colorectal adenocarcinoma	TCGA-A6-A56B	1	1	0	1	1
colorectal adenocarcinoma	TCGA-A6-A5ZU	1	1	1	1	1
colorectal adenocarcinoma	TCGA-AA-3489	1	0	1	1	1
colorectal adenocarcinoma	TCGA-AA-3492	0	0	0	0	0
colorectal adenocarcinoma	TCGA-AA-3495	1	1	1	1	1
colorectal adenocarcinoma	TCGA-AA-3496	1	0	1	0	1
colorectal adenocarcinoma	TCGA-AA-3502	1	0	0	0	1
colorectal adenocarcinoma	TCGA-AA-3506	1	0	1	0	1

colorectal adenocarcinoma	TCGA-AA-3509	0	0	0	0	0
colorectal adenocarcinoma	TCGA-AA-3511	1	1	1	1	1
colorectal adenocarcinoma	TCGA-AA-3526	1	1	0	1	1
colorectal adenocarcinoma	TCGA-AA-3655	0	0	1	0	1
colorectal adenocarcinoma	TCGA-AA-3660	1	0	1	0	1
colorectal adenocarcinoma	TCGA-AA-3662	1	1	1	1	1
colorectal adenocarcinoma	TCGA-AA-3663	0	0	0	0	0
colorectal adenocarcinoma	TCGA-AA-3675	0	0	0	0	0
colorectal adenocarcinoma	TCGA-AA-3685	1	1	1	1	1
colorectal adenocarcinoma	TCGA-AA-3697	1	1	1	1	1
colorectal adenocarcinoma	TCGA-AA-3712	1	1	1	1	1
colorectal adenocarcinoma	TCGA-AA-3713	1	0	0	0	0
colorectal adenocarcinoma	TCGA-AA-A01P	0	0	0	1	0
colorectal adenocarcinoma	TCGA-AA-A01X	1	1	1	1	1
colorectal adenocarcinoma	TCGA-AA-A01Z	1	1	1	1	1
colorectal adenocarcinoma	TCGA-AA-A02K	0	0	0	0	0
colorectal adenocarcinoma	TCGA-AA-A02Y	1	0	0	0	1
colorectal adenocarcinoma	TCGA-AD-5900	0	0	0	0	0
colorectal adenocarcinoma	TCGA-AD-6548	0	0	1	0	1
colorectal adenocarcinoma	TCGA-AD-6888	1	1	1	0	1
colorectal adenocarcinoma	TCGA-AD-6889	0	0	0	0	0
colorectal adenocarcinoma	TCGA-AD-6890	1	1	1	1	1
colorectal adenocarcinoma	TCGA-AD-6895	0	0	0	0	0
colorectal adenocarcinoma	TCGA-AD-6899	1	1	1	1	1
colorectal adenocarcinoma	TCGA-AD-6901	1	1	1	1	1
colorectal adenocarcinoma	TCGA-AD-6963	1	0	1	1	1
colorectal adenocarcinoma	TCGA-AD-6964	0	0	0	0	0
colorectal adenocarcinoma	TCGA-AD-6965	1	1	1	1	1
colorectal adenocarcinoma	TCGA-AD-A5EJ	0	0	0	0	0
colorectal adenocarcinoma	TCGA-AD-A5EK	1	1	1	1	1
colorectal adenocarcinoma	TCGA-AF-2687	1	1	1	1	1
colorectal adenocarcinoma	TCGA-AF-2690	1	1	1	1	1
colorectal adenocarcinoma	TCGA-AF-2693	1	1	1	1	1
colorectal adenocarcinoma	TCGA-AF-3911	1	1	1	1	1
colorectal adenocarcinoma	TCGA-AF-4110	1	1	1	1	1
colorectal adenocarcinoma	TCGA-AF-5654	1	0	0	0	0
colorectal adenocarcinoma	TCGA-AF-6136	1	1	1	1	1
colorectal adenocarcinoma	TCGA-AF-6655	1	0	1	0	1
colorectal adenocarcinoma	TCGA-AF-6672	1	0	1	1	1
colorectal adenocarcinoma	TCGA-AF-A56K	1	1	1	1	1
colorectal adenocarcinoma	TCGA-AF-A56L	1	1	1	1	1
colorectal adenocarcinoma	TCGA-AF-A56N	1	1	1	1	1
colorectal adenocarcinoma	TCGA-AG-3591	1	1	1	1	1
colorectal adenocarcinoma	TCGA-AG-3592	1	1	0	0	1
colorectal adenocarcinoma	TCGA-AG-3725	1	1	1	1	1
colorectal adenocarcinoma	TCGA-AG-3731	1	1	1	1	1
colorectal adenocarcinoma	TCGA-AG-3732	1	1	1	1	1
colorectal adenocarcinoma	TCGA-AG-3742	1	1	1	1	1
colorectal adenocarcinoma	TCGA-AG-3902	1	1	1	1	1
colorectal adenocarcinoma	TCGA-AG-4021	1	1	1	1	1
colorectal adenocarcinoma	TCGA-AG-4022	1	0	0	0	0
colorectal adenocarcinoma	TCGA-AH-6544	1	1	1	1	1
colorectal adenocarcinoma	TCGA-AH-6547	0	1	0	0	0
colorectal adenocarcinoma	TCGA-AH-6549	0	0	0	0	0
colorectal adenocarcinoma	TCGA-AH-6643	1	1	1	1	1
colorectal adenocarcinoma	TCGA-AH-6644	1	1	1	1	1
colorectal adenocarcinoma	TCGA-AH-6897	1	0	1	0	1
colorectal adenocarcinoma	TCGA-AH-6903	0	0	0	0	0

colorectal adenocarcinoma	TCGA-AM-5820	1	1	1	1	1
colorectal adenocarcinoma	TCGA-AM-5821	0	0	0	1	1
colorectal adenocarcinoma	TCGA-AU-3779	1	1	1	1	1
colorectal adenocarcinoma	TCGA-AU-6004	0	0	0	0	0
colorectal adenocarcinoma	TCGA-AY-5543	1	0	1	1	1
colorectal adenocarcinoma	TCGA-AY-6196	1	1	1	1	1
colorectal adenocarcinoma	TCGA-AY-6197	0	0	0	0	0
colorectal adenocarcinoma	TCGA-AY-6386	0	0	0	0	0
colorectal adenocarcinoma	TCGA-AY-A54L	1	1	1	1	1
colorectal adenocarcinoma	TCGA-AY-A69D	1	1	1	1	1
colorectal adenocarcinoma	TCGA-AY-A71X	1	0	1	0	1
colorectal adenocarcinoma	TCGA-AY-A8YK	1	1	0	1	1
colorectal adenocarcinoma	TCGA-AZ-4313	0	0	0	0	0
colorectal adenocarcinoma	TCGA-AZ-4315	0	0	0	0	0
colorectal adenocarcinoma	TCGA-AZ-4323	1	1	1	1	1
colorectal adenocarcinoma	TCGA-AZ-4614	1	1	1	1	1
colorectal adenocarcinoma	TCGA-AZ-4615	0	0	0	1	0
colorectal adenocarcinoma	TCGA-AZ-4616	1	1	1	1	1
colorectal adenocarcinoma	TCGA-AZ-4682	1	1	0	1	1
colorectal adenocarcinoma	TCGA-AZ-4684	1	1	1	1	1
colorectal adenocarcinoma	TCGA-AZ-5403	1	1	1	1	1
colorectal adenocarcinoma	TCGA-AZ-5407	1	0	1	1	1
colorectal adenocarcinoma	TCGA-AZ-6598	1	0	0	1	0
colorectal adenocarcinoma	TCGA-AZ-6599	0	0	0	0	0
colorectal adenocarcinoma	TCGA-AZ-6600	1	1	1	1	1
colorectal adenocarcinoma	TCGA-AZ-6601	0	0	0	0	0
colorectal adenocarcinoma	TCGA-AZ-6603	1	1	1	1	1
colorectal adenocarcinoma	TCGA-AZ-6605	1	1	1	1	1
colorectal adenocarcinoma	TCGA-AZ-6606	1	1	0	0	1
colorectal adenocarcinoma	TCGA-AZ-6607	1	1	1	1	1
colorectal adenocarcinoma	TCGA-AZ-6608	1	1	1	1	1
colorectal adenocarcinoma	TCGA-BM-6198	1	1	1	1	1
colorectal adenocarcinoma	TCGA-CA-5254	1	1	1	1	1
colorectal adenocarcinoma	TCGA-CA-5255	1	1	1	1	1
colorectal adenocarcinoma	TCGA-CA-5256	1	1	1	1	1
colorectal adenocarcinoma	TCGA-CA-5796	0	0	0	1	1
colorectal adenocarcinoma	TCGA-CA-5797	1	1	1	1	1
colorectal adenocarcinoma	TCGA-CA-6715	1	1	1	1	1
colorectal adenocarcinoma	TCGA-CA-6716	1	1	1	1	1
colorectal adenocarcinoma	TCGA-CA-6717	0	0	0	0	0
colorectal adenocarcinoma	TCGA-CA-6718	0	0	0	0	1
colorectal adenocarcinoma	TCGA-CA-6719	1	1	1	1	1
colorectal adenocarcinoma	TCGA-CI-6619	1	1	1	1	1
colorectal adenocarcinoma	TCGA-CI-6620	1	1	1	0	1
colorectal adenocarcinoma	TCGA-CI-6621	1	1	0	1	1
colorectal adenocarcinoma	TCGA-CI-6622	1	1	1	1	1
colorectal adenocarcinoma	TCGA-CI-6623	1	1	0	1	1
colorectal adenocarcinoma	TCGA-CI-6624	1	1	0	1	1
colorectal adenocarcinoma	TCGA-CK-4947	0	0	0	0	0
colorectal adenocarcinoma	TCGA-CK-4948	1	1	1	1	1
colorectal adenocarcinoma	TCGA-CK-4950	0	0	0	0	0
colorectal adenocarcinoma	TCGA-CK-4951	0	0	0	0	1
colorectal adenocarcinoma	TCGA-CK-4952	1	1	1	1	1
colorectal adenocarcinoma	TCGA-CK-5912	1	1	1	1	1
colorectal adenocarcinoma	TCGA-CK-5913	0	0	0	0	0
colorectal adenocarcinoma	TCGA-CK-5914	1	1	1	1	1
colorectal adenocarcinoma	TCGA-CK-5915	1	1	1	1	1
colorectal adenocarcinoma	TCGA-CK-5916	0	0	0	0	0

colorectal adenocarcinoma	TCGA-CK-6746	0	0	0	0	0
colorectal adenocarcinoma	TCGA-CK-6747	0	0	0	0	0
colorectal adenocarcinoma	TCGA-CK-6748	1	1	0	1	1
colorectal adenocarcinoma	TCGA-CK-6751	0	0	0	0	0
colorectal adenocarcinoma	TCGA-CL-4957	1	1	1	1	1
colorectal adenocarcinoma	TCGA-CL-5917	1	0	0	1	0
colorectal adenocarcinoma	TCGA-CL-5918	1	1	1	1	1
colorectal adenocarcinoma	TCGA-CM-4743	0	0	0	0	0
colorectal adenocarcinoma	TCGA-CM-4744	0	0	0	0	0
colorectal adenocarcinoma	TCGA-CM-4747	1	1	1	1	1
colorectal adenocarcinoma	TCGA-CM-4751	1	1	1	1	1
colorectal adenocarcinoma	TCGA-CM-5344	1	1	1	1	1
colorectal adenocarcinoma	TCGA-CM-5348	1	1	1	0	1
colorectal adenocarcinoma	TCGA-CM-5349	1	1	0	1	1
colorectal adenocarcinoma	TCGA-CM-5860	0	0	1	0	0
colorectal adenocarcinoma	TCGA-CM-5861	0	0	0	0	0
colorectal adenocarcinoma	TCGA-CM-5862	1	1	1	1	1
colorectal adenocarcinoma	TCGA-CM-5863	1	1	1	1	1
colorectal adenocarcinoma	TCGA-CM-5864	1	1	1	1	1
colorectal adenocarcinoma	TCGA-CM-5868	1	1	1	1	1
colorectal adenocarcinoma	TCGA-CM-6161	0	0	0	0	0
colorectal adenocarcinoma	TCGA-CM-6162	0	0	0	0	0
colorectal adenocarcinoma	TCGA-CM-6163	1	1	1	1	1
colorectal adenocarcinoma	TCGA-CM-6164	1	1	1	1	1
colorectal adenocarcinoma	TCGA-CM-6165	1	0	0	1	1
colorectal adenocarcinoma	TCGA-CM-6166	1	0	1	0	1
colorectal adenocarcinoma	TCGA-CM-6167	0	0	1	0	1
colorectal adenocarcinoma	TCGA-CM-6168	0	0	0	0	0
colorectal adenocarcinoma	TCGA-CM-6169	1	1	1	1	1
colorectal adenocarcinoma	TCGA-CM-6170	1	1	1	1	1
colorectal adenocarcinoma	TCGA-CM-6171	0	0	0	0	0
colorectal adenocarcinoma	TCGA-CM-6172	1	1	1	1	1
colorectal adenocarcinoma	TCGA-CM-6674	0	0	0	0	0
colorectal adenocarcinoma	TCGA-CM-6675	1	1	1	0	1
colorectal adenocarcinoma	TCGA-CM-6676	1	1	1	1	1
colorectal adenocarcinoma	TCGA-CM-6677	1	0	1	1	1
colorectal adenocarcinoma	TCGA-CM-6678	1	1	1	1	1
colorectal adenocarcinoma	TCGA-CM-6679	1	1	1	1	1
colorectal adenocarcinoma	TCGA-CM-6680	1	1	0	1	1
colorectal adenocarcinoma	TCGA-D5-5537	1	1	1	1	1
colorectal adenocarcinoma	TCGA-D5-5538	1	1	1	0	1
colorectal adenocarcinoma	TCGA-D5-5539	1	0	1	1	1
colorectal adenocarcinoma	TCGA-D5-5540	1	1	1	1	1
colorectal adenocarcinoma	TCGA-D5-5541	1	0	1	1	1
colorectal adenocarcinoma	TCGA-D5-6529	1	1	1	1	1
colorectal adenocarcinoma	TCGA-D5-6530	0	0	0	0	0
colorectal adenocarcinoma	TCGA-D5-6531	1	1	1	1	1
colorectal adenocarcinoma	TCGA-D5-6532	1	0	1	0	1
colorectal adenocarcinoma	TCGA-D5-6533	1	1	1	1	1
colorectal adenocarcinoma	TCGA-D5-6534	1	1	1	1	1
colorectal adenocarcinoma	TCGA-D5-6535	1	1	1	1	1
colorectal adenocarcinoma	TCGA-D5-6536	1	1	1	1	1
colorectal adenocarcinoma	TCGA-D5-6537	1	1	1	0	0
colorectal adenocarcinoma	TCGA-D5-6538	1	0	1	0	1
colorectal adenocarcinoma	TCGA-D5-6539	1	1	1	1	1
colorectal adenocarcinoma	TCGA-D5-6540	0	0	0	0	0
colorectal adenocarcinoma	TCGA-D5-6541	1	1	1	1	1
colorectal adenocarcinoma	TCGA-D5-6898	1	1	1	1	1

colorectal adenocarcinoma	TCGA-D5-6920	0	0	0	0	0
colorectal adenocarcinoma	TCGA-D5-6922	1	1	1	1	1
colorectal adenocarcinoma	TCGA-D5-6923	1	1	1	1	1
colorectal adenocarcinoma	TCGA-D5-6924	1	1	1	1	1
colorectal adenocarcinoma	TCGA-D5-6926	1	1	1	1	1
colorectal adenocarcinoma	TCGA-D5-6927	0	0	0	0	0
colorectal adenocarcinoma	TCGA-D5-6928	0	0	0	0	0
colorectal adenocarcinoma	TCGA-D5-6929	1	1	1	1	1
colorectal adenocarcinoma	TCGA-D5-6930	0	0	0	0	0
colorectal adenocarcinoma	TCGA-D5-6931	0	0	1	0	0
colorectal adenocarcinoma	TCGA-D5-6932	1	1	1	1	1
colorectal adenocarcinoma	TCGA-D5-7000	0	0	0	0	0
colorectal adenocarcinoma	TCGA-DC-4745	1	1	1	1	1
colorectal adenocarcinoma	TCGA-DC-4749	1	1	1	1	1
colorectal adenocarcinoma	TCGA-DC-5337	1	1	0	0	0
colorectal adenocarcinoma	TCGA-DC-5869	1	1	1	1	1
colorectal adenocarcinoma	TCGA-DC-6154	1	1	0	1	0
colorectal adenocarcinoma	TCGA-DC-6155	1	1	1	1	1
colorectal adenocarcinoma	TCGA-DC-6156	1	1	1	1	1
colorectal adenocarcinoma	TCGA-DC-6157	1	1	1	1	1
colorectal adenocarcinoma	TCGA-DC-6158	1	1	1	1	1
colorectal adenocarcinoma	TCGA-DC-6160	1	1	1	1	1
colorectal adenocarcinoma	TCGA-DC-6681	1	1	0	1	0
colorectal adenocarcinoma	TCGA-DC-6682	1	1	1	1	1
colorectal adenocarcinoma	TCGA-DC-6683	1	1	1	1	1
colorectal adenocarcinoma	TCGA-DM-A0X9	1	0	1	0	0
colorectal adenocarcinoma	TCGA-DM-A0XD	1	0	1	0	1
colorectal adenocarcinoma	TCGA-DM-A0XF	1	1	1	1	1
colorectal adenocarcinoma	TCGA-DM-A1D0	1	1	1	1	1
colorectal adenocarcinoma	TCGA-DM-A1D4	1	1	1	1	1
colorectal adenocarcinoma	TCGA-DM-A1D6	1	1	0	1	1
colorectal adenocarcinoma	TCGA-DM-A1D7	1	0	1	1	1
colorectal adenocarcinoma	TCGA-DM-A1D8	1	1	1	1	1
colorectal adenocarcinoma	TCGA-DM-A1D9	1	1	1	1	1
colorectal adenocarcinoma	TCGA-DM-A1DA	1	1	0	1	1
colorectal adenocarcinoma	TCGA-DM-A1DB	1	1	1	1	1
colorectal adenocarcinoma	TCGA-DM-A1HA	1	1	1	1	1
colorectal adenocarcinoma	TCGA-DM-A1HB	0	0	0	0	0
colorectal adenocarcinoma	TCGA-DM-A280	0	0	0	0	0
colorectal adenocarcinoma	TCGA-DM-A282	1	1	1	1	1
colorectal adenocarcinoma	TCGA-DM-A285	1	1	1	1	1
colorectal adenocarcinoma	TCGA-DM-A288	1	1	1	1	1
colorectal adenocarcinoma	TCGA-DM-A28A	1	1	1	1	1
colorectal adenocarcinoma	TCGA-DM-A28C	0	0	0	0	0
colorectal adenocarcinoma	TCGA-DM-A28E	1	0	1	0	1
colorectal adenocarcinoma	TCGA-DM-A28F	1	1	1	0	1
colorectal adenocarcinoma	TCGA-DM-A28G	0	1	0	0	0
colorectal adenocarcinoma	TCGA-DM-A28H	1	1	1	1	1
colorectal adenocarcinoma	TCGA-DM-A28K	1	0	0	0	0
colorectal adenocarcinoma	TCGA-DM-A28M	1	1	0	1	1
colorectal adenocarcinoma	TCGA-DT-5265	1	1	1	1	1
colorectal adenocarcinoma	TCGA-DY-A0XA	1	1	1	1	1
colorectal adenocarcinoma	TCGA-DY-A1DC	1	0	1	1	1
colorectal adenocarcinoma	TCGA-DY-A1DD	1	1	1	1	1
colorectal adenocarcinoma	TCGA-DY-A1DE	1	1	1	1	1
colorectal adenocarcinoma	TCGA-DY-A1DF	1	1	1	1	1
colorectal adenocarcinoma	TCGA-DY-A1DG	1	1	1	1	1
colorectal adenocarcinoma	TCGA-DY-A1H8	1	1	0	1	0

colorectal adenocarcinoma	TCGA-EF-5830	1	1	1	1	1
colorectal adenocarcinoma	TCGA-EF-5831	1	1	1	1	1
colorectal adenocarcinoma	TCGA-EI-6506	1	1	1	1	1
colorectal adenocarcinoma	TCGA-EI-6507	0	0	0	0	0
colorectal adenocarcinoma	TCGA-EI-6508	1	1	1	0	1
colorectal adenocarcinoma	TCGA-EI-6509	1	1	1	1	1
colorectal adenocarcinoma	TCGA-EI-6510	1	0	1	1	1
colorectal adenocarcinoma	TCGA-EI-6511	1	1	1	1	1
colorectal adenocarcinoma	TCGA-EI-6512	1	1	1	1	1
colorectal adenocarcinoma	TCGA-EI-6513	1	0	1	0	1
colorectal adenocarcinoma	TCGA-EI-6514	1	1	1	1	1
colorectal adenocarcinoma	TCGA-EI-6881	1	1	1	1	1
colorectal adenocarcinoma	TCGA-EI-6882	0	0	0	0	1
colorectal adenocarcinoma	TCGA-EI-6883	1	1	1	1	1
colorectal adenocarcinoma	TCGA-EI-6884	1	0	1	1	1
colorectal adenocarcinoma	TCGA-EI-6885	1	1	1	1	1
colorectal adenocarcinoma	TCGA-EI-6917	0	0	0	0	0
colorectal adenocarcinoma	TCGA-EI-7002	1	1	1	1	1
colorectal adenocarcinoma	TCGA-EI-7004	1	1	1	1	1
colorectal adenocarcinoma	TCGA-F4-6459	1	1	1	0	1
colorectal adenocarcinoma	TCGA-F4-6460	1	1	1	1	1
colorectal adenocarcinoma	TCGA-F4-6461	1	0	1	1	1
colorectal adenocarcinoma	TCGA-F4-6463	0	0	0	0	0
colorectal adenocarcinoma	TCGA-F4-6569	1	0	0	0	0
colorectal adenocarcinoma	TCGA-F4-6570	0	0	0	0	0
colorectal adenocarcinoma	TCGA-F4-6703	0	0	0	1	1
colorectal adenocarcinoma	TCGA-F4-6704	1	1	1	1	1
colorectal adenocarcinoma	TCGA-F4-6805	1	1	1	0	1
colorectal adenocarcinoma	TCGA-F4-6806	1	1	0	1	0
colorectal adenocarcinoma	TCGA-F4-6807	1	0	1	1	1
colorectal adenocarcinoma	TCGA-F4-6808	1	1	1	1	1
colorectal adenocarcinoma	TCGA-F4-6809	1	1	1	1	1
colorectal adenocarcinoma	TCGA-F4-6854	1	0	0	1	1
colorectal adenocarcinoma	TCGA-F4-6855	1	1	0	1	1
colorectal adenocarcinoma	TCGA-F4-6856	0	0	0	0	0
colorectal adenocarcinoma	TCGA-F5-6464	0	0	1	0	1
colorectal adenocarcinoma	TCGA-F5-6465	1	0	1	0	1
colorectal adenocarcinoma	TCGA-F5-6571	1	1	1	1	1
colorectal adenocarcinoma	TCGA-F5-6702	1	1	1	1	1
colorectal adenocarcinoma	TCGA-F5-6810	1	1	1	1	1
colorectal adenocarcinoma	TCGA-F5-6811	1	1	1	1	1
colorectal adenocarcinoma	TCGA-F5-6812	1	0	1	1	1
colorectal adenocarcinoma	TCGA-F5-6813	1	1	1	1	1
colorectal adenocarcinoma	TCGA-F5-6814	0	1	1	1	1
colorectal adenocarcinoma	TCGA-F5-6861	0	0	0	0	0
colorectal adenocarcinoma	TCGA-F5-6863	1	1	1	1	1
colorectal adenocarcinoma	TCGA-F5-6864	1	1	1	1	1
colorectal adenocarcinoma	TCGA-G4-6293	0	0	0	0	0
colorectal adenocarcinoma	TCGA-G4-6294	1	0	0	0	0
colorectal adenocarcinoma	TCGA-G4-6295	1	1	1	1	1
colorectal adenocarcinoma	TCGA-G4-6297	1	1	1	1	1
colorectal adenocarcinoma	TCGA-G4-6298	1	1	1	1	1
colorectal adenocarcinoma	TCGA-G4-6299	1	1	0	1	1
colorectal adenocarcinoma	TCGA-G4-6302	0	0	0	0	0
colorectal adenocarcinoma	TCGA-G4-6303	1	1	1	1	1
colorectal adenocarcinoma	TCGA-G4-6304	0	0	0	0	0
colorectal adenocarcinoma	TCGA-G4-6306	1	1	1	0	1
colorectal adenocarcinoma	TCGA-G4-6307	1	0	1	1	1

colorectal adenocarcinoma	TCGA-G4-6309	0	0	0	0	0
colorectal adenocarcinoma	TCGA-G4-6310	1	0	1	1	1
colorectal adenocarcinoma	TCGA-G4-6311	1	0	1	0	1
colorectal adenocarcinoma	TCGA-G4-6314	1	1	1	1	1
colorectal adenocarcinoma	TCGA-G4-6315	1	1	1	1	1
colorectal adenocarcinoma	TCGA-G4-6317	1	1	1	1	1
colorectal adenocarcinoma	TCGA-G4-6320	0	0	1	0	1
colorectal adenocarcinoma	TCGA-G4-6321	0	0	0	0	0
colorectal adenocarcinoma	TCGA-G4-6322	1	1	0	0	1
colorectal adenocarcinoma	TCGA-G4-6323	1	0	0	0	0
colorectal adenocarcinoma	TCGA-G4-6586	1	0	1	0	0
colorectal adenocarcinoma	TCGA-G4-6588	0	0	0	0	0
colorectal adenocarcinoma	TCGA-G4-6625	1	1	1	1	1
colorectal adenocarcinoma	TCGA-G4-6626	1	1	1	1	1
colorectal adenocarcinoma	TCGA-G4-6627	1	0	0	0	0
colorectal adenocarcinoma	TCGA-G4-6628	0	0	0	0	0
colorectal adenocarcinoma	TCGA-G5-6233	1	1	1	1	1
colorectal adenocarcinoma	TCGA-G5-6235	1	0	1	0	1
colorectal adenocarcinoma	TCGA-G5-6572	1	1	0	1	1
colorectal adenocarcinoma	TCGA-G5-6641	1	1	1	1	1
colorectal adenocarcinoma	TCGA-NH-A50T	1	1	1	1	1
colorectal adenocarcinoma	TCGA-NH-A50U	1	1	1	1	1
colorectal adenocarcinoma	TCGA-NH-A50V	1	0	1	1	1
colorectal adenocarcinoma	TCGA-NH-A5IV	0	0	0	0	0
colorectal adenocarcinoma	TCGA-NH-A6GA	1	1	1	1	1
colorectal adenocarcinoma	TCGA-NH-A6GB	1	0	1	0	1
colorectal adenocarcinoma	TCGA-NH-A6GC	1	1	1	1	1
colorectal adenocarcinoma	TCGA-NH-A8F7	1	0	1	0	1
colorectal adenocarcinoma	TCGA-NH-A8F8	1	1	1	1	1
colorectal adenocarcinoma	TCGA-QG-A5YV	1	1	1	1	1
colorectal adenocarcinoma	TCGA-QG-A5YW	1	0	1	1	1
colorectal adenocarcinoma	TCGA-QG-A5YX	1	0	1	0	1
colorectal adenocarcinoma	TCGA-QG-A5Z1	1	0	1	0	1
colorectal adenocarcinoma	TCGA-QG-A5Z2	0	0	0	0	0
colorectal adenocarcinoma	TCGA-QL-A97D	1	1	1	1	1
colorectal adenocarcinoma	TCGA-RU-A8FL	1	1	1	1	1
colorectal adenocarcinoma	TCGA-SS-A7HO	1	1	1	1	1
colorectal adenocarcinoma	TCGA-T9-A92H	1	1	1	1	1
colorectal adenocarcinoma	TCGA-WS-AB45	0	0	0	0	0
Endometrial Carcinoma	TCGA-2E-A9G8	1	1	1	1	1
Endometrial Carcinoma	TCGA-4E-A92E	0	0	0	0	0
Endometrial Carcinoma	TCGA-5B-A90C	1	1	1	0	1
Endometrial Carcinoma	TCGA-5S-A9Q8	0	0	0	0	0
Endometrial Carcinoma	TCGA-A5-A1OH	1	1	1	1	1
Endometrial Carcinoma	TCGA-A5-A2K2	1	1	1	1	1
Endometrial Carcinoma	TCGA-A5-A2K3	1	1	1	1	1
Endometrial Carcinoma	TCGA-A5-A2K4	1	1	1	1	1
Endometrial Carcinoma	TCGA-A5-A2K5	0	0	0	0	0
Endometrial Carcinoma	TCGA-A5-A2K7	0	0	0	0	0
Endometrial Carcinoma	TCGA-A5-A3LO	1	1	1	1	1
Endometrial Carcinoma	TCGA-A5-A3LP	1	1	1	1	1
Endometrial Carcinoma	TCGA-A5-A7WJ	0	0	0	0	0
Endometrial Carcinoma	TCGA-A5-A7WK	1	1	1	1	1
Endometrial Carcinoma	TCGA-A5-AB3J	0	0	0	0	0
Endometrial Carcinoma	TCGA-AJ-A23N	1	1	1	1	1
Endometrial Carcinoma	TCGA-AJ-A2QM	1	1	1	1	1
Endometrial Carcinoma	TCGA-AJ-A2QO	0	0	0	0	0
Endometrial Carcinoma	TCGA-AJ-A3BD	1	1	1	1	1

Endometrial Carcinoma	TCGA-AJ-A3BF	1	1	1	1	1
Endometrial Carcinoma	TCGA-AJ-A3BG	0	0	0	0	0
Endometrial Carcinoma	TCGA-AJ-A3BH	0	0	0	0	0
Endometrial Carcinoma	TCGA-AJ-A3BI	1	1	1	1	1
Endometrial Carcinoma	TCGA-AJ-A3BK	0	0	0	0	0
Endometrial Carcinoma	TCGA-AJ-A3EJ	1	1	1	1	1
Endometrial Carcinoma	TCGA-AJ-A3EK	1	0	0	0	0
Endometrial Carcinoma	TCGA-AJ-A3EL	0	0	0	0	0
Endometrial Carcinoma	TCGA-AJ-A3EM	1	1	1	1	1
Endometrial Carcinoma	TCGA-AJ-A3I9	1	1	1	1	1
Endometrial Carcinoma	TCGA-AJ-A3IA	1	1	1	1	1
Endometrial Carcinoma	TCGA-AJ-A3NC	0	0	0	0	0
Endometrial Carcinoma	TCGA-AJ-A3NE	0	0	0	0	0
Endometrial Carcinoma	TCGA-AJ-A3NF	1	1	1	1	1
Endometrial Carcinoma	TCGA-AJ-A3NG	0	0	0	0	0
Endometrial Carcinoma	TCGA-AJ-A3NH	1	1	1	1	1
Endometrial Carcinoma	TCGA-AJ-A3OJ	0	0	0	0	0
Endometrial Carcinoma	TCGA-AJ-A3OK	0	0	0	0	0
Endometrial Carcinoma	TCGA-AJ-A3OL	0	0	0	0	0
Endometrial Carcinoma	TCGA-AJ-A3QS	1	1	1	1	1
Endometrial Carcinoma	TCGA-AJ-A3TW	1	1	1	1	1
Endometrial Carcinoma	TCGA-AJ-A5DV	0	0	0	0	0
Endometrial Carcinoma	TCGA-AJ-A5DW	0	0	0	0	0
Endometrial Carcinoma	TCGA-AJ-A6NU	1	1	1	1	1
Endometrial Carcinoma	TCGA-AJ-A8CT	0	0	0	0	0
Endometrial Carcinoma	TCGA-AJ-A8CV	0	0	0	0	0
Endometrial Carcinoma	TCGA-AJ-A8CW	0	0	0	0	0
Endometrial Carcinoma	TCGA-AP-A3K1	1	1	1	1	1
Endometrial Carcinoma	TCGA-AP-A5FX	1	1	1	1	1
Endometrial Carcinoma	TCGA-AX-A05W	1	0	0	0	0
Endometrial Carcinoma	TCGA-AX-A1C7	1	1	1	1	1
Endometrial Carcinoma	TCGA-AX-A2H4	1	1	1	1	1
Endometrial Carcinoma	TCGA-AX-A2HH	0	0	1	0	1
Endometrial Carcinoma	TCGA-AX-A2IN	0	0	0	0	0
Endometrial Carcinoma	TCGA-AX-A3FS	1	1	0	1	1
Endometrial Carcinoma	TCGA-AX-A3FT	0	0	0	0	0
Endometrial Carcinoma	TCGA-AX-A3FV	1	1	1	1	1
Endometrial Carcinoma	TCGA-AX-A3FW	1	1	1	1	1
Endometrial Carcinoma	TCGA-AX-A3FX	1	1	1	1	1
Endometrial Carcinoma	TCGA-AX-A3FZ	1	1	1	1	1
Endometrial Carcinoma	TCGA-AX-A3G1	1	1	1	1	1
Endometrial Carcinoma	TCGA-AX-A3G3	1	1	1	1	1
Endometrial Carcinoma	TCGA-AX-A3G4	1	1	1	1	1
Endometrial Carcinoma	TCGA-AX-A3G6	1	1	1	1	1
Endometrial Carcinoma	TCGA-AX-A3G7	1	1	1	1	1
Endometrial Carcinoma	TCGA-AX-A3G8	0	0	0	0	0
Endometrial Carcinoma	TCGA-AX-A3G9	0	0	0	0	0
Endometrial Carcinoma	TCGA-AX-A3GB	0	0	0	0	0
Endometrial Carcinoma	TCGA-AX-A3GI	1	1	1	1	1
Endometrial Carcinoma	TCGA-B5-A0JN	1	1	1	1	1
Endometrial Carcinoma	TCGA-B5-A0JR	1	0	0	0	1
Endometrial Carcinoma	TCGA-B5-A0K9	1	0	0	1	0
Endometrial Carcinoma	TCGA-B5-A11R	1	1	0	0	1
Endometrial Carcinoma	TCGA-B5-A1MS	1	1	1	1	1
Endometrial Carcinoma	TCGA-B5-A1MW	0	0	0	0	0
Endometrial Carcinoma	TCGA-B5-A3F9	1	1	1	1	1
Endometrial Carcinoma	TCGA-B5-A3FA	0	0	0	0	0
Endometrial Carcinoma	TCGA-B5-A3FB	1	0	1	1	0

Endometrial Carcinoma	TCGA-B5-A3FC	0	0	0	0	0
Endometrial Carcinoma	TCGA-B5-A3FD	1	1	1	1	1
Endometrial Carcinoma	TCGA-B5-A3FH	0	0	0	0	0
Endometrial Carcinoma	TCGA-B5-A3S1	1	1	1	1	1
Endometrial Carcinoma	TCGA-B5-A5OC	0	0	0	0	0
Endometrial Carcinoma	TCGA-B5-A5OD	1	1	1	1	1
Endometrial Carcinoma	TCGA-B5-A5OE	1	1	1	1	1
Endometrial Carcinoma	TCGA-BG-A0MK	0	0	0	0	0
Endometrial Carcinoma	TCGA-BG-A3EW	0	0	0	0	0
Endometrial Carcinoma	TCGA-BG-A3PP	1	1	1	1	1
Endometrial Carcinoma	TCGA-BK-A139	0	0	0	0	0
Endometrial Carcinoma	TCGA-BK-A13B	0	0	0	0	0
Endometrial Carcinoma	TCGA-BK-A4ZD	1	1	1	1	1
Endometrial Carcinoma	TCGA-BK-A56F	0	0	0	0	0
Endometrial Carcinoma	TCGA-BK-A6W3	0	0	0	0	0
Endometrial Carcinoma	TCGA-BK-A6W4	1	0	0	1	0
Endometrial Carcinoma	TCGA-BS-A0V4	0	0	0	0	0
Endometrial Carcinoma	TCGA-BS-A0V7	1	0	0	0	0
Endometrial Carcinoma	TCGA-D1-A2G0	0	0	0	0	0
Endometrial Carcinoma	TCGA-D1-A3DA	0	0	0	0	0
Endometrial Carcinoma	TCGA-D1-A3DG	1	1	1	1	1
Endometrial Carcinoma	TCGA-D1-A3DH	1	1	1	1	1
Endometrial Carcinoma	TCGA-D1-A3JP	1	1	1	1	1
Endometrial Carcinoma	TCGA-D1-A3JQ	1	1	1	1	1
Endometrial Carcinoma	TCGA-DF-A2KN	0	0	0	0	0
Endometrial Carcinoma	TCGA-DF-A2KR	1	1	1	1	1
Endometrial Carcinoma	TCGA-DF-A2KU	0	0	0	0	0
Endometrial Carcinoma	TCGA-DF-A2KV	0	0	0	0	0
Endometrial Carcinoma	TCGA-DF-A2KY	0	0	0	0	0
Endometrial Carcinoma	TCGA-DF-A2KZ	0	0	0	0	0
Endometrial Carcinoma	TCGA-DF-A2L0	1	1	1	1	1
Endometrial Carcinoma	TCGA-DI-A1BU	0	0	0	0	0
Endometrial Carcinoma	TCGA-DI-A1C3	0	0	0	0	0
Endometrial Carcinoma	TCGA-DI-A2QT	1	1	1	1	1
Endometrial Carcinoma	TCGA-DI-A2QY	1	1	1	1	1
Endometrial Carcinoma	TCGA-E6-A2P8	0	0	0	0	0
Endometrial Carcinoma	TCGA-E6-A2P9	0	0	0	0	0
Endometrial Carcinoma	TCGA-E6-A8L9	1	1	1	1	1
Endometrial Carcinoma	TCGA-EO-A1Y7	1	1	1	1	1
Endometrial Carcinoma	TCGA-EO-A22U	0	0	0	0	0
Endometrial Carcinoma	TCGA-EO-A22X	0	0	1	0	0
Endometrial Carcinoma	TCGA-EO-A22Y	0	0	0	0	0
Endometrial Carcinoma	TCGA-EO-A3AS	1	1	1	1	1
Endometrial Carcinoma	TCGA-EO-A3AU	0	0	0	0	0
Endometrial Carcinoma	TCGA-EO-A3AV	0	0	0	0	0
Endometrial Carcinoma	TCGA-EO-A3AY	0	0	0	0	0
Endometrial Carcinoma	TCGA-EO-A3AZ	1	1	1	1	1
Endometrial Carcinoma	TCGA-EO-A3B0	0	0	0	0	0
Endometrial Carcinoma	TCGA-EO-A3B1	1	1	1	1	1
Endometrial Carcinoma	TCGA-EO-A3KU	1	1	1	1	1
Endometrial Carcinoma	TCGA-EO-A3KW	1	1	1	1	1
Endometrial Carcinoma	TCGA-EO-A3KX	0	0	0	0	0
Endometrial Carcinoma	TCGA-EO-A3L0	0	0	0	0	0
Endometrial Carcinoma	TCGA-EY-A1GL	0	0	0	0	0
Endometrial Carcinoma	TCGA-EY-A1GO	1	1	1	1	1
Endometrial Carcinoma	TCGA-EY-A1GP	1	1	1	1	1
Endometrial Carcinoma	TCGA-EY-A1GX	0	0	0	0	0
Endometrial Carcinoma	TCGA-EY-A210	1	1	1	1	1

Endometrial Carcinoma	TCGA-EY-A2ON	1	1	1	1	1
Endometrial Carcinoma	TCGA-EY-A2OO	1	1	0	1	1
Endometrial Carcinoma	TCGA-EY-A2OP	0	0	0	0	0
Endometrial Carcinoma	TCGA-EY-A2OQ	1	1	1	1	1
Endometrial Carcinoma	TCGA-EY-A3L3	1	1	1	1	1
Endometrial Carcinoma	TCGA-EY-A3QX	1	1	1	1	1
Endometrial Carcinoma	TCGA-EY-A4KR	1	1	1	1	1
Endometrial Carcinoma	TCGA-EY-A547	1	1	1	1	1
Endometrial Carcinoma	TCGA-EY-A548	0	0	0	0	0
Endometrial Carcinoma	TCGA-EY-A549	0	0	0	0	0
Endometrial Carcinoma	TCGA-EY-A54A	1	1	1	1	1
Endometrial Carcinoma	TCGA-EY-A5W2	0	0	0	0	0
Endometrial Carcinoma	TCGA-EY-A72D	1	1	1	1	1
Endometrial Carcinoma	TCGA-FI-A2EY	1	1	1	1	1
Endometrial Carcinoma	TCGA-FI-A3PV	1	1	1	1	1
Endometrial Carcinoma	TCGA-FI-A3PX	1	1	1	1	1
Endometrial Carcinoma	TCGA-H5-A2HR	1	1	1	1	1
Endometrial Carcinoma	TCGA-JU-AAVI	1	1	1	1	1
Endometrial Carcinoma	TCGA-K6-A3WQ	1	1	1	1	1
Endometrial Carcinoma	TCGA-KJ-A3U4	1	1	1	1	1
Endometrial Carcinoma	TCGA-KP-A3VZ	1	1	1	1	1
Endometrial Carcinoma	TCGA-KP-A3W0	1	1	1	1	1
Endometrial Carcinoma	TCGA-KP-A3W1	1	1	1	1	1
Endometrial Carcinoma	TCGA-KP-A3W3	1	1	1	1	1
Endometrial Carcinoma	TCGA-KP-A3W4	1	1	1	1	1
Endometrial Carcinoma	TCGA-PG-A5BC	1	1	1	1	1
Endometrial Carcinoma	TCGA-PG-A6IB	0	0	0	0	0
Endometrial Carcinoma	TCGA-PG-A7D5	1	1	1	1	1
Endometrial Carcinoma	TCGA-PG-A914	1	1	1	1	1
Endometrial Carcinoma	TCGA-PG-A915	1	1	1	1	1
Endometrial Carcinoma	TCGA-PG-A916	0	0	0	0	0
Endometrial Carcinoma	TCGA-PG-A917	0	0	1	0	0
Endometrial Carcinoma	TCGA-QF-A5YS	0	0	0	0	0
Endometrial Carcinoma	TCGA-QF-A5YT	0	0	0	0	0
Endometrial Carcinoma	TCGA-QS-A5YQ	0	0	0	0	0
Endometrial Carcinoma	TCGA-QS-A5YR	0	0	0	0	0
Endometrial Carcinoma	TCGA-QS-A744	1	0	0	0	0
Endometrial Carcinoma	TCGA-QS-A8F1	1	1	1	1	1
Endometrial Carcinoma	TCGA-SJ-A6ZI	0	0	0	1	1
Endometrial Carcinoma	TCGA-SJ-A6ZJ	0	0	0	0	0
Endometrial Carcinoma	TCGA-SL-A6J9	1	1	1	1	1
Endometrial Carcinoma	TCGA-SL-A6JA	0	0	0	0	0
Esophageal carcinoma	TCGA-2H-A9GF	1	1	1	1	1
Esophageal carcinoma	TCGA-2H-A9GG	0	0	0	0	0
Esophageal carcinoma	TCGA-2H-A9GH	1	1	1	1	1
Esophageal carcinoma	TCGA-2H-A9GI	1	1	1	1	1
Esophageal carcinoma	TCGA-2H-A9GJ	1	1	1	1	1
Esophageal carcinoma	TCGA-2H-A9GK	1	1	1	1	1
Esophageal carcinoma	TCGA-2H-A9GL	1	1	1	1	1
Esophageal carcinoma	TCGA-2H-A9GM	1	1	1	1	1
Esophageal carcinoma	TCGA-2H-A9GN	1	1	1	1	1
Esophageal carcinoma	TCGA-2H-A9GO	1	1	1	1	1
Esophageal carcinoma	TCGA-2H-A9GQ	1	1	1	1	1
Esophageal carcinoma	TCGA-2H-A9GR	1	1	1	1	1
Esophageal carcinoma	TCGA-IC-A6RE	1	1	1	1	1
Esophageal carcinoma	TCGA-IC-A6RF	0	1	0	0	1
Esophageal carcinoma	TCGA-IG-A3I8	1	1	1	1	1
Esophageal carcinoma	TCGA-IG-A3QL	1	1	1	1	1

Esophageal carcinoma	TCGA-IG-A3Y9	1	1	1	1	1
Esophageal carcinoma	TCGA-IG-A3YA	1	1	0	1	1
Esophageal carcinoma	TCGA-IG-A3YB	1	1	1	1	1
Esophageal carcinoma	TCGA-IG-A3YC	1	1	1	1	1
Esophageal carcinoma	TCGA-IG-A4P3	1	1	1	1	1
Esophageal carcinoma	TCGA-IG-A4QS	1	1	1	1	1
Esophageal carcinoma	TCGA-IG-A4QT	0	0	0	0	0
Esophageal carcinoma	TCGA-IG-A50L	1	1	1	1	1
Esophageal carcinoma	TCGA-IG-A51D	1	1	1	1	1
Esophageal carcinoma	TCGA-IG-A5B8	1	1	1	1	1
Esophageal carcinoma	TCGA-IG-A5S3	1	1	1	1	1
Esophageal carcinoma	TCGA-IG-A625	1	1	0	1	1
Esophageal carcinoma	TCGA-IG-A6QS	1	1	0	1	1
Esophageal carcinoma	TCGA-IG-A7DP	1	0	0	0	0
Esophageal carcinoma	TCGA-IG-A8O2	1	1	1	1	1
Esophageal carcinoma	TCGA-IG-A97H	1	1	1	1	1
Esophageal carcinoma	TCGA-IG-A97I	1	1	0	1	1
Esophageal carcinoma	TCGA-JY-A6F8	1	1	1	1	1
Esophageal carcinoma	TCGA-JY-A6FA	1	1	1	1	1
Esophageal carcinoma	TCGA-JY-A6FB	1	1	1	1	1
Esophageal carcinoma	TCGA-JY-A6FD	1	1	1	1	1
Esophageal carcinoma	TCGA-JY-A6FE	1	1	1	1	1
Esophageal carcinoma	TCGA-JY-A6FG	1	1	0	1	1
Esophageal carcinoma	TCGA-JY-A6FH	1	1	1	1	1
Esophageal carcinoma	TCGA-JY-A938	1	1	1	1	1
Esophageal carcinoma	TCGA-JY-A939	1	1	0	1	1
Esophageal carcinoma	TCGA-JY-A93C	1	1	1	1	1
Esophageal carcinoma	TCGA-JY-A93D	1	1	1	1	1
Esophageal carcinoma	TCGA-JY-A93E	1	1	1	1	1
Esophageal carcinoma	TCGA-JY-A93F	1	0	0	0	0
Esophageal carcinoma	TCGA-KH-A6WC	1	0	0	0	1
Esophageal carcinoma	TCGA-L5-A43C	1	1	1	1	1
Esophageal carcinoma	TCGA-L5-A43E	1	1	1	1	1
Esophageal carcinoma	TCGA-L5-A43H	1	1	1	1	1
Esophageal carcinoma	TCGA-L5-A43I	1	1	1	1	1
Esophageal carcinoma	TCGA-L5-A43J	1	1	1	1	1
Esophageal carcinoma	TCGA-L5-A43M	1	1	1	1	1
Esophageal carcinoma	TCGA-L5-A4OE	1	1	1	1	1
Esophageal carcinoma	TCGA-L5-A4OF	1	1	1	1	1
Esophageal carcinoma	TCGA-L5-A4OG	1	1	1	1	1
Esophageal carcinoma	TCGA-L5-A4OH	1	1	1	1	1
Esophageal carcinoma	TCGA-L5-A4OI	0	1	0	1	1
Esophageal carcinoma	TCGA-L5-A4OJ	1	1	1	1	1
Esophageal carcinoma	TCGA-L5-A4OM	1	1	1	1	1
Esophageal carcinoma	TCGA-L5-A4ON	0	1	0	0	1
Esophageal carcinoma	TCGA-L5-A4OO	1	1	1	1	1
Esophageal carcinoma	TCGA-L5-A4OP	1	1	1	1	1
Esophageal carcinoma	TCGA-L5-A4OQ	1	1	1	1	1
Esophageal carcinoma	TCGA-L5-A4OR	1	1	1	1	1
Esophageal carcinoma	TCGA-L5-A4OS	1	1	1	1	1
Esophageal carcinoma	TCGA-L5-A4OT	1	1	1	1	1
Esophageal carcinoma	TCGA-L5-A4OU	1	1	0	1	1
Esophageal carcinoma	TCGA-L5-A4OW	1	1	1	1	1
Esophageal carcinoma	TCGA-L5-A4OX	1	1	1	1	1
Esophageal carcinoma	TCGA-L5-A88S	1	1	1	1	1
Esophageal carcinoma	TCGA-L5-A88T	0	0	0	0	0
Esophageal carcinoma	TCGA-L5-A88V	1	1	1	1	1
Esophageal carcinoma	TCGA-L5-A88W	1	1	0	0	0

Esophageal carcinoma	TCGA-L5-A88Y	1	1	1	1	1
Esophageal carcinoma	TCGA-L5-A88Z	1	1	0	1	1
Esophageal carcinoma	TCGA-L5-A891	1	1	1	1	1
Esophageal carcinoma	TCGA-L5-A893	1	1	1	1	1
Esophageal carcinoma	TCGA-L5-A8NE	1	1	1	1	1
Esophageal carcinoma	TCGA-L5-A8NF	1	1	1	1	1
Esophageal carcinoma	TCGA-L5-A8NG	1	1	1	1	1
Esophageal carcinoma	TCGA-L5-A8NH	1	1	1	1	1
Esophageal carcinoma	TCGA-L5-A8NI	1	1	1	1	1
Esophageal carcinoma	TCGA-L5-A8NJ	1	1	1	1	1
Esophageal carcinoma	TCGA-L5-A8NK	1	1	1	1	1
Esophageal carcinoma	TCGA-L5-A8NL	1	1	1	1	1
Esophageal carcinoma	TCGA-L5-A8NM	1	1	1	0	1
Esophageal carcinoma	TCGA-L5-A8NN	1	1	1	1	1
Esophageal carcinoma	TCGA-L5-A8NQ	1	0	1	0	1
Esophageal carcinoma	TCGA-L5-A8NR	1	1	1	1	1
Esophageal carcinoma	TCGA-L5-A8NS	1	1	1	1	1
Esophageal carcinoma	TCGA-L5-A8NT	1	1	1	1	1
Esophageal carcinoma	TCGA-L5-A8NU	1	1	1	1	1
Esophageal carcinoma	TCGA-L5-A8NV	1	1	1	1	1
Esophageal carcinoma	TCGA-L5-A8NW	1	1	1	1	1
Esophageal carcinoma	TCGA-L7-A56G	1	1	1	1	1
Esophageal carcinoma	TCGA-L7-A6VZ	1	1	1	1	1
Esophageal carcinoma	TCGA-LN-A49K	1	1	1	1	1
Esophageal carcinoma	TCGA-LN-A49L	1	1	1	1	1
Esophageal carcinoma	TCGA-LN-A49M	1	1	1	1	1
Esophageal carcinoma	TCGA-LN-A49N	1	1	1	1	1
Esophageal carcinoma	TCGA-LN-A49O	1	0	1	1	1
Esophageal carcinoma	TCGA-LN-A49P	1	1	0	1	1
Esophageal carcinoma	TCGA-LN-A49R	1	1	1	1	1
Esophageal carcinoma	TCGA-LN-A49S	1	1	1	1	1
Esophageal carcinoma	TCGA-LN-A49U	1	1	1	1	1
Esophageal carcinoma	TCGA-LN-A49V	1	1	1	1	1
Esophageal carcinoma	TCGA-LN-A49W	1	1	1	1	1
Esophageal carcinoma	TCGA-LN-A49X	0	1	1	1	1
Esophageal carcinoma	TCGA-LN-A49Y	1	1	1	1	1
Esophageal carcinoma	TCGA-LN-A4A1	1	1	1	1	1
Esophageal carcinoma	TCGA-LN-A4A2	1	1	1	1	1
Esophageal carcinoma	TCGA-LN-A4A3	1	1	1	1	1
Esophageal carcinoma	TCGA-LN-A4A4	1	0	0	1	1
Esophageal carcinoma	TCGA-LN-A4A5	1	1	1	1	1
Esophageal carcinoma	TCGA-LN-A4A6	1	1	1	1	1
Esophageal carcinoma	TCGA-LN-A4A8	1	1	1	1	1
Esophageal carcinoma	TCGA-LN-A4A9	0	1	0	1	1
Esophageal carcinoma	TCGA-LN-A4MQ	1	1	1	1	1
Esophageal carcinoma	TCGA-LN-A4MR	1	1	1	1	1
Esophageal carcinoma	TCGA-LN-A5U5	1	1	1	1	1
Esophageal carcinoma	TCGA-LN-A5U6	1	1	1	1	1
Esophageal carcinoma	TCGA-LN-A5U7	1	1	1	1	1
Esophageal carcinoma	TCGA-LN-A7HV	1	1	1	1	1
Esophageal carcinoma	TCGA-LN-A7HW	1	1	1	1	1
Esophageal carcinoma	TCGA-LN-A7HX	1	1	1	1	1
Esophageal carcinoma	TCGA-LN-A7HY	1	1	1	1	1
Esophageal carcinoma	TCGA-LN-A7HZ	1	1	1	1	1
Esophageal carcinoma	TCGA-LN-A8HZ	1	1	1	1	1
Esophageal carcinoma	TCGA-LN-A8I0	1	1	1	1	1
Esophageal carcinoma	TCGA-LN-A8I1	1	1	1	1	1
Esophageal carcinoma	TCGA-LN-A9FO	1	1	1	1	1

Esophageal carcinoma	TCGA-LN-A9FP	1	1	0	1	1
Esophageal carcinoma	TCGA-LN-A9FQ	1	1	1	1	1
Esophageal carcinoma	TCGA-LN-A9FR	1	1	1	1	1
Esophageal carcinoma	TCGA-M9-A5M8	1	1	1	1	1
Esophageal carcinoma	TCGA-Q9-A6FU	1	1	1	1	1
Esophageal carcinoma	TCGA-Q9-A6FW	1	1	1	1	1
Esophageal carcinoma	TCGA-R6-A6DN	1	1	1	1	1
Esophageal carcinoma	TCGA-R6-A6DQ	1	1	1	1	1
Esophageal carcinoma	TCGA-R6-A6KZ	1	1	1	1	1
Esophageal carcinoma	TCGA-R6-A6L4	1	1	0	1	1
Esophageal carcinoma	TCGA-R6-A6L6	1	0	1	1	1
Esophageal carcinoma	TCGA-R6-A6XG	1	1	1	1	1
Esophageal carcinoma	TCGA-R6-A6XQ	1	1	1	1	1
Esophageal carcinoma	TCGA-R6-A6Y0	1	1	1	1	1
Esophageal carcinoma	TCGA-R6-A6Y2	1	1	1	1	1
Esophageal carcinoma	TCGA-R6-A8W5	1	1	1	1	1
Esophageal carcinoma	TCGA-R6-A8W8	1	1	1	1	1
Esophageal carcinoma	TCGA-R6-A8WC	1	1	1	1	1
Esophageal carcinoma	TCGA-R6-A8WG	1	1	1	1	1
Esophageal carcinoma	TCGA-RE-A7BO	1	1	1	1	1
Esophageal carcinoma	TCGA-S8-A6BV	1	1	1	1	1
Esophageal carcinoma	TCGA-S8-A6BW	1	1	1	1	1
Esophageal carcinoma	TCGA-V5-A7RB	1	1	0	1	1
Esophageal carcinoma	TCGA-V5-A7RC	1	1	1	1	1
Esophageal carcinoma	TCGA-V5-A7RC	1	1	1	0	1
Esophageal carcinoma	TCGA-V5-A7RE	1	1	1	1	1
Esophageal carcinoma	TCGA-V5-AASV	1	1	1	1	1
Esophageal carcinoma	TCGA-V5-AASW	1	1	1	1	1
Esophageal carcinoma	TCGA-V5-AASX	1	1	1	1	1
Esophageal carcinoma	TCGA-VR-A8EO	1	1	1	1	1
Esophageal carcinoma	TCGA-VR-A8EP	1	1	1	1	1
Esophageal carcinoma	TCGA-VR-A8EQ	1	1	1	1	1
Esophageal carcinoma	TCGA-VR-A8ER	1	1	1	1	1
Esophageal carcinoma	TCGA-VR-A8ET	1	1	1	1	1
Esophageal carcinoma	TCGA-VR-A8EU	1	1	1	1	1
Esophageal carcinoma	TCGA-VR-A8EW	1	1	1	1	1
Esophageal carcinoma	TCGA-VR-A8EX	1	1	1	1	1
Esophageal carcinoma	TCGA-VR-A8EY	0	1	0	1	1
Esophageal carcinoma	TCGA-VR-A8EZ	1	1	1	1	1
Esophageal carcinoma	TCGA-VR-A8Q7	1	1	1	1	1
Esophageal carcinoma	TCGA-VR-AA4D	1	1	1	1	1
Esophageal carcinoma	TCGA-VR-AA4G	1	1	0	1	1
Esophageal carcinoma	TCGA-VR-AA7B	1	1	1	1	1
Esophageal carcinoma	TCGA-VR-AA7D	1	1	1	1	1
Esophageal carcinoma	TCGA-VR-AA7I	1	1	1	1	1
Esophageal carcinoma	TCGA-X8-AAAR	1	1	1	1	1
Esophageal carcinoma	TCGA-XP-A8T6	1	1	1	1	1
Esophageal carcinoma	TCGA-XP-A8T8	1	1	1	1	1
Esophageal carcinoma	TCGA-Z6-A8JD	1	1	1	1	1
Esophageal carcinoma	TCGA-Z6-A8JE	1	1	1	1	1
Esophageal carcinoma	TCGA-Z6-A9VB	1	1	1	1	1
Esophageal carcinoma	TCGA-Z6-AAPN	1	1	1	1	1
Esophageal carcinoma	TCGA-ZR-A9CJ	1	1	1	1	1
Glioblastoma Multiforme	TCGA-02-0047	1	0	0	1	1
Glioblastoma Multiforme	TCGA-02-0055	1	1	1	1	1
Glioblastoma Multiforme	TCGA-02-2483	1	1	1	1	1
Glioblastoma Multiforme	TCGA-02-2485	1	1	0	0	1
Glioblastoma Multiforme	TCGA-02-2486	1	1	0	1	1

Glioblastoma Multiforme	TCGA-06-0125	1	1	0	0	1
Glioblastoma Multiforme	TCGA-06-0125	0	0	0	0	0
Glioblastoma Multiforme	TCGA-06-0129	1	0	1	1	1
Glioblastoma Multiforme	TCGA-06-0130	1	1	1	1	1
Glioblastoma Multiforme	TCGA-06-0132	1	1	0	1	1
Glioblastoma Multiforme	TCGA-06-0138	1	0	1	0	1
Glioblastoma Multiforme	TCGA-06-0141	1	1	1	1	1
Glioblastoma Multiforme	TCGA-06-0152	0	0	0	0	0
Glioblastoma Multiforme	TCGA-06-0156	0	0	0	0	0
Glioblastoma Multiforme	TCGA-06-0157	1	1	0	0	1
Glioblastoma Multiforme	TCGA-06-0158	1	1	1	0	1
Glioblastoma Multiforme	TCGA-06-0168	1	1	0	1	1
Glioblastoma Multiforme	TCGA-06-0171	0	0	0	0	0
Glioblastoma Multiforme	TCGA-06-0174	1	0	0	1	1
Glioblastoma Multiforme	TCGA-06-0178	0	0	0	0	0
Glioblastoma Multiforme	TCGA-06-0184	1	1	1	0	1
Glioblastoma Multiforme	TCGA-06-0187	1	1	1	0	1
Glioblastoma Multiforme	TCGA-06-0190	1	1	1	0	1
Glioblastoma Multiforme	TCGA-06-0190	0	0	0	0	0
Glioblastoma Multiforme	TCGA-06-0210	1	1	0	0	1
Glioblastoma Multiforme	TCGA-06-0210	0	0	0	0	0
Glioblastoma Multiforme	TCGA-06-0211	1	1	0	0	1
Glioblastoma Multiforme	TCGA-06-0211	0	0	0	0	0
Glioblastoma Multiforme	TCGA-06-0219	1	1	0	1	1
Glioblastoma Multiforme	TCGA-06-0221	0	0	0	0	0
Glioblastoma Multiforme	TCGA-06-0238	1	0	0	0	1
Glioblastoma Multiforme	TCGA-06-0644	1	1	1	1	1
Glioblastoma Multiforme	TCGA-06-0645	1	1	0	1	1
Glioblastoma Multiforme	TCGA-06-0646	1	1	1	1	1
Glioblastoma Multiforme	TCGA-06-0649	1	1	1	1	1
Glioblastoma Multiforme	TCGA-06-0686	1	1	1	1	1
Glioblastoma Multiforme	TCGA-06-0743	1	1	0	1	1
Glioblastoma Multiforme	TCGA-06-0744	1	1	1	1	1
Glioblastoma Multiforme	TCGA-06-0745	1	1	1	1	1
Glioblastoma Multiforme	TCGA-06-0747	1	1	0	0	1
Glioblastoma Multiforme	TCGA-06-0749	1	1	0	1	1
Glioblastoma Multiforme	TCGA-06-0750	1	1	1	0	1
Glioblastoma Multiforme	TCGA-06-0878	1	1	0	1	1
Glioblastoma Multiforme	TCGA-06-0882	1	1	1	1	1
Glioblastoma Multiforme	TCGA-06-1804	1	1	0	1	1
Glioblastoma Multiforme	TCGA-06-2557	1	1	1	1	1
Glioblastoma Multiforme	TCGA-06-2558	1	0	0	1	1
Glioblastoma Multiforme	TCGA-06-2559	1	0	0	1	1
Glioblastoma Multiforme	TCGA-06-2561	1	1	1	1	1
Glioblastoma Multiforme	TCGA-06-2562	1	1	0	1	1
Glioblastoma Multiforme	TCGA-06-2563	0	1	0	0	1
Glioblastoma Multiforme	TCGA-06-2564	1	0	0	0	1
Glioblastoma Multiforme	TCGA-06-2565	1	1	0	0	1
Glioblastoma Multiforme	TCGA-06-2567	1	1	0	1	1
Glioblastoma Multiforme	TCGA-06-2569	1	1	1	1	1
Glioblastoma Multiforme	TCGA-06-2570	1	1	1	0	1
Glioblastoma Multiforme	TCGA-06-5408	1	1	1	1	1
Glioblastoma Multiforme	TCGA-06-5410	1	1	1	1	1
Glioblastoma Multiforme	TCGA-06-5411	1	1	1	1	1
Glioblastoma Multiforme	TCGA-06-5412	1	1	0	0	1
Glioblastoma Multiforme	TCGA-06-5413	1	1	1	1	1
Glioblastoma Multiforme	TCGA-06-5414	1	1	0	0	1
Glioblastoma Multiforme	TCGA-06-5415	1	1	1	0	1

Glioblastoma Multiforme	TCGA-06-5416	1	0	1	1	1
Glioblastoma Multiforme	TCGA-06-5417	0	0	0	0	0
Glioblastoma Multiforme	TCGA-06-5418	1	1	0	1	1
Glioblastoma Multiforme	TCGA-06-5856	1	1	1	1	1
Glioblastoma Multiforme	TCGA-06-5858	1	0	0	0	1
Glioblastoma Multiforme	TCGA-06-5859	1	1	1	1	1
Glioblastoma Multiforme	TCGA-08-0386	1	1	1	1	1
Glioblastoma Multiforme	TCGA-12-0616	1	0	1	0	1
Glioblastoma Multiforme	TCGA-12-0618	1	0	0	1	1
Glioblastoma Multiforme	TCGA-12-0619	1	1	1	1	1
Glioblastoma Multiforme	TCGA-12-0821	1	1	0	1	1
Glioblastoma Multiforme	TCGA-12-1597	0	0	0	0	0
Glioblastoma Multiforme	TCGA-12-3650	1	0	0	0	1
Glioblastoma Multiforme	TCGA-12-3652	1	1	0	0	1
Glioblastoma Multiforme	TCGA-12-3653	1	1	1	1	1
Glioblastoma Multiforme	TCGA-12-5295	1	1	1	1	1
Glioblastoma Multiforme	TCGA-12-5299	1	0	1	0	1
Glioblastoma Multiforme	TCGA-14-0736	0	0	0	0	0
Glioblastoma Multiforme	TCGA-14-0781	1	0	0	0	1
Glioblastoma Multiforme	TCGA-14-0787	1	1	0	0	1
Glioblastoma Multiforme	TCGA-14-0789	1	0	0	0	1
Glioblastoma Multiforme	TCGA-14-0790	1	1	0	0	1
Glioblastoma Multiforme	TCGA-14-0817	1	1	1	1	1
Glioblastoma Multiforme	TCGA-14-1034	1	1	0	1	1
Glioblastoma Multiforme	TCGA-14-1034	0	0	0	0	0
Glioblastoma Multiforme	TCGA-14-1402	0	0	0	0	0
Glioblastoma Multiforme	TCGA-14-1823	1	1	1	1	1
Glioblastoma Multiforme	TCGA-14-1825	1	0	0	0	1
Glioblastoma Multiforme	TCGA-14-1829	1	0	0	0	1
Glioblastoma Multiforme	TCGA-14-2554	1	1	0	0	1
Glioblastoma Multiforme	TCGA-15-0742	1	1	1	1	1
Glioblastoma Multiforme	TCGA-15-1444	1	1	1	0	1
Glioblastoma Multiforme	TCGA-16-0846	1	1	1	1	1
Glioblastoma Multiforme	TCGA-16-1045	1	1	1	1	1
Glioblastoma Multiforme	TCGA-19-0957	0	0	0	0	0
Glioblastoma Multiforme	TCGA-19-1389	0	0	0	0	0
Glioblastoma Multiforme	TCGA-19-1390	1	1	1	1	1
Glioblastoma Multiforme	TCGA-19-1787	0	0	1	0	0
Glioblastoma Multiforme	TCGA-19-2619	1	1	1	1	1
Glioblastoma Multiforme	TCGA-19-2620	1	1	0	1	1
Glioblastoma Multiforme	TCGA-19-2624	1	0	0	0	1
Glioblastoma Multiforme	TCGA-19-2625	1	1	1	1	1
Glioblastoma Multiforme	TCGA-19-2629	1	1	1	1	1
Glioblastoma Multiforme	TCGA-19-4065	1	1	1	0	1
Glioblastoma Multiforme	TCGA-19-4065	0	0	0	0	0
Glioblastoma Multiforme	TCGA-19-5960	1	0	1	1	1
Glioblastoma Multiforme	TCGA-26-1442	0	1	1	0	1
Glioblastoma Multiforme	TCGA-26-5132	1	0	1	1	1
Glioblastoma Multiforme	TCGA-26-5133	1	1	1	1	1
Glioblastoma Multiforme	TCGA-26-5134	1	0	0	0	1
Glioblastoma Multiforme	TCGA-26-5135	1	0	1	0	1
Glioblastoma Multiforme	TCGA-26-5136	1	1	1	1	1
Glioblastoma Multiforme	TCGA-26-5139	1	1	0	0	1
Glioblastoma Multiforme	TCGA-27-1830	1	0	0	1	1
Glioblastoma Multiforme	TCGA-27-1831	1	0	0	0	1
Glioblastoma Multiforme	TCGA-27-1832	1	0	1	1	1
Glioblastoma Multiforme	TCGA-27-1834	1	1	1	1	1
Glioblastoma Multiforme	TCGA-27-1835	1	1	1	1	1

Glioblastoma Multiforme	TCGA-27-1837	1	1	0	0	1
Glioblastoma Multiforme	TCGA-27-2519	1	1	0	1	1
Glioblastoma Multiforme	TCGA-27-2521	1	1	1	1	1
Glioblastoma Multiforme	TCGA-27-2523	1	0	0	0	1
Glioblastoma Multiforme	TCGA-27-2524	1	1	1	1	1
Glioblastoma Multiforme	TCGA-27-2526	1	1	0	0	1
Glioblastoma Multiforme	TCGA-27-2528	1	1	0	0	1
Glioblastoma Multiforme	TCGA-28-1747	1	0	0	0	1
Glioblastoma Multiforme	TCGA-28-1753	1	1	0	0	1
Glioblastoma Multiforme	TCGA-28-2499	0	0	0	0	0
Glioblastoma Multiforme	TCGA-28-2509	1	1	1	1	1
Glioblastoma Multiforme	TCGA-28-2510	1	0	0	0	1
Glioblastoma Multiforme	TCGA-28-2513	1	1	0	0	1
Glioblastoma Multiforme	TCGA-28-2514	1	1	1	1	1
Glioblastoma Multiforme	TCGA-28-5204	1	1	1	1	1
Glioblastoma Multiforme	TCGA-28-5207	1	0	1	1	1
Glioblastoma Multiforme	TCGA-28-5208	1	1	0	1	1
Glioblastoma Multiforme	TCGA-28-5209	1	1	1	0	1
Glioblastoma Multiforme	TCGA-28-5213	1	1	1	1	1
Glioblastoma Multiforme	TCGA-28-5215	1	1	1	1	1
Glioblastoma Multiforme	TCGA-28-5216	1	1	0	1	1
Glioblastoma Multiforme	TCGA-28-5218	0	0	0	0	1
Glioblastoma Multiforme	TCGA-28-5220	1	1	1	0	1
Glioblastoma Multiforme	TCGA-32-1970	1	1	0	1	1
Glioblastoma Multiforme	TCGA-32-1980	1	0	1	0	1
Glioblastoma Multiforme	TCGA-32-1982	1	1	0	0	1
Glioblastoma Multiforme	TCGA-32-2615	1	1	1	1	1
Glioblastoma Multiforme	TCGA-32-2616	1	0	1	1	1
Glioblastoma Multiforme	TCGA-32-2632	1	0	0	1	1
Glioblastoma Multiforme	TCGA-32-2634	1	1	0	1	1
Glioblastoma Multiforme	TCGA-32-2638	1	0	0	0	1
Glioblastoma Multiforme	TCGA-32-4213	1	1	0	0	1
Glioblastoma Multiforme	TCGA-32-5222	1	1	0	0	1
Glioblastoma Multiforme	TCGA-41-2571	1	0	1	1	1
Glioblastoma Multiforme	TCGA-41-2572	1	1	1	0	1
Glioblastoma Multiforme	TCGA-41-3915	1	1	0	1	1
Glioblastoma Multiforme	TCGA-41-4097	1	1	1	1	1
Glioblastoma Multiforme	TCGA-41-5651	1	1	1	1	1
Glioblastoma Multiforme	TCGA-76-4925	1	1	1	1	1
Glioblastoma Multiforme	TCGA-76-4926	1	0	0	1	1
Glioblastoma Multiforme	TCGA-76-4927	0	0	0	0	0
Glioblastoma Multiforme	TCGA-76-4928	1	1	1	1	1
Glioblastoma Multiforme	TCGA-76-4929	1	1	1	1	1
Glioblastoma Multiforme	TCGA-76-4931	1	1	1	0	1
Glioblastoma Multiforme	TCGA-76-4932	0	0	0	0	0
Head and Neck squamous	TCGA-4P-AA8J	1	1	1	1	1
Head and Neck squamous	TCGA-BA-4074	1	1	1	1	1
Head and Neck squamous	TCGA-BA-4075	1	1	1	1	1
Head and Neck squamous	TCGA-BA-4076	1	1	1	1	1
Head and Neck squamous	TCGA-BA-4077	1	1	1	1	1
Head and Neck squamous	TCGA-BA-4078	1	1	1	1	1
Head and Neck squamous	TCGA-BA-5149	1	1	1	1	1
Head and Neck squamous	TCGA-BA-5151	1	1	1	1	1
Head and Neck squamous	TCGA-BA-5152	1	1	1	1	1
Head and Neck squamous	TCGA-BA-5153	1	0	1	1	1
Head and Neck squamous	TCGA-BA-5555	1	1	1	1	1
Head and Neck squamous	TCGA-BA-5556	0	0	0	0	1
Head and Neck squamous	TCGA-BA-5557	1	1	1	0	1

Head and Neck squamous	TCGA-BA-5558	1	1	0	1	1
Head and Neck squamous	TCGA-BA-5559	1	1	1	1	1
Head and Neck squamous	TCGA-BA-6868	1	1	1	1	1
Head and Neck squamous	TCGA-BA-6869	1	1	0	1	1
Head and Neck squamous	TCGA-BA-6870	1	1	1	1	1
Head and Neck squamous	TCGA-BA-6871	1	1	1	1	1
Head and Neck squamous	TCGA-BA-6872	1	1	1	1	1
Head and Neck squamous	TCGA-BA-6873	1	1	1	1	1
Head and Neck squamous	TCGA-BA-7269	1	1	1	1	1
Head and Neck squamous	TCGA-BA-A4IF	1	1	1	1	1
Head and Neck squamous	TCGA-BA-A4IG	1	1	1	1	1
Head and Neck squamous	TCGA-BA-A4IH	1	1	1	1	1
Head and Neck squamous	TCGA-BA-A4II	1	1	1	1	1
Head and Neck squamous	TCGA-BA-A6D8	1	1	1	1	1
Head and Neck squamous	TCGA-BA-A6DA	1	0	1	1	1
Head and Neck squamous	TCGA-BA-A6DB	1	1	1	1	1
Head and Neck squamous	TCGA-BA-A6DD	1	1	0	1	1
Head and Neck squamous	TCGA-BA-A6DE	1	1	1	1	1
Head and Neck squamous	TCGA-BA-A6DG	0	0	0	0	1
Head and Neck squamous	TCGA-BA-A6DI	1	1	1	1	1
Head and Neck squamous	TCGA-BA-A6DJ	1	1	0	0	1
Head and Neck squamous	TCGA-BA-A6DL	1	1	1	1	1
Head and Neck squamous	TCGA-BA-A8YP	1	1	1	1	1
Head and Neck squamous	TCGA-BB-4217	1	1	1	0	1
Head and Neck squamous	TCGA-BB-4223	1	1	1	1	1
Head and Neck squamous	TCGA-BB-4224	1	1	1	1	1
Head and Neck squamous	TCGA-BB-4225	1	0	1	1	1
Head and Neck squamous	TCGA-BB-4227	1	1	1	1	1
Head and Neck squamous	TCGA-BB-4228	1	1	0	0	1
Head and Neck squamous	TCGA-BB-7861	1	0	0	1	1
Head and Neck squamous	TCGA-BB-7862	1	1	1	1	1
Head and Neck squamous	TCGA-BB-7863	0	1	0	1	1
Head and Neck squamous	TCGA-BB-7864	0	0	0	1	0
Head and Neck squamous	TCGA-BB-7866	1	0	1	1	1
Head and Neck squamous	TCGA-BB-7870	1	1	1	1	1
Head and Neck squamous	TCGA-BB-7871	1	1	1	1	1
Head and Neck squamous	TCGA-BB-7872	1	1	1	1	1
Head and Neck squamous	TCGA-BB-8596	1	1	1	1	1
Head and Neck squamous	TCGA-BB-8601	1	1	1	1	1
Head and Neck squamous	TCGA-BB-A5HU	1	1	1	1	1
Head and Neck squamous	TCGA-BB-A5HY	1	1	1	1	1
Head and Neck squamous	TCGA-BB-A5HZ	1	1	1	1	1
Head and Neck squamous	TCGA-BB-A6UM	1	0	0	1	1
Head and Neck squamous	TCGA-BB-A6UO	1	1	1	1	1
Head and Neck squamous	TCGA-C9-A47Z	1	1	1	1	1
Head and Neck squamous	TCGA-C9-A480	1	1	0	1	1
Head and Neck squamous	TCGA-CN-4722	0	0	0	0	0
Head and Neck squamous	TCGA-CN-4723	1	1	1	1	1
Head and Neck squamous	TCGA-CN-4725	1	1	1	1	1
Head and Neck squamous	TCGA-CN-4726	1	1	0	1	1
Head and Neck squamous	TCGA-CN-4727	1	1	1	1	1
Head and Neck squamous	TCGA-CN-4728	1	0	1	1	1
Head and Neck squamous	TCGA-CN-4729	1	1	1	1	1
Head and Neck squamous	TCGA-CN-4730	1	1	1	1	1
Head and Neck squamous	TCGA-CN-4731	1	1	1	1	1
Head and Neck squamous	TCGA-CN-4733	0	0	0	0	1
Head and Neck squamous	TCGA-CN-4734	0	0	0	0	0
Head and Neck squamous	TCGA-CN-4735	1	1	1	1	1

Head and Neck squamous	TCGA-CN-4736	1	1	1	1	1
Head and Neck squamous	TCGA-CN-4737	1	1	1	1	1
Head and Neck squamous	TCGA-CN-4738	1	1	1	1	1
Head and Neck squamous	TCGA-CN-4739	1	1	1	1	1
Head and Neck squamous	TCGA-CN-4740	1	1	1	1	1
Head and Neck squamous	TCGA-CN-4741	1	0	1	0	1
Head and Neck squamous	TCGA-CN-4742	1	1	1	1	1
Head and Neck squamous	TCGA-CN-5355	1	1	1	1	1
Head and Neck squamous	TCGA-CN-5356	1	1	1	1	1
Head and Neck squamous	TCGA-CN-5358	0	1	0	1	1
Head and Neck squamous	TCGA-CN-5359	1	0	1	1	1
Head and Neck squamous	TCGA-CN-5360	1	1	1	1	1
Head and Neck squamous	TCGA-CN-5361	0	0	0	0	0
Head and Neck squamous	TCGA-CN-5363	1	1	1	1	1
Head and Neck squamous	TCGA-CN-5364	1	0	1	1	1
Head and Neck squamous	TCGA-CN-5365	1	1	1	1	1
Head and Neck squamous	TCGA-CN-5366	1	1	1	1	1
Head and Neck squamous	TCGA-CN-5367	1	1	1	1	1
Head and Neck squamous	TCGA-CN-5369	0	0	0	0	1
Head and Neck squamous	TCGA-CN-5370	1	1	1	1	1
Head and Neck squamous	TCGA-CN-5373	1	1	0	1	1
Head and Neck squamous	TCGA-CN-5374	1	1	1	1	1
Head and Neck squamous	TCGA-CN-6010	1	0	1	1	1
Head and Neck squamous	TCGA-CN-6011	1	1	1	1	1
Head and Neck squamous	TCGA-CN-6012	1	1	1	1	1
Head and Neck squamous	TCGA-CN-6013	1	1	1	1	1
Head and Neck squamous	TCGA-CN-6016	1	1	1	1	1
Head and Neck squamous	TCGA-CN-6017	0	0	0	0	1
Head and Neck squamous	TCGA-CN-6018	1	1	1	1	1
Head and Neck squamous	TCGA-CN-6019	1	1	1	1	1
Head and Neck squamous	TCGA-CN-6020	1	1	1	1	1
Head and Neck squamous	TCGA-CN-6021	1	1	0	1	1
Head and Neck squamous	TCGA-CN-6022	1	1	1	1	1
Head and Neck squamous	TCGA-CN-6023	1	0	0	0	1
Head and Neck squamous	TCGA-CN-6024	1	1	1	1	1
Head and Neck squamous	TCGA-CN-6988	1	1	1	1	1
Head and Neck squamous	TCGA-CN-6989	1	1	0	1	1
Head and Neck squamous	TCGA-CN-6992	1	1	1	1	1
Head and Neck squamous	TCGA-CN-6994	1	1	1	1	1
Head and Neck squamous	TCGA-CN-6995	1	1	1	1	1
Head and Neck squamous	TCGA-CN-6996	1	1	1	1	1
Head and Neck squamous	TCGA-CN-6997	1	1	1	1	1
Head and Neck squamous	TCGA-CN-6998	1	0	0	1	1
Head and Neck squamous	TCGA-CN-A497	1	1	0	1	1
Head and Neck squamous	TCGA-CN-A498	1	1	1	1	1
Head and Neck squamous	TCGA-CN-A499	1	1	1	1	1
Head and Neck squamous	TCGA-CN-A49A	1	1	1	1	1
Head and Neck squamous	TCGA-CN-A49B	1	1	1	1	1
Head and Neck squamous	TCGA-CN-A49C	1	0	1	1	1
Head and Neck squamous	TCGA-CN-A63T	1	1	1	1	1
Head and Neck squamous	TCGA-CN-A63U	1	1	1	1	1
Head and Neck squamous	TCGA-CN-A63V	0	0	0	0	0
Head and Neck squamous	TCGA-CN-A63W	1	1	1	1	1
Head and Neck squamous	TCGA-CN-A641	1	1	1	1	1
Head and Neck squamous	TCGA-CN-A642	0	0	1	0	1
Head and Neck squamous	TCGA-CN-A6UY	1	0	1	1	1
Head and Neck squamous	TCGA-CN-A6V1	1	0	0	0	1
Head and Neck squamous	TCGA-CN-A6V3	1	1	1	1	1

Head and Neck squamous	TCGA-CN-A6V6	1	1	0	0	1
Head and Neck squamous	TCGA-CN-A6V7	1	1	1	1	1
Head and Neck squamous	TCGA-CQ-5323	1	0	1	0	1
Head and Neck squamous	TCGA-CQ-5324	0	0	0	0	1
Head and Neck squamous	TCGA-CQ-5325	1	1	1	1	1
Head and Neck squamous	TCGA-CQ-5326	1	1	1	1	1
Head and Neck squamous	TCGA-CQ-5327	1	1	0	1	1
Head and Neck squamous	TCGA-CQ-5329	0	0	1	0	1
Head and Neck squamous	TCGA-CQ-5330	1	1	1	1	1
Head and Neck squamous	TCGA-CQ-5331	1	0	0	1	1
Head and Neck squamous	TCGA-CQ-5332	1	1	1	1	1
Head and Neck squamous	TCGA-CQ-5333	0	0	0	0	1
Head and Neck squamous	TCGA-CQ-5334	0	0	0	1	1
Head and Neck squamous	TCGA-CQ-6218	1	1	1	1	1
Head and Neck squamous	TCGA-CQ-6219	0	0	0	0	0
Head and Neck squamous	TCGA-CQ-6220	1	1	1	1	1
Head and Neck squamous	TCGA-CQ-6221	1	1	1	1	1
Head and Neck squamous	TCGA-CQ-6222	0	0	0	0	0
Head and Neck squamous	TCGA-CQ-6223	1	0	1	1	1
Head and Neck squamous	TCGA-CQ-6224	1	1	1	1	1
Head and Neck squamous	TCGA-CQ-6225	1	1	1	1	1
Head and Neck squamous	TCGA-CQ-6227	1	1	1	1	1
Head and Neck squamous	TCGA-CQ-6228	1	1	1	1	1
Head and Neck squamous	TCGA-CQ-6229	1	1	1	1	1
Head and Neck squamous	TCGA-CQ-7063	0	1	0	0	1
Head and Neck squamous	TCGA-CQ-7065	1	1	1	1	1
Head and Neck squamous	TCGA-CQ-7067	1	1	1	1	1
Head and Neck squamous	TCGA-CQ-7068	0	1	0	0	1
Head and Neck squamous	TCGA-CQ-7069	1	1	1	1	1
Head and Neck squamous	TCGA-CQ-7071	1	0	1	0	1
Head and Neck squamous	TCGA-CQ-7072	1	1	1	1	1
Head and Neck squamous	TCGA-CQ-A4C6	1	1	0	1	1
Head and Neck squamous	TCGA-CQ-A4C7	1	1	1	1	1
Head and Neck squamous	TCGA-CQ-A4C9	1	1	1	1	1
Head and Neck squamous	TCGA-CQ-A4CA	0	1	1	1	1
Head and Neck squamous	TCGA-CQ-A4CB	1	1	0	1	1
Head and Neck squamous	TCGA-CQ-A4CD	1	0	1	1	1
Head and Neck squamous	TCGA-CQ-A4CE	1	1	1	1	1
Head and Neck squamous	TCGA-CQ-A4CG	1	0	0	0	0
Head and Neck squamous	TCGA-CQ-A4CH	0	0	0	0	1
Head and Neck squamous	TCGA-CQ-A4CI	1	1	1	1	1
Head and Neck squamous	TCGA-CR-5243	1	1	1	1	1
Head and Neck squamous	TCGA-CR-5247	1	1	1	1	1
Head and Neck squamous	TCGA-CR-5248	1	1	1	1	1
Head and Neck squamous	TCGA-CR-5249	1	1	0	0	1
Head and Neck squamous	TCGA-CR-5250	1	0	0	1	1
Head and Neck squamous	TCGA-CR-6467	1	0	0	0	1
Head and Neck squamous	TCGA-CR-6470	1	1	0	1	1
Head and Neck squamous	TCGA-CR-6471	1	1	0	0	0
Head and Neck squamous	TCGA-CR-6472	1	1	1	0	1
Head and Neck squamous	TCGA-CR-6473	1	1	1	1	1
Head and Neck squamous	TCGA-CR-6474	1	1	1	1	1
Head and Neck squamous	TCGA-CR-6477	1	1	1	1	1
Head and Neck squamous	TCGA-CR-6478	1	1	1	1	1
Head and Neck squamous	TCGA-CR-6480	1	0	0	0	1
Head and Neck squamous	TCGA-CR-6481	1	1	1	1	1
Head and Neck squamous	TCGA-CR-6482	1	0	0	1	1
Head and Neck squamous	TCGA-CR-6484	1	1	1	1	1

Head and Neck squamous	TCGA-CR-6487	1	0	0	1	1
Head and Neck squamous	TCGA-CR-6488	1	1	1	1	1
Head and Neck squamous	TCGA-CR-6491	1	1	1	1	1
Head and Neck squamous	TCGA-CR-6492	1	0	0	1	1
Head and Neck squamous	TCGA-CR-6493	1	1	1	1	1
Head and Neck squamous	TCGA-CR-7364	1	1	1	1	1
Head and Neck squamous	TCGA-CR-7365	1	1	1	1	1
Head and Neck squamous	TCGA-CR-7367	1	0	1	1	1
Head and Neck squamous	TCGA-CR-7368	1	1	1	1	1
Head and Neck squamous	TCGA-CR-7369	1	1	1	1	1
Head and Neck squamous	TCGA-CR-7370	1	1	1	1	1
Head and Neck squamous	TCGA-CR-7371	1	1	1	1	1
Head and Neck squamous	TCGA-CR-7372	1	1	1	1	1
Head and Neck squamous	TCGA-CR-7373	1	1	1	1	1
Head and Neck squamous	TCGA-CR-7374	1	1	1	1	1
Head and Neck squamous	TCGA-CR-7376	0	1	0	0	1
Head and Neck squamous	TCGA-CR-7377	1	1	1	1	1
Head and Neck squamous	TCGA-CR-7379	1	0	1	1	1
Head and Neck squamous	TCGA-CR-7380	1	1	0	1	1
Head and Neck squamous	TCGA-CR-7382	0	0	0	0	1
Head and Neck squamous	TCGA-CR-7383	1	1	0	1	1
Head and Neck squamous	TCGA-CR-7385	1	0	1	1	1
Head and Neck squamous	TCGA-CR-7386	1	1	1	1	1
Head and Neck squamous	TCGA-CR-7388	1	1	0	1	1
Head and Neck squamous	TCGA-CR-7389	1	0	1	1	1
Head and Neck squamous	TCGA-CR-7390	1	1	1	1	1
Head and Neck squamous	TCGA-CR-7391	0	0	0	0	0
Head and Neck squamous	TCGA-CR-7392	0	0	0	0	1
Head and Neck squamous	TCGA-CR-7393	0	0	0	0	1
Head and Neck squamous	TCGA-CR-7394	1	0	0	0	1
Head and Neck squamous	TCGA-CR-7395	1	0	0	0	1
Head and Neck squamous	TCGA-CR-7397	0	0	0	1	1
Head and Neck squamous	TCGA-CR-7398	1	1	1	1	1
Head and Neck squamous	TCGA-CR-7399	1	1	1	1	1
Head and Neck squamous	TCGA-CR-7401	1	1	0	1	1
Head and Neck squamous	TCGA-CR-7402	1	0	0	1	1
Head and Neck squamous	TCGA-CR-7404	1	1	1	0	1
Head and Neck squamous	TCGA-CV-5430	1	1	1	1	1
Head and Neck squamous	TCGA-CV-5431	1	1	1	1	1
Head and Neck squamous	TCGA-CV-5432	1	1	1	1	1
Head and Neck squamous	TCGA-CV-5434	1	1	1	1	1
Head and Neck squamous	TCGA-CV-5435	1	1	1	1	1
Head and Neck squamous	TCGA-CV-5436	1	1	1	1	1
Head and Neck squamous	TCGA-CV-5439	1	1	1	1	1
Head and Neck squamous	TCGA-CV-5440	1	1	1	1	1
Head and Neck squamous	TCGA-CV-5441	1	1	1	1	1
Head and Neck squamous	TCGA-CV-5442	1	0	0	1	1
Head and Neck squamous	TCGA-CV-5443	1	0	1	0	1
Head and Neck squamous	TCGA-CV-5444	1	1	1	1	1
Head and Neck squamous	TCGA-CV-5966	1	1	1	1	1
Head and Neck squamous	TCGA-CV-5970	1	1	1	1	1
Head and Neck squamous	TCGA-CV-5971	0	0	0	1	0
Head and Neck squamous	TCGA-CV-5973	1	1	1	1	1
Head and Neck squamous	TCGA-CV-5976	1	1	1	1	1
Head and Neck squamous	TCGA-CV-5977	1	1	1	1	1
Head and Neck squamous	TCGA-CV-5978	1	1	1	1	1
Head and Neck squamous	TCGA-CV-5979	1	1	1	1	1
Head and Neck squamous	TCGA-CV-6003	1	1	1	1	1

Head and Neck squamous	TCGA-CV-6433	1	0	0	1	1
Head and Neck squamous	TCGA-CV-6436	1	1	0	0	1
Head and Neck squamous	TCGA-CV-6441	1	1	1	1	1
Head and Neck squamous	TCGA-CV-6933	1	1	1	1	1
Head and Neck squamous	TCGA-CV-6934	1	1	0	1	1
Head and Neck squamous	TCGA-CV-6935	1	1	0	1	1
Head and Neck squamous	TCGA-CV-6936	1	1	1	1	1
Head and Neck squamous	TCGA-CV-6937	1	1	1	1	1
Head and Neck squamous	TCGA-CV-6938	0	0	0	0	1
Head and Neck squamous	TCGA-CV-6939	1	0	0	0	1
Head and Neck squamous	TCGA-CV-6940	1	1	1	1	1
Head and Neck squamous	TCGA-CV-6941	1	1	0	1	1
Head and Neck squamous	TCGA-CV-6942	1	1	0	0	1
Head and Neck squamous	TCGA-CV-6943	0	1	0	0	1
Head and Neck squamous	TCGA-CV-6945	1	1	1	1	1
Head and Neck squamous	TCGA-CV-6948	1	1	1	1	1
Head and Neck squamous	TCGA-CV-6950	1	1	1	1	1
Head and Neck squamous	TCGA-CV-6951	1	0	1	1	1
Head and Neck squamous	TCGA-CV-6952	1	1	1	1	1
Head and Neck squamous	TCGA-CV-6953	1	0	1	0	1
Head and Neck squamous	TCGA-CV-6954	1	1	1	1	1
Head and Neck squamous	TCGA-CV-6955	1	0	0	0	1
Head and Neck squamous	TCGA-CV-6956	1	1	1	1	1
Head and Neck squamous	TCGA-CV-6959	1	1	1	1	1
Head and Neck squamous	TCGA-CV-6960	1	1	1	1	1
Head and Neck squamous	TCGA-CV-6961	1	0	1	1	1
Head and Neck squamous	TCGA-CV-6962	1	1	1	1	1
Head and Neck squamous	TCGA-CV-7089	1	1	0	1	1
Head and Neck squamous	TCGA-CV-7090	1	0	1	1	1
Head and Neck squamous	TCGA-CV-7091	1	1	1	1	1
Head and Neck squamous	TCGA-CV-7095	1	1	1	1	1
Head and Neck squamous	TCGA-CV-7097	1	1	1	1	1
Head and Neck squamous	TCGA-CV-7099	0	0	1	1	1
Head and Neck squamous	TCGA-CV-7100	1	1	1	1	1
Head and Neck squamous	TCGA-CV-7101	1	1	1	1	1
Head and Neck squamous	TCGA-CV-7102	1	1	1	1	1
Head and Neck squamous	TCGA-CV-7103	1	1	1	1	1
Head and Neck squamous	TCGA-CV-7104	1	1	1	1	1
Head and Neck squamous	TCGA-CV-7177	1	1	1	1	1
Head and Neck squamous	TCGA-CV-7178	1	0	1	1	1
Head and Neck squamous	TCGA-CV-7180	1	1	1	1	1
Head and Neck squamous	TCGA-CV-7183	0	0	0	0	0
Head and Neck squamous	TCGA-CV-7235	1	1	1	1	1
Head and Neck squamous	TCGA-CV-7236	1	1	0	1	1
Head and Neck squamous	TCGA-CV-7238	1	1	0	1	1
Head and Neck squamous	TCGA-CV-7242	1	1	1	1	1
Head and Neck squamous	TCGA-CV-7243	1	1	1	1	1
Head and Neck squamous	TCGA-CV-7245	1	1	1	1	1
Head and Neck squamous	TCGA-CV-7247	1	1	1	1	1
Head and Neck squamous	TCGA-CV-7248	1	1	1	1	1
Head and Neck squamous	TCGA-CV-7250	1	0	0	0	0
Head and Neck squamous	TCGA-CV-7252	1	1	1	1	1
Head and Neck squamous	TCGA-CV-7253	1	1	1	1	1
Head and Neck squamous	TCGA-CV-7254	1	1	1	1	1
Head and Neck squamous	TCGA-CV-7255	1	1	1	1	1
Head and Neck squamous	TCGA-CV-7261	1	1	1	1	1
Head and Neck squamous	TCGA-CV-7263	1	1	1	1	1
Head and Neck squamous	TCGA-CV-7406	1	1	1	0	1

Head and Neck squamous	TCGA-CV-7407	1	1	1	1	1
Head and Neck squamous	TCGA-CV-7409	1	1	1	1	1
Head and Neck squamous	TCGA-CV-7410	1	0	0	1	0
Head and Neck squamous	TCGA-CV-7411	1	0	0	0	1
Head and Neck squamous	TCGA-CV-7413	1	1	1	1	1
Head and Neck squamous	TCGA-CV-7414	1	1	1	1	1
Head and Neck squamous	TCGA-CV-7415	1	1	1	1	1
Head and Neck squamous	TCGA-CV-7416	1	1	1	1	1
Head and Neck squamous	TCGA-CV-7418	1	1	1	1	1
Head and Neck squamous	TCGA-CV-7421	1	1	1	1	1
Head and Neck squamous	TCGA-CV-7422	1	1	0	1	1
Head and Neck squamous	TCGA-CV-7423	1	1	1	1	1
Head and Neck squamous	TCGA-CV-7424	1	1	1	1	1
Head and Neck squamous	TCGA-CV-7425	1	1	1	0	1
Head and Neck squamous	TCGA-CV-7427	0	0	0	0	1
Head and Neck squamous	TCGA-CV-7428	0	0	1	1	1
Head and Neck squamous	TCGA-CV-7429	1	1	1	1	1
Head and Neck squamous	TCGA-CV-7430	1	1	1	1	1
Head and Neck squamous	TCGA-CV-7432	1	1	1	1	1
Head and Neck squamous	TCGA-CV-7433	1	1	0	1	1
Head and Neck squamous	TCGA-CV-7434	1	1	0	1	1
Head and Neck squamous	TCGA-CV-7435	1	1	1	1	1
Head and Neck squamous	TCGA-CV-7437	0	0	0	0	1
Head and Neck squamous	TCGA-CV-7438	1	1	1	1	1
Head and Neck squamous	TCGA-CV-7440	1	1	1	1	1
Head and Neck squamous	TCGA-CV-7446	0	0	0	0	0
Head and Neck squamous	TCGA-CV-7568	0	0	0	0	0
Head and Neck squamous	TCGA-CV-A45O	1	0	0	1	1
Head and Neck squamous	TCGA-CV-A45P	0	0	0	0	1
Head and Neck squamous	TCGA-CV-A45Q	0	1	0	0	1
Head and Neck squamous	TCGA-CV-A45R	1	1	1	1	1
Head and Neck squamous	TCGA-CV-A45T	0	0	0	0	0
Head and Neck squamous	TCGA-CV-A45U	1	1	1	1	1
Head and Neck squamous	TCGA-CV-A45V	1	0	0	1	1
Head and Neck squamous	TCGA-CV-A45W	1	1	1	1	1
Head and Neck squamous	TCGA-CV-A45X	1	1	1	1	1
Head and Neck squamous	TCGA-CV-A45Y	1	1	0	1	1
Head and Neck squamous	TCGA-CV-A45Z	1	0	1	1	1
Head and Neck squamous	TCGA-CV-A460	1	1	1	1	1
Head and Neck squamous	TCGA-CV-A461	1	1	1	1	1
Head and Neck squamous	TCGA-CV-A463	0	0	0	0	1
Head and Neck squamous	TCGA-CV-A464	1	1	0	1	1
Head and Neck squamous	TCGA-CV-A465	1	1	1	1	1
Head and Neck squamous	TCGA-CV-A468	0	1	0	0	1
Head and Neck squamous	TCGA-CV-A6JD	0	1	0	0	1
Head and Neck squamous	TCGA-CV-A6JE	1	1	1	1	1
Head and Neck squamous	TCGA-CV-A6JM	1	1	0	1	1
Head and Neck squamous	TCGA-CV-A6JN	1	1	1	1	1
Head and Neck squamous	TCGA-CV-A6JO	1	1	0	0	1
Head and Neck squamous	TCGA-CV-A6JT	1	1	1	1	1
Head and Neck squamous	TCGA-CV-A6JU	1	1	1	1	1
Head and Neck squamous	TCGA-CV-A6JY	1	1	1	1	1
Head and Neck squamous	TCGA-CV-A6JZ	1	1	0	1	1
Head and Neck squamous	TCGA-CV-A6K0	0	0	0	0	0
Head and Neck squamous	TCGA-CV-A6K1	1	1	1	1	1
Head and Neck squamous	TCGA-CV-A6K2	0	0	1	0	1
Head and Neck squamous	TCGA-CX-7082	1	1	1	1	1
Head and Neck squamous	TCGA-CX-7085	0	0	0	0	1

Head and Neck squamous	TCGA-CX-7086	1	1	1	1	1
Head and Neck squamous	TCGA-CX-7219	1	1	1	1	1
Head and Neck squamous	TCGA-CX-A4AQ	1	1	1	1	1
Head and Neck squamous	TCGA-D6-6515	1	0	0	0	1
Head and Neck squamous	TCGA-D6-6516	1	1	1	1	1
Head and Neck squamous	TCGA-D6-6517	1	1	1	1	1
Head and Neck squamous	TCGA-D6-6823	1	1	1	1	1
Head and Neck squamous	TCGA-D6-6824	1	0	0	1	0
Head and Neck squamous	TCGA-D6-6825	1	1	1	1	1
Head and Neck squamous	TCGA-D6-6826	1	1	1	1	1
Head and Neck squamous	TCGA-D6-6827	0	0	1	0	1
Head and Neck squamous	TCGA-D6-8568	1	1	0	1	1
Head and Neck squamous	TCGA-D6-8569	1	1	1	1	1
Head and Neck squamous	TCGA-D6-A4Z9	1	1	0	1	1
Head and Neck squamous	TCGA-D6-A4ZB	1	1	1	1	1
Head and Neck squamous	TCGA-D6-A6EK	0	0	0	0	1
Head and Neck squamous	TCGA-D6-A6EM	1	1	1	1	1
Head and Neck squamous	TCGA-D6-A6EN	0	1	0	1	1
Head and Neck squamous	TCGA-D6-A6EO	1	1	0	1	1
Head and Neck squamous	TCGA-D6-A6EP	1	1	1	1	1
Head and Neck squamous	TCGA-D6-A6EQ	1	1	1	1	1
Head and Neck squamous	TCGA-D6-A6ES	1	1	1	1	1
Head and Neck squamous	TCGA-D6-A74Q	1	1	1	1	1
Head and Neck squamous	TCGA-DQ-5624	1	1	1	1	1
Head and Neck squamous	TCGA-DQ-5625	1	1	0	1	1
Head and Neck squamous	TCGA-DQ-5629	1	1	1	1	1
Head and Neck squamous	TCGA-DQ-5630	1	1	1	1	1
Head and Neck squamous	TCGA-DQ-5631	1	1	1	1	1
Head and Neck squamous	TCGA-DQ-7588	1	1	1	1	1
Head and Neck squamous	TCGA-DQ-7589	1	1	1	1	1
Head and Neck squamous	TCGA-DQ-7590	1	0	1	0	1
Head and Neck squamous	TCGA-DQ-7591	1	1	1	1	1
Head and Neck squamous	TCGA-DQ-7592	1	1	1	1	1
Head and Neck squamous	TCGA-DQ-7593	1	0	1	1	1
Head and Neck squamous	TCGA-DQ-7594	1	1	1	1	1
Head and Neck squamous	TCGA-DQ-7595	1	1	1	1	1
Head and Neck squamous	TCGA-DQ-7596	1	0	0	0	1
Head and Neck squamous	TCGA-F7-7848	1	1	1	1	1
Head and Neck squamous	TCGA-F7-8298	1	1	0	1	1
Head and Neck squamous	TCGA-F7-8489	1	1	0	1	1
Head and Neck squamous	TCGA-F7-A50G	1	1	1	1	1
Head and Neck squamous	TCGA-F7-A50I	1	1	1	1	1
Head and Neck squamous	TCGA-F7-A50J	1	0	1	1	1
Head and Neck squamous	TCGA-F7-A61S	1	1	0	1	1
Head and Neck squamous	TCGA-F7-A61V	1	1	1	1	1
Head and Neck squamous	TCGA-F7-A61W	1	1	1	1	1
Head and Neck squamous	TCGA-F7-A620	1	1	1	1	1
Head and Neck squamous	TCGA-F7-A622	1	1	0	1	1
Head and Neck squamous	TCGA-F7-A623	1	1	1	1	1
Head and Neck squamous	TCGA-F7-A624	1	1	0	1	1
Head and Neck squamous	TCGA-H7-7774	1	0	0	0	1
Head and Neck squamous	TCGA-H7-8501	1	1	0	1	1
Head and Neck squamous	TCGA-H7-8502	1	1	0	1	1
Head and Neck squamous	TCGA-H7-A6C4	1	1	0	1	1
Head and Neck squamous	TCGA-H7-A76A	1	0	0	1	1
Head and Neck squamous	TCGA-HD-7229	1	1	1	1	1
Head and Neck squamous	TCGA-HD-7753	1	1	1	1	1
Head and Neck squamous	TCGA-HD-7754	1	1	0	0	1

Head and Neck squamous	TCGA-HD-7831	1	1	1	1	1
Head and Neck squamous	TCGA-HD-7832	1	1	0	1	1
Head and Neck squamous	TCGA-HD-7917	1	0	1	0	1
Head and Neck squamous	TCGA-HD-8224	1	1	1	1	1
Head and Neck squamous	TCGA-HD-8314	1	1	1	1	1
Head and Neck squamous	TCGA-HD-8634	1	1	0	1	1
Head and Neck squamous	TCGA-HD-8635	1	1	1	1	1
Head and Neck squamous	TCGA-HD-A4C1	1	0	0	0	1
Head and Neck squamous	TCGA-HD-A633	0	1	1	1	1
Head and Neck squamous	TCGA-HD-A634	0	0	0	0	1
Head and Neck squamous	TCGA-HD-A6HZ	1	1	1	1	1
Head and Neck squamous	TCGA-HD-A6I0	0	1	0	1	1
Head and Neck squamous	TCGA-HL-7533	1	0	1	1	1
Head and Neck squamous	TCGA-IQ-7630	1	1	1	1	1
Head and Neck squamous	TCGA-IQ-7631	0	0	0	0	1
Head and Neck squamous	TCGA-IQ-7632	1	0	1	1	1
Head and Neck squamous	TCGA-IQ-A61E	1	1	1	1	1
Head and Neck squamous	TCGA-IQ-A61G	1	1	1	1	1
Head and Neck squamous	TCGA-IQ-A61H	1	1	1	1	1
Head and Neck squamous	TCGA-IQ-A61I	1	1	1	1	1
Head and Neck squamous	TCGA-IQ-A61J	1	1	1	1	1
Head and Neck squamous	TCGA-IQ-A61O	1	1	1	1	1
Head and Neck squamous	TCGA-IQ-A6SG	1	1	1	1	1
Head and Neck squamous	TCGA-IQ-A6SH	1	1	1	1	1
Head and Neck squamous	TCGA-KU-A66S	1	1	0	1	1
Head and Neck squamous	TCGA-KU-A66T	1	1	1	1	1
Head and Neck squamous	TCGA-KU-A6H7	1	1	1	1	1
Head and Neck squamous	TCGA-KU-A6H7	0	0	0	0	0
Head and Neck squamous	TCGA-KU-A6H8	0	0	0	0	1
Head and Neck squamous	TCGA-MT-A51W	1	1	1	1	1
Head and Neck squamous	TCGA-MT-A51X	1	1	1	1	1
Head and Neck squamous	TCGA-MT-A67A	0	1	0	0	1
Head and Neck squamous	TCGA-MT-A67D	1	1	1	1	1
Head and Neck squamous	TCGA-MT-A67F	1	0	0	1	1
Head and Neck squamous	TCGA-MT-A7BN	1	1	1	1	1
Head and Neck squamous	TCGA-MZ-A5BI	1	1	1	1	1
Head and Neck squamous	TCGA-MZ-A6I9	1	1	1	0	1
Head and Neck squamous	TCGA-MZ-A7D7	1	1	1	1	1
Head and Neck squamous	TCGA-P3-A5Q5	1	1	0	0	1
Head and Neck squamous	TCGA-P3-A5Q6	1	1	1	1	1
Head and Neck squamous	TCGA-P3-A5QA	1	1	1	1	1
Head and Neck squamous	TCGA-P3-A5QE	1	1	1	1	1
Head and Neck squamous	TCGA-P3-A5QF	1	1	1	0	1
Head and Neck squamous	TCGA-P3-A6SW	1	1	1	1	1
Head and Neck squamous	TCGA-P3-A6SX	1	1	1	1	1
Head and Neck squamous	TCGA-P3-A6T0	1	0	1	1	1
Head and Neck squamous	TCGA-P3-A6T2	1	0	0	0	1
Head and Neck squamous	TCGA-P3-A6T3	1	1	1	1	1
Head and Neck squamous	TCGA-P3-A6T4	1	1	1	1	1
Head and Neck squamous	TCGA-P3-A6T5	1	1	1	1	1
Head and Neck squamous	TCGA-P3-A6T6	1	0	1	0	1
Head and Neck squamous	TCGA-P3-A6T7	1	1	1	1	1
Head and Neck squamous	TCGA-P3-A6T8	1	1	1	1	1
Head and Neck squamous	TCGA-QK-A64Z	1	1	1	1	1
Head and Neck squamous	TCGA-QK-A652	1	1	1	1	1
Head and Neck squamous	TCGA-QK-A6IF	1	0	1	0	1
Head and Neck squamous	TCGA-QK-A6IG	1	1	1	1	1
Head and Neck squamous	TCGA-QK-A6IH	1	1	1	1	1

Head and Neck squamous	TCGA-QK-A6II	0	0	1	0	1
Head and Neck squamous	TCGA-QK-A6IJ	1	1	1	1	1
Head and Neck squamous	TCGA-QK-A6V9	1	0	0	1	1
Head and Neck squamous	TCGA-QK-A6VB	1	1	1	1	1
Head and Neck squamous	TCGA-QK-A6VC	1	1	1	1	1
Head and Neck squamous	TCGA-QK-A8Z7	1	1	1	1	1
Head and Neck squamous	TCGA-QK-A8Z8	1	1	1	1	1
Head and Neck squamous	TCGA-QK-A8Z9	1	1	1	1	1
Head and Neck squamous	TCGA-QK-A8ZA	1	1	1	1	1
Head and Neck squamous	TCGA-QK-A8ZB	1	1	1	1	1
Head and Neck squamous	TCGA-QK-AA3J	1	1	1	1	1
Head and Neck squamous	TCGA-QK-AA3K	1	1	1	1	1
Head and Neck squamous	TCGA-RS-A6TO	1	0	1	1	1
Head and Neck squamous	TCGA-RS-A6TP	1	1	1	0	1
Head and Neck squamous	TCGA-T2-A6WX	0	0	0	0	1
Head and Neck squamous	TCGA-T2-A6WZ	1	1	1	1	1
Head and Neck squamous	TCGA-T2-A6X0	1	1	1	1	1
Head and Neck squamous	TCGA-T2-A6X2	0	0	0	0	1
Head and Neck squamous	TCGA-T3-A92M	1	1	1	1	1
Head and Neck squamous	TCGA-T3-A92N	1	1	1	1	1
Head and Neck squamous	TCGA-TN-A7HI	1	0	0	0	1
Head and Neck squamous	TCGA-TN-A7HJ	1	1	0	1	1
Head and Neck squamous	TCGA-TN-A7HL	1	1	1	1	1
Head and Neck squamous	TCGA-UF-A718	1	1	1	1	1
Head and Neck squamous	TCGA-UF-A719	1	1	1	1	1
Head and Neck squamous	TCGA-UF-A71A	1	1	1	1	1
Head and Neck squamous	TCGA-UF-A71A	0	0	0	0	0
Head and Neck squamous	TCGA-UF-A71B	1	1	1	1	1
Head and Neck squamous	TCGA-UF-A71D	1	1	1	1	1
Head and Neck squamous	TCGA-UF-A71E	1	1	1	1	1
Head and Neck squamous	TCGA-UF-A7J9	1	1	1	1	1
Head and Neck squamous	TCGA-UF-A7JA	1	1	1	1	1
Head and Neck squamous	TCGA-UF-A7JC	1	1	1	1	1
Head and Neck squamous	TCGA-UF-A7JD	1	1	1	1	1
Head and Neck squamous	TCGA-UF-A7JF	1	1	1	1	1
Head and Neck squamous	TCGA-UF-A7JH	1	1	1	1	1
Head and Neck squamous	TCGA-UF-A7JJ	1	1	0	0	1
Head and Neck squamous	TCGA-UF-A7JK	1	1	1	1	1
Head and Neck squamous	TCGA-UF-A7JO	1	1	1	1	1
Head and Neck squamous	TCGA-UF-A7JS	1	1	0	1	1
Head and Neck squamous	TCGA-UF-A7JT	1	0	0	0	1
Head and Neck squamous	TCGA-UF-A7JV	1	1	0	1	1
Head and Neck squamous	TCGA-UP-A6WW	1	1	1	1	1
Head and Neck squamous	TCGA-WA-A7GZ	1	1	1	1	1
Head and Neck squamous	TCGA-WA-A7H4	1	1	0	1	1
Kindeny Renal clear carcinoma	TCGA-3Z-A93Z	1	0	0	1	0
Kindeny Renal clear carcinoma	TCGA-6D-AA2E	0	0	0	0	0
Kindeny Renal clear carcinoma	TCGA-A3-3306	1	1	1	1	1
Kindeny Renal clear carcinoma	TCGA-A3-3307	0	0	0	1	0
Kindeny Renal clear carcinoma	TCGA-A3-3308	1	1	0	1	1
Kindeny Renal clear carcinoma	TCGA-A3-3311	1	1	0	1	1
Kindeny Renal clear carcinoma	TCGA-A3-3313	1	1	1	1	1
Kindeny Renal clear carcinoma	TCGA-A3-3316	1	1	1	1	1
Kindeny Renal clear carcinoma	TCGA-A3-3317	1	1	1	1	1
Kindeny Renal clear carcinoma	TCGA-A3-3319	1	1	1	1	1
Kindeny Renal clear carcinoma	TCGA-A3-3320	1	0	1	1	1
Kindeny Renal clear carcinoma	TCGA-A3-3322	1	1	0	1	0
Kindeny Renal clear carcinoma	TCGA-A3-3323	1	0	1	1	0

Kindeny Renal clear carcinoma	TCGA-A3-3324	1	0	0	1	0
Kindeny Renal clear carcinoma	TCGA-A3-3325	1	1	1	1	1
Kindeny Renal clear carcinoma	TCGA-A3-3326	0	0	0	1	0
Kindeny Renal clear carcinoma	TCGA-A3-3328	0	0	0	0	0
Kindeny Renal clear carcinoma	TCGA-A3-3329	0	0	0	0	0
Kindeny Renal clear carcinoma	TCGA-A3-3331	1	0	0	1	1
Kindeny Renal clear carcinoma	TCGA-A3-3335	1	0	1	1	0
Kindeny Renal clear carcinoma	TCGA-A3-3343	0	0	0	1	0
Kindeny Renal clear carcinoma	TCGA-A3-3346	1	1	1	1	1
Kindeny Renal clear carcinoma	TCGA-A3-3347	1	1	1	1	1
Kindeny Renal clear carcinoma	TCGA-A3-3349	1	0	0	1	0
Kindeny Renal clear carcinoma	TCGA-A3-3351	1	0	0	1	0
Kindeny Renal clear carcinoma	TCGA-A3-3352	1	1	1	1	1
Kindeny Renal clear carcinoma	TCGA-A3-3357	1	0	0	1	0
Kindeny Renal clear carcinoma	TCGA-A3-3358	0	0	0	1	0
Kindeny Renal clear carcinoma	TCGA-A3-3359	1	0	0	1	0
Kindeny Renal clear carcinoma	TCGA-A3-3362	0	0	0	1	0
Kindeny Renal clear carcinoma	TCGA-A3-3363	1	0	1	1	1
Kindeny Renal clear carcinoma	TCGA-A3-3365	1	1	1	1	1
Kindeny Renal clear carcinoma	TCGA-A3-3367	1	1	0	1	1
Kindeny Renal clear carcinoma	TCGA-A3-3370	1	0	0	1	0
Kindeny Renal clear carcinoma	TCGA-A3-3372	1	1	0	1	1
Kindeny Renal clear carcinoma	TCGA-A3-3373	1	1	0	1	1
Kindeny Renal clear carcinoma	TCGA-A3-3374	1	1	1	1	1
Kindeny Renal clear carcinoma	TCGA-A3-3376	1	0	0	1	0
Kindeny Renal clear carcinoma	TCGA-A3-3378	1	1	0	1	0
Kindeny Renal clear carcinoma	TCGA-A3-3380	1	1	1	1	1
Kindeny Renal clear carcinoma	TCGA-A3-3382	1	1	1	1	1
Kindeny Renal clear carcinoma	TCGA-A3-3383	0	0	0	1	1
Kindeny Renal clear carcinoma	TCGA-A3-3385	0	1	0	0	0
Kindeny Renal clear carcinoma	TCGA-A3-3387	1	0	0	1	0
Kindeny Renal clear carcinoma	TCGA-A3-A6NI	1	0	0	1	0
Kindeny Renal clear carcinoma	TCGA-A3-A6NJ	0	0	0	1	0
Kindeny Renal clear carcinoma	TCGA-A3-A6NL	0	0	0	1	0
Kindeny Renal clear carcinoma	TCGA-A3-A6NN	1	1	0	1	1
Kindeny Renal clear carcinoma	TCGA-A3-A8CQ	0	0	1	1	0
Kindeny Renal clear carcinoma	TCGA-A3-A8OU	1	1	0	1	1
Kindeny Renal clear carcinoma	TCGA-A3-A8OV	1	0	0	1	0
Kindeny Renal clear carcinoma	TCGA-A3-A8OW	0	0	0	1	0
Kindeny Renal clear carcinoma	TCGA-A3-A8OX	0	0	0	0	0
Kindeny Renal clear carcinoma	TCGA-AK-3425	1	1	1	1	1
Kindeny Renal clear carcinoma	TCGA-AK-3426	1	1	1	1	1
Kindeny Renal clear carcinoma	TCGA-AK-3427	1	1	0	1	0
Kindeny Renal clear carcinoma	TCGA-AK-3428	0	0	0	1	0
Kindeny Renal clear carcinoma	TCGA-AK-3429	1	1	0	1	0
Kindeny Renal clear carcinoma	TCGA-AK-3431	0	0	0	1	1
Kindeny Renal clear carcinoma	TCGA-AK-3433	1	1	1	1	1
Kindeny Renal clear carcinoma	TCGA-AK-3434	0	1	1	1	0
Kindeny Renal clear carcinoma	TCGA-AK-3436	1	1	1	1	1
Kindeny Renal clear carcinoma	TCGA-AK-3440	1	1	1	1	1
Kindeny Renal clear carcinoma	TCGA-AK-3443	1	0	1	0	1
Kindeny Renal clear carcinoma	TCGA-AK-3444	0	1	0	1	0
Kindeny Renal clear carcinoma	TCGA-AK-3445	1	1	0	1	1
Kindeny Renal clear carcinoma	TCGA-AK-3447	1	1	1	1	1
Kindeny Renal clear carcinoma	TCGA-AK-3450	1	1	0	1	1
Kindeny Renal clear carcinoma	TCGA-AK-3451	1	0	0	1	0
Kindeny Renal clear carcinoma	TCGA-AK-3453	0	1	0	1	1
Kindeny Renal clear carcinoma	TCGA-AK-3454	1	0	1	1	0

Kindeny Renal clear carcinoma	TCGA-AK-3455	1	0	0	1	0
Kindeny Renal clear carcinoma	TCGA-AK-3456	1	1	1	1	1
Kindeny Renal clear carcinoma	TCGA-AK-3458	1	1	1	1	1
Kindeny Renal clear carcinoma	TCGA-AK-3460	1	0	0	1	0
Kindeny Renal clear carcinoma	TCGA-AK-3461	1	0	1	1	1
Kindeny Renal clear carcinoma	TCGA-AK-3465	1	1	1	1	1
Kindeny Renal clear carcinoma	TCGA-AS-3777	1	1	1	1	1
Kindeny Renal clear carcinoma	TCGA-AS-3778	0	1	0	1	1
Kindeny Renal clear carcinoma	TCGA-B0-4688	1	1	1	1	1
Kindeny Renal clear carcinoma	TCGA-B0-4690	1	1	1	1	1
Kindeny Renal clear carcinoma	TCGA-B0-4691	1	1	0	1	1
Kindeny Renal clear carcinoma	TCGA-B0-4693	1	0	0	1	0
Kindeny Renal clear carcinoma	TCGA-B0-4694	1	1	1	1	1
Kindeny Renal clear carcinoma	TCGA-B0-4696	1	1	1	1	1
Kindeny Renal clear carcinoma	TCGA-B0-4697	0	0	0	1	0
Kindeny Renal clear carcinoma	TCGA-B0-4698	1	1	1	1	1
Kindeny Renal clear carcinoma	TCGA-B0-4699	1	1	1	1	1
Kindeny Renal clear carcinoma	TCGA-B0-4700	1	1	0	0	0
Kindeny Renal clear carcinoma	TCGA-B0-4701	0	0	0	1	0
Kindeny Renal clear carcinoma	TCGA-B0-4703	1	0	0	1	0
Kindeny Renal clear carcinoma	TCGA-B0-4706	0	0	0	1	0
Kindeny Renal clear carcinoma	TCGA-B0-4707	1	1	1	1	1
Kindeny Renal clear carcinoma	TCGA-B0-4710	1	1	0	1	1
Kindeny Renal clear carcinoma	TCGA-B0-4712	1	1	1	1	1
Kindeny Renal clear carcinoma	TCGA-B0-4713	1	1	0	1	0
Kindeny Renal clear carcinoma	TCGA-B0-4714	0	1	0	1	0
Kindeny Renal clear carcinoma	TCGA-B0-4718	1	1	0	1	1
Kindeny Renal clear carcinoma	TCGA-B0-4810	1	1	1	1	0
Kindeny Renal clear carcinoma	TCGA-B0-4811	1	1	0	1	1
Kindeny Renal clear carcinoma	TCGA-B0-4813	1	1	1	1	1
Kindeny Renal clear carcinoma	TCGA-B0-4814	1	0	0	1	1
Kindeny Renal clear carcinoma	TCGA-B0-4815	1	1	0	1	1
Kindeny Renal clear carcinoma	TCGA-B0-4816	0	0	0	1	0
Kindeny Renal clear carcinoma	TCGA-B0-4817	1	1	1	1	1
Kindeny Renal clear carcinoma	TCGA-B0-4818	0	0	0	1	0
Kindeny Renal clear carcinoma	TCGA-B0-4819	1	1	0	1	1
Kindeny Renal clear carcinoma	TCGA-B0-4821	1	1	1	1	1
Kindeny Renal clear carcinoma	TCGA-B0-4822	1	1	1	1	1
Kindeny Renal clear carcinoma	TCGA-B0-4823	1	0	0	1	0
Kindeny Renal clear carcinoma	TCGA-B0-4824	0	0	1	1	0
Kindeny Renal clear carcinoma	TCGA-B0-4827	1	1	0	1	1
Kindeny Renal clear carcinoma	TCGA-B0-4828	1	1	1	1	1
Kindeny Renal clear carcinoma	TCGA-B0-4833	1	0	1	1	1
Kindeny Renal clear carcinoma	TCGA-B0-4834	1	1	0	1	0
Kindeny Renal clear carcinoma	TCGA-B0-4836	1	1	1	1	1
Kindeny Renal clear carcinoma	TCGA-B0-4837	1	0	1	1	1
Kindeny Renal clear carcinoma	TCGA-B0-4838	1	1	0	1	0
Kindeny Renal clear carcinoma	TCGA-B0-4839	1	0	0	0	0
Kindeny Renal clear carcinoma	TCGA-B0-4841	1	0	0	1	0
Kindeny Renal clear carcinoma	TCGA-B0-4842	1	1	0	1	1
Kindeny Renal clear carcinoma	TCGA-B0-4843	1	1	0	1	1
Kindeny Renal clear carcinoma	TCGA-B0-4844	1	1	1	1	1
Kindeny Renal clear carcinoma	TCGA-B0-4845	1	1	0	1	1
Kindeny Renal clear carcinoma	TCGA-B0-4846	0	1	0	1	0
Kindeny Renal clear carcinoma	TCGA-B0-4847	1	0	0	1	0
Kindeny Renal clear carcinoma	TCGA-B0-4848	1	1	1	1	1
Kindeny Renal clear carcinoma	TCGA-B0-4849	0	0	1	1	0
Kindeny Renal clear carcinoma	TCGA-B0-4852	1	1	0	1	1

Kindeny Renal clear carcinoma	TCGA-B0-4945	1	1	0	1	1
Kindeny Renal clear carcinoma	TCGA-B0-5075	1	0	0	1	0
Kindeny Renal clear carcinoma	TCGA-B0-5077	1	0	1	1	1
Kindeny Renal clear carcinoma	TCGA-B0-5080	0	0	0	1	1
Kindeny Renal clear carcinoma	TCGA-B0-5081	1	0	0	1	0
Kindeny Renal clear carcinoma	TCGA-B0-5083	0	0	0	0	0
Kindeny Renal clear carcinoma	TCGA-B0-5084	1	1	1	1	1
Kindeny Renal clear carcinoma	TCGA-B0-5085	0	0	0	1	0
Kindeny Renal clear carcinoma	TCGA-B0-5088	0	0	0	1	1
Kindeny Renal clear carcinoma	TCGA-B0-5092	1	1	0	1	1
Kindeny Renal clear carcinoma	TCGA-B0-5094	1	1	0	1	1
Kindeny Renal clear carcinoma	TCGA-B0-5095	1	1	1	1	1
Kindeny Renal clear carcinoma	TCGA-B0-5096	1	1	0	1	1
Kindeny Renal clear carcinoma	TCGA-B0-5097	1	1	0	1	1
Kindeny Renal clear carcinoma	TCGA-B0-5098	1	1	1	1	1
Kindeny Renal clear carcinoma	TCGA-B0-5099	1	0	0	1	1
Kindeny Renal clear carcinoma	TCGA-B0-5100	1	1	0	0	1
Kindeny Renal clear carcinoma	TCGA-B0-5102	1	1	1	1	0
Kindeny Renal clear carcinoma	TCGA-B0-5104	0	0	0	1	1
Kindeny Renal clear carcinoma	TCGA-B0-5106	1	0	0	0	0
Kindeny Renal clear carcinoma	TCGA-B0-5107	1	1	1	1	1
Kindeny Renal clear carcinoma	TCGA-B0-5108	0	0	1	1	0
Kindeny Renal clear carcinoma	TCGA-B0-5109	1	1	0	0	1
Kindeny Renal clear carcinoma	TCGA-B0-5110	0	0	0	1	0
Kindeny Renal clear carcinoma	TCGA-B0-5113	1	1	0	1	1
Kindeny Renal clear carcinoma	TCGA-B0-5115	1	0	0	1	0
Kindeny Renal clear carcinoma	TCGA-B0-5116	1	1	0	1	1
Kindeny Renal clear carcinoma	TCGA-B0-5117	1	1	1	1	1
Kindeny Renal clear carcinoma	TCGA-B0-5119	0	1	1	1	0
Kindeny Renal clear carcinoma	TCGA-B0-5120	1	0	0	1	1
Kindeny Renal clear carcinoma	TCGA-B0-5121	1	1	0	1	1
Kindeny Renal clear carcinoma	TCGA-B0-5399	1	0	0	1	0
Kindeny Renal clear carcinoma	TCGA-B0-5400	1	0	0	0	0
Kindeny Renal clear carcinoma	TCGA-B0-5402	1	1	1	1	1
Kindeny Renal clear carcinoma	TCGA-B0-5690	0	0	0	1	0
Kindeny Renal clear carcinoma	TCGA-B0-5691	0	1	0	1	1
Kindeny Renal clear carcinoma	TCGA-B0-5692	1	0	0	1	1
Kindeny Renal clear carcinoma	TCGA-B0-5693	0	0	0	1	0
Kindeny Renal clear carcinoma	TCGA-B0-5694	1	1	0	1	1
Kindeny Renal clear carcinoma	TCGA-B0-5695	0	1	0	1	0
Kindeny Renal clear carcinoma	TCGA-B0-5696	1	1	0	1	1
Kindeny Renal clear carcinoma	TCGA-B0-5697	1	1	0	1	0
Kindeny Renal clear carcinoma	TCGA-B0-5698	1	0	0	1	0
Kindeny Renal clear carcinoma	TCGA-B0-5699	0	0	0	1	1
Kindeny Renal clear carcinoma	TCGA-B0-5700	0	0	0	0	0
Kindeny Renal clear carcinoma	TCGA-B0-5701	1	1	0	1	1
Kindeny Renal clear carcinoma	TCGA-B0-5702	0	0	1	1	1
Kindeny Renal clear carcinoma	TCGA-B0-5703	1	1	1	1	1
Kindeny Renal clear carcinoma	TCGA-B0-5705	0	0	1	1	0
Kindeny Renal clear carcinoma	TCGA-B0-5706	1	0	0	1	1
Kindeny Renal clear carcinoma	TCGA-B0-5707	0	0	0	0	0
Kindeny Renal clear carcinoma	TCGA-B0-5709	0	1	0	1	1
Kindeny Renal clear carcinoma	TCGA-B0-5710	1	1	1	1	0
Kindeny Renal clear carcinoma	TCGA-B0-5711	1	0	0	1	0
Kindeny Renal clear carcinoma	TCGA-B0-5712	1	1	0	1	1
Kindeny Renal clear carcinoma	TCGA-B0-5713	1	1	0	1	0
Kindeny Renal clear carcinoma	TCGA-B0-5812	0	0	0	1	0
Kindeny Renal clear carcinoma	TCGA-B2-3923	1	1	1	1	1

(indeny Renal clear carcinoma	TCGA-B2-3924	1	0	0	1	0
(indeny Renal clear carcinoma	TCGA-B2-4098	1	1	0	1	1
(indeny Renal clear carcinoma	TCGA-B2-4099	0	0	0	1	0
(indeny Renal clear carcinoma	TCGA-B2-4101	1	0	0	1	0
(indeny Renal clear carcinoma	TCGA-B2-4102	1	0	0	1	0
(indeny Renal clear carcinoma	TCGA-B2-5633	1	1	0	1	1
(indeny Renal clear carcinoma	TCGA-B2-5635	1	0	0	0	0
(indeny Renal clear carcinoma	TCGA-B2-5636	0	0	0	0	0
(indeny Renal clear carcinoma	TCGA-B2-5639	1	0	0	1	0
(indeny Renal clear carcinoma	TCGA-B2-5641	1	1	1	1	1
(indeny Renal clear carcinoma	TCGA-B2-A4SR	0	1	0	1	0
(indeny Renal clear carcinoma	TCGA-B4-5377	0	1	1	1	0
(indeny Renal clear carcinoma	TCGA-B4-5378	0	0	0	0	0
(indeny Renal clear carcinoma	TCGA-B4-5832	1	1	1	1	1
(indeny Renal clear carcinoma	TCGA-B4-5834	0	0	0	1	0
(indeny Renal clear carcinoma	TCGA-B4-5835	1	1	1	1	1
(indeny Renal clear carcinoma	TCGA-B4-5836	1	1	0	1	0
(indeny Renal clear carcinoma	TCGA-B4-5838	1	1	1	1	1
(indeny Renal clear carcinoma	TCGA-B4-5843	1	0	0	1	0
(indeny Renal clear carcinoma	TCGA-B4-5844	0	1	0	1	0
(indeny Renal clear carcinoma	TCGA-B8-4143	1	1	1	1	1
(indeny Renal clear carcinoma	TCGA-B8-4146	0	0	0	1	1
(indeny Renal clear carcinoma	TCGA-B8-4148	0	1	1	1	0
(indeny Renal clear carcinoma	TCGA-B8-4151	0	1	0	1	0
(indeny Renal clear carcinoma	TCGA-B8-4153	0	0	0	1	0
(indeny Renal clear carcinoma	TCGA-B8-4154	0	0	0	1	0
(indeny Renal clear carcinoma	TCGA-B8-4619	1	1	0	1	0
(indeny Renal clear carcinoma	TCGA-B8-4620	1	1	1	1	1
(indeny Renal clear carcinoma	TCGA-B8-4621	1	0	0	0	0
(indeny Renal clear carcinoma	TCGA-B8-4622	1	1	0	1	1
(indeny Renal clear carcinoma	TCGA-B8-5158	1	1	1	1	1
(indeny Renal clear carcinoma	TCGA-B8-5159	1	0	0	1	0
(indeny Renal clear carcinoma	TCGA-B8-5162	1	1	0	1	1
(indeny Renal clear carcinoma	TCGA-B8-5163	1	1	0	1	0
(indeny Renal clear carcinoma	TCGA-B8-5164	1	1	0	1	1
(indeny Renal clear carcinoma	TCGA-B8-5165	0	0	0	1	0
(indeny Renal clear carcinoma	TCGA-B8-5545	1	1	0	1	1
(indeny Renal clear carcinoma	TCGA-B8-5546	0	0	0	0	0
(indeny Renal clear carcinoma	TCGA-B8-5549	1	1	0	1	1
(indeny Renal clear carcinoma	TCGA-B8-5550	1	1	0	1	1
(indeny Renal clear carcinoma	TCGA-B8-5551	1	1	0	1	1
(indeny Renal clear carcinoma	TCGA-B8-5552	0	0	0	1	0
(indeny Renal clear carcinoma	TCGA-B8-5553	1	0	0	1	0
(indeny Renal clear carcinoma	TCGA-B8-A54D	1	0	0	1	0
(indeny Renal clear carcinoma	TCGA-B8-A54E	0	0	0	1	1
(indeny Renal clear carcinoma	TCGA-B8-A54F	1	0	0	1	1
(indeny Renal clear carcinoma	TCGA-B8-A54G	1	0	1	1	0
(indeny Renal clear carcinoma	TCGA-B8-A54H	0	0	0	1	0
(indeny Renal clear carcinoma	TCGA-B8-A54I	1	1	1	1	0
(indeny Renal clear carcinoma	TCGA-B8-A54J	0	0	0	1	0
(indeny Renal clear carcinoma	TCGA-B8-A54K	0	0	0	1	0
(indeny Renal clear carcinoma	TCGA-B8-A7U6	1	0	0	1	1
(indeny Renal clear carcinoma	TCGA-B8-A8YJ	0	0	0	0	0
(indeny Renal clear carcinoma	TCGA-BP-4158	1	0	0	1	0
(indeny Renal clear carcinoma	TCGA-BP-4159	0	0	0	1	0
(indeny Renal clear carcinoma	TCGA-BP-4160	1	0	0	1	1
(indeny Renal clear carcinoma	TCGA-BP-4161	1	1	0	1	1
(indeny Renal clear carcinoma	TCGA-BP-4162	0	0	0	1	0

Kindeny Renal clear carcinoma	TCGA-BP-4163	1	0	0	1	1
Kindeny Renal clear carcinoma	TCGA-BP-4164	1	1	1	1	1
Kindeny Renal clear carcinoma	TCGA-BP-4165	0	0	0	0	0
Kindeny Renal clear carcinoma	TCGA-BP-4166	1	0	1	1	1
Kindeny Renal clear carcinoma	TCGA-BP-4167	1	1	1	1	1
Kindeny Renal clear carcinoma	TCGA-BP-4169	0	1	0	1	0
Kindeny Renal clear carcinoma	TCGA-BP-4170	0	0	0	1	0
Kindeny Renal clear carcinoma	TCGA-BP-4173	1	0	0	0	0
Kindeny Renal clear carcinoma	TCGA-BP-4174	1	1	0	1	0
Kindeny Renal clear carcinoma	TCGA-BP-4176	1	1	1	1	1
Kindeny Renal clear carcinoma	TCGA-BP-4177	0	0	0	0	0
Kindeny Renal clear carcinoma	TCGA-BP-4325	0	0	1	1	0
Kindeny Renal clear carcinoma	TCGA-BP-4326	1	1	0	1	0
Kindeny Renal clear carcinoma	TCGA-BP-4327	0	1	1	1	0
Kindeny Renal clear carcinoma	TCGA-BP-4329	1	0	0	1	1
Kindeny Renal clear carcinoma	TCGA-BP-4330	0	0	0	1	0
Kindeny Renal clear carcinoma	TCGA-BP-4331	1	0	1	1	0
Kindeny Renal clear carcinoma	TCGA-BP-4332	0	0	0	1	0
Kindeny Renal clear carcinoma	TCGA-BP-4334	1	1	1	1	1
Kindeny Renal clear carcinoma	TCGA-BP-4335	0	0	0	1	0
Kindeny Renal clear carcinoma	TCGA-BP-4337	0	0	0	1	0
Kindeny Renal clear carcinoma	TCGA-BP-4338	1	1	0	1	0
Kindeny Renal clear carcinoma	TCGA-BP-4340	1	1	0	1	1
Kindeny Renal clear carcinoma	TCGA-BP-4341	0	1	0	1	0
Kindeny Renal clear carcinoma	TCGA-BP-4342	1	1	1	1	1
Kindeny Renal clear carcinoma	TCGA-BP-4343	1	1	0	1	1
Kindeny Renal clear carcinoma	TCGA-BP-4344	1	0	0	1	0
Kindeny Renal clear carcinoma	TCGA-BP-4345	0	0	0	0	0
Kindeny Renal clear carcinoma	TCGA-BP-4346	1	0	0	1	0
Kindeny Renal clear carcinoma	TCGA-BP-4347	1	1	0	1	1
Kindeny Renal clear carcinoma	TCGA-BP-4349	0	0	0	1	1
Kindeny Renal clear carcinoma	TCGA-BP-4351	1	1	1	1	1
Kindeny Renal clear carcinoma	TCGA-BP-4352	1	1	1	1	1
Kindeny Renal clear carcinoma	TCGA-BP-4353	1	1	1	1	1
Kindeny Renal clear carcinoma	TCGA-BP-4354	1	1	0	1	0
Kindeny Renal clear carcinoma	TCGA-BP-4355	0	0	0	1	0
Kindeny Renal clear carcinoma	TCGA-BP-4756	1	0	0	0	0
Kindeny Renal clear carcinoma	TCGA-BP-4758	1	0	0	1	0
Kindeny Renal clear carcinoma	TCGA-BP-4759	0	0	0	1	0
Kindeny Renal clear carcinoma	TCGA-BP-4760	0	0	0	0	0
Kindeny Renal clear carcinoma	TCGA-BP-4761	1	1	1	1	1
Kindeny Renal clear carcinoma	TCGA-BP-4762	1	0	1	1	1
Kindeny Renal clear carcinoma	TCGA-BP-4763	1	1	0	1	1
Kindeny Renal clear carcinoma	TCGA-BP-4765	0	0	0	1	0
Kindeny Renal clear carcinoma	TCGA-BP-4766	1	1	0	1	1
Kindeny Renal clear carcinoma	TCGA-BP-4768	1	1	1	1	1
Kindeny Renal clear carcinoma	TCGA-BP-4769	0	0	0	0	0
Kindeny Renal clear carcinoma	TCGA-BP-4770	1	1	1	1	1
Kindeny Renal clear carcinoma	TCGA-BP-4771	1	1	0	1	1
Kindeny Renal clear carcinoma	TCGA-BP-4774	1	0	0	1	0
Kindeny Renal clear carcinoma	TCGA-BP-4775	1	1	1	1	1
Kindeny Renal clear carcinoma	TCGA-BP-4776	0	0	0	1	0
Kindeny Renal clear carcinoma	TCGA-BP-4777	1	0	1	1	0
Kindeny Renal clear carcinoma	TCGA-BP-4781	1	1	1	1	1
Kindeny Renal clear carcinoma	TCGA-BP-4782	1	1	0	1	1
Kindeny Renal clear carcinoma	TCGA-BP-4784	0	0	0	0	0
Kindeny Renal clear carcinoma	TCGA-BP-4787	1	1	1	1	1
Kindeny Renal clear carcinoma	TCGA-BP-4789	0	0	0	1	0

(indeny Renal clear carcinoma	TCGA-BP-4790	0	0	0	1	0
(indeny Renal clear carcinoma	TCGA-BP-4795	0	1	0	0	0
(indeny Renal clear carcinoma	TCGA-BP-4797	1	1	1	1	1
(indeny Renal clear carcinoma	TCGA-BP-4798	1	1	1	1	1
(indeny Renal clear carcinoma	TCGA-BP-4799	1	1	1	1	1
(indeny Renal clear carcinoma	TCGA-BP-4801	1	1	0	1	0
(indeny Renal clear carcinoma	TCGA-BP-4803	1	1	0	1	1
(indeny Renal clear carcinoma	TCGA-BP-4804	0	0	0	0	0
(indeny Renal clear carcinoma	TCGA-BP-4807	0	0	0	1	0
(indeny Renal clear carcinoma	TCGA-BP-4959	1	1	1	1	0
(indeny Renal clear carcinoma	TCGA-BP-4960	1	1	1	1	1
(indeny Renal clear carcinoma	TCGA-BP-4961	1	0	0	1	0
(indeny Renal clear carcinoma	TCGA-BP-4962	1	1	1	1	1
(indeny Renal clear carcinoma	TCGA-BP-4963	1	1	1	1	1
(indeny Renal clear carcinoma	TCGA-BP-4964	1	1	0	1	1
(indeny Renal clear carcinoma	TCGA-BP-4965	1	1	0	1	0
(indeny Renal clear carcinoma	TCGA-BP-4967	1	1	0	1	1
(indeny Renal clear carcinoma	TCGA-BP-4968	1	1	1	1	1
(indeny Renal clear carcinoma	TCGA-BP-4969	0	0	0	1	0
(indeny Renal clear carcinoma	TCGA-BP-4970	1	1	0	1	0
(indeny Renal clear carcinoma	TCGA-BP-4971	1	0	0	1	0
(indeny Renal clear carcinoma	TCGA-BP-4972	0	0	0	1	0
(indeny Renal clear carcinoma	TCGA-BP-4973	1	1	0	1	1
(indeny Renal clear carcinoma	TCGA-BP-4974	1	0	1	1	0
(indeny Renal clear carcinoma	TCGA-BP-4975	1	0	1	1	1
(indeny Renal clear carcinoma	TCGA-BP-4976	0	0	0	0	0
(indeny Renal clear carcinoma	TCGA-BP-4977	1	0	0	1	0
(indeny Renal clear carcinoma	TCGA-BP-4981	1	0	0	1	1
(indeny Renal clear carcinoma	TCGA-BP-4982	0	0	0	1	0
(indeny Renal clear carcinoma	TCGA-BP-4983	1	1	1	1	1
(indeny Renal clear carcinoma	TCGA-BP-4985	1	1	1	1	1
(indeny Renal clear carcinoma	TCGA-BP-4986	1	1	1	1	1
(indeny Renal clear carcinoma	TCGA-BP-4987	0	0	1	1	0
(indeny Renal clear carcinoma	TCGA-BP-4988	0	0	0	0	0
(indeny Renal clear carcinoma	TCGA-BP-4989	1	1	0	1	0
(indeny Renal clear carcinoma	TCGA-BP-4991	1	0	0	1	0
(indeny Renal clear carcinoma	TCGA-BP-4992	1	0	1	1	1
(indeny Renal clear carcinoma	TCGA-BP-4993	1	0	0	1	0
(indeny Renal clear carcinoma	TCGA-BP-4994	1	1	1	1	1
(indeny Renal clear carcinoma	TCGA-BP-4995	1	1	0	1	1
(indeny Renal clear carcinoma	TCGA-BP-4998	1	1	0	1	0
(indeny Renal clear carcinoma	TCGA-BP-4999	1	0	0	1	0
(indeny Renal clear carcinoma	TCGA-BP-5000	1	1	1	1	1
(indeny Renal clear carcinoma	TCGA-BP-5001	0	1	0	1	0
(indeny Renal clear carcinoma	TCGA-BP-5004	1	1	0	1	1
(indeny Renal clear carcinoma	TCGA-BP-5006	0	0	0	1	0
(indeny Renal clear carcinoma	TCGA-BP-5007	0	1	0	1	0
(indeny Renal clear carcinoma	TCGA-BP-5008	1	0	0	1	0
(indeny Renal clear carcinoma	TCGA-BP-5009	1	1	0	1	1
(indeny Renal clear carcinoma	TCGA-BP-5010	1	1	0	1	1
(indeny Renal clear carcinoma	TCGA-BP-5168	0	0	0	1	0
(indeny Renal clear carcinoma	TCGA-BP-5169	1	1	1	1	1
(indeny Renal clear carcinoma	TCGA-BP-5170	1	0	0	1	0
(indeny Renal clear carcinoma	TCGA-BP-5173	1	1	0	1	1
(indeny Renal clear carcinoma	TCGA-BP-5174	1	0	0	1	0
(indeny Renal clear carcinoma	TCGA-BP-5175	1	1	1	1	1
(indeny Renal clear carcinoma	TCGA-BP-5176	0	1	0	1	0
(indeny Renal clear carcinoma	TCGA-BP-5177	1	1	0	1	1

Kindeny Renal clear carcinoma	TCGA-BP-5178	1	1	1	1	1
Kindeny Renal clear carcinoma	TCGA-BP-5180	1	1	1	1	1
Kindeny Renal clear carcinoma	TCGA-BP-5181	1	1	0	1	0
Kindeny Renal clear carcinoma	TCGA-BP-5182	1	1	0	1	0
Kindeny Renal clear carcinoma	TCGA-BP-5183	1	1	0	1	1
Kindeny Renal clear carcinoma	TCGA-BP-5184	0	0	0	1	0
Kindeny Renal clear carcinoma	TCGA-BP-5185	1	1	1	1	1
Kindeny Renal clear carcinoma	TCGA-BP-5186	0	0	0	1	0
Kindeny Renal clear carcinoma	TCGA-BP-5187	1	0	0	1	0
Kindeny Renal clear carcinoma	TCGA-BP-5189	1	1	0	1	1
Kindeny Renal clear carcinoma	TCGA-BP-5190	1	0	0	1	0
Kindeny Renal clear carcinoma	TCGA-BP-5191	1	1	1	0	1
Kindeny Renal clear carcinoma	TCGA-BP-5192	1	0	0	1	0
Kindeny Renal clear carcinoma	TCGA-BP-5194	0	0	0	1	0
Kindeny Renal clear carcinoma	TCGA-BP-5195	1	0	0	1	0
Kindeny Renal clear carcinoma	TCGA-BP-5196	1	1	1	1	1
Kindeny Renal clear carcinoma	TCGA-BP-5198	1	1	1	1	1
Kindeny Renal clear carcinoma	TCGA-BP-5199	1	0	0	1	0
Kindeny Renal clear carcinoma	TCGA-BP-5200	1	1	0	1	1
Kindeny Renal clear carcinoma	TCGA-BP-5201	1	1	1	1	1
Kindeny Renal clear carcinoma	TCGA-BP-5202	0	0	0	1	0
Kindeny Renal clear carcinoma	TCGA-CJ-4634	0	0	1	1	0
Kindeny Renal clear carcinoma	TCGA-CJ-4635	1	1	1	1	1
Kindeny Renal clear carcinoma	TCGA-CJ-4636	1	1	0	1	1
Kindeny Renal clear carcinoma	TCGA-CJ-4637	1	1	0	1	1
Kindeny Renal clear carcinoma	TCGA-CJ-4638	1	1	0	1	1
Kindeny Renal clear carcinoma	TCGA-CJ-4639	0	0	0	1	1
Kindeny Renal clear carcinoma	TCGA-CJ-4640	1	1	1	1	1
Kindeny Renal clear carcinoma	TCGA-CJ-4641	1	1	0	1	0
Kindeny Renal clear carcinoma	TCGA-CJ-4642	1	1	0	1	1
Kindeny Renal clear carcinoma	TCGA-CJ-4643	0	1	1	1	1
Kindeny Renal clear carcinoma	TCGA-CJ-4644	1	1	0	1	1
Kindeny Renal clear carcinoma	TCGA-CJ-4868	1	1	0	1	1
Kindeny Renal clear carcinoma	TCGA-CJ-4869	1	1	0	1	0
Kindeny Renal clear carcinoma	TCGA-CJ-4870	0	1	0	1	1
Kindeny Renal clear carcinoma	TCGA-CJ-4871	1	1	1	1	1
Kindeny Renal clear carcinoma	TCGA-CJ-4872	1	0	1	1	1
Kindeny Renal clear carcinoma	TCGA-CJ-4873	1	1	0	1	0
Kindeny Renal clear carcinoma	TCGA-CJ-4874	1	0	0	1	1
Kindeny Renal clear carcinoma	TCGA-CJ-4875	1	0	0	0	0
Kindeny Renal clear carcinoma	TCGA-CJ-4876	1	1	0	1	1
Kindeny Renal clear carcinoma	TCGA-CJ-4878	1	1	1	1	0
Kindeny Renal clear carcinoma	TCGA-CJ-4881	1	1	1	1	1
Kindeny Renal clear carcinoma	TCGA-CJ-4882	1	0	0	1	1
Kindeny Renal clear carcinoma	TCGA-CJ-4884	1	1	0	1	1
Kindeny Renal clear carcinoma	TCGA-CJ-4885	1	1	0	1	1
Kindeny Renal clear carcinoma	TCGA-CJ-4886	1	0	0	1	0
Kindeny Renal clear carcinoma	TCGA-CJ-4887	0	1	1	1	0
Kindeny Renal clear carcinoma	TCGA-CJ-4888	1	1	0	1	1
Kindeny Renal clear carcinoma	TCGA-CJ-4889	1	1	0	1	1
Kindeny Renal clear carcinoma	TCGA-CJ-4890	1	1	0	0	1
Kindeny Renal clear carcinoma	TCGA-CJ-4891	1	1	0	1	1
Kindeny Renal clear carcinoma	TCGA-CJ-4892	0	0	0	1	0
Kindeny Renal clear carcinoma	TCGA-CJ-4893	1	0	1	1	1
Kindeny Renal clear carcinoma	TCGA-CJ-4894	1	1	0	1	1
Kindeny Renal clear carcinoma	TCGA-CJ-4895	1	1	1	1	1
Kindeny Renal clear carcinoma	TCGA-CJ-4897	1	1	1	1	1
Kindeny Renal clear carcinoma	TCGA-CJ-4899	0	0	0	1	0

<indeny Renal clear carcinoma	TCGA-CJ-4900	1	1	0	1	1
<indeny Renal clear carcinoma	TCGA-CJ-4901	1	1	1	1	1
<indeny Renal clear carcinoma	TCGA-CJ-4902	0	1	0	1	1
<indeny Renal clear carcinoma	TCGA-CJ-4903	1	1	0	1	1
<indeny Renal clear carcinoma	TCGA-CJ-4904	1	1	1	1	1
<indeny Renal clear carcinoma	TCGA-CJ-4905	0	0	0	1	0
<indeny Renal clear carcinoma	TCGA-CJ-4907	1	1	1	1	1
<indeny Renal clear carcinoma	TCGA-CJ-4908	1	1	0	1	1
<indeny Renal clear carcinoma	TCGA-CJ-4912	1	1	0	1	1
<indeny Renal clear carcinoma	TCGA-CJ-4916	1	0	0	1	0
<indeny Renal clear carcinoma	TCGA-CJ-4918	0	0	0	1	0
<indeny Renal clear carcinoma	TCGA-CJ-4920	1	1	0	1	1
<indeny Renal clear carcinoma	TCGA-CJ-4923	1	0	0	1	1
<indeny Renal clear carcinoma	TCGA-CJ-5671	1	1	0	1	1
<indeny Renal clear carcinoma	TCGA-CJ-5672	1	1	0	1	0
<indeny Renal clear carcinoma	TCGA-CJ-5675	1	0	1	1	0
<indeny Renal clear carcinoma	TCGA-CJ-5676	1	1	1	1	1
<indeny Renal clear carcinoma	TCGA-CJ-5677	1	1	1	1	1
<indeny Renal clear carcinoma	TCGA-CJ-5678	1	1	0	1	0
<indeny Renal clear carcinoma	TCGA-CJ-5679	1	1	1	1	1
<indeny Renal clear carcinoma	TCGA-CJ-5680	1	1	1	1	1
<indeny Renal clear carcinoma	TCGA-CJ-5681	0	0	1	0	0
<indeny Renal clear carcinoma	TCGA-CJ-5682	1	1	0	1	1
<indeny Renal clear carcinoma	TCGA-CJ-5683	1	0	0	1	0
<indeny Renal clear carcinoma	TCGA-CJ-5684	0	0	0	1	0
<indeny Renal clear carcinoma	TCGA-CJ-5686	1	0	0	1	1
<indeny Renal clear carcinoma	TCGA-CJ-5689	1	1	0	1	1
<indeny Renal clear carcinoma	TCGA-CJ-6027	1	1	1	1	1
<indeny Renal clear carcinoma	TCGA-CJ-6028	1	1	1	1	1
<indeny Renal clear carcinoma	TCGA-CJ-6030	1	1	0	1	1
<indeny Renal clear carcinoma	TCGA-CJ-6031	1	1	0	1	1
<indeny Renal clear carcinoma	TCGA-CJ-6032	1	0	1	1	1
<indeny Renal clear carcinoma	TCGA-CJ-6033	1	1	0	1	1
<indeny Renal clear carcinoma	TCGA-CW-5580	1	1	0	1	1
<indeny Renal clear carcinoma	TCGA-CW-5581	0	1	0	1	0
<indeny Renal clear carcinoma	TCGA-CW-5583	0	0	0	1	0
<indeny Renal clear carcinoma	TCGA-CW-5584	1	1	1	1	1
<indeny Renal clear carcinoma	TCGA-CW-5585	1	0	1	1	0
<indeny Renal clear carcinoma	TCGA-CW-5587	1	0	0	1	0
<indeny Renal clear carcinoma	TCGA-CW-5588	1	1	1	1	1
<indeny Renal clear carcinoma	TCGA-CW-5589	0	0	0	1	0
<indeny Renal clear carcinoma	TCGA-CW-5590	0	0	0	0	0
<indeny Renal clear carcinoma	TCGA-CW-5591	0	0	0	1	0
<indeny Renal clear carcinoma	TCGA-CW-6087	1	0	0	0	1
<indeny Renal clear carcinoma	TCGA-CW-6088	1	1	0	1	1
<indeny Renal clear carcinoma	TCGA-CW-6090	1	1	0	1	1
<indeny Renal clear carcinoma	TCGA-CW-6093	0	1	0	1	0
<indeny Renal clear carcinoma	TCGA-CW-6097	1	1	0	1	1
<indeny Renal clear carcinoma	TCGA-CZ-4853	0	0	1	1	0
<indeny Renal clear carcinoma	TCGA-CZ-4854	1	1	0	0	1
<indeny Renal clear carcinoma	TCGA-CZ-4856	0	0	0	1	0
<indeny Renal clear carcinoma	TCGA-CZ-4857	1	1	0	1	1
<indeny Renal clear carcinoma	TCGA-CZ-4858	1	1	1	1	1
<indeny Renal clear carcinoma	TCGA-CZ-4859	0	0	1	1	0
<indeny Renal clear carcinoma	TCGA-CZ-4860	1	1	1	1	1
<indeny Renal clear carcinoma	TCGA-CZ-4861	1	0	0	1	1
<indeny Renal clear carcinoma	TCGA-CZ-4862	1	1	0	1	1
<indeny Renal clear carcinoma	TCGA-CZ-4863	1	0	0	1	0

Kindeny Renal clear carcinoma	TCGA-CZ-4864	1	1	0	1	0
Kindeny Renal clear carcinoma	TCGA-CZ-4865	0	1	0	1	1
Kindeny Renal clear carcinoma	TCGA-CZ-4866	1	1	1	1	1
Kindeny Renal clear carcinoma	TCGA-CZ-5451	1	0	0	1	1
Kindeny Renal clear carcinoma	TCGA-CZ-5452	1	1	1	1	0
Kindeny Renal clear carcinoma	TCGA-CZ-5453	1	1	0	1	1
Kindeny Renal clear carcinoma	TCGA-CZ-5454	0	0	0	1	0
Kindeny Renal clear carcinoma	TCGA-CZ-5455	1	0	0	1	1
Kindeny Renal clear carcinoma	TCGA-CZ-5456	1	1	1	1	1
Kindeny Renal clear carcinoma	TCGA-CZ-5457	1	1	1	1	1
Kindeny Renal clear carcinoma	TCGA-CZ-5458	0	0	0	1	1
Kindeny Renal clear carcinoma	TCGA-CZ-5459	1	0	0	1	1
Kindeny Renal clear carcinoma	TCGA-CZ-5460	1	1	1	1	1
Kindeny Renal clear carcinoma	TCGA-CZ-5461	1	1	0	1	1
Kindeny Renal clear carcinoma	TCGA-CZ-5462	1	1	0	1	1
Kindeny Renal clear carcinoma	TCGA-CZ-5463	1	1	0	1	1
Kindeny Renal clear carcinoma	TCGA-CZ-5464	0	0	0	1	0
Kindeny Renal clear carcinoma	TCGA-CZ-5465	1	1	1	1	1
Kindeny Renal clear carcinoma	TCGA-CZ-5466	1	1	0	1	1
Kindeny Renal clear carcinoma	TCGA-CZ-5467	1	1	0	1	0
Kindeny Renal clear carcinoma	TCGA-CZ-5468	1	1	1	1	1
Kindeny Renal clear carcinoma	TCGA-CZ-5469	1	1	1	1	1
Kindeny Renal clear carcinoma	TCGA-CZ-5470	1	1	0	1	1
Kindeny Renal clear carcinoma	TCGA-CZ-5982	1	1	1	1	1
Kindeny Renal clear carcinoma	TCGA-CZ-5984	1	1	1	1	1
Kindeny Renal clear carcinoma	TCGA-CZ-5985	1	1	1	1	1
Kindeny Renal clear carcinoma	TCGA-CZ-5986	1	1	0	1	1
Kindeny Renal clear carcinoma	TCGA-CZ-5987	1	1	1	1	1
Kindeny Renal clear carcinoma	TCGA-CZ-5988	1	1	0	1	1
Kindeny Renal clear carcinoma	TCGA-CZ-5989	1	1	0	1	0
Kindeny Renal clear carcinoma	TCGA-DV-5565	1	1	1	1	1
Kindeny Renal clear carcinoma	TCGA-DV-5566	1	0	0	1	1
Kindeny Renal clear carcinoma	TCGA-DV-5567	0	0	0	0	0
Kindeny Renal clear carcinoma	TCGA-DV-5568	1	0	1	1	0
Kindeny Renal clear carcinoma	TCGA-DV-5569	0	0	0	1	0
Kindeny Renal clear carcinoma	TCGA-DV-5573	1	0	1	1	0
Kindeny Renal clear carcinoma	TCGA-DV-5574	1	1	0	1	0
Kindeny Renal clear carcinoma	TCGA-DV-5575	1	1	1	1	1
Kindeny Renal clear carcinoma	TCGA-DV-5576	1	1	0	0	1
Kindeny Renal clear carcinoma	TCGA-DV-A4VX	1	0	0	1	1
Kindeny Renal clear carcinoma	TCGA-DV-A4VZ	0	0	0	0	0
Kindeny Renal clear carcinoma	TCGA-DV-A4W0	1	1	0	1	1
Kindeny Renal clear carcinoma	TCGA-DV-A4W0	0	0	0	0	0
Kindeny Renal clear carcinoma	TCGA-EU-5904	0	0	0	1	0
Kindeny Renal clear carcinoma	TCGA-EU-5905	0	0	0	0	0
Kindeny Renal clear carcinoma	TCGA-EU-5906	0	0	0	1	0
Kindeny Renal clear carcinoma	TCGA-EU-5907	1	1	1	1	1
Kindeny Renal clear carcinoma	TCGA-G6-A5PC	1	1	0	1	1
Kindeny Renal clear carcinoma	TCGA-G6-A8L6	0	0	0	1	0
Kindeny Renal clear carcinoma	TCGA-G6-A8L7	1	1	1	1	1
Kindeny Renal clear carcinoma	TCGA-G6-A8L8	1	1	1	1	1
Kindeny Renal clear carcinoma	TCGA-GK-A6C7	1	1	0	1	0
Kindeny Renal clear carcinoma	TCGA-MM-A563	1	1	1	1	1
Kindeny Renal clear carcinoma	TCGA-MM-A564	1	0	0	1	0
Kindeny Renal clear carcinoma	TCGA-MM-A84U	1	1	0	1	1
Kindeny Renal clear carcinoma	TCGA-MW-A4EC	1	0	1	1	0
Kindeny Renal clear carcinoma	TCGA-T7-A92I	1	1	1	1	0
leny Renal Papillary cell carcin	TCGA-2K-A9WE	0	0	0	0	0

leny Renal Papillary cell carcin	TCGA-2Z-A9J1	0	0	0	0	0
leny Renal Papillary cell carcin	TCGA-2Z-A9J2	0	0	0	0	0
leny Renal Papillary cell carcin	TCGA-2Z-A9J3	0	1	0	0	0
leny Renal Papillary cell carcin	TCGA-2Z-A9J5	0	0	0	0	0
leny Renal Papillary cell carcin	TCGA-2Z-A9J6	0	0	0	0	0
leny Renal Papillary cell carcin	TCGA-2Z-A9J7	0	0	0	0	1
leny Renal Papillary cell carcin	TCGA-2Z-A9J8	1	1	1	1	1
leny Renal Papillary cell carcin	TCGA-2Z-A9J9	0	0	0	0	0
leny Renal Papillary cell carcin	TCGA-2Z-A9JD	0	0	0	0	0
leny Renal Papillary cell carcin	TCGA-2Z-A9JE	0	0	0	0	1
leny Renal Papillary cell carcin	TCGA-2Z-A9JG	1	1	1	1	1
leny Renal Papillary cell carcin	TCGA-2Z-A9JI	1	1	1	1	1
leny Renal Papillary cell carcin	TCGA-2Z-A9JJ	1	1	1	0	1
leny Renal Papillary cell carcin	TCGA-2Z-A9JK	1	0	0	1	1
leny Renal Papillary cell carcin	TCGA-2Z-A9JL	0	0	0	0	0
leny Renal Papillary cell carcin	TCGA-2Z-A9JM	0	0	0	0	0
leny Renal Papillary cell carcin	TCGA-2Z-A9JN	0	0	0	0	0
leny Renal Papillary cell carcin	TCGA-2Z-A9JO	0	0	0	0	0
leny Renal Papillary cell carcin	TCGA-2Z-A9JP	1	0	0	0	0
leny Renal Papillary cell carcin	TCGA-2Z-A9JQ	0	0	0	0	0
leny Renal Papillary cell carcin	TCGA-2Z-A9JR	0	0	0	0	0
leny Renal Papillary cell carcin	TCGA-2Z-A9JS	0	1	0	0	0
leny Renal Papillary cell carcin	TCGA-2Z-A9JT	0	0	0	0	0
leny Renal Papillary cell carcin	TCGA-4A-A93W	0	0	0	0	0
leny Renal Papillary cell carcin	TCGA-4A-A93X	1	1	1	1	1
leny Renal Papillary cell carcin	TCGA-4A-A93Y	0	0	0	0	0
leny Renal Papillary cell carcin	TCGA-5P-A9JU	1	0	1	1	1
leny Renal Papillary cell carcin	TCGA-5P-A9JV	1	1	1	1	1
leny Renal Papillary cell carcin	TCGA-5P-A9JW	1	0	0	0	0
leny Renal Papillary cell carcin	TCGA-5P-A9JY	1	1	0	1	1
leny Renal Papillary cell carcin	TCGA-5P-A9JZ	0	0	0	1	0
leny Renal Papillary cell carcin	TCGA-5P-A9K0	0	0	0	0	0
leny Renal Papillary cell carcin	TCGA-5P-A9K2	0	0	0	0	0
leny Renal Papillary cell carcin	TCGA-5P-A9K3	1	1	1	1	1
leny Renal Papillary cell carcin	TCGA-5P-A9K4	0	0	0	0	0
leny Renal Papillary cell carcin	TCGA-5P-A9K6	0	0	0	1	0
leny Renal Papillary cell carcin	TCGA-5P-A9K8	1	1	0	1	1
leny Renal Papillary cell carcin	TCGA-5P-A9K9	1	1	1	1	1
leny Renal Papillary cell carcin	TCGA-5P-A9KA	0	0	0	0	0
leny Renal Papillary cell carcin	TCGA-5P-A9KC	1	1	1	1	1
leny Renal Papillary cell carcin	TCGA-5P-A9KE	0	0	1	0	0
leny Renal Papillary cell carcin	TCGA-5P-A9KF	0	0	0	0	0
leny Renal Papillary cell carcin	TCGA-5P-A9KH	1	1	1	1	1
leny Renal Papillary cell carcin	TCGA-A4-7286	1	1	1	1	1
leny Renal Papillary cell carcin	TCGA-A4-7287	1	1	1	1	1
leny Renal Papillary cell carcin	TCGA-A4-7288	0	0	0	1	1
leny Renal Papillary cell carcin	TCGA-A4-7583	1	1	0	1	1
leny Renal Papillary cell carcin	TCGA-A4-7584	0	0	0	0	0
leny Renal Papillary cell carcin	TCGA-A4-7585	0	0	0	0	0
leny Renal Papillary cell carcin	TCGA-A4-7732	0	0	0	0	0
leny Renal Papillary cell carcin	TCGA-A4-7734	0	0	0	0	0
leny Renal Papillary cell carcin	TCGA-A4-7828	0	1	0	0	0
leny Renal Papillary cell carcin	TCGA-A4-7915	0	0	1	0	1
leny Renal Papillary cell carcin	TCGA-A4-7996	1	0	0	0	0
leny Renal Papillary cell carcin	TCGA-A4-7997	1	1	1	0	1
leny Renal Papillary cell carcin	TCGA-A4-8098	1	1	0	1	1
leny Renal Papillary cell carcin	TCGA-A4-8310	1	1	1	0	1
leny Renal Papillary cell carcin	TCGA-A4-8311	0	0	0	0	0

leny Renal Papillary cell carcin	TCGA-A4-8312	0	1	0	0	0
leny Renal Papillary cell carcin	TCGA-A4-8515	0	0	0	0	0
leny Renal Papillary cell carcin	TCGA-A4-8516	0	0	0	0	0
leny Renal Papillary cell carcin	TCGA-A4-8517	0	1	1	0	1
leny Renal Papillary cell carcin	TCGA-A4-8518	0	0	0	0	0
leny Renal Papillary cell carcin	TCGA-A4-8630	0	0	0	0	0
leny Renal Papillary cell carcin	TCGA-A4-A48D	0	0	0	0	0
leny Renal Papillary cell carcin	TCGA-A4-A4ZT	0	0	0	0	0
leny Renal Papillary cell carcin	TCGA-A4-A57E	1	1	1	1	1
leny Renal Papillary cell carcin	TCGA-A4-A5DU	0	0	0	0	0
leny Renal Papillary cell carcin	TCGA-A4-A5XZ	0	1	0	0	0
leny Renal Papillary cell carcin	TCGA-A4-A5Y0	0	0	0	1	1
leny Renal Papillary cell carcin	TCGA-A4-A5Y1	1	1	1	1	1
leny Renal Papillary cell carcin	TCGA-A4-A6HP	0	0	0	0	0
leny Renal Papillary cell carcin	TCGA-A4-A772	0	0	0	0	0
leny Renal Papillary cell carcin	TCGA-A4-A7UZ	0	0	0	0	1
leny Renal Papillary cell carcin	TCGA-AL-3466	1	1	1	1	1
leny Renal Papillary cell carcin	TCGA-AL-3467	0	0	0	1	0
leny Renal Papillary cell carcin	TCGA-AL-3468	0	0	0	0	0
leny Renal Papillary cell carcin	TCGA-AL-3471	0	0	0	0	0
leny Renal Papillary cell carcin	TCGA-AL-3472	0	0	0	0	0
leny Renal Papillary cell carcin	TCGA-AL-3473	1	0	0	1	0
leny Renal Papillary cell carcin	TCGA-AL-7173	1	0	0	0	0
leny Renal Papillary cell carcin	TCGA-AL-A5DJ	1	1	1	1	1
leny Renal Papillary cell carcin	TCGA-AT-A5NU	0	0	0	0	0
leny Renal Papillary cell carcin	TCGA-B1-5398	0	0	0	1	1
leny Renal Papillary cell carcin	TCGA-B1-7332	1	1	1	1	1
leny Renal Papillary cell carcin	TCGA-B1-A47M	0	0	0	0	0
leny Renal Papillary cell carcin	TCGA-B1-A47N	0	0	0	0	0
leny Renal Papillary cell carcin	TCGA-B1-A47O	0	0	0	1	1
leny Renal Papillary cell carcin	TCGA-B1-A654	0	0	0	0	0
leny Renal Papillary cell carcin	TCGA-B1-A655	1	1	0	0	1
leny Renal Papillary cell carcin	TCGA-B1-A656	1	0	0	0	0
leny Renal Papillary cell carcin	TCGA-B1-A657	1	0	0	0	0
leny Renal Papillary cell carcin	TCGA-B3-3925	0	0	0	1	1
leny Renal Papillary cell carcin	TCGA-B3-3926	1	1	1	1	1
leny Renal Papillary cell carcin	TCGA-B3-4103	0	0	0	0	0
leny Renal Papillary cell carcin	TCGA-B3-4104	1	1	0	0	1
leny Renal Papillary cell carcin	TCGA-B3-8121	0	0	0	0	0
leny Renal Papillary cell carcin	TCGA-B3-A6W5	0	0	0	0	0
leny Renal Papillary cell carcin	TCGA-B9-4113	0	0	0	0	0
leny Renal Papillary cell carcin	TCGA-B9-4114	0	0	0	0	0
leny Renal Papillary cell carcin	TCGA-B9-4115	0	0	0	0	0
leny Renal Papillary cell carcin	TCGA-B9-4116	1	1	0	1	1
leny Renal Papillary cell carcin	TCGA-B9-4117	0	0	0	0	0
leny Renal Papillary cell carcin	TCGA-B9-4617	0	0	0	0	0
leny Renal Papillary cell carcin	TCGA-B9-5155	0	0	1	0	0
leny Renal Papillary cell carcin	TCGA-B9-5156	0	0	0	0	0
leny Renal Papillary cell carcin	TCGA-B9-7268	0	0	0	0	0
leny Renal Papillary cell carcin	TCGA-B9-A44B	1	1	1	1	1
leny Renal Papillary cell carcin	TCGA-B9-A5W7	0	0	1	0	0
leny Renal Papillary cell carcin	TCGA-B9-A5W8	1	1	0	1	1
leny Renal Papillary cell carcin	TCGA-B9-A5W9	1	0	0	0	0
leny Renal Papillary cell carcin	TCGA-B9-A69E	1	1	1	1	1
leny Renal Papillary cell carcin	TCGA-B9-A8YH	0	0	0	0	0
leny Renal Papillary cell carcin	TCGA-B9-A8YI	1	0	1	1	0
leny Renal Papillary cell carcin	TCGA-BQ-5875	1	1	1	1	1
leny Renal Papillary cell carcin	TCGA-BQ-5876	1	0	1	0	1

leny Renal Papillary cell carcin	TCGA-BQ-5877	1	1	1	1	1
leny Renal Papillary cell carcin	TCGA-BQ-5878	0	0	0	0	0
leny Renal Papillary cell carcin	TCGA-BQ-5879	1	1	1	1	1
leny Renal Papillary cell carcin	TCGA-BQ-5880	0	1	0	0	0
leny Renal Papillary cell carcin	TCGA-BQ-5881	0	0	0	0	1
leny Renal Papillary cell carcin	TCGA-BQ-5882	1	1	1	1	1
leny Renal Papillary cell carcin	TCGA-BQ-5883	0	0	0	0	0
leny Renal Papillary cell carcin	TCGA-BQ-5884	0	1	0	1	1
leny Renal Papillary cell carcin	TCGA-BQ-5885	1	1	1	1	1
leny Renal Papillary cell carcin	TCGA-BQ-5886	0	0	0	0	0
leny Renal Papillary cell carcin	TCGA-BQ-5887	1	0	1	0	1
leny Renal Papillary cell carcin	TCGA-BQ-5888	1	1	1	0	1
leny Renal Papillary cell carcin	TCGA-BQ-5889	1	1	0	1	1
leny Renal Papillary cell carcin	TCGA-BQ-5890	0	0	1	1	1
leny Renal Papillary cell carcin	TCGA-BQ-5891	1	1	1	1	1
leny Renal Papillary cell carcin	TCGA-BQ-5892	0	0	0	1	0
leny Renal Papillary cell carcin	TCGA-BQ-5893	1	1	0	0	1
leny Renal Papillary cell carcin	TCGA-BQ-5894	1	1	1	1	1
leny Renal Papillary cell carcin	TCGA-BQ-7044	1	1	0	1	1
leny Renal Papillary cell carcin	TCGA-BQ-7045	1	0	0	0	0
leny Renal Papillary cell carcin	TCGA-BQ-7046	0	0	0	0	0
leny Renal Papillary cell carcin	TCGA-BQ-7048	1	1	1	0	1
leny Renal Papillary cell carcin	TCGA-BQ-7049	0	1	0	0	1
leny Renal Papillary cell carcin	TCGA-BQ-7050	0	0	0	1	1
leny Renal Papillary cell carcin	TCGA-BQ-7051	1	0	0	1	1
leny Renal Papillary cell carcin	TCGA-BQ-7053	0	0	0	0	0
leny Renal Papillary cell carcin	TCGA-BQ-7055	1	1	0	1	1
leny Renal Papillary cell carcin	TCGA-BQ-7056	1	1	1	1	1
leny Renal Papillary cell carcin	TCGA-BQ-7058	1	0	1	0	1
leny Renal Papillary cell carcin	TCGA-BQ-7059	1	0	0	0	0
leny Renal Papillary cell carcin	TCGA-BQ-7060	0	0	0	0	0
leny Renal Papillary cell carcin	TCGA-BQ-7061	0	1	0	1	1
leny Renal Papillary cell carcin	TCGA-BQ-7062	0	0	0	0	0
leny Renal Papillary cell carcin	TCGA-DW-5560	0	1	0	1	0
leny Renal Papillary cell carcin	TCGA-DW-5561	0	0	0	0	0
leny Renal Papillary cell carcin	TCGA-DW-7834	0	0	0	0	0
leny Renal Papillary cell carcin	TCGA-DW-7836	0	0	0	0	0
leny Renal Papillary cell carcin	TCGA-DW-7837	0	0	0	0	0
leny Renal Papillary cell carcin	TCGA-DW-7838	0	0	0	0	0
leny Renal Papillary cell carcin	TCGA-DW-7839	0	0	0	0	0
leny Renal Papillary cell carcin	TCGA-DW-7840	1	0	0	0	0
leny Renal Papillary cell carcin	TCGA-DW-7841	0	0	0	0	0
leny Renal Papillary cell carcin	TCGA-DW-7842	0	0	0	0	0
leny Renal Papillary cell carcin	TCGA-DW-7963	0	0	0	0	0
leny Renal Papillary cell carcin	TCGA-DZ-6131	1	1	1	1	1
leny Renal Papillary cell carcin	TCGA-DZ-6132	1	0	0	0	0
leny Renal Papillary cell carcin	TCGA-DZ-6133	0	1	0	0	0
leny Renal Papillary cell carcin	TCGA-DZ-6134	1	0	0	0	0
leny Renal Papillary cell carcin	TCGA-DZ-6135	0	0	0	0	0
leny Renal Papillary cell carcin	TCGA-EV-5901	0	0	0	0	0
leny Renal Papillary cell carcin	TCGA-EV-5902	1	1	1	1	1
leny Renal Papillary cell carcin	TCGA-EV-5903	1	0	0	0	0
leny Renal Papillary cell carcin	TCGA-F9-A4JJ	1	1	1	1	1
leny Renal Papillary cell carcin	TCGA-F9-A7Q0	1	1	1	1	1
leny Renal Papillary cell carcin	TCGA-F9-A7VF	0	0	0	0	0
leny Renal Papillary cell carcin	TCGA-F9-A8NY	0	1	0	1	1
leny Renal Papillary cell carcin	TCGA-F9-A97G	1	0	0	0	1
leny Renal Papillary cell carcin	TCGA-G7-6789	1	1	1	1	1

leny Renal Papillary cell carcin	TCGA-G7-6790	0	0	0	0	0
leny Renal Papillary cell carcin	TCGA-G7-6792	0	0	0	0	0
leny Renal Papillary cell carcin	TCGA-G7-6793	1	1	1	1	1
leny Renal Papillary cell carcin	TCGA-G7-6795	0	0	0	0	0
leny Renal Papillary cell carcin	TCGA-G7-6796	1	1	0	1	1
leny Renal Papillary cell carcin	TCGA-G7-6797	0	1	1	1	1
leny Renal Papillary cell carcin	TCGA-G7-7501	1	1	0	1	1
leny Renal Papillary cell carcin	TCGA-G7-7502	1	1	1	1	1
leny Renal Papillary cell carcin	TCGA-G7-A4TM	0	0	0	0	0
leny Renal Papillary cell carcin	TCGA-G7-A8LB	0	0	0	0	0
leny Renal Papillary cell carcin	TCGA-G7-A8LC	1	1	1	1	1
leny Renal Papillary cell carcin	TCGA-G7-A8LD	1	1	1	1	1
leny Renal Papillary cell carcin	TCGA-G7-A8LE	0	0	0	0	0
leny Renal Papillary cell carcin	TCGA-GL-6846	0	0	0	0	1
leny Renal Papillary cell carcin	TCGA-GL-7773	1	0	0	0	0
leny Renal Papillary cell carcin	TCGA-GL-7966	1	1	1	1	1
leny Renal Papillary cell carcin	TCGA-GL-8500	1	1	0	1	0
leny Renal Papillary cell carcin	TCGA-GL-A4EM	0	0	0	0	0
leny Renal Papillary cell carcin	TCGA-GL-A59R	0	0	0	1	0
leny Renal Papillary cell carcin	TCGA-GL-A59T	0	1	0	0	0
leny Renal Papillary cell carcin	TCGA-GL-A9DC	0	0	0	0	0
leny Renal Papillary cell carcin	TCGA-GL-A9DD	0	0	0	0	0
leny Renal Papillary cell carcin	TCGA-GL-A9DE	1	0	0	0	0
leny Renal Papillary cell carcin	TCGA-HE-7128	0	0	0	0	0
leny Renal Papillary cell carcin	TCGA-HE-7129	0	0	0	1	1
leny Renal Papillary cell carcin	TCGA-HE-7130	1	1	1	1	1
leny Renal Papillary cell carcin	TCGA-HE-A5NF	0	0	0	0	0
leny Renal Papillary cell carcin	TCGA-HE-A5NH	0	0	0	0	0
leny Renal Papillary cell carcin	TCGA-HE-A5NI	0	0	0	0	0
leny Renal Papillary cell carcin	TCGA-HE-A5NJ	0	0	0	1	1
leny Renal Papillary cell carcin	TCGA-HE-A5NK	1	1	0	1	1
leny Renal Papillary cell carcin	TCGA-HE-A5NL	0	0	0	0	0
leny Renal Papillary cell carcin	TCGA-IA-A40U	1	0	0	0	0
leny Renal Papillary cell carcin	TCGA-IA-A40X	0	0	0	0	0
leny Renal Papillary cell carcin	TCGA-IA-A40Y	1	1	1	1	1
leny Renal Papillary cell carcin	TCGA-IA-A83S	0	0	0	0	0
leny Renal Papillary cell carcin	TCGA-IA-A83T	0	0	0	0	1
leny Renal Papillary cell carcin	TCGA-IA-A83V	0	0	0	0	0
leny Renal Papillary cell carcin	TCGA-IA-A83W	0	0	0	0	0
leny Renal Papillary cell carcin	TCGA-IZ-8195	0	0	0	0	0
leny Renal Papillary cell carcin	TCGA-IZ-8196	0	0	0	0	0
leny Renal Papillary cell carcin	TCGA-IZ-A6M8	0	0	0	0	0
leny Renal Papillary cell carcin	TCGA-IZ-A6M9	0	0	0	0	0
leny Renal Papillary cell carcin	TCGA-J7-6720	0	0	0	0	0
leny Renal Papillary cell carcin	TCGA-J7-8537	1	1	1	1	1
leny Renal Papillary cell carcin	TCGA-J7-A8I2	1	1	1	0	1
leny Renal Papillary cell carcin	TCGA-KV-A6GD	0	0	0	0	0
leny Renal Papillary cell carcin	TCGA-KV-A6GE	0	0	0	0	0
leny Renal Papillary cell carcin	TCGA-KV-A74V	0	1	0	0	0
leny Renal Papillary cell carcin	TCGA-MH-A55W	0	0	0	0	0
leny Renal Papillary cell carcin	TCGA-MH-A55Z	0	0	0	0	1
leny Renal Papillary cell carcin	TCGA-MH-A560	0	0	0	0	0
leny Renal Papillary cell carcin	TCGA-MH-A561	0	0	0	0	0
leny Renal Papillary cell carcin	TCGA-MH-A562	0	0	0	0	0
leny Renal Papillary cell carcin	TCGA-MH-A854	1	0	0	0	0
leny Renal Papillary cell carcin	TCGA-MH-A855	1	0	0	0	0
leny Renal Papillary cell carcin	TCGA-MH-A856	0	0	0	1	1
leny Renal Papillary cell carcin	TCGA-MH-A857	1	1	0	0	0

leny Renal Papillary cell carcin	TCGA-O9-A75Z	0	0	0	0	0
leny Renal Papillary cell carcin	TCGA-P4-A5E6	0	0	0	0	0
leny Renal Papillary cell carcin	TCGA-P4-A5E7	1	0	0	1	0
leny Renal Papillary cell carcin	TCGA-P4-A5E8	1	1	1	1	1
leny Renal Papillary cell carcin	TCGA-P4-A5EA	1	1	1	1	1
leny Renal Papillary cell carcin	TCGA-P4-A5EB	1	0	1	1	1
leny Renal Papillary cell carcin	TCGA-P4-A5ED	0	0	0	0	0
leny Renal Papillary cell carcin	TCGA-P4-AAVK	0	0	0	0	0
leny Renal Papillary cell carcin	TCGA-P4-AAVL	1	1	0	1	1
leny Renal Papillary cell carcin	TCGA-P4-AAVM	0	0	0	0	0
leny Renal Papillary cell carcin	TCGA-P4-AAVO	0	1	0	1	1
leny Renal Papillary cell carcin	TCGA-PJ-A5Z8	1	1	0	1	1
leny Renal Papillary cell carcin	TCGA-PJ-A5Z9	0	0	0	0	0
leny Renal Papillary cell carcin	TCGA-Q2-A5QZ	1	1	1	1	1
leny Renal Papillary cell carcin	TCGA-SX-A71R	0	0	0	0	0
leny Renal Papillary cell carcin	TCGA-SX-A71S	0	1	0	1	0
leny Renal Papillary cell carcin	TCGA-SX-A71U	0	0	0	0	0
leny Renal Papillary cell carcin	TCGA-SX-A71V	0	0	1	0	1
leny Renal Papillary cell carcin	TCGA-SX-A71W	0	0	0	0	0
leny Renal Papillary cell carcin	TCGA-SX-A7SL	0	0	1	1	1
leny Renal Papillary cell carcin	TCGA-SX-A7SM	1	0	0	1	1
leny Renal Papillary cell carcin	TCGA-SX-A7SN	0	0	0	0	0
leny Renal Papillary cell carcin	TCGA-SX-A7SO	1	1	0	0	0
leny Renal Papillary cell carcin	TCGA-SX-A7SP	0	0	0	0	0
leny Renal Papillary cell carcin	TCGA-SX-A7SQ	0	0	0	0	0
leny Renal Papillary cell carcin	TCGA-SX-A7SR	0	0	0	0	0
leny Renal Papillary cell carcin	TCGA-SX-A7SS	0	0	0	0	1
leny Renal Papillary cell carcin	TCGA-SX-A7SU	0	0	0	0	0
leny Renal Papillary cell carcin	TCGA-UN-AAZ9	1	1	1	1	1
leny Renal Papillary cell carcin	TCGA-UZ-A9PJ	0	0	0	0	0
leny Renal Papillary cell carcin	TCGA-UZ-A9PK	0	0	0	0	0
leny Renal Papillary cell carcin	TCGA-UZ-A9PL	1	0	0	0	0
leny Renal Papillary cell carcin	TCGA-UZ-A9PM	0	0	0	0	0
leny Renal Papillary cell carcin	TCGA-UZ-A9PN	1	1	1	1	1
leny Renal Papillary cell carcin	TCGA-UZ-A9PO	0	0	0	0	0
leny Renal Papillary cell carcin	TCGA-UZ-A9PP	0	0	1	1	1
leny Renal Papillary cell carcin	TCGA-UZ-A9PQ	1	1	1	1	1
leny Renal Papillary cell carcin	TCGA-UZ-A9PR	0	0	0	0	0
leny Renal Papillary cell carcin	TCGA-UZ-A9PS	0	1	0	0	1
leny Renal Papillary cell carcin	TCGA-UZ-A9PS	0	0	0	0	0
leny Renal Papillary cell carcin	TCGA-UZ-A9PU	0	0	0	0	0
leny Renal Papillary cell carcin	TCGA-UZ-A9PV	0	0	0	0	0
leny Renal Papillary cell carcin	TCGA-UZ-A9PX	0	0	0	0	0
leny Renal Papillary cell carcin	TCGA-UZ-A9PZ	0	0	0	0	0
leny Renal Papillary cell carcin	TCGA-UZ-A9Q0	0	0	0	0	0
leny Renal Papillary cell carcin	TCGA-UZ-A9Q1	1	0	0	0	0
leny Renal Papillary cell carcin	TCGA-V9-A7HT	0	0	0	0	0
leny Renal Papillary cell carcin	TCGA-WN-A9G9	1	1	0	1	1
leny Renal Papillary cell carcin	TCGA-WN-AB4C	0	0	1	1	1
leny Renal Papillary cell carcin	TCGA-Y8-A894	0	1	0	0	1
leny Renal Papillary cell carcin	TCGA-Y8-A895	0	0	0	0	0
leny Renal Papillary cell carcin	TCGA-Y8-A896	1	1	1	1	1
leny Renal Papillary cell carcin	TCGA-Y8-A897	1	0	0	0	0
leny Renal Papillary cell carcin	TCGA-Y8-A898	0	0	0	0	0
leny Renal Papillary cell carcin	TCGA-Y8-A8RY	0	0	0	0	1
leny Renal Papillary cell carcin	TCGA-Y8-A8RZ	1	1	0	1	1
leny Renal Papillary cell carcin	TCGA-Y8-A8S0	0	0	0	0	0
leny Renal Papillary cell carcin	TCGA-Y8-A8S1	1	0	0	1	1

.iver Hepatocecullar Carcinoma	TCGA-2V-A95S	1	0	0	0	0
.iver Hepatocecullar Carcinoma	TCGA-2Y-A9GS	1	1	1	1	1
.iver Hepatocecullar Carcinoma	TCGA-2Y-A9GT	0	0	1	0	1
.iver Hepatocecullar Carcinoma	TCGA-2Y-A9GU	1	1	1	0	1
.iver Hepatocecullar Carcinoma	TCGA-2Y-A9GV	1	0	1	0	1
.iver Hepatocecullar Carcinoma	TCGA-2Y-A9GW	1	1	1	0	0
.iver Hepatocecullar Carcinoma	TCGA-2Y-A9GX	1	1	0	1	0
.iver Hepatocecullar Carcinoma	TCGA-2Y-A9GY	1	1	1	1	1
.iver Hepatocecullar Carcinoma	TCGA-2Y-A9GZ	1	1	1	1	1
.iver Hepatocecullar Carcinoma	TCGA-2Y-A9H0	1	1	1	1	1
.iver Hepatocecullar Carcinoma	TCGA-2Y-A9H1	1	1	0	0	0
.iver Hepatocecullar Carcinoma	TCGA-2Y-A9H2	1	1	1	1	1
.iver Hepatocecullar Carcinoma	TCGA-2Y-A9H3	1	1	1	1	1
.iver Hepatocecullar Carcinoma	TCGA-2Y-A9H4	1	0	1	1	1
.iver Hepatocecullar Carcinoma	TCGA-2Y-A9H5	1	1	1	1	1
.iver Hepatocecullar Carcinoma	TCGA-2Y-A9H6	1	1	1	1	1
.iver Hepatocecullar Carcinoma	TCGA-2Y-A9H7	0	1	1	1	1
.iver Hepatocecullar Carcinoma	TCGA-2Y-A9H8	1	1	1	1	1
.iver Hepatocecullar Carcinoma	TCGA-2Y-A9H9	1	1	1	0	1
.iver Hepatocecullar Carcinoma	TCGA-2Y-A9HA	1	1	1	1	1
.iver Hepatocecullar Carcinoma	TCGA-2Y-A9HB	1	1	1	0	0
.iver Hepatocecullar Carcinoma	TCGA-3K-AAZ8	1	1	1	1	1
.iver Hepatocecullar Carcinoma	TCGA-4R-AA8I	0	1	1	0	1
.iver Hepatocecullar Carcinoma	TCGA-5C-A9VG	1	1	1	1	1
.iver Hepatocecullar Carcinoma	TCGA-5C-A9VH	1	1	0	1	1
.iver Hepatocecullar Carcinoma	TCGA-5C-AAPD	1	1	1	1	1
.iver Hepatocecullar Carcinoma	TCGA-5R-AA1C	0	1	1	0	0
.iver Hepatocecullar Carcinoma	TCGA-5R-AA1D	1	0	0	1	1
.iver Hepatocecullar Carcinoma	TCGA-5R-AAAM	1	1	1	1	1
.iver Hepatocecullar Carcinoma	TCGA-BC-4072	1	1	1	1	1
.iver Hepatocecullar Carcinoma	TCGA-BC-4073	1	0	1	0	1
.iver Hepatocecullar Carcinoma	TCGA-BC-A10Q	1	1	1	1	1
.iver Hepatocecullar Carcinoma	TCGA-BC-A10R	1	1	1	1	1
.iver Hepatocecullar Carcinoma	TCGA-BC-A10S	1	1	1	1	1
.iver Hepatocecullar Carcinoma	TCGA-BC-A10T	1	1	1	1	1
.iver Hepatocecullar Carcinoma	TCGA-BC-A10U	1	1	1	0	1
.iver Hepatocecullar Carcinoma	TCGA-BC-A10W	1	1	1	1	1
.iver Hepatocecullar Carcinoma	TCGA-BC-A10X	0	0	0	0	0
.iver Hepatocecullar Carcinoma	TCGA-BC-A10Y	1	1	1	1	1
.iver Hepatocecullar Carcinoma	TCGA-BC-A10Z	1	1	1	1	1
.iver Hepatocecullar Carcinoma	TCGA-BC-A110	0	0	1	0	1
.iver Hepatocecullar Carcinoma	TCGA-BC-A112	1	1	1	1	1
.iver Hepatocecullar Carcinoma	TCGA-BC-A216	1	1	1	1	1
.iver Hepatocecullar Carcinoma	TCGA-BC-A217	1	1	1	1	1
.iver Hepatocecullar Carcinoma	TCGA-BC-A3KF	1	1	1	1	1
.iver Hepatocecullar Carcinoma	TCGA-BC-A3KG	1	1	1	1	1
.iver Hepatocecullar Carcinoma	TCGA-BC-A5W4	1	1	1	1	1
.iver Hepatocecullar Carcinoma	TCGA-BC-A69H	1	1	1	1	1
.iver Hepatocecullar Carcinoma	TCGA-BC-A69I	0	1	1	0	0
.iver Hepatocecullar Carcinoma	TCGA-BC-A8YO	1	1	1	0	1
.iver Hepatocecullar Carcinoma	TCGA-BD-A2L6	1	0	1	0	1
.iver Hepatocecullar Carcinoma	TCGA-BD-A3EP	1	1	1	1	1
.iver Hepatocecullar Carcinoma	TCGA-BD-A3ER	1	1	1	1	0
.iver Hepatocecullar Carcinoma	TCGA-BW-A5NO	1	1	1	1	1
.iver Hepatocecullar Carcinoma	TCGA-BW-A5NP	1	1	1	1	1
.iver Hepatocecullar Carcinoma	TCGA-BW-A5NQ	1	1	1	1	1
.iver Hepatocecullar Carcinoma	TCGA-CC-5258	1	1	1	1	1
.iver Hepatocecullar Carcinoma	TCGA-CC-5260	1	1	1	1	1

.iver Hepatocecullar Carcinoma	TCGA-CC-5261	1	1	1	1	1
.iver Hepatocecullar Carcinoma	TCGA-CC-5262	0	1	1	0	1
.iver Hepatocecullar Carcinoma	TCGA-CC-5263	1	1	1	1	1
.iver Hepatocecullar Carcinoma	TCGA-CC-5264	1	1	1	1	1
.iver Hepatocecullar Carcinoma	TCGA-CC-A1HT	1	1	1	1	1
.iver Hepatocecullar Carcinoma	TCGA-CC-A3M9	1	1	1	1	1
.iver Hepatocecullar Carcinoma	TCGA-CC-A3MA	1	1	1	1	1
.iver Hepatocecullar Carcinoma	TCGA-CC-A3MB	1	1	1	1	1
.iver Hepatocecullar Carcinoma	TCGA-CC-A3MC	1	1	1	1	1
.iver Hepatocecullar Carcinoma	TCGA-CC-A5UC	0	1	1	1	1
.iver Hepatocecullar Carcinoma	TCGA-CC-A5UD	1	1	1	1	1
.iver Hepatocecullar Carcinoma	TCGA-CC-A5UE	1	1	1	1	1
.iver Hepatocecullar Carcinoma	TCGA-CC-A7IE	1	1	0	1	1
.iver Hepatocecullar Carcinoma	TCGA-CC-A7IF	1	1	1	1	1
.iver Hepatocecullar Carcinoma	TCGA-CC-A7IG	0	1	1	1	1
.iver Hepatocecullar Carcinoma	TCGA-CC-A7IH	1	1	0	1	0
.iver Hepatocecullar Carcinoma	TCGA-CC-A7II	1	1	1	1	1
.iver Hepatocecullar Carcinoma	TCGA-CC-A7IJ	1	1	1	1	1
.iver Hepatocecullar Carcinoma	TCGA-CC-A7IK	1	1	1	1	1
.iver Hepatocecullar Carcinoma	TCGA-CC-A7IL	1	1	1	1	1
.iver Hepatocecullar Carcinoma	TCGA-CC-A8HT	1	1	1	1	1
.iver Hepatocecullar Carcinoma	TCGA-CC-A8HU	1	1	1	1	1
.iver Hepatocecullar Carcinoma	TCGA-CC-A8HV	1	1	1	1	1
.iver Hepatocecullar Carcinoma	TCGA-CC-A9FS	1	1	1	1	1
.iver Hepatocecullar Carcinoma	TCGA-CC-A9FU	1	1	1	1	1
.iver Hepatocecullar Carcinoma	TCGA-CC-A9FV	0	0	0	0	0
.iver Hepatocecullar Carcinoma	TCGA-CC-A9FW	1	1	1	1	1
.iver Hepatocecullar Carcinoma	TCGA-DD-A113	1	1	1	1	1
.iver Hepatocecullar Carcinoma	TCGA-DD-A114	1	1	1	1	1
.iver Hepatocecullar Carcinoma	TCGA-DD-A115	1	1	1	1	1
.iver Hepatocecullar Carcinoma	TCGA-DD-A116	1	1	1	1	1
.iver Hepatocecullar Carcinoma	TCGA-DD-A118	1	1	1	1	1
.iver Hepatocecullar Carcinoma	TCGA-DD-A119	1	1	1	1	1
.iver Hepatocecullar Carcinoma	TCGA-DD-A11A	1	1	1	1	1
.iver Hepatocecullar Carcinoma	TCGA-DD-A11B	1	1	0	1	1
.iver Hepatocecullar Carcinoma	TCGA-DD-A11C	1	1	1	1	1
.iver Hepatocecullar Carcinoma	TCGA-DD-A11D	0	1	1	0	1
.iver Hepatocecullar Carcinoma	TCGA-DD-A1EA	0	0	1	0	1
.iver Hepatocecullar Carcinoma	TCGA-DD-A1EB	0	1	1	0	1
.iver Hepatocecullar Carcinoma	TCGA-DD-A1EC	0	0	1	0	0
.iver Hepatocecullar Carcinoma	TCGA-DD-A1ED	0	0	0	0	0
.iver Hepatocecullar Carcinoma	TCGA-DD-A1EE	1	1	1	0	1
.iver Hepatocecullar Carcinoma	TCGA-DD-A1EF	1	1	1	1	1
.iver Hepatocecullar Carcinoma	TCGA-DD-A1EG	1	1	1	1	1
.iver Hepatocecullar Carcinoma	TCGA-DD-A1EH	1	1	1	1	1
.iver Hepatocecullar Carcinoma	TCGA-DD-A1EI	1	1	1	1	1
.iver Hepatocecullar Carcinoma	TCGA-DD-A1EJ	1	1	1	1	1
.iver Hepatocecullar Carcinoma	TCGA-DD-A1EK	1	1	1	0	1
.iver Hepatocecullar Carcinoma	TCGA-DD-A1EL	1	1	1	1	1
.iver Hepatocecullar Carcinoma	TCGA-DD-A39V	1	1	0	1	1
.iver Hepatocecullar Carcinoma	TCGA-DD-A39W	0	0	0	0	1
.iver Hepatocecullar Carcinoma	TCGA-DD-A39X	1	1	1	1	1
.iver Hepatocecullar Carcinoma	TCGA-DD-A39Y	1	1	1	1	1
.iver Hepatocecullar Carcinoma	TCGA-DD-A39Z	0	1	1	1	1
.iver Hepatocecullar Carcinoma	TCGA-DD-A3A1	1	1	1	1	1
.iver Hepatocecullar Carcinoma	TCGA-DD-A3A2	0	0	0	0	1
.iver Hepatocecullar Carcinoma	TCGA-DD-A3A3	1	1	1	1	1
.iver Hepatocecullar Carcinoma	TCGA-DD-A3A4	0	1	1	0	1

.iver Hepatocecullar Carcinoma	TCGA-DD-A3A5	1	0	1	1	1
.iver Hepatocecullar Carcinoma	TCGA-DD-A3A6	0	0	0	1	1
.iver Hepatocecullar Carcinoma	TCGA-DD-A3A7	1	1	1	1	1
.iver Hepatocecullar Carcinoma	TCGA-DD-A3A8	1	1	1	1	1
.iver Hepatocecullar Carcinoma	TCGA-DD-A3A9	1	1	1	1	1
.iver Hepatocecullar Carcinoma	TCGA-DD-A4NA	1	1	1	1	1
.iver Hepatocecullar Carcinoma	TCGA-DD-A4NB	1	1	1	1	1
.iver Hepatocecullar Carcinoma	TCGA-DD-A4ND	1	1	0	1	1
.iver Hepatocecullar Carcinoma	TCGA-DD-A4NF	0	0	0	0	0
.iver Hepatocecullar Carcinoma	TCGA-DD-A4NH	1	1	1	1	1
.iver Hepatocecullar Carcinoma	TCGA-DD-A4NI	1	1	1	1	1
.iver Hepatocecullar Carcinoma	TCGA-DD-A4NJ	1	1	1	1	1
.iver Hepatocecullar Carcinoma	TCGA-DD-A4NK	0	1	1	0	0
.iver Hepatocecullar Carcinoma	TCGA-DD-A4NL	0	0	0	0	0
.iver Hepatocecullar Carcinoma	TCGA-DD-A4NN	1	1	0	1	1
.iver Hepatocecullar Carcinoma	TCGA-DD-A4NO	1	1	1	1	1
.iver Hepatocecullar Carcinoma	TCGA-DD-A4NP	0	1	0	1	1
.iver Hepatocecullar Carcinoma	TCGA-DD-A4NQ	1	1	1	1	1
.iver Hepatocecullar Carcinoma	TCGA-DD-A4NR	0	1	0	0	0
.iver Hepatocecullar Carcinoma	TCGA-DD-A4NS	0	1	1	0	0
.iver Hepatocecullar Carcinoma	TCGA-DD-A4NV	0	1	0	0	1
.iver Hepatocecullar Carcinoma	TCGA-DD-A73A	1	1	0	1	1
.iver Hepatocecullar Carcinoma	TCGA-DD-A73B	1	1	1	1	1
.iver Hepatocecullar Carcinoma	TCGA-DD-A73C	0	1	0	0	0
.iver Hepatocecullar Carcinoma	TCGA-DD-A73D	0	1	1	0	0
.iver Hepatocecullar Carcinoma	TCGA-DD-A73E	1	1	1	0	1
.iver Hepatocecullar Carcinoma	TCGA-DD-A73F	1	1	1	1	1
.iver Hepatocecullar Carcinoma	TCGA-DD-A73G	1	1	1	1	0
.iver Hepatocecullar Carcinoma	TCGA-DD-AA3A	1	1	1	0	1
.iver Hepatocecullar Carcinoma	TCGA-DD-AAC8	1	1	1	1	1
.iver Hepatocecullar Carcinoma	TCGA-DD-AAC9	0	0	1	0	1
.iver Hepatocecullar Carcinoma	TCGA-DD-AACA	1	1	1	1	1
.iver Hepatocecullar Carcinoma	TCGA-DD-AACB	1	1	1	1	1
.iver Hepatocecullar Carcinoma	TCGA-DD-AACC	0	1	1	1	1
.iver Hepatocecullar Carcinoma	TCGA-DD-AACD	1	1	1	1	1
.iver Hepatocecullar Carcinoma	TCGA-DD-AACE	1	1	1	1	1
.iver Hepatocecullar Carcinoma	TCGA-DD-AACF	1	0	1	0	1
.iver Hepatocecullar Carcinoma	TCGA-DD-AACG	1	1	1	1	1
.iver Hepatocecullar Carcinoma	TCGA-DD-AACH	1	0	1	1	1
.iver Hepatocecullar Carcinoma	TCGA-DD-AACI	1	0	1	0	1
.iver Hepatocecullar Carcinoma	TCGA-DD-AACJ	0	1	1	1	1
.iver Hepatocecullar Carcinoma	TCGA-DD-AACK	0	1	0	1	1
.iver Hepatocecullar Carcinoma	TCGA-DD-AACL	1	1	1	1	1
.iver Hepatocecullar Carcinoma	TCGA-DD-AACN	1	1	1	1	1
.iver Hepatocecullar Carcinoma	TCGA-DD-AACO	1	1	1	1	1
.iver Hepatocecullar Carcinoma	TCGA-DD-AACP	1	1	1	1	1
.iver Hepatocecullar Carcinoma	TCGA-DD-AACQ	1	1	1	0	1
.iver Hepatocecullar Carcinoma	TCGA-DD-AACS	1	1	1	0	1
.iver Hepatocecullar Carcinoma	TCGA-DD-AACT	1	1	1	0	0
.iver Hepatocecullar Carcinoma	TCGA-DD-AACU	1	1	1	1	1
.iver Hepatocecullar Carcinoma	TCGA-DD-AACV	1	1	1	0	1
.iver Hepatocecullar Carcinoma	TCGA-DD-AACW	1	1	0	1	1
.iver Hepatocecullar Carcinoma	TCGA-DD-AACX	1	0	1	0	1
.iver Hepatocecullar Carcinoma	TCGA-DD-AACY	1	0	1	1	1
.iver Hepatocecullar Carcinoma	TCGA-DD-AACZ	1	1	1	1	1
.iver Hepatocecullar Carcinoma	TCGA-DD-AAD0	1	1	1	1	1
.iver Hepatocecullar Carcinoma	TCGA-DD-AAD1	1	1	1	1	1
.iver Hepatocecullar Carcinoma	TCGA-DD-AAD2	0	1	0	0	1

.iver Hepatocecullar Carcinom:	TCGA-DD-AAD3	1	1	1	0	1
.iver Hepatocecullar Carcinom:	TCGA-DD-AAD5	1	1	1	1	1
.iver Hepatocecullar Carcinom:	TCGA-DD-AAD6	1	1	1	1	1
.iver Hepatocecullar Carcinom:	TCGA-DD-AAD8	1	1	1	1	1
.iver Hepatocecullar Carcinom:	TCGA-DD-AADA	1	1	0	1	1
.iver Hepatocecullar Carcinom:	TCGA-DD-AADB	1	1	1	1	1
.iver Hepatocecullar Carcinom:	TCGA-DD-AADC	1	1	1	1	1
.iver Hepatocecullar Carcinom:	TCGA-DD-AADD	1	1	1	0	1
.iver Hepatocecullar Carcinom:	TCGA-DD-AADF	1	1	1	1	1
.iver Hepatocecullar Carcinom:	TCGA-DD-AADG	0	1	1	0	1
.iver Hepatocecullar Carcinom:	TCGA-DD-AADI	1	1	1	1	1
.iver Hepatocecullar Carcinom:	TCGA-DD-AADJ	1	1	0	1	1
.iver Hepatocecullar Carcinom:	TCGA-DD-AADK	1	1	1	1	1
.iver Hepatocecullar Carcinom:	TCGA-DD-AADL	1	1	1	1	1
.iver Hepatocecullar Carcinom:	TCGA-DD-AADM	1	1	1	1	1
.iver Hepatocecullar Carcinom:	TCGA-DD-AADN	1	1	1	1	1
.iver Hepatocecullar Carcinom:	TCGA-DD-AADO	1	1	1	1	1
.iver Hepatocecullar Carcinom:	TCGA-DD-AADP	1	1	1	1	1
.iver Hepatocecullar Carcinom:	TCGA-DD-AADQ	0	1	1	1	1
.iver Hepatocecullar Carcinom:	TCGA-DD-AADR	1	1	1	1	1
.iver Hepatocecullar Carcinom:	TCGA-DD-AADS	0	0	0	1	1
.iver Hepatocecullar Carcinom:	TCGA-DD-AADU	1	0	0	1	1
.iver Hepatocecullar Carcinom:	TCGA-DD-AADV	1	1	1	1	1
.iver Hepatocecullar Carcinom:	TCGA-DD-AADW	1	1	1	1	1
.iver Hepatocecullar Carcinom:	TCGA-DD-AADY	1	1	1	1	1
.iver Hepatocecullar Carcinom:	TCGA-DD-AAE0	1	1	1	1	1
.iver Hepatocecullar Carcinom:	TCGA-DD-AAE1	0	1	1	1	1
.iver Hepatocecullar Carcinom:	TCGA-DD-AAE2	1	1	1	0	1
.iver Hepatocecullar Carcinom:	TCGA-DD-AAE3	1	1	1	1	1
.iver Hepatocecullar Carcinom:	TCGA-DD-AAE4	1	1	1	1	1
.iver Hepatocecullar Carcinom:	TCGA-DD-AAE6	1	1	1	0	1
.iver Hepatocecullar Carcinom:	TCGA-DD-AAE7	0	1	0	1	0
.iver Hepatocecullar Carcinom:	TCGA-DD-AAE9	0	1	1	0	0
.iver Hepatocecullar Carcinom:	TCGA-DD-AAEA	1	1	1	1	1
.iver Hepatocecullar Carcinom:	TCGA-DD-AAEB	0	1	0	0	1
.iver Hepatocecullar Carcinom:	TCGA-DD-AAED	1	1	1	1	1
.iver Hepatocecullar Carcinom:	TCGA-DD-AAEE	1	1	1	1	1
.iver Hepatocecullar Carcinom:	TCGA-DD-AAEG	1	0	1	1	0
.iver Hepatocecullar Carcinom:	TCGA-DD-AAEH	1	1	1	0	0
.iver Hepatocecullar Carcinom:	TCGA-DD-AAEI	1	1	1	1	1
.iver Hepatocecullar Carcinom:	TCGA-DD-AAEK	1	1	1	1	1
.iver Hepatocecullar Carcinom:	TCGA-DD-AAVP	1	0	1	0	1
.iver Hepatocecullar Carcinom:	TCGA-DD-AAVQ	1	1	1	1	1
.iver Hepatocecullar Carcinom:	TCGA-DD-AAVR	1	0	1	1	1
.iver Hepatocecullar Carcinom:	TCGA-DD-AAVS	1	1	1	1	1
.iver Hepatocecullar Carcinom:	TCGA-DD-AAVU	1	1	1	1	1
.iver Hepatocecullar Carcinom:	TCGA-DD-AAVV	1	0	1	1	1
.iver Hepatocecullar Carcinom:	TCGA-DD-AAVW	1	1	1	1	1
.iver Hepatocecullar Carcinom:	TCGA-DD-AAVX	0	0	0	0	1
.iver Hepatocecullar Carcinom:	TCGA-DD-AAVY	0	1	1	0	1
.iver Hepatocecullar Carcinom:	TCGA-DD-AAVZ	1	1	0	1	1
.iver Hepatocecullar Carcinom:	TCGA-DD-AAW0	1	1	0	1	1
.iver Hepatocecullar Carcinom:	TCGA-DD-AAW1	0	0	1	0	1
.iver Hepatocecullar Carcinom:	TCGA-DD-AAW2	0	1	1	0	0
.iver Hepatocecullar Carcinom:	TCGA-DD-AAW3	1	1	1	1	1
.iver Hepatocecullar Carcinom:	TCGA-ED-A459	1	1	1	1	1
.iver Hepatocecullar Carcinom:	TCGA-ED-A4XI	0	1	1	0	1
.iver Hepatocecullar Carcinom:	TCGA-ED-A5KG	1	1	0	1	0

.iver Hepatocecullar Carcinoma	TCGA-ED-A627	0	0	0	0	0
.iver Hepatocecullar Carcinoma	TCGA-ED-A66X	1	1	0	1	1
.iver Hepatocecullar Carcinoma	TCGA-ED-A66Y	1	1	1	1	1
.iver Hepatocecullar Carcinoma	TCGA-ED-A7PX	1	1	1	1	1
.iver Hepatocecullar Carcinoma	TCGA-ED-A7PY	1	1	0	1	1
.iver Hepatocecullar Carcinoma	TCGA-ED-A7PZ	1	1	1	1	1
.iver Hepatocecullar Carcinoma	TCGA-ED-A7XO	1	1	1	1	1
.iver Hepatocecullar Carcinoma	TCGA-ED-A7XP	1	1	1	1	1
.iver Hepatocecullar Carcinoma	TCGA-ED-A82E	1	1	1	1	1
.iver Hepatocecullar Carcinoma	TCGA-ED-A8O5	1	1	1	1	1
.iver Hepatocecullar Carcinoma	TCGA-ED-A8O6	1	1	1	1	1
.iver Hepatocecullar Carcinoma	TCGA-ED-A97K	1	1	1	1	1
.iver Hepatocecullar Carcinoma	TCGA-EP-A12J	1	1	1	1	1
.iver Hepatocecullar Carcinoma	TCGA-EP-A26S	0	1	0	0	0
.iver Hepatocecullar Carcinoma	TCGA-EP-A2KA	1	1	1	1	1
.iver Hepatocecullar Carcinoma	TCGA-EP-A2KB	1	1	0	1	1
.iver Hepatocecullar Carcinoma	TCGA-EP-A2KC	1	1	1	1	1
.iver Hepatocecullar Carcinoma	TCGA-EP-A3JL	1	0	0	1	1
.iver Hepatocecullar Carcinoma	TCGA-EP-A3RK	1	1	1	1	1
.iver Hepatocecullar Carcinoma	TCGA-ES-A2HS	1	1	1	1	1
.iver Hepatocecullar Carcinoma	TCGA-ES-A2HT	1	1	1	1	1
.iver Hepatocecullar Carcinoma	TCGA-FV-A23B	1	1	1	1	1
.iver Hepatocecullar Carcinoma	TCGA-FV-A2QQ	1	1	1	1	1
.iver Hepatocecullar Carcinoma	TCGA-FV-A2QR	1	1	1	0	1
.iver Hepatocecullar Carcinoma	TCGA-FV-A3I0	1	1	1	1	1
.iver Hepatocecullar Carcinoma	TCGA-FV-A3I1	1	1	1	1	1
.iver Hepatocecullar Carcinoma	TCGA-FV-A3R2	1	1	1	1	1
.iver Hepatocecullar Carcinoma	TCGA-FV-A3R3	1	1	0	1	0
.iver Hepatocecullar Carcinoma	TCGA-FV-A495	1	1	1	1	1
.iver Hepatocecullar Carcinoma	TCGA-FV-A496	1	1	1	1	1
.iver Hepatocecullar Carcinoma	TCGA-FV-A4ZP	1	1	1	1	1
.iver Hepatocecullar Carcinoma	TCGA-FV-A4ZQ	0	1	0	0	1
.iver Hepatocecullar Carcinoma	TCGA-G3-A25S	1	1	1	1	1
.iver Hepatocecullar Carcinoma	TCGA-G3-A25T	1	1	1	1	1
.iver Hepatocecullar Carcinoma	TCGA-G3-A25U	1	0	1	0	1
.iver Hepatocecullar Carcinoma	TCGA-G3-A25V	1	1	1	1	1
.iver Hepatocecullar Carcinoma	TCGA-G3-A25X	1	1	1	1	1
.iver Hepatocecullar Carcinoma	TCGA-G3-A25Y	0	1	1	1	1
.iver Hepatocecullar Carcinoma	TCGA-G3-A25Z	1	1	1	1	1
.iver Hepatocecullar Carcinoma	TCGA-G3-A3CG	1	1	0	0	0
.iver Hepatocecullar Carcinoma	TCGA-G3-A3CH	1	1	1	1	1
.iver Hepatocecullar Carcinoma	TCGA-G3-A3CI	1	0	0	1	1
.iver Hepatocecullar Carcinoma	TCGA-G3-A3CJ	0	1	0	0	1
.iver Hepatocecullar Carcinoma	TCGA-G3-A3CK	0	0	0	0	0
.iver Hepatocecullar Carcinoma	TCGA-G3-A5SI	0	1	1	1	1
.iver Hepatocecullar Carcinoma	TCGA-G3-A5SJ	1	1	1	1	1
.iver Hepatocecullar Carcinoma	TCGA-G3-A5SL	0	1	1	1	0
.iver Hepatocecullar Carcinoma	TCGA-G3-A5SM	1	1	1	1	1
.iver Hepatocecullar Carcinoma	TCGA-G3-A6UC	1	1	1	0	0
.iver Hepatocecullar Carcinoma	TCGA-G3-A7M5	1	1	1	1	1
.iver Hepatocecullar Carcinoma	TCGA-G3-A7M6	1	1	1	0	1
.iver Hepatocecullar Carcinoma	TCGA-G3-A7M7	0	1	0	0	1
.iver Hepatocecullar Carcinoma	TCGA-G3-A7M8	1	0	0	0	0
.iver Hepatocecullar Carcinoma	TCGA-G3-A7M9	1	1	1	1	1
.iver Hepatocecullar Carcinoma	TCGA-G3-AAUZ	0	1	1	0	1
.iver Hepatocecullar Carcinoma	TCGA-G3-AAV0	1	1	0	0	1
.iver Hepatocecullar Carcinoma	TCGA-G3-AAV1	1	1	1	1	1
.iver Hepatocecullar Carcinoma	TCGA-G3-AAV2	0	1	0	0	1

.iver Hepatocecullar Carcinom‹	TCGA-G3-AAV3	1	0	1	1	1
.iver Hepatocecullar Carcinom‹	TCGA-G3-AAV4	1	1	1	1	1
.iver Hepatocecullar Carcinom‹	TCGA-G3-AAV5	1	1	1	1	1
.iver Hepatocecullar Carcinom‹	TCGA-G3-AAV6	1	1	1	1	1
.iver Hepatocecullar Carcinom‹	TCGA-G3-AAV7	1	1	1	1	1
.iver Hepatocecullar Carcinom‹	TCGA-GJ-A3OU	1	1	1	1	1
.iver Hepatocecullar Carcinom‹	TCGA-GJ-A6C0	1	1	1	0	1
.iver Hepatocecullar Carcinom‹	TCGA-GJ-A9DB	1	1	1	0	1
.iver Hepatocecullar Carcinom‹	TCGA-HP-A5MZ	1	1	0	1	1
.iver Hepatocecullar Carcinom‹	TCGA-HP-A5N0	1	0	1	0	1
.iver Hepatocecullar Carcinom‹	TCGA-K7-A5RF	0	0	0	0	0
.iver Hepatocecullar Carcinom‹	TCGA-K7-A5RG	1	0	0	1	1
.iver Hepatocecullar Carcinom‹	TCGA-K7-A6G5	1	1	0	1	1
.iver Hepatocecullar Carcinom‹	TCGA-K7-AAU7	1	1	1	1	1
.iver Hepatocecullar Carcinom‹	TCGA-KR-A7K0	0	0	1	0	1
.iver Hepatocecullar Carcinom‹	TCGA-KR-A7K2	1	1	1	1	1
.iver Hepatocecullar Carcinom‹	TCGA-KR-A7K7	1	1	0	1	1
.iver Hepatocecullar Carcinom‹	TCGA-KR-A7K8	1	1	1	1	1
.iver Hepatocecullar Carcinom‹	TCGA-LG-A6GG	1	1	1	0	1
.iver Hepatocecullar Carcinom‹	TCGA-LG-A9QC	1	1	0	1	1
.iver Hepatocecullar Carcinom‹	TCGA-LG-A9QD	1	1	0	1	1
.iver Hepatocecullar Carcinom‹	TCGA-MI-A75C	0	1	1	0	0
.iver Hepatocecullar Carcinom‹	TCGA-MI-A75E	0	1	1	0	1
.iver Hepatocecullar Carcinom‹	TCGA-MI-A75G	1	1	1	1	1
.iver Hepatocecullar Carcinom‹	TCGA-MI-A75H	0	1	1	0	0
.iver Hepatocecullar Carcinom‹	TCGA-MI-A75I	1	1	1	1	1
.iver Hepatocecullar Carcinom‹	TCGA-MR-A520	0	0	0	0	0
.iver Hepatocecullar Carcinom‹	TCGA-MR-A8JO	1	1	1	1	1
.iver Hepatocecullar Carcinom‹	TCGA-NI-A4U2	1	0	1	0	1
.iver Hepatocecullar Carcinom‹	TCGA-NI-A8LF	1	1	1	0	1
.iver Hepatocecullar Carcinom‹	TCGA-O8-A75V	1	1	1	1	1
.iver Hepatocecullar Carcinom‹	TCGA-PD-A5DF	1	1	1	0	1
.iver Hepatocecullar Carcinom‹	TCGA-QA-A7B7	1	1	1	1	1
.iver Hepatocecullar Carcinom‹	TCGA-RC-A6M3	1	1	1	1	1
.iver Hepatocecullar Carcinom‹	TCGA-RC-A6M4	1	1	1	1	1
.iver Hepatocecullar Carcinom‹	TCGA-RC-A6M5	1	1	1	1	1
.iver Hepatocecullar Carcinom‹	TCGA-RC-A6M6	1	1	1	1	1
.iver Hepatocecullar Carcinom‹	TCGA-RC-A7S9	1	1	0	0	1
.iver Hepatocecullar Carcinom‹	TCGA-RC-A7SB	1	1	1	0	1
.iver Hepatocecullar Carcinom‹	TCGA-RC-A7SF	1	1	0	0	1
.iver Hepatocecullar Carcinom‹	TCGA-RC-A7SH	1	1	1	1	1
.iver Hepatocecullar Carcinom‹	TCGA-RC-A7SK	1	1	1	1	1
.iver Hepatocecullar Carcinom‹	TCGA-RG-A7D4	1	1	1	1	1
.iver Hepatocecullar Carcinom‹	TCGA-T1-A6J8	1	1	1	1	1
.iver Hepatocecullar Carcinom‹	TCGA-UB-A7MA	1	1	1	1	1
.iver Hepatocecullar Carcinom‹	TCGA-UB-A7MB	1	1	1	0	1
.iver Hepatocecullar Carcinom‹	TCGA-UB-A7MC	1	1	1	1	1
.iver Hepatocecullar Carcinom‹	TCGA-UB-A7MD	1	1	1	1	1
.iver Hepatocecullar Carcinom‹	TCGA-UB-A7ME	1	1	1	0	1
.iver Hepatocecullar Carcinom‹	TCGA-UB-A7MF	1	1	1	1	1
.iver Hepatocecullar Carcinom‹	TCGA-UB-AA0U	1	1	1	1	1
.iver Hepatocecullar Carcinom‹	TCGA-UB-AA0V	0	0	0	0	0
.iver Hepatocecullar Carcinom‹	TCGA-WJ-A86L	1	1	1	1	1
.iver Hepatocecullar Carcinom‹	TCGA-WQ-A9G7	1	1	1	1	1
.iver Hepatocecullar Carcinom‹	TCGA-WQ-AB4B	1	1	1	1	1
.iver Hepatocecullar Carcinom‹	TCGA-WX-AA44	1	1	1	1	1
.iver Hepatocecullar Carcinom‹	TCGA-WX-AA46	0	0	0	0	0
.iver Hepatocecullar Carcinom‹	TCGA-WX-AA47	1	1	0	1	0

.iver Hepatocecullar Carcinoma	TCGA-XR-A8TD	1	1	1	1	1
.iver Hepatocecullar Carcinoma	TCGA-XR-A8TE	0	0	0	0	0
.iver Hepatocecullar Carcinoma	TCGA-XR-A8TF	1	1	1	1	1
.iver Hepatocecullar Carcinoma	TCGA-XR-A8TG	1	1	1	0	1
.iver Hepatocecullar Carcinoma	TCGA-YA-A8S7	1	1	1	1	1
.iver Hepatocecullar Carcinoma	TCGA-ZP-A9CV	1	1	0	0	0
.iver Hepatocecullar Carcinoma	TCGA-ZP-A9CY	1	0	0	0	0
.iver Hepatocecullar Carcinoma	TCGA-ZP-A9CZ	1	1	0	1	1
.iver Hepatocecullar Carcinoma	TCGA-ZP-A9D0	1	1	0	0	1
.iver Hepatocecullar Carcinoma	TCGA-ZP-A9D1	1	1	1	1	1
.iver Hepatocecullar Carcinoma	TCGA-ZP-A9D2	1	1	1	0	1
.iver Hepatocecullar Carcinoma	TCGA-ZP-A9D4	1	1	1	1	1
.iver Hepatocecullar Carcinoma	TCGA-ZS-A9CD	1	1	0	1	1
.iver Hepatocecullar Carcinoma	TCGA-ZS-A9CE	1	1	1	1	1
.iver Hepatocecullar Carcinoma	TCGA-ZS-A9CF	1	1	1	1	1
.iver Hepatocecullar Carcinoma	TCGA-ZS-A9CG	1	1	0	1	1
Lung Adenocarcinoma	TCGA-05-4244	1	1	1	1	1
Lung Adenocarcinoma	TCGA-05-4249	1	1	1	1	1
Lung Adenocarcinoma	TCGA-05-4250	1	1	1	1	1
Lung Adenocarcinoma	TCGA-05-4382	1	1	1	1	1
Lung Adenocarcinoma	TCGA-05-4384	1	1	1	0	1
Lung Adenocarcinoma	TCGA-05-4389	1	1	1	1	1
Lung Adenocarcinoma	TCGA-05-4390	1	1	1	1	1
Lung Adenocarcinoma	TCGA-05-4395	1	1	1	1	1
Lung Adenocarcinoma	TCGA-05-4396	1	1	1	1	1
Lung Adenocarcinoma	TCGA-05-4397	1	1	1	1	1
Lung Adenocarcinoma	TCGA-05-4398	1	1	1	1	1
Lung Adenocarcinoma	TCGA-05-4402	1	1	1	1	1
Lung Adenocarcinoma	TCGA-05-4403	0	0	0	0	1
Lung Adenocarcinoma	TCGA-05-4405	1	1	1	0	1
Lung Adenocarcinoma	TCGA-05-4410	1	1	1	1	1
Lung Adenocarcinoma	TCGA-05-4415	1	1	1	1	1
Lung Adenocarcinoma	TCGA-05-4417	1	1	1	1	1
Lung Adenocarcinoma	TCGA-05-4418	1	1	1	1	1
Lung Adenocarcinoma	TCGA-05-4420	1	1	1	1	1
Lung Adenocarcinoma	TCGA-05-4422	1	1	1	1	1
Lung Adenocarcinoma	TCGA-05-4424	1	1	1	1	1
Lung Adenocarcinoma	TCGA-05-4425	1	1	1	1	1
Lung Adenocarcinoma	TCGA-05-4426	1	1	1	1	1
Lung Adenocarcinoma	TCGA-05-4427	1	1	1	1	1
Lung Adenocarcinoma	TCGA-05-4430	1	0	1	1	1
Lung Adenocarcinoma	TCGA-05-4432	1	1	1	1	1
Lung Adenocarcinoma	TCGA-05-4433	1	0	1	1	1
Lung Adenocarcinoma	TCGA-05-4434	1	1	1	1	1
Lung Adenocarcinoma	TCGA-05-5420	1	1	1	1	1
Lung Adenocarcinoma	TCGA-05-5423	1	1	0	0	1
Lung Adenocarcinoma	TCGA-05-5425	1	1	1	1	1
Lung Adenocarcinoma	TCGA-05-5428	1	1	1	1	1
Lung Adenocarcinoma	TCGA-05-5429	1	1	1	1	1
Lung Adenocarcinoma	TCGA-05-5715	1	1	1	1	1
Lung Adenocarcinoma	TCGA-35-3615	0	1	1	0	0
Lung Adenocarcinoma	TCGA-35-4122	1	1	1	1	1
Lung Adenocarcinoma	TCGA-35-4123	1	1	1	1	1
Lung Adenocarcinoma	TCGA-35-5375	1	1	1	1	1
Lung Adenocarcinoma	TCGA-38-4625	1	1	1	1	1
Lung Adenocarcinoma	TCGA-38-4626	0	1	0	0	0
Lung Adenocarcinoma	TCGA-38-4627	1	1	0	1	1
Lung Adenocarcinoma	TCGA-38-4628	1	1	1	1	1

Lung Adenocarcinoma	TCGA-38-4629	1	1	1	0	1
Lung Adenocarcinoma	TCGA-38-4630	1	1	1	1	1
Lung Adenocarcinoma	TCGA-38-4631	1	1	1	1	1
Lung Adenocarcinoma	TCGA-38-4632	1	1	1	1	1
Lung Adenocarcinoma	TCGA-38-6178	1	1	1	1	1
Lung Adenocarcinoma	TCGA-38-7271	0	0	0	0	1
Lung Adenocarcinoma	TCGA-38-A44F	1	1	0	0	1
Lung Adenocarcinoma	TCGA-44-2655	1	1	1	0	1
Lung Adenocarcinoma	TCGA-44-2656	1	1	1	1	1
Lung Adenocarcinoma	TCGA-44-2657	1	1	1	1	1
Lung Adenocarcinoma	TCGA-44-2659	1	1	1	1	1
Lung Adenocarcinoma	TCGA-44-2661	1	1	1	1	1
Lung Adenocarcinoma	TCGA-44-2662	1	1	1	1	1
Lung Adenocarcinoma	TCGA-44-2665	1	1	1	1	1
Lung Adenocarcinoma	TCGA-44-2666	1	1	1	1	1
Lung Adenocarcinoma	TCGA-44-2668	1	1	1	1	1
Lung Adenocarcinoma	TCGA-44-3396	1	0	1	0	1
Lung Adenocarcinoma	TCGA-44-3398	1	0	0	0	1
Lung Adenocarcinoma	TCGA-44-3918	1	1	1	1	1
Lung Adenocarcinoma	TCGA-44-3919	1	0	1	1	1
Lung Adenocarcinoma	TCGA-44-4112	1	1	1	1	1
Lung Adenocarcinoma	TCGA-44-5643	1	1	1	1	1
Lung Adenocarcinoma	TCGA-44-5644	1	1	1	1	1
Lung Adenocarcinoma	TCGA-44-5645	1	0	0	0	1
Lung Adenocarcinoma	TCGA-44-6145	1	1	1	1	1
Lung Adenocarcinoma	TCGA-44-6146	1	1	1	0	1
Lung Adenocarcinoma	TCGA-44-6147	1	1	1	1	1
Lung Adenocarcinoma	TCGA-44-6148	0	0	0	0	0
Lung Adenocarcinoma	TCGA-44-6774	1	1	1	1	1
Lung Adenocarcinoma	TCGA-44-6775	1	1	1	1	1
Lung Adenocarcinoma	TCGA-44-6776	1	0	1	1	1
Lung Adenocarcinoma	TCGA-44-6777	1	1	1	1	1
Lung Adenocarcinoma	TCGA-44-6778	1	1	1	1	1
Lung Adenocarcinoma	TCGA-44-6779	1	1	1	1	1
Lung Adenocarcinoma	TCGA-44-7659	1	1	1	0	0
Lung Adenocarcinoma	TCGA-44-7660	1	1	1	1	1
Lung Adenocarcinoma	TCGA-44-7661	1	1	1	1	1
Lung Adenocarcinoma	TCGA-44-7662	1	1	1	1	1
Lung Adenocarcinoma	TCGA-44-7667	1	1	1	1	1
Lung Adenocarcinoma	TCGA-44-7669	1	1	1	0	1
Lung Adenocarcinoma	TCGA-44-7670	1	1	1	1	1
Lung Adenocarcinoma	TCGA-44-7671	1	1	1	1	1
Lung Adenocarcinoma	TCGA-44-7672	1	1	0	1	1
Lung Adenocarcinoma	TCGA-44-8117	1	1	1	1	1
Lung Adenocarcinoma	TCGA-44-8119	1	1	1	1	1
Lung Adenocarcinoma	TCGA-44-8120	1	1	1	1	1
Lung Adenocarcinoma	TCGA-44-A479	1	1	1	1	1
Lung Adenocarcinoma	TCGA-44-A47A	0	0	0	0	0
Lung Adenocarcinoma	TCGA-44-A47B	1	1	1	1	1
Lung Adenocarcinoma	TCGA-44-A47G	1	0	0	0	0
Lung Adenocarcinoma	TCGA-44-A4SS	1	1	1	1	1
Lung Adenocarcinoma	TCGA-44-A4SU	1	1	1	1	1
Lung Adenocarcinoma	TCGA-49-4486	1	1	1	1	1
Lung Adenocarcinoma	TCGA-49-4487	1	1	1	1	1
Lung Adenocarcinoma	TCGA-49-4488	1	1	1	1	1
Lung Adenocarcinoma	TCGA-49-4490	1	1	1	1	1
Lung Adenocarcinoma	TCGA-49-4494	1	1	1	1	1
Lung Adenocarcinoma	TCGA-49-4501	1	1	1	1	1

Lung Adenocarcinoma	TCGA-49-4505	1	1	1	1	1
Lung Adenocarcinoma	TCGA-49-4506	1	1	0	1	1
Lung Adenocarcinoma	TCGA-49-4507	1	1	1	1	1
Lung Adenocarcinoma	TCGA-49-4510	1	1	1	1	1
Lung Adenocarcinoma	TCGA-49-4512	1	1	1	1	1
Lung Adenocarcinoma	TCGA-49-4514	1	1	1	1	1
Lung Adenocarcinoma	TCGA-49-6742	1	1	1	1	1
Lung Adenocarcinoma	TCGA-49-6743	1	1	1	1	1
Lung Adenocarcinoma	TCGA-49-6744	1	1	1	1	1
Lung Adenocarcinoma	TCGA-49-6745	1	1	1	1	1
Lung Adenocarcinoma	TCGA-49-6761	1	1	1	1	1
Lung Adenocarcinoma	TCGA-49-6767	1	1	1	1	1
Lung Adenocarcinoma	TCGA-49-AAQV	1	1	1	1	1
Lung Adenocarcinoma	TCGA-49-AAR0	1	1	1	1	1
Lung Adenocarcinoma	TCGA-49-AAR2	1	1	1	1	1
Lung Adenocarcinoma	TCGA-49-AAR3	1	1	0	0	1
Lung Adenocarcinoma	TCGA-49-AAR4	1	1	1	1	1
Lung Adenocarcinoma	TCGA-49-AAR9	1	1	1	1	1
Lung Adenocarcinoma	TCGA-49-AARE	1	1	1	1	1
Lung Adenocarcinoma	TCGA-49-AARN	1	1	1	1	1
Lung Adenocarcinoma	TCGA-49-AARO	1	1	1	1	1
Lung Adenocarcinoma	TCGA-49-AARQ	1	1	1	1	1
Lung Adenocarcinoma	TCGA-49-AARR	0	0	0	0	0
Lung Adenocarcinoma	TCGA-4B-A93V	0	0	0	0	1
Lung Adenocarcinoma	TCGA-50-5044	1	0	0	1	1
Lung Adenocarcinoma	TCGA-50-5045	1	1	1	1	1
Lung Adenocarcinoma	TCGA-50-5049	1	1	1	1	1
Lung Adenocarcinoma	TCGA-50-5051	1	1	1	1	1
Lung Adenocarcinoma	TCGA-50-5055	1	1	0	0	1
Lung Adenocarcinoma	TCGA-50-5066	1	1	1	1	1
Lung Adenocarcinoma	TCGA-50-5066	0	0	0	0	0
Lung Adenocarcinoma	TCGA-50-5068	1	1	1	1	1
Lung Adenocarcinoma	TCGA-50-5072	1	1	1	1	1
Lung Adenocarcinoma	TCGA-50-5930	1	1	1	1	1
Lung Adenocarcinoma	TCGA-50-5931	1	1	1	1	1
Lung Adenocarcinoma	TCGA-50-5932	1	1	1	1	1
Lung Adenocarcinoma	TCGA-50-5933	1	1	1	1	1
Lung Adenocarcinoma	TCGA-50-5935	1	1	1	1	1
Lung Adenocarcinoma	TCGA-50-5936	1	1	1	1	1
Lung Adenocarcinoma	TCGA-50-5939	1	1	1	1	1
Lung Adenocarcinoma	TCGA-50-5941	1	1	1	1	1
Lung Adenocarcinoma	TCGA-50-5942	1	0	1	1	0
Lung Adenocarcinoma	TCGA-50-5944	1	1	1	1	1
Lung Adenocarcinoma	TCGA-50-5946	1	1	1	1	1
Lung Adenocarcinoma	TCGA-50-5946	0	0	0	0	0
Lung Adenocarcinoma	TCGA-50-6590	1	0	1	1	1
Lung Adenocarcinoma	TCGA-50-6591	1	1	1	1	1
Lung Adenocarcinoma	TCGA-50-6592	1	1	1	1	1
Lung Adenocarcinoma	TCGA-50-6593	1	1	1	1	1
Lung Adenocarcinoma	TCGA-50-6594	1	1	1	1	1
Lung Adenocarcinoma	TCGA-50-6595	1	1	0	1	1
Lung Adenocarcinoma	TCGA-50-6597	1	1	1	1	1
Lung Adenocarcinoma	TCGA-50-6673	1	1	1	1	1
Lung Adenocarcinoma	TCGA-50-7109	1	1	1	1	1
Lung Adenocarcinoma	TCGA-50-8457	1	0	1	1	1
Lung Adenocarcinoma	TCGA-50-8459	1	1	0	0	1
Lung Adenocarcinoma	TCGA-50-8460	1	1	0	1	1
Lung Adenocarcinoma	TCGA-53-7624	1	1	1	1	1

Lung Adenocarcinoma	TCGA-53-7626	1	1	1	1	1
Lung Adenocarcinoma	TCGA-53-7813	1	1	1	1	1
Lung Adenocarcinoma	TCGA-53-A4EZ	1	1	1	1	1
Lung Adenocarcinoma	TCGA-55-1592	1	1	1	1	1
Lung Adenocarcinoma	TCGA-55-1594	1	1	1	1	1
Lung Adenocarcinoma	TCGA-55-1595	1	1	1	1	1
Lung Adenocarcinoma	TCGA-55-1596	1	1	1	1	1
Lung Adenocarcinoma	TCGA-55-5899	1	1	1	1	1
Lung Adenocarcinoma	TCGA-55-6543	0	0	1	0	1
Lung Adenocarcinoma	TCGA-55-6642	1	1	1	1	1
Lung Adenocarcinoma	TCGA-55-6712	1	1	1	1	1
Lung Adenocarcinoma	TCGA-55-6968	1	1	1	1	1
Lung Adenocarcinoma	TCGA-55-6969	1	1	1	1	1
Lung Adenocarcinoma	TCGA-55-6970	0	0	1	0	0
Lung Adenocarcinoma	TCGA-55-6971	1	1	1	1	1
Lung Adenocarcinoma	TCGA-55-6972	1	1	1	1	1
Lung Adenocarcinoma	TCGA-55-6975	1	1	1	1	1
Lung Adenocarcinoma	TCGA-55-6978	1	1	1	1	1
Lung Adenocarcinoma	TCGA-55-6979	1	1	1	1	1
Lung Adenocarcinoma	TCGA-55-6980	1	1	1	1	1
Lung Adenocarcinoma	TCGA-55-6981	1	1	1	1	1
Lung Adenocarcinoma	TCGA-55-6982	1	1	1	1	1
Lung Adenocarcinoma	TCGA-55-6983	1	0	1	1	1
Lung Adenocarcinoma	TCGA-55-6984	0	0	1	0	1
Lung Adenocarcinoma	TCGA-55-6985	1	1	1	1	1
Lung Adenocarcinoma	TCGA-55-6986	1	1	1	1	1
Lung Adenocarcinoma	TCGA-55-6987	1	1	0	1	1
Lung Adenocarcinoma	TCGA-55-7227	0	0	0	0	0
Lung Adenocarcinoma	TCGA-55-7281	1	1	1	1	1
Lung Adenocarcinoma	TCGA-55-7283	1	1	1	1	1
Lung Adenocarcinoma	TCGA-55-7284	1	0	0	0	1
Lung Adenocarcinoma	TCGA-55-7570	1	1	1	1	1
Lung Adenocarcinoma	TCGA-55-7573	1	1	1	1	1
Lung Adenocarcinoma	TCGA-55-7574	1	0	1	1	1
Lung Adenocarcinoma	TCGA-55-7576	1	1	1	1	1
Lung Adenocarcinoma	TCGA-55-7724	1	1	1	1	1
Lung Adenocarcinoma	TCGA-55-7725	1	0	1	0	0
Lung Adenocarcinoma	TCGA-55-7726	1	1	1	1	1
Lung Adenocarcinoma	TCGA-55-7727	1	1	1	1	1
Lung Adenocarcinoma	TCGA-55-7728	1	0	1	0	1
Lung Adenocarcinoma	TCGA-55-7815	1	0	1	1	1
Lung Adenocarcinoma	TCGA-55-7816	0	0	0	0	0
Lung Adenocarcinoma	TCGA-55-7903	1	1	1	1	1
Lung Adenocarcinoma	TCGA-55-7907	1	1	1	1	1
Lung Adenocarcinoma	TCGA-55-7910	1	0	1	1	1
Lung Adenocarcinoma	TCGA-55-7911	1	1	1	1	1
Lung Adenocarcinoma	TCGA-55-7913	1	1	1	1	1
Lung Adenocarcinoma	TCGA-55-7914	1	1	1	1	1
Lung Adenocarcinoma	TCGA-55-7994	1	1	1	1	1
Lung Adenocarcinoma	TCGA-55-7995	1	1	1	1	1
Lung Adenocarcinoma	TCGA-55-8085	1	1	1	1	1
Lung Adenocarcinoma	TCGA-55-8087	1	0	1	0	0
Lung Adenocarcinoma	TCGA-55-8089	1	1	1	0	1
Lung Adenocarcinoma	TCGA-55-8090	1	1	1	1	1
Lung Adenocarcinoma	TCGA-55-8091	1	1	1	1	1
Lung Adenocarcinoma	TCGA-55-8092	1	1	1	1	1
Lung Adenocarcinoma	TCGA-55-8094	1	1	1	1	1
Lung Adenocarcinoma	TCGA-55-8096	1	0	0	1	0

Lung Adenocarcinoma	TCGA-55-8097	1	1	1	0	1
Lung Adenocarcinoma	TCGA-55-8203	1	1	1	1	1
Lung Adenocarcinoma	TCGA-55-8204	1	1	1	1	1
Lung Adenocarcinoma	TCGA-55-8205	1	1	1	1	1
Lung Adenocarcinoma	TCGA-55-8206	1	1	0	1	1
Lung Adenocarcinoma	TCGA-55-8207	1	1	1	1	1
Lung Adenocarcinoma	TCGA-55-8208	0	1	0	0	0
Lung Adenocarcinoma	TCGA-55-8299	0	0	0	0	0
Lung Adenocarcinoma	TCGA-55-8301	1	1	0	1	1
Lung Adenocarcinoma	TCGA-55-8302	1	1	1	0	1
Lung Adenocarcinoma	TCGA-55-8505	1	1	1	1	1
Lung Adenocarcinoma	TCGA-55-8506	1	1	1	1	1
Lung Adenocarcinoma	TCGA-55-8507	1	1	1	1	1
Lung Adenocarcinoma	TCGA-55-8508	1	1	1	1	1
Lung Adenocarcinoma	TCGA-55-8510	1	1	1	1	1
Lung Adenocarcinoma	TCGA-55-8511	1	1	1	1	1
Lung Adenocarcinoma	TCGA-55-8512	1	1	1	1	1
Lung Adenocarcinoma	TCGA-55-8513	0	0	0	0	0
Lung Adenocarcinoma	TCGA-55-8514	1	1	1	1	1
Lung Adenocarcinoma	TCGA-55-8614	1	1	1	1	1
Lung Adenocarcinoma	TCGA-55-8615	1	1	1	1	1
Lung Adenocarcinoma	TCGA-55-8616	1	1	1	1	1
Lung Adenocarcinoma	TCGA-55-8619	1	0	1	0	0
Lung Adenocarcinoma	TCGA-55-8620	1	0	1	1	1
Lung Adenocarcinoma	TCGA-55-8621	1	0	0	1	0
Lung Adenocarcinoma	TCGA-55-A48X	1	1	1	1	1
Lung Adenocarcinoma	TCGA-55-A48Y	1	1	1	1	1
Lung Adenocarcinoma	TCGA-55-A48Z	1	1	1	1	1
Lung Adenocarcinoma	TCGA-55-A490	1	1	1	1	1
Lung Adenocarcinoma	TCGA-55-A491	1	1	1	1	1
Lung Adenocarcinoma	TCGA-55-A492	1	1	1	1	1
Lung Adenocarcinoma	TCGA-55-A493	1	1	1	1	1
Lung Adenocarcinoma	TCGA-55-A494	1	1	1	1	0
Lung Adenocarcinoma	TCGA-55-A4DF	1	1	1	1	1
Lung Adenocarcinoma	TCGA-55-A4DG	1	1	1	1	1
Lung Adenocarcinoma	TCGA-55-A57B	1	1	1	0	1
Lung Adenocarcinoma	TCGA-62-8394	1	1	1	1	1
Lung Adenocarcinoma	TCGA-62-8395	1	1	1	0	1
Lung Adenocarcinoma	TCGA-62-8397	1	1	1	1	1
Lung Adenocarcinoma	TCGA-62-8398	1	1	1	1	1
Lung Adenocarcinoma	TCGA-62-8399	1	1	1	1	1
Lung Adenocarcinoma	TCGA-62-8402	1	1	1	1	1
Lung Adenocarcinoma	TCGA-62-A46O	1	1	1	1	1
Lung Adenocarcinoma	TCGA-62-A46P	1	0	1	1	1
Lung Adenocarcinoma	TCGA-62-A46R	1	1	1	1	1
Lung Adenocarcinoma	TCGA-62-A46S	1	1	1	1	1
Lung Adenocarcinoma	TCGA-62-A46U	1	1	1	1	1
Lung Adenocarcinoma	TCGA-62-A46V	1	1	1	1	1
Lung Adenocarcinoma	TCGA-62-A46Y	1	1	1	1	1
Lung Adenocarcinoma	TCGA-62-A470	1	1	1	1	1
Lung Adenocarcinoma	TCGA-62-A471	1	1	1	1	1
Lung Adenocarcinoma	TCGA-62-A472	1	1	1	1	1
Lung Adenocarcinoma	TCGA-64-1676	1	1	1	1	1
Lung Adenocarcinoma	TCGA-64-1677	1	1	1	1	1
Lung Adenocarcinoma	TCGA-64-1678	1	1	1	1	1
Lung Adenocarcinoma	TCGA-64-1679	1	1	1	1	1
Lung Adenocarcinoma	TCGA-64-1680	1	1	0	0	1
Lung Adenocarcinoma	TCGA-64-1681	1	1	1	1	1

Lung Adenocarcinoma	TCGA-64-5774	1	1	1	1	1
Lung Adenocarcinoma	TCGA-64-5775	1	1	1	1	1
Lung Adenocarcinoma	TCGA-64-5778	1	1	1	1	1
Lung Adenocarcinoma	TCGA-64-5779	1	1	1	1	1
Lung Adenocarcinoma	TCGA-64-5781	1	1	1	1	1
Lung Adenocarcinoma	TCGA-64-5815	1	1	1	1	1
Lung Adenocarcinoma	TCGA-67-3770	1	1	1	1	1
Lung Adenocarcinoma	TCGA-67-3771	1	1	1	1	1
Lung Adenocarcinoma	TCGA-67-3772	1	1	1	1	1
Lung Adenocarcinoma	TCGA-67-3773	1	0	1	0	1
Lung Adenocarcinoma	TCGA-67-3774	1	1	1	1	1
Lung Adenocarcinoma	TCGA-67-4679	1	0	1	0	0
Lung Adenocarcinoma	TCGA-67-6215	1	0	1	0	1
Lung Adenocarcinoma	TCGA-67-6216	1	1	1	0	1
Lung Adenocarcinoma	TCGA-67-6217	1	1	1	1	1
Lung Adenocarcinoma	TCGA-69-7760	1	1	1	1	1
Lung Adenocarcinoma	TCGA-69-7761	1	1	1	1	1
Lung Adenocarcinoma	TCGA-69-7763	1	1	1	1	1
Lung Adenocarcinoma	TCGA-69-7764	1	1	1	1	1
Lung Adenocarcinoma	TCGA-69-7765	1	1	1	1	1
Lung Adenocarcinoma	TCGA-69-7973	1	1	1	1	1
Lung Adenocarcinoma	TCGA-69-7974	1	1	1	1	1
Lung Adenocarcinoma	TCGA-69-7978	1	1	1	1	1
Lung Adenocarcinoma	TCGA-69-7979	1	1	1	1	1
Lung Adenocarcinoma	TCGA-69-7980	1	1	1	1	1
Lung Adenocarcinoma	TCGA-69-8253	1	0	1	1	1
Lung Adenocarcinoma	TCGA-69-8254	1	0	1	0	0
Lung Adenocarcinoma	TCGA-69-8255	1	1	1	1	1
Lung Adenocarcinoma	TCGA-69-8453	1	1	1	1	1
Lung Adenocarcinoma	TCGA-69-A59K	1	1	1	1	1
Lung Adenocarcinoma	TCGA-71-6725	1	1	1	1	1
Lung Adenocarcinoma	TCGA-71-8520	0	0	0	0	0
Lung Adenocarcinoma	TCGA-73-4658	1	0	1	0	1
Lung Adenocarcinoma	TCGA-73-4659	1	1	1	1	1
Lung Adenocarcinoma	TCGA-73-4662	1	1	1	1	1
Lung Adenocarcinoma	TCGA-73-4666	1	1	1	1	1
Lung Adenocarcinoma	TCGA-73-4668	1	1	1	1	1
Lung Adenocarcinoma	TCGA-73-4670	1	1	1	1	1
Lung Adenocarcinoma	TCGA-73-4675	1	1	1	1	1
Lung Adenocarcinoma	TCGA-73-4676	0	1	0	0	1
Lung Adenocarcinoma	TCGA-73-4677	1	1	1	1	1
Lung Adenocarcinoma	TCGA-73-7498	1	1	1	1	1
Lung Adenocarcinoma	TCGA-73-7499	1	1	1	1	1
Lung Adenocarcinoma	TCGA-73-A9RS	1	1	1	1	1
Lung Adenocarcinoma	TCGA-75-5122	1	1	0	1	1
Lung Adenocarcinoma	TCGA-75-5125	1	1	1	1	1
Lung Adenocarcinoma	TCGA-75-5126	0	1	0	1	1
Lung Adenocarcinoma	TCGA-75-5146	1	1	1	1	1
Lung Adenocarcinoma	TCGA-75-5147	1	1	1	1	1
Lung Adenocarcinoma	TCGA-75-6203	0	0	0	0	0
Lung Adenocarcinoma	TCGA-75-6205	1	1	1	1	1
Lung Adenocarcinoma	TCGA-75-6206	1	0	1	1	1
Lung Adenocarcinoma	TCGA-75-6207	1	1	1	1	1
Lung Adenocarcinoma	TCGA-75-6211	1	1	1	1	1
Lung Adenocarcinoma	TCGA-75-6212	1	1	1	1	1
Lung Adenocarcinoma	TCGA-75-6214	1	1	1	1	1
Lung Adenocarcinoma	TCGA-75-7025	1	1	1	1	1
Lung Adenocarcinoma	TCGA-75-7027	1	1	1	1	1

Lung Adenocarcinoma	TCGA-75-7030	0	0	0	0	0
Lung Adenocarcinoma	TCGA-75-7031	1	1	1	1	1
Lung Adenocarcinoma	TCGA-78-7143	1	1	1	1	1
Lung Adenocarcinoma	TCGA-78-7145	1	1	1	1	1
Lung Adenocarcinoma	TCGA-78-7146	1	1	1	1	1
Lung Adenocarcinoma	TCGA-78-7147	1	1	1	1	1
Lung Adenocarcinoma	TCGA-78-7148	1	1	1	1	1
Lung Adenocarcinoma	TCGA-78-7149	1	1	1	1	1
Lung Adenocarcinoma	TCGA-78-7150	1	1	1	1	1
Lung Adenocarcinoma	TCGA-78-7152	1	1	1	1	1
Lung Adenocarcinoma	TCGA-78-7153	1	1	1	1	1
Lung Adenocarcinoma	TCGA-78-7154	1	1	1	1	1
Lung Adenocarcinoma	TCGA-78-7155	1	1	1	1	1
Lung Adenocarcinoma	TCGA-78-7156	1	1	1	1	1
Lung Adenocarcinoma	TCGA-78-7158	1	0	1	1	1
Lung Adenocarcinoma	TCGA-78-7159	1	1	1	1	1
Lung Adenocarcinoma	TCGA-78-7160	1	1	1	1	1
Lung Adenocarcinoma	TCGA-78-7161	1	1	1	1	1
Lung Adenocarcinoma	TCGA-78-7162	1	1	1	0	1
Lung Adenocarcinoma	TCGA-78-7163	0	1	0	1	1
Lung Adenocarcinoma	TCGA-78-7166	1	1	1	0	1
Lung Adenocarcinoma	TCGA-78-7167	1	1	1	1	1
Lung Adenocarcinoma	TCGA-78-7220	1	1	1	1	1
Lung Adenocarcinoma	TCGA-78-7535	1	1	1	1	1
Lung Adenocarcinoma	TCGA-78-7536	1	1	1	1	1
Lung Adenocarcinoma	TCGA-78-7537	1	0	1	1	0
Lung Adenocarcinoma	TCGA-78-7539	1	1	1	1	1
Lung Adenocarcinoma	TCGA-78-7540	1	1	1	0	1
Lung Adenocarcinoma	TCGA-78-7542	1	1	1	1	1
Lung Adenocarcinoma	TCGA-78-7633	1	1	1	1	1
Lung Adenocarcinoma	TCGA-78-8640	1	1	1	1	1
Lung Adenocarcinoma	TCGA-78-8648	0	0	0	0	0
Lung Adenocarcinoma	TCGA-78-8655	1	1	1	1	1
Lung Adenocarcinoma	TCGA-78-8660	1	1	1	1	1
Lung Adenocarcinoma	TCGA-78-8662	0	1	0	0	0
Lung Adenocarcinoma	TCGA-80-5607	1	0	1	1	1
Lung Adenocarcinoma	TCGA-80-5608	1	1	1	1	1
Lung Adenocarcinoma	TCGA-80-5611	1	1	1	1	1
Lung Adenocarcinoma	TCGA-83-5908	1	1	1	1	1
Lung Adenocarcinoma	TCGA-86-6562	1	1	1	1	1
Lung Adenocarcinoma	TCGA-86-6851	1	1	1	1	1
Lung Adenocarcinoma	TCGA-86-7701	1	1	1	1	1
Lung Adenocarcinoma	TCGA-86-7711	1	1	1	1	1
Lung Adenocarcinoma	TCGA-86-7713	1	1	1	1	1
Lung Adenocarcinoma	TCGA-86-7714	1	0	1	1	0
Lung Adenocarcinoma	TCGA-86-7953	1	1	1	1	1
Lung Adenocarcinoma	TCGA-86-7954	1	1	1	1	1
Lung Adenocarcinoma	TCGA-86-7955	1	1	1	1	1
Lung Adenocarcinoma	TCGA-86-8054	1	1	1	1	1
Lung Adenocarcinoma	TCGA-86-8055	1	1	1	1	1
Lung Adenocarcinoma	TCGA-86-8056	1	1	1	1	1
Lung Adenocarcinoma	TCGA-86-8073	1	1	1	1	1
Lung Adenocarcinoma	TCGA-86-8074	1	1	1	1	1
Lung Adenocarcinoma	TCGA-86-8075	1	1	1	1	1
Lung Adenocarcinoma	TCGA-86-8076	0	0	0	0	0
Lung Adenocarcinoma	TCGA-86-8278	1	0	1	1	1
Lung Adenocarcinoma	TCGA-86-8279	1	1	1	1	1
Lung Adenocarcinoma	TCGA-86-8280	1	1	1	1	1

Lung Adenocarcinoma	TCGA-86-8281	0	0	1	0	0
Lung Adenocarcinoma	TCGA-86-8358	1	1	1	1	1
Lung Adenocarcinoma	TCGA-86-8359	1	1	1	1	1
Lung Adenocarcinoma	TCGA-86-8585	1	1	1	1	1
Lung Adenocarcinoma	TCGA-86-8668	1	1	1	1	1
Lung Adenocarcinoma	TCGA-86-8669	1	1	1	1	1
Lung Adenocarcinoma	TCGA-86-8671	0	0	0	1	1
Lung Adenocarcinoma	TCGA-86-8672	1	1	1	1	1
Lung Adenocarcinoma	TCGA-86-8673	0	0	0	1	1
Lung Adenocarcinoma	TCGA-86-8674	0	0	1	0	0
Lung Adenocarcinoma	TCGA-86-A456	1	1	1	1	1
Lung Adenocarcinoma	TCGA-86-A4D0	1	1	1	1	1
Lung Adenocarcinoma	TCGA-86-A4JF	1	1	1	1	1
Lung Adenocarcinoma	TCGA-86-A4P7	0	1	0	0	0
Lung Adenocarcinoma	TCGA-86-A4P8	0	0	0	0	0
Lung Adenocarcinoma	TCGA-91-6828	1	1	1	1	1
Lung Adenocarcinoma	TCGA-91-6829	1	1	1	1	1
Lung Adenocarcinoma	TCGA-91-6830	1	1	1	1	1
Lung Adenocarcinoma	TCGA-91-6831	1	1	1	1	1
Lung Adenocarcinoma	TCGA-91-6835	1	1	1	1	1
Lung Adenocarcinoma	TCGA-91-6836	1	1	1	1	1
Lung Adenocarcinoma	TCGA-91-6840	1	1	1	1	1
Lung Adenocarcinoma	TCGA-91-6847	1	1	1	1	1
Lung Adenocarcinoma	TCGA-91-6848	1	1	1	1	1
Lung Adenocarcinoma	TCGA-91-6849	1	1	1	0	1
Lung Adenocarcinoma	TCGA-91-7771	1	1	1	1	1
Lung Adenocarcinoma	TCGA-91-8496	0	0	0	0	0
Lung Adenocarcinoma	TCGA-91-8497	1	1	1	1	1
Lung Adenocarcinoma	TCGA-91-8499	1	1	1	1	1
Lung Adenocarcinoma	TCGA-91-A4BC	1	1	1	1	1
Lung Adenocarcinoma	TCGA-91-A4BD	1	1	1	0	0
Lung Adenocarcinoma	TCGA-93-7347	1	1	1	1	1
Lung Adenocarcinoma	TCGA-93-7348	1	1	1	1	1
Lung Adenocarcinoma	TCGA-93-8067	1	1	1	1	1
Lung Adenocarcinoma	TCGA-93-A4JN	0	1	0	1	1
Lung Adenocarcinoma	TCGA-93-A4JO	1	1	1	1	1
Lung Adenocarcinoma	TCGA-93-A4JP	1	0	1	0	1
Lung Adenocarcinoma	TCGA-93-A4JQ	1	1	1	1	1
Lung Adenocarcinoma	TCGA-95-7039	1	1	1	1	1
Lung Adenocarcinoma	TCGA-95-7043	1	1	1	1	1
Lung Adenocarcinoma	TCGA-95-7562	1	1	1	1	1
Lung Adenocarcinoma	TCGA-95-7567	1	1	1	1	1
Lung Adenocarcinoma	TCGA-95-7944	1	1	1	1	1
Lung Adenocarcinoma	TCGA-95-7947	1	0	1	1	1
Lung Adenocarcinoma	TCGA-95-7948	1	1	1	1	1
Lung Adenocarcinoma	TCGA-95-8039	1	1	1	1	1
Lung Adenocarcinoma	TCGA-95-8494	1	1	1	1	1
Lung Adenocarcinoma	TCGA-95-A4VK	1	1	1	0	1
Lung Adenocarcinoma	TCGA-95-A4VN	1	1	1	1	1
Lung Adenocarcinoma	TCGA-95-A4VP	1	1	1	1	1
Lung Adenocarcinoma	TCGA-97-7546	1	1	1	1	1
Lung Adenocarcinoma	TCGA-97-7547	1	0	1	0	1
Lung Adenocarcinoma	TCGA-97-7552	1	0	0	1	1
Lung Adenocarcinoma	TCGA-97-7553	0	0	0	0	1
Lung Adenocarcinoma	TCGA-97-7554	1	1	1	1	1
Lung Adenocarcinoma	TCGA-97-7937	1	1	1	1	1
Lung Adenocarcinoma	TCGA-97-7938	1	1	1	1	1
Lung Adenocarcinoma	TCGA-97-7941	1	0	0	0	0

Lung Adenocarcinoma	TCGA-97-8171	1	1	1	0	1
Lung Adenocarcinoma	TCGA-97-8172	1	1	1	1	1
Lung Adenocarcinoma	TCGA-97-8174	1	1	1	1	1
Lung Adenocarcinoma	TCGA-97-8175	1	1	1	1	1
Lung Adenocarcinoma	TCGA-97-8176	1	1	1	1	1
Lung Adenocarcinoma	TCGA-97-8177	1	1	1	1	1
Lung Adenocarcinoma	TCGA-97-8179	0	1	0	0	1
Lung Adenocarcinoma	TCGA-97-8547	1	1	1	1	1
Lung Adenocarcinoma	TCGA-97-8552	1	1	0	1	1
Lung Adenocarcinoma	TCGA-97-A4LX	1	1	1	1	1
Lung Adenocarcinoma	TCGA-97-A4M0	1	1	1	1	1
Lung Adenocarcinoma	TCGA-97-A4M1	1	1	1	1	1
Lung Adenocarcinoma	TCGA-97-A4M2	0	0	1	0	0
Lung Adenocarcinoma	TCGA-97-A4M3	1	1	1	1	1
Lung Adenocarcinoma	TCGA-97-A4M5	1	1	1	1	1
Lung Adenocarcinoma	TCGA-97-A4M6	1	1	1	1	1
Lung Adenocarcinoma	TCGA-97-A4M7	1	1	1	1	1
Lung Adenocarcinoma	TCGA-99-7458	1	1	1	1	1
Lung Adenocarcinoma	TCGA-99-8025	1	1	1	1	1
Lung Adenocarcinoma	TCGA-99-8028	0	0	0	1	1
Lung Adenocarcinoma	TCGA-99-8032	1	1	0	1	1
Lung Adenocarcinoma	TCGA-99-8033	1	1	1	1	1
Lung Adenocarcinoma	TCGA-99-AA5R	0	0	0	0	0
Lung Adenocarcinoma	TCGA-J2-8192	1	1	1	1	1
Lung Adenocarcinoma	TCGA-J2-8194	1	1	1	0	0
Lung Adenocarcinoma	TCGA-J2-A4AD	1	1	1	1	1
Lung Adenocarcinoma	TCGA-J2-A4AE	1	0	1	0	1
Lung Adenocarcinoma	TCGA-J2-A4AG	1	1	0	1	1
Lung Adenocarcinoma	TCGA-L4-A4E5	1	1	1	1	1
Lung Adenocarcinoma	TCGA-L4-A4E6	0	0	0	0	0
Lung Adenocarcinoma	TCGA-L9-A443	1	0	1	1	1
Lung Adenocarcinoma	TCGA-L9-A444	1	1	1	1	1
Lung Adenocarcinoma	TCGA-L9-A50W	1	1	1	1	1
Lung Adenocarcinoma	TCGA-L9-A5IP	1	1	1	0	0
Lung Adenocarcinoma	TCGA-L9-A743	1	1	1	1	1
Lung Adenocarcinoma	TCGA-L9-A7SV	1	1	1	1	1
Lung Adenocarcinoma	TCGA-L9-A8F4	1	1	1	1	1
Lung Adenocarcinoma	TCGA-MN-A4N1	1	1	1	1	1
Lung Adenocarcinoma	TCGA-MN-A4N4	1	1	1	1	1
Lung Adenocarcinoma	TCGA-MN-A4N5	1	1	1	1	1
Lung Adenocarcinoma	TCGA-MP-A4SV	1	1	1	1	1
Lung Adenocarcinoma	TCGA-MP-A4SW	1	1	1	1	1
Lung Adenocarcinoma	TCGA-MP-A4SY	1	1	1	1	1
Lung Adenocarcinoma	TCGA-MP-A4T4	1	1	1	1	1
Lung Adenocarcinoma	TCGA-MP-A4T6	1	1	1	1	1
Lung Adenocarcinoma	TCGA-MP-A4T7	1	1	1	1	1
Lung Adenocarcinoma	TCGA-MP-A4T8	1	1	1	1	1
Lung Adenocarcinoma	TCGA-MP-A4T9	1	1	0	0	1
Lung Adenocarcinoma	TCGA-MP-A4TA	1	1	1	1	1
Lung Adenocarcinoma	TCGA-MP-A4TC	1	1	1	1	1
Lung Adenocarcinoma	TCGA-MP-A4TD	1	1	1	1	1
Lung Adenocarcinoma	TCGA-MP-A4TE	1	1	1	1	1
Lung Adenocarcinoma	TCGA-MP-A4TF	1	1	1	1	1
Lung Adenocarcinoma	TCGA-MP-A4TH	1	1	1	1	0
Lung Adenocarcinoma	TCGA-MP-A4TI	1	1	1	1	1
Lung Adenocarcinoma	TCGA-MP-A4TJ	1	1	1	1	1
Lung Adenocarcinoma	TCGA-MP-A4TK	1	1	1	1	1
Lung Adenocarcinoma	TCGA-MP-A5C7	1	1	1	1	1

Lung Adenocarcinoma	TCGA-NJ-A4YF	1	1	1	1	1
Lung Adenocarcinoma	TCGA-NJ-A4YG	1	0	1	1	1
Lung Adenocarcinoma	TCGA-NJ-A4YI	1	1	1	1	1
Lung Adenocarcinoma	TCGA-NJ-A4YP	0	0	1	0	0
Lung Adenocarcinoma	TCGA-NJ-A4YQ	1	1	1	1	1
Lung Adenocarcinoma	TCGA-NJ-A55A	0	0	1	0	0
Lung Adenocarcinoma	TCGA-NJ-A55O	1	0	1	1	1
Lung Adenocarcinoma	TCGA-NJ-A55R	1	1	1	1	1
Lung Adenocarcinoma	TCGA-NJ-A7XG	1	1	1	1	1
Lung Adenocarcinoma	TCGA-O1-A52J	1	1	1	1	1
Lung Adenocarcinoma	TCGA-S2-AA1A	0	0	0	0	0
.ung Squamous Cell Carcinom	TCGA-18-3406	1	1	1	1	1
.ung Squamous Cell Carcinom	TCGA-18-3407	1	0	1	1	1
.ung Squamous Cell Carcinom	TCGA-18-3408	1	1	1	1	1
.ung Squamous Cell Carcinom	TCGA-18-3409	1	1	1	1	1
.ung Squamous Cell Carcinom	TCGA-18-3410	1	1	1	1	1
.ung Squamous Cell Carcinom	TCGA-18-3411	1	1	1	1	1
.ung Squamous Cell Carcinom	TCGA-18-3412	1	1	1	1	1
.ung Squamous Cell Carcinom	TCGA-18-3414	1	1	1	1	1
.ung Squamous Cell Carcinom	TCGA-18-3415	1	1	1	1	1
.ung Squamous Cell Carcinom	TCGA-18-3416	1	1	1	1	1
.ung Squamous Cell Carcinom	TCGA-18-3417	1	1	1	1	1
.ung Squamous Cell Carcinom	TCGA-18-3419	1	1	1	0	1
.ung Squamous Cell Carcinom	TCGA-18-3421	1	1	1	1	1
.ung Squamous Cell Carcinom	TCGA-18-4083	1	1	1	1	1
.ung Squamous Cell Carcinom	TCGA-18-4086	1	1	1	1	1
.ung Squamous Cell Carcinom	TCGA-18-4721	1	1	1	1	1
.ung Squamous Cell Carcinom	TCGA-18-5592	1	1	1	1	1
.ung Squamous Cell Carcinom	TCGA-18-5595	1	1	0	1	1
.ung Squamous Cell Carcinom	TCGA-21-1070	1	1	1	1	1
.ung Squamous Cell Carcinom	TCGA-21-1071	1	1	1	1	1
.ung Squamous Cell Carcinom	TCGA-21-1072	1	1	1	1	1
.ung Squamous Cell Carcinom	TCGA-21-1075	1	1	1	1	1
.ung Squamous Cell Carcinom	TCGA-21-1076	1	1	1	1	1
.ung Squamous Cell Carcinom	TCGA-21-1077	1	1	1	1	1
.ung Squamous Cell Carcinom	TCGA-21-1078	1	0	0	0	0
.ung Squamous Cell Carcinom	TCGA-21-1079	1	1	1	1	1
.ung Squamous Cell Carcinom	TCGA-21-1080	1	1	1	1	1
.ung Squamous Cell Carcinom	TCGA-21-1081	1	0	1	0	0
.ung Squamous Cell Carcinom	TCGA-21-1082	1	1	1	1	1
.ung Squamous Cell Carcinom	TCGA-21-1083	1	1	1	1	1
.ung Squamous Cell Carcinom	TCGA-21-5782	1	1	1	1	1
.ung Squamous Cell Carcinom	TCGA-21-5783	1	1	1	1	1
.ung Squamous Cell Carcinom	TCGA-21-5784	1	1	1	0	1
.ung Squamous Cell Carcinom	TCGA-21-5786	1	1	1	1	1
.ung Squamous Cell Carcinom	TCGA-21-5787	1	1	1	1	1
.ung Squamous Cell Carcinom	TCGA-21-A5DI	1	1	1	1	1
.ung Squamous Cell Carcinom	TCGA-22-0940	1	1	1	1	1
.ung Squamous Cell Carcinom	TCGA-22-0944	1	1	1	1	1
.ung Squamous Cell Carcinom	TCGA-22-1000	1	1	1	1	1
.ung Squamous Cell Carcinom	TCGA-22-1002	1	1	1	1	1
.ung Squamous Cell Carcinom	TCGA-22-1005	1	1	1	0	1
.ung Squamous Cell Carcinom	TCGA-22-1011	1	1	1	1	1
.ung Squamous Cell Carcinom	TCGA-22-1012	1	1	1	1	1
.ung Squamous Cell Carcinom	TCGA-22-1016	1	1	1	1	1
.ung Squamous Cell Carcinom	TCGA-22-1017	1	1	1	1	1
.ung Squamous Cell Carcinom	TCGA-22-4591	1	1	1	1	1
.ung Squamous Cell Carcinom	TCGA-22-4593	1	1	1	1	1

.ung Squamous Cell Carcinom	TCGA-22-4594	1	1	1	1	1
.ung Squamous Cell Carcinom	TCGA-22-4595	1	1	1	1	1
.ung Squamous Cell Carcinom	TCGA-22-4596	1	1	1	1	1
.ung Squamous Cell Carcinom	TCGA-22-4599	1	1	1	1	1
.ung Squamous Cell Carcinom	TCGA-22-4601	1	1	1	1	1
.ung Squamous Cell Carcinom	TCGA-22-4604	1	1	1	1	1
.ung Squamous Cell Carcinom	TCGA-22-4605	1	1	1	1	1
.ung Squamous Cell Carcinom	TCGA-22-4607	1	1	1	1	1
.ung Squamous Cell Carcinom	TCGA-22-4609	1	1	1	1	1
.ung Squamous Cell Carcinom	TCGA-22-4613	1	1	1	1	1
.ung Squamous Cell Carcinom	TCGA-22-5471	1	1	1	1	1
.ung Squamous Cell Carcinom	TCGA-22-5472	1	1	1	1	1
.ung Squamous Cell Carcinom	TCGA-22-5473	1	1	1	1	1
.ung Squamous Cell Carcinom	TCGA-22-5474	1	1	1	1	1
.ung Squamous Cell Carcinom	TCGA-22-5477	1	1	1	1	1
.ung Squamous Cell Carcinom	TCGA-22-5478	1	1	1	1	1
.ung Squamous Cell Carcinom	TCGA-22-5479	1	1	1	1	1
.ung Squamous Cell Carcinom	TCGA-22-5480	1	1	1	1	1
.ung Squamous Cell Carcinom	TCGA-22-5481	1	1	1	1	1
.ung Squamous Cell Carcinom	TCGA-22-5482	1	1	1	1	1
.ung Squamous Cell Carcinom	TCGA-22-5483	1	1	1	1	1
.ung Squamous Cell Carcinom	TCGA-22-5485	1	1	1	1	1
.ung Squamous Cell Carcinom	TCGA-22-5489	1	0	1	1	1
.ung Squamous Cell Carcinom	TCGA-22-5491	1	1	1	1	1
.ung Squamous Cell Carcinom	TCGA-22-5492	1	1	1	1	1
.ung Squamous Cell Carcinom	TCGA-22-A5C4	1	1	1	1	1
.ung Squamous Cell Carcinom	TCGA-33-4532	1	1	1	1	1
.ung Squamous Cell Carcinom	TCGA-33-4533	1	1	1	1	1
.ung Squamous Cell Carcinom	TCGA-33-4538	1	1	1	1	1
.ung Squamous Cell Carcinom	TCGA-33-4547	1	1	1	1	1
.ung Squamous Cell Carcinom	TCGA-33-4566	1	1	1	1	1
.ung Squamous Cell Carcinom	TCGA-33-4582	1	1	1	1	1
.ung Squamous Cell Carcinom	TCGA-33-4583	1	1	1	1	1
.ung Squamous Cell Carcinom	TCGA-33-4586	1	1	1	1	1
.ung Squamous Cell Carcinom	TCGA-33-4587	1	1	1	1	1
.ung Squamous Cell Carcinom	TCGA-33-4589	1	1	0	1	1
.ung Squamous Cell Carcinom	TCGA-33-6737	1	1	1	1	1
.ung Squamous Cell Carcinom	TCGA-33-6738	1	1	1	1	1
.ung Squamous Cell Carcinom	TCGA-33-A4WN	1	1	1	1	1
.ung Squamous Cell Carcinom	TCGA-33-A5GW	1	1	1	1	1
.ung Squamous Cell Carcinom	TCGA-33-AAS8	1	1	1	1	1
.ung Squamous Cell Carcinom	TCGA-33-AASB	1	1	1	1	1
.ung Squamous Cell Carcinom	TCGA-33-AASD	1	1	1	1	1
.ung Squamous Cell Carcinom	TCGA-33-AASI	1	1	1	1	1
.ung Squamous Cell Carcinom	TCGA-33-AASJ	1	1	1	1	1
.ung Squamous Cell Carcinom	TCGA-33-AASL	1	1	1	1	1
.ung Squamous Cell Carcinom	TCGA-34-2596	1	1	1	1	1
.ung Squamous Cell Carcinom	TCGA-34-2600	1	1	1	1	1
.ung Squamous Cell Carcinom	TCGA-34-2608	1	1	1	1	1
.ung Squamous Cell Carcinom	TCGA-34-5231	1	1	1	1	1
.ung Squamous Cell Carcinom	TCGA-34-5232	1	1	0	1	1
.ung Squamous Cell Carcinom	TCGA-34-5234	0	0	0	0	0
.ung Squamous Cell Carcinom	TCGA-34-5236	1	1	1	1	1
.ung Squamous Cell Carcinom	TCGA-34-5239	1	1	1	1	1
.ung Squamous Cell Carcinom	TCGA-34-5240	1	1	1	0	1
.ung Squamous Cell Carcinom	TCGA-34-5241	1	1	1	1	1
.ung Squamous Cell Carcinom	TCGA-34-5927	1	1	1	1	1
.ung Squamous Cell Carcinom	TCGA-34-5928	1	1	1	1	1

.ung Squamous Cell Carcinom	TCGA-34-5929	1	1	1	1	1
.ung Squamous Cell Carcinom	TCGA-34-7107	1	1	1	1	1
.ung Squamous Cell Carcinom	TCGA-34-8454	1	1	1	1	1
.ung Squamous Cell Carcinom	TCGA-34-8455	1	1	1	1	1
.ung Squamous Cell Carcinom	TCGA-34-8456	1	1	1	1	1
.ung Squamous Cell Carcinom	TCGA-34-A5IX	1	1	1	1	1
.ung Squamous Cell Carcinom	TCGA-37-3783	1	1	1	1	1
.ung Squamous Cell Carcinom	TCGA-37-3789	1	1	1	1	1
.ung Squamous Cell Carcinom	TCGA-37-3792	1	1	1	1	1
.ung Squamous Cell Carcinom	TCGA-37-4129	1	1	1	1	1
.ung Squamous Cell Carcinom	TCGA-37-4130	1	1	1	1	1
.ung Squamous Cell Carcinom	TCGA-37-4132	1	1	1	1	1
.ung Squamous Cell Carcinom	TCGA-37-4133	1	1	1	1	1
.ung Squamous Cell Carcinom	TCGA-37-4135	1	1	1	1	1
.ung Squamous Cell Carcinom	TCGA-37-4141	1	1	1	1	1
.ung Squamous Cell Carcinom	TCGA-37-5819	1	1	1	1	1
.ung Squamous Cell Carcinom	TCGA-37-A5EL	1	1	1	1	1
.ung Squamous Cell Carcinom	TCGA-37-A5EM	0	0	0	0	0
.ung Squamous Cell Carcinom	TCGA-37-A5EN	1	1	1	1	1
.ung Squamous Cell Carcinom	TCGA-39-5011	1	1	1	1	1
.ung Squamous Cell Carcinom	TCGA-39-5016	1	1	1	1	1
.ung Squamous Cell Carcinom	TCGA-39-5019	1	1	1	1	1
.ung Squamous Cell Carcinom	TCGA-39-5021	1	1	1	1	1
.ung Squamous Cell Carcinom	TCGA-39-5022	1	0	0	1	1
.ung Squamous Cell Carcinom	TCGA-39-5024	1	1	1	1	1
.ung Squamous Cell Carcinom	TCGA-39-5027	1	1	1	1	1
.ung Squamous Cell Carcinom	TCGA-39-5028	1	1	0	1	1
.ung Squamous Cell Carcinom	TCGA-39-5029	1	1	1	1	1
.ung Squamous Cell Carcinom	TCGA-39-5030	1	1	1	1	1
.ung Squamous Cell Carcinom	TCGA-39-5031	1	1	1	1	1
.ung Squamous Cell Carcinom	TCGA-39-5034	1	1	1	1	1
.ung Squamous Cell Carcinom	TCGA-39-5035	1	1	1	1	1
.ung Squamous Cell Carcinom	TCGA-39-5036	1	1	1	1	1
.ung Squamous Cell Carcinom	TCGA-39-5037	1	1	1	1	1
.ung Squamous Cell Carcinom	TCGA-39-5039	1	0	1	0	1
.ung Squamous Cell Carcinom	TCGA-39-5040	1	1	1	1	1
.ung Squamous Cell Carcinom	TCGA-43-2576	1	1	1	1	1
.ung Squamous Cell Carcinom	TCGA-43-2578	1	1	1	1	1
.ung Squamous Cell Carcinom	TCGA-43-2581	1	1	1	1	1
.ung Squamous Cell Carcinom	TCGA-43-3394	1	1	1	1	1
.ung Squamous Cell Carcinom	TCGA-43-3920	1	1	1	1	1
.ung Squamous Cell Carcinom	TCGA-43-5668	1	1	1	1	1
.ung Squamous Cell Carcinom	TCGA-43-5670	1	1	1	1	1
.ung Squamous Cell Carcinom	TCGA-43-6143	1	1	1	1	1
.ung Squamous Cell Carcinom	TCGA-43-6647	1	1	1	0	1
.ung Squamous Cell Carcinom	TCGA-43-6770	1	1	1	1	1
.ung Squamous Cell Carcinom	TCGA-43-6771	1	1	0	1	1
.ung Squamous Cell Carcinom	TCGA-43-6773	1	0	1	0	1
.ung Squamous Cell Carcinom	TCGA-43-7656	1	1	1	1	1
.ung Squamous Cell Carcinom	TCGA-43-7657	1	1	1	1	1
.ung Squamous Cell Carcinom	TCGA-43-7658	1	0	1	1	1
.ung Squamous Cell Carcinom	TCGA-43-8115	1	1	1	1	1
.ung Squamous Cell Carcinom	TCGA-43-8116	1	1	1	1	1
.ung Squamous Cell Carcinom	TCGA-43-8118	1	1	1	1	1
.ung Squamous Cell Carcinom	TCGA-43-A474	1	1	1	1	1
.ung Squamous Cell Carcinom	TCGA-43-A475	1	0	1	1	1
.ung Squamous Cell Carcinom	TCGA-43-A56U	1	1	1	1	1
.ung Squamous Cell Carcinom	TCGA-43-A56V	1	1	1	1	1

.ung Squamous Cell Carcinom	TCGA-46-3765	1	1	1	1	1
.ung Squamous Cell Carcinom	TCGA-46-3766	1	1	1	1	1
.ung Squamous Cell Carcinom	TCGA-46-3767	1	1	1	1	1
.ung Squamous Cell Carcinom	TCGA-46-3768	1	1	1	1	1
.ung Squamous Cell Carcinom	TCGA-46-3769	1	1	0	0	1
.ung Squamous Cell Carcinom	TCGA-46-6025	1	1	1	1	1
.ung Squamous Cell Carcinom	TCGA-46-6026	1	1	1	1	1
.ung Squamous Cell Carcinom	TCGA-51-4079	1	1	1	1	1
.ung Squamous Cell Carcinom	TCGA-51-4080	1	1	1	1	1
.ung Squamous Cell Carcinom	TCGA-51-4081	1	1	1	1	1
.ung Squamous Cell Carcinom	TCGA-51-6867	0	0	0	0	0
.ung Squamous Cell Carcinom	TCGA-52-7622	1	1	1	1	1
.ung Squamous Cell Carcinom	TCGA-52-7809	1	1	1	1	1
.ung Squamous Cell Carcinom	TCGA-52-7810	1	1	1	1	1
.ung Squamous Cell Carcinom	TCGA-52-7811	1	1	1	1	1
.ung Squamous Cell Carcinom	TCGA-52-7812	1	1	1	1	1
.ung Squamous Cell Carcinom	TCGA-56-1622	1	1	1	1	1
.ung Squamous Cell Carcinom	TCGA-56-5897	1	1	1	1	1
.ung Squamous Cell Carcinom	TCGA-56-5898	1	1	1	1	1
.ung Squamous Cell Carcinom	TCGA-56-6545	1	1	1	1	1
.ung Squamous Cell Carcinom	TCGA-56-6546	1	1	1	1	1
.ung Squamous Cell Carcinom	TCGA-56-7221	1	1	1	1	1
.ung Squamous Cell Carcinom	TCGA-56-7222	1	1	1	1	1
.ung Squamous Cell Carcinom	TCGA-56-7223	1	1	1	1	1
.ung Squamous Cell Carcinom	TCGA-56-7579	1	1	1	1	1
.ung Squamous Cell Carcinom	TCGA-56-7580	1	1	1	1	1
.ung Squamous Cell Carcinom	TCGA-56-7582	1	1	1	1	1
.ung Squamous Cell Carcinom	TCGA-56-7730	1	1	1	1	1
.ung Squamous Cell Carcinom	TCGA-56-7731	1	1	1	1	1
.ung Squamous Cell Carcinom	TCGA-56-7822	1	1	1	1	1
.ung Squamous Cell Carcinom	TCGA-56-7823	1	1	1	1	1
.ung Squamous Cell Carcinom	TCGA-56-8082	1	1	1	1	1
.ung Squamous Cell Carcinom	TCGA-56-8083	1	1	1	1	1
.ung Squamous Cell Carcinom	TCGA-56-8201	1	1	0	1	1
.ung Squamous Cell Carcinom	TCGA-56-8304	1	1	1	1	1
.ung Squamous Cell Carcinom	TCGA-56-8305	1	1	1	1	1
.ung Squamous Cell Carcinom	TCGA-56-8307	1	1	1	1	1
.ung Squamous Cell Carcinom	TCGA-56-8308	1	1	1	1	1
.ung Squamous Cell Carcinom	TCGA-56-8309	1	1	1	1	1
.ung Squamous Cell Carcinom	TCGA-56-8503	1	1	1	1	1
.ung Squamous Cell Carcinom	TCGA-56-8504	1	1	0	1	1
.ung Squamous Cell Carcinom	TCGA-56-8622	1	1	1	1	1
.ung Squamous Cell Carcinom	TCGA-56-8623	0	0	0	0	0
.ung Squamous Cell Carcinom	TCGA-56-8624	1	1	1	1	1
.ung Squamous Cell Carcinom	TCGA-56-8625	1	1	1	1	1
.ung Squamous Cell Carcinom	TCGA-56-8626	1	1	1	1	1
.ung Squamous Cell Carcinom	TCGA-56-8628	1	1	1	1	1
.ung Squamous Cell Carcinom	TCGA-56-8629	1	1	1	1	1
.ung Squamous Cell Carcinom	TCGA-56-A49D	1	1	1	1	1
.ung Squamous Cell Carcinom	TCGA-56-A4BW	1	1	1	1	1
.ung Squamous Cell Carcinom	TCGA-56-A4BX	1	1	1	1	1
.ung Squamous Cell Carcinom	TCGA-56-A4BY	1	1	1	1	1
.ung Squamous Cell Carcinom	TCGA-56-A4ZJ	1	1	1	1	1
.ung Squamous Cell Carcinom	TCGA-56-A4ZK	1	1	1	1	1
.ung Squamous Cell Carcinom	TCGA-56-A5DR	1	1	1	0	1
.ung Squamous Cell Carcinom	TCGA-56-A5DS	1	1	1	1	1
.ung Squamous Cell Carcinom	TCGA-56-A62T	1	1	1	1	1
.ung Squamous Cell Carcinom	TCGA-58-8386	1	1	1	1	1

.ung Squamous Cell Carcinom	TCGA-58-8387	1	1	1	0	1
.ung Squamous Cell Carcinom	TCGA-58-8388	1	1	1	1	1
.ung Squamous Cell Carcinom	TCGA-58-8390	1	1	1	1	1
.ung Squamous Cell Carcinom	TCGA-58-8391	1	1	1	1	1
.ung Squamous Cell Carcinom	TCGA-58-8392	1	1	1	1	1
.ung Squamous Cell Carcinom	TCGA-58-8393	1	1	1	1	1
.ung Squamous Cell Carcinom	TCGA-58-A46J	1	1	1	0	1
.ung Squamous Cell Carcinom	TCGA-58-A46K	1	1	1	1	1
.ung Squamous Cell Carcinom	TCGA-58-A46L	1	1	1	1	1
.ung Squamous Cell Carcinom	TCGA-58-A46M	1	1	1	1	1
.ung Squamous Cell Carcinom	TCGA-58-A46N	1	0	0	0	0
.ung Squamous Cell Carcinom	TCGA-60-2695	1	1	1	1	1
.ung Squamous Cell Carcinom	TCGA-60-2696	1	1	1	1	1
.ung Squamous Cell Carcinom	TCGA-60-2697	0	0	0	0	0
.ung Squamous Cell Carcinom	TCGA-60-2698	1	1	1	1	1
.ung Squamous Cell Carcinom	TCGA-60-2703	1	1	1	1	1
.ung Squamous Cell Carcinom	TCGA-60-2704	1	0	0	1	1
.ung Squamous Cell Carcinom	TCGA-60-2706	1	1	1	1	1
.ung Squamous Cell Carcinom	TCGA-60-2707	1	1	1	1	1
.ung Squamous Cell Carcinom	TCGA-60-2708	1	1	1	1	1
.ung Squamous Cell Carcinom	TCGA-60-2709	1	0	1	1	1
.ung Squamous Cell Carcinom	TCGA-60-2710	1	1	1	1	1
.ung Squamous Cell Carcinom	TCGA-60-2711	1	1	1	1	1
.ung Squamous Cell Carcinom	TCGA-60-2712	1	1	1	1	1
.ung Squamous Cell Carcinom	TCGA-60-2713	1	1	1	1	1
.ung Squamous Cell Carcinom	TCGA-60-2714	1	1	1	1	1
.ung Squamous Cell Carcinom	TCGA-60-2715	1	0	0	1	1
.ung Squamous Cell Carcinom	TCGA-60-2716	0	0	0	0	0
.ung Squamous Cell Carcinom	TCGA-60-2719	1	1	1	1	1
.ung Squamous Cell Carcinom	TCGA-60-2720	1	1	1	1	1
.ung Squamous Cell Carcinom	TCGA-60-2721	1	1	1	1	1
.ung Squamous Cell Carcinom	TCGA-60-2722	1	1	1	1	1
.ung Squamous Cell Carcinom	TCGA-60-2723	1	1	1	1	1
.ung Squamous Cell Carcinom	TCGA-60-2724	1	1	1	1	1
.ung Squamous Cell Carcinom	TCGA-60-2725	1	1	1	1	1
.ung Squamous Cell Carcinom	TCGA-60-2726	1	1	1	1	1
.ung Squamous Cell Carcinom	TCGA-63-5128	1	1	1	1	1
.ung Squamous Cell Carcinom	TCGA-63-5131	1	1	1	1	1
.ung Squamous Cell Carcinom	TCGA-63-6202	1	1	1	1	1
.ung Squamous Cell Carcinom	TCGA-63-7020	1	1	1	1	1
.ung Squamous Cell Carcinom	TCGA-63-7021	1	0	1	1	1
.ung Squamous Cell Carcinom	TCGA-63-7022	1	1	1	1	1
.ung Squamous Cell Carcinom	TCGA-63-7023	1	1	1	1	1
.ung Squamous Cell Carcinom	TCGA-63-A5M9	1	1	1	1	1
.ung Squamous Cell Carcinom	TCGA-63-A5MB	1	1	1	1	1
.ung Squamous Cell Carcinom	TCGA-63-A5MG	1	1	1	0	1
.ung Squamous Cell Carcinom	TCGA-63-A5MH	1	1	1	1	1
.ung Squamous Cell Carcinom	TCGA-63-A5MI	1	1	1	1	1
.ung Squamous Cell Carcinom	TCGA-63-A5MJ	1	1	1	1	1
.ung Squamous Cell Carcinom	TCGA-63-A5ML	1	1	1	1	1
.ung Squamous Cell Carcinom	TCGA-63-A5MM	0	1	0	0	1
.ung Squamous Cell Carcinom	TCGA-63-A5MN	0	0	0	1	1
.ung Squamous Cell Carcinom	TCGA-63-A5MP	1	1	1	1	1
.ung Squamous Cell Carcinom	TCGA-63-A5MR	1	1	1	1	1
.ung Squamous Cell Carcinom	TCGA-63-A5MS	1	1	1	1	1
.ung Squamous Cell Carcinom	TCGA-63-A5MT	1	1	1	1	1
.ung Squamous Cell Carcinom	TCGA-63-A5MU	1	1	1	1	1
.ung Squamous Cell Carcinom	TCGA-63-A5MV	1	1	1	1	1

.ung Squamous Cell Carcinom	TCGA-63-A5MW	1	1	1	1	1
.ung Squamous Cell Carcinom	TCGA-63-A5MY	1	1	1	1	1
.ung Squamous Cell Carcinom	TCGA-66-2727	1	1	1	1	1
.ung Squamous Cell Carcinom	TCGA-66-2734	1	1	1	1	1
.ung Squamous Cell Carcinom	TCGA-66-2737	1	1	1	1	1
.ung Squamous Cell Carcinom	TCGA-66-2742	1	1	1	1	1
.ung Squamous Cell Carcinom	TCGA-66-2744	1	0	1	1	1
.ung Squamous Cell Carcinom	TCGA-66-2753	1	1	1	1	1
.ung Squamous Cell Carcinom	TCGA-66-2754	1	1	1	1	1
.ung Squamous Cell Carcinom	TCGA-66-2755	1	1	1	1	1
.ung Squamous Cell Carcinom	TCGA-66-2756	1	1	1	1	1
.ung Squamous Cell Carcinom	TCGA-66-2757	1	1	1	1	1
.ung Squamous Cell Carcinom	TCGA-66-2758	1	1	1	1	1
.ung Squamous Cell Carcinom	TCGA-66-2759	1	1	1	1	1
.ung Squamous Cell Carcinom	TCGA-66-2763	1	1	1	1	1
.ung Squamous Cell Carcinom	TCGA-66-2765	1	1	1	1	1
.ung Squamous Cell Carcinom	TCGA-66-2766	1	1	1	1	1
.ung Squamous Cell Carcinom	TCGA-66-2767	1	1	1	1	1
.ung Squamous Cell Carcinom	TCGA-66-2768	1	1	1	1	1
.ung Squamous Cell Carcinom	TCGA-66-2769	1	1	1	1	1
.ung Squamous Cell Carcinom	TCGA-66-2770	1	1	0	1	1
.ung Squamous Cell Carcinom	TCGA-66-2771	1	1	1	1	1
.ung Squamous Cell Carcinom	TCGA-66-2773	1	1	1	1	1
.ung Squamous Cell Carcinom	TCGA-66-2777	1	1	1	1	1
.ung Squamous Cell Carcinom	TCGA-66-2778	1	1	0	1	1
.ung Squamous Cell Carcinom	TCGA-66-2780	1	1	1	1	1
.ung Squamous Cell Carcinom	TCGA-66-2781	1	1	1	1	1
.ung Squamous Cell Carcinom	TCGA-66-2782	1	1	1	1	1
.ung Squamous Cell Carcinom	TCGA-66-2783	1	1	1	1	1
.ung Squamous Cell Carcinom	TCGA-66-2785	1	1	1	1	1
.ung Squamous Cell Carcinom	TCGA-66-2786	1	1	1	1	1
.ung Squamous Cell Carcinom	TCGA-66-2787	1	1	1	1	1
.ung Squamous Cell Carcinom	TCGA-66-2788	1	1	1	1	1
.ung Squamous Cell Carcinom	TCGA-66-2789	1	1	1	1	1
.ung Squamous Cell Carcinom	TCGA-66-2790	1	1	1	1	1
.ung Squamous Cell Carcinom	TCGA-66-2791	1	1	1	1	1
.ung Squamous Cell Carcinom	TCGA-66-2792	1	1	1	1	1
.ung Squamous Cell Carcinom	TCGA-66-2793	1	1	1	1	1
.ung Squamous Cell Carcinom	TCGA-66-2794	1	1	1	1	1
.ung Squamous Cell Carcinom	TCGA-66-2795	1	1	1	1	1
.ung Squamous Cell Carcinom	TCGA-66-2800	1	1	1	1	1
.ung Squamous Cell Carcinom	TCGA-68-7755	1	1	1	1	1
.ung Squamous Cell Carcinom	TCGA-68-7756	1	1	1	1	1
.ung Squamous Cell Carcinom	TCGA-68-7757	1	1	1	1	1
.ung Squamous Cell Carcinom	TCGA-68-8250	1	1	1	1	1
.ung Squamous Cell Carcinom	TCGA-68-8251	1	1	1	1	1
.ung Squamous Cell Carcinom	TCGA-68-A59I	1	1	1	1	1
.ung Squamous Cell Carcinom	TCGA-68-A59J	1	1	1	1	1
.ung Squamous Cell Carcinom	TCGA-6A-AB49	1	1	1	1	1
.ung Squamous Cell Carcinom	TCGA-70-6722	1	1	1	1	1
.ung Squamous Cell Carcinom	TCGA-70-6723	1	1	1	1	1
.ung Squamous Cell Carcinom	TCGA-77-6842	1	1	1	1	1
.ung Squamous Cell Carcinom	TCGA-77-6843	1	1	1	1	1
.ung Squamous Cell Carcinom	TCGA-77-6844	1	1	1	1	1
.ung Squamous Cell Carcinom	TCGA-77-6845	1	1	1	1	1
.ung Squamous Cell Carcinom	TCGA-77-7138	1	1	1	1	1
.ung Squamous Cell Carcinom	TCGA-77-7139	1	1	1	1	1
.ung Squamous Cell Carcinom	TCGA-77-7140	1	0	0	0	1

.ung Squamous Cell Carcinom	TCGA-77-7141	1	1	1	1	1
.ung Squamous Cell Carcinom	TCGA-77-7142	1	1	1	1	1
.ung Squamous Cell Carcinom	TCGA-77-7335	1	1	1	1	1
.ung Squamous Cell Carcinom	TCGA-77-7337	1	1	1	1	1
.ung Squamous Cell Carcinom	TCGA-77-7338	1	1	1	1	1
.ung Squamous Cell Carcinom	TCGA-77-7463	1	1	1	1	1
.ung Squamous Cell Carcinom	TCGA-77-7465	1	1	1	1	1
.ung Squamous Cell Carcinom	TCGA-77-8007	1	1	1	1	1
.ung Squamous Cell Carcinom	TCGA-77-8008	1	1	1	1	1
.ung Squamous Cell Carcinom	TCGA-77-8009	1	1	1	1	1
.ung Squamous Cell Carcinom	TCGA-77-8128	1	1	1	1	1
.ung Squamous Cell Carcinom	TCGA-77-8130	1	1	1	1	1
.ung Squamous Cell Carcinom	TCGA-77-8131	1	1	1	1	1
.ung Squamous Cell Carcinom	TCGA-77-8133	1	1	1	1	1
.ung Squamous Cell Carcinom	TCGA-77-8136	1	1	1	1	1
.ung Squamous Cell Carcinom	TCGA-77-8138	1	1	1	1	1
.ung Squamous Cell Carcinom	TCGA-77-8139	1	1	1	1	1
.ung Squamous Cell Carcinom	TCGA-77-8140	1	1	1	1	1
.ung Squamous Cell Carcinom	TCGA-77-8143	1	1	1	1	1
.ung Squamous Cell Carcinom	TCGA-77-8144	1	1	1	1	1
.ung Squamous Cell Carcinom	TCGA-77-8145	1	1	1	1	1
.ung Squamous Cell Carcinom	TCGA-77-8146	0	0	0	0	0
.ung Squamous Cell Carcinom	TCGA-77-8148	1	1	1	1	1
.ung Squamous Cell Carcinom	TCGA-77-8150	1	1	1	1	1
.ung Squamous Cell Carcinom	TCGA-77-8153	1	1	1	1	1
.ung Squamous Cell Carcinom	TCGA-77-8154	1	1	1	1	1
.ung Squamous Cell Carcinom	TCGA-77-8156	1	1	1	1	1
.ung Squamous Cell Carcinom	TCGA-77-A5FZ	0	0	0	0	0
.ung Squamous Cell Carcinom	TCGA-77-A5G1	1	1	1	1	1
.ung Squamous Cell Carcinom	TCGA-77-A5G3	1	1	1	1	1
.ung Squamous Cell Carcinom	TCGA-77-A5G6	1	1	1	1	1
.ung Squamous Cell Carcinom	TCGA-77-A5G7	1	1	1	1	1
.ung Squamous Cell Carcinom	TCGA-77-A5G8	1	1	1	1	1
.ung Squamous Cell Carcinom	TCGA-77-A5GA	1	1	1	1	1
.ung Squamous Cell Carcinom	TCGA-77-A5GB	1	1	1	1	1
.ung Squamous Cell Carcinom	TCGA-77-A5GF	1	1	1	1	1
.ung Squamous Cell Carcinom	TCGA-77-A5GH	1	1	1	1	1
.ung Squamous Cell Carcinom	TCGA-79-5596	1	1	1	1	1
.ung Squamous Cell Carcinom	TCGA-85-6175	1	1	1	1	1
.ung Squamous Cell Carcinom	TCGA-85-6560	1	1	1	1	1
.ung Squamous Cell Carcinom	TCGA-85-6561	1	1	1	1	1
.ung Squamous Cell Carcinom	TCGA-85-6798	1	1	1	1	1
.ung Squamous Cell Carcinom	TCGA-85-7696	1	1	1	1	1
.ung Squamous Cell Carcinom	TCGA-85-7697	1	1	1	1	1
.ung Squamous Cell Carcinom	TCGA-85-7698	1	1	1	1	1
.ung Squamous Cell Carcinom	TCGA-85-7699	1	1	1	1	1
.ung Squamous Cell Carcinom	TCGA-85-7710	1	1	1	1	1
.ung Squamous Cell Carcinom	TCGA-85-7843	1	1	1	1	1
.ung Squamous Cell Carcinom	TCGA-85-7844	1	1	1	1	1
.ung Squamous Cell Carcinom	TCGA-85-7950	1	1	1	1	1
.ung Squamous Cell Carcinom	TCGA-85-8048	1	1	0	1	1
.ung Squamous Cell Carcinom	TCGA-85-8049	1	1	1	1	1
.ung Squamous Cell Carcinom	TCGA-85-8052	1	1	1	1	1
.ung Squamous Cell Carcinom	TCGA-85-8070	1	1	1	1	1
.ung Squamous Cell Carcinom	TCGA-85-8071	1	1	1	1	1
.ung Squamous Cell Carcinom	TCGA-85-8072	1	1	1	1	1
.ung Squamous Cell Carcinom	TCGA-85-8276	1	1	1	1	1
.ung Squamous Cell Carcinom	TCGA-85-8277	1	1	1	1	1

.ung Squamous Cell Carcinom	TCGA-85-8287	1	1	1	1	1
.ung Squamous Cell Carcinom	TCGA-85-8288	1	1	1	1	1
.ung Squamous Cell Carcinom	TCGA-85-8350	1	1	1	1	1
.ung Squamous Cell Carcinom	TCGA-85-8351	1	1	1	1	1
.ung Squamous Cell Carcinom	TCGA-85-8352	1	1	1	1	1
.ung Squamous Cell Carcinom	TCGA-85-8353	1	1	1	1	1
.ung Squamous Cell Carcinom	TCGA-85-8354	1	1	1	0	1
.ung Squamous Cell Carcinom	TCGA-85-8355	1	1	1	1	1
.ung Squamous Cell Carcinom	TCGA-85-8479	1	1	1	1	1
.ung Squamous Cell Carcinom	TCGA-85-8481	1	1	1	1	1
.ung Squamous Cell Carcinom	TCGA-85-8580	1	1	1	1	1
.ung Squamous Cell Carcinom	TCGA-85-8582	1	0	0	0	1
.ung Squamous Cell Carcinom	TCGA-85-8584	1	1	1	1	1
.ung Squamous Cell Carcinom	TCGA-85-8664	1	1	1	1	1
.ung Squamous Cell Carcinom	TCGA-85-8666	1	1	1	1	1
.ung Squamous Cell Carcinom	TCGA-85-A4CL	1	1	1	1	1
.ung Squamous Cell Carcinom	TCGA-85-A4CN	1	1	1	1	1
.ung Squamous Cell Carcinom	TCGA-85-A4JB	1	1	1	1	1
.ung Squamous Cell Carcinom	TCGA-85-A4JC	1	1	1	1	1
.ung Squamous Cell Carcinom	TCGA-85-A4PA	0	0	0	0	0
.ung Squamous Cell Carcinom	TCGA-85-A4QQ	1	1	1	1	1
.ung Squamous Cell Carcinom	TCGA-85-A4QR	1	1	1	1	1
.ung Squamous Cell Carcinom	TCGA-85-A50M	1	1	1	1	1
.ung Squamous Cell Carcinom	TCGA-85-A50Z	1	1	1	1	1
.ung Squamous Cell Carcinom	TCGA-85-A510	1	1	1	1	1
.ung Squamous Cell Carcinom	TCGA-85-A511	1	1	1	1	1
.ung Squamous Cell Carcinom	TCGA-85-A512	1	1	1	1	1
.ung Squamous Cell Carcinom	TCGA-85-A513	1	1	1	1	1
.ung Squamous Cell Carcinom	TCGA-85-A53L	1	1	1	1	1
.ung Squamous Cell Carcinom	TCGA-85-A5B5	1	1	1	1	1
.ung Squamous Cell Carcinom	TCGA-90-6837	0	0	0	0	0
.ung Squamous Cell Carcinom	TCGA-90-7766	1	1	1	1	1
.ung Squamous Cell Carcinom	TCGA-90-7767	1	1	1	1	1
.ung Squamous Cell Carcinom	TCGA-90-7769	1	1	1	1	1
.ung Squamous Cell Carcinom	TCGA-90-7964	1	1	1	1	1
.ung Squamous Cell Carcinom	TCGA-90-A4ED	1	1	1	1	1
.ung Squamous Cell Carcinom	TCGA-90-A4EE	1	1	1	1	1
.ung Squamous Cell Carcinom	TCGA-90-A59Q	1	1	1	1	1
.ung Squamous Cell Carcinom	TCGA-92-7340	1	1	1	0	1
.ung Squamous Cell Carcinom	TCGA-92-7341	1	1	1	1	1
.ung Squamous Cell Carcinom	TCGA-92-8063	1	1	1	1	1
.ung Squamous Cell Carcinom	TCGA-92-8064	1	1	1	1	1
.ung Squamous Cell Carcinom	TCGA-92-8065	1	1	1	1	1
.ung Squamous Cell Carcinom	TCGA-94-7033	1	1	1	1	1
.ung Squamous Cell Carcinom	TCGA-94-7557	1	1	1	1	1
.ung Squamous Cell Carcinom	TCGA-94-7943	1	1	1	1	1
.ung Squamous Cell Carcinom	TCGA-94-8035	1	1	1	1	1
.ung Squamous Cell Carcinom	TCGA-94-8490	1	1	1	1	1
.ung Squamous Cell Carcinom	TCGA-94-8491	1	1	1	1	1
.ung Squamous Cell Carcinom	TCGA-94-A4VJ	1	1	1	1	1
.ung Squamous Cell Carcinom	TCGA-94-A5I4	1	1	1	1	1
.ung Squamous Cell Carcinom	TCGA-94-A5I6	1	1	1	1	1
.ung Squamous Cell Carcinom	TCGA-96-7544	1	1	1	1	1
.ung Squamous Cell Carcinom	TCGA-96-7545	1	1	1	1	1
.ung Squamous Cell Carcinom	TCGA-96-8169	1	1	1	1	1
.ung Squamous Cell Carcinom	TCGA-96-8170	1	1	1	1	1
.ung Squamous Cell Carcinom	TCGA-96-A4JK	1	1	1	1	1
.ung Squamous Cell Carcinom	TCGA-96-A4JL	1	1	1	1	1

.ung Squamous Cell Carcinom	TCGA-98-7454	1	0	0	1	1
.ung Squamous Cell Carcinom	TCGA-98-8020	1	1	1	1	1
.ung Squamous Cell Carcinom	TCGA-98-8021	1	1	1	1	1
.ung Squamous Cell Carcinom	TCGA-98-8022	1	1	1	1	1
.ung Squamous Cell Carcinom	TCGA-98-8023	1	1	1	1	1
.ung Squamous Cell Carcinom	TCGA-98-A538	1	1	1	1	1
.ung Squamous Cell Carcinom	TCGA-98-A539	1	1	1	1	1
.ung Squamous Cell Carcinom	TCGA-98-A53A	1	1	1	1	1
.ung Squamous Cell Carcinom	TCGA-98-A53B	1	1	1	1	1
.ung Squamous Cell Carcinom	TCGA-98-A53C	0	0	0	0	0
.ung Squamous Cell Carcinom	TCGA-98-A53D	0	0	0	0	0
.ung Squamous Cell Carcinom	TCGA-98-A53H	0	0	0	0	0
.ung Squamous Cell Carcinom	TCGA-98-A53I	1	1	1	1	1
.ung Squamous Cell Carcinom	TCGA-98-A53J	1	1	1	1	1
.ung Squamous Cell Carcinom	TCGA-J1-A4AH	1	1	1	1	1
.ung Squamous Cell Carcinom	TCGA-L3-A4E7	1	1	1	1	1
.ung Squamous Cell Carcinom	TCGA-L3-A524	1	1	1	1	1
.ung Squamous Cell Carcinom	TCGA-LA-A446	1	1	1	1	1
.ung Squamous Cell Carcinom	TCGA-LA-A7SW	1	1	1	1	1
.ung Squamous Cell Carcinom	TCGA-MF-A522	1	1	1	1	1
.ung Squamous Cell Carcinom	TCGA-NC-A5HD	1	1	1	1	1
.ung Squamous Cell Carcinom	TCGA-NC-A5HE	1	1	0	1	1
.ung Squamous Cell Carcinom	TCGA-NC-A5HF	1	1	1	1	1
.ung Squamous Cell Carcinom	TCGA-NC-A5HG	1	1	1	1	1
.ung Squamous Cell Carcinom	TCGA-NC-A5HH	1	1	1	0	0
.ung Squamous Cell Carcinom	TCGA-NC-A5HI	1	1	1	1	1
.ung Squamous Cell Carcinom	TCGA-NC-A5HJ	1	1	1	1	1
.ung Squamous Cell Carcinom	TCGA-NC-A5HK	1	1	1	1	1
.ung Squamous Cell Carcinom	TCGA-NC-A5HL	1	1	1	1	1
.ung Squamous Cell Carcinom	TCGA-NC-A5HM	1	1	1	1	1
.ung Squamous Cell Carcinom	TCGA-NC-A5HN	1	1	1	1	1
.ung Squamous Cell Carcinom	TCGA-NC-A5HO	1	1	1	1	1
.ung Squamous Cell Carcinom	TCGA-NC-A5HP	1	1	1	1	1
.ung Squamous Cell Carcinom	TCGA-NC-A5HQ	1	1	1	1	1
.ung Squamous Cell Carcinom	TCGA-NC-A5HR	1	1	1	1	1
.ung Squamous Cell Carcinom	TCGA-NC-A5HT	1	1	1	0	1
.ung Squamous Cell Carcinom	TCGA-NK-A5CR	1	1	1	1	1
.ung Squamous Cell Carcinom	TCGA-NK-A5CT	1	1	1	1	1
.ung Squamous Cell Carcinom	TCGA-NK-A5CX	1	1	1	1	1
.ung Squamous Cell Carcinom	TCGA-NK-A5D1	1	1	1	1	1
.ung Squamous Cell Carcinom	TCGA-NK-A7XE	1	0	1	0	1
.ung Squamous Cell Carcinom	TCGA-O2-A52N	1	1	1	1	1
.ung Squamous Cell Carcinom	TCGA-O2-A52Q	1	1	1	1	1
.ung Squamous Cell Carcinom	TCGA-O2-A52S	1	1	1	1	1
.ung Squamous Cell Carcinom	TCGA-O2-A52V	1	1	1	1	1
.ung Squamous Cell Carcinom	TCGA-O2-A52W	1	1	1	1	1
.ung Squamous Cell Carcinom	TCGA-O2-A5IB	1	1	1	1	1
.ung Squamous Cell Carcinom	TCGA-XC-AA0X	1	1	1	1	1
Lymphoid Neoplasm	TCGA-FA-8693	0	0	0	1	1
Lymphoid Neoplasm	TCGA-FA-A4BB	0	0	0	0	0
Lymphoid Neoplasm	TCGA-FA-A4XK	0	0	0	0	1
Lymphoid Neoplasm	TCGA-FA-A6HN	1	0	1	1	1
Lymphoid Neoplasm	TCGA-FA-A6HO	1	1	1	1	1
Lymphoid Neoplasm	TCGA-FA-A7DS	1	0	1	0	1
Lymphoid Neoplasm	TCGA-FA-A7Q1	1	1	0	1	1
Lymphoid Neoplasm	TCGA-FA-A82F	1	1	1	1	1
Lymphoid Neoplasm	TCGA-FA-A86F	1	1	0	1	1
Lymphoid Neoplasm	TCGA-FF-8041	0	0	0	0	0

Lymphoid Neoplasm	TCGA-FF-8042	1	0	1	1	1
Lymphoid Neoplasm	TCGA-FF-8043	1	0	1	1	1
Lymphoid Neoplasm	TCGA-FF-8046	1	0	0	1	1
Lymphoid Neoplasm	TCGA-FF-8047	1	1	0	1	1
Lymphoid Neoplasm	TCGA-FF-8061	0	1	1	0	0
Lymphoid Neoplasm	TCGA-FF-8062	1	0	0	1	0
Lymphoid Neoplasm	TCGA-FF-A7CQ	1	0	0	0	1
Lymphoid Neoplasm	TCGA-FF-A7CR	0	1	1	0	1
Lymphoid Neoplasm	TCGA-FF-A7CW	1	1	1	0	1
Lymphoid Neoplasm	TCGA-FF-A7CX	0	0	1	0	0
Lymphoid Neoplasm	TCGA-FM-8000	0	0	0	0	1
Lymphoid Neoplasm	TCGA-G8-6324	0	0	0	0	1
Lymphoid Neoplasm	TCGA-G8-6325	1	1	1	1	1
Lymphoid Neoplasm	TCGA-G8-6326	1	0	0	0	1
Lymphoid Neoplasm	TCGA-G8-6906	0	0	1	1	1
Lymphoid Neoplasm	TCGA-G8-6907	1	1	0	0	1
Lymphoid Neoplasm	TCGA-G8-6909	1	1	0	0	1
Lymphoid Neoplasm	TCGA-G8-6914	1	0	0	1	0
Lymphoid Neoplasm	TCGA-GR-7351	0	1	1	0	1
Lymphoid Neoplasm	TCGA-GR-7353	1	1	0	1	1
Lymphoid Neoplasm	TCGA-GR-A4D4	1	1	1	1	1
Lymphoid Neoplasm	TCGA-GR-A4D5	1	0	1	0	0
Lymphoid Neoplasm	TCGA-GR-A4D6	1	1	1	1	1
Lymphoid Neoplasm	TCGA-GR-A4D9	1	0	1	0	1
Lymphoid Neoplasm	TCGA-GS-A9TQ	1	1	1	1	1
Lymphoid Neoplasm	TCGA-GS-A9TT	0	0	1	0	1
Lymphoid Neoplasm	TCGA-GS-A9TU	1	0	0	0	1
Lymphoid Neoplasm	TCGA-GS-A9TV	1	1	0	1	1
Lymphoid Neoplasm	TCGA-GS-A9TW	0	0	1	1	1
Lymphoid Neoplasm	TCGA-GS-A9TX	0	0	1	0	1
Lymphoid Neoplasm	TCGA-GS-A9TY	1	1	1	1	1
Lymphoid Neoplasm	TCGA-GS-A9TZ	1	1	1	1	1
Lymphoid Neoplasm	TCGA-GS-A9U3	0	0	0	0	1
Lymphoid Neoplasm	TCGA-GS-A9U4	0	0	1	1	1
Lymphoid Neoplasm	TCGA-RQ-A68N	1	1	1	1	1
Lymphoid Neoplasm	TCGA-RQ-A6JB	1	1	1	0	1
Lymphoid Neoplasm	TCGA-RQ-AAAT	1	1	1	1	1
Lymphoid Neoplasm	TCGA-VB-A8QN	1	0	1	0	1
Mesothelioma	TCGA-3H-AB3K	0	1	1	1	1
Mesothelioma	TCGA-3H-AB3L	1	1	1	1	1
Mesothelioma	TCGA-3H-AB3M	1	1	1	1	1
Mesothelioma	TCGA-3H-AB3O	0	0	0	0	0
Mesothelioma	TCGA-3H-AB3S	1	0	0	1	1
Mesothelioma	TCGA-3H-AB3T	1	1	1	1	1
Mesothelioma	TCGA-3H-AB3U	0	0	0	0	0
Mesothelioma	TCGA-3H-AB3X	1	1	1	1	1
Mesothelioma	TCGA-3U-A98D	1	1	1	1	1
Mesothelioma	TCGA-3U-A98E	0	0	1	0	0
Mesothelioma	TCGA-3U-A98F	1	1	1	1	1
Mesothelioma	TCGA-3U-A98G	0	0	0	0	0
Mesothelioma	TCGA-3U-A98H	0	0	0	1	1
Mesothelioma	TCGA-3U-A98I	1	1	1	1	1
Mesothelioma	TCGA-3U-A98J	1	1	0	1	1
Mesothelioma	TCGA-LK-A4NW	1	1	1	0	1
Mesothelioma	TCGA-LK-A4NY	1	1	1	1	1
Mesothelioma	TCGA-LK-A4NZ	0	1	1	1	1
Mesothelioma	TCGA-LK-A4O0	1	1	1	1	1
Mesothelioma	TCGA-LK-A4O2	1	1	1	1	1

Mesothelioma	TCGA-LK-A4O4	0	0	0	0	0
Mesothelioma	TCGA-LK-A4O5	1	1	1	1	1
Mesothelioma	TCGA-LK-A4O6	1	1	0	1	1
Mesothelioma	TCGA-LK-A4O7	1	1	1	1	1
Mesothelioma	TCGA-MQ-A4KX	0	0	1	0	0
Mesothelioma	TCGA-MQ-A4LC	0	1	1	1	1
Mesothelioma	TCGA-MQ-A4LI	1	1	1	1	1
Mesothelioma	TCGA-MQ-A4LJ	1	1	0	1	1
Mesothelioma	TCGA-MQ-A4LM	1	1	1	1	1
Mesothelioma	TCGA-MQ-A4LP	1	1	1	1	1
Mesothelioma	TCGA-MQ-A4LV	1	1	1	1	1
Mesothelioma	TCGA-MQ-A6BL	1	1	1	1	1
Mesothelioma	TCGA-MQ-A6BN	1	0	1	0	1
Mesothelioma	TCGA-MQ-A6BQ	1	1	1	1	1
Mesothelioma	TCGA-MQ-A6BR	1	0	0	0	1
Mesothelioma	TCGA-MQ-A6BS	1	1	1	1	1
Mesothelioma	TCGA-NQ-A57I	1	1	1	1	1
Mesothelioma	TCGA-NQ-A638	1	1	1	1	1
Mesothelioma	TCGA-SC-A6LM	1	1	1	1	1
Mesothelioma	TCGA-SC-A6LN	1	1	1	1	1
Mesothelioma	TCGA-SC-A6LP	0	0	0	0	1
Mesothelioma	TCGA-SC-A6LQ	1	1	1	1	1
Mesothelioma	TCGA-SC-A6LR	1	0	0	0	0
Mesothelioma	TCGA-SC-AA5Z	0	0	0	0	1
Mesothelioma	TCGA-SH-A7BC	1	1	1	1	1
Mesothelioma	TCGA-SH-A7BD	1	1	1	1	1
Mesothelioma	TCGA-SH-A7BH	1	1	1	1	1
Mesothelioma	TCGA-SH-A9CT	1	1	1	1	1
Mesothelioma	TCGA-SH-A9CU	1	1	1	1	1
Mesothelioma	TCGA-TS-A7OU	1	0	1	0	1
Mesothelioma	TCGA-TS-A7OY	1	1	1	1	1
Mesothelioma	TCGA-TS-A7OZ	1	1	1	1	1
Mesothelioma	TCGA-TS-A7P0	1	1	1	1	1
Mesothelioma	TCGA-TS-A7P1	1	1	1	1	1
Mesothelioma	TCGA-TS-A7P3	1	0	1	1	1
Mesothelioma	TCGA-TS-A7P6	1	1	1	1	1
Mesothelioma	TCGA-TS-A7P7	1	1	1	1	1
Mesothelioma	TCGA-TS-A7P8	1	0	0	1	1
Mesothelioma	TCGA-TS-A7PB	1	1	0	1	1
Mesothelioma	TCGA-TS-A8AF	1	1	1	1	1
Mesothelioma	TCGA-TS-A8AI	1	1	1	1	1
Mesothelioma	TCGA-TS-A8AS	0	0	1	0	0
Mesothelioma	TCGA-TS-A8AV	0	0	0	0	0
Mesothelioma	TCGA-TS-A8AY	1	1	1	1	1
Mesothelioma	TCGA-UD-AABY	1	0	0	1	1
Mesothelioma	TCGA-UD-AABZ	1	1	1	1	1
Mesothelioma	TCGA-UD-AAC1	0	1	0	0	1
Mesothelioma	TCGA-UD-AAC4	1	1	0	1	1
Mesothelioma	TCGA-UD-AAC5	1	1	1	1	1
Mesothelioma	TCGA-UD-AAC6	1	1	1	1	1
Mesothelioma	TCGA-UD-AAC7	1	1	1	1	1
Mesothelioma	TCGA-UT-A88C	0	1	1	1	1
Mesothelioma	TCGA-UT-A88D	0	0	0	1	1
Mesothelioma	TCGA-UT-A88E	1	1	0	1	1
Mesothelioma	TCGA-UT-A88G	1	1	1	1	1
Mesothelioma	TCGA-UT-A97Y	1	1	1	1	1
Mesothelioma	TCGA-XT-AASU	1	1	1	1	1
Mesothelioma	TCGA-YS-A95B	1	1	1	1	1

Mesothelioma	TCGA-YS-A95C	1	1	0	1	1
Mesothelioma	TCGA-YS-AA4M	0	0	0	0	0
Mesothelioma	TCGA-ZN-A9VO	1	1	1	1	1
Mesothelioma	TCGA-ZN-A9VP	1	1	1	1	1
Mesothelioma	TCGA-ZN-A9VQ	1	1	1	1	1
Mesothelioma	TCGA-ZN-A9VS	1	1	1	1	1
Mesothelioma	TCGA-ZN-A9VU	1	1	1	1	1
Mesothelioma	TCGA-ZN-A9VV	1	0	0	1	1
Mesothelioma	TCGA-ZN-A9VW	1	1	0	1	1
arian Serous Cystadenocarcinc	TCGA-04-1348	1	1	1	1	1
arian Serous Cystadenocarcinc	TCGA-04-1357	1	1	1	1	1
arian Serous Cystadenocarcinc	TCGA-04-1362	1	1	1	1	1
arian Serous Cystadenocarcinc	TCGA-04-1364	1	1	1	1	1
arian Serous Cystadenocarcinc	TCGA-04-1365	1	1	1	1	1
arian Serous Cystadenocarcinc	TCGA-04-1514	1	1	1	1	1
arian Serous Cystadenocarcinc	TCGA-04-1519	1	1	1	1	1
arian Serous Cystadenocarcinc	TCGA-09-0364	1	1	1	1	1
arian Serous Cystadenocarcinc	TCGA-09-0366	1	1	1	1	1
arian Serous Cystadenocarcinc	TCGA-09-0367	1	1	1	1	1
arian Serous Cystadenocarcinc	TCGA-09-0369	1	1	1	1	1
arian Serous Cystadenocarcinc	TCGA-09-1659	1	1	1	1	1
arian Serous Cystadenocarcinc	TCGA-09-1662	1	1	1	1	1
arian Serous Cystadenocarcinc	TCGA-09-1666	1	1	1	1	1
arian Serous Cystadenocarcinc	TCGA-09-1667	1	1	1	1	1
arian Serous Cystadenocarcinc	TCGA-09-1668	1	1	1	1	1
arian Serous Cystadenocarcinc	TCGA-09-1669	1	1	1	1	1
arian Serous Cystadenocarcinc	TCGA-09-1670	1	1	1	1	1
arian Serous Cystadenocarcinc	TCGA-09-1673	1	1	1	1	1
arian Serous Cystadenocarcinc	TCGA-09-1674	1	1	1	1	1
arian Serous Cystadenocarcinc	TCGA-09-2044	1	1	1	1	1
arian Serous Cystadenocarcinc	TCGA-09-2045	1	1	1	0	1
arian Serous Cystadenocarcinc	TCGA-09-2048	1	1	1	1	1
arian Serous Cystadenocarcinc	TCGA-09-2051	1	1	1	1	1
arian Serous Cystadenocarcinc	TCGA-09-2054	1	1	1	1	1
arian Serous Cystadenocarcinc	TCGA-09-2056	1	1	1	1	1
arian Serous Cystadenocarcinc	TCGA-10-0928	1	1	1	1	1
arian Serous Cystadenocarcinc	TCGA-10-0936	1	1	1	1	1
arian Serous Cystadenocarcinc	TCGA-13-0730	1	1	1	1	1
arian Serous Cystadenocarcinc	TCGA-13-0799	1	1	1	1	1
arian Serous Cystadenocarcinc	TCGA-13-0800	1	1	1	1	1
arian Serous Cystadenocarcinc	TCGA-13-0801	1	1	1	1	1
arian Serous Cystadenocarcinc	TCGA-13-0890	1	1	1	1	1
arian Serous Cystadenocarcinc	TCGA-13-0893	1	1	1	0	1
arian Serous Cystadenocarcinc	TCGA-13-0897	1	1	1	1	1
arian Serous Cystadenocarcinc	TCGA-13-0899	1	1	1	1	1
arian Serous Cystadenocarcinc	TCGA-13-0913	1	1	1	1	1
arian Serous Cystadenocarcinc	TCGA-13-0916	1	1	1	1	1
arian Serous Cystadenocarcinc	TCGA-13-0920	1	1	1	1	1
arian Serous Cystadenocarcinc	TCGA-13-0924	1	1	1	1	1
arian Serous Cystadenocarcinc	TCGA-13-1403	1	1	1	1	1
arian Serous Cystadenocarcinc	TCGA-13-1405	1	1	1	1	1
arian Serous Cystadenocarcinc	TCGA-13-1410	1	1	1	1	1
arian Serous Cystadenocarcinc	TCGA-13-1411	1	1	1	1	1
arian Serous Cystadenocarcinc	TCGA-13-1481	1	1	1	1	1
arian Serous Cystadenocarcinc	TCGA-13-1489	0	0	0	0	0
arian Serous Cystadenocarcinc	TCGA-13-1497	1	1	1	1	1
arian Serous Cystadenocarcinc	TCGA-13-1498	1	1	1	1	1
arian Serous Cystadenocarcinc	TCGA-13-1505	1	1	1	1	1

arian Serous Cystadenocarcino	TCGA-13-1506	1	1	1	1	1
arian Serous Cystadenocarcino	TCGA-13-1507	1	1	1	1	1
arian Serous Cystadenocarcino	TCGA-13-1511	1	1	1	1	1
arian Serous Cystadenocarcino	TCGA-13-1512	1	1	1	1	1
arian Serous Cystadenocarcino	TCGA-13-2060	1	1	1	1	1
arian Serous Cystadenocarcino	TCGA-13-A5FT	1	1	1	1	1
arian Serous Cystadenocarcino	TCGA-20-1682	1	1	1	1	1
arian Serous Cystadenocarcino	TCGA-20-1683	1	1	1	1	1
arian Serous Cystadenocarcino	TCGA-20-1684	1	1	1	1	1
arian Serous Cystadenocarcino	TCGA-20-1685	1	1	1	1	1
arian Serous Cystadenocarcino	TCGA-20-1687	1	1	1	1	1
arian Serous Cystadenocarcino	TCGA-23-1023	1	1	1	1	1
arian Serous Cystadenocarcino	TCGA-23-1026	1	1	1	1	1
arian Serous Cystadenocarcino	TCGA-23-1027	1	1	1	1	1
arian Serous Cystadenocarcino	TCGA-23-1029	1	1	1	1	1
arian Serous Cystadenocarcino	TCGA-23-1109	1	1	1	1	1
arian Serous Cystadenocarcino	TCGA-23-1111	1	1	1	1	1
arian Serous Cystadenocarcino	TCGA-23-1114	1	1	1	1	1
arian Serous Cystadenocarcino	TCGA-23-1120	1	1	1	1	1
arian Serous Cystadenocarcino	TCGA-23-1122	1	1	1	1	1
arian Serous Cystadenocarcino	TCGA-23-1123	1	1	1	1	1
arian Serous Cystadenocarcino	TCGA-23-1809	1	0	1	1	1
arian Serous Cystadenocarcino	TCGA-23-2077	1	1	1	1	1
arian Serous Cystadenocarcino	TCGA-23-2078	1	1	1	1	1
arian Serous Cystadenocarcino	TCGA-23-2081	1	1	1	1	1
arian Serous Cystadenocarcino	TCGA-23-2084	1	1	1	1	1
arian Serous Cystadenocarcino	TCGA-24-0975	1	1	1	1	1
arian Serous Cystadenocarcino	TCGA-24-1103	1	1	1	1	1
arian Serous Cystadenocarcino	TCGA-24-1413	1	1	1	1	1
arian Serous Cystadenocarcino	TCGA-24-1416	1	1	1	1	1
arian Serous Cystadenocarcino	TCGA-24-1417	1	1	1	1	1
arian Serous Cystadenocarcino	TCGA-24-1418	1	0	1	1	1
arian Serous Cystadenocarcino	TCGA-24-1419	1	1	1	1	1
arian Serous Cystadenocarcino	TCGA-24-1423	1	1	1	1	1
arian Serous Cystadenocarcino	TCGA-24-1424	1	1	1	1	1
arian Serous Cystadenocarcino	TCGA-24-1425	1	1	1	1	1
arian Serous Cystadenocarcino	TCGA-24-1427	1	1	1	1	1
arian Serous Cystadenocarcino	TCGA-24-1428	1	1	1	1	1
arian Serous Cystadenocarcino	TCGA-24-1430	1	1	1	1	1
arian Serous Cystadenocarcino	TCGA-24-1434	1	1	1	1	1
arian Serous Cystadenocarcino	TCGA-24-1435	1	1	1	1	1
arian Serous Cystadenocarcino	TCGA-24-1436	1	1	1	1	1
arian Serous Cystadenocarcino	TCGA-24-1463	1	1	1	1	1
arian Serous Cystadenocarcino	TCGA-24-1467	1	1	1	1	1
arian Serous Cystadenocarcino	TCGA-24-1469	1	1	1	1	1
arian Serous Cystadenocarcino	TCGA-24-1474	1	1	1	1	1
arian Serous Cystadenocarcino	TCGA-24-1544	1	1	1	1	1
arian Serous Cystadenocarcino	TCGA-24-1545	1	1	1	1	1
arian Serous Cystadenocarcino	TCGA-24-1546	1	1	1	1	1
arian Serous Cystadenocarcino	TCGA-24-1548	1	1	1	1	1
arian Serous Cystadenocarcino	TCGA-24-1549	1	1	1	1	1
arian Serous Cystadenocarcino	TCGA-24-1550	1	1	1	1	1
arian Serous Cystadenocarcino	TCGA-24-1551	1	1	1	1	1
arian Serous Cystadenocarcino	TCGA-24-1552	1	1	1	1	1
arian Serous Cystadenocarcino	TCGA-24-1553	1	1	1	1	1
arian Serous Cystadenocarcino	TCGA-24-1555	1	1	1	1	1
arian Serous Cystadenocarcino	TCGA-24-1556	1	1	1	1	1
arian Serous Cystadenocarcino	TCGA-24-1557	1	1	1	1	1

ırian Serous Cystadenocarcinc	TCGA-24-1558	1	1	1	1	1
ırian Serous Cystadenocarcinc	TCGA-24-1560	1	1	1	1	1
ırian Serous Cystadenocarcinc	TCGA-24-1562	1	1	1	1	1
ırian Serous Cystadenocarcinc	TCGA-24-1563	1	1	1	1	1
ırian Serous Cystadenocarcinc	TCGA-24-1564	1	1	1	1	1
ırian Serous Cystadenocarcinc	TCGA-24-1567	1	1	1	1	1
ırian Serous Cystadenocarcinc	TCGA-24-1603	1	1	1	1	1
ırian Serous Cystadenocarcinc	TCGA-24-1604	1	1	1	1	1
ırian Serous Cystadenocarcinc	TCGA-24-1616	1	1	1	1	1
ırian Serous Cystadenocarcinc	TCGA-24-1842	1	1	1	1	1
ırian Serous Cystadenocarcinc	TCGA-24-1843	1	1	1	1	1
ırian Serous Cystadenocarcinc	TCGA-24-1844	1	1	1	1	1
ırian Serous Cystadenocarcinc	TCGA-24-1846	1	1	1	1	1
ırian Serous Cystadenocarcinc	TCGA-24-1847	1	1	1	1	1
ırian Serous Cystadenocarcinc	TCGA-24-1849	1	1	1	1	1
ırian Serous Cystadenocarcinc	TCGA-24-1850	1	1	1	1	1
ırian Serous Cystadenocarcinc	TCGA-24-1924	1	1	1	1	1
ırian Serous Cystadenocarcinc	TCGA-24-1928	1	1	1	1	1
ırian Serous Cystadenocarcinc	TCGA-24-1930	1	1	1	1	1
ırian Serous Cystadenocarcinc	TCGA-24-2019	1	1	1	1	1
ırian Serous Cystadenocarcinc	TCGA-24-2020	1	1	1	1	1
ırian Serous Cystadenocarcinc	TCGA-24-2023	1	1	1	1	1
ırian Serous Cystadenocarcinc	TCGA-24-2024	1	1	1	1	1
ırian Serous Cystadenocarcinc	TCGA-24-2026	1	1	1	1	1
ırian Serous Cystadenocarcinc	TCGA-24-2027	1	1	1	1	1
ırian Serous Cystadenocarcinc	TCGA-24-2033	1	1	1	1	1
ırian Serous Cystadenocarcinc	TCGA-24-2035	1	1	1	1	1
ırian Serous Cystadenocarcinc	TCGA-24-2036	1	1	1	1	1
ırian Serous Cystadenocarcinc	TCGA-24-2038	0	0	0	0	0
ırian Serous Cystadenocarcinc	TCGA-24-2254	1	1	1	1	1
ırian Serous Cystadenocarcinc	TCGA-24-2261	1	1	1	1	1
ırian Serous Cystadenocarcinc	TCGA-24-2262	1	1	1	1	1
ırian Serous Cystadenocarcinc	TCGA-24-2267	1	1	1	1	1
ırian Serous Cystadenocarcinc	TCGA-24-2271	1	1	1	1	1
ırian Serous Cystadenocarcinc	TCGA-24-2281	1	1	1	1	1
ırian Serous Cystadenocarcinc	TCGA-24-2288	1	1	1	1	1
ırian Serous Cystadenocarcinc	TCGA-24-2289	1	1	1	1	1
ırian Serous Cystadenocarcinc	TCGA-24-2290	1	1	1	1	1
ırian Serous Cystadenocarcinc	TCGA-24-2293	1	1	1	1	1
ırian Serous Cystadenocarcinc	TCGA-24-2297	1	1	1	1	1
ırian Serous Cystadenocarcinc	TCGA-24-2298	1	1	1	1	1
ırian Serous Cystadenocarcinc	TCGA-25-1312	1	1	1	1	1
ırian Serous Cystadenocarcinc	TCGA-25-1313	1	1	1	1	1
ırian Serous Cystadenocarcinc	TCGA-25-1314	1	1	1	1	1
ırian Serous Cystadenocarcinc	TCGA-25-1315	1	1	1	1	1
ırian Serous Cystadenocarcinc	TCGA-25-1316	1	1	1	1	1
ırian Serous Cystadenocarcinc	TCGA-25-1317	1	1	1	1	1
ırian Serous Cystadenocarcinc	TCGA-25-1318	1	1	1	1	1
ırian Serous Cystadenocarcinc	TCGA-25-1319	1	1	1	1	1
ırian Serous Cystadenocarcinc	TCGA-25-1320	1	1	1	1	1
ırian Serous Cystadenocarcinc	TCGA-25-1321	1	1	1	1	1
ırian Serous Cystadenocarcinc	TCGA-25-1322	1	1	1	1	1
ırian Serous Cystadenocarcinc	TCGA-25-1323	1	1	1	1	1
ırian Serous Cystadenocarcinc	TCGA-25-1324	0	0	0	0	0
ırian Serous Cystadenocarcinc	TCGA-25-1326	0	0	0	0	0
ırian Serous Cystadenocarcinc	TCGA-25-1328	0	0	0	0	0
ırian Serous Cystadenocarcinc	TCGA-25-1329	1	1	1	1	1
ırian Serous Cystadenocarcinc	TCGA-25-1623	1	0	1	1	1

arian Serous Cystadenocarcinc	TCGA-25-1625	1	1	1	1	1
arian Serous Cystadenocarcinc	TCGA-25-1626	1	1	1	1	1
arian Serous Cystadenocarcinc	TCGA-25-1627	1	1	1	1	1
arian Serous Cystadenocarcinc	TCGA-25-1628	1	1	1	1	1
arian Serous Cystadenocarcinc	TCGA-25-1630	1	1	1	1	1
arian Serous Cystadenocarcinc	TCGA-25-1631	1	1	1	1	1
arian Serous Cystadenocarcinc	TCGA-25-1632	1	1	1	1	1
arian Serous Cystadenocarcinc	TCGA-25-1633	1	1	1	1	1
arian Serous Cystadenocarcinc	TCGA-25-1634	1	1	1	1	1
arian Serous Cystadenocarcinc	TCGA-25-1635	1	1	1	1	1
arian Serous Cystadenocarcinc	TCGA-25-1870	1	1	1	1	1
arian Serous Cystadenocarcinc	TCGA-25-1871	1	1	1	1	1
arian Serous Cystadenocarcinc	TCGA-25-2042	1	0	1	1	1
arian Serous Cystadenocarcinc	TCGA-25-2391	1	1	1	1	1
arian Serous Cystadenocarcinc	TCGA-25-2392	1	1	1	1	1
arian Serous Cystadenocarcinc	TCGA-25-2393	1	1	1	1	1
arian Serous Cystadenocarcinc	TCGA-25-2396	1	1	1	1	1
arian Serous Cystadenocarcinc	TCGA-25-2397	1	1	1	1	1
arian Serous Cystadenocarcinc	TCGA-25-2398	1	1	1	1	1
arian Serous Cystadenocarcinc	TCGA-25-2399	1	1	1	1	1
arian Serous Cystadenocarcinc	TCGA-25-2400	1	1	1	1	1
arian Serous Cystadenocarcinc	TCGA-25-2401	1	1	1	1	1
arian Serous Cystadenocarcinc	TCGA-25-2404	1	1	1	1	1
arian Serous Cystadenocarcinc	TCGA-25-2409	1	1	1	1	1
arian Serous Cystadenocarcinc	TCGA-29-1688	1	1	1	1	1
arian Serous Cystadenocarcinc	TCGA-29-1690	1	1	0	1	0
arian Serous Cystadenocarcinc	TCGA-29-1691	1	1	1	1	1
arian Serous Cystadenocarcinc	TCGA-29-1693	1	1	1	1	1
arian Serous Cystadenocarcinc	TCGA-29-1694	1	1	1	1	1
arian Serous Cystadenocarcinc	TCGA-29-1695	1	1	1	1	1
arian Serous Cystadenocarcinc	TCGA-29-1696	1	1	1	1	1
arian Serous Cystadenocarcinc	TCGA-29-1697	1	1	1	1	1
arian Serous Cystadenocarcinc	TCGA-29-1698	1	1	1	1	1
arian Serous Cystadenocarcinc	TCGA-29-1699	1	1	1	1	1
arian Serous Cystadenocarcinc	TCGA-29-1701	1	1	1	1	1
arian Serous Cystadenocarcinc	TCGA-29-1702	1	1	1	1	1
arian Serous Cystadenocarcinc	TCGA-29-1703	1	1	1	1	1
arian Serous Cystadenocarcinc	TCGA-29-1705	1	1	1	1	1
arian Serous Cystadenocarcinc	TCGA-29-1710	1	1	1	1	1
arian Serous Cystadenocarcinc	TCGA-29-1711	1	1	1	1	1
arian Serous Cystadenocarcinc	TCGA-29-1761	1	1	1	1	1
arian Serous Cystadenocarcinc	TCGA-29-1762	1	1	1	1	1
arian Serous Cystadenocarcinc	TCGA-29-1763	1	1	1	1	1
arian Serous Cystadenocarcinc	TCGA-29-1770	1	1	1	1	1
arian Serous Cystadenocarcinc	TCGA-29-1770	0	0	0	0	0
arian Serous Cystadenocarcinc	TCGA-29-1776	1	1	1	1	1
arian Serous Cystadenocarcinc	TCGA-29-1778	1	1	1	1	1
arian Serous Cystadenocarcinc	TCGA-29-1781	1	1	1	1	1
arian Serous Cystadenocarcinc	TCGA-29-1783	1	1	1	1	1
arian Serous Cystadenocarcinc	TCGA-29-1784	1	1	1	1	1
arian Serous Cystadenocarcinc	TCGA-29-1785	1	1	1	1	1
arian Serous Cystadenocarcinc	TCGA-29-2414	1	1	1	1	1
arian Serous Cystadenocarcinc	TCGA-29-2414	0	0	0	0	0
arian Serous Cystadenocarcinc	TCGA-29-2425	1	1	1	1	1
arian Serous Cystadenocarcinc	TCGA-29-2427	1	1	1	1	1
arian Serous Cystadenocarcinc	TCGA-29-2428	1	1	1	1	1
arian Serous Cystadenocarcinc	TCGA-29-A5NZ	1	0	1	1	1
arian Serous Cystadenocarcinc	TCGA-30-1718	1	1	1	1	1

arian Serous Cystadenocarcinc	TCGA-30-1855	1	1	1	1	1
arian Serous Cystadenocarcinc	TCGA-30-1857	1	1	1	1	1
arian Serous Cystadenocarcinc	TCGA-30-1860	1	1	1	1	1
arian Serous Cystadenocarcinc	TCGA-30-1861	1	1	1	1	1
arian Serous Cystadenocarcinc	TCGA-30-1862	1	1	1	1	1
arian Serous Cystadenocarcinc	TCGA-30-1891	1	1	1	1	1
arian Serous Cystadenocarcinc	TCGA-31-1944	1	1	1	1	1
arian Serous Cystadenocarcinc	TCGA-31-1946	1	1	1	1	1
arian Serous Cystadenocarcinc	TCGA-31-1951	1	1	1	1	1
arian Serous Cystadenocarcinc	TCGA-31-1953	1	1	1	1	1
arian Serous Cystadenocarcinc	TCGA-31-1956	1	1	1	1	1
arian Serous Cystadenocarcinc	TCGA-31-1959	1	1	1	1	1
arian Serous Cystadenocarcinc	TCGA-36-1568	1	1	1	1	1
arian Serous Cystadenocarcinc	TCGA-36-1569	1	1	1	1	1
arian Serous Cystadenocarcinc	TCGA-36-1570	1	1	1	1	1
arian Serous Cystadenocarcinc	TCGA-36-1571	1	1	1	1	1
arian Serous Cystadenocarcinc	TCGA-36-1574	1	1	1	1	1
arian Serous Cystadenocarcinc	TCGA-36-1575	1	1	1	1	1
arian Serous Cystadenocarcinc	TCGA-36-1576	1	1	1	1	1
arian Serous Cystadenocarcinc	TCGA-36-1577	1	1	1	1	1
arian Serous Cystadenocarcinc	TCGA-36-1578	1	1	1	1	1
arian Serous Cystadenocarcinc	TCGA-36-1580	1	1	1	1	1
arian Serous Cystadenocarcinc	TCGA-36-1581	1	1	1	1	1
arian Serous Cystadenocarcinc	TCGA-3P-A9WA	1	1	1	0	1
arian Serous Cystadenocarcinc	TCGA-57-1582	1	1	1	1	1
arian Serous Cystadenocarcinc	TCGA-57-1583	1	1	1	1	1
arian Serous Cystadenocarcinc	TCGA-57-1584	1	1	1	1	1
arian Serous Cystadenocarcinc	TCGA-57-1585	1	1	1	1	1
arian Serous Cystadenocarcinc	TCGA-57-1993	1	1	1	1	1
arian Serous Cystadenocarcinc	TCGA-57-1994	1	1	1	1	1
arian Serous Cystadenocarcinc	TCGA-59-2348	1	1	1	1	1
arian Serous Cystadenocarcinc	TCGA-59-2350	1	1	1	1	1
arian Serous Cystadenocarcinc	TCGA-59-2351	1	1	1	1	1
arian Serous Cystadenocarcinc	TCGA-59-2352	1	1	1	1	1
arian Serous Cystadenocarcinc	TCGA-59-2354	1	1	1	1	1
arian Serous Cystadenocarcinc	TCGA-59-2355	1	1	1	1	1
arian Serous Cystadenocarcinc	TCGA-59-2363	1	1	1	1	1
arian Serous Cystadenocarcinc	TCGA-59-A5PD	1	1	1	1	1
arian Serous Cystadenocarcinc	TCGA-5X-AA5U	1	1	1	1	1
arian Serous Cystadenocarcinc	TCGA-61-1721	0	0	0	0	0
arian Serous Cystadenocarcinc	TCGA-61-1724	1	1	1	1	1
arian Serous Cystadenocarcinc	TCGA-61-1725	1	1	1	1	1
arian Serous Cystadenocarcinc	TCGA-61-1728	1	1	1	1	1
arian Serous Cystadenocarcinc	TCGA-61-1733	1	1	1	1	1
arian Serous Cystadenocarcinc	TCGA-61-1736	1	1	1	1	1
arian Serous Cystadenocarcinc	TCGA-61-1737	1	1	1	1	1
arian Serous Cystadenocarcinc	TCGA-61-1738	1	1	1	1	1
arian Serous Cystadenocarcinc	TCGA-61-1740	1	1	1	1	1
arian Serous Cystadenocarcinc	TCGA-61-1741	1	1	1	1	1
arian Serous Cystadenocarcinc	TCGA-61-1743	1	1	1	1	1
arian Serous Cystadenocarcinc	TCGA-61-1900	1	1	1	1	1
arian Serous Cystadenocarcinc	TCGA-61-1907	1	1	1	1	1
arian Serous Cystadenocarcinc	TCGA-61-1910	1	1	1	1	1
arian Serous Cystadenocarcinc	TCGA-61-1914	1	1	1	1	1
arian Serous Cystadenocarcinc	TCGA-61-1917	1	1	1	1	1
arian Serous Cystadenocarcinc	TCGA-61-1918	1	1	1	1	1
arian Serous Cystadenocarcinc	TCGA-61-1919	1	1	1	1	1
arian Serous Cystadenocarcinc	TCGA-61-1995	1	1	1	1	1

arian Serous Cystadenocarcino	TCGA-61-1998	1	1	1	1	1
arian Serous Cystadenocarcino	TCGA-61-2000	1	1	1	1	1
arian Serous Cystadenocarcino	TCGA-61-2002	1	1	1	1	1
arian Serous Cystadenocarcino	TCGA-61-2003	1	1	1	1	1
arian Serous Cystadenocarcino	TCGA-61-2008	1	1	1	1	1
arian Serous Cystadenocarcino	TCGA-61-2008	0	0	0	0	0
arian Serous Cystadenocarcino	TCGA-61-2009	1	1	1	1	1
arian Serous Cystadenocarcino	TCGA-61-2012	1	1	1	1	1
arian Serous Cystadenocarcino	TCGA-61-2016	1	1	1	1	1
arian Serous Cystadenocarcino	TCGA-61-2088	1	1	1	1	1
arian Serous Cystadenocarcino	TCGA-61-2092	1	1	1	1	1
arian Serous Cystadenocarcino	TCGA-61-2094	1	1	1	1	1
arian Serous Cystadenocarcino	TCGA-61-2095	0	1	1	1	1
arian Serous Cystadenocarcino	TCGA-61-2097	1	1	1	1	1
arian Serous Cystadenocarcino	TCGA-61-2098	1	1	1	1	1
arian Serous Cystadenocarcino	TCGA-61-2101	1	1	1	1	1
arian Serous Cystadenocarcino	TCGA-61-2102	1	1	1	1	1
arian Serous Cystadenocarcino	TCGA-61-2104	1	1	1	1	1
arian Serous Cystadenocarcino	TCGA-61-2109	1	1	1	1	1
arian Serous Cystadenocarcino	TCGA-61-2110	1	1	1	1	1
arian Serous Cystadenocarcino	TCGA-61-2111	1	1	1	1	1
arian Serous Cystadenocarcino	TCGA-61-2113	1	1	1	1	1
arian Serous Cystadenocarcino	TCGA-OY-A56P	1	1	1	1	1
arian Serous Cystadenocarcino	TCGA-OY-A56Q	1	1	1	1	1
arian Serous Cystadenocarcino	TCGA-VG-A8LO	1	1	1	1	1
arian Serous Cystadenocarcino	TCGA-WR-A838	1	1	1	1	1
Pancreatic Adenocarcinoma	TCGA-2J-AAB1	1	1	0	0	1
Pancreatic Adenocarcinoma	TCGA-2J-AAB4	1	1	1	0	1
Pancreatic Adenocarcinoma	TCGA-2J-AAB6	1	1	0	0	1
Pancreatic Adenocarcinoma	TCGA-2J-AAB8	1	1	0	1	1
Pancreatic Adenocarcinoma	TCGA-2J-AAB9	0	0	0	0	1
Pancreatic Adenocarcinoma	TCGA-2J-AABA	1	1	1	1	1
Pancreatic Adenocarcinoma	TCGA-2J-AABE	1	1	1	1	1
Pancreatic Adenocarcinoma	TCGA-2J-AABF	1	1	0	1	1
Pancreatic Adenocarcinoma	TCGA-2J-AABH	1	1	1	1	1
Pancreatic Adenocarcinoma	TCGA-2J-AABI	1	1	1	1	1
Pancreatic Adenocarcinoma	TCGA-2J-AABK	1	1	1	1	1
Pancreatic Adenocarcinoma	TCGA-2J-AABO	1	1	1	1	1
Pancreatic Adenocarcinoma	TCGA-2J-AABP	1	1	1	1	1
Pancreatic Adenocarcinoma	TCGA-2J-AABR	1	0	0	0	1
Pancreatic Adenocarcinoma	TCGA-2J-AABT	1	1	1	1	1
Pancreatic Adenocarcinoma	TCGA-2J-AABU	1	1	1	1	1
Pancreatic Adenocarcinoma	TCGA-2J-AABV	1	1	0	0	1
Pancreatic Adenocarcinoma	TCGA-2L-AAQA	1	1	1	1	1
Pancreatic Adenocarcinoma	TCGA-2L-AAQE	1	1	1	1	1
Pancreatic Adenocarcinoma	TCGA-2L-AAQI	1	1	1	1	1
Pancreatic Adenocarcinoma	TCGA-2L-AAQJ	1	1	1	1	1
Pancreatic Adenocarcinoma	TCGA-2L-AAQL	1	1	1	1	1
Pancreatic Adenocarcinoma	TCGA-2L-AAQM	1	1	1	1	1
Pancreatic Adenocarcinoma	TCGA-3A-A9I5	1	1	0	1	1
Pancreatic Adenocarcinoma	TCGA-3A-A9I7	1	1	0	0	1
Pancreatic Adenocarcinoma	TCGA-3A-A9I9	1	1	1	1	1
Pancreatic Adenocarcinoma	TCGA-3A-A9IB	1	1	1	1	1
Pancreatic Adenocarcinoma	TCGA-3A-A9IC	1	1	1	1	1
Pancreatic Adenocarcinoma	TCGA-3A-A9IH	1	1	1	1	1
Pancreatic Adenocarcinoma	TCGA-3A-A9IJ	0	0	0	0	0
Pancreatic Adenocarcinoma	TCGA-3A-A9IL	1	1	1	1	1
Pancreatic Adenocarcinoma	TCGA-3A-A9IN	0	0	1	0	1

Pancreatic Adenocarcinoma	TCGA-3A-A9IO	1	0	0	1	0
Pancreatic Adenocarcinoma	TCGA-3A-A9IR	0	1	0	0	0
Pancreatic Adenocarcinoma	TCGA-3A-A9IS	1	1	1	1	1
Pancreatic Adenocarcinoma	TCGA-3A-A9IU	1	1	1	0	1
Pancreatic Adenocarcinoma	TCGA-3A-A9IV	0	0	0	0	1
Pancreatic Adenocarcinoma	TCGA-3A-A9IX	1	1	1	1	1
Pancreatic Adenocarcinoma	TCGA-3A-A9IZ	1	1	1	0	1
Pancreatic Adenocarcinoma	TCGA-3A-A9J0	1	1	1	1	1
Pancreatic Adenocarcinoma	TCGA-3E-AAAY	0	1	0	0	1
Pancreatic Adenocarcinoma	TCGA-3E-AAAZ	1	1	1	1	1
Pancreatic Adenocarcinoma	TCGA-F2-6879	1	1	1	1	1
Pancreatic Adenocarcinoma	TCGA-F2-6880	0	0	0	0	0
Pancreatic Adenocarcinoma	TCGA-F2-7273	0	0	0	0	0
Pancreatic Adenocarcinoma	TCGA-F2-7276	0	0	0	0	0
Pancreatic Adenocarcinoma	TCGA-F2-A44G	1	1	1	0	1
Pancreatic Adenocarcinoma	TCGA-F2-A44H	1	1	1	1	1
Pancreatic Adenocarcinoma	TCGA-F2-A7TX	1	0	1	0	1
Pancreatic Adenocarcinoma	TCGA-F2-A8YN	1	1	1	1	1
Pancreatic Adenocarcinoma	TCGA-FB-A4P5	0	0	0	0	0
Pancreatic Adenocarcinoma	TCGA-FB-A4P6	0	0	0	0	0
Pancreatic Adenocarcinoma	TCGA-FB-A545	1	1	1	1	1
Pancreatic Adenocarcinoma	TCGA-FB-A5VM	1	0	1	1	1
Pancreatic Adenocarcinoma	TCGA-FB-A78T	1	1	1	1	1
Pancreatic Adenocarcinoma	TCGA-FB-A7DR	1	1	0	1	1
Pancreatic Adenocarcinoma	TCGA-FB-AAPP	1	1	1	1	1
Pancreatic Adenocarcinoma	TCGA-FB-AAPQ	1	1	1	1	1
Pancreatic Adenocarcinoma	TCGA-FB-AAPS	1	1	1	0	1
Pancreatic Adenocarcinoma	TCGA-FB-AAPU	1	1	1	1	1
Pancreatic Adenocarcinoma	TCGA-FB-AAPY	1	1	1	1	1
Pancreatic Adenocarcinoma	TCGA-FB-AAPZ	1	1	1	1	1
Pancreatic Adenocarcinoma	TCGA-FB-AAQ0	1	1	1	1	1
Pancreatic Adenocarcinoma	TCGA-FB-AAQ1	1	1	1	1	1
Pancreatic Adenocarcinoma	TCGA-FB-AAQ2	1	1	1	1	1
Pancreatic Adenocarcinoma	TCGA-FB-AAQ3	1	1	1	1	1
Pancreatic Adenocarcinoma	TCGA-FB-AAQ6	1	1	1	1	1
Pancreatic Adenocarcinoma	TCGA-H6-8124	1	1	1	1	1
Pancreatic Adenocarcinoma	TCGA-H6-A45N	1	0	0	0	1
Pancreatic Adenocarcinoma	TCGA-H8-A6C1	1	1	1	1	1
Pancreatic Adenocarcinoma	TCGA-HV-A5A3	1	1	1	1	1
Pancreatic Adenocarcinoma	TCGA-HV-A5A4	1	1	1	1	1
Pancreatic Adenocarcinoma	TCGA-HV-A5A5	1	1	1	1	1
Pancreatic Adenocarcinoma	TCGA-HV-A5A6	1	1	1	1	1
Pancreatic Adenocarcinoma	TCGA-HV-A7OL	1	0	1	1	1
Pancreatic Adenocarcinoma	TCGA-HV-A7OP	1	1	1	1	1
Pancreatic Adenocarcinoma	TCGA-HV-AA8V	1	0	1	0	1
Pancreatic Adenocarcinoma	TCGA-HV-AA8X	1	1	1	1	1
Pancreatic Adenocarcinoma	TCGA-HZ-7289	1	1	1	1	1
Pancreatic Adenocarcinoma	TCGA-HZ-7918	1	1	1	1	1
Pancreatic Adenocarcinoma	TCGA-HZ-7919	1	1	1	1	1
Pancreatic Adenocarcinoma	TCGA-HZ-7920	0	0	0	0	0
Pancreatic Adenocarcinoma	TCGA-HZ-7922	1	1	1	1	1
Pancreatic Adenocarcinoma	TCGA-HZ-7923	0	0	0	0	0
Pancreatic Adenocarcinoma	TCGA-HZ-7924	0	0	0	0	0
Pancreatic Adenocarcinoma	TCGA-HZ-7925	1	1	1	1	1
Pancreatic Adenocarcinoma	TCGA-HZ-7926	1	0	0	0	1
Pancreatic Adenocarcinoma	TCGA-HZ-8001	1	0	1	0	1
Pancreatic Adenocarcinoma	TCGA-HZ-8002	0	0	0	0	0
Pancreatic Adenocarcinoma	TCGA-HZ-8003	0	0	0	0	1

Pancreatic Adenocarcinoma	TCGA-HZ-8005	1	1	1	1	1
Pancreatic Adenocarcinoma	TCGA-HZ-8315	1	0	1	0	1
Pancreatic Adenocarcinoma	TCGA-HZ-8317	0	0	0	0	0
Pancreatic Adenocarcinoma	TCGA-HZ-8519	0	0	0	0	1
Pancreatic Adenocarcinoma	TCGA-HZ-8636	1	1	1	1	1
Pancreatic Adenocarcinoma	TCGA-HZ-8637	1	1	1	1	1
Pancreatic Adenocarcinoma	TCGA-HZ-8638	0	0	0	0	0
Pancreatic Adenocarcinoma	TCGA-HZ-A49G	1	1	0	0	1
Pancreatic Adenocarcinoma	TCGA-HZ-A49H	0	0	0	0	0
Pancreatic Adenocarcinoma	TCGA-HZ-A49I	1	1	1	0	1
Pancreatic Adenocarcinoma	TCGA-HZ-A4BH	1	1	1	1	1
Pancreatic Adenocarcinoma	TCGA-HZ-A4BK	1	1	1	1	1
Pancreatic Adenocarcinoma	TCGA-HZ-A77O	1	1	1	1	1
Pancreatic Adenocarcinoma	TCGA-HZ-A77P	1	1	1	0	1
Pancreatic Adenocarcinoma	TCGA-HZ-A77Q	1	0	1	1	1
Pancreatic Adenocarcinoma	TCGA-HZ-A8P0	1	1	1	0	1
Pancreatic Adenocarcinoma	TCGA-HZ-A8P1	1	1	0	1	1
Pancreatic Adenocarcinoma	TCGA-HZ-A9TJ	1	1	1	1	1
Pancreatic Adenocarcinoma	TCGA-HZ-A9TJ	0	0	0	0	0
Pancreatic Adenocarcinoma	TCGA-IB-7644	1	1	1	1	1
Pancreatic Adenocarcinoma	TCGA-IB-7645	1	1	1	0	1
Pancreatic Adenocarcinoma	TCGA-IB-7646	1	1	1	1	1
Pancreatic Adenocarcinoma	TCGA-IB-7647	1	1	1	1	1
Pancreatic Adenocarcinoma	TCGA-IB-7649	1	1	0	1	1
Pancreatic Adenocarcinoma	TCGA-IB-7651	1	1	1	1	1
Pancreatic Adenocarcinoma	TCGA-IB-7652	1	1	1	1	1
Pancreatic Adenocarcinoma	TCGA-IB-7654	1	1	1	1	1
Pancreatic Adenocarcinoma	TCGA-IB-7885	1	1	1	0	1
Pancreatic Adenocarcinoma	TCGA-IB-7886	1	1	1	1	1
Pancreatic Adenocarcinoma	TCGA-IB-7887	1	1	1	1	1
Pancreatic Adenocarcinoma	TCGA-IB-7888	1	1	1	0	1
Pancreatic Adenocarcinoma	TCGA-IB-7889	1	1	1	1	1
Pancreatic Adenocarcinoma	TCGA-IB-7890	1	1	1	1	1
Pancreatic Adenocarcinoma	TCGA-IB-7891	1	0	1	0	1
Pancreatic Adenocarcinoma	TCGA-IB-7893	1	0	1	1	1
Pancreatic Adenocarcinoma	TCGA-IB-7897	0	1	0	0	0
Pancreatic Adenocarcinoma	TCGA-IB-8126	1	1	0	0	0
Pancreatic Adenocarcinoma	TCGA-IB-8127	1	1	1	1	1
Pancreatic Adenocarcinoma	TCGA-IB-A5SO	1	0	0	0	1
Pancreatic Adenocarcinoma	TCGA-IB-A5SP	1	1	1	1	1
Pancreatic Adenocarcinoma	TCGA-IB-A5SQ	1	0	0	1	0
Pancreatic Adenocarcinoma	TCGA-IB-A5SS	1	1	1	1	1
Pancreatic Adenocarcinoma	TCGA-IB-A5ST	0	0	0	0	1
Pancreatic Adenocarcinoma	TCGA-IB-A6UF	1	1	1	1	1
Pancreatic Adenocarcinoma	TCGA-IB-A6UG	1	1	1	1	1
Pancreatic Adenocarcinoma	TCGA-IB-A7LX	1	1	1	1	1
Pancreatic Adenocarcinoma	TCGA-IB-A7M4	1	1	1	1	1
Pancreatic Adenocarcinoma	TCGA-IB-AAUM	1	0	0	0	0
Pancreatic Adenocarcinoma	TCGA-IB-AAUN	1	1	1	1	1
Pancreatic Adenocarcinoma	TCGA-IB-AAUO	1	1	1	1	1
Pancreatic Adenocarcinoma	TCGA-IB-AAUP	1	0	0	0	1
Pancreatic Adenocarcinoma	TCGA-IB-AAUQ	1	1	1	1	1
Pancreatic Adenocarcinoma	TCGA-IB-AAUR	0	0	0	0	0
Pancreatic Adenocarcinoma	TCGA-IB-AAUS	1	0	0	0	0
Pancreatic Adenocarcinoma	TCGA-IB-AAUT	0	0	0	0	0
Pancreatic Adenocarcinoma	TCGA-IB-AAUU	1	1	1	1	1
Pancreatic Adenocarcinoma	TCGA-IB-AAUV	0	0	0	0	0
Pancreatic Adenocarcinoma	TCGA-IB-AAUW	0	0	0	0	0

Pancreatic Adenocarcinoma	TCGA-L1-A7W4	1	1	1	1	1
Pancreatic Adenocarcinoma	TCGA-LB-A7SX	1	1	1	1	1
Pancreatic Adenocarcinoma	TCGA-LB-A8F3	1	1	0	1	1
Pancreatic Adenocarcinoma	TCGA-LB-A9Q5	0	0	0	0	1
Pancreatic Adenocarcinoma	TCGA-M8-A5N4	1	1	1	1	1
Pancreatic Adenocarcinoma	TCGA-OE-A75W	1	1	1	1	1
Pancreatic Adenocarcinoma	TCGA-PZ-A5RE	1	1	1	1	1
Pancreatic Adenocarcinoma	TCGA-Q3-A5QY	0	0	0	0	0
Pancreatic Adenocarcinoma	TCGA-Q3-AA2A	1	1	1	1	1
Pancreatic Adenocarcinoma	TCGA-RB-A7B8	1	1	0	1	1
Pancreatic Adenocarcinoma	TCGA-RB-AA9M	1	1	0	1	1
Pancreatic Adenocarcinoma	TCGA-RL-AAAS	0	0	0	0	0
Pancreatic Adenocarcinoma	TCGA-S4-A8RM	1	1	1	1	1
Pancreatic Adenocarcinoma	TCGA-S4-A8RO	1	1	1	1	1
Pancreatic Adenocarcinoma	TCGA-S4-A8RP	1	1	1	1	1
Pancreatic Adenocarcinoma	TCGA-US-A774	1	0	0	0	1
Pancreatic Adenocarcinoma	TCGA-US-A776	1	1	1	1	1
Pancreatic Adenocarcinoma	TCGA-US-A779	1	1	1	1	1
Pancreatic Adenocarcinoma	TCGA-US-A77E	0	0	0	1	1
Pancreatic Adenocarcinoma	TCGA-US-A77G	1	1	1	0	1
Pancreatic Adenocarcinoma	TCGA-US-A77J	0	0	0	0	0
Pancreatic Adenocarcinoma	TCGA-XD-AAUG	1	0	0	0	1
Pancreatic Adenocarcinoma	TCGA-XD-AAUH	0	0	0	0	1
Pancreatic Adenocarcinoma	TCGA-XD-AAUI	1	1	1	1	1
Pancreatic Adenocarcinoma	TCGA-XD-AAUL	1	1	1	1	1
Pancreatic Adenocarcinoma	TCGA-XN-A8T3	1	1	1	1	1
Pancreatic Adenocarcinoma	TCGA-XN-A8T5	0	0	0	0	1
Pancreatic Adenocarcinoma	TCGA-YB-A89D	1	0	1	1	1
Pancreatic Adenocarcinoma	TCGA-YH-A8SY	1	1	1	1	1
Pancreatic Adenocarcinoma	TCGA-YY-A8LH	1	1	1	1	1
Pancreatic Adenocarcinoma	TCGA-Z5-AAPL	0	0	0	0	1
ochromocytoma and Paragangl	TCGA-P7-A5NX	0	0	0	0	0
ochromocytoma and Paragangl	TCGA-P7-A5NY	0	0	0	0	0
ochromocytoma and Paragangl	TCGA-P7-A5NY	0	0	0	0	0
ochromocytoma and Paragangl	TCGA-P8-A5KC	0	0	0	0	0
ochromocytoma and Paragangl	TCGA-P8-A5KD	0	1	1	1	1
ochromocytoma and Paragangl	TCGA-P8-A6RX	1	0	0	1	1
ochromocytoma and Paragangl	TCGA-P8-A6RY	0	0	0	1	1
ochromocytoma and Paragangl	TCGA-PR-A5PF	1	1	1	1	1
ochromocytoma and Paragangl	TCGA-PR-A5PG	0	1	1	1	1
ochromocytoma and Paragangl	TCGA-PR-A5PH	1	1	0	1	0
ochromocytoma and Paragangl	TCGA-QR-A6GO	1	1	1	1	1
ochromocytoma and Paragangl	TCGA-QR-A6GR	0	0	1	1	1
ochromocytoma and Paragangl	TCGA-QR-A6GS	0	0	1	1	1
ochromocytoma and Paragangl	TCGA-QR-A6GT	1	1	1	1	1
ochromocytoma and Paragangl	TCGA-QR-A6GU	1	0	1	1	1
ochromocytoma and Paragangl	TCGA-QR-A6GW	0	0	0	0	0
ochromocytoma and Paragangl	TCGA-QR-A6GX	1	1	0	1	1
ochromocytoma and Paragangl	TCGA-QR-A6GY	1	0	1	1	1
ochromocytoma and Paragangl	TCGA-QR-A6GZ	1	0	0	1	1
ochromocytoma and Paragangl	TCGA-QR-A6GZ	0	0	0	0	0
ochromocytoma and Paragangl	TCGA-QR-A6H0	1	1	0	1	0
ochromocytoma and Paragangl	TCGA-QR-A6H1	1	1	1	1	1
ochromocytoma and Paragangl	TCGA-QR-A6H2	1	0	1	1	1
ochromocytoma and Paragangl	TCGA-QR-A6H3	1	1	0	1	0
ochromocytoma and Paragangl	TCGA-QR-A6H4	0	0	0	0	0
ochromocytoma and Paragangl	TCGA-QR-A6H5	1	0	0	1	1
ochromocytoma and Paragangl	TCGA-QR-A6H6	1	1	1	1	1

›chromocytoma and Paragangl	TCGA-QR-A6ZZ	0	0	0	0	0
›chromocytoma and Paragangl	TCGA-QR-A700	1	1	1	1	1
›chromocytoma and Paragangl	TCGA-QR-A702	1	0	1	1	1
›chromocytoma and Paragangl	TCGA-QR-A703	0	0	0	0	0
›chromocytoma and Paragangl	TCGA-QR-A705	0	0	0	0	0
›chromocytoma and Paragangl	TCGA-QR-A706	0	1	0	1	0
›chromocytoma and Paragangl	TCGA-QR-A707	0	0	0	0	0
›chromocytoma and Paragangl	TCGA-QR-A708	0	0	0	0	0
›chromocytoma and Paragangl	TCGA-QR-A70A	0	0	0	0	0
›chromocytoma and Paragangl	TCGA-QR-A70C	0	0	0	1	1
›chromocytoma and Paragangl	TCGA-QR-A70D	1	1	0	1	0
›chromocytoma and Paragangl	TCGA-QR-A70E	1	1	0	1	1
›chromocytoma and Paragangl	TCGA-QR-A70G	0	0	0	1	1
›chromocytoma and Paragangl	TCGA-QR-A70H	1	1	1	1	1
›chromocytoma and Paragangl	TCGA-QR-A70I	0	0	1	0	1
›chromocytoma and Paragangl	TCGA-QR-A70J	1	1	1	1	1
›chromocytoma and Paragangl	TCGA-QR-A70K	1	1	1	1	1
›chromocytoma and Paragangl	TCGA-QR-A70M	0	0	0	0	0
›chromocytoma and Paragangl	TCGA-QR-A70N	1	0	0	1	1
›chromocytoma and Paragangl	TCGA-QR-A70O	0	0	0	0	0
›chromocytoma and Paragangl	TCGA-QR-A70P	0	1	0	1	1
›chromocytoma and Paragangl	TCGA-QR-A70Q	0	0	0	0	0
›chromocytoma and Paragangl	TCGA-QR-A70R	1	1	1	1	1
›chromocytoma and Paragangl	TCGA-QR-A70T	0	0	0	0	0
›chromocytoma and Paragangl	TCGA-QR-A70U	0	0	0	0	0
›chromocytoma and Paragangl	TCGA-QR-A70V	0	0	0	0	0
›chromocytoma and Paragangl	TCGA-QR-A70W	1	1	1	1	1
›chromocytoma and Paragangl	TCGA-QR-A70X	1	1	1	1	1
›chromocytoma and Paragangl	TCGA-QR-A7IN	1	1	1	1	1
›chromocytoma and Paragangl	TCGA-QR-A7IP	1	1	1	1	1
›chromocytoma and Paragangl	TCGA-QT-A5XJ	1	1	1	1	1
›chromocytoma and Paragangl	TCGA-QT-A5XK	1	1	0	1	1
›chromocytoma and Paragangl	TCGA-QT-A5XL	1	0	0	1	1
›chromocytoma and Paragangl	TCGA-QT-A5XM	1	1	0	1	1
›chromocytoma and Paragangl	TCGA-QT-A5XN	1	1	1	1	1
›chromocytoma and Paragangl	TCGA-QT-A5XO	1	1	0	1	1
›chromocytoma and Paragangl	TCGA-QT-A5XP	1	1	1	1	1
›chromocytoma and Paragangl	TCGA-QT-A69Q	1	1	1	1	1
›chromocytoma and Paragangl	TCGA-QT-A7U0	0	0	0	0	0
›chromocytoma and Paragangl	TCGA-RM-A68T	1	1	0	1	1
›chromocytoma and Paragangl	TCGA-RM-A68W	1	0	1	0	1
›chromocytoma and Paragangl	TCGA-RT-A6Y9	1	0	0	1	1
›chromocytoma and Paragangl	TCGA-RT-A6YA	1	1	1	1	1
›chromocytoma and Paragangl	TCGA-RT-A6YC	0	1	0	1	0
›chromocytoma and Paragangl	TCGA-RW-A67V	0	1	1	1	1
›chromocytoma and Paragangl	TCGA-RW-A67W	1	1	1	1	0
›chromocytoma and Paragangl	TCGA-RW-A67X	1	1	1	1	1
›chromocytoma and Paragangl	TCGA-RW-A67Y	1	1	1	1	1
›chromocytoma and Paragangl	TCGA-RW-A680	1	1	0	1	0
›chromocytoma and Paragangl	TCGA-RW-A681	1	0	1	1	1
›chromocytoma and Paragangl	TCGA-RW-A684	0	1	0	1	0
›chromocytoma and Paragangl	TCGA-RW-A685	1	1	1	1	1
›chromocytoma and Paragangl	TCGA-RW-A686	0	1	0	1	0
›chromocytoma and Paragangl	TCGA-RW-A686	0	0	0	0	0
›chromocytoma and Paragangl	TCGA-RW-A688	1	1	1	1	1
›chromocytoma and Paragangl	TCGA-RW-A689	1	1	1	1	1
›chromocytoma and Paragangl	TCGA-RW-A68A	1	1	1	1	1
›chromocytoma and Paragangl	TCGA-RW-A68B	1	1	0	1	0

ochromocytoma and Paragangl	TCGA-RW-A68C	1	1	1	1	1
ochromocytoma and Paragangl	TCGA-RW-A68D	1	1	0	1	0
ochromocytoma and Paragangl	TCGA-RW-A68F	0	0	1	0	1
ochromocytoma and Paragangl	TCGA-RW-A68G	0	0	1	0	1
ochromocytoma and Paragangl	TCGA-RW-A7CZ	1	0	0	1	1
ochromocytoma and Paragangl	TCGA-RW-A7D0	1	1	1	1	1
ochromocytoma and Paragangl	TCGA-RW-A8AZ	1	1	1	1	1
ochromocytoma and Paragangl	TCGA-RX-A8JQ	0	1	0	1	0
ochromocytoma and Paragangl	TCGA-S7-A7WL	1	0	1	1	1
ochromocytoma and Paragangl	TCGA-S7-A7WM	1	1	1	1	1
ochromocytoma and Paragangl	TCGA-S7-A7WN	0	1	1	1	1
ochromocytoma and Paragangl	TCGA-S7-A7WO	0	0	0	0	0
ochromocytoma and Paragangl	TCGA-S7-A7WP	1	0	0	1	1
ochromocytoma and Paragangl	TCGA-S7-A7WQ	1	1	1	1	1
ochromocytoma and Paragangl	TCGA-S7-A7WR	1	1	1	1	1
ochromocytoma and Paragangl	TCGA-S7-A7WT	0	1	1	1	1
ochromocytoma and Paragangl	TCGA-S7-A7WU	0	0	0	0	1
ochromocytoma and Paragangl	TCGA-S7-A7WV	1	0	0	0	0
ochromocytoma and Paragangl	TCGA-S7-A7WW	0	0	1	0	1
ochromocytoma and Paragangl	TCGA-S7-A7WX	1	1	1	1	1
ochromocytoma and Paragangl	TCGA-S7-A7X0	1	1	1	1	1
ochromocytoma and Paragangl	TCGA-S7-A7X1	1	1	1	1	1
ochromocytoma and Paragangl	TCGA-S7-A7X2	1	1	1	1	1
ochromocytoma and Paragangl	TCGA-SA-A6C2	0	0	0	0	0
ochromocytoma and Paragangl	TCGA-SP-A6QC	0	1	1	1	1
ochromocytoma and Paragangl	TCGA-SP-A6QD	1	1	1	1	1
ochromocytoma and Paragangl	TCGA-SP-A6QF	1	1	0	1	1
ochromocytoma and Paragangl	TCGA-SP-A6QG	1	1	1	1	1
ochromocytoma and Paragangl	TCGA-SP-A6QH	0	0	0	0	0
ochromocytoma and Paragangl	TCGA-SP-A6QI	1	1	0	1	0
ochromocytoma and Paragangl	TCGA-SP-A6QJ	1	1	1	1	1
ochromocytoma and Paragangl	TCGA-SP-A6QK	1	1	0	1	1
ochromocytoma and Paragangl	TCGA-SQ-A6I4	1	1	0	1	0
ochromocytoma and Paragangl	TCGA-SQ-A6I6	1	1	1	1	1
ochromocytoma and Paragangl	TCGA-SR-A6MP	1	1	1	1	1
ochromocytoma and Paragangl	TCGA-SR-A6MQ	1	1	1	1	1
ochromocytoma and Paragangl	TCGA-SR-A6MR	0	0	0	0	0
ochromocytoma and Paragangl	TCGA-SR-A6MS	1	1	0	1	0
ochromocytoma and Paragangl	TCGA-SR-A6MT	1	1	1	1	1
ochromocytoma and Paragangl	TCGA-SR-A6MU	1	1	0	1	0
ochromocytoma and Paragangl	TCGA-SR-A6MV	1	1	0	1	1
ochromocytoma and Paragangl	TCGA-SR-A6MX	1	1	1	1	1
ochromocytoma and Paragangl	TCGA-SR-A6MX	0	0	0	0	0
ochromocytoma and Paragangl	TCGA-SR-A6MX	0	0	0	0	0
ochromocytoma and Paragangl	TCGA-SR-A6MY	1	1	1	1	1
ochromocytoma and Paragangl	TCGA-SR-A6MZ	1	1	0	1	1
ochromocytoma and Paragangl	TCGA-SR-A6N0	0	0	0	0	1
ochromocytoma and Paragangl	TCGA-TT-A6YJ	1	1	1	1	1
ochromocytoma and Paragangl	TCGA-TT-A6YK	1	1	1	1	1
ochromocytoma and Paragangl	TCGA-TT-A6YN	1	1	1	1	1
ochromocytoma and Paragangl	TCGA-TT-A6YO	0	0	0	0	0
ochromocytoma and Paragangl	TCGA-TT-A6YP	1	0	0	1	1
ochromocytoma and Paragangl	TCGA-W2-A7H5	1	1	0	1	1
ochromocytoma and Paragangl	TCGA-W2-A7H7	1	1	0	1	1
ochromocytoma and Paragangl	TCGA-W2-A7HA	1	1	1	1	1
ochromocytoma and Paragangl	TCGA-W2-A7HB	1	1	1	1	1
ochromocytoma and Paragangl	TCGA-W2-A7HC	1	1	1	1	1
ochromocytoma and Paragangl	TCGA-W2-A7HD	1	1	0	1	1

ochromocytoma and Paragangl	TCGA-W2-A7HE	0	1	0	1	1
ochromocytoma and Paragangl	TCGA-W2-A7HF	1	1	0	1	1
ochromocytoma and Paragangl	TCGA-W2-A7HH	0	0	0	0	0
ochromocytoma and Paragangl	TCGA-W2-A7UY	1	1	1	1	1
ochromocytoma and Paragangl	TCGA-WB-A80K	1	0	0	1	1
ochromocytoma and Paragangl	TCGA-WB-A80L	1	0	0	0	1
ochromocytoma and Paragangl	TCGA-WB-A80M	1	1	1	1	1
ochromocytoma and Paragangl	TCGA-WB-A80N	1	1	1	1	1
ochromocytoma and Paragangl	TCGA-WB-A80O	0	1	1	1	1
ochromocytoma and Paragangl	TCGA-WB-A80P	1	1	1	1	1
ochromocytoma and Paragangl	TCGA-WB-A80Q	0	0	0	0	1
ochromocytoma and Paragangl	TCGA-WB-A80V	1	1	1	1	1
ochromocytoma and Paragangl	TCGA-WB-A80Y	1	0	1	1	1
ochromocytoma and Paragangl	TCGA-WB-A814	1	0	0	1	1
ochromocytoma and Paragangl	TCGA-WB-A815	1	0	1	1	1
ochromocytoma and Paragangl	TCGA-WB-A816	1	1	0	1	0
ochromocytoma and Paragangl	TCGA-WB-A817	0	0	0	0	0
ochromocytoma and Paragangl	TCGA-WB-A818	1	1	1	1	1
ochromocytoma and Paragangl	TCGA-WB-A819	1	1	1	1	1
ochromocytoma and Paragangl	TCGA-WB-A81A	0	0	0	1	1
ochromocytoma and Paragangl	TCGA-WB-A81D	0	1	0	1	1
ochromocytoma and Paragangl	TCGA-WB-A81E	1	1	0	1	0
ochromocytoma and Paragangl	TCGA-WB-A81F	1	0	1	1	1
ochromocytoma and Paragangl	TCGA-WB-A81G	1	1	0	1	0
ochromocytoma and Paragangl	TCGA-WB-A81H	0	1	0	1	1
ochromocytoma and Paragangl	TCGA-WB-A81I	0	0	0	0	0
ochromocytoma and Paragangl	TCGA-WB-A81J	1	1	1	0	1
ochromocytoma and Paragangl	TCGA-WB-A81K	1	1	0	1	0
ochromocytoma and Paragangl	TCGA-WB-A81M	0	0	1	1	1
ochromocytoma and Paragangl	TCGA-WB-A81N	0	0	1	1	1
ochromocytoma and Paragangl	TCGA-WB-A81P	1	1	1	1	1
ochromocytoma and Paragangl	TCGA-WB-A81Q	1	1	1	1	1
ochromocytoma and Paragangl	TCGA-WB-A81R	1	1	1	1	1
ochromocytoma and Paragangl	TCGA-WB-A81S	1	1	1	1	1
ochromocytoma and Paragangl	TCGA-WB-A81T	1	1	1	1	1
ochromocytoma and Paragangl	TCGA-WB-A81V	0	1	0	1	1
ochromocytoma and Paragangl	TCGA-WB-A81W	1	1	1	1	1
ochromocytoma and Paragangl	TCGA-WB-A820	1	1	1	1	1
ochromocytoma and Paragangl	TCGA-WB-A821	1	1	1	1	1
ochromocytoma and Paragangl	TCGA-WB-A822	1	1	1	1	1
ochromocytoma and Paragangl	TCGA-XG-A823	1	0	1	1	1
Prostate Adenocarcinoma	TCGA-2A-A8VL	0	0	0	1	1
Prostate Adenocarcinoma	TCGA-2A-A8VO	0	0	1	0	1
Prostate Adenocarcinoma	TCGA-2A-A8VT	1	0	1	1	1
Prostate Adenocarcinoma	TCGA-2A-A8VV	0	1	0	1	0
Prostate Adenocarcinoma	TCGA-2A-A8VX	0	1	1	0	0
Prostate Adenocarcinoma	TCGA-2A-A8W1	1	1	1	0	0
Prostate Adenocarcinoma	TCGA-2A-A8W3	1	1	1	0	1
Prostate Adenocarcinoma	TCGA-2A-AAYF	0	0	0	0	1
Prostate Adenocarcinoma	TCGA-2A-AAYO	0	1	0	0	0
Prostate Adenocarcinoma	TCGA-2A-AAYU	1	1	1	0	1
Prostate Adenocarcinoma	TCGA-4L-AA1F	1	1	1	1	1
Prostate Adenocarcinoma	TCGA-CH-5737	1	0	0	1	1
Prostate Adenocarcinoma	TCGA-CH-5738	0	0	0	0	0
Prostate Adenocarcinoma	TCGA-CH-5739	1	1	0	1	1
Prostate Adenocarcinoma	TCGA-CH-5740	1	0	1	0	1
Prostate Adenocarcinoma	TCGA-CH-5741	1	1	0	0	0
Prostate Adenocarcinoma	TCGA-CH-5743	0	0	0	0	0

Prostate Adenocarcinoma	TCGA-CH-5744	1	1	1	0	0
Prostate Adenocarcinoma	TCGA-CH-5745	0	0	0	0	0
Prostate Adenocarcinoma	TCGA-CH-5746	1	0	0	0	0
Prostate Adenocarcinoma	TCGA-CH-5748	1	0	1	0	0
Prostate Adenocarcinoma	TCGA-CH-5750	0	1	1	0	0
Prostate Adenocarcinoma	TCGA-CH-5751	1	1	1	1	1
Prostate Adenocarcinoma	TCGA-CH-5752	1	0	1	0	1
Prostate Adenocarcinoma	TCGA-CH-5753	1	1	1	1	1
Prostate Adenocarcinoma	TCGA-CH-5754	1	1	1	1	1
Prostate Adenocarcinoma	TCGA-CH-5761	1	1	1	1	1
Prostate Adenocarcinoma	TCGA-CH-5762	0	0	0	0	1
Prostate Adenocarcinoma	TCGA-CH-5763	0	0	0	0	0
Prostate Adenocarcinoma	TCGA-CH-5764	0	1	0	1	1
Prostate Adenocarcinoma	TCGA-CH-5765	1	1	1	0	1
Prostate Adenocarcinoma	TCGA-CH-5766	1	1	1	1	1
Prostate Adenocarcinoma	TCGA-CH-5767	1	1	1	1	1
Prostate Adenocarcinoma	TCGA-CH-5768	1	1	1	0	1
Prostate Adenocarcinoma	TCGA-CH-5769	1	0	0	1	1
Prostate Adenocarcinoma	TCGA-CH-5771	0	1	0	0	0
Prostate Adenocarcinoma	TCGA-CH-5772	1	0	0	1	1
Prostate Adenocarcinoma	TCGA-CH-5788	0	1	1	0	1
Prostate Adenocarcinoma	TCGA-CH-5789	0	0	0	0	0
Prostate Adenocarcinoma	TCGA-CH-5790	1	1	1	0	1
Prostate Adenocarcinoma	TCGA-CH-5791	1	1	1	1	1
Prostate Adenocarcinoma	TCGA-CH-5792	1	0	1	0	1
Prostate Adenocarcinoma	TCGA-CH-5794	1	1	0	0	1
Prostate Adenocarcinoma	TCGA-EJ-5494	0	0	0	0	0
Prostate Adenocarcinoma	TCGA-EJ-5495	1	0	0	0	0
Prostate Adenocarcinoma	TCGA-EJ-5496	0	0	0	0	0
Prostate Adenocarcinoma	TCGA-EJ-5497	1	0	0	1	0
Prostate Adenocarcinoma	TCGA-EJ-5498	0	1	0	1	0
Prostate Adenocarcinoma	TCGA-EJ-5499	1	1	1	0	1
Prostate Adenocarcinoma	TCGA-EJ-5501	1	1	1	0	1
Prostate Adenocarcinoma	TCGA-EJ-5502	0	0	0	0	0
Prostate Adenocarcinoma	TCGA-EJ-5503	0	0	0	0	0
Prostate Adenocarcinoma	TCGA-EJ-5504	1	0	1	0	0
Prostate Adenocarcinoma	TCGA-EJ-5505	0	0	1	1	0
Prostate Adenocarcinoma	TCGA-EJ-5506	0	0	1	0	0
Prostate Adenocarcinoma	TCGA-EJ-5507	1	1	1	1	1
Prostate Adenocarcinoma	TCGA-EJ-5508	1	0	0	1	0
Prostate Adenocarcinoma	TCGA-EJ-5509	1	0	1	0	1
Prostate Adenocarcinoma	TCGA-EJ-5510	1	1	1	0	0
Prostate Adenocarcinoma	TCGA-EJ-5511	1	1	1	1	1
Prostate Adenocarcinoma	TCGA-EJ-5512	0	0	0	0	0
Prostate Adenocarcinoma	TCGA-EJ-5514	1	1	1	0	1
Prostate Adenocarcinoma	TCGA-EJ-5515	0	0	0	0	0
Prostate Adenocarcinoma	TCGA-EJ-5516	1	0	1	0	1
Prostate Adenocarcinoma	TCGA-EJ-5517	1	1	1	0	0
Prostate Adenocarcinoma	TCGA-EJ-5518	1	0	1	0	1
Prostate Adenocarcinoma	TCGA-EJ-5519	1	1	1	0	1
Prostate Adenocarcinoma	TCGA-EJ-5521	1	1	1	1	1
Prostate Adenocarcinoma	TCGA-EJ-5522	1	0	1	0	1
Prostate Adenocarcinoma	TCGA-EJ-5524	1	1	1	0	1
Prostate Adenocarcinoma	TCGA-EJ-5525	1	1	1	0	1
Prostate Adenocarcinoma	TCGA-EJ-5526	1	0	1	0	1
Prostate Adenocarcinoma	TCGA-EJ-5527	1	1	0	1	1
Prostate Adenocarcinoma	TCGA-EJ-5530	1	1	0	0	0
Prostate Adenocarcinoma	TCGA-EJ-5531	1	1	1	1	0

Prostate Adenocarcinoma	TCGA-EJ-5532	1	1	0	1	1
Prostate Adenocarcinoma	TCGA-EJ-5542	1	1	1	0	0
Prostate Adenocarcinoma	TCGA-EJ-7115	1	0	1	0	1
Prostate Adenocarcinoma	TCGA-EJ-7123	1	1	1	1	0
Prostate Adenocarcinoma	TCGA-EJ-7125	0	0	0	0	0
Prostate Adenocarcinoma	TCGA-EJ-7218	1	0	1	0	0
Prostate Adenocarcinoma	TCGA-EJ-7312	1	0	1	0	1
Prostate Adenocarcinoma	TCGA-EJ-7314	1	0	1	0	0
Prostate Adenocarcinoma	TCGA-EJ-7315	1	0	1	0	1
Prostate Adenocarcinoma	TCGA-EJ-7317	0	0	1	0	1
Prostate Adenocarcinoma	TCGA-EJ-7318	0	1	1	0	1
Prostate Adenocarcinoma	TCGA-EJ-7321	0	0	0	0	1
Prostate Adenocarcinoma	TCGA-EJ-7325	1	1	1	0	1
Prostate Adenocarcinoma	TCGA-EJ-7327	1	0	1	0	1
Prostate Adenocarcinoma	TCGA-EJ-7328	1	1	1	0	1
Prostate Adenocarcinoma	TCGA-EJ-7330	0	1	1	0	1
Prostate Adenocarcinoma	TCGA-EJ-7331	1	0	1	0	0
Prostate Adenocarcinoma	TCGA-EJ-7781	1	1	1	1	1
Prostate Adenocarcinoma	TCGA-EJ-7782	0	0	1	1	0
Prostate Adenocarcinoma	TCGA-EJ-7783	1	1	0	1	1
Prostate Adenocarcinoma	TCGA-EJ-7784	1	1	1	1	1
Prostate Adenocarcinoma	TCGA-EJ-7785	1	0	1	0	0
Prostate Adenocarcinoma	TCGA-EJ-7786	1	0	1	0	1
Prostate Adenocarcinoma	TCGA-EJ-7788	1	1	1	1	0
Prostate Adenocarcinoma	TCGA-EJ-7789	1	1	1	0	1
Prostate Adenocarcinoma	TCGA-EJ-7791	1	0	0	0	0
Prostate Adenocarcinoma	TCGA-EJ-7792	0	0	0	1	0
Prostate Adenocarcinoma	TCGA-EJ-7793	0	0	0	0	0
Prostate Adenocarcinoma	TCGA-EJ-7794	1	0	0	0	0
Prostate Adenocarcinoma	TCGA-EJ-7797	0	0	1	0	1
Prostate Adenocarcinoma	TCGA-EJ-8468	1	0	1	0	0
Prostate Adenocarcinoma	TCGA-EJ-8469	1	1	0	1	1
Prostate Adenocarcinoma	TCGA-EJ-8470	1	1	1	0	0
Prostate Adenocarcinoma	TCGA-EJ-8472	1	1	1	1	1
Prostate Adenocarcinoma	TCGA-EJ-8474	1	1	1	0	1
Prostate Adenocarcinoma	TCGA-EJ-A46B	0	0	0	0	0
Prostate Adenocarcinoma	TCGA-EJ-A46D	0	0	0	0	0
Prostate Adenocarcinoma	TCGA-EJ-A46E	0	0	0	0	0
Prostate Adenocarcinoma	TCGA-EJ-A46F	1	0	1	0	1
Prostate Adenocarcinoma	TCGA-EJ-A46G	1	1	1	0	1
Prostate Adenocarcinoma	TCGA-EJ-A46H	0	0	0	0	0
Prostate Adenocarcinoma	TCGA-EJ-A46I	0	0	0	0	0
Prostate Adenocarcinoma	TCGA-EJ-A65B	1	0	0	0	1
Prostate Adenocarcinoma	TCGA-EJ-A65D	1	1	1	1	0
Prostate Adenocarcinoma	TCGA-EJ-A65E	1	1	1	1	0
Prostate Adenocarcinoma	TCGA-EJ-A65F	1	0	1	0	0
Prostate Adenocarcinoma	TCGA-EJ-A65G	0	0	0	0	0
Prostate Adenocarcinoma	TCGA-EJ-A65J	1	0	1	0	1
Prostate Adenocarcinoma	TCGA-EJ-A65M	1	0	1	0	1
Prostate Adenocarcinoma	TCGA-EJ-A6RA	0	0	0	0	0
Prostate Adenocarcinoma	TCGA-EJ-A6RC	0	0	0	0	0
Prostate Adenocarcinoma	TCGA-EJ-A7NF	1	0	1	0	1
Prostate Adenocarcinoma	TCGA-EJ-A7NG	0	0	0	0	0
Prostate Adenocarcinoma	TCGA-EJ-A7NH	1	1	1	1	1
Prostate Adenocarcinoma	TCGA-EJ-A7NJ	0	0	0	0	0
Prostate Adenocarcinoma	TCGA-EJ-A7NK	0	0	0	0	0
Prostate Adenocarcinoma	TCGA-EJ-A7NM	1	0	0	0	1
Prostate Adenocarcinoma	TCGA-EJ-A7NN	0	0	0	0	0

Prostate Adenocarcinoma	TCGA-EJ-A8FN	0	0	1	0	1
Prostate Adenocarcinoma	TCGA-EJ-A8FO	0	0	0	0	0
Prostate Adenocarcinoma	TCGA-EJ-A8FP	0	0	0	0	0
Prostate Adenocarcinoma	TCGA-EJ-A8FS	1	1	1	0	1
Prostate Adenocarcinoma	TCGA-EJ-A8FU	0	0	0	0	0
Prostate Adenocarcinoma	TCGA-EJ-AB20	0	0	0	0	0
Prostate Adenocarcinoma	TCGA-EJ-AB27	0	0	0	0	0
Prostate Adenocarcinoma	TCGA-FC-7708	1	0	0	1	1
Prostate Adenocarcinoma	TCGA-FC-7961	1	1	1	1	1
Prostate Adenocarcinoma	TCGA-FC-A4JI	1	1	1	1	1
Prostate Adenocarcinoma	TCGA-FC-A5OB	0	0	0	0	0
Prostate Adenocarcinoma	TCGA-FC-A66V	0	0	0	0	0
Prostate Adenocarcinoma	TCGA-FC-A6HD	0	0	0	0	0
Prostate Adenocarcinoma	TCGA-FC-A8O0	0	0	0	0	0
Prostate Adenocarcinoma	TCGA-G9-6329	1	1	0	1	0
Prostate Adenocarcinoma	TCGA-G9-6332	1	1	0	0	1
Prostate Adenocarcinoma	TCGA-G9-6333	1	1	1	0	0
Prostate Adenocarcinoma	TCGA-G9-6336	0	0	0	0	0
Prostate Adenocarcinoma	TCGA-G9-6338	1	0	1	0	1
Prostate Adenocarcinoma	TCGA-G9-6339	1	1	1	0	1
Prostate Adenocarcinoma	TCGA-G9-6342	1	1	0	0	1
Prostate Adenocarcinoma	TCGA-G9-6343	0	1	0	0	0
Prostate Adenocarcinoma	TCGA-G9-6347	0	0	0	0	0
Prostate Adenocarcinoma	TCGA-G9-6348	1	0	1	0	0
Prostate Adenocarcinoma	TCGA-G9-6351	1	0	0	1	0
Prostate Adenocarcinoma	TCGA-G9-6353	0	0	0	0	0
Prostate Adenocarcinoma	TCGA-G9-6354	1	1	1	0	1
Prostate Adenocarcinoma	TCGA-G9-6356	1	0	0	1	1
Prostate Adenocarcinoma	TCGA-G9-6361	1	1	1	1	1
Prostate Adenocarcinoma	TCGA-G9-6362	1	0	1	1	1
Prostate Adenocarcinoma	TCGA-G9-6363	1	1	0	1	1
Prostate Adenocarcinoma	TCGA-G9-6364	1	1	1	0	1
Prostate Adenocarcinoma	TCGA-G9-6365	0	0	0	0	0
Prostate Adenocarcinoma	TCGA-G9-6366	1	0	0	1	1
Prostate Adenocarcinoma	TCGA-G9-6367	0	0	0	0	0
Prostate Adenocarcinoma	TCGA-G9-6369	0	1	0	1	1
Prostate Adenocarcinoma	TCGA-G9-6370	0	0	0	0	0
Prostate Adenocarcinoma	TCGA-G9-6371	0	1	0	0	0
Prostate Adenocarcinoma	TCGA-G9-6373	1	1	1	1	1
Prostate Adenocarcinoma	TCGA-G9-6377	1	1	1	1	1
Prostate Adenocarcinoma	TCGA-G9-6378	1	0	0	0	0
Prostate Adenocarcinoma	TCGA-G9-6379	1	0	1	1	1
Prostate Adenocarcinoma	TCGA-G9-6384	1	0	1	0	1
Prostate Adenocarcinoma	TCGA-G9-6385	0	0	0	0	0
Prostate Adenocarcinoma	TCGA-G9-6494	0	1	1	0	1
Prostate Adenocarcinoma	TCGA-G9-6496	0	0	1	1	0
Prostate Adenocarcinoma	TCGA-G9-6498	0	0	0	1	0
Prostate Adenocarcinoma	TCGA-G9-6499	1	1	1	1	1
Prostate Adenocarcinoma	TCGA-G9-7509	0	1	1	0	1
Prostate Adenocarcinoma	TCGA-G9-7510	1	1	1	0	1
Prostate Adenocarcinoma	TCGA-G9-7519	0	0	0	0	0
Prostate Adenocarcinoma	TCGA-G9-7521	1	1	1	1	1
Prostate Adenocarcinoma	TCGA-G9-7522	1	0	0	0	0
Prostate Adenocarcinoma	TCGA-G9-7523	1	1	0	1	0
Prostate Adenocarcinoma	TCGA-G9-7525	1	0	1	0	0
Prostate Adenocarcinoma	TCGA-G9-A9S0	1	1	1	1	1
Prostate Adenocarcinoma	TCGA-G9-A9S4	0	0	0	0	0
Prostate Adenocarcinoma	TCGA-G9-A9S7	1	1	1	0	1

Prostate Adenocarcinoma	TCGA-H9-7775	1	1	0	1	0
Prostate Adenocarcinoma	TCGA-H9-A6BX	0	0	0	0	0
Prostate Adenocarcinoma	TCGA-H9-A6BY	0	0	1	0	1
Prostate Adenocarcinoma	TCGA-HC-7075	0	0	0	0	0
Prostate Adenocarcinoma	TCGA-HC-7077	1	1	0	0	0
Prostate Adenocarcinoma	TCGA-HC-7078	1	1	1	1	1
Prostate Adenocarcinoma	TCGA-HC-7079	0	0	0	0	0
Prostate Adenocarcinoma	TCGA-HC-7080	1	1	1	1	1
Prostate Adenocarcinoma	TCGA-HC-7081	1	0	1	0	0
Prostate Adenocarcinoma	TCGA-HC-7209	1	0	1	0	1
Prostate Adenocarcinoma	TCGA-HC-7210	1	1	0	0	1
Prostate Adenocarcinoma	TCGA-HC-7211	1	0	1	0	1
Prostate Adenocarcinoma	TCGA-HC-7212	1	1	0	0	1
Prostate Adenocarcinoma	TCGA-HC-7213	1	1	1	1	1
Prostate Adenocarcinoma	TCGA-HC-7230	1	0	1	0	1
Prostate Adenocarcinoma	TCGA-HC-7231	1	1	0	0	1
Prostate Adenocarcinoma	TCGA-HC-7232	1	0	0	0	1
Prostate Adenocarcinoma	TCGA-HC-7233	0	0	0	0	0
Prostate Adenocarcinoma	TCGA-HC-7736	1	1	1	1	1
Prostate Adenocarcinoma	TCGA-HC-7737	1	0	0	0	1
Prostate Adenocarcinoma	TCGA-HC-7738	1	0	0	0	0
Prostate Adenocarcinoma	TCGA-HC-7740	0	0	0	0	0
Prostate Adenocarcinoma	TCGA-HC-7742	1	1	1	1	1
Prostate Adenocarcinoma	TCGA-HC-7744	1	0	1	0	1
Prostate Adenocarcinoma	TCGA-HC-7745	0	1	0	1	1
Prostate Adenocarcinoma	TCGA-HC-7747	1	0	1	1	1
Prostate Adenocarcinoma	TCGA-HC-7748	1	0	0	0	0
Prostate Adenocarcinoma	TCGA-HC-7749	1	1	1	1	1
Prostate Adenocarcinoma	TCGA-HC-7750	0	0	0	0	1
Prostate Adenocarcinoma	TCGA-HC-7752	1	1	0	1	1
Prostate Adenocarcinoma	TCGA-HC-7817	1	0	1	0	0
Prostate Adenocarcinoma	TCGA-HC-7818	0	0	0	0	0
Prostate Adenocarcinoma	TCGA-HC-7819	1	1	0	0	0
Prostate Adenocarcinoma	TCGA-HC-7820	1	0	1	0	0
Prostate Adenocarcinoma	TCGA-HC-7821	1	1	1	1	1
Prostate Adenocarcinoma	TCGA-HC-8213	0	0	0	0	0
Prostate Adenocarcinoma	TCGA-HC-8216	1	1	1	1	1
Prostate Adenocarcinoma	TCGA-HC-8256	1	1	0	1	1
Prostate Adenocarcinoma	TCGA-HC-8257	1	0	1	1	1
Prostate Adenocarcinoma	TCGA-HC-8258	0	1	0	0	0
Prostate Adenocarcinoma	TCGA-HC-8259	0	0	0	0	0
Prostate Adenocarcinoma	TCGA-HC-8260	0	0	0	0	1
Prostate Adenocarcinoma	TCGA-HC-8261	0	0	0	0	0
Prostate Adenocarcinoma	TCGA-HC-8262	1	1	1	0	0
Prostate Adenocarcinoma	TCGA-HC-8264	1	0	1	0	1
Prostate Adenocarcinoma	TCGA-HC-8265	1	1	1	1	1
Prostate Adenocarcinoma	TCGA-HC-8266	1	1	1	1	1
Prostate Adenocarcinoma	TCGA-HC-A48F	1	1	1	1	1
Prostate Adenocarcinoma	TCGA-HC-A4ZV	1	1	1	1	1
Prostate Adenocarcinoma	TCGA-HC-A631	1	1	1	0	1
Prostate Adenocarcinoma	TCGA-HC-A632	1	1	1	1	1
Prostate Adenocarcinoma	TCGA-HC-A6AL	1	1	1	0	0
Prostate Adenocarcinoma	TCGA-HC-A6AN	1	1	1	0	0
Prostate Adenocarcinoma	TCGA-HC-A6AO	0	1	1	0	1
Prostate Adenocarcinoma	TCGA-HC-A6AP	0	0	1	0	0
Prostate Adenocarcinoma	TCGA-HC-A6AQ	0	1	0	0	0
Prostate Adenocarcinoma	TCGA-HC-A6AS	1	0	1	0	0
Prostate Adenocarcinoma	TCGA-HC-A6HX	0	1	0	1	0

Prostate Adenocarcinoma	TCGA-HC-A6HY	1	0	1	0	1
Prostate Adenocarcinoma	TCGA-HC-A76W	1	1	1	1	1
Prostate Adenocarcinoma	TCGA-HC-A76X	0	0	0	0	1
Prostate Adenocarcinoma	TCGA-HC-A8CY	1	1	1	0	1
Prostate Adenocarcinoma	TCGA-HC-A8D0	1	1	0	0	1
Prostate Adenocarcinoma	TCGA-HC-A8D1	0	0	1	0	0
Prostate Adenocarcinoma	TCGA-HC-A9TE	1	1	1	1	1
Prostate Adenocarcinoma	TCGA-HC-A9TH	1	1	1	1	1
Prostate Adenocarcinoma	TCGA-HI-7168	1	1	1	1	1
Prostate Adenocarcinoma	TCGA-HI-7169	0	0	0	0	0
Prostate Adenocarcinoma	TCGA-HI-7170	1	1	0	0	0
Prostate Adenocarcinoma	TCGA-HI-7171	1	1	1	1	1
Prostate Adenocarcinoma	TCGA-J4-8198	0	0	0	0	0
Prostate Adenocarcinoma	TCGA-J4-8200	1	0	0	0	0
Prostate Adenocarcinoma	TCGA-J4-A67K	1	0	1	0	1
Prostate Adenocarcinoma	TCGA-J4-A67L	1	1	1	0	1
Prostate Adenocarcinoma	TCGA-J4-A67M	1	1	1	0	1
Prostate Adenocarcinoma	TCGA-J4-A67N	1	1	1	0	1
Prostate Adenocarcinoma	TCGA-J4-A67O	1	0	0	0	0
Prostate Adenocarcinoma	TCGA-J4-A67Q	0	0	0	0	1
Prostate Adenocarcinoma	TCGA-J4-A67R	0	0	0	0	0
Prostate Adenocarcinoma	TCGA-J4-A67S	1	1	0	0	1
Prostate Adenocarcinoma	TCGA-J4-A67T	1	0	1	0	1
Prostate Adenocarcinoma	TCGA-J4-A6G1	0	0	0	0	0
Prostate Adenocarcinoma	TCGA-J4-A6G3	0	0	0	0	0
Prostate Adenocarcinoma	TCGA-J4-A6M7	0	0	0	0	0
Prostate Adenocarcinoma	TCGA-J4-A83I	1	1	0	1	1
Prostate Adenocarcinoma	TCGA-J4-A83J	1	0	1	0	1
Prostate Adenocarcinoma	TCGA-J4-A83K	0	0	1	0	0
Prostate Adenocarcinoma	TCGA-J4-A83L	0	0	1	0	1
Prostate Adenocarcinoma	TCGA-J4-A83M	1	0	0	0	0
Prostate Adenocarcinoma	TCGA-J4-A83N	1	1	0	0	0
Prostate Adenocarcinoma	TCGA-J4-AATV	0	0	0	0	0
Prostate Adenocarcinoma	TCGA-J4-AATZ	1	1	1	1	1
Prostate Adenocarcinoma	TCGA-J4-AAU2	0	0	0	0	0
Prostate Adenocarcinoma	TCGA-J9-A52B	1	1	1	1	1
Prostate Adenocarcinoma	TCGA-J9-A52C	1	1	1	0	1
Prostate Adenocarcinoma	TCGA-J9-A52D	0	0	0	0	0
Prostate Adenocarcinoma	TCGA-J9-A52E	1	1	1	1	1
Prostate Adenocarcinoma	TCGA-J9-A8CK	0	1	1	1	1
Prostate Adenocarcinoma	TCGA-J9-A8CL	1	1	1	1	1
Prostate Adenocarcinoma	TCGA-J9-A8CM	1	0	1	0	1
Prostate Adenocarcinoma	TCGA-J9-A8CN	1	1	0	0	0
Prostate Adenocarcinoma	TCGA-J9-A8CP	1	0	1	0	1
Prostate Adenocarcinoma	TCGA-KC-A4BL	1	0	0	1	1
Prostate Adenocarcinoma	TCGA-KC-A4BN	0	0	0	1	0
Prostate Adenocarcinoma	TCGA-KC-A4BR	0	0	0	0	0
Prostate Adenocarcinoma	TCGA-KC-A4BV	1	1	1	0	1
Prostate Adenocarcinoma	TCGA-KC-A7F3	1	0	1	0	0
Prostate Adenocarcinoma	TCGA-KC-A7F5	0	0	0	0	0
Prostate Adenocarcinoma	TCGA-KC-A7F6	1	1	1	0	1
Prostate Adenocarcinoma	TCGA-KC-A7FA	1	1	1	1	1
Prostate Adenocarcinoma	TCGA-KC-A7FD	0	1	0	0	0
Prostate Adenocarcinoma	TCGA-KC-A7FE	0	0	0	0	0
Prostate Adenocarcinoma	TCGA-KK-A59V	1	1	1	1	1
Prostate Adenocarcinoma	TCGA-KK-A59X	1	1	1	1	1
Prostate Adenocarcinoma	TCGA-KK-A59Y	1	1	0	1	1
Prostate Adenocarcinoma	TCGA-KK-A59Z	1	1	1	1	1

Prostate Adenocarcinoma	TCGA-KK-A5A1	0	0	0	0	0
Prostate Adenocarcinoma	TCGA-KK-A6DY	1	1	1	0	1
Prostate Adenocarcinoma	TCGA-KK-A6E0	1	1	1	1	1
Prostate Adenocarcinoma	TCGA-KK-A6E1	1	1	1	1	1
Prostate Adenocarcinoma	TCGA-KK-A6E2	0	0	0	0	1
Prostate Adenocarcinoma	TCGA-KK-A6E3	0	0	0	0	0
Prostate Adenocarcinoma	TCGA-KK-A6E4	0	1	1	1	1
Prostate Adenocarcinoma	TCGA-KK-A6E5	1	1	1	0	1
Prostate Adenocarcinoma	TCGA-KK-A6E6	1	1	1	1	1
Prostate Adenocarcinoma	TCGA-KK-A6E7	1	1	1	1	1
Prostate Adenocarcinoma	TCGA-KK-A6E8	0	1	0	0	1
Prostate Adenocarcinoma	TCGA-KK-A7AP	1	1	1	1	1
Prostate Adenocarcinoma	TCGA-KK-A7AQ	1	1	1	1	1
Prostate Adenocarcinoma	TCGA-KK-A7AU	1	1	0	0	1
Prostate Adenocarcinoma	TCGA-KK-A7AV	0	0	1	0	0
Prostate Adenocarcinoma	TCGA-KK-A7AW	1	1	1	1	1
Prostate Adenocarcinoma	TCGA-KK-A7AY	1	1	0	1	1
Prostate Adenocarcinoma	TCGA-KK-A7AZ	1	1	1	1	1
Prostate Adenocarcinoma	TCGA-KK-A7B0	1	0	1	1	1
Prostate Adenocarcinoma	TCGA-KK-A7B1	1	0	1	0	1
Prostate Adenocarcinoma	TCGA-KK-A7B2	0	0	0	0	0
Prostate Adenocarcinoma	TCGA-KK-A7B3	1	1	1	1	1
Prostate Adenocarcinoma	TCGA-KK-A7B4	1	1	1	1	1
Prostate Adenocarcinoma	TCGA-KK-A8I4	1	0	1	0	0
Prostate Adenocarcinoma	TCGA-KK-A8I5	1	1	1	0	1
Prostate Adenocarcinoma	TCGA-KK-A8I6	1	1	1	0	1
Prostate Adenocarcinoma	TCGA-KK-A8I7	1	1	1	0	1
Prostate Adenocarcinoma	TCGA-KK-A8I8	1	0	1	0	1
Prostate Adenocarcinoma	TCGA-KK-A8I9	1	0	1	0	1
Prostate Adenocarcinoma	TCGA-KK-A8IA	1	1	1	1	1
Prostate Adenocarcinoma	TCGA-KK-A8IB	1	1	1	1	1
Prostate Adenocarcinoma	TCGA-KK-A8IC	1	1	1	0	1
Prostate Adenocarcinoma	TCGA-KK-A8ID	1	1	1	0	1
Prostate Adenocarcinoma	TCGA-KK-A8IF	1	1	1	1	1
Prostate Adenocarcinoma	TCGA-KK-A8IG	1	1	1	1	1
Prostate Adenocarcinoma	TCGA-KK-A8IH	1	1	1	0	1
Prostate Adenocarcinoma	TCGA-KK-A8II	1	1	1	0	1
Prostate Adenocarcinoma	TCGA-KK-A8IJ	0	1	1	0	1
Prostate Adenocarcinoma	TCGA-KK-A8IK	1	1	1	1	1
Prostate Adenocarcinoma	TCGA-KK-A8IL	1	0	0	0	1
Prostate Adenocarcinoma	TCGA-KK-A8IM	1	1	0	0	1
Prostate Adenocarcinoma	TCGA-M7-A71Y	0	0	0	1	1
Prostate Adenocarcinoma	TCGA-M7-A71Z	0	1	1	0	1
Prostate Adenocarcinoma	TCGA-M7-A720	0	0	0	0	1
Prostate Adenocarcinoma	TCGA-M7-A721	1	0	1	0	1
Prostate Adenocarcinoma	TCGA-M7-A722	0	0	1	0	0
Prostate Adenocarcinoma	TCGA-M7-A723	0	0	0	0	0
Prostate Adenocarcinoma	TCGA-M7-A724	1	0	1	0	1
Prostate Adenocarcinoma	TCGA-M7-A725	1	1	1	1	1
Prostate Adenocarcinoma	TCGA-MG-AAMC	1	1	1	0	1
Prostate Adenocarcinoma	TCGA-QU-A6IL	0	1	1	1	0
Prostate Adenocarcinoma	TCGA-QU-A6IM	0	0	0	0	0
Prostate Adenocarcinoma	TCGA-QU-A6IN	1	0	0	1	1
Prostate Adenocarcinoma	TCGA-QU-A6IO	1	1	1	0	0
Prostate Adenocarcinoma	TCGA-QU-A6IP	0	0	0	0	0
Prostate Adenocarcinoma	TCGA-SU-A7E7	1	0	0	0	1
Prostate Adenocarcinoma	TCGA-TK-A8OK	1	1	1	0	1
Prostate Adenocarcinoma	TCGA-TP-A8TT	1	0	0	0	1

Prostate Adenocarcinoma	TCGA-TP-A8TV	1	1	1	1	1
Prostate Adenocarcinoma	TCGA-V1-A8MF	1	0	1	0	1
Prostate Adenocarcinoma	TCGA-V1-A8MG	1	0	1	0	1
Prostate Adenocarcinoma	TCGA-V1-A8MJ	1	1	0	1	1
Prostate Adenocarcinoma	TCGA-V1-A8MK	0	0	0	0	0
Prostate Adenocarcinoma	TCGA-V1-A8ML	0	0	1	0	1
Prostate Adenocarcinoma	TCGA-V1-A8MM	0	1	1	1	0
Prostate Adenocarcinoma	TCGA-V1-A8MU	1	1	1	1	1
Prostate Adenocarcinoma	TCGA-V1-A8WL	1	1	0	1	0
Prostate Adenocarcinoma	TCGA-V1-A8WN	0	0	0	0	1
Prostate Adenocarcinoma	TCGA-V1-A8WS	1	1	1	1	1
Prostate Adenocarcinoma	TCGA-V1-A8WV	1	1	1	1	1
Prostate Adenocarcinoma	TCGA-V1-A8WW	1	1	1	1	1
Prostate Adenocarcinoma	TCGA-V1-A8X3	0	1	0	1	1
Prostate Adenocarcinoma	TCGA-V1-A9O5	1	1	1	1	1
Prostate Adenocarcinoma	TCGA-V1-A9O5	0	0	0	0	0
Prostate Adenocarcinoma	TCGA-V1-A9O7	1	1	1	1	1
Prostate Adenocarcinoma	TCGA-V1-A9O9	1	0	1	0	1
Prostate Adenocarcinoma	TCGA-V1-A9OA	1	1	1	1	1
Prostate Adenocarcinoma	TCGA-V1-A9OF	0	0	1	0	0
Prostate Adenocarcinoma	TCGA-V1-A9OH	0	0	1	0	0
Prostate Adenocarcinoma	TCGA-V1-A9OL	1	1	1	1	1
Prostate Adenocarcinoma	TCGA-V1-A9OQ	1	0	1	0	0
Prostate Adenocarcinoma	TCGA-V1-A9OT	1	0	1	1	1
Prostate Adenocarcinoma	TCGA-V1-A9OX	1	0	1	0	1
Prostate Adenocarcinoma	TCGA-V1-A9OY	0	0	0	0	1
Prostate Adenocarcinoma	TCGA-V1-A9Z7	1	1	1	1	1
Prostate Adenocarcinoma	TCGA-V1-A9Z8	1	1	0	0	0
Prostate Adenocarcinoma	TCGA-V1-A9Z9	1	0	1	0	0
Prostate Adenocarcinoma	TCGA-V1-A9ZG	1	0	0	0	1
Prostate Adenocarcinoma	TCGA-V1-A9ZI	1	1	1	1	1
Prostate Adenocarcinoma	TCGA-V1-A9ZK	1	1	1	1	1
Prostate Adenocarcinoma	TCGA-V1-A9ZR	1	1	0	0	1
Prostate Adenocarcinoma	TCGA-VN-A88I	0	1	0	0	0
Prostate Adenocarcinoma	TCGA-VN-A88K	1	1	0	1	1
Prostate Adenocarcinoma	TCGA-VN-A88L	1	0	0	0	1
Prostate Adenocarcinoma	TCGA-VN-A88M	0	1	0	0	0
Prostate Adenocarcinoma	TCGA-VN-A88N	1	0	1	0	1
Prostate Adenocarcinoma	TCGA-VN-A88O	0	1	1	0	0
Prostate Adenocarcinoma	TCGA-VN-A88P	1	1	0	0	1
Prostate Adenocarcinoma	TCGA-VN-A88Q	1	0	0	0	1
Prostate Adenocarcinoma	TCGA-VN-A88R	1	1	1	0	1
Prostate Adenocarcinoma	TCGA-VN-A943	1	0	0	0	0
Prostate Adenocarcinoma	TCGA-VP-A872	1	0	1	1	1
Prostate Adenocarcinoma	TCGA-VP-A875	1	1	1	1	1
Prostate Adenocarcinoma	TCGA-VP-A876	1	1	1	1	1
Prostate Adenocarcinoma	TCGA-VP-A878	0	0	1	0	0
Prostate Adenocarcinoma	TCGA-VP-A879	0	0	0	0	0
Prostate Adenocarcinoma	TCGA-VP-A87B	1	1	1	1	0
Prostate Adenocarcinoma	TCGA-VP-A87C	1	1	1	0	1
Prostate Adenocarcinoma	TCGA-VP-A87D	1	1	1	0	1
Prostate Adenocarcinoma	TCGA-VP-A87E	0	0	0	0	0
Prostate Adenocarcinoma	TCGA-VP-A87H	1	0	1	0	1
Prostate Adenocarcinoma	TCGA-VP-A87J	1	1	1	1	1
Prostate Adenocarcinoma	TCGA-VP-A87K	1	1	1	1	1
Prostate Adenocarcinoma	TCGA-VP-AA1N	1	1	1	1	1
Prostate Adenocarcinoma	TCGA-WW-A8ZI	1	0	0	0	1
Prostate Adenocarcinoma	TCGA-X4-A8KQ	1	1	1	1	1

Prostate Adenocarcinoma	TCGA-X4-A8KS	0	0	1	0	0
Prostate Adenocarcinoma	TCGA-XA-A8JR	0	0	1	0	0
Prostate Adenocarcinoma	TCGA-XJ-A83F	1	1	1	0	1
Prostate Adenocarcinoma	TCGA-XJ-A83G	1	1	1	0	1
Prostate Adenocarcinoma	TCGA-XJ-A83H	1	1	1	1	0
Prostate Adenocarcinoma	TCGA-XJ-A9DI	1	0	1	0	1
Prostate Adenocarcinoma	TCGA-XJ-A9DK	0	0	0	0	0
Prostate Adenocarcinoma	TCGA-XJ-A9DQ	0	1	0	0	0
Prostate Adenocarcinoma	TCGA-XJ-A9DX	1	1	1	1	1
Prostate Adenocarcinoma	TCGA-XK-AAIR	1	1	1	0	0
Prostate Adenocarcinoma	TCGA-XK-AAIV	1	1	1	1	1
Prostate Adenocarcinoma	TCGA-XK-AAIW	1	1	1	1	1
Prostate Adenocarcinoma	TCGA-XK-AAJ3	1	0	1	0	0
Prostate Adenocarcinoma	TCGA-XK-AAJA	1	1	1	1	1
Prostate Adenocarcinoma	TCGA-XK-AAJP	1	0	1	0	1
Prostate Adenocarcinoma	TCGA-XK-AAJR	0	1	1	0	1
Prostate Adenocarcinoma	TCGA-XK-AAJT	1	0	1	1	1
Prostate Adenocarcinoma	TCGA-XK-AAJU	0	0	0	0	0
Prostate Adenocarcinoma	TCGA-XK-AAK1	0	0	1	0	0
Prostate Adenocarcinoma	TCGA-XQ-A8TA	1	1	1	1	1
Prostate Adenocarcinoma	TCGA-XQ-A8TB	1	0	0	1	1
Prostate Adenocarcinoma	TCGA-Y6-A8TL	0	0	1	0	0
Prostate Adenocarcinoma	TCGA-Y6-A9XI	1	0	1	0	1
Prostate Adenocarcinoma	TCGA-YJ-A8SW	1	1	0	1	1
Prostate Adenocarcinoma	TCGA-YL-A8HJ	0	0	1	0	1
Prostate Adenocarcinoma	TCGA-YL-A8HK	1	1	1	0	1
Prostate Adenocarcinoma	TCGA-YL-A8HL	1	0	1	1	1
Prostate Adenocarcinoma	TCGA-YL-A8HM	1	1	1	1	1
Prostate Adenocarcinoma	TCGA-YL-A8HO	1	1	1	1	1
Prostate Adenocarcinoma	TCGA-YL-A8S8	1	0	1	0	1
Prostate Adenocarcinoma	TCGA-YL-A8S9	0	1	0	0	1
Prostate Adenocarcinoma	TCGA-YL-A8SA	1	0	1	0	1
Prostate Adenocarcinoma	TCGA-YL-A8SB	1	1	1	1	1
Prostate Adenocarcinoma	TCGA-YL-A8SC	1	1	1	0	1
Prostate Adenocarcinoma	TCGA-YL-A8SF	1	1	0	0	1
Prostate Adenocarcinoma	TCGA-YL-A8SH	1	0	1	0	1
Prostate Adenocarcinoma	TCGA-YL-A8SI	0	0	0	0	0
Prostate Adenocarcinoma	TCGA-YL-A8SJ	1	1	1	1	1
Prostate Adenocarcinoma	TCGA-YL-A8SK	1	1	1	0	1
Prostate Adenocarcinoma	TCGA-YL-A8SL	1	1	1	1	1
Prostate Adenocarcinoma	TCGA-YL-A8SO	1	0	1	0	1
Prostate Adenocarcinoma	TCGA-YL-A8SP	1	0	0	1	1
Prostate Adenocarcinoma	TCGA-YL-A8SQ	1	1	1	1	1
Prostate Adenocarcinoma	TCGA-YL-A8SR	1	1	1	0	1
Prostate Adenocarcinoma	TCGA-YL-A9WH	1	1	1	1	1
Prostate Adenocarcinoma	TCGA-YL-A9WI	0	1	1	0	0
Prostate Adenocarcinoma	TCGA-YL-A9WJ	1	1	1	0	0
Prostate Adenocarcinoma	TCGA-YL-A9WK	1	1	1	1	1
Prostate Adenocarcinoma	TCGA-YL-A9WL	1	1	1	1	1
Prostate Adenocarcinoma	TCGA-YL-A9WX	1	1	0	0	1
Prostate Adenocarcinoma	TCGA-YL-A9WY	1	1	1	1	1
Prostate Adenocarcinoma	TCGA-ZG-A8QW	1	0	1	1	1
Prostate Adenocarcinoma	TCGA-ZG-A8QX	0	0	1	0	0
Prostate Adenocarcinoma	TCGA-ZG-A8QY	0	0	1	1	0
Prostate Adenocarcinoma	TCGA-ZG-A8QZ	1	1	1	1	1
Prostate Adenocarcinoma	TCGA-ZG-A9KY	1	1	1	1	1
Prostate Adenocarcinoma	TCGA-ZG-A9L0	1	1	1	1	1
Prostate Adenocarcinoma	TCGA-ZG-A9L1	1	0	1	1	1

Prostate Adenocarcinoma	TCGA-ZG-A9L2	1	0	1	1	0
Prostate Adenocarcinoma	TCGA-ZG-A9L4	1	1	1	0	1
Prostate Adenocarcinoma	TCGA-ZG-A9L5	1	1	1	1	1
Prostate Adenocarcinoma	TCGA-ZG-A9L6	1	0	1	0	1
Prostate Adenocarcinoma	TCGA-ZG-A9L9	1	1	1	1	1
Prostate Adenocarcinoma	TCGA-ZG-A9LB	1	1	1	1	1
Prostate Adenocarcinoma	TCGA-ZG-A9LM	1	1	1	1	1
Prostate Adenocarcinoma	TCGA-ZG-A9LN	1	0	1	0	1
Prostate Adenocarcinoma	TCGA-ZG-A9LS	1	1	1	0	1
Prostate Adenocarcinoma	TCGA-ZG-A9LU	1	1	1	1	1
Prostate Adenocarcinoma	TCGA-ZG-A9LY	1	1	0	1	1
Prostate Adenocarcinoma	TCGA-ZG-A9LZ	1	1	1	1	1
Prostate Adenocarcinoma	TCGA-ZG-A9M4	1	1	1	1	1
Prostate Adenocarcinoma	TCGA-ZG-A9MC	1	1	1	1	1
Prostate Adenocarcinoma	TCGA-ZG-A9N3	1	1	1	1	1
Prostate Adenocarcinoma	TCGA-ZG-A9ND	1	1	1	1	1
Prostate Adenocarcinoma	TCGA-ZG-A9NI	1	1	1	1	1
Sarcoma	TCGA-3B-A9HI	1	1	1	0	1
Sarcoma	TCGA-3B-A9HJ	0	0	1	0	0
Sarcoma	TCGA-3B-A9HL	1	1	1	1	1
Sarcoma	TCGA-3B-A9HO	1	1	1	1	1
Sarcoma	TCGA-3B-A9HP	1	0	1	0	1
Sarcoma	TCGA-3B-A9HQ	1	1	0	1	1
Sarcoma	TCGA-3B-A9HR	1	1	1	1	1
Sarcoma	TCGA-3B-A9HS	1	1	1	1	1
Sarcoma	TCGA-3B-A9HT	0	0	0	0	1
Sarcoma	TCGA-3B-A9HU	1	1	1	0	1
Sarcoma	TCGA-3B-A9HV	1	1	1	1	1
Sarcoma	TCGA-3B-A9HX	1	1	1	0	1
Sarcoma	TCGA-3B-A9HY	1	1	1	1	1
Sarcoma	TCGA-3B-A9HZ	1	1	1	0	1
Sarcoma	TCGA-3B-A9I0	1	1	1	0	1
Sarcoma	TCGA-3B-A9I1	1	1	1	1	1
Sarcoma	TCGA-3B-A9I3	1	1	1	1	1
Sarcoma	TCGA-3R-A8YX	1	1	1	1	1
Sarcoma	TCGA-DX-A1KU	1	1	0	1	1
Sarcoma	TCGA-DX-A1KW	1	1	1	1	1
Sarcoma	TCGA-DX-A1KX	1	1	1	1	1
Sarcoma	TCGA-DX-A1KY	1	1	1	1	1
Sarcoma	TCGA-DX-A1KZ	1	1	1	0	1
Sarcoma	TCGA-DX-A1L0	1	1	1	1	1
Sarcoma	TCGA-DX-A1L1	1	0	1	1	1
Sarcoma	TCGA-DX-A1L2	1	1	1	1	1
Sarcoma	TCGA-DX-A1L3	1	0	1	1	0
Sarcoma	TCGA-DX-A1L4	0	0	0	1	0
Sarcoma	TCGA-DX-A23R	1	1	1	1	1
Sarcoma	TCGA-DX-A23T	1	1	0	1	1
Sarcoma	TCGA-DX-A23U	0	0	0	1	0
Sarcoma	TCGA-DX-A23V	0	1	0	1	1
Sarcoma	TCGA-DX-A23Y	1	1	1	1	1
Sarcoma	TCGA-DX-A23Z	0	0	1	0	1
Sarcoma	TCGA-DX-A240	0	0	0	0	0
Sarcoma	TCGA-DX-A2IZ	1	0	0	1	1
Sarcoma	TCGA-DX-A2J0	1	1	1	0	1
Sarcoma	TCGA-DX-A2J1	0	0	0	0	1
Sarcoma	TCGA-DX-A2J4	0	0	0	0	1
Sarcoma	TCGA-DX-A3LS	1	1	1	1	1
Sarcoma	TCGA-DX-A3LT	1	1	1	1	1

Sarcoma	TCGA-DX-A3LU	1	1	0	1	0
Sarcoma	TCGA-DX-A3LW	1	1	1	1	1
Sarcoma	TCGA-DX-A3LY	1	1	1	1	1
Sarcoma	TCGA-DX-A3M1	1	1	1	1	1
Sarcoma	TCGA-DX-A3M2	1	1	1	1	1
Sarcoma	TCGA-DX-A3U5	1	0	1	0	1
Sarcoma	TCGA-DX-A3U6	0	1	0	1	0
Sarcoma	TCGA-DX-A3U7	1	1	1	1	1
Sarcoma	TCGA-DX-A3U8	0	1	0	0	1
Sarcoma	TCGA-DX-A3U9	1	1	1	1	1
Sarcoma	TCGA-DX-A3UA	1	1	1	1	1
Sarcoma	TCGA-DX-A3UB	0	1	0	0	0
Sarcoma	TCGA-DX-A3UC	1	1	1	0	1
Sarcoma	TCGA-DX-A3UD	1	1	1	1	1
Sarcoma	TCGA-DX-A3UE	1	1	1	0	1
Sarcoma	TCGA-DX-A3UF	1	1	1	1	1
Sarcoma	TCGA-DX-A48J	1	1	1	1	1
Sarcoma	TCGA-DX-A48K	1	1	1	1	1
Sarcoma	TCGA-DX-A48L	1	1	1	1	1
Sarcoma	TCGA-DX-A48N	1	0	1	1	0
Sarcoma	TCGA-DX-A48O	1	1	1	1	1
Sarcoma	TCGA-DX-A48P	1	1	1	1	1
Sarcoma	TCGA-DX-A48R	1	1	1	1	1
Sarcoma	TCGA-DX-A48U	1	1	1	1	1
Sarcoma	TCGA-DX-A6B7	1	1	1	1	1
Sarcoma	TCGA-DX-A6B8	1	1	1	1	1
Sarcoma	TCGA-DX-A6B9	1	1	1	1	1
Sarcoma	TCGA-DX-A6BA	1	1	1	1	1
Sarcoma	TCGA-DX-A6BB	1	1	1	1	1
Sarcoma	TCGA-DX-A6BE	0	0	0	1	0
Sarcoma	TCGA-DX-A6BF	1	1	1	1	1
Sarcoma	TCGA-DX-A6BG	1	1	0	1	0
Sarcoma	TCGA-DX-A6BH	0	0	0	1	1
Sarcoma	TCGA-DX-A6YQ	1	1	1	1	1
Sarcoma	TCGA-DX-A6YR	1	1	1	1	1
Sarcoma	TCGA-DX-A6YS	1	1	1	1	1
Sarcoma	TCGA-DX-A6YT	1	1	1	1	1
Sarcoma	TCGA-DX-A6YU	1	0	1	1	1
Sarcoma	TCGA-DX-A6YV	1	1	1	1	1
Sarcoma	TCGA-DX-A6YX	1	1	1	1	1
Sarcoma	TCGA-DX-A6YZ	1	1	1	1	1
Sarcoma	TCGA-DX-A6Z0	1	1	1	0	1
Sarcoma	TCGA-DX-A6Z2	1	1	1	0	1
Sarcoma	TCGA-DX-A7EF	1	1	1	1	1
Sarcoma	TCGA-DX-A7EI	1	1	1	1	1
Sarcoma	TCGA-DX-A7EL	1	1	1	1	1
Sarcoma	TCGA-DX-A7EM	1	1	1	0	1
Sarcoma	TCGA-DX-A7EN	1	1	1	1	1
Sarcoma	TCGA-DX-A7EO	1	0	1	1	1
Sarcoma	TCGA-DX-A7EQ	0	1	0	0	1
Sarcoma	TCGA-DX-A7ER	0	0	0	0	0
Sarcoma	TCGA-DX-A7ES	1	0	1	0	0
Sarcoma	TCGA-DX-A7ET	1	1	1	1	1
Sarcoma	TCGA-DX-A7EU	0	0	0	0	0
Sarcoma	TCGA-DX-A8BG	1	1	1	1	1
Sarcoma	TCGA-DX-A8BH	1	1	1	1	1
Sarcoma	TCGA-DX-A8BJ	1	1	1	1	1
Sarcoma	TCGA-DX-A8BK	1	0	1	1	1

Sarcoma	TCGA-DX-A8BL	1	1	1	1	1
Sarcoma	TCGA-DX-A8BM	1	1	1	1	1
Sarcoma	TCGA-DX-A8BN	1	1	1	1	1
Sarcoma	TCGA-DX-A8BO	1	1	1	1	1
Sarcoma	TCGA-DX-A8BP	1	1	1	1	1
Sarcoma	TCGA-DX-A8BQ	0	0	0	0	0
Sarcoma	TCGA-DX-A8BR	1	1	1	1	1
Sarcoma	TCGA-DX-A8BS	1	1	1	1	1
Sarcoma	TCGA-DX-A8BT	1	0	0	1	1
Sarcoma	TCGA-DX-A8BU	1	1	1	1	1
Sarcoma	TCGA-DX-A8BV	1	1	1	1	1
Sarcoma	TCGA-DX-A8BX	1	0	1	0	1
Sarcoma	TCGA-DX-A8BZ	1	1	1	1	1
Sarcoma	TCGA-DX-AATS	1	1	1	1	1
Sarcoma	TCGA-DX-AB2E	1	1	1	1	1
Sarcoma	TCGA-DX-AB2F	1	1	1	1	1
Sarcoma	TCGA-DX-AB2G	1	0	1	0	1
Sarcoma	TCGA-DX-AB2H	1	1	1	1	1
Sarcoma	TCGA-DX-AB2J	0	1	0	1	1
Sarcoma	TCGA-DX-AB2L	0	0	0	0	0
Sarcoma	TCGA-DX-AB2O	1	1	1	1	1
Sarcoma	TCGA-DX-AB2P	1	1	1	1	1
Sarcoma	TCGA-DX-AB2Q	1	1	1	1	1
Sarcoma	TCGA-DX-AB2S	0	0	0	0	0
Sarcoma	TCGA-DX-AB2T	1	1	1	1	1
Sarcoma	TCGA-DX-AB2V	1	1	1	1	1
Sarcoma	TCGA-DX-AB2W	1	1	1	1	1
Sarcoma	TCGA-DX-AB2X	1	1	1	1	1
Sarcoma	TCGA-DX-AB2Z	1	1	1	1	1
Sarcoma	TCGA-DX-AB30	0	0	0	0	1
Sarcoma	TCGA-DX-AB32	1	1	1	1	1
Sarcoma	TCGA-DX-AB35	1	1	1	1	1
Sarcoma	TCGA-DX-AB36	1	1	0	1	1
Sarcoma	TCGA-DX-AB37	1	0	1	1	1
Sarcoma	TCGA-DX-AB3A	1	1	1	1	1
Sarcoma	TCGA-DX-AB3B	0	0	0	0	1
Sarcoma	TCGA-DX-AB3C	1	0	0	1	0
Sarcoma	TCGA-FX-A2QS	1	1	1	1	1
Sarcoma	TCGA-FX-A3NJ	1	1	1	0	1
Sarcoma	TCGA-FX-A3NK	1	1	1	1	1
Sarcoma	TCGA-FX-A3RE	1	1	0	1	1
Sarcoma	TCGA-FX-A3TO	1	1	1	1	1
Sarcoma	TCGA-FX-A48G	1	1	1	1	1
Sarcoma	TCGA-FX-A76Y	1	1	1	0	1
Sarcoma	TCGA-FX-A8OO	0	0	0	0	0
Sarcoma	TCGA-HB-A2OT	1	1	1	1	1
Sarcoma	TCGA-HB-A3L4	1	1	1	1	1
Sarcoma	TCGA-HB-A3YV	1	1	1	1	1
Sarcoma	TCGA-HB-A43Z	1	1	1	1	1
Sarcoma	TCGA-HB-A5W3	1	1	1	1	1
Sarcoma	TCGA-HS-A5N7	1	1	1	0	1
Sarcoma	TCGA-HS-A5N8	1	1	1	1	1
Sarcoma	TCGA-HS-A5N9	1	1	1	1	1
Sarcoma	TCGA-HS-A5NA	1	1	1	1	1
Sarcoma	TCGA-IE-A3OV	1	1	1	0	1
Sarcoma	TCGA-IE-A4EH	1	1	1	1	1
Sarcoma	TCGA-IE-A4EI	1	1	0	0	1
Sarcoma	TCGA-IE-A4EJ	1	1	1	1	1

Sarcoma	TCGA-IE-A4EK	1	1	1	0	1
Sarcoma	TCGA-IE-A6BZ	1	1	1	1	1
Sarcoma	TCGA-IF-A3RQ	1	0	1	1	1
Sarcoma	TCGA-IF-A4AJ	1	1	1	1	1
Sarcoma	TCGA-IF-A4AK	1	1	1	1	1
Sarcoma	TCGA-IS-A3K6	1	0	1	0	1
Sarcoma	TCGA-IS-A3K7	1	1	1	1	1
Sarcoma	TCGA-IS-A3K8	1	1	0	1	1
Sarcoma	TCGA-IS-A3KA	1	1	1	1	1
Sarcoma	TCGA-IW-A3M4	1	1	0	1	1
Sarcoma	TCGA-IW-A3M5	1	1	1	1	1
Sarcoma	TCGA-IW-A3M6	1	1	1	1	1
Sarcoma	TCGA-JV-A5VE	1	1	1	1	1
Sarcoma	TCGA-JV-A5VF	1	1	1	1	1
Sarcoma	TCGA-JV-A75J	1	1	1	1	1
Sarcoma	TCGA-K1-A3PN	1	1	1	1	1
Sarcoma	TCGA-K1-A3PN	0	0	0	0	0
Sarcoma	TCGA-K1-A3PO	1	0	1	0	0
Sarcoma	TCGA-K1-A42W	1	1	1	1	1
Sarcoma	TCGA-K1-A42X	1	1	1	1	1
Sarcoma	TCGA-K1-A42X	0	0	0	0	0
Sarcoma	TCGA-K1-A6RT	0	0	0	0	1
Sarcoma	TCGA-K1-A6RU	1	1	1	1	1
Sarcoma	TCGA-K1-A6RV	1	1	1	1	1
Sarcoma	TCGA-KD-A5QS	1	1	1	1	1
Sarcoma	TCGA-KD-A5QT	0	1	1	1	1
Sarcoma	TCGA-KD-A5QU	1	1	1	1	1
Sarcoma	TCGA-KF-A41W	1	1	1	1	1
Sarcoma	TCGA-LI-A67I	1	1	1	1	1
Sarcoma	TCGA-LI-A9QH	1	1	1	1	1
Sarcoma	TCGA-MB-A5Y8	1	1	1	1	1
Sarcoma	TCGA-MB-A5Y9	1	1	0	0	1
Sarcoma	TCGA-MB-A5YA	1	0	1	0	1
Sarcoma	TCGA-MB-A8JK	1	1	1	0	1
Sarcoma	TCGA-MB-A8JL	1	1	1	1	1
Sarcoma	TCGA-MJ-A68H	1	1	1	1	1
Sarcoma	TCGA-MJ-A68J	0	0	0	1	1
Sarcoma	TCGA-MJ-A850	0	0	0	0	0
Sarcoma	TCGA-MO-A47P	0	0	0	0	0
Sarcoma	TCGA-MO-A47R	1	1	0	1	1
Sarcoma	TCGA-N1-A6IA	1	1	1	1	1
Sarcoma	TCGA-PC-A5DK	1	1	0	0	1
Sarcoma	TCGA-PC-A5DL	1	1	1	1	1
Sarcoma	TCGA-PC-A5DM	1	1	1	1	1
Sarcoma	TCGA-PC-A5DN	0	1	0	0	1
Sarcoma	TCGA-PC-A5DO	1	1	1	1	1
Sarcoma	TCGA-PC-A5DP	1	1	1	1	1
Sarcoma	TCGA-PT-A8TR	0	0	0	0	0
Sarcoma	TCGA-QC-A6FX	1	1	1	0	1
Sarcoma	TCGA-QC-A7B5	1	1	1	1	1
Sarcoma	TCGA-QC-AA9N	1	1	1	1	1
Sarcoma	TCGA-QQ-A5V2	0	0	0	0	0
Sarcoma	TCGA-QQ-A5V9	1	1	1	0	1
Sarcoma	TCGA-QQ-A5VA	1	1	1	1	1
Sarcoma	TCGA-QQ-A5VB	1	1	1	0	1
Sarcoma	TCGA-QQ-A5VC	1	1	1	1	1
Sarcoma	TCGA-QQ-A5VD	1	1	1	1	1
Sarcoma	TCGA-QQ-A8VB	1	1	1	1	1

Sarcoma	TCGA-QQ-A8VD	0	0	0	0	0
Sarcoma	TCGA-QQ-A8VF	1	1	1	1	1
Sarcoma	TCGA-QQ-A8VG	0	0	0	0	1
Sarcoma	TCGA-QQ-A8VH	1	1	1	1	1
Sarcoma	TCGA-RN-A68Q	0	0	0	0	0
Sarcoma	TCGA-RN-AAAQ	1	1	1	1	1
Sarcoma	TCGA-SG-A6Z4	1	1	1	1	1
Sarcoma	TCGA-SG-A6Z7	1	1	1	1	1
Sarcoma	TCGA-SG-A849	1	1	1	1	1
Sarcoma	TCGA-SI-A71O	1	1	1	0	1
Sarcoma	TCGA-SI-A71O	0	0	0	0	0
Sarcoma	TCGA-SI-A71P	1	0	1	1	1
Sarcoma	TCGA-SI-A71Q	1	1	1	1	1
Sarcoma	TCGA-SI-AA8B	1	1	1	1	1
Sarcoma	TCGA-SI-AA8C	1	1	1	1	1
Sarcoma	TCGA-UE-A6QT	1	0	0	0	1
Sarcoma	TCGA-UE-A6QU	1	1	1	1	1
Sarcoma	TCGA-VT-A80G	1	1	1	0	1
Sarcoma	TCGA-VT-A80J	1	1	1	1	1
Sarcoma	TCGA-VT-A80J	0	0	0	0	0
Sarcoma	TCGA-VT-AB3D	1	1	1	1	1
Sarcoma	TCGA-WK-A8XO	1	0	1	1	1
Sarcoma	TCGA-WK-A8XQ	1	0	0	0	0
Sarcoma	TCGA-WK-A8XS	1	1	1	1	1
Sarcoma	TCGA-WK-A8XT	0	1	0	1	0
Sarcoma	TCGA-WK-A8XX	1	1	1	1	1
Sarcoma	TCGA-WK-A8XY	1	1	1	1	1
Sarcoma	TCGA-WK-A8XZ	0	0	0	0	0
Sarcoma	TCGA-WK-A8Y0	1	1	1	1	1
Sarcoma	TCGA-WP-A9GB	1	1	1	1	1
Sarcoma	TCGA-X2-A95T	1	0	1	1	1
Sarcoma	TCGA-X6-A7W8	1	1	1	1	1
Sarcoma	TCGA-X6-A7WA	1	1	1	1	1
Sarcoma	TCGA-X6-A7WB	1	1	1	1	1
Sarcoma	TCGA-X6-A7WC	1	1	1	1	1
Sarcoma	TCGA-X6-A7WD	1	1	1	1	1
Sarcoma	TCGA-X6-A8C2	1	1	1	1	1
Sarcoma	TCGA-X6-A8C3	1	1	1	0	1
Sarcoma	TCGA-X6-A8C4	1	1	1	1	1
Sarcoma	TCGA-X6-A8C5	1	1	1	1	1
Sarcoma	TCGA-X6-A8C6	1	1	1	1	1
Sarcoma	TCGA-X6-A8C7	0	0	0	1	1
Sarcoma	TCGA-X9-A971	1	1	1	1	1
Sarcoma	TCGA-X9-A973	0	1	0	1	1
Sarcoma	TCGA-Z4-A8JB	0	0	1	0	0
Sarcoma	TCGA-Z4-A9VC	1	0	0	0	0
Sarcoma	TCGA-Z4-AAPF	0	1	1	1	1
Sarcoma	TCGA-Z4-AAPG	1	1	1	1	1
Skin Cuteneous Melanoma	TCGA-3N-A9WB	0	0	1	1	1
Skin Cuteneous Melanoma	TCGA-3N-A9WC	1	1	1	1	1
Skin Cuteneous Melanoma	TCGA-3N-A9WD	1	1	1	1	1
Skin Cuteneous Melanoma	TCGA-BF-A1PU	0	0	0	0	0
Skin Cuteneous Melanoma	TCGA-BF-A1PV	0	0	0	0	0
Skin Cuteneous Melanoma	TCGA-BF-A1PX	0	0	0	0	0
Skin Cuteneous Melanoma	TCGA-BF-A1PZ	0	0	0	0	0
Skin Cuteneous Melanoma	TCGA-BF-A1Q0	0	0	0	0	0
Skin Cuteneous Melanoma	TCGA-BF-A3DJ	0	0	0	0	0
Skin Cuteneous Melanoma	TCGA-BF-A3DL	0	0	0	0	0

Skin Cuteneous Melanoma	TCGA-BF-A3DM	0	0	0	0	0
Skin Cuteneous Melanoma	TCGA-BF-A3DN	0	0	0	0	0
Skin Cuteneous Melanoma	TCGA-BF-A5EO	0	0	0	0	0
Skin Cuteneous Melanoma	TCGA-BF-A5EP	0	0	0	0	0
Skin Cuteneous Melanoma	TCGA-BF-A5EQ	0	0	0	0	0
Skin Cuteneous Melanoma	TCGA-BF-A5ER	0	0	0	0	0
Skin Cuteneous Melanoma	TCGA-BF-A5ES	0	0	0	0	0
Skin Cuteneous Melanoma	TCGA-BF-A9VF	0	0	0	0	0
Skin Cuteneous Melanoma	TCGA-BF-AAOU	0	0	0	0	0
Skin Cuteneous Melanoma	TCGA-BF-AAOX	0	0	0	0	0
Skin Cuteneous Melanoma	TCGA-BF-AAP0	1	1	0	1	1
Skin Cuteneous Melanoma	TCGA-BF-AAP1	0	0	0	0	0
Skin Cuteneous Melanoma	TCGA-BF-AAP2	0	0	0	0	0
Skin Cuteneous Melanoma	TCGA-BF-AAP4	0	0	0	0	0
Skin Cuteneous Melanoma	TCGA-BF-AAP6	0	0	0	0	0
Skin Cuteneous Melanoma	TCGA-BF-AAP7	0	0	0	0	0
Skin Cuteneous Melanoma	TCGA-BF-AAP8	0	0	0	0	0
Skin Cuteneous Melanoma	TCGA-D3-A1Q1	1	1	1	1	1
Skin Cuteneous Melanoma	TCGA-D3-A1Q3	1	1	1	0	1
Skin Cuteneous Melanoma	TCGA-D3-A1Q4	1	1	1	1	1
Skin Cuteneous Melanoma	TCGA-D3-A1Q5	1	1	1	1	1
Skin Cuteneous Melanoma	TCGA-D3-A1Q6	1	1	1	1	1
Skin Cuteneous Melanoma	TCGA-D3-A1Q7	1	0	0	1	1
Skin Cuteneous Melanoma	TCGA-D3-A1Q8	1	1	1	1	1
Skin Cuteneous Melanoma	TCGA-D3-A1Q9	1	1	1	1	1
Skin Cuteneous Melanoma	TCGA-D3-A1QA	0	0	0	0	0
Skin Cuteneous Melanoma	TCGA-D3-A1QA	1	1	1	1	1
Skin Cuteneous Melanoma	TCGA-D3-A1QB	1	1	1	1	1
Skin Cuteneous Melanoma	TCGA-D3-A2J6	1	1	1	0	1
Skin Cuteneous Melanoma	TCGA-D3-A2J7	1	1	1	1	1
Skin Cuteneous Melanoma	TCGA-D3-A2J8	1	0	0	1	1
Skin Cuteneous Melanoma	TCGA-D3-A2J9	0	0	0	0	0
Skin Cuteneous Melanoma	TCGA-D3-A2JA	1	1	1	1	1
Skin Cuteneous Melanoma	TCGA-D3-A2JB	0	1	1	1	1
Skin Cuteneous Melanoma	TCGA-D3-A2JC	1	1	1	1	1
Skin Cuteneous Melanoma	TCGA-D3-A2JD	1	1	1	1	1
Skin Cuteneous Melanoma	TCGA-D3-A2JE	1	0	1	1	1
Skin Cuteneous Melanoma	TCGA-D3-A2JF	1	1	1	1	1
Skin Cuteneous Melanoma	TCGA-D3-A2JG	1	1	1	1	1
Skin Cuteneous Melanoma	TCGA-D3-A2JH	0	1	1	1	0
Skin Cuteneous Melanoma	TCGA-D3-A2JK	1	1	1	1	1
Skin Cuteneous Melanoma	TCGA-D3-A2JL	1	1	1	1	1
Skin Cuteneous Melanoma	TCGA-D3-A2JN	1	1	1	1	1
Skin Cuteneous Melanoma	TCGA-D3-A2JO	1	1	1	1	1
Skin Cuteneous Melanoma	TCGA-D3-A2JP	1	1	1	1	1
Skin Cuteneous Melanoma	TCGA-D3-A3BZ	0	1	0	1	0
Skin Cuteneous Melanoma	TCGA-D3-A3C1	1	1	1	1	1
Skin Cuteneous Melanoma	TCGA-D3-A3C3	1	1	1	1	1
Skin Cuteneous Melanoma	TCGA-D3-A3C6	1	1	1	1	1
Skin Cuteneous Melanoma	TCGA-D3-A3C7	1	1	0	1	1
Skin Cuteneous Melanoma	TCGA-D3-A3C8	0	1	0	0	1
Skin Cuteneous Melanoma	TCGA-D3-A3CB	0	0	0	1	1
Skin Cuteneous Melanoma	TCGA-D3-A3CC	1	1	1	1	1
Skin Cuteneous Melanoma	TCGA-D3-A3CE	1	1	1	1	1
Skin Cuteneous Melanoma	TCGA-D3-A3CF	1	1	1	1	1
Skin Cuteneous Melanoma	TCGA-D3-A3ML	1	1	1	1	1
Skin Cuteneous Melanoma	TCGA-D3-A3MO	1	1	0	1	1
Skin Cuteneous Melanoma	TCGA-D3-A3MR	1	1	1	1	1

Skin Cuteneous Melanoma	TCGA-D3-A3MU	1	1	1	1	1
Skin Cuteneous Melanoma	TCGA-D3-A3MV	1	1	1	1	1
Skin Cuteneous Melanoma	TCGA-D3-A51E	0	1	1	1	1
Skin Cuteneous Melanoma	TCGA-D3-A51F	1	0	0	1	1
Skin Cuteneous Melanoma	TCGA-D3-A51G	1	0	1	1	1
Skin Cuteneous Melanoma	TCGA-D3-A51H	1	1	0	0	0
Skin Cuteneous Melanoma	TCGA-D3-A51J	1	1	1	1	1
Skin Cuteneous Melanoma	TCGA-D3-A51K	1	1	1	0	1
Skin Cuteneous Melanoma	TCGA-D3-A51N	1	1	1	1	1
Skin Cuteneous Melanoma	TCGA-D3-A51R	1	0	0	1	1
Skin Cuteneous Melanoma	TCGA-D3-A51T	1	1	1	1	1
Skin Cuteneous Melanoma	TCGA-D3-A5GL	1	1	1	1	1
Skin Cuteneous Melanoma	TCGA-D3-A5GN	1	1	1	1	1
Skin Cuteneous Melanoma	TCGA-D3-A5GO	1	1	1	1	1
Skin Cuteneous Melanoma	TCGA-D3-A5GR	1	1	1	1	1
Skin Cuteneous Melanoma	TCGA-D3-A5GS	1	1	1	1	1
Skin Cuteneous Melanoma	TCGA-D3-A5GT	0	0	0	0	0
Skin Cuteneous Melanoma	TCGA-D3-A5GU	1	1	0	1	1
Skin Cuteneous Melanoma	TCGA-D3-A8GB	1	1	1	1	1
Skin Cuteneous Melanoma	TCGA-D3-A8GC	1	1	1	1	1
Skin Cuteneous Melanoma	TCGA-D3-A8GD	1	1	1	1	1
Skin Cuteneous Melanoma	TCGA-D3-A8GE	1	0	0	0	1
Skin Cuteneous Melanoma	TCGA-D3-A8GI	1	1	1	0	1
Skin Cuteneous Melanoma	TCGA-D3-A8GJ	1	0	0	1	1
Skin Cuteneous Melanoma	TCGA-D3-A8GK	0	0	0	0	0
Skin Cuteneous Melanoma	TCGA-D3-A8GL	1	1	1	0	1
Skin Cuteneous Melanoma	TCGA-D3-A8GM	1	1	1	1	1
Skin Cuteneous Melanoma	TCGA-D3-A8GN	1	1	1	1	1
Skin Cuteneous Melanoma	TCGA-D3-A8GO	1	1	1	1	1
Skin Cuteneous Melanoma	TCGA-D3-A8GP	1	1	1	1	1
Skin Cuteneous Melanoma	TCGA-D3-A8GQ	0	0	0	0	1
Skin Cuteneous Melanoma	TCGA-D3-A8GR	1	1	1	1	1
Skin Cuteneous Melanoma	TCGA-D3-A8GS	1	1	1	0	1
Skin Cuteneous Melanoma	TCGA-D3-A8GV	1	1	1	1	1
Skin Cuteneous Melanoma	TCGA-D9-A148	1	1	1	1	1
Skin Cuteneous Melanoma	TCGA-D9-A149	1	1	1	1	1
Skin Cuteneous Melanoma	TCGA-D9-A1JW	1	1	1	1	1
Skin Cuteneous Melanoma	TCGA-D9-A1JX	1	1	1	1	1
Skin Cuteneous Melanoma	TCGA-D9-A1X3	1	1	1	1	1
Skin Cuteneous Melanoma	TCGA-D9-A3Z1	1	1	1	1	1
Skin Cuteneous Melanoma	TCGA-D9-A3Z3	1	1	1	1	1
Skin Cuteneous Melanoma	TCGA-D9-A3Z4	0	0	0	0	0
Skin Cuteneous Melanoma	TCGA-D9-A4Z2	0	0	0	0	0
Skin Cuteneous Melanoma	TCGA-D9-A4Z3	0	0	0	0	0
Skin Cuteneous Melanoma	TCGA-D9-A4Z5	0	0	0	0	0
Skin Cuteneous Melanoma	TCGA-D9-A4Z6	1	1	1	1	1
Skin Cuteneous Melanoma	TCGA-D9-A6E9	1	1	1	1	1
Skin Cuteneous Melanoma	TCGA-D9-A6EA	1	1	1	1	1
Skin Cuteneous Melanoma	TCGA-D9-A6EC	1	1	1	1	1
Skin Cuteneous Melanoma	TCGA-D9-A6EG	1	1	1	1	1
Skin Cuteneous Melanoma	TCGA-DA-A1HV	1	1	1	1	1
Skin Cuteneous Melanoma	TCGA-DA-A1HW	1	1	1	1	1
Skin Cuteneous Melanoma	TCGA-DA-A1HY	1	1	1	1	1
Skin Cuteneous Melanoma	TCGA-DA-A1I0	1	1	1	1	1
Skin Cuteneous Melanoma	TCGA-DA-A1I1	1	1	1	1	1
Skin Cuteneous Melanoma	TCGA-DA-A1I2	1	1	1	1	1
Skin Cuteneous Melanoma	TCGA-DA-A1I4	1	1	1	1	1
Skin Cuteneous Melanoma	TCGA-DA-A1I5	1	1	1	1	1

Skin Cuteneous Melanoma	TCGA-DA-A1I7	1	1	1	1	1
Skin Cuteneous Melanoma	TCGA-DA-A1I8	1	1	1	1	1
Skin Cuteneous Melanoma	TCGA-DA-A1IA	1	1	1	1	1
Skin Cuteneous Melanoma	TCGA-DA-A1IB	0	0	0	1	0
Skin Cuteneous Melanoma	TCGA-DA-A1IC	1	1	1	0	1
Skin Cuteneous Melanoma	TCGA-DA-A3F2	0	0	0	0	0
Skin Cuteneous Melanoma	TCGA-DA-A3F3	1	1	1	1	1
Skin Cuteneous Melanoma	TCGA-DA-A3F5	1	1	1	1	1
Skin Cuteneous Melanoma	TCGA-DA-A3F8	1	1	0	1	1
Skin Cuteneous Melanoma	TCGA-DA-A95V	1	1	1	1	1
Skin Cuteneous Melanoma	TCGA-DA-A95W	1	0	1	0	1
Skin Cuteneous Melanoma	TCGA-DA-A95X	1	1	1	1	1
Skin Cuteneous Melanoma	TCGA-DA-A95Y	1	1	1	1	1
Skin Cuteneous Melanoma	TCGA-DA-A95Z	1	1	1	1	1
Skin Cuteneous Melanoma	TCGA-DA-A960	0	0	0	0	0
Skin Cuteneous Melanoma	TCGA-EB-A1NK	0	0	0	0	0
Skin Cuteneous Melanoma	TCGA-EB-A24C	0	0	0	0	0
Skin Cuteneous Melanoma	TCGA-EB-A24D	0	0	0	0	0
Skin Cuteneous Melanoma	TCGA-EB-A299	0	0	0	0	0
Skin Cuteneous Melanoma	TCGA-EB-A3HV	0	0	0	0	0
Skin Cuteneous Melanoma	TCGA-EB-A3XB	0	0	0	0	0
Skin Cuteneous Melanoma	TCGA-EB-A3XC	0	0	0	0	0
Skin Cuteneous Melanoma	TCGA-EB-A3XD	0	0	0	0	0
Skin Cuteneous Melanoma	TCGA-EB-A3XE	0	0	0	0	0
Skin Cuteneous Melanoma	TCGA-EB-A3XF	0	0	0	0	0
Skin Cuteneous Melanoma	TCGA-EB-A3Y6	0	0	0	0	0
Skin Cuteneous Melanoma	TCGA-EB-A3Y7	0	0	0	0	0
Skin Cuteneous Melanoma	TCGA-EB-A41A	0	0	0	0	0
Skin Cuteneous Melanoma	TCGA-EB-A41B	0	0	0	0	0
Skin Cuteneous Melanoma	TCGA-EB-A42Y	0	0	0	0	0
Skin Cuteneous Melanoma	TCGA-EB-A42Z	0	0	0	0	0
Skin Cuteneous Melanoma	TCGA-EB-A430	0	0	0	0	0
Skin Cuteneous Melanoma	TCGA-EB-A431	0	0	0	0	0
Skin Cuteneous Melanoma	TCGA-EB-A44N	0	0	0	0	0
Skin Cuteneous Melanoma	TCGA-EB-A44O	0	0	0	0	0
Skin Cuteneous Melanoma	TCGA-EB-A44P	0	0	0	0	0
Skin Cuteneous Melanoma	TCGA-EB-A44Q	0	1	1	1	1
Skin Cuteneous Melanoma	TCGA-EB-A44R	1	1	1	1	1
Skin Cuteneous Melanoma	TCGA-EB-A4IQ	0	0	0	0	0
Skin Cuteneous Melanoma	TCGA-EB-A4IS	0	0	0	0	0
Skin Cuteneous Melanoma	TCGA-EB-A4OY	0	0	0	0	0
Skin Cuteneous Melanoma	TCGA-EB-A4OZ	0	0	0	0	0
Skin Cuteneous Melanoma	TCGA-EB-A4P0	0	0	0	0	0
Skin Cuteneous Melanoma	TCGA-EB-A4XL	0	0	0	0	0
Skin Cuteneous Melanoma	TCGA-EB-A51B	0	0	0	0	0
Skin Cuteneous Melanoma	TCGA-EB-A550	0	0	0	0	0
Skin Cuteneous Melanoma	TCGA-EB-A551	0	0	0	0	0
Skin Cuteneous Melanoma	TCGA-EB-A553	0	0	0	0	0
Skin Cuteneous Melanoma	TCGA-EB-A57M	0	0	0	0	0
Skin Cuteneous Melanoma	TCGA-EB-A5FP	0	0	0	0	0
Skin Cuteneous Melanoma	TCGA-EB-A5KH	1	1	1	1	1
Skin Cuteneous Melanoma	TCGA-EB-A5SE	0	0	0	0	0
Skin Cuteneous Melanoma	TCGA-EB-A5SF	0	0	0	0	0
Skin Cuteneous Melanoma	TCGA-EB-A5SG	1	1	1	1	1
Skin Cuteneous Melanoma	TCGA-EB-A5SH	1	1	1	1	1
Skin Cuteneous Melanoma	TCGA-EB-A5UL	1	1	1	1	1
Skin Cuteneous Melanoma	TCGA-EB-A5UM	0	0	0	0	0
Skin Cuteneous Melanoma	TCGA-EB-A5UN	1	1	1	1	1

Skin Cuteneous Melanoma	TCGA-EB-A5VU	0	0	0	0	0
Skin Cuteneous Melanoma	TCGA-EB-A5VV	0	0	0	0	0
Skin Cuteneous Melanoma	TCGA-EB-A6L9	0	0	1	0	0
Skin Cuteneous Melanoma	TCGA-EB-A6QY	0	0	0	0	0
Skin Cuteneous Melanoma	TCGA-EB-A6QZ	0	0	0	0	0
Skin Cuteneous Melanoma	TCGA-EB-A6R0	0	0	0	0	0
Skin Cuteneous Melanoma	TCGA-EB-A82B	0	0	0	0	0
Skin Cuteneous Melanoma	TCGA-EB-A82C	0	0	0	0	0
Skin Cuteneous Melanoma	TCGA-EB-A85I	0	0	0	0	0
Skin Cuteneous Melanoma	TCGA-EB-A85J	0	0	0	0	0
Skin Cuteneous Melanoma	TCGA-EB-A97M	0	0	0	0	0
Skin Cuteneous Melanoma	TCGA-EE-A17X	1	1	1	1	1
Skin Cuteneous Melanoma	TCGA-EE-A17Y	1	1	1	1	1
Skin Cuteneous Melanoma	TCGA-EE-A17Z	1	0	0	1	1
Skin Cuteneous Melanoma	TCGA-EE-A180	1	1	1	1	1
Skin Cuteneous Melanoma	TCGA-EE-A181	1	1	1	1	1
Skin Cuteneous Melanoma	TCGA-EE-A182	1	1	1	1	1
Skin Cuteneous Melanoma	TCGA-EE-A183	0	1	1	1	1
Skin Cuteneous Melanoma	TCGA-EE-A184	0	1	1	1	1
Skin Cuteneous Melanoma	TCGA-EE-A185	1	1	1	1	1
Skin Cuteneous Melanoma	TCGA-EE-A20B	1	1	1	1	1
Skin Cuteneous Melanoma	TCGA-EE-A20C	1	1	1	1	1
Skin Cuteneous Melanoma	TCGA-EE-A20F	1	1	0	1	1
Skin Cuteneous Melanoma	TCGA-EE-A20H	1	1	1	1	1
Skin Cuteneous Melanoma	TCGA-EE-A20I	1	1	1	1	1
Skin Cuteneous Melanoma	TCGA-EE-A29A	1	0	1	1	1
Skin Cuteneous Melanoma	TCGA-EE-A29B	1	1	1	1	0
Skin Cuteneous Melanoma	TCGA-EE-A29C	1	1	1	1	1
Skin Cuteneous Melanoma	TCGA-EE-A29D	1	1	1	1	1
Skin Cuteneous Melanoma	TCGA-EE-A29E	1	1	1	1	1
Skin Cuteneous Melanoma	TCGA-EE-A29G	1	1	1	1	1
Skin Cuteneous Melanoma	TCGA-EE-A29H	1	1	1	1	1
Skin Cuteneous Melanoma	TCGA-EE-A29L	1	1	1	1	1
Skin Cuteneous Melanoma	TCGA-EE-A29M	1	1	1	1	1
Skin Cuteneous Melanoma	TCGA-EE-A29N	1	1	1	1	1
Skin Cuteneous Melanoma	TCGA-EE-A29P	1	1	1	1	1
Skin Cuteneous Melanoma	TCGA-EE-A29Q	1	1	1	1	1
Skin Cuteneous Melanoma	TCGA-EE-A29R	0	1	1	1	1
Skin Cuteneous Melanoma	TCGA-EE-A29S	1	1	1	1	1
Skin Cuteneous Melanoma	TCGA-EE-A29T	1	1	1	1	1
Skin Cuteneous Melanoma	TCGA-EE-A29V	1	1	1	1	1
Skin Cuteneous Melanoma	TCGA-EE-A29W	1	1	1	1	1
Skin Cuteneous Melanoma	TCGA-EE-A29X	1	1	1	1	1
Skin Cuteneous Melanoma	TCGA-EE-A2A0	1	1	1	1	1
Skin Cuteneous Melanoma	TCGA-EE-A2A1	0	1	1	1	1
Skin Cuteneous Melanoma	TCGA-EE-A2A2	1	1	1	1	1
Skin Cuteneous Melanoma	TCGA-EE-A2A5	1	1	1	1	1
Skin Cuteneous Melanoma	TCGA-EE-A2A6	1	1	1	1	1
Skin Cuteneous Melanoma	TCGA-EE-A2GB	1	1	1	1	1
Skin Cuteneous Melanoma	TCGA-EE-A2GC	1	1	1	1	1
Skin Cuteneous Melanoma	TCGA-EE-A2GD	1	1	1	1	1
Skin Cuteneous Melanoma	TCGA-EE-A2GE	1	1	1	1	1
Skin Cuteneous Melanoma	TCGA-EE-A2GH	1	1	1	1	1
Skin Cuteneous Melanoma	TCGA-EE-A2GI	1	1	1	0	1
Skin Cuteneous Melanoma	TCGA-EE-A2GJ	1	1	0	1	1
Skin Cuteneous Melanoma	TCGA-EE-A2GK	0	0	0	0	0
Skin Cuteneous Melanoma	TCGA-EE-A2GL	1	1	1	1	1
Skin Cuteneous Melanoma	TCGA-EE-A2GM	1	1	1	1	1

Skin Cuteneous Melanoma	TCGA-EE-A2GN	1	0	1	1	1
Skin Cuteneous Melanoma	TCGA-EE-A2GO	1	1	1	1	1
Skin Cuteneous Melanoma	TCGA-EE-A2GP	1	1	1	1	1
Skin Cuteneous Melanoma	TCGA-EE-A2GR	1	1	1	1	1
Skin Cuteneous Melanoma	TCGA-EE-A2GS	1	1	1	1	1
Skin Cuteneous Melanoma	TCGA-EE-A2GT	1	1	1	1	1
Skin Cuteneous Melanoma	TCGA-EE-A2GU	1	1	1	1	1
Skin Cuteneous Melanoma	TCGA-EE-A2M5	1	1	1	1	1
Skin Cuteneous Melanoma	TCGA-EE-A2M6	1	1	1	1	1
Skin Cuteneous Melanoma	TCGA-EE-A2M7	1	0	1	1	1
Skin Cuteneous Melanoma	TCGA-EE-A2M8	1	1	0	1	1
Skin Cuteneous Melanoma	TCGA-EE-A2MC	1	1	1	1	1
Skin Cuteneous Melanoma	TCGA-EE-A2MD	1	1	1	1	1
Skin Cuteneous Melanoma	TCGA-EE-A2ME	0	0	0	1	1
Skin Cuteneous Melanoma	TCGA-EE-A2MF	1	1	1	1	1
Skin Cuteneous Melanoma	TCGA-EE-A2MG	1	1	1	1	1
Skin Cuteneous Melanoma	TCGA-EE-A2MH	1	1	1	1	1
Skin Cuteneous Melanoma	TCGA-EE-A2MI	1	1	0	1	1
Skin Cuteneous Melanoma	TCGA-EE-A2MJ	1	1	1	0	1
Skin Cuteneous Melanoma	TCGA-EE-A2MK	1	1	1	1	1
Skin Cuteneous Melanoma	TCGA-EE-A2ML	1	1	0	1	1
Skin Cuteneous Melanoma	TCGA-EE-A2MM	1	1	1	1	1
Skin Cuteneous Melanoma	TCGA-EE-A2MN	1	1	0	1	1
Skin Cuteneous Melanoma	TCGA-EE-A2MP	1	1	1	1	1
Skin Cuteneous Melanoma	TCGA-EE-A2MQ	1	1	1	1	1
Skin Cuteneous Melanoma	TCGA-EE-A2MR	1	1	1	0	1
Skin Cuteneous Melanoma	TCGA-EE-A2MS	1	1	1	1	1
Skin Cuteneous Melanoma	TCGA-EE-A2MT	1	1	1	0	1
Skin Cuteneous Melanoma	TCGA-EE-A2MU	1	1	1	1	1
Skin Cuteneous Melanoma	TCGA-EE-A3AA	1	1	1	1	1
Skin Cuteneous Melanoma	TCGA-EE-A3AB	1	1	1	1	1
Skin Cuteneous Melanoma	TCGA-EE-A3AC	1	1	1	1	1
Skin Cuteneous Melanoma	TCGA-EE-A3AD	1	1	1	1	1
Skin Cuteneous Melanoma	TCGA-EE-A3AE	1	1	1	1	1
Skin Cuteneous Melanoma	TCGA-EE-A3AF	1	1	1	1	1
Skin Cuteneous Melanoma	TCGA-EE-A3AG	1	1	1	1	1
Skin Cuteneous Melanoma	TCGA-EE-A3AH	1	1	1	1	1
Skin Cuteneous Melanoma	TCGA-EE-A3J3	1	1	1	1	1
Skin Cuteneous Melanoma	TCGA-EE-A3J4	1	1	1	1	1
Skin Cuteneous Melanoma	TCGA-EE-A3J5	1	1	1	1	1
Skin Cuteneous Melanoma	TCGA-EE-A3J7	1	1	1	1	1
Skin Cuteneous Melanoma	TCGA-EE-A3J8	1	1	1	1	1
Skin Cuteneous Melanoma	TCGA-EE-A3JA	1	1	1	1	1
Skin Cuteneous Melanoma	TCGA-EE-A3JB	1	1	1	1	1
Skin Cuteneous Melanoma	TCGA-EE-A3JD	1	1	1	1	1
Skin Cuteneous Melanoma	TCGA-EE-A3JE	1	1	1	1	1
Skin Cuteneous Melanoma	TCGA-EE-A3JH	1	1	1	1	1
Skin Cuteneous Melanoma	TCGA-EE-A3JI	1	1	1	1	1
Skin Cuteneous Melanoma	TCGA-ER-A193	1	1	1	1	1
Skin Cuteneous Melanoma	TCGA-ER-A194	0	0	0	0	0
Skin Cuteneous Melanoma	TCGA-ER-A195	1	1	1	1	1
Skin Cuteneous Melanoma	TCGA-ER-A196	0	0	0	0	0
Skin Cuteneous Melanoma	TCGA-ER-A197	1	1	1	1	1
Skin Cuteneous Melanoma	TCGA-ER-A198	1	0	1	1	1
Skin Cuteneous Melanoma	TCGA-ER-A199	1	1	1	1	1
Skin Cuteneous Melanoma	TCGA-ER-A19A	0	0	1	0	0
Skin Cuteneous Melanoma	TCGA-ER-A19B	1	1	1	1	1
Skin Cuteneous Melanoma	TCGA-ER-A19C	1	1	1	1	1

Skin Cuteneous Melanoma	TCGA-ER-A19D	1	1	0	1	1
Skin Cuteneous Melanoma	TCGA-ER-A19E	1	1	1	1	1
Skin Cuteneous Melanoma	TCGA-ER-A19F	1	1	1	1	1
Skin Cuteneous Melanoma	TCGA-ER-A19G	1	1	1	1	1
Skin Cuteneous Melanoma	TCGA-ER-A19H	1	1	1	1	1
Skin Cuteneous Melanoma	TCGA-ER-A19J	1	1	1	1	1
Skin Cuteneous Melanoma	TCGA-ER-A19K	0	0	0	0	0
Skin Cuteneous Melanoma	TCGA-ER-A19L	1	1	1	1	1
Skin Cuteneous Melanoma	TCGA-ER-A19M	0	1	1	1	1
Skin Cuteneous Melanoma	TCGA-ER-A19N	1	1	1	1	1
Skin Cuteneous Melanoma	TCGA-ER-A19O	0	1	1	1	1
Skin Cuteneous Melanoma	TCGA-ER-A19P	1	1	1	1	1
Skin Cuteneous Melanoma	TCGA-ER-A19Q	1	1	1	1	1
Skin Cuteneous Melanoma	TCGA-ER-A19S	1	1	1	1	1
Skin Cuteneous Melanoma	TCGA-ER-A19T	1	1	1	1	1
Skin Cuteneous Melanoma	TCGA-ER-A19T	0	0	0	0	0
Skin Cuteneous Melanoma	TCGA-ER-A19W	1	1	1	1	1
Skin Cuteneous Melanoma	TCGA-ER-A1A1	1	0	0	0	0
Skin Cuteneous Melanoma	TCGA-ER-A2NB	0	0	0	0	0
Skin Cuteneous Melanoma	TCGA-ER-A2NC	1	0	0	1	1
Skin Cuteneous Melanoma	TCGA-ER-A2ND	1	1	1	1	1
Skin Cuteneous Melanoma	TCGA-ER-A2NE	1	1	1	1	1
Skin Cuteneous Melanoma	TCGA-ER-A2NF	0	1	1	0	1
Skin Cuteneous Melanoma	TCGA-ER-A2NF	0	0	0	0	0
Skin Cuteneous Melanoma	TCGA-ER-A2NG	1	1	1	1	1
Skin Cuteneous Melanoma	TCGA-ER-A2NH	1	1	1	1	1
Skin Cuteneous Melanoma	TCGA-ER-A3ES	1	1	0	1	1
Skin Cuteneous Melanoma	TCGA-ER-A3ET	1	1	1	1	1
Skin Cuteneous Melanoma	TCGA-ER-A3EV	1	1	1	1	1
Skin Cuteneous Melanoma	TCGA-ER-A3PL	1	1	1	1	1
Skin Cuteneous Melanoma	TCGA-ER-A42H	0	0	0	0	0
Skin Cuteneous Melanoma	TCGA-ER-A42K	1	1	1	1	1
Skin Cuteneous Melanoma	TCGA-ER-A42L	1	1	1	1	1
Skin Cuteneous Melanoma	TCGA-FR-A2OS	0	0	0	0	0
Skin Cuteneous Melanoma	TCGA-FR-A3R1	0	0	0	0	0
Skin Cuteneous Melanoma	TCGA-FR-A3YN	1	1	1	1	1
Skin Cuteneous Melanoma	TCGA-FR-A3YO	1	1	1	1	1
Skin Cuteneous Melanoma	TCGA-FR-A44A	1	1	1	1	1
Skin Cuteneous Melanoma	TCGA-FR-A69P	1	1	1	1	1
Skin Cuteneous Melanoma	TCGA-FR-A726	0	0	0	0	0
Skin Cuteneous Melanoma	TCGA-FR-A728	0	0	0	0	0
Skin Cuteneous Melanoma	TCGA-FR-A729	1	1	1	1	1
Skin Cuteneous Melanoma	TCGA-FR-A7U8	1	1	1	1	1
Skin Cuteneous Melanoma	TCGA-FR-A7U9	1	1	1	1	1
Skin Cuteneous Melanoma	TCGA-FR-A7UA	0	0	0	1	1
Skin Cuteneous Melanoma	TCGA-FR-A8YC	1	1	1	0	1
Skin Cuteneous Melanoma	TCGA-FR-A8YD	0	0	1	0	1
Skin Cuteneous Melanoma	TCGA-FR-A8YE	1	1	1	0	1
Skin Cuteneous Melanoma	TCGA-FS-A1YW	1	1	1	1	1
Skin Cuteneous Melanoma	TCGA-FS-A1YX	1	1	1	1	1
Skin Cuteneous Melanoma	TCGA-FS-A1YY	1	1	1	1	1
Skin Cuteneous Melanoma	TCGA-FS-A1Z0	1	1	1	1	1
Skin Cuteneous Melanoma	TCGA-FS-A1Z3	1	1	0	1	1
Skin Cuteneous Melanoma	TCGA-FS-A1Z4	1	1	1	1	1
Skin Cuteneous Melanoma	TCGA-FS-A1Z7	1	1	0	1	1
Skin Cuteneous Melanoma	TCGA-FS-A1ZA	1	1	1	1	1
Skin Cuteneous Melanoma	TCGA-FS-A1ZB	1	1	1	1	1
Skin Cuteneous Melanoma	TCGA-FS-A1ZC	1	1	1	1	1

Skin Cuteneous Melanoma	TCGA-FS-A1ZD	1	1	1	1	1
Skin Cuteneous Melanoma	TCGA-FS-A1ZE	1	1	1	1	1
Skin Cuteneous Melanoma	TCGA-FS-A1ZF	1	1	1	1	1
Skin Cuteneous Melanoma	TCGA-FS-A1ZG	0	1	1	1	1
Skin Cuteneous Melanoma	TCGA-FS-A1ZH	1	1	1	1	1
Skin Cuteneous Melanoma	TCGA-FS-A1ZJ	1	1	1	1	1
Skin Cuteneous Melanoma	TCGA-FS-A1ZK	1	1	0	1	1
Skin Cuteneous Melanoma	TCGA-FS-A1ZM	1	1	1	1	1
Skin Cuteneous Melanoma	TCGA-FS-A1ZN	0	0	0	0	0
Skin Cuteneous Melanoma	TCGA-FS-A1ZP	0	0	1	1	0
Skin Cuteneous Melanoma	TCGA-FS-A1ZQ	1	1	1	1	1
Skin Cuteneous Melanoma	TCGA-FS-A1ZR	1	1	1	1	1
Skin Cuteneous Melanoma	TCGA-FS-A1ZS	1	1	1	1	1
Skin Cuteneous Melanoma	TCGA-FS-A1ZT	1	1	1	1	1
Skin Cuteneous Melanoma	TCGA-FS-A1ZU	1	1	1	1	1
Skin Cuteneous Melanoma	TCGA-FS-A1ZW	1	1	1	1	1
Skin Cuteneous Melanoma	TCGA-FS-A1ZY	1	1	1	1	1
Skin Cuteneous Melanoma	TCGA-FS-A1ZZ	1	1	1	1	1
Skin Cuteneous Melanoma	TCGA-FS-A4F0	1	1	0	1	1
Skin Cuteneous Melanoma	TCGA-FS-A4F2	1	1	1	0	1
Skin Cuteneous Melanoma	TCGA-FS-A4F4	1	1	1	1	1
Skin Cuteneous Melanoma	TCGA-FS-A4F5	1	1	1	1	1
Skin Cuteneous Melanoma	TCGA-FS-A4F8	1	1	1	1	1
Skin Cuteneous Melanoma	TCGA-FS-A4F9	1	1	1	1	1
Skin Cuteneous Melanoma	TCGA-FS-A4FB	1	1	1	1	1
Skin Cuteneous Melanoma	TCGA-FS-A4FC	1	1	0	1	1
Skin Cuteneous Melanoma	TCGA-FS-A4FD	1	1	1	1	1
Skin Cuteneous Melanoma	TCGA-FW-A3I3	1	1	1	1	1
Skin Cuteneous Melanoma	TCGA-FW-A3R5	1	1	1	1	1
Skin Cuteneous Melanoma	TCGA-FW-A3TU	1	1	1	1	1
Skin Cuteneous Melanoma	TCGA-FW-A3TV	1	1	1	1	1
Skin Cuteneous Melanoma	TCGA-FW-A5DX	0	0	0	0	0
Skin Cuteneous Melanoma	TCGA-FW-A5DY	0	0	0	1	1
Skin Cuteneous Melanoma	TCGA-GF-A2C7	0	0	0	0	0
Skin Cuteneous Melanoma	TCGA-GF-A3OT	1	0	0	1	1
Skin Cuteneous Melanoma	TCGA-GF-A4EO	1	1	1	1	1
Skin Cuteneous Melanoma	TCGA-GF-A6C8	1	1	1	1	1
Skin Cuteneous Melanoma	TCGA-GF-A6C9	1	1	1	1	1
Skin Cuteneous Melanoma	TCGA-GF-A769	0	0	0	0	0
Skin Cuteneous Melanoma	TCGA-GN-A262	1	1	1	1	1
Skin Cuteneous Melanoma	TCGA-GN-A263	0	0	0	0	0
Skin Cuteneous Melanoma	TCGA-GN-A264	1	1	1	1	1
Skin Cuteneous Melanoma	TCGA-GN-A265	1	1	1	1	1
Skin Cuteneous Melanoma	TCGA-GN-A266	1	1	1	1	1
Skin Cuteneous Melanoma	TCGA-GN-A267	1	1	1	1	1
Skin Cuteneous Melanoma	TCGA-GN-A268	1	1	1	1	1
Skin Cuteneous Melanoma	TCGA-GN-A26A	1	1	1	1	1
Skin Cuteneous Melanoma	TCGA-GN-A26C	0	0	0	0	0
Skin Cuteneous Melanoma	TCGA-GN-A26D	1	1	1	1	1
Skin Cuteneous Melanoma	TCGA-GN-A4U3	1	1	1	1	1
Skin Cuteneous Melanoma	TCGA-GN-A4U4	0	1	1	1	1
Skin Cuteneous Melanoma	TCGA-GN-A4U5	0	0	0	0	0
Skin Cuteneous Melanoma	TCGA-GN-A4U7	1	1	1	1	1
Skin Cuteneous Melanoma	TCGA-GN-A4U8	1	0	1	1	1
Skin Cuteneous Melanoma	TCGA-GN-A4U9	1	1	1	1	1
Skin Cuteneous Melanoma	TCGA-GN-A8LK	1	1	1	1	1
Skin Cuteneous Melanoma	TCGA-GN-A8LL	1	1	1	1	1
Skin Cuteneous Melanoma	TCGA-GN-A8LN	0	0	0	0	0

Skin Cuteneous Melanoma	TCGA-GN-A9SD	1	1	0	0	1
Skin Cuteneous Melanoma	TCGA-HR-A2OG	1	1	1	0	1
Skin Cuteneous Melanoma	TCGA-HR-A2OH	1	1	1	0	1
Skin Cuteneous Melanoma	TCGA-HR-A5NC	0	0	0	0	0
Skin Cuteneous Melanoma	TCGA-IH-A3EA	0	0	0	0	0
Skin Cuteneous Melanoma	TCGA-LH-A9QB	1	0	1	0	1
Skin Cuteneous Melanoma	TCGA-OD-A75X	1	1	1	1	1
Skin Cuteneous Melanoma	TCGA-QB-A6FS	1	1	1	1	1
Skin Cuteneous Melanoma	TCGA-QB-AA9O	1	1	1	1	1
Skin Cuteneous Melanoma	TCGA-RP-A690	0	1	0	1	0
Skin Cuteneous Melanoma	TCGA-RP-A693	1	1	1	1	1
Skin Cuteneous Melanoma	TCGA-RP-A694	1	1	1	0	1
Skin Cuteneous Melanoma	TCGA-RP-A695	1	1	1	1	1
Skin Cuteneous Melanoma	TCGA-RP-A6K9	1	1	1	1	1
Skin Cuteneous Melanoma	TCGA-W3-A824	1	1	0	0	0
Skin Cuteneous Melanoma	TCGA-W3-A825	1	1	1	1	1
Skin Cuteneous Melanoma	TCGA-W3-A828	1	1	0	1	1
Skin Cuteneous Melanoma	TCGA-W3-AA1O	0	1	1	0	1
Skin Cuteneous Melanoma	TCGA-W3-AA1Q	0	0	0	0	0
Skin Cuteneous Melanoma	TCGA-W3-AA1R	1	1	1	1	1
Skin Cuteneous Melanoma	TCGA-W3-AA1V	1	1	1	1	1
Skin Cuteneous Melanoma	TCGA-W3-AA1W	0	0	0	0	0
Skin Cuteneous Melanoma	TCGA-W3-AA21	1	1	1	1	1
Skin Cuteneous Melanoma	TCGA-WE-A8JZ	1	1	1	1	1
Skin Cuteneous Melanoma	TCGA-WE-A8K1	1	0	1	0	1
Skin Cuteneous Melanoma	TCGA-WE-A8K4	0	0	0	0	0
Skin Cuteneous Melanoma	TCGA-WE-A8K5	1	1	0	1	1
Skin Cuteneous Melanoma	TCGA-WE-A8K6	1	1	1	1	1
Skin Cuteneous Melanoma	TCGA-WE-A8ZM	1	1	1	1	1
Skin Cuteneous Melanoma	TCGA-WE-A8ZN	0	0	0	0	1
Skin Cuteneous Melanoma	TCGA-WE-A8ZO	1	1	0	0	1
Skin Cuteneous Melanoma	TCGA-WE-A8ZQ	1	1	1	1	1
Skin Cuteneous Melanoma	TCGA-WE-A8ZR	0	0	0	0	0
Skin Cuteneous Melanoma	TCGA-WE-A8ZT	1	0	0	0	1
Skin Cuteneous Melanoma	TCGA-WE-A8ZX	1	1	1	1	1
Skin Cuteneous Melanoma	TCGA-WE-A8ZY	1	1	1	1	1
Skin Cuteneous Melanoma	TCGA-WE-AA9Y	1	1	1	1	1
Skin Cuteneous Melanoma	TCGA-WE-AAA0	1	1	1	1	1
Skin Cuteneous Melanoma	TCGA-WE-AAA3	1	1	1	1	1
Skin Cuteneous Melanoma	TCGA-WE-AAA4	1	1	1	1	1
Skin Cuteneous Melanoma	TCGA-XV-A9VZ	0	0	0	0	0
Skin Cuteneous Melanoma	TCGA-XV-A9W2	0	0	0	0	0
Skin Cuteneous Melanoma	TCGA-XV-A9W5	0	0	0	0	0
Skin Cuteneous Melanoma	TCGA-XV-AAZV	0	0	0	0	0
Skin Cuteneous Melanoma	TCGA-XV-AAZW	0	0	0	0	0
Skin Cuteneous Melanoma	TCGA-XV-AAZY	0	0	0	0	0
Skin Cuteneous Melanoma	TCGA-XV-AB01	0	0	0	0	0
Skin Cuteneous Melanoma	TCGA-YD-A89C	0	0	0	0	0
Skin Cuteneous Melanoma	TCGA-YD-A9TA	1	1	1	0	1
Skin Cuteneous Melanoma	TCGA-YD-A9TB	1	1	1	1	1
Skin Cuteneous Melanoma	TCGA-YG-AA3N	0	0	0	0	0
Skin Cuteneous Melanoma	TCGA-YG-AA3O	1	1	1	1	1
Skin Cuteneous Melanoma	TCGA-YG-AA3P	1	1	1	1	1
Skin Cuteneous Melanoma	TCGA-Z2-A8RT	1	1	0	1	1
Skin Cuteneous Melanoma	TCGA-Z2-AA3S	1	1	1	1	1
Skin Cuteneous Melanoma	TCGA-Z2-AA3V	1	1	1	0	1
Stomach adenocarcinoma	TCGA-3M-AB46	1	1	1	1	1
Stomach adenocarcinoma	TCGA-3M-AB47	0	0	0	0	0

Stomach adenocarcinoma	TCGA-B7-5816	1	1	0	1	0
Stomach adenocarcinoma	TCGA-B7-5818	1	0	1	0	1
Stomach adenocarcinoma	TCGA-B7-A5TI	1	1	1	0	1
Stomach adenocarcinoma	TCGA-B7-A5TJ	1	1	1	1	1
Stomach adenocarcinoma	TCGA-B7-A5TK	1	0	0	0	0
Stomach adenocarcinoma	TCGA-B7-A5TN	1	1	1	1	1
Stomach adenocarcinoma	TCGA-BR-4187	0	0	0	0	0
Stomach adenocarcinoma	TCGA-BR-4191	1	1	1	1	1
Stomach adenocarcinoma	TCGA-BR-4201	1	1	0	1	1
Stomach adenocarcinoma	TCGA-BR-4253	1	0	0	0	0
Stomach adenocarcinoma	TCGA-BR-4255	1	1	1	1	1
Stomach adenocarcinoma	TCGA-BR-4256	1	0	0	1	1
Stomach adenocarcinoma	TCGA-BR-4257	1	1	1	1	1
Stomach adenocarcinoma	TCGA-BR-4267	1	1	1	1	1
Stomach adenocarcinoma	TCGA-BR-4279	1	1	0	1	1
Stomach adenocarcinoma	TCGA-BR-4280	0	0	0	0	1
Stomach adenocarcinoma	TCGA-BR-4292	1	1	1	0	1
Stomach adenocarcinoma	TCGA-BR-4294	1	1	1	1	1
Stomach adenocarcinoma	TCGA-BR-4357	1	1	1	1	1
Stomach adenocarcinoma	TCGA-BR-4361	1	0	1	0	1
Stomach adenocarcinoma	TCGA-BR-4362	1	1	0	1	1
Stomach adenocarcinoma	TCGA-BR-4363	0	0	1	1	1
Stomach adenocarcinoma	TCGA-BR-4366	1	1	1	1	1
Stomach adenocarcinoma	TCGA-BR-4367	1	1	1	1	1
Stomach adenocarcinoma	TCGA-BR-4368	0	1	0	0	1
Stomach adenocarcinoma	TCGA-BR-4369	1	1	1	1	1
Stomach adenocarcinoma	TCGA-BR-4370	0	1	1	0	1
Stomach adenocarcinoma	TCGA-BR-4371	1	1	0	1	0
Stomach adenocarcinoma	TCGA-BR-6452	1	1	1	1	1
Stomach adenocarcinoma	TCGA-BR-6453	0	0	0	0	0
Stomach adenocarcinoma	TCGA-BR-6454	1	1	1	1	1
Stomach adenocarcinoma	TCGA-BR-6455	1	0	0	1	0
Stomach adenocarcinoma	TCGA-BR-6456	1	1	1	1	1
Stomach adenocarcinoma	TCGA-BR-6457	1	1	1	0	1
Stomach adenocarcinoma	TCGA-BR-6458	1	1	1	1	1
Stomach adenocarcinoma	TCGA-BR-6563	0	0	0	0	1
Stomach adenocarcinoma	TCGA-BR-6564	1	1	0	1	1
Stomach adenocarcinoma	TCGA-BR-6565	1	1	1	1	1
Stomach adenocarcinoma	TCGA-BR-6566	0	0	0	1	1
Stomach adenocarcinoma	TCGA-BR-6705	1	1	0	1	1
Stomach adenocarcinoma	TCGA-BR-6706	1	0	0	0	0
Stomach adenocarcinoma	TCGA-BR-6707	1	0	0	0	0
Stomach adenocarcinoma	TCGA-BR-6709	1	1	1	1	1
Stomach adenocarcinoma	TCGA-BR-6801	0	0	0	0	1
Stomach adenocarcinoma	TCGA-BR-6802	1	0	0	0	1
Stomach adenocarcinoma	TCGA-BR-6803	1	1	0	1	1
Stomach adenocarcinoma	TCGA-BR-6852	1	1	0	0	1
Stomach adenocarcinoma	TCGA-BR-7196	1	0	0	0	0
Stomach adenocarcinoma	TCGA-BR-7197	0	1	1	1	1
Stomach adenocarcinoma	TCGA-BR-7703	1	0	0	1	1
Stomach adenocarcinoma	TCGA-BR-7704	1	1	1	1	1
Stomach adenocarcinoma	TCGA-BR-7707	0	0	0	1	1
Stomach adenocarcinoma	TCGA-BR-7715	1	1	1	1	1
Stomach adenocarcinoma	TCGA-BR-7716	1	1	1	1	1
Stomach adenocarcinoma	TCGA-BR-7717	1	1	0	0	1
Stomach adenocarcinoma	TCGA-BR-7722	1	0	1	0	1
Stomach adenocarcinoma	TCGA-BR-7723	1	1	1	1	1
Stomach adenocarcinoma	TCGA-BR-7851	1	1	0	1	1

Stomach adenocarcinoma	TCGA-BR-7901	1	1	1	1	1
Stomach adenocarcinoma	TCGA-BR-7957	1	0	0	1	1
Stomach adenocarcinoma	TCGA-BR-7958	1	1	1	1	1
Stomach adenocarcinoma	TCGA-BR-7959	1	1	1	1	1
Stomach adenocarcinoma	TCGA-BR-8058	1	1	0	1	1
Stomach adenocarcinoma	TCGA-BR-8059	1	0	1	1	1
Stomach adenocarcinoma	TCGA-BR-8060	1	1	1	1	1
Stomach adenocarcinoma	TCGA-BR-8077	1	1	1	1	1
Stomach adenocarcinoma	TCGA-BR-8078	1	1	1	1	1
Stomach adenocarcinoma	TCGA-BR-8080	1	1	1	1	1
Stomach adenocarcinoma	TCGA-BR-8081	1	1	0	1	1
Stomach adenocarcinoma	TCGA-BR-8284	1	1	0	0	0
Stomach adenocarcinoma	TCGA-BR-8286	1	1	1	1	1
Stomach adenocarcinoma	TCGA-BR-8289	1	1	1	1	1
Stomach adenocarcinoma	TCGA-BR-8291	1	1	1	1	1
Stomach adenocarcinoma	TCGA-BR-8295	1	1	1	1	1
Stomach adenocarcinoma	TCGA-BR-8296	1	1	1	0	1
Stomach adenocarcinoma	TCGA-BR-8297	1	0	1	1	1
Stomach adenocarcinoma	TCGA-BR-8361	1	1	0	0	1
Stomach adenocarcinoma	TCGA-BR-8362	1	1	1	0	1
Stomach adenocarcinoma	TCGA-BR-8363	1	1	0	1	1
Stomach adenocarcinoma	TCGA-BR-8364	1	1	0	0	1
Stomach adenocarcinoma	TCGA-BR-8365	1	1	0	1	1
Stomach adenocarcinoma	TCGA-BR-8366	1	0	0	1	0
Stomach adenocarcinoma	TCGA-BR-8367	0	0	0	0	0
Stomach adenocarcinoma	TCGA-BR-8368	1	1	0	1	1
Stomach adenocarcinoma	TCGA-BR-8369	1	1	1	1	1
Stomach adenocarcinoma	TCGA-BR-8371	0	0	0	0	0
Stomach adenocarcinoma	TCGA-BR-8372	1	0	0	0	1
Stomach adenocarcinoma	TCGA-BR-8373	1	1	1	1	1
Stomach adenocarcinoma	TCGA-BR-8380	0	0	0	0	0
Stomach adenocarcinoma	TCGA-BR-8381	1	0	0	0	0
Stomach adenocarcinoma	TCGA-BR-8382	1	1	0	0	0
Stomach adenocarcinoma	TCGA-BR-8384	1	1	0	1	1
Stomach adenocarcinoma	TCGA-BR-8483	1	1	1	1	1
Stomach adenocarcinoma	TCGA-BR-8484	1	1	1	1	1
Stomach adenocarcinoma	TCGA-BR-8485	1	1	1	1	1
Stomach adenocarcinoma	TCGA-BR-8486	1	1	1	1	1
Stomach adenocarcinoma	TCGA-BR-8487	1	1	0	0	1
Stomach adenocarcinoma	TCGA-BR-8588	1	0	0	0	0
Stomach adenocarcinoma	TCGA-BR-8589	1	1	0	0	1
Stomach adenocarcinoma	TCGA-BR-8590	1	0	0	1	1
Stomach adenocarcinoma	TCGA-BR-8591	1	1	0	1	1
Stomach adenocarcinoma	TCGA-BR-8592	0	0	0	0	0
Stomach adenocarcinoma	TCGA-BR-8676	1	0	0	0	0
Stomach adenocarcinoma	TCGA-BR-8677	1	1	0	1	1
Stomach adenocarcinoma	TCGA-BR-8678	1	1	1	1	1
Stomach adenocarcinoma	TCGA-BR-8679	1	1	1	1	1
Stomach adenocarcinoma	TCGA-BR-8680	1	1	1	0	1
Stomach adenocarcinoma	TCGA-BR-8682	0	1	1	1	1
Stomach adenocarcinoma	TCGA-BR-8683	1	1	1	1	1
Stomach adenocarcinoma	TCGA-BR-8686	1	1	0	1	1
Stomach adenocarcinoma	TCGA-BR-8687	1	1	1	1	1
Stomach adenocarcinoma	TCGA-BR-8690	1	1	1	1	1
Stomach adenocarcinoma	TCGA-BR-A44T	0	0	0	0	0
Stomach adenocarcinoma	TCGA-BR-A44U	1	1	1	1	1
Stomach adenocarcinoma	TCGA-BR-A452	1	1	1	1	1
Stomach adenocarcinoma	TCGA-BR-A453	0	0	0	0	0

Stomach adenocarcinoma	TCGA-BR-A4CR	1	1	1	1	1
Stomach adenocarcinoma	TCGA-BR-A4CS	1	1	1	1	1
Stomach adenocarcinoma	TCGA-BR-A4IU	0	0	0	0	1
Stomach adenocarcinoma	TCGA-BR-A4IV	0	0	0	0	0
Stomach adenocarcinoma	TCGA-BR-A4IY	1	1	1	1	1
Stomach adenocarcinoma	TCGA-BR-A4IZ	0	0	0	0	0
Stomach adenocarcinoma	TCGA-BR-A4J1	1	1	1	1	1
Stomach adenocarcinoma	TCGA-BR-A4J2	0	1	1	0	0
Stomach adenocarcinoma	TCGA-BR-A4J4	0	1	0	1	0
Stomach adenocarcinoma	TCGA-BR-A4J5	0	0	0	0	0
Stomach adenocarcinoma	TCGA-BR-A4J6	1	1	1	1	1
Stomach adenocarcinoma	TCGA-BR-A4J7	1	0	0	1	1
Stomach adenocarcinoma	TCGA-BR-A4J8	1	1	1	1	1
Stomach adenocarcinoma	TCGA-BR-A4J9	1	0	0	0	0
Stomach adenocarcinoma	TCGA-BR-A4PD	1	1	1	1	1
Stomach adenocarcinoma	TCGA-BR-A4PE	1	1	1	1	1
Stomach adenocarcinoma	TCGA-BR-A4PF	1	1	1	1	1
Stomach adenocarcinoma	TCGA-BR-A4QI	1	1	1	1	1
Stomach adenocarcinoma	TCGA-BR-A4QL	1	1	1	0	1
Stomach adenocarcinoma	TCGA-BR-A4QM	0	0	0	1	1
Stomach adenocarcinoma	TCGA-CD-5798	0	0	0	0	1
Stomach adenocarcinoma	TCGA-CD-5799	1	1	1	1	1
Stomach adenocarcinoma	TCGA-CD-5800	1	1	1	1	1
Stomach adenocarcinoma	TCGA-CD-5801	1	1	1	1	1
Stomach adenocarcinoma	TCGA-CD-5803	1	1	1	1	1
Stomach adenocarcinoma	TCGA-CD-5804	1	1	1	1	1
Stomach adenocarcinoma	TCGA-CD-5813	0	0	0	0	0
Stomach adenocarcinoma	TCGA-CD-8524	1	1	1	1	1
Stomach adenocarcinoma	TCGA-CD-8525	1	1	1	1	1
Stomach adenocarcinoma	TCGA-CD-8526	1	1	1	1	1
Stomach adenocarcinoma	TCGA-CD-8527	1	1	1	1	1
Stomach adenocarcinoma	TCGA-CD-8528	1	1	1	1	1
Stomach adenocarcinoma	TCGA-CD-8529	1	1	1	1	1
Stomach adenocarcinoma	TCGA-CD-8530	1	1	1	1	1
Stomach adenocarcinoma	TCGA-CD-8531	1	0	0	1	1
Stomach adenocarcinoma	TCGA-CD-8532	1	1	1	1	1
Stomach adenocarcinoma	TCGA-CD-8533	1	1	1	1	1
Stomach adenocarcinoma	TCGA-CD-8534	1	1	1	1	1
Stomach adenocarcinoma	TCGA-CD-8535	1	1	1	1	1
Stomach adenocarcinoma	TCGA-CD-8536	1	0	0	1	0
Stomach adenocarcinoma	TCGA-CD-A486	1	1	1	1	1
Stomach adenocarcinoma	TCGA-CD-A487	1	1	1	1	1
Stomach adenocarcinoma	TCGA-CD-A489	1	1	1	1	1
Stomach adenocarcinoma	TCGA-CD-A48A	1	1	1	1	1
Stomach adenocarcinoma	TCGA-CD-A48C	1	1	1	1	1
Stomach adenocarcinoma	TCGA-CD-A4MG	0	1	0	0	1
Stomach adenocarcinoma	TCGA-CD-A4MH	1	1	1	1	1
Stomach adenocarcinoma	TCGA-CD-A4MI	1	0	0	0	1
Stomach adenocarcinoma	TCGA-CD-A4MJ	0	1	0	0	1
Stomach adenocarcinoma	TCGA-CG-4301	1	1	1	1	1
Stomach adenocarcinoma	TCGA-CG-4304	1	0	1	0	1
Stomach adenocarcinoma	TCGA-CG-4305	1	1	0	0	1
Stomach adenocarcinoma	TCGA-CG-4306	0	1	0	1	1
Stomach adenocarcinoma	TCGA-CG-4436	1	1	1	1	1
Stomach adenocarcinoma	TCGA-CG-4437	1	1	0	1	1
Stomach adenocarcinoma	TCGA-CG-4438	1	1	1	1	1
Stomach adenocarcinoma	TCGA-CG-4440	1	1	1	1	1
Stomach adenocarcinoma	TCGA-CG-4441	1	1	1	0	1

Stomach adenocarcinoma	TCGA-CG-4442	1	1	1	0	1
Stomach adenocarcinoma	TCGA-CG-4443	1	1	1	1	1
Stomach adenocarcinoma	TCGA-CG-4444	1	1	1	1	1
Stomach adenocarcinoma	TCGA-CG-4449	1	1	1	1	1
Stomach adenocarcinoma	TCGA-CG-4460	1	1	1	1	1
Stomach adenocarcinoma	TCGA-CG-4462	0	0	0	0	1
Stomach adenocarcinoma	TCGA-CG-4465	0	1	1	1	0
Stomach adenocarcinoma	TCGA-CG-4466	1	1	1	1	1
Stomach adenocarcinoma	TCGA-CG-4469	1	1	1	0	1
Stomach adenocarcinoma	TCGA-CG-4472	1	1	0	1	1
Stomach adenocarcinoma	TCGA-CG-4474	1	1	0	0	1
Stomach adenocarcinoma	TCGA-CG-4475	1	1	1	1	1
Stomach adenocarcinoma	TCGA-CG-4476	1	1	1	1	1
Stomach adenocarcinoma	TCGA-CG-4477	1	1	1	1	1
Stomach adenocarcinoma	TCGA-CG-5716	0	0	0	0	0
Stomach adenocarcinoma	TCGA-CG-5717	1	0	0	1	1
Stomach adenocarcinoma	TCGA-CG-5718	1	1	1	1	1
Stomach adenocarcinoma	TCGA-CG-5719	1	1	1	1	1
Stomach adenocarcinoma	TCGA-CG-5720	0	0	0	0	1
Stomach adenocarcinoma	TCGA-CG-5721	1	1	0	1	1
Stomach adenocarcinoma	TCGA-CG-5722	1	0	0	0	0
Stomach adenocarcinoma	TCGA-CG-5723	0	1	0	1	1
Stomach adenocarcinoma	TCGA-CG-5724	1	1	1	1	1
Stomach adenocarcinoma	TCGA-CG-5725	1	1	1	1	1
Stomach adenocarcinoma	TCGA-CG-5726	0	1	1	1	1
Stomach adenocarcinoma	TCGA-CG-5732	1	1	1	1	1
Stomach adenocarcinoma	TCGA-CG-5734	0	0	0	0	0
Stomach adenocarcinoma	TCGA-D7-5577	1	0	0	1	1
Stomach adenocarcinoma	TCGA-D7-5578	1	1	1	1	1
Stomach adenocarcinoma	TCGA-D7-6518	1	1	1	1	1
Stomach adenocarcinoma	TCGA-D7-6519	1	1	1	1	1
Stomach adenocarcinoma	TCGA-D7-6520	1	1	1	0	1
Stomach adenocarcinoma	TCGA-D7-6521	1	1	1	1	1
Stomach adenocarcinoma	TCGA-D7-6522	0	0	0	0	0
Stomach adenocarcinoma	TCGA-D7-6524	0	0	0	0	0
Stomach adenocarcinoma	TCGA-D7-6525	1	1	1	1	1
Stomach adenocarcinoma	TCGA-D7-6526	1	1	1	1	1
Stomach adenocarcinoma	TCGA-D7-6527	1	1	1	1	1
Stomach adenocarcinoma	TCGA-D7-6528	1	1	1	1	1
Stomach adenocarcinoma	TCGA-D7-6815	1	1	1	1	1
Stomach adenocarcinoma	TCGA-D7-6817	1	1	1	1	1
Stomach adenocarcinoma	TCGA-D7-6818	1	1	1	1	1
Stomach adenocarcinoma	TCGA-D7-6820	1	1	1	1	1
Stomach adenocarcinoma	TCGA-D7-6822	1	1	1	1	1
Stomach adenocarcinoma	TCGA-D7-8570	1	1	1	1	1
Stomach adenocarcinoma	TCGA-D7-8572	1	1	1	0	0
Stomach adenocarcinoma	TCGA-D7-8573	1	1	1	1	1
Stomach adenocarcinoma	TCGA-D7-8574	0	0	0	0	0
Stomach adenocarcinoma	TCGA-D7-8575	1	1	0	1	1
Stomach adenocarcinoma	TCGA-D7-8576	1	1	1	1	1
Stomach adenocarcinoma	TCGA-D7-8578	1	1	1	1	1
Stomach adenocarcinoma	TCGA-D7-8579	0	1	0	0	1
Stomach adenocarcinoma	TCGA-D7-A4YT	1	1	1	1	1
Stomach adenocarcinoma	TCGA-D7-A4YU	1	1	1	1	1
Stomach adenocarcinoma	TCGA-D7-A4YV	1	0	1	0	1
Stomach adenocarcinoma	TCGA-D7-A4YX	1	0	0	0	0
Stomach adenocarcinoma	TCGA-D7-A4YY	1	1	1	1	1
Stomach adenocarcinoma	TCGA-D7-A4Z0	0	0	0	0	1

Stomach adenocarcinoma	TCGA-D7-A6EV	1	1	0	1	1
Stomach adenocarcinoma	TCGA-D7-A6EX	1	1	1	1	1
Stomach adenocarcinoma	TCGA-D7-A6EY	1	1	1	0	1
Stomach adenocarcinoma	TCGA-D7-A6EZ	1	1	1	1	1
Stomach adenocarcinoma	TCGA-D7-A6F0	1	1	1	1	1
Stomach adenocarcinoma	TCGA-D7-A6F2	1	0	1	1	1
Stomach adenocarcinoma	TCGA-D7-A747	1	1	0	0	0
Stomach adenocarcinoma	TCGA-D7-A748	0	0	0	0	0
Stomach adenocarcinoma	TCGA-D7-A74A	1	1	1	1	1
Stomach adenocarcinoma	TCGA-EQ-8122	1	1	1	1	1
Stomach adenocarcinoma	TCGA-EQ-A4SO	1	1	1	1	1
Stomach adenocarcinoma	TCGA-F1-6177	1	1	0	1	0
Stomach adenocarcinoma	TCGA-F1-6874	0	0	0	0	1
Stomach adenocarcinoma	TCGA-F1-6875	1	1	1	1	1
Stomach adenocarcinoma	TCGA-F1-A448	0	1	0	1	1
Stomach adenocarcinoma	TCGA-F1-A72C	1	1	1	1	1
Stomach adenocarcinoma	TCGA-FP-7735	1	0	1	0	1
Stomach adenocarcinoma	TCGA-FP-7829	1	0	1	1	1
Stomach adenocarcinoma	TCGA-FP-7916	1	0	1	1	1
Stomach adenocarcinoma	TCGA-FP-7998	1	0	0	1	1
Stomach adenocarcinoma	TCGA-FP-8099	1	1	1	1	1
Stomach adenocarcinoma	TCGA-FP-8209	0	0	0	0	0
Stomach adenocarcinoma	TCGA-FP-8210	0	0	0	0	0
Stomach adenocarcinoma	TCGA-FP-8211	1	1	1	1	1
Stomach adenocarcinoma	TCGA-FP-8631	1	1	1	1	1
Stomach adenocarcinoma	TCGA-FP-A4BE	0	0	0	0	0
Stomach adenocarcinoma	TCGA-FP-A4BF	1	1	1	1	1
Stomach adenocarcinoma	TCGA-FP-A8CX	1	1	1	1	1
Stomach adenocarcinoma	TCGA-FP-A9TM	1	1	1	1	1
Stomach adenocarcinoma	TCGA-HF-7131	1	1	1	1	1
Stomach adenocarcinoma	TCGA-HF-7132	1	0	1	1	1
Stomach adenocarcinoma	TCGA-HF-7133	1	1	1	1	1
Stomach adenocarcinoma	TCGA-HF-7134	1	0	0	0	0
Stomach adenocarcinoma	TCGA-HF-7136	1	1	1	1	1
Stomach adenocarcinoma	TCGA-HF-A5NB	1	0	1	0	1
Stomach adenocarcinoma	TCGA-HJ-7597	1	1	1	1	1
Stomach adenocarcinoma	TCGA-HU-8238	1	1	1	1	1
Stomach adenocarcinoma	TCGA-HU-8243	1	1	1	1	1
Stomach adenocarcinoma	TCGA-HU-8244	0	1	0	0	1
Stomach adenocarcinoma	TCGA-HU-8249	1	1	1	1	1
Stomach adenocarcinoma	TCGA-HU-8602	1	1	1	0	1
Stomach adenocarcinoma	TCGA-HU-8604	1	1	1	1	1
Stomach adenocarcinoma	TCGA-HU-8608	1	0	0	0	0
Stomach adenocarcinoma	TCGA-HU-8610	1	1	1	1	1
Stomach adenocarcinoma	TCGA-HU-A4G2	1	0	0	0	0
Stomach adenocarcinoma	TCGA-HU-A4G3	1	0	1	0	1
Stomach adenocarcinoma	TCGA-HU-A4G6	1	1	0	1	0
Stomach adenocarcinoma	TCGA-HU-A4G8	1	1	1	0	1
Stomach adenocarcinoma	TCGA-HU-A4G9	0	0	0	1	1
Stomach adenocarcinoma	TCGA-HU-A4GC	1	1	1	1	1
Stomach adenocarcinoma	TCGA-HU-A4GD	1	1	1	1	1
Stomach adenocarcinoma	TCGA-HU-A4GF	1	1	1	1	1
Stomach adenocarcinoma	TCGA-HU-A4GH	1	1	1	1	1
Stomach adenocarcinoma	TCGA-HU-A4GJ	0	0	0	0	0
Stomach adenocarcinoma	TCGA-HU-A4GN	1	1	1	0	1
Stomach adenocarcinoma	TCGA-HU-A4GP	1	1	1	1	1
Stomach adenocarcinoma	TCGA-HU-A4GQ	1	1	0	0	1
Stomach adenocarcinoma	TCGA-HU-A4GT	1	1	0	1	1

Stomach adenocarcinoma	TCGA-HU-A4GU	1	1	1	1	1
Stomach adenocarcinoma	TCGA-HU-A4GX	1	1	0	0	1
Stomach adenocarcinoma	TCGA-HU-A4GY	1	0	1	1	1
Stomach adenocarcinoma	TCGA-HU-A4H0	1	0	1	1	1
Stomach adenocarcinoma	TCGA-HU-A4H2	1	1	1	1	1
Stomach adenocarcinoma	TCGA-HU-A4H3	1	0	1	0	1
Stomach adenocarcinoma	TCGA-HU-A4H4	1	1	1	1	1
Stomach adenocarcinoma	TCGA-HU-A4H5	1	1	1	1	1
Stomach adenocarcinoma	TCGA-HU-A4H6	1	1	1	1	1
Stomach adenocarcinoma	TCGA-HU-A4H8	0	1	1	1	1
Stomach adenocarcinoma	TCGA-HU-A4HB	1	0	0	0	0
Stomach adenocarcinoma	TCGA-HU-A4HD	1	1	1	1	1
Stomach adenocarcinoma	TCGA-IN-7806	1	1	1	1	1
Stomach adenocarcinoma	TCGA-IN-7808	1	1	1	0	1
Stomach adenocarcinoma	TCGA-IN-8462	1	1	1	1	1
Stomach adenocarcinoma	TCGA-IN-8663	1	1	1	1	1
Stomach adenocarcinoma	TCGA-IN-A6RI	1	1	1	1	1
Stomach adenocarcinoma	TCGA-IN-A6RJ	1	1	1	1	1
Stomach adenocarcinoma	TCGA-IN-A6RL	1	1	1	1	1
Stomach adenocarcinoma	TCGA-IN-A6RN	1	1	1	1	1
Stomach adenocarcinoma	TCGA-IN-A6RO	1	1	1	1	1
Stomach adenocarcinoma	TCGA-IN-A6RR	1	1	1	1	1
Stomach adenocarcinoma	TCGA-IN-A6RS	1	1	1	1	1
Stomach adenocarcinoma	TCGA-IN-A7NR	1	1	1	1	1
Stomach adenocarcinoma	TCGA-IN-A7NT	1	1	1	1	1
Stomach adenocarcinoma	TCGA-IN-A7NU	1	1	1	1	1
Stomach adenocarcinoma	TCGA-IN-AB1V	1	1	1	1	1
Stomach adenocarcinoma	TCGA-IN-AB1X	1	0	1	0	1
Stomach adenocarcinoma	TCGA-IP-7968	1	1	1	1	1
Stomach adenocarcinoma	TCGA-KB-A6F7	1	1	1	1	1
Stomach adenocarcinoma	TCGA-KB-A93G	1	1	1	1	1
Stomach adenocarcinoma	TCGA-KB-A93H	1	1	1	1	1
Stomach adenocarcinoma	TCGA-KB-A93J	1	1	1	1	1
Stomach adenocarcinoma	TCGA-MX-A5UG	1	1	0	1	1
Stomach adenocarcinoma	TCGA-MX-A5UJ	1	1	1	1	0
Stomach adenocarcinoma	TCGA-MX-A663	1	1	1	1	1
Stomach adenocarcinoma	TCGA-MX-A666	1	1	1	1	1
Stomach adenocarcinoma	TCGA-R5-A7O7	1	1	1	1	1
Stomach adenocarcinoma	TCGA-R5-A7ZE	1	1	1	1	1
Stomach adenocarcinoma	TCGA-R5-A7ZF	1	1	1	1	1
Stomach adenocarcinoma	TCGA-R5-A7ZI	1	1	0	1	1
Stomach adenocarcinoma	TCGA-R5-A7ZR	1	1	1	1	1
Stomach adenocarcinoma	TCGA-R5-A805	1	1	1	1	1
Stomach adenocarcinoma	TCGA-RD-A7BS	1	1	0	1	1
Stomach adenocarcinoma	TCGA-RD-A7BT	1	1	1	1	1
Stomach adenocarcinoma	TCGA-RD-A7BW	1	1	1	1	1
Stomach adenocarcinoma	TCGA-RD-A7C1	1	1	1	1	1
Stomach adenocarcinoma	TCGA-RD-A8MV	1	1	1	1	1
Stomach adenocarcinoma	TCGA-RD-A8MW	1	1	1	1	1
Stomach adenocarcinoma	TCGA-RD-A8N0	0	0	0	0	0
Stomach adenocarcinoma	TCGA-RD-A8N1	1	1	1	1	1
Stomach adenocarcinoma	TCGA-RD-A8N2	0	0	0	0	0
Stomach adenocarcinoma	TCGA-RD-A8N4	0	0	0	0	0
Stomach adenocarcinoma	TCGA-RD-A8N5	0	0	1	0	0
Stomach adenocarcinoma	TCGA-RD-A8N6	1	1	1	1	1
Stomach adenocarcinoma	TCGA-RD-A8N9	1	1	1	1	1
Stomach adenocarcinoma	TCGA-RD-A8NB	0	0	0	0	0
Stomach adenocarcinoma	TCGA-SW-A7EA	0	1	1	0	0

Stomach adenocarcinoma	TCGA-SW-A7EB	1	1	1	1	1
Stomach adenocarcinoma	TCGA-VQ-A8DT	1	1	1	1	1
Stomach adenocarcinoma	TCGA-VQ-A8DU	1	1	1	1	1
Stomach adenocarcinoma	TCGA-VQ-A8DV	1	1	1	1	1
Stomach adenocarcinoma	TCGA-VQ-A8DZ	1	1	1	1	1
Stomach adenocarcinoma	TCGA-VQ-A8E0	1	1	1	1	1
Stomach adenocarcinoma	TCGA-VQ-A8E2	1	1	1	1	1
Stomach adenocarcinoma	TCGA-VQ-A8E3	1	0	0	0	1
Stomach adenocarcinoma	TCGA-VQ-A8E7	1	1	1	1	1
Stomach adenocarcinoma	TCGA-VQ-A8P2	1	1	1	1	1
Stomach adenocarcinoma	TCGA-VQ-A8P3	1	1	1	0	1
Stomach adenocarcinoma	TCGA-VQ-A8P5	1	1	1	1	1
Stomach adenocarcinoma	TCGA-VQ-A8P8	0	0	0	0	0
Stomach adenocarcinoma	TCGA-VQ-A8PB	0	0	0	0	0
Stomach adenocarcinoma	TCGA-VQ-A8PC	1	1	1	1	1
Stomach adenocarcinoma	TCGA-VQ-A8PD	1	0	0	0	0
Stomach adenocarcinoma	TCGA-VQ-A8PE	1	1	1	1	1
Stomach adenocarcinoma	TCGA-VQ-A8PF	1	0	0	0	1
Stomach adenocarcinoma	TCGA-VQ-A8PH	1	1	1	1	1
Stomach adenocarcinoma	TCGA-VQ-A8PJ	1	1	1	1	1
Stomach adenocarcinoma	TCGA-VQ-A8PK	1	1	1	1	1
Stomach adenocarcinoma	TCGA-VQ-A8PM	1	1	1	1	1
Stomach adenocarcinoma	TCGA-VQ-A8PO	0	0	0	1	0
Stomach adenocarcinoma	TCGA-VQ-A8PP	0	0	0	0	0
Stomach adenocarcinoma	TCGA-VQ-A8PQ	0	0	0	0	0
Stomach adenocarcinoma	TCGA-VQ-A8PU	1	1	1	1	1
Stomach adenocarcinoma	TCGA-VQ-A8PX	1	1	1	1	1
Stomach adenocarcinoma	TCGA-VQ-A91A	1	1	1	1	1
Stomach adenocarcinoma	TCGA-VQ-A91D	0	0	0	0	0
Stomach adenocarcinoma	TCGA-VQ-A91E	0	0	0	0	0
Stomach adenocarcinoma	TCGA-VQ-A91K	1	1	0	1	0
Stomach adenocarcinoma	TCGA-VQ-A91N	1	1	1	1	1
Stomach adenocarcinoma	TCGA-VQ-A91Q	1	1	1	1	1
Stomach adenocarcinoma	TCGA-VQ-A91S	1	0	0	1	1
Stomach adenocarcinoma	TCGA-VQ-A91U	1	1	1	1	1
Stomach adenocarcinoma	TCGA-VQ-A91V	1	1	1	1	1
Stomach adenocarcinoma	TCGA-VQ-A91W	0	0	0	0	0
Stomach adenocarcinoma	TCGA-VQ-A91X	1	1	1	1	1
Stomach adenocarcinoma	TCGA-VQ-A91Y	1	1	1	1	1
Stomach adenocarcinoma	TCGA-VQ-A91Z	1	1	1	1	1
Stomach adenocarcinoma	TCGA-VQ-A922	1	1	1	1	1
Stomach adenocarcinoma	TCGA-VQ-A923	1	0	1	1	1
Stomach adenocarcinoma	TCGA-VQ-A924	0	0	0	0	0
Stomach adenocarcinoma	TCGA-VQ-A925	1	1	1	1	1
Stomach adenocarcinoma	TCGA-VQ-A927	0	0	0	0	0
Stomach adenocarcinoma	TCGA-VQ-A928	1	0	1	1	1
Stomach adenocarcinoma	TCGA-VQ-A92D	1	1	1	1	1
Stomach adenocarcinoma	TCGA-VQ-A94O	1	1	1	1	1
Stomach adenocarcinoma	TCGA-VQ-A94P	0	0	0	0	0
Stomach adenocarcinoma	TCGA-VQ-A94R	1	1	1	1	1
Stomach adenocarcinoma	TCGA-VQ-A94T	1	1	1	1	1
Stomach adenocarcinoma	TCGA-VQ-A94U	1	1	1	1	1
Stomach adenocarcinoma	TCGA-VQ-AA64	1	1	1	1	1
Stomach adenocarcinoma	TCGA-VQ-AA68	1	1	1	1	1
Stomach adenocarcinoma	TCGA-VQ-AA69	1	0	0	0	0
Stomach adenocarcinoma	TCGA-VQ-AA6A	1	1	1	1	1
Stomach adenocarcinoma	TCGA-VQ-AA6B	1	1	1	1	1
Stomach adenocarcinoma	TCGA-VQ-AA6D	1	1	1	1	1

Stomach adenocarcinoma	TCGA-VQ-AA6F	1	1	1	1	1
Stomach adenocarcinoma	TCGA-VQ-AA6G	1	1	1	1	1
Stomach adenocarcinoma	TCGA-VQ-AA6I	1	1	1	1	1
Stomach adenocarcinoma	TCGA-VQ-AA6J	1	1	1	1	1
Stomach adenocarcinoma	TCGA-VQ-AA6K	1	1	1	1	1
Stomach adenocarcinoma	TCGA-ZA-A8F6	0	0	1	0	1
Stomach adenocarcinoma	TCGA-ZQ-A9CR	1	1	1	1	1
Testicular Germ Cell cancer	TCGA-2G-AAEW	1	1	1	1	1
Testicular Germ Cell cancer	TCGA-2G-AAEX	1	0	1	0	1
Testicular Germ Cell cancer	TCGA-2G-AAF1	1	0	0	0	1
Testicular Germ Cell cancer	TCGA-2G-AAF4	1	1	1	1	1
Testicular Germ Cell cancer	TCGA-2G-AAF6	1	1	1	1	1
Testicular Germ Cell cancer	TCGA-2G-AAF8	1	1	1	0	1
Testicular Germ Cell cancer	TCGA-2G-AAFE	1	1	1	1	1
Testicular Germ Cell cancer	TCGA-2G-AAFG	1	1	1	1	1
Testicular Germ Cell cancer	TCGA-2G-AAFG	0	0	0	0	0
Testicular Germ Cell cancer	TCGA-2G-AAFH	1	1	1	0	1
Testicular Germ Cell cancer	TCGA-2G-AAFI	1	1	1	1	1
Testicular Germ Cell cancer	TCGA-2G-AAFJ	1	1	1	1	1
Testicular Germ Cell cancer	TCGA-2G-AAFL	1	1	0	1	1
Testicular Germ Cell cancer	TCGA-2G-AAFM	1	1	1	1	1
Testicular Germ Cell cancer	TCGA-2G-AAFN	1	1	1	1	1
Testicular Germ Cell cancer	TCGA-2G-AAFO	1	1	1	0	1
Testicular Germ Cell cancer	TCGA-2G-AAFV	0	0	1	0	0
Testicular Germ Cell cancer	TCGA-2G-AAFY	1	1	1	1	1
Testicular Germ Cell cancer	TCGA-2G-AAFZ	0	0	0	1	1
Testicular Germ Cell cancer	TCGA-2G-AAG0	1	1	1	1	1
Testicular Germ Cell cancer	TCGA-2G-AAG3	1	1	1	1	1
Testicular Germ Cell cancer	TCGA-2G-AAG5	1	0	0	0	0
Testicular Germ Cell cancer	TCGA-2G-AAG6	1	1	1	1	1
Testicular Germ Cell cancer	TCGA-2G-AAG7	1	1	1	1	1
Testicular Germ Cell cancer	TCGA-2G-AAG8	1	1	1	1	1
Testicular Germ Cell cancer	TCGA-2G-AAG9	1	1	1	1	1
Testicular Germ Cell cancer	TCGA-2G-AAGA	1	1	1	1	1
Testicular Germ Cell cancer	TCGA-2G-AAGC	1	1	1	1	1
Testicular Germ Cell cancer	TCGA-2G-AAGE	1	1	1	1	1
Testicular Germ Cell cancer	TCGA-2G-AAGF	1	1	1	1	1
Testicular Germ Cell cancer	TCGA-2G-AAGG	1	1	1	1	1
Testicular Germ Cell cancer	TCGA-2G-AAGI	1	0	1	1	1
Testicular Germ Cell cancer	TCGA-2G-AAGI	0	0	0	0	0
Testicular Germ Cell cancer	TCGA-2G-AAGJ	1	1	1	1	1
Testicular Germ Cell cancer	TCGA-2G-AAGK	1	1	1	0	1
Testicular Germ Cell cancer	TCGA-2G-AAGM	1	1	1	1	1
Testicular Germ Cell cancer	TCGA-2G-AAGN	1	1	1	1	1
Testicular Germ Cell cancer	TCGA-2G-AAGO	1	1	1	1	1
Testicular Germ Cell cancer	TCGA-2G-AAGP	1	1	1	1	1
Testicular Germ Cell cancer	TCGA-2G-AAGS	1	1	1	1	1
Testicular Germ Cell cancer	TCGA-2G-AAGT	1	1	1	0	1
Testicular Germ Cell cancer	TCGA-2G-AAGV	1	1	1	1	1
Testicular Germ Cell cancer	TCGA-2G-AAGW	1	1	1	1	1
Testicular Germ Cell cancer	TCGA-2G-AAGX	1	1	1	0	1
Testicular Germ Cell cancer	TCGA-2G-AAGY	1	1	1	0	1
Testicular Germ Cell cancer	TCGA-2G-AAGY	0	0	0	0	0
Testicular Germ Cell cancer	TCGA-2G-AAGZ	1	1	1	0	1
Testicular Germ Cell cancer	TCGA-2G-AAH0	1	1	1	1	1
Testicular Germ Cell cancer	TCGA-2G-AAH2	1	1	1	1	1
Testicular Germ Cell cancer	TCGA-2G-AAH3	1	1	1	1	1
Testicular Germ Cell cancer	TCGA-2G-AAH4	1	1	1	1	1

Testicular Germ Cell cancer	TCGA-2G-AAH8	1	1	1	0	1
Testicular Germ Cell cancer	TCGA-2G-AAHA	1	0	0	0	0
Testicular Germ Cell cancer	TCGA-2G-AAHC	1	1	1	1	1
Testicular Germ Cell cancer	TCGA-2G-AAHG	1	1	1	0	1
Testicular Germ Cell cancer	TCGA-2G-AAHL	1	1	1	1	1
Testicular Germ Cell cancer	TCGA-2G-AAHN	1	1	1	1	1
Testicular Germ Cell cancer	TCGA-2G-AAHP	1	1	1	1	1
Testicular Germ Cell cancer	TCGA-2G-AAHP	0	0	0	0	0
Testicular Germ Cell cancer	TCGA-2G-AAHT	1	1	1	0	1
Testicular Germ Cell cancer	TCGA-2G-AAKD	1	1	1	1	1
Testicular Germ Cell cancer	TCGA-2G-AAKG	1	1	1	1	1
Testicular Germ Cell cancer	TCGA-2G-AAKG	0	0	0	0	0
Testicular Germ Cell cancer	TCGA-2G-AAKH	1	1	0	1	1
Testicular Germ Cell cancer	TCGA-2G-AAKL	1	1	1	1	1
Testicular Germ Cell cancer	TCGA-2G-AAKM	1	1	1	1	1
Testicular Germ Cell cancer	TCGA-2G-AAKO	1	0	0	1	0
Testicular Germ Cell cancer	TCGA-2G-AAKO	0	0	0	0	0
Testicular Germ Cell cancer	TCGA-2G-AAL5	1	1	1	1	1
Testicular Germ Cell cancer	TCGA-2G-AAL7	1	1	1	1	1
Testicular Germ Cell cancer	TCGA-2G-AALF	1	1	1	1	1
Testicular Germ Cell cancer	TCGA-2G-AALG	1	1	1	1	1
Testicular Germ Cell cancer	TCGA-2G-AALN	1	1	1	1	1
Testicular Germ Cell cancer	TCGA-2G-AALO	0	1	0	0	1
Testicular Germ Cell cancer	TCGA-2G-AALP	1	1	1	1	1
Testicular Germ Cell cancer	TCGA-2G-AALQ	1	1	1	1	1
Testicular Germ Cell cancer	TCGA-2G-AALR	1	1	0	1	1
Testicular Germ Cell cancer	TCGA-2G-AALS	1	1	1	1	1
Testicular Germ Cell cancer	TCGA-2G-AALT	1	1	1	0	1
Testicular Germ Cell cancer	TCGA-2G-AALW	1	1	1	1	1
Testicular Germ Cell cancer	TCGA-2G-AALX	1	0	1	0	1
Testicular Germ Cell cancer	TCGA-2G-AALY	1	1	1	1	1
Testicular Germ Cell cancer	TCGA-2G-AALZ	1	1	1	1	1
Testicular Germ Cell cancer	TCGA-2G-AAM2	1	1	1	1	1
Testicular Germ Cell cancer	TCGA-2G-AAM3	1	1	1	1	1
Testicular Germ Cell cancer	TCGA-2G-AAM4	0	1	0	0	0
Testicular Germ Cell cancer	TCGA-2X-A9D5	1	1	1	1	1
Testicular Germ Cell cancer	TCGA-2X-A9D6	1	1	1	0	1
Testicular Germ Cell cancer	TCGA-4K-AA1G	1	1	1	1	1
Testicular Germ Cell cancer	TCGA-4K-AA1H	1	0	0	0	1
Testicular Germ Cell cancer	TCGA-4K-AA1I	1	1	1	0	1
Testicular Germ Cell cancer	TCGA-4K-AAAL	1	1	1	0	1
Testicular Germ Cell cancer	TCGA-S6-A8JW	1	0	0	0	1
Testicular Germ Cell cancer	TCGA-S6-A8JX	1	1	1	0	1
Testicular Germ Cell cancer	TCGA-S6-A8JY	1	1	1	0	1
Testicular Germ Cell cancer	TCGA-SB-A6J6	1	1	1	1	1
Testicular Germ Cell cancer	TCGA-SB-A76C	1	1	1	1	1
Testicular Germ Cell cancer	TCGA-SN-A6IS	1	1	1	1	1
Testicular Germ Cell cancer	TCGA-SN-A84W	1	1	1	1	1
Testicular Germ Cell cancer	TCGA-SN-A84X	0	0	0	1	0
Testicular Germ Cell cancer	TCGA-SN-A84Y	1	1	1	1	1
Testicular Germ Cell cancer	TCGA-SO-A8JP	1	1	1	1	1
Testicular Germ Cell cancer	TCGA-VF-A8A8	1	1	1	0	1
Testicular Germ Cell cancer	TCGA-VF-A8A9	1	1	1	1	1
Testicular Germ Cell cancer	TCGA-VF-A8AA	1	0	0	0	0
Testicular Germ Cell cancer	TCGA-VF-A8AB	1	1	1	1	1
Testicular Germ Cell cancer	TCGA-VF-A8AC	1	1	1	1	1
Testicular Germ Cell cancer	TCGA-VF-A8AD	1	1	1	1	1
Testicular Germ Cell cancer	TCGA-VF-A8AE	1	1	1	1	1

Testicular Germ Cell cancer	TCGA-W4-A7U2	1	1	1	1	1
Testicular Germ Cell cancer	TCGA-W4-A7U3	1	1	1	0	1
Testicular Germ Cell cancer	TCGA-W4-A7U4	1	1	1	0	1
Testicular Germ Cell cancer	TCGA-WZ-A7V3	1	1	0	1	1
Testicular Germ Cell cancer	TCGA-WZ-A7V4	1	1	1	1	1
Testicular Germ Cell cancer	TCGA-WZ-A7V5	1	1	1	1	1
Testicular Germ Cell cancer	TCGA-WZ-A8D5	1	1	1	1	1
Testicular Germ Cell cancer	TCGA-X3-A8G4	1	1	1	1	1
Testicular Germ Cell cancer	TCGA-XE-A8H1	1	1	1	1	1
Testicular Germ Cell cancer	TCGA-XE-A8H4	1	1	1	0	1
Testicular Germ Cell cancer	TCGA-XE-A8H5	1	1	1	1	1
Testicular Germ Cell cancer	TCGA-XE-A9SE	1	1	1	0	1
Testicular Germ Cell cancer	TCGA-XE-AANI	1	1	1	1	1
Testicular Germ Cell cancer	TCGA-XE-AANJ	1	1	1	1	1
Testicular Germ Cell cancer	TCGA-XE-AANR	1	1	0	1	1
Testicular Germ Cell cancer	TCGA-XE-AANV	1	0	0	0	1
Testicular Germ Cell cancer	TCGA-XE-AAO3	1	1	1	0	1
Testicular Germ Cell cancer	TCGA-XE-AAO4	1	1	1	1	1
Testicular Germ Cell cancer	TCGA-XE-AAO6	1	1	1	1	1
Testicular Germ Cell cancer	TCGA-XE-AAOB	1	1	1	0	1
Testicular Germ Cell cancer	TCGA-XE-AAOC	0	0	0	0	0
Testicular Germ Cell cancer	TCGA-XE-AAOD	1	1	1	0	1
Testicular Germ Cell cancer	TCGA-XE-AAOF	1	1	1	1	1
Testicular Germ Cell cancer	TCGA-XE-AAOJ	1	1	1	1	1
Testicular Germ Cell cancer	TCGA-XE-AAOL	1	1	1	1	1
Testicular Germ Cell cancer	TCGA-XY-A89B	1	1	1	1	1
Testicular Germ Cell cancer	TCGA-XY-A8S2	1	1	1	1	1
Testicular Germ Cell cancer	TCGA-XY-A8S3	1	1	1	0	1
Testicular Germ Cell cancer	TCGA-XY-A9T9	1	1	1	1	1
Testicular Germ Cell cancer	TCGA-YU-A90P	1	1	1	1	1
Testicular Germ Cell cancer	TCGA-YU-A90Q	1	1	1	1	1
Testicular Germ Cell cancer	TCGA-YU-A90S	0	0	0	0	0
Testicular Germ Cell cancer	TCGA-YU-A90W	1	1	1	0	1
Testicular Germ Cell cancer	TCGA-YU-A90Y	1	1	1	1	1
Testicular Germ Cell cancer	TCGA-YU-A912	1	1	1	1	1
Testicular Germ Cell cancer	TCGA-YU-A94D	0	0	0	0	0
Testicular Germ Cell cancer	TCGA-YU-A94I	1	1	1	1	1
Testicular Germ Cell cancer	TCGA-YU-AA4L	1	1	1	1	1
Testicular Germ Cell cancer	TCGA-YU-AA61	1	1	1	1	1
Testicular Germ Cell cancer	TCGA-ZM-AA05	1	1	1	1	1
Testicular Germ Cell cancer	TCGA-ZM-AA06	1	1	1	1	1
Testicular Germ Cell cancer	TCGA-ZM-AA0B	1	1	1	1	1
Testicular Germ Cell cancer	TCGA-ZM-AA0D	1	1	1	1	1
Testicular Germ Cell cancer	TCGA-ZM-AA0E	1	1	0	0	1
Testicular Germ Cell cancer	TCGA-ZM-AA0F	1	1	1	0	1
Testicular Germ Cell cancer	TCGA-ZM-AA0H	1	1	1	1	1
Testicular Germ Cell cancer	TCGA-ZM-AA0N	1	1	1	1	1
Thymoma	TCGA-3G-AB0O	0	0	0	0	0
Thymoma	TCGA-3G-AB0Q	0	0	0	0	0
Thymoma	TCGA-3G-AB0T	0	0	0	0	0
Thymoma	TCGA-3G-AB14	0	0	0	1	1
Thymoma	TCGA-3G-AB19	0	0	0	1	1
Thymoma	TCGA-3Q-A9WF	0	0	0	0	0
Thymoma	TCGA-3S-A8YW	1	0	1	1	1
Thymoma	TCGA-3S-AAYX	0	0	0	0	0
Thymoma	TCGA-3T-AA9L	0	0	0	0	0
Thymoma	TCGA-4V-A9QI	0	0	0	0	1
Thymoma	TCGA-4V-A9QJ	1	0	1	1	1

Thymoma	TCGA-4V-A9QL	0	0	0	0	0
Thymoma	TCGA-4V-A9QM	0	0	0	0	0
Thymoma	TCGA-4V-A9QN	1	1	1	1	1
Thymoma	TCGA-4V-A9QR	0	0	1	0	0
Thymoma	TCGA-4V-A9QS	0	0	0	0	0
Thymoma	TCGA-4V-A9QT	0	0	0	0	0
Thymoma	TCGA-4V-A9QU	0	0	0	0	0
Thymoma	TCGA-4V-A9QW	0	0	0	0	0
Thymoma	TCGA-4V-A9QX	0	0	0	1	1
Thymoma	TCGA-4X-A9F9	0	0	0	0	0
Thymoma	TCGA-4X-A9FA	0	0	0	0	0
Thymoma	TCGA-4X-A9FB	0	0	0	0	0
Thymoma	TCGA-4X-A9FC	0	0	0	0	0
Thymoma	TCGA-4X-A9FD	1	1	1	1	1
Thymoma	TCGA-5G-A9ZZ	0	0	1	0	1
Thymoma	TCGA-5K-AAAP	0	0	0	1	1
Thymoma	TCGA-5U-AB0D	1	1	1	1	1
Thymoma	TCGA-5U-AB0E	1	1	1	1	1
Thymoma	TCGA-5U-AB0F	0	0	0	1	1
Thymoma	TCGA-5V-A9RR	0	0	0	0	0
Thymoma	TCGA-X7-A8D6	0	0	0	0	0
Thymoma	TCGA-X7-A8D7	1	0	0	0	0
Thymoma	TCGA-X7-A8D8	0	0	0	0	0
Thymoma	TCGA-X7-A8D9	1	0	0	0	1
Thymoma	TCGA-X7-A8DB	0	0	0	0	0
Thymoma	TCGA-X7-A8DD	0	0	0	0	0
Thymoma	TCGA-X7-A8DE	0	0	0	0	0
Thymoma	TCGA-X7-A8DF	0	0	0	0	0
Thymoma	TCGA-X7-A8DG	0	0	0	0	0
Thymoma	TCGA-X7-A8DJ	0	0	0	0	0
Thymoma	TCGA-X7-A8M0	1	0	1	1	1
Thymoma	TCGA-X7-A8M1	0	1	0	0	0
Thymoma	TCGA-X7-A8M3	0	1	0	0	1
Thymoma	TCGA-X7-A8M4	0	0	0	0	0
Thymoma	TCGA-X7-A8M5	0	0	0	0	0
Thymoma	TCGA-X7-A8M6	0	0	0	0	0
Thymoma	TCGA-X7-A8M7	0	0	0	0	0
Thymoma	TCGA-X7-A8M8	0	0	0	0	0
Thymoma	TCGA-XH-A853	0	1	0	1	0
Thymoma	TCGA-XM-A8R8	0	0	0	0	0
Thymoma	TCGA-XM-A8R9	0	0	0	0	0
Thymoma	TCGA-XM-A8RB	0	0	0	0	0
Thymoma	TCGA-XM-A8RC	1	0	1	1	1
Thymoma	TCGA-XM-A8RD	1	0	1	0	0
Thymoma	TCGA-XM-A8RE	0	0	0	0	0
Thymoma	TCGA-XM-A8RF	0	1	0	1	1
Thymoma	TCGA-XM-A8RG	1	0	1	0	1
Thymoma	TCGA-XM-A8RH	0	0	0	0	0
Thymoma	TCGA-XM-A8RI	0	0	0	0	0
Thymoma	TCGA-XM-A8RL	0	0	0	0	0
Thymoma	TCGA-XM-AAZ1	0	0	0	1	0
Thymoma	TCGA-XM-AAZ2	0	0	0	0	1
Thymoma	TCGA-XM-AAZ3	0	0	0	0	0
Thymoma	TCGA-XU-A92O	0	0	0	1	0
Thymoma	TCGA-XU-A92Q	0	0	0	0	0
Thymoma	TCGA-XU-A92T	0	0	0	0	0
Thymoma	TCGA-XU-A92U	0	0	0	0	0
Thymoma	TCGA-XU-A92V	0	0	0	0	1

Thymoma	TCGA-XU-A92W	0	0	0	0	0
Thymoma	TCGA-XU-A92X	1	1	1	1	1
Thymoma	TCGA-XU-A92Y	0	0	0	0	0
Thymoma	TCGA-XU-A92Z	0	0	0	1	1
Thymoma	TCGA-XU-A930	1	1	1	1	1
Thymoma	TCGA-XU-A931	1	0	1	0	0
Thymoma	TCGA-XU-A932	0	0	1	0	0
Thymoma	TCGA-XU-A933	0	1	1	1	1
Thymoma	TCGA-XU-A936	1	1	1	1	1
Thymoma	TCGA-XU-AAXV	0	0	0	0	0
Thymoma	TCGA-XU-AAXW	0	0	0	0	0
Thymoma	TCGA-XU-AAXX	0	0	0	0	0
Thymoma	TCGA-XU-AAXY	1	0	0	0	0
Thymoma	TCGA-XU-AAXZ	0	0	0	0	0
Thymoma	TCGA-XU-AAY0	0	0	0	0	0
Thymoma	TCGA-XU-AAY1	0	0	0	0	0
Thymoma	TCGA-YT-A95D	0	0	0	0	0
Thymoma	TCGA-YT-A95E	0	0	0	0	0
Thymoma	TCGA-YT-A95F	0	0	0	0	0
Thymoma	TCGA-YT-A95G	1	1	1	1	1
Thymoma	TCGA-YT-A95H	0	0	0	0	0
Thymoma	TCGA-ZB-A961	1	0	1	0	1
Thymoma	TCGA-ZB-A962	0	1	1	0	1
Thymoma	TCGA-ZB-A963	0	0	0	1	1
Thymoma	TCGA-ZB-A964	0	0	0	0	0
Thymoma	TCGA-ZB-A965	0	0	0	0	0
Thymoma	TCGA-ZB-A966	0	1	0	1	1
Thymoma	TCGA-ZB-A969	0	1	0	1	1
Thymoma	TCGA-ZB-A96A	0	0	0	1	1
Thymoma	TCGA-ZB-A96B	0	0	0	0	0
Thymoma	TCGA-ZB-A96C	0	0	0	0	0
Thymoma	TCGA-ZB-A96D	0	0	0	0	0
Thymoma	TCGA-ZB-A96E	0	0	0	0	0
Thymoma	TCGA-ZB-A96F	1	0	1	0	1
Thymoma	TCGA-ZB-A96G	0	0	0	0	0
Thymoma	TCGA-ZB-A96H	0	1	0	0	1
Thymoma	TCGA-ZB-A96I	1	0	1	1	0
Thymoma	TCGA-ZB-A96K	1	0	1	0	1
Thymoma	TCGA-ZB-A96L	1	1	1	1	1
Thymoma	TCGA-ZB-A96M	0	0	0	0	0
Thymoma	TCGA-ZB-A96O	0	0	0	0	0
Thymoma	TCGA-ZB-A96P	0	0	0	0	0
Thymoma	TCGA-ZB-A96Q	0	0	0	0	0
Thymoma	TCGA-ZB-A96R	0	0	0	0	0
Thymoma	TCGA-ZB-A96V	0	0	0	0	1
Thymoma	TCGA-ZC-AAA7	0	0	1	0	1
Thymoma	TCGA-ZC-AAAA	1	1	1	1	1
Thymoma	TCGA-ZC-AAAF	0	0	0	1	1
Thymoma	TCGA-ZC-AAAH	0	0	0	0	0
Thymoma	TCGA-ZL-A9V6	0	0	0	0	0
Thymoma	TCGA-ZT-A8OM	0	0	0	1	1
Thyroid Carcinoma	TCGA-4C-A93U	1	1	0	1	1
Thyroid Carcinoma	TCGA-BJ-A0YZ	1	0	0	0	0
Thyroid Carcinoma	TCGA-BJ-A0Z0	1	0	0	0	1
Thyroid Carcinoma	TCGA-BJ-A0Z2	0	0	0	1	1
Thyroid Carcinoma	TCGA-BJ-A0Z3	0	0	0	1	0
Thyroid Carcinoma	TCGA-BJ-A0Z5	0	0	0	1	1
Thyroid Carcinoma	TCGA-BJ-A0Z9	0	0	0	1	0

Thyroid Carcinoma	TCGA-BJ-A0ZA	0	0	0	1	0
Thyroid Carcinoma	TCGA-BJ-A0ZB	0	0	0	1	0
Thyroid Carcinoma	TCGA-BJ-A0ZC	1	0	0	0	1
Thyroid Carcinoma	TCGA-BJ-A0ZE	0	0	0	0	0
Thyroid Carcinoma	TCGA-BJ-A0ZG	0	0	0	0	0
Thyroid Carcinoma	TCGA-BJ-A0ZH	0	0	0	1	1
Thyroid Carcinoma	TCGA-BJ-A0ZJ	0	0	0	0	0
Thyroid Carcinoma	TCGA-BJ-A18Y	0	0	0	1	1
Thyroid Carcinoma	TCGA-BJ-A18Z	0	0	0	1	0
Thyroid Carcinoma	TCGA-BJ-A190	1	1	0	1	1
Thyroid Carcinoma	TCGA-BJ-A191	0	0	0	0	0
Thyroid Carcinoma	TCGA-BJ-A192	0	0	1	0	1
Thyroid Carcinoma	TCGA-BJ-A28R	0	0	0	1	0
Thyroid Carcinoma	TCGA-BJ-A28S	0	0	0	1	0
Thyroid Carcinoma	TCGA-BJ-A28T	0	0	0	0	1
Thyroid Carcinoma	TCGA-BJ-A28V	0	0	0	0	0
Thyroid Carcinoma	TCGA-BJ-A28W	0	0	0	0	0
Thyroid Carcinoma	TCGA-BJ-A28X	0	0	0	1	0
Thyroid Carcinoma	TCGA-BJ-A28Z	0	0	0	0	0
Thyroid Carcinoma	TCGA-BJ-A290	0	0	0	1	0
Thyroid Carcinoma	TCGA-BJ-A291	0	0	0	0	0
Thyroid Carcinoma	TCGA-BJ-A2N7	0	0	0	1	0
Thyroid Carcinoma	TCGA-BJ-A2N8	1	1	0	1	1
Thyroid Carcinoma	TCGA-BJ-A2N9	0	0	0	0	0
Thyroid Carcinoma	TCGA-BJ-A2NA	0	0	0	1	0
Thyroid Carcinoma	TCGA-BJ-A2P4	0	0	0	1	1
Thyroid Carcinoma	TCGA-BJ-A3EZ	1	0	0	1	0
Thyroid Carcinoma	TCGA-BJ-A3F0	0	0	0	1	1
Thyroid Carcinoma	TCGA-BJ-A3PR	0	0	0	1	0
Thyroid Carcinoma	TCGA-BJ-A3PT	0	0	0	1	1
Thyroid Carcinoma	TCGA-BJ-A3PU	0	0	0	1	0
Thyroid Carcinoma	TCGA-BJ-A45C	0	0	0	0	0
Thyroid Carcinoma	TCGA-BJ-A45D	0	0	0	1	1
Thyroid Carcinoma	TCGA-BJ-A45E	0	0	0	0	0
Thyroid Carcinoma	TCGA-BJ-A45F	0	0	0	1	1
Thyroid Carcinoma	TCGA-BJ-A45G	0	0	0	1	0
Thyroid Carcinoma	TCGA-BJ-A45H	0	0	0	0	0
Thyroid Carcinoma	TCGA-BJ-A45I	0	0	0	1	0
Thyroid Carcinoma	TCGA-BJ-A45J	0	0	0	1	0
Thyroid Carcinoma	TCGA-BJ-A45K	0	0	0	0	0
Thyroid Carcinoma	TCGA-BJ-A4O8	0	1	0	1	0
Thyroid Carcinoma	TCGA-BJ-A4O9	1	0	0	1	0
Thyroid Carcinoma	TCGA-CE-A13K	0	0	0	0	0
Thyroid Carcinoma	TCGA-CE-A27D	0	0	0	0	0
Thyroid Carcinoma	TCGA-CE-A3MD	0	0	0	0	0
Thyroid Carcinoma	TCGA-CE-A3ME	0	0	0	1	0
Thyroid Carcinoma	TCGA-CE-A481	0	0	0	0	0
Thyroid Carcinoma	TCGA-CE-A482	0	0	0	0	0
Thyroid Carcinoma	TCGA-CE-A483	0	0	0	0	0
Thyroid Carcinoma	TCGA-CE-A484	0	0	0	1	1
Thyroid Carcinoma	TCGA-CE-A485	0	0	0	0	0
Thyroid Carcinoma	TCGA-DE-A0XZ	1	0	0	1	1
Thyroid Carcinoma	TCGA-DE-A0Y2	0	0	0	0	0
Thyroid Carcinoma	TCGA-DE-A0Y3	0	0	0	1	0
Thyroid Carcinoma	TCGA-DE-A2OL	0	0	0	0	0
Thyroid Carcinoma	TCGA-DE-A3KN	1	0	0	0	1
Thyroid Carcinoma	TCGA-DE-A4M8	1	0	0	1	1
Thyroid Carcinoma	TCGA-DE-A4M9	0	0	0	0	0

Thyroid Carcinoma	TCGA-DE-A4MA	0	0	0	0	0
Thyroid Carcinoma	TCGA-DE-A4MB	1	1	0	0	0
Thyroid Carcinoma	TCGA-DE-A4MC	0	0	0	0	0
Thyroid Carcinoma	TCGA-DE-A4MD	0	0	0	0	0
Thyroid Carcinoma	TCGA-DE-A4MD	0	0	0	0	1
Thyroid Carcinoma	TCGA-DE-A69J	0	0	0	0	0
Thyroid Carcinoma	TCGA-DE-A69K	0	0	0	0	0
Thyroid Carcinoma	TCGA-DE-A7U5	0	0	0	0	0
Thyroid Carcinoma	TCGA-DJ-A13L	1	1	0	1	0
Thyroid Carcinoma	TCGA-DJ-A13M	0	0	0	1	1
Thyroid Carcinoma	TCGA-DJ-A13O	0	0	0	1	0
Thyroid Carcinoma	TCGA-DJ-A13P	0	0	0	1	0
Thyroid Carcinoma	TCGA-DJ-A13R	0	0	0	0	0
Thyroid Carcinoma	TCGA-DJ-A13S	0	0	0	1	0
Thyroid Carcinoma	TCGA-DJ-A13T	0	0	0	1	1
Thyroid Carcinoma	TCGA-DJ-A13U	0	0	0	1	0
Thyroid Carcinoma	TCGA-DJ-A13V	0	0	0	1	0
Thyroid Carcinoma	TCGA-DJ-A13W	0	0	0	0	0
Thyroid Carcinoma	TCGA-DJ-A13X	0	0	0	1	0
Thyroid Carcinoma	TCGA-DJ-A1QD	0	0	0	1	0
Thyroid Carcinoma	TCGA-DJ-A1QE	1	0	0	1	0
Thyroid Carcinoma	TCGA-DJ-A1QF	0	0	0	1	0
Thyroid Carcinoma	TCGA-DJ-A1QG	0	0	0	1	1
Thyroid Carcinoma	TCGA-DJ-A1QH	0	0	0	1	0
Thyroid Carcinoma	TCGA-DJ-A1QI	0	0	0	1	0
Thyroid Carcinoma	TCGA-DJ-A1QL	0	0	0	0	0
Thyroid Carcinoma	TCGA-DJ-A1QM	0	0	0	1	0
Thyroid Carcinoma	TCGA-DJ-A1QN	0	0	0	1	1
Thyroid Carcinoma	TCGA-DJ-A1QO	0	0	0	1	0
Thyroid Carcinoma	TCGA-DJ-A1QQ	0	0	0	1	0
Thyroid Carcinoma	TCGA-DJ-A2PN	0	0	0	1	0
Thyroid Carcinoma	TCGA-DJ-A2PO	0	0	0	1	0
Thyroid Carcinoma	TCGA-DJ-A2PP	0	0	0	1	0
Thyroid Carcinoma	TCGA-DJ-A2PQ	0	0	0	1	0
Thyroid Carcinoma	TCGA-DJ-A2PR	0	0	0	1	0
Thyroid Carcinoma	TCGA-DJ-A2PS	0	1	0	1	1
Thyroid Carcinoma	TCGA-DJ-A2PT	0	0	0	1	0
Thyroid Carcinoma	TCGA-DJ-A2PU	0	0	0	1	0
Thyroid Carcinoma	TCGA-DJ-A2PV	1	0	0	1	0
Thyroid Carcinoma	TCGA-DJ-A2PW	0	0	0	1	0
Thyroid Carcinoma	TCGA-DJ-A2PX	0	0	0	0	0
Thyroid Carcinoma	TCGA-DJ-A2PY	0	0	0	1	0
Thyroid Carcinoma	TCGA-DJ-A2PZ	0	0	0	1	0
Thyroid Carcinoma	TCGA-DJ-A2Q0	1	1	1	1	1
Thyroid Carcinoma	TCGA-DJ-A2Q1	0	0	0	0	0
Thyroid Carcinoma	TCGA-DJ-A2Q2	0	0	0	0	0
Thyroid Carcinoma	TCGA-DJ-A2Q3	0	0	0	1	0
Thyroid Carcinoma	TCGA-DJ-A2Q4	0	0	0	1	0
Thyroid Carcinoma	TCGA-DJ-A2Q5	0	0	0	1	0
Thyroid Carcinoma	TCGA-DJ-A2Q6	0	0	0	1	0
Thyroid Carcinoma	TCGA-DJ-A2Q7	0	0	0	1	1
Thyroid Carcinoma	TCGA-DJ-A2Q9	0	0	0	1	0
Thyroid Carcinoma	TCGA-DJ-A2QA	0	0	0	1	1
Thyroid Carcinoma	TCGA-DJ-A2QB	0	0	0	1	1
Thyroid Carcinoma	TCGA-DJ-A2QC	0	0	0	1	0
Thyroid Carcinoma	TCGA-DJ-A3UK	0	0	0	1	1
Thyroid Carcinoma	TCGA-DJ-A3UM	0	0	0	1	0
Thyroid Carcinoma	TCGA-DJ-A3UN	1	0	1	1	1

Thyroid Carcinoma	TCGA-DJ-A3UO	0	0	0	1	1
Thyroid Carcinoma	TCGA-DJ-A3UP	0	0	0	1	1
Thyroid Carcinoma	TCGA-DJ-A3UQ	0	0	0	1	0
Thyroid Carcinoma	TCGA-DJ-A3UR	0	0	0	1	0
Thyroid Carcinoma	TCGA-DJ-A3US	0	0	0	0	0
Thyroid Carcinoma	TCGA-DJ-A3UT	0	0	0	1	0
Thyroid Carcinoma	TCGA-DJ-A3UU	0	0	0	1	0
Thyroid Carcinoma	TCGA-DJ-A3UW	0	0	0	1	0
Thyroid Carcinoma	TCGA-DJ-A3UX	0	0	0	1	0
Thyroid Carcinoma	TCGA-DJ-A3UY	0	0	0	1	0
Thyroid Carcinoma	TCGA-DJ-A3UZ	0	0	0	0	0
Thyroid Carcinoma	TCGA-DJ-A3V0	0	0	0	0	0
Thyroid Carcinoma	TCGA-DJ-A3V2	0	0	0	0	0
Thyroid Carcinoma	TCGA-DJ-A3V3	0	1	0	0	0
Thyroid Carcinoma	TCGA-DJ-A3V4	0	0	0	0	0
Thyroid Carcinoma	TCGA-DJ-A3V5	0	0	0	0	0
Thyroid Carcinoma	TCGA-DJ-A3V7	0	0	0	1	0
Thyroid Carcinoma	TCGA-DJ-A3V8	0	0	0	0	0
Thyroid Carcinoma	TCGA-DJ-A3V9	0	0	0	0	0
Thyroid Carcinoma	TCGA-DJ-A3VA	0	0	0	1	0
Thyroid Carcinoma	TCGA-DJ-A3VB	0	0	0	1	0
Thyroid Carcinoma	TCGA-DJ-A3VD	0	0	0	0	0
Thyroid Carcinoma	TCGA-DJ-A3VE	0	0	0	1	0
Thyroid Carcinoma	TCGA-DJ-A3VF	0	0	0	1	0
Thyroid Carcinoma	TCGA-DJ-A3VG	0	0	0	0	0
Thyroid Carcinoma	TCGA-DJ-A3VI	0	0	0	0	0
Thyroid Carcinoma	TCGA-DJ-A3VJ	1	0	0	1	0
Thyroid Carcinoma	TCGA-DJ-A3VK	0	0	0	1	1
Thyroid Carcinoma	TCGA-DJ-A3VL	0	0	0	1	0
Thyroid Carcinoma	TCGA-DJ-A3VM	0	0	0	1	1
Thyroid Carcinoma	TCGA-DJ-A4UL	0	0	0	1	0
Thyroid Carcinoma	TCGA-DJ-A4UP	0	0	0	0	0
Thyroid Carcinoma	TCGA-DJ-A4UQ	0	0	0	0	0
Thyroid Carcinoma	TCGA-DJ-A4UR	0	0	0	1	1
Thyroid Carcinoma	TCGA-DJ-A4UT	0	0	0	0	0
Thyroid Carcinoma	TCGA-DJ-A4UW	0	0	0	1	0
Thyroid Carcinoma	TCGA-DJ-A4V0	0	0	0	0	0
Thyroid Carcinoma	TCGA-DJ-A4V2	0	0	0	1	0
Thyroid Carcinoma	TCGA-DJ-A4V4	0	0	0	1	0
Thyroid Carcinoma	TCGA-DJ-A4V5	0	0	0	0	0
Thyroid Carcinoma	TCGA-DO-A1JZ	0	0	0	0	1
Thyroid Carcinoma	TCGA-DO-A1K0	0	0	0	1	0
Thyroid Carcinoma	TCGA-DO-A2HM	1	1	0	1	1
Thyroid Carcinoma	TCGA-E3-A3DY	0	0	0	1	0
Thyroid Carcinoma	TCGA-E3-A3DZ	0	0	0	1	1
Thyroid Carcinoma	TCGA-E3-A3E0	0	0	0	0	0
Thyroid Carcinoma	TCGA-E3-A3E1	0	0	0	1	0
Thyroid Carcinoma	TCGA-E3-A3E2	0	0	0	1	0
Thyroid Carcinoma	TCGA-E3-A3E3	0	0	0	1	0
Thyroid Carcinoma	TCGA-E3-A3E5	0	0	0	1	1
Thyroid Carcinoma	TCGA-E8-A242	0	0	0	1	0
Thyroid Carcinoma	TCGA-E8-A2EA	0	0	0	1	0
Thyroid Carcinoma	TCGA-E8-A2JQ	0	0	0	0	0
Thyroid Carcinoma	TCGA-E8-A3X7	0	0	0	0	0
Thyroid Carcinoma	TCGA-E8-A413	0	0	0	1	0
Thyroid Carcinoma	TCGA-E8-A414	0	0	0	0	0
Thyroid Carcinoma	TCGA-E8-A415	0	0	0	1	0
Thyroid Carcinoma	TCGA-E8-A416	0	0	0	0	0

Thyroid Carcinoma	TCGA-E8-A417	0	0	0	0	0
Thyroid Carcinoma	TCGA-E8-A418	1	0	0	1	0
Thyroid Carcinoma	TCGA-E8-A419	0	0	0	1	0
Thyroid Carcinoma	TCGA-E8-A432	1	1	1	1	1
Thyroid Carcinoma	TCGA-E8-A433	0	0	0	1	0
Thyroid Carcinoma	TCGA-E8-A434	0	0	0	1	1
Thyroid Carcinoma	TCGA-E8-A436	0	0	0	1	0
Thyroid Carcinoma	TCGA-E8-A437	0	0	0	1	0
Thyroid Carcinoma	TCGA-E8-A438	0	0	0	0	0
Thyroid Carcinoma	TCGA-E8-A44K	0	0	0	1	0
Thyroid Carcinoma	TCGA-E8-A44M	0	0	0	0	0
Thyroid Carcinoma	TCGA-EL-A3CL	0	0	0	1	1
Thyroid Carcinoma	TCGA-EL-A3CM	0	0	0	1	0
Thyroid Carcinoma	TCGA-EL-A3CN	0	0	0	1	0
Thyroid Carcinoma	TCGA-EL-A3CO	0	0	0	0	0
Thyroid Carcinoma	TCGA-EL-A3CP	0	0	0	1	0
Thyroid Carcinoma	TCGA-EL-A3CR	0	0	0	1	0
Thyroid Carcinoma	TCGA-EL-A3CS	0	0	0	1	0
Thyroid Carcinoma	TCGA-EL-A3CT	0	0	0	1	0
Thyroid Carcinoma	TCGA-EL-A3CU	0	0	0	1	0
Thyroid Carcinoma	TCGA-EL-A3CV	0	0	0	1	0
Thyroid Carcinoma	TCGA-EL-A3CW	0	0	0	1	0
Thyroid Carcinoma	TCGA-EL-A3CX	0	0	0	0	0
Thyroid Carcinoma	TCGA-EL-A3CY	0	0	0	0	0
Thyroid Carcinoma	TCGA-EL-A3CZ	0	0	0	0	0
Thyroid Carcinoma	TCGA-EL-A3D0	0	0	0	1	1
Thyroid Carcinoma	TCGA-EL-A3D1	0	0	0	1	1
Thyroid Carcinoma	TCGA-EL-A3D4	0	0	0	1	1
Thyroid Carcinoma	TCGA-EL-A3D5	1	0	1	0	1
Thyroid Carcinoma	TCGA-EL-A3D6	0	0	0	1	1
Thyroid Carcinoma	TCGA-EL-A3GO	1	0	0	1	1
Thyroid Carcinoma	TCGA-EL-A3GP	1	1	0	1	1
Thyroid Carcinoma	TCGA-EL-A3GQ	0	0	0	1	1
Thyroid Carcinoma	TCGA-EL-A3GR	0	0	0	1	0
Thyroid Carcinoma	TCGA-EL-A3GS	0	0	0	1	0
Thyroid Carcinoma	TCGA-EL-A3GU	0	0	0	1	0
Thyroid Carcinoma	TCGA-EL-A3GV	0	0	0	1	0
Thyroid Carcinoma	TCGA-EL-A3GW	0	0	0	1	1
Thyroid Carcinoma	TCGA-EL-A3GX	0	0	0	1	0
Thyroid Carcinoma	TCGA-EL-A3GY	0	0	0	1	0
Thyroid Carcinoma	TCGA-EL-A3GZ	0	0	0	1	0
Thyroid Carcinoma	TCGA-EL-A3H1	0	0	0	0	0
Thyroid Carcinoma	TCGA-EL-A3H2	1	0	1	0	1
Thyroid Carcinoma	TCGA-EL-A3H3	0	0	0	0	0
Thyroid Carcinoma	TCGA-EL-A3H4	0	0	0	1	0
Thyroid Carcinoma	TCGA-EL-A3H5	0	0	0	1	0
Thyroid Carcinoma	TCGA-EL-A3H7	0	0	0	1	0
Thyroid Carcinoma	TCGA-EL-A3H8	0	0	0	1	0
Thyroid Carcinoma	TCGA-EL-A3MW	0	0	0	1	0
Thyroid Carcinoma	TCGA-EL-A3MX	1	1	0	1	1
Thyroid Carcinoma	TCGA-EL-A3MY	0	0	0	1	0
Thyroid Carcinoma	TCGA-EL-A3MZ	0	0	0	1	0
Thyroid Carcinoma	TCGA-EL-A3N2	0	0	0	1	0
Thyroid Carcinoma	TCGA-EL-A3N3	0	0	0	1	0
Thyroid Carcinoma	TCGA-EL-A3T0	0	0	0	0	0
Thyroid Carcinoma	TCGA-EL-A3T1	0	0	0	1	0
Thyroid Carcinoma	TCGA-EL-A3T2	0	0	0	1	1
Thyroid Carcinoma	TCGA-EL-A3T3	0	0	0	1	0

Thyroid Carcinoma	TCGA-EL-A3T6	0	0	0	1	0
Thyroid Carcinoma	TCGA-EL-A3T7	0	0	0	1	0
Thyroid Carcinoma	TCGA-EL-A3T8	0	0	0	1	0
Thyroid Carcinoma	TCGA-EL-A3T9	1	1	0	0	1
Thyroid Carcinoma	TCGA-EL-A3TA	0	0	0	1	0
Thyroid Carcinoma	TCGA-EL-A3TB	0	0	0	0	0
Thyroid Carcinoma	TCGA-EL-A3ZG	0	0	0	0	0
Thyroid Carcinoma	TCGA-EL-A3ZH	0	0	0	1	0
Thyroid Carcinoma	TCGA-EL-A3ZK	1	1	0	0	1
Thyroid Carcinoma	TCGA-EL-A3ZL	0	0	0	1	1
Thyroid Carcinoma	TCGA-EL-A3ZM	0	0	1	0	1
Thyroid Carcinoma	TCGA-EL-A3ZN	0	0	0	0	0
Thyroid Carcinoma	TCGA-EL-A3ZO	1	1	0	0	1
Thyroid Carcinoma	TCGA-EL-A3ZP	0	0	0	0	0
Thyroid Carcinoma	TCGA-EL-A3ZQ	0	0	0	1	0
Thyroid Carcinoma	TCGA-EL-A3ZR	0	0	0	0	1
Thyroid Carcinoma	TCGA-EL-A3ZS	0	0	0	0	0
Thyroid Carcinoma	TCGA-EL-A3ZT	0	0	0	1	0
Thyroid Carcinoma	TCGA-EL-A4JV	0	0	0	1	1
Thyroid Carcinoma	TCGA-EL-A4JW	0	0	0	1	0
Thyroid Carcinoma	TCGA-EL-A4JX	1	0	0	1	0
Thyroid Carcinoma	TCGA-EL-A4JZ	1	0	0	1	1
Thyroid Carcinoma	TCGA-EL-A4K0	0	0	0	1	0
Thyroid Carcinoma	TCGA-EL-A4K1	0	0	0	0	0
Thyroid Carcinoma	TCGA-EL-A4K2	0	0	0	1	1
Thyroid Carcinoma	TCGA-EL-A4K4	0	0	0	1	0
Thyroid Carcinoma	TCGA-EL-A4K6	1	1	0	1	1
Thyroid Carcinoma	TCGA-EL-A4K7	0	0	0	0	0
Thyroid Carcinoma	TCGA-EL-A4K9	0	0	0	1	1
Thyroid Carcinoma	TCGA-EL-A4KD	0	0	0	0	0
Thyroid Carcinoma	TCGA-EL-A4KG	0	0	0	1	0
Thyroid Carcinoma	TCGA-EL-A4KH	0	0	0	1	0
Thyroid Carcinoma	TCGA-EL-A4KI	1	1	0	1	1
Thyroid Carcinoma	TCGA-EM-A1CS	0	0	0	1	1
Thyroid Carcinoma	TCGA-EM-A1CT	0	0	0	1	1
Thyroid Carcinoma	TCGA-EM-A1CU	0	0	0	1	0
Thyroid Carcinoma	TCGA-EM-A1CV	0	0	0	1	0
Thyroid Carcinoma	TCGA-EM-A1CW	0	0	0	0	0
Thyroid Carcinoma	TCGA-EM-A1YA	0	0	0	0	0
Thyroid Carcinoma	TCGA-EM-A1YB	1	1	0	0	1
Thyroid Carcinoma	TCGA-EM-A1YC	0	0	0	0	1
Thyroid Carcinoma	TCGA-EM-A1YD	0	0	0	0	0
Thyroid Carcinoma	TCGA-EM-A1YE	1	1	1	1	1
Thyroid Carcinoma	TCGA-EM-A22I	0	0	0	1	0
Thyroid Carcinoma	TCGA-EM-A22J	0	0	0	1	0
Thyroid Carcinoma	TCGA-EM-A22K	0	0	0	1	1
Thyroid Carcinoma	TCGA-EM-A22L	0	0	0	1	1
Thyroid Carcinoma	TCGA-EM-A22M	0	0	0	1	1
Thyroid Carcinoma	TCGA-EM-A22N	0	0	1	0	1
Thyroid Carcinoma	TCGA-EM-A22O	0	0	0	1	1
Thyroid Carcinoma	TCGA-EM-A22P	0	0	0	1	1
Thyroid Carcinoma	TCGA-EM-A22Q	0	0	0	1	0
Thyroid Carcinoma	TCGA-EM-A2CJ	0	0	0	1	1
Thyroid Carcinoma	TCGA-EM-A2CK	0	0	0	0	0
Thyroid Carcinoma	TCGA-EM-A2CL	0	0	0	0	0
Thyroid Carcinoma	TCGA-EM-A2CN	0	0	0	1	0
Thyroid Carcinoma	TCGA-EM-A2CO	0	0	1	0	1
Thyroid Carcinoma	TCGA-EM-A2CP	0	0	0	0	0

Thyroid Carcinoma	TCGA-EM-A2CQ	0	0	0	0	0
Thyroid Carcinoma	TCGA-EM-A2CR	0	0	0	0	0
Thyroid Carcinoma	TCGA-EM-A2CS	0	0	0	0	0
Thyroid Carcinoma	TCGA-EM-A2CS	0	0	0	0	0
Thyroid Carcinoma	TCGA-EM-A2CT	0	0	0	1	0
Thyroid Carcinoma	TCGA-EM-A2CU	0	0	0	0	0
Thyroid Carcinoma	TCGA-EM-A2OV	0	0	0	0	0
Thyroid Carcinoma	TCGA-EM-A2OW	0	0	0	0	0
Thyroid Carcinoma	TCGA-EM-A2OX	0	0	0	1	1
Thyroid Carcinoma	TCGA-EM-A2OY	1	1	1	1	1
Thyroid Carcinoma	TCGA-EM-A2OZ	0	0	0	1	0
Thyroid Carcinoma	TCGA-EM-A2P0	0	0	0	1	0
Thyroid Carcinoma	TCGA-EM-A2P1	0	0	0	0	0
Thyroid Carcinoma	TCGA-EM-A2P1	0	0	0	1	0
Thyroid Carcinoma	TCGA-EM-A2P2	1	0	0	1	0
Thyroid Carcinoma	TCGA-EM-A2P3	0	0	0	1	0
Thyroid Carcinoma	TCGA-EM-A3AI	0	0	0	0	0
Thyroid Carcinoma	TCGA-EM-A3AJ	0	0	0	0	0
Thyroid Carcinoma	TCGA-EM-A3AK	0	0	0	1	0
Thyroid Carcinoma	TCGA-EM-A3AL	0	0	0	0	0
Thyroid Carcinoma	TCGA-EM-A3AN	0	0	0	0	0
Thyroid Carcinoma	TCGA-EM-A3AO	0	0	0	0	0
Thyroid Carcinoma	TCGA-EM-A3AP	0	0	0	1	1
Thyroid Carcinoma	TCGA-EM-A3AQ	0	0	0	0	0
Thyroid Carcinoma	TCGA-EM-A3AR	0	0	0	1	0
Thyroid Carcinoma	TCGA-EM-A3FJ	0	0	0	1	0
Thyroid Carcinoma	TCGA-EM-A3FK	0	0	0	1	0
Thyroid Carcinoma	TCGA-EM-A3FL	0	0	0	0	0
Thyroid Carcinoma	TCGA-EM-A3FM	0	0	0	1	0
Thyroid Carcinoma	TCGA-EM-A3FN	0	0	0	0	0
Thyroid Carcinoma	TCGA-EM-A3FO	0	0	0	1	1
Thyroid Carcinoma	TCGA-EM-A3FP	0	0	0	0	0
Thyroid Carcinoma	TCGA-EM-A3FQ	0	0	0	0	0
Thyroid Carcinoma	TCGA-EM-A3FQ	0	0	0	0	0
Thyroid Carcinoma	TCGA-EM-A3FR	0	0	0	0	0
Thyroid Carcinoma	TCGA-EM-A3O3	1	1	0	1	1
Thyroid Carcinoma	TCGA-EM-A3O6	1	0	0	0	0
Thyroid Carcinoma	TCGA-EM-A3O7	0	0	0	1	0
Thyroid Carcinoma	TCGA-EM-A3O8	0	0	0	1	0
Thyroid Carcinoma	TCGA-EM-A3O9	1	1	1	1	1
Thyroid Carcinoma	TCGA-EM-A3OA	0	0	0	1	0
Thyroid Carcinoma	TCGA-EM-A3OB	0	1	1	1	1
Thyroid Carcinoma	TCGA-EM-A3ST	0	0	0	0	0
Thyroid Carcinoma	TCGA-EM-A3SU	0	0	0	0	0
Thyroid Carcinoma	TCGA-EM-A3SU	0	0	0	0	0
Thyroid Carcinoma	TCGA-EM-A3SX	0	0	0	1	1
Thyroid Carcinoma	TCGA-EM-A3SY	1	1	1	1	1
Thyroid Carcinoma	TCGA-EM-A3SZ	0	0	0	1	1
Thyroid Carcinoma	TCGA-EM-A4FF	0	0	0	0	0
Thyroid Carcinoma	TCGA-EM-A4FH	0	0	0	0	0
Thyroid Carcinoma	TCGA-EM-A4FK	0	0	0	1	0
Thyroid Carcinoma	TCGA-EM-A4FM	0	0	0	1	0
Thyroid Carcinoma	TCGA-EM-A4FN	0	0	0	0	0
Thyroid Carcinoma	TCGA-EM-A4FO	0	0	0	1	1
Thyroid Carcinoma	TCGA-EM-A4FQ	0	0	0	1	0
Thyroid Carcinoma	TCGA-EM-A4FR	0	0	0	1	1
Thyroid Carcinoma	TCGA-EM-A4FU	0	0	0	1	1
Thyroid Carcinoma	TCGA-EM-A4FV	0	0	0	1	0

Thyroid Carcinoma	TCGA-EM-A4G1	0	0	0	1	0
Thyroid Carcinoma	TCGA-ET-A25G	0	0	0	1	0
Thyroid Carcinoma	TCGA-ET-A25I	0	0	0	1	0
Thyroid Carcinoma	TCGA-ET-A25J	0	0	0	1	0
Thyroid Carcinoma	TCGA-ET-A25K	0	0	0	1	0
Thyroid Carcinoma	TCGA-ET-A25L	1	0	0	1	1
Thyroid Carcinoma	TCGA-ET-A25M	0	0	0	0	0
Thyroid Carcinoma	TCGA-ET-A25N	0	0	0	0	0
Thyroid Carcinoma	TCGA-ET-A25O	0	0	0	1	1
Thyroid Carcinoma	TCGA-ET-A25P	0	0	0	0	0
Thyroid Carcinoma	TCGA-ET-A25R	0	0	0	1	0
Thyroid Carcinoma	TCGA-ET-A2MX	0	0	1	1	0
Thyroid Carcinoma	TCGA-ET-A2MY	0	0	0	1	0
Thyroid Carcinoma	TCGA-ET-A2MZ	0	0	0	1	0
Thyroid Carcinoma	TCGA-ET-A2N0	0	0	0	1	0
Thyroid Carcinoma	TCGA-ET-A2N3	0	0	0	0	0
Thyroid Carcinoma	TCGA-ET-A2N4	0	0	0	1	1
Thyroid Carcinoma	TCGA-ET-A2N5	0	0	0	0	0
Thyroid Carcinoma	TCGA-ET-A39I	0	0	0	0	0
Thyroid Carcinoma	TCGA-ET-A39J	0	0	0	1	0
Thyroid Carcinoma	TCGA-ET-A39K	0	0	0	1	0
Thyroid Carcinoma	TCGA-ET-A39L	0	0	0	1	1
Thyroid Carcinoma	TCGA-ET-A39M	0	0	0	1	1
Thyroid Carcinoma	TCGA-ET-A39N	1	1	1	1	1
Thyroid Carcinoma	TCGA-ET-A39O	0	0	0	1	0
Thyroid Carcinoma	TCGA-ET-A39P	0	0	0	1	0
Thyroid Carcinoma	TCGA-ET-A39R	0	0	0	0	0
Thyroid Carcinoma	TCGA-ET-A39S	0	0	0	1	0
Thyroid Carcinoma	TCGA-ET-A39T	0	0	0	1	0
Thyroid Carcinoma	TCGA-ET-A3BN	0	0	0	0	0
Thyroid Carcinoma	TCGA-ET-A3BO	0	0	0	1	0
Thyroid Carcinoma	TCGA-ET-A3BP	1	0	0	1	1
Thyroid Carcinoma	TCGA-ET-A3BQ	0	0	0	1	0
Thyroid Carcinoma	TCGA-ET-A3BS	0	0	0	1	0
Thyroid Carcinoma	TCGA-ET-A3BT	0	0	0	1	0
Thyroid Carcinoma	TCGA-ET-A3BU	0	0	0	1	0
Thyroid Carcinoma	TCGA-ET-A3BV	0	0	1	1	1
Thyroid Carcinoma	TCGA-ET-A3BW	0	0	0	1	0
Thyroid Carcinoma	TCGA-ET-A3BX	0	0	0	1	0
Thyroid Carcinoma	TCGA-ET-A3DO	0	0	0	1	0
Thyroid Carcinoma	TCGA-ET-A3DP	0	0	0	1	0
Thyroid Carcinoma	TCGA-ET-A3DQ	1	0	0	0	1
Thyroid Carcinoma	TCGA-ET-A3DR	0	0	0	0	0
Thyroid Carcinoma	TCGA-ET-A3DS	0	0	0	0	1
Thyroid Carcinoma	TCGA-ET-A3DT	0	0	0	1	0
Thyroid Carcinoma	TCGA-ET-A3DU	0	0	0	1	0
Thyroid Carcinoma	TCGA-ET-A3DV	0	0	0	0	0
Thyroid Carcinoma	TCGA-ET-A3DW	0	0	0	1	0
Thyroid Carcinoma	TCGA-ET-A40P	0	0	0	1	1
Thyroid Carcinoma	TCGA-ET-A40Q	0	0	0	0	0
Thyroid Carcinoma	TCGA-ET-A40R	0	0	0	0	0
Thyroid Carcinoma	TCGA-ET-A40S	0	0	0	0	0
Thyroid Carcinoma	TCGA-ET-A40T	0	0	0	1	1
Thyroid Carcinoma	TCGA-ET-A4KN	1	0	0	0	0
Thyroid Carcinoma	TCGA-ET-A4KQ	0	0	0	0	0
Thyroid Carcinoma	TCGA-FE-A22Z	0	1	1	1	1
Thyroid Carcinoma	TCGA-FE-A230	0	0	0	1	0
Thyroid Carcinoma	TCGA-FE-A231	1	1	0	1	0

Thyroid Carcinoma	TCGA-FE-A232	0	0	0	1	0
Thyroid Carcinoma	TCGA-FE-A233	0	0	0	1	0
Thyroid Carcinoma	TCGA-FE-A234	0	0	0	1	0
Thyroid Carcinoma	TCGA-FE-A235	0	0	0	1	0
Thyroid Carcinoma	TCGA-FE-A236	0	0	0	1	0
Thyroid Carcinoma	TCGA-FE-A237	0	0	0	1	1
Thyroid Carcinoma	TCGA-FE-A238	0	1	1	0	1
Thyroid Carcinoma	TCGA-FE-A239	0	0	1	0	1
Thyroid Carcinoma	TCGA-FE-A23A	0	0	0	1	0
Thyroid Carcinoma	TCGA-FE-A3PA	0	0	0	0	0
Thyroid Carcinoma	TCGA-FE-A3PB	0	0	0	1	1
Thyroid Carcinoma	TCGA-FE-A3PC	1	0	0	1	0
Thyroid Carcinoma	TCGA-FE-A3PD	0	0	0	0	0
Thyroid Carcinoma	TCGA-FK-A3S3	0	0	0	0	0
Thyroid Carcinoma	TCGA-FK-A3SB	0	0	0	1	0
Thyroid Carcinoma	TCGA-FK-A3SD	0	0	0	0	0
Thyroid Carcinoma	TCGA-FK-A3SE	0	0	0	0	0
Thyroid Carcinoma	TCGA-FK-A3SG	1	1	0	0	1
Thyroid Carcinoma	TCGA-FK-A3SH	0	0	0	1	0
Thyroid Carcinoma	TCGA-FK-A4UB	0	0	0	0	0
Thyroid Carcinoma	TCGA-FY-A2QD	1	0	0	0	0
Thyroid Carcinoma	TCGA-FY-A3BL	0	0	0	1	0
Thyroid Carcinoma	TCGA-FY-A3I4	0	0	0	1	0
Thyroid Carcinoma	TCGA-FY-A3I5	1	1	1	1	1
Thyroid Carcinoma	TCGA-FY-A3NM	0	0	0	0	0
Thyroid Carcinoma	TCGA-FY-A3NN	0	0	0	1	0
Thyroid Carcinoma	TCGA-FY-A3NP	0	0	0	0	0
Thyroid Carcinoma	TCGA-FY-A3ON	0	0	0	1	0
Thyroid Carcinoma	TCGA-FY-A3R6	0	0	0	0	0
Thyroid Carcinoma	TCGA-FY-A3R7	0	0	0	1	1
Thyroid Carcinoma	TCGA-FY-A3R8	0	0	0	1	0
Thyroid Carcinoma	TCGA-FY-A3R9	0	0	0	1	0
Thyroid Carcinoma	TCGA-FY-A3RA	0	0	0	1	0
Thyroid Carcinoma	TCGA-FY-A3TY	0	0	0	0	0
Thyroid Carcinoma	TCGA-FY-A3W9	0	1	0	1	1
Thyroid Carcinoma	TCGA-FY-A3WA	1	0	1	1	1
Thyroid Carcinoma	TCGA-FY-A3YR	0	0	0	0	0
Thyroid Carcinoma	TCGA-FY-A40K	0	0	0	1	0
Thyroid Carcinoma	TCGA-FY-A40L	0	0	0	0	0
Thyroid Carcinoma	TCGA-FY-A40M	1	1	0	0	1
Thyroid Carcinoma	TCGA-FY-A40N	0	0	0	1	0
Thyroid Carcinoma	TCGA-FY-A4B0	1	1	1	0	1
Thyroid Carcinoma	TCGA-FY-A4B3	0	0	0	1	0
Thyroid Carcinoma	TCGA-FY-A4B4	0	0	0	1	1
Thyroid Carcinoma	TCGA-FY-A76V	0	0	0	0	0
Thyroid Carcinoma	TCGA-GE-A2C6	0	0	0	1	1
Thyroid Carcinoma	TCGA-H2-A26U	0	0	0	1	0
Thyroid Carcinoma	TCGA-H2-A2K9	0	0	0	1	0
Thyroid Carcinoma	TCGA-H2-A3RH	0	0	0	0	0
Thyroid Carcinoma	TCGA-H2-A3RI	0	0	0	1	0
Thyroid Carcinoma	TCGA-H2-A421	0	0	0	1	0
Thyroid Carcinoma	TCGA-H2-A422	0	0	0	0	0
Thyroid Carcinoma	TCGA-IM-A3EB	0	1	0	1	0
Thyroid Carcinoma	TCGA-IM-A3ED	0	0	0	1	0
Thyroid Carcinoma	TCGA-IM-A3U2	1	1	0	1	0
Thyroid Carcinoma	TCGA-IM-A3U3	0	0	0	1	1
Thyroid Carcinoma	TCGA-IM-A41Y	0	0	0	0	0
Thyroid Carcinoma	TCGA-IM-A41Z	0	0	0	1	1

Thyroid Carcinoma	TCGA-IM-A420	0	0	0	0	0
Thyroid Carcinoma	TCGA-IM-A4EB	0	0	0	0	0
Thyroid Carcinoma	TCGA-J8-A3NZ	0	0	0	1	0
Thyroid Carcinoma	TCGA-J8-A3O0	0	0	0	1	1
Thyroid Carcinoma	TCGA-J8-A3O1	0	0	0	0	0
Thyroid Carcinoma	TCGA-J8-A3O2	0	0	0	0	0
Thyroid Carcinoma	TCGA-J8-A3O2	0	0	0	0	0
Thyroid Carcinoma	TCGA-J8-A3YD	0	0	0	1	1
Thyroid Carcinoma	TCGA-J8-A3YE	0	0	0	1	1
Thyroid Carcinoma	TCGA-J8-A3YF	0	0	0	0	0
Thyroid Carcinoma	TCGA-J8-A3YG	0	0	0	0	0
Thyroid Carcinoma	TCGA-J8-A3YH	0	0	0	0	0
Thyroid Carcinoma	TCGA-J8-A3YH	0	0	0	1	1
Thyroid Carcinoma	TCGA-J8-A42S	0	0	0	0	0
Thyroid Carcinoma	TCGA-J8-A4HW	0	0	0	0	0
Thyroid Carcinoma	TCGA-J8-A4HW	0	0	0	0	0
Thyroid Carcinoma	TCGA-J8-A4HY	0	0	0	0	0
Thyroid Carcinoma	TCGA-KS-A41F	0	0	0	0	0
Thyroid Carcinoma	TCGA-KS-A41I	0	0	0	1	1
Thyroid Carcinoma	TCGA-KS-A41J	0	0	0	1	0
Thyroid Carcinoma	TCGA-KS-A41L	0	0	0	0	0
Thyroid Carcinoma	TCGA-KS-A4I1	0	0	0	0	0
Thyroid Carcinoma	TCGA-KS-A4I3	0	0	0	0	0
Thyroid Carcinoma	TCGA-KS-A4I5	0	1	0	1	0
Thyroid Carcinoma	TCGA-KS-A4I7	0	0	0	1	1
Thyroid Carcinoma	TCGA-KS-A4I9	0	0	0	1	0
Thyroid Carcinoma	TCGA-KS-A4IB	0	0	0	1	1
Thyroid Carcinoma	TCGA-KS-A4IC	0	0	0	0	0
Thyroid Carcinoma	TCGA-KS-A4ID	0	0	0	1	0
Thyroid Carcinoma	TCGA-L6-A4EP	0	0	0	1	0
Thyroid Carcinoma	TCGA-L6-A4EQ	0	0	0	0	0
Thyroid Carcinoma	TCGA-L6-A4ET	0	1	0	1	0
Thyroid Carcinoma	TCGA-L6-A4EU	0	0	0	1	0
Thyroid Carcinoma	TCGA-MK-A4N6	0	0	0	1	0
Thyroid Carcinoma	TCGA-MK-A4N7	0	0	0	1	0
Thyroid Carcinoma	TCGA-MK-A4N9	0	0	0	1	0
Thyroid Carcinoma	TCGA-MK-A84Z	0	0	0	1	1
Thyroid Carcinoma	TCGA-QD-A8IV	0	0	0	1	1
Uterine Carcinosarcoma	TCGA-N5-A4R8	0	0	0	0	0
Uterine Carcinosarcoma	TCGA-N5-A4RA	1	1	1	1	1
Uterine Carcinosarcoma	TCGA-N5-A4RD	1	1	1	1	1
Uterine Carcinosarcoma	TCGA-N5-A4RF	1	1	1	1	1
Uterine Carcinosarcoma	TCGA-N5-A4RJ	1	1	1	1	1
Uterine Carcinosarcoma	TCGA-N5-A4RM	1	1	1	1	1
Uterine Carcinosarcoma	TCGA-N5-A4RN	1	1	1	1	1
Uterine Carcinosarcoma	TCGA-N5-A4RO	1	1	1	1	1
Uterine Carcinosarcoma	TCGA-N5-A4RS	1	1	1	1	1
Uterine Carcinosarcoma	TCGA-N5-A4RT	1	1	1	1	1
Uterine Carcinosarcoma	TCGA-N5-A4RU	1	1	1	1	1
Uterine Carcinosarcoma	TCGA-N5-A4RV	1	1	1	1	1
Uterine Carcinosarcoma	TCGA-N5-A59E	1	1	1	1	1
Uterine Carcinosarcoma	TCGA-N5-A59F	1	1	1	1	1
Uterine Carcinosarcoma	TCGA-N6-A4V9	1	1	1	1	1
Uterine Carcinosarcoma	TCGA-N6-A4VC	1	1	1	1	1
Uterine Carcinosarcoma	TCGA-N6-A4VD	1	1	1	1	0
Uterine Carcinosarcoma	TCGA-N6-A4VE	1	1	1	1	1
Uterine Carcinosarcoma	TCGA-N6-A4VF	1	1	1	1	1
Uterine Carcinosarcoma	TCGA-N6-A4VG	1	1	1	1	1

Uterine Carcinosarcoma	TCGA-N7-A4Y0	1	0	0	1	1
Uterine Carcinosarcoma	TCGA-N7-A4Y5	1	1	1	1	1
Uterine Carcinosarcoma	TCGA-N7-A4Y8	1	1	1	1	1
Uterine Carcinosarcoma	TCGA-N7-A59B	1	1	1	1	1
Uterine Carcinosarcoma	TCGA-N8-A4PI	1	1	1	1	1
Uterine Carcinosarcoma	TCGA-N8-A4PL	1	1	1	1	1
Uterine Carcinosarcoma	TCGA-N8-A4PM	1	1	1	1	1
Uterine Carcinosarcoma	TCGA-N8-A4PN	1	1	1	1	1
Uterine Carcinosarcoma	TCGA-N8-A4PO	1	1	1	1	1
Uterine Carcinosarcoma	TCGA-N8-A4PP	1	1	1	1	1
Uterine Carcinosarcoma	TCGA-N8-A4PQ	1	1	1	1	1
Uterine Carcinosarcoma	TCGA-N8-A56S	1	1	1	1	1
Uterine Carcinosarcoma	TCGA-N9-A4PZ	1	1	1	1	1
Uterine Carcinosarcoma	TCGA-N9-A4Q1	1	1	1	1	1
Uterine Carcinosarcoma	TCGA-N9-A4Q3	1	1	1	1	1
Uterine Carcinosarcoma	TCGA-N9-A4Q4	1	1	1	1	1
Uterine Carcinosarcoma	TCGA-N9-A4Q7	1	1	1	1	1
Uterine Carcinosarcoma	TCGA-N9-A4Q8	1	1	1	1	1
Uterine Carcinosarcoma	TCGA-NA-A4QV	1	1	1	1	1
Uterine Carcinosarcoma	TCGA-NA-A4QW	1	1	1	1	1
Uterine Carcinosarcoma	TCGA-NA-A4QX	1	1	1	1	1
Uterine Carcinosarcoma	TCGA-NA-A4QY	1	1	1	1	1
Uterine Carcinosarcoma	TCGA-NA-A4R0	1	1	1	1	1
Uterine Carcinosarcoma	TCGA-NA-A4R1	1	1	1	1	1
Uterine Carcinosarcoma	TCGA-NA-A5I1	1	1	1	1	1
Uterine Carcinosarcoma	TCGA-ND-A4W6	1	1	1	1	1
Uterine Carcinosarcoma	TCGA-ND-A4WA	1	1	1	1	1
Uterine Carcinosarcoma	TCGA-ND-A4WC	1	1	0	1	1
Uterine Carcinosarcoma	TCGA-ND-A4WF	1	1	1	1	1
Uterine Carcinosarcoma	TCGA-NF-A4WU	1	1	1	1	1
Uterine Carcinosarcoma	TCGA-NF-A4WX	1	1	1	1	1
Uterine Carcinosarcoma	TCGA-NF-A4X2	1	1	1	1	1
Uterine Carcinosarcoma	TCGA-NF-A5CP	1	1	1	1	1
Uterine Carcinosarcoma	TCGA-NG-A4VU	1	1	1	1	1
Uterine Carcinosarcoma	TCGA-NG-A4VW	1	0	0	1	0
Uterine Carcinosarcoma	TCGA-QM-A5NM	1	1	1	1	1
Uterine Carcinosarcoma	TCGA-QN-A5NN	1	1	1	1	1
Uveal melanoma	TCGA-RZ-AB0B	1	1	0	1	1
Uveal melanoma	TCGA-V3-A9ZX	1	1	1	1	0
Uveal melanoma	TCGA-V3-A9ZY	0	0	0	0	0
Uveal melanoma	TCGA-V4-A9E5	0	0	0	0	0
Uveal melanoma	TCGA-V4-A9E7	1	1	1	1	1
Uveal melanoma	TCGA-V4-A9E8	1	1	1	1	1
Uveal melanoma	TCGA-V4-A9E9	0	0	1	0	0
Uveal melanoma	TCGA-V4-A9EA	1	0	1	1	1
Uveal melanoma	TCGA-V4-A9EC	1	0	1	0	0
Uveal melanoma	TCGA-V4-A9ED	1	0	0	1	0
Uveal melanoma	TCGA-V4-A9EE	1	1	0	1	1
Uveal melanoma	TCGA-V4-A9EF	1	0	0	1	0
Uveal melanoma	TCGA-V4-A9EH	0	0	0	0	0
Uveal melanoma	TCGA-V4-A9EI	1	1	1	1	1
Uveal melanoma	TCGA-V4-A9EJ	0	0	0	0	0
Uveal melanoma	TCGA-V4-A9EK	0	0	0	0	0
Uveal melanoma	TCGA-V4-A9EL	1	1	1	1	0
Uveal melanoma	TCGA-V4-A9EM	0	0	0	0	0
Uveal melanoma	TCGA-V4-A9EO	1	1	0	1	0
Uveal melanoma	TCGA-V4-A9EQ	1	1	0	1	0
Uveal melanoma	TCGA-V4-A9ES	1	1	0	1	1

Uveal melanoma	TCGA-V4-A9ET	0	0	1	0	0
Uveal melanoma	TCGA-V4-A9EU	1	1	1	1	1
Uveal melanoma	TCGA-V4-A9EV	1	0	0	1	1
Uveal melanoma	TCGA-V4-A9EW	1	1	1	1	0
Uveal melanoma	TCGA-V4-A9EX	1	1	1	1	1
Uveal melanoma	TCGA-V4-A9EY	1	0	0	0	0
Uveal melanoma	TCGA-V4-A9EZ	1	1	1	1	1
Uveal melanoma	TCGA-V4-A9F0	1	1	1	1	0
Uveal melanoma	TCGA-V4-A9F1	1	1	0	1	1
Uveal melanoma	TCGA-V4-A9F2	0	0	0	0	0
Uveal melanoma	TCGA-V4-A9F3	1	1	1	1	0
Uveal melanoma	TCGA-V4-A9F4	0	0	1	0	1
Uveal melanoma	TCGA-V4-A9F5	1	1	1	1	1
Uveal melanoma	TCGA-V4-A9F7	0	0	0	0	0
Uveal melanoma	TCGA-V4-A9F8	1	1	0	1	0
Uveal melanoma	TCGA-VD-A8K7	0	0	0	0	0
Uveal melanoma	TCGA-VD-A8K8	1	1	0	1	0
Uveal melanoma	TCGA-VD-A8K9	0	0	0	0	0
Uveal melanoma	TCGA-VD-A8KA	1	1	1	1	0
Uveal melanoma	TCGA-VD-A8KB	1	0	1	1	1
Uveal melanoma	TCGA-VD-A8KD	1	1	0	1	0
Uveal melanoma	TCGA-VD-A8KE	0	0	0	0	0
Uveal melanoma	TCGA-VD-A8KF	1	1	0	1	0
Uveal melanoma	TCGA-VD-A8KG	0	0	0	0	0
Uveal melanoma	TCGA-VD-A8KH	1	1	0	1	1
Uveal melanoma	TCGA-VD-A8KI	1	1	1	1	0
Uveal melanoma	TCGA-VD-A8KJ	1	1	1	1	1
Uveal melanoma	TCGA-VD-A8KK	1	0	0	1	1
Uveal melanoma	TCGA-VD-A8KL	1	1	0	1	1
Uveal melanoma	TCGA-VD-A8KM	1	1	0	1	0
Uveal melanoma	TCGA-VD-A8KN	1	0	1	1	0
Uveal melanoma	TCGA-VD-A8KO	1	0	0	0	0
Uveal melanoma	TCGA-VD-AA8M	1	1	1	0	0
Uveal melanoma	TCGA-VD-AA8N	1	1	1	1	0
Uveal melanoma	TCGA-VD-AA8O	1	1	1	1	1
Uveal melanoma	TCGA-VD-AA8P	1	1	1	1	1
Uveal melanoma	TCGA-VD-AA8Q	0	0	1	0	1
Uveal melanoma	TCGA-VD-AA8R	0	0	0	0	0
Uveal melanoma	TCGA-VD-AA8S	0	0	0	0	0
Uveal melanoma	TCGA-VD-AA8T	1	1	0	1	1
Uveal melanoma	TCGA-WC-A87T	0	0	0	0	1
Uveal melanoma	TCGA-WC-A87U	0	0	0	0	0
Uveal melanoma	TCGA-WC-A87W	1	1	1	1	1
Uveal melanoma	TCGA-WC-A87Y	1	1	0	1	0
Uveal melanoma	TCGA-WC-A880	0	0	1	0	1
Uveal melanoma	TCGA-WC-A881	1	1	1	1	1
Uveal melanoma	TCGA-WC-A882	1	1	0	1	0
Uveal melanoma	TCGA-WC-A883	1	1	1	1	1
Uveal melanoma	TCGA-WC-A884	0	1	1	0	1
Uveal melanoma	TCGA-WC-A885	1	1	1	1	1
Uveal melanoma	TCGA-WC-A888	1	1	0	1	0
Uveal melanoma	TCGA-WC-A88A	1	1	1	1	0
Uveal melanoma	TCGA-WC-AA9A	1	1	0	1	0
Uveal melanoma	TCGA-WC-AA9E	1	1	1	1	1
Uveal melanoma	TCGA-YZ-A980	1	1	0	1	0
Uveal melanoma	TCGA-YZ-A982	1	1	1	1	1
Uveal melanoma	TCGA-YZ-A983	0	0	0	0	1
Uveal melanoma	TCGA-YZ-A984	1	0	0	1	1

Uveal melanoma	TCGA-YZ-A985	1	0	1	1	0
